# Supplementary material for: School-age outcomes of children after perinatal brain injury: a systematic review and meta-analysis
Source: BMJ Paediatr Open. 2023 Jun 2;7(1):e001810. doi: 10.1136/bmjpo-2022-001810 (PMC10255042; doi:10.1136/bmjpo-2022-001810)
Supplement: Supplementary data [file bmjpo-2022-001810supp001.pdf]

**Supplement 1: databases searched**
Cochrane Central Register of Controlled Trials
EBSCO–CINAHL (Cumulative Index to Nursing and Allied Health Literature)
Google Scholar
Ovid–EMBASE
Ovid–MEDLINE
Ovid–MEDLINE E-pub ahead of print
Ovid–MEDLINE In-Process and Other Non-Indexed Citations
PubMed
Scopus
Web of Knowledge (Science Citation Index Expanded and Conference Proceedings Citation Index Science)
