# Supplementary material for: School-age outcomes of children after perinatal brain injury: a systematic review and meta-analysis
Source: BMJ Paediatr Open. 2023 Jun 2;7(1):e001810. doi: 10.1136/bmjpo-2022-001810 (PMC10255042; doi:10.1136/bmjpo-2022-001810)
Supplement: Supplementary data [file bmjpo-2022-001810supp002.pdf]

Supplement 2: Medline Ovid Search Strategy

1. exp CHILD/
2. exp Child, Preschool/
3. exp ADOLESCENT/
4. exp INFANT/ or exp INFANT, NEWBORN/
5. (child* or toddler* or baby or infant* or adolescent*).mp.
6. 1 or 2 or 3 or 4 or 5
7. exp Educational Status/
8. exp Child Development/
9. exp Learning Disorders/
10. exp Educational Measurement/
11. exp SCHOOLS/
12. exp Academic Performance/
13. school performance.mp.
14. exp COGNITION/
15. exp LEARNING/
16. exp SPATIAL LEARNING/
17. exp VERBAL LEARNING/
18. exp SOCIAL LEARNING/
19. exp Intelligence Tests/
20. exp INTELLIGENCE/
21. exp Intellectual Disability/
22. exp Neurodevelopmental Disorders/
23. neurodevelopm*.mp.
24. (nervous system dys* or CNS dys*).mp. [mp=title, abstract, heading word, drug trade name, original title, device manufacturer, drug manufacturer, device trade name, keyword, floating subheading word, candidate term word]
25. (nervous system abnorm* or CNS abnorm*).mp. [mp=title, abstract, heading word, drug trade name, original title, device manufacturer, drug manufacturer, device trade name, keyword, floating subheading word, candidate term word]
26. (nervous system malform* or CNS malform*).mp. [mp=title, abstract, heading word, drug trade name, original title, device manufacturer, drug manufacturer, device trade name, keyword, floating subheading word, candidate term word]
27. (nervous system dis* or CNS dis*).mp. [mp=title, abstract, heading word, drug trade name, original title, device manufacturer, drug manufacturer, device trade name, keyword, floating subheading word, candidate term word]
28. (mental health condi* or mental health dis*).mp. [mp=title, abstract, heading word, drug trade name, original title, device manufacturer, drug manufacturer, device trade name, keyword, floating subheading word, candidate term word]
29. mental health outcome.mp.
30. behaviour* abnorm*.mp.
31. cognitive impairment.mp. or exp Cognitive Dysfunction/
32. visual impairment.mp. or exp Vision Disorders/
33. visual develop*.mp.
34. (visual dis* or visual dys*).mp. [mp=title, abstract, heading word, drug trade name, original title, device manufacturer, drug manufacturer, device trade name, keyword, floating subheading word, candidate term word]

35. (nystagmus or strabismus).mp.
36. (visual acuity or refractive error*).mp.
37. hearing impairment.mp. or exp Hearing Loss/
38. exp Deafness/
39. exp DEAF-BLIND DISORDERS/
40. exp Hearing Loss, Sensorineural/
41. exp Movement Disorders/
42. exp Cerebral Palsy/
43. motor impairment.mp.
44. (seizure* or convulsi*).mp.
45. exp EPILEPSY/ or epilepsy.mp.
46. exp Executive Function/
47. visual-motor impairment.mp.
48. numeracy.mp.
49. literacy.mp. or exp LITERACY/
50. jaundice.mp.
51. exp Language Development Disorders/ or exp Child Language/ or language impairment.mp. or exp Reading/ or exp Dyslexia/ or reading impairment.mp.
52. 7 or 8 or 9 or 10 or 11 or 12 or 13 or 14 or 15 or 16 or 17 or 18 or 19 or 20 or 21 or 22 or 23 or 24 or 25 or 26 or 27 or 28 or 29 or 30 or 31 or 32 or 33 or 34 or 35 or 36 or 37 or 38 or 39 or 40 or 41 or 42 or 43 or 44 or 45 or 46 or 47 or 48
53. 49 or 50 or 51
54. 52 or 53
55. exp JAUNDICE, NEONATAL/
56. exp JAUNDICE/
57. exp Hyperbilirubinemia, Neonatal/
58. exp Hyperbilirubinemia/
59. hyperbilirubin*.mp.
60. exp Hyperbilirubinemia, Hereditary/
61. bilirubin encephalopathy.mp.
62. bilirubin-induced neuro*.mp.
63. exchange transfusion.mp.
64. exp ASPHYXIA NEONATORUM/
65. (exp ASPHYXIA/ or asphyxia.mp.) and neonat*.mp.
66. exp Hypoxia-Ischemia, Brain/ and neonat*.mp.
67. perinatal asphyxia.mp.
68. birth asphyxia.mp.
69. (hypoxic-ischemic encephalopathy or hypoxic-ischaemic encephalopathy).mp.
70. neonatal encephalopathy.mp.
71. (exp Cerebral Hemorrhage/ or exp Intracranial Hemorrhages/ or exp Brain Ischemia/ or intracranial haemorrhage.mp. or exp Subarachnoid Hemorrhage/ or exp Stroke/) and neonat*.mp.
72. perinatal stroke.mp.
73. (central nervous system infection.mp. or exp Central Nervous System Infections/) and neonat*.mp.
74. (exp Meningoencephalitis/ or meningo-encephalitis.mp.) and neonat*.mp.
75. (MENINGITIS/ or meningitis.mp.) and neonat*.mp.

76. exp MENINGITIS, VIRAL/ and neonat*.mp.
77. (meningoencephalitis and neonat*).mp.
78. (encephalitis.mp. or exp ENCEPHALITIS, VIRAL/ or exp INFECTIOUS ENCEPHALITIS/ or exp ENCEPHALITIS/) and neonat*.mp.
79. kernicterus.mp. or exp KERNICTERUS/
80. preterm white matter disease.mp.
81. (periventricular leukomalacia.mp. or exp Leukomalacia, Periventricular/) and neonat*.mp.
82. (therapeutic hypothermia.mp. or exp Hypothermia, Induced/) and neonat*.mp.
83. ((subdural haemorrhage or subdural hemorrhage) and neonat*).mp.
84. (exp Hematoma, Subdural/ or subdural haemorrhage.mp. or exp Craniocerebral Trauma/) and neonat*.mp.
85. (intraventricular haemorrhage and neonat*).mp.
86. (tentorial tear and neonat*).mp.
87. (parenchymal haemorrhage and neonat*).mp.
88. (ventriculoperitoneal shunt.mp. or exp Cerebrospinal Fluid Shunts/ or exp Ventriculoperitoneal Shunt/) and neonat*.mp.
89. ((ventricular drain or Rickham reservoir or CSF shunt) and neonat*).mp.
90. neonatal stroke.mp.
91. (cerebrovascular accident and neonat*).mp.
92. neonatal cerebral ischaemia.mp.
93. (exp Intracranial Thrombosis/ or cerebral venous thrombosis.mp.) and neonat*.mp.
94. (seizure.mp. or exp Seizures/) and neonat*.mp.
95. 55 or 56 or 57 or 58 or 59 or 60 or 61 or 62 or 63 or 64 or 65 or 66 or 67 or 68 or 69 or 70 or 71 or 72 or 73 or 74 or 75 or 76 or 77 or 78 or 79 or 80 or 81 or 82 or 83 or 84 or 85 or 86 or 87 or 88 or 89 or 90 or 91 or 92 or 93 or 94
96. exp Cohort Studies/
97. exp Retrospective Studies/
98. (cohort* or (case$ and control$)).tw.
99. exp Cross-Sectional Studies/
100. exp Randomized Controlled Trial/
101. 96 or 97 or 98 or 99 or 100
102. exp "REVIEW"/
103. exp Case Reports/
104. Animals/
105. animal stud*.mp.
106. 102 or 103 or 104 or 105
107. 6 and 52 and 95 and 101
108. 107 not 106
