# Supplementary material for: School-age outcomes of children after perinatal brain injury: a systematic review and meta-analysis
Source: BMJ Paediatr Open. 2023 Jun 2;7(1):e001810. doi: 10.1136/bmjpo-2022-001810 (PMC10255042; doi:10.1136/bmjpo-2022-001810)
Supplement: Supplementary data [file bmjpo-2022-001810supp003.pdf]

Supplement 3: included studies of school-aged outcomes after perinatal brain injury

* overlapping study data; Ω potential error in manuscript; Adjusted Odds Ratio (aOR); Autism spectrum Disorder (ASD); Attention Deficit Hyperactivity Disorder (ADHD); Bayley Scale of Infant Development (BSID); Child Behaviour Checklist (CBCL); Clinical Evaluation of Language Fundamentals (CELF); Cystic Periventricular leukomalacia (cPVL); Gross Motor Function Classification System, (GMFCS); Haemorrhagic parenchymal infarction (HPI); Hazard Ratio (HR); International Classification of Disease (ICD); Intraventricular haemorrhage (IVH); Intelligence Quotient (IQ); Kaufman Assessment Battery for Children (K-ABC); Mental Developmental Index (MDI); Peabody Picture Vocabulary Test (PPVT); Periventricular (PV); Periventricular leukomalacia (PVL); National Institute of Child Health and Human Development (NICHD); Neonatal Intensive Care Unit (NICU); Psychomotor Development Index (PDI); Retinopathy of Prematurity (ROP); Small for Gestational Age (SGA); Spontaneous Intestinal Perforation (SIP); Standard Deviation (SD); Standard Error (SE); Test of Motor Impairment (TOMI); Very low birthweight (VLBW); Visuomotor integration (VMI); Wechsler Abbreviated Scale of Intelligence (WASI); Wechsler Intelligence Scale for Children (WISC); Wechsler Preschool & Primary Scale of Intelligence (WPPSI); White Matter Injury (WMI); Wide Range Achievement Test (WRAT)

	Author<br>Year<br>Country<br>Study type	Population<br>Exposures<br>Comparator<br>Ascertainment/ definition	Outcomes	Main result(s)
1	Adant 2019[9]<br><br>Belgium<br><br>Retrospective cohort	**Population**<br>• Gestation ≤32 weeks with and without spontaneous intestinal perforation (SIP)<br>• Born 1994-2014<br><br>**Exposure (n=19)**<br>• IVH grade 3-4<br><br>**Comparator (n=44)**<br>• Matched on gender, gestational age, date of birth (multiples matched to sibling without SIP)<br>• No IVH<br><br>**Ascertainment/ definition**<br>• Clinical record review	**Outcomes**<br>• Functional disability (composite)<br>• Cognitive<br>• Motor<br>• Visual<br>• Behavioural/ mental health<br>• Wellbeing<br>• Quality of life<br>• Physical health<br><br>**Measurement/ assessment**<br>• BSID II<br>• Telephone survey (parents)<br>• PedsQL<br>• IQ testing<br><br>**Follow-up**<br>• 67% follow-up at 7-11 months<br>• 41% follow-up at 18-22 months<br>• 49% follow-up at 4-10 years<br>• 86% follow-up telephone survey	**Outcomes of those with SIP compared to controls without SIP – by IVH subgroup**<br><br>**Disability**<br>aOR 8.79 95%CI (1.72, 44.86)<br><br>**Multiple disabilities**<br>aOR 5.97 95%CI (1.61, 22.15)<br><br>**Cognitive**<br>**Regular education system (not a special educational needs school)**<br>aOR 8.73 95%CI (2.1, 36.72*)*<br><br>**Visual outcomes** (wearing glasses)<br>aOR 0.474 95%CI (0.13, 1.69)<br><br>**Behavioural/ mental health disorder** (including attention problems, conduct problems and autism spectrum disorders)<br>aOR 1.24 95%CI (0.32, 4.8)<br><br>**PedsQL low quality of life score**<br>aOR 0.87 95%CI (0.77, 0.99)<br><br>**PedsQL low physical health score**<br>aOR 0.82 95%CI (0.66, 1.01)
2*	Beaino 2010[68]<br><br>France<br><br>Prospective cohort	**Population**<br>• Gestation <33 weeks<br>• Born 1997<br><br>**Exposure**<br>• IVH grade 1 (n=173)<br>• IVH grade 2 (n=117)<br>• IVH grade 3 (n=32)<br>• Intraparenchymal haemorrhage (IPH) (n=6)<br>• Persistent echodensities or ventricular dilatation (n=241)<br>• cPVL (n=66)<br><br>**Comparator (n=1153)**<br>• Unmatched<br>• No IVH<br><br>**Ascertainment/ definition**<br>• Ultrasound imaging undertaken and reviewed by neonatologists or radiographers	**Outcomes**<br>• Cerebral palsy<br><br>**Measurement/assessment**<br>• Standardised questionnaires completed by physicians<br><br>**Follow-up**<br>• 5 years<br>• 77% follow-up	**Cerebral palsy**<br>**Grade 3 IVH**<br>OR 3.75 95%CI (2.41–5.85)<br><br>**Grade 3 IVH or echodensities of ventricular dilatation**<br>Model A aOR 3.25 95%CI (2.02–5.22)<br>Model B aOR 3.40 95%CI (2.07–5.60)<br>Model C aOR 3.31 95%CI (2.00–5.48)<br><br>**cPVL**<br>OR 33.41 95%CI (19.25–57.96)<br><br>**Cystic PVL or IPH**<br>Model A aOR 29.66 95%CI (16.71–52.62)<br>Model B aOR 28.41 95%CI (15.65–51.59)<br>Model C n/a
3	Brouwer 2012[18]<br><br>Netherlands<br><br>Prospective cohort	**Population**<br>• Gestation <32 weeks<br>• Born 1999-2004<br><br>**Exposure (n=32)**<br>• Post-haemorrhagic ventricular dilatation after IVH grade 3-4 requiring neurosurgical intervention<br>• No PVL<br><br>**Comparator (n=23)**<br>• Matched on gestation, birthweight, and sex<br>• No IVH<br><br>**Ascertainment/ definition**<br>• Ultrasound diagnosis<br>• Papile classification	**Outcomes**<br>• Motor<br>• Cerebral palsy<br>• Cognitive<br>• Behavioural<br><br>**Measurement/ assessment**<br>• Movement ABC<br>• GMFCS<br>• WPPSI (3rd edition Dutch version)<br>• Revisie Amsterdamse Kinder Intelligentietest<br>• Snijders Oomen Nonverbal Intelligence Test 2.5-7 – Revised<br>• CBCL<br>• Teacher Report Form<br><br>**Follow-up**<br>• 4-8 years (median 5.7)<br>• 97% follow-up	**Cerebral palsy**<br>IVH grade 3 n=0<br>IVH grade 4 n=8, 53%; all unilateral spastic cerebral palsy<br>GMFCS level 1, n=5<br>GMFCS level 2, n=2<br>GMFCS level 3, n=1<br><br>**Movement ABC motor score (for those without cerebral palsy)**<br>**Score <p 5 (definite motor problems)**<br>IVH grade 3 n=6, 26%<br>IVH grade 4 n=3, 13%<br>No IVH n=0<br><br>**Score p 5-15 (borderline motor function)**<br>IVH grade 3 (n=6; 26%)<br>IVH grade 4 (n=0; 0%)<br>No IVH (n=5; 29.4%)<br><br>**Score p> 15**<br>IVH grade 3 n=6, 26%<br>IVH grade 4 n=0, 0%<br>No IVH n=12, 70.6%<br><br>**Cognition**<br>**Wechsler intelligence test (mean ±SD)**<br>**Verbal scale**<br>IVH n=23, 97±13<br>IVH <30weeks' gestation n=16, 94±13<br>No IVH n=24, 96±13;<br><br>**Performance scale**<br>IVH, n=23, 94±16;<br>IVH <30weeks' gestation n=16, 93±15<br>No IVH n=24, 103±14;<br><br>**Production scale**

				IVH n=23, 87±22;<br>IVH <30weeks' gestation n=16, 85±24<br>No IVH n=24, 93±14<br><br>**Intelligence quotient (n; mean +/-SD)**<br>IVH grade 3 n=17; IQ 96±15;<br>IQ>85 n=13 (76.5%)<br><br>IVH IV n=15; IQ 91±10;<br>IQ >85 n=9 (64.3%)<br><br>IVH <30 weeks' gestation n=23; IQ 92±17;<br>IQ>85 n=15 (65.2%)<br><br>No IVH n=23; IQ 98±15,<br>IQ>85 n=17 (74%)<br><br>**Behavioural outcomes**<br>**CBCL parental score: mean T score ±SD, n in subclinical range (%)**<br>**Total scale**<br>IVH n=26: 48.2 ±8.4, n=3 (12%)<br>IVH <30 weeks' gestation n=20: 46.9 ±8.3, n=2 (10%)<br>No IVH <30 weeks' gestation n=23: 44.3 ±7.8, n=1 (4%)<br><br>**Internalising problem scale**<br>IVH: 49.2 ±8.9, n=5 (19%)<br>IVH <30 weeks' gestation: 28.2 ±8.4, n=3 (15%)<br>No IVH <30 weeks' gestation: 49.2 ±9.1, n=5 (21%)<br><br>**Externalizing problem scale**<br>IVH: 46.8 ±9.4, n=2 (8%)<br>IVH <30 weeks' gestation: 45.1 ±9.5, n=1 (15%)<br>No IVH < 30weeks' gestation: 43.7 ±7.5, n=0 (0%)<br><br>**TRF teachers score: mean T score ±SD, n in subclinical range (%)**<br>**Total scale**<br>IVH n=25: 54.7 ±8.7, n=6 (24%)<br>IVH <30 weeks' gestation n=19: 53.9 ±9.0, n=4 (21%)<br>No IVH <30 weeks' gestation n=22: 50.9 ±9.8, n=4 (18%)<br><br>**Internalising problem scale**<br>IVH: 53.2 ±10.8, 4 (16%)<br>IVH <30 weeks' gestation: 52.2 ±11.7, n=3 (16%)<br>No IVH <30 weeks' gestation: 52.4 ±11.4, n=7 (32%)<br><br>**Externalizing problem scale**<br>IVH: 54.3 ±6.7, 3 (12%)<br>IVH <30 weeks' gestation: 54.1 ±7.0, n=2 (11%)<br>No IVH <30 weeks' gestation: 49.7 ±7.7, n=2 (9%)<br><br>N=13 (41%) had repeated a school class, had educational help and/or attended special education
4	Campbell 2021[10]<br><br>USA<br><br>Prospective cohort study	**Population (n=858)**<br>• Gestation 23-27 weeks<br>• Born 2002-2004<br><br>**Exposure**<br>• IVH without WMI (n=124)<br>• WMI without IVH (n=30)<br>• IVH and WMI (n=63)<br><br>**Comparator (n=641)**<br>• Unmatched<br>• No IVH or WMI<br><br>**Ascertainment/ definition**<br>• Ultrasound imaging reviewed by two independent blinded radiologists<br>• WMI: parenchymal echolucency or moderate to severe ventriculomegaly on a late scan	**Outcomes**<br>• Neurocognitive development (composite)<br>• Cognitive<br>• Cerebral palsy<br>• Behavioural/ mental health<br>• Epilepsy<br>• Quality of life<br><br>**Measurement/ assessment**<br>• Differential Ability Scale II<br>• NEPSY II<br>• Neurological exam<br>• GMFCS<br>• Parental questionnaire<br>• Social Communication Questionnaire<br>• Child Symptom Inventory 4<br>• Peds QoL 4<br><br>**Follow up**<br>• 10 years<br>• 74% follow-up	**Neurodevelopmental burden**<br>**No impairments**<br>IVH and WMI n=24, 38%<br>WMI n=12, 40%<br>IVH n= 86, 69%<br>No IVH or WMI n=487, 76%<br><br>**No cognitive impairment; 1 or more of cerebral palsy, ASD, or epilepsy**<br>IVH and WMI n=4, 6%<br>WMI n=4, 13%<br>IVH n=7, 6%<br>No IVH or WMI n=26, 4%<br><br>**Cognitive**<br>**Normal cognitive function**<br>IVH and WMI n=8, 13%<br>WMI n=5, 17%<br>IVH n=41, 33%<br>No IVH or WMI n=235, 37%<br><br>**Cognitive impairment (moderate to severe)**<br>**IVH and WMI**<br>n=35, 56%<br>OR 5.01 95% CI (2.94, 8.54)<br>aOR 4.49 95% CI (2.49, 8.11)<br><br>**WMI**<br>n=14, 47%<br>OR 3.51 95% CI (1.67, 7.37)<br>aOR 5.07 95% CI (2.13, 12.02)<br><br>**IVH**<br>n=31, 25%<br>OR 1.34 95% CI (0.85, 2.1)<br>aOR 1.21 95% CI (0.73, 1.98)<br><br>**No IVH or WMI**<br>n=128. 20%<br>Reference category<br><br>**Low cognitive function**<br>IVH and WMI n=18. 30%<br>WMI n=10, 34%<br>IVH n=50, 41%<br>No IVH or WMI n=269, 43%<br><br>**Moderate cognitive impairment**<br>IVH and WMI n=17, 28%

				WMI n=7, 24%<br>IVH n=24, 20%<br>No IVH or WMI n=93, 15%<br><br>**Severe cognitive impairment**<br>IVH and WMI n=18, 30%<br>WMI n=7, 24%<br>IVH n=7, 6%<br>No IVH or WMI n=35, 6%<br><br>**Nonverbal IQ**<br>**IVH vs. No IVH or WMI**<br>Crude mean difference -3 95%CI (-6.6, 0.6)<br><br>**Full scale IQ**<br>**IVH vs No IVH or WMI**<br>Crude mean difference -2.2 95%CI (-5.7, 1.4)<br><br>**Cerebral palsy**<br>**IVH and WMI**<br>n=32, 51%<br>OR 16.85 95% CI (9.29, 30.55)<br>aOR 13.43 95% CI (7, 25.78)<br><br>**WMI**<br>n=14, 47%<br>OR 14.28 95% CI (6.48, 41.48)<br>aOR 18.63 95% CI (7.37, 47.06)<br><br>**IVH**<br>n=9, 7%<br>OR 1.28 95% CI (0.6, 2.72)<br>aOR 1.19 95% CI (0.54, 2.61)<br><br>**No IVH or WMI**<br>n=37, 6%<br>Reference category<br><br>**GMFCS>0**<br>IVH and WMI n=16, 25%<br>WMI n=10, 33%<br>IVH n=4, 3%<br>No IVH or WMI n=13, 2%<br><br>**Epilepsy**<br>**IVH and WMI**<br>n=12, 19%<br>OR 5.44 95 % CI (2.72, 10.86)<br>aOR 4.89 95% CI (2.31, 10.35)<br><br>**WMI**<br>n=8, 27%;<br>OR 6.92 95% CI (2.86, 16.75)<br>aOR 7.56 95% CI (2.85, 20.06)<br><br>**IVH**<br>n= 11, 9%;<br>OR 1.85 95% CI (0.91, 3.78)<br>aOR 1.5 95% CI (0.68, 3.3)<br><br>**No IVH or WMI**<br>n=25, 4%<br>Reference category<br><br>**Neuropsychiatric/ behavioural outcomes**<br>**ASD**<br>**IVH and WMI**<br>n=4, 6%<br>OR 0.97 95% CI (0.34, 2.79)<br>aOR 0.58 95% CI (0.19, 1.77)<br><br>**WMI**<br>n=2, 7%<br>OR 1.02 95% CI (0.23, 4.42)<br>aOR 0.74 95% CI (0.09, 5.88)<br><br>**IVH**<br>n=11, 9%<br>OR 1.39 95% CI (0.69, 2.78)<br>aOR 1.24 95% CI (0.59, 2.6)<br><br>**No IVH or WMI**<br>n=42, 7%<br>Reference category<br><br>**Social responsiveness scale** (over 65 among children with IQ >85 excluding those with ASD)<br>IVH and WMI n=5, 8%<br>WMI n=4, 13%<br>IVH n=14, 11%<br>No IVH or WMI n=62, 10%<br><br>**ADHD**<br>IVH and WMI n=13, 24%<br>WMI n=3, 10%<br><br>IVH n=31, 25%<br>OR 1.6 95% CI (1.1, 2.5)<br><br>No IVH or WMI n=97, 15%

				**Anxiety (parent-reported)**<br>IVH and WMI n=6, 10%<br>WMI n=3, 10%<br>IVH n=10, 8%<br>No IVH or WMI n=98, 15%<br><br>**Anxiety (teacher-reported)**<br>IVH and WMI n=12, 19%<br>WMI n=3, 10%<br>IVH n=14, 11%<br>No IVH or WMI n=88, 14%<br><br>**Depression (parent-reported)**<br>IVH and WMI n=7, 11%<br>WMI n=7, 23%<br>IVH n=14, 11%<br>No IVH or WMI n=100, 16%<br><br>**Depression (teacher-reported)**<br>IVH and WMI n=20, 32%<br>WMI n=7 23%<br>IVH n=18, 15%<br>No IVH or WMI n=96, 15%<br><br>**Poor quality of life (<70)**<br>IVH and WMI n=31, 49%<br>WMI n=12, 40%<br>IVH n=41, 25%<br>No IVH or WMI n=131, 20%
5	Cheong 2018[11]<br><br>Australia<br><br>Three prospective cohort studies	**Population**<br>• Gestation 22-27 weeks<br>• Born 1991-1992; 1997-1998; 2005-2006<br><br>**Exposure**<br>• IVH grade 3-4 (n=100)<br>• cPVL (n=38)<br><br>**Comparator**<br>• Unmatched<br>• No IVH grade 3-4 (n=446)<br>• No cPVL (n=508)<br><br>**Ascertainment/ definition**<br>• Not specified	**Outcomes**<br>• Survival with major disability (composite)<br>• Survival without major disability (composite)<br>• Cognitive<br>• Cerebral palsy<br>• Visual impairment (acuity less than 6/60 in better eye)<br>• Hearing impairment (requiring hearing aid or cochlear amplification)<br>**Assessment/ measurement**<br>• GMFCS<br>• WISC III<br>• WISC IV<br>• Differential Abilities Scales 2nd edition<br>**Follow-up**<br>• 8 years<br>• 91% follow-up of survivors	**Survival with major disability**<br>**IVH grade 3-4**<br>OR 2·98 95% CI (1·34, 6·63) p=0.01<br>aOR 2·61 95%CI (1·11–6·15) p=0·028<br><br>1997 and 2005 cohort only:<br>OR 4·01 95% CI (1·25, 12·84) p=0.02<br><br>**cPVL**<br>OR 8·11 95% CI (3·24, 20·30) p<0.001<br>aOR 9·17 95% CI (3·57–23·53) p<0·0001<br><br>1997 and 2005 cohort only<br>OR 17·0 95% CI (4·19, 69·02) p<0·001
6	Chou 2020[69]<br><br>Taiwan<br><br>Retrospective cohort study	**Population**<br>• Preterms infants <37 weeks' gestation (n=21,474)<br>• Infants born small for gestational age (n=2206)<br>• Born 2000-2010<br><br>**Exposure**<br>• Preterm with cerebral haemorrhage<br>• SGA with cerebral haemorrhage<br><br>**Comparator (n=94,720)**<br>• Matched 1:4 on gender, urbanisation of residential area and parental occupation<br>• No cerebral haemorrhage<br><br>**Ascertainment/ definition**<br>• National children's medical record database<br>• ICD 9 codes	**Outcome**<br>• Epilepsy<br><br>**Assessment/ measurement**<br>• ICD 9<br><br>**Follow-up**<br>• 2-12 years (mean 9 years)<br>• Completeness of follow-up not specified	**Epilepsy**<br>**Preterm with cerebral haemorrhage**<br>HR 42.4 95%CI (29.8, 60.3)<br>aHR 42.5 95 %CI (29.6, 60.5)<br><br>**SGA with cerebral haemorrhage**<br>HR 39.3 95%CI (5.51, 274.5)<br>aHR 38.7 95%CI (5.43, 275.5)
7	Davidovitch 2020[29]<br><br>Israel<br><br>Retrospective cohort study	**Population (n=4963)**<br>• VLBW infants ≤1500g<br>• Born 1999-2012<br><br>**Exposure**<br>• IVH grade 3-4 (n=256)<br>• PVL (n=200)<br>• Post-haemorrhagic hydrocephalus (n=152)<br><br>**Comparator**<br>• Unmatched<br>• No IVH grade 3-4 (n=4600)<br>• No PVL (n=3813)<br>• No post-haemorrhagic hydrocephalus (n=4810)<br><br>**Ascertainment/ definition**<br>• Israel national very low birthweight infant database linked to electronic medical records.<br>• Ultrasound diagnosis<br>• Papile classification	**Outcome**<br>• ASD<br><br>**Assessment/ measurement**<br>• Physical, neurological, and developmental assessment (by a qualified healthcare professional)<br>• Independent psychological assessment<br><br>**Follow-up**<br>• 8- 15 years (median 11.6)<br>• Only those linked to electronic medical records included	**ASD**<br>IVH n=10, 3.9%<br>No IVH n=103, 2.2% p=0.085<br><br>PVL n=5, 2.5%<br>No PVL n=88, 2.3% p=0.86<br><br>Post-haemorrhagic hydrocephalus n=7, 4.6%<br>No post-haemorrhagic hydrocephalus n=106, 2.2% p=0.051<br><br>IVH, PVL, post-haemorrhagic hydrocephalus or ROP n=27,23.9%<br>No brain injury n=571, 11.8% p<0.0001<br>aOR 1.62 95% CI (0.96–2.73)
8	Doyle 2000[70]<br><br>Australia	**Population**<br>• Birthweight 500–1499 g<br>• Born 1980-1981; 1992	**Outcomes**<br>• Survival<br>• Cerebral palsy	**Cerebral Palsy**<br><br>Grade of IVH

	Prospective Cohort	**Exposure**<br>**1980s epoch**<br>• IVH grade 1 (n=18)<br>• IVH grade 2 (n=9)<br>• IVH grade 3 (n=7)<br>• IVH grade 4 (n=4)<br><br>**1992 epoch**<br>• IVH grade 1 (n=23)<br>• IVH grade 2 (n=10)<br>• IVH grade 3 (n=9)<br>• IVH grade 4 (n=1)<br><br>**Comparator**<br>• Unmatched<br>• No intracranial haemorrhage (n=223)<br>• 1980s epoch (n=110)<br>• 1992 epoch (n=113)<br><br>**Ascertainment/ definition**<br>• Ultrasound imaging<br>• Post-mortem examination<br>• Papile classification	**Measurement/assessment**<br>• Clinical assessment by blinded paediatricians<br>• Functional assessment<br><br>**Follow-up**<br>• 5 years<br>• 93% follow-up for 1980s epoch<br>• 94% follow-up for 1992 epoch	**1980s epoch**<br>No IVH n=5, 5%<br>IVH grade 3 n=2, 29%<br>IVH grade 4 n=0<br><br>**1992s epoch**<br>No IVH n=4, 4%<br>IVH grade 3 n=3, 33%<br>IVH grade 4 n=1, 100%
9	Hintz 2018[17]<br><br>USA<br><br>Retrospective cohort	**Population**<br>• Gestation 24-28 weeks<br>• Born 2005-2009<br><br>**Exposure**<br><u>MRI</u><br>• Mild WMI (n=223)<br>• Moderate WMI (n=51)<br>• Severe WMI (n=15)<br><br>• Any cerebellar lesion (n=57)<br><br>• Significant cerebellar lesion (n=39)<br><br><u>Early cranial ultrasound</u><br>• No IVH 3-4 or cPVL (n=341)<br>• IVH 3-4 or cPVL (n=32)<br><br><u>Late cranial ultrasound</u><br>• No porencephalic cyst, cPVL, moderate to severe ventricular enlargement or shunt (n=354)<br>• Porencephalic cyst, cPVL, moderate to severe ventricular enlargement or shunt (n=19)<br><br>**Comparator**<br>• No white matter injury on MRI (n=84)<br>• No cerebellar lesion on MRI (n=316)<br>• No IVH 3-4 or cPVL (n=32)<br>• Normal early cranial ultrasound (n=227)<br>• No porencephalic cyst, cPVL moderate to severe ventricular enlargement or shunt (n=19)<br>• Normal late cranial ultrasound (n=284)<br><br>**Ascertainment/ definition**<br>• NICHD neonatal research network (NEURO study and SUPPORT cohort)<br>• Two masked central imaging readers for all cranial ultrasounds and one for MRI<br>• All had cranial ultrasound and MRI (at 35-42 weeks)<br>• Unilateral and bilateral cranial ultrasound lesions combined	**Outcomes**<br>• Moderate to severe disability (composite)<br>• Minimal or no disability<br>• Cognitive<br>• Cerebral palsy<br>• Hearing<br>• Vision<br><br>**Measurement/ assessment**<br>• WISC IV<br>• Neurological exam<br>• GMFCS<br>• Clinical examination<br>• Parental report<br><br>**Follow-up**<br>• 6-7 years<br>• 83.3% follow-up of survivors	<u>White matter injury</u><br>**Moderate to severe disability**<br>No white matter injury, n=8, 9%<br>Mild white matter injury, n=27, 12%<br>Moderate white matter injury, n=8, 15%<br>Severe white matter injury, n=14, 82%<br>p<0.0001<br><br>Moderate or severe white matter injury<br>aOR 1.1 95% CI (0.42, 2.92)<br><br>**Minimal or no disability**<br>No white matter injury, n=47, 55%<br>Mild white matter injury, n=88, 224%<br>Moderate white matter injury, n=15, 28%<br>Severe white matter injury, n=0, 0%<br>p<0.0001<br><br>**Cognitive impairment (FSIQ mean (SD))**<br>No white matter injury, 90.1 (15.5)<br>Mild white matter injury, 85.9 (16.8)<br>Moderate white matter injury, 84 (17)<br>Severe white matter injury, 62.7 (19.6)<br>p<0.0001<br><br>**Cognitive impairment FSIQ <70**<br>No white matter injury, n=7, 8%<br>Mild white matter injury, n=25, 11%<br>Moderate white matter injury, n=6, 12%<br>Severe white matter injury, n=9, 60%<br>p<0.0001<br><br>Moderate or severe white matter injury<br>aOR 1.14 95% CI (0.39, 3.26)<br><br>**Cognitive impairment FSIQ <85**<br>No white matter injury, n=27, 32%<br>Mild white matter injury, n=100, 45%<br>Moderate white matter injury, n=29, 57%<br>Severe white matter injury, n=13, 87%<br>p<0.0001<br><br>**No cognitive impairment FSIQ ≥85**<br>No white matter injury, n=57, 68%<br>Mild white matter injury, n=123, 55%<br>Moderate white matter injury, n=22, 43%<br>Severe white matter injury, n=2, 13%<br>p<0.0001<br><br>**Any cerebral palsy**<br>No white matter injury, n=2, 2%<br>Mild white matter injury, n=6, 3%<br>Moderate white matter injury, n=4, 7%<br>Severe white matter injury, n=10, 59%<br>p<0.0001<br><br>**Cerebral palsy with GMFCS ≥2**<br>No white matter injury, n=0, 0%<br>Mild white matter injury, n=1, 0%<br>Moderate white matter injury, n=1, 2%<br>Severe white matter injury, n=4, 24%<br>p<0.0001<br><br><u>Cerebellar lesions</u><br>**Moderate to severe disability**<br>No cerebellar lesion, n=37, 12%<br>Any cerebellar lesion, n=20, 33% p<0.0001<br>Significant cerebellar lesion, n=15, 36%<br><br>Significant cerebellar lesions<br>aOR 2.71 95% CI (1.09, 6.71)<br><br>**Minimal or no disability**

				No cerebellar lesion, n=135, 42%<br>Any cerebellar lesion n=15, 25% p<0.0001<br>Significant cerebellar lesion, n=15, 36%<br><br>**Cognitive impairment (FSIQ mean (SD))**<br>No cerebellar lesion, 87 (16.5)<br>Any cerebellar lesion 78.4 (20) p=0.001<br>Significant cerebellar lesion 76.8 (20.4)<br><br>**Cognitive impairment FSIQ <70**<br>No cerebellar lesion, n=32, 10%<br>Any cerebellar lesion, n=15, 26% p=0.001<br>Significant cerebellar lesion, n=10, 26%<br><br>Significant cerebellar lesions<br>aOR 1.96 95% CI (0.72, 5.36)<br><br>**Cognitive impairment FSIQ <85**<br>No cerebellar lesion, n=136, 43%<br>Any cerebellar lesion, n=33, 58% p=0.038<br>Significant cerebellar lesion, n=22, 56%<br><br>**No cognitive impairment FSIQ ≥85**<br>No cerebellar lesion, n=180, 57%<br>Any cerebellar lesion, n=24, 42% P=0.038<br>Significant cerebellar lesion, n=17, 44%<br><br>**Any cerebral palsy**<br>No cerebellar lesion, n=13, 4%<br>Any cerebellar lesion, n=9, 15% p=0.001<br>Significant cerebellar lesion, n=9, 21%<br><br>**Cerebral palsy with GMFCS ≥2**<br>No cerebellar lesion, n=3, 1%<br>Any cerebellar lesion, n=3, 5% p=0.19<br>Significant cerebellar lesion, n=3, 7%<br><br>**Early cranial ultrasound abnormalities**<br>**Moderate to severe disability**<br>No IVH 3-4 or cPVL, n=43, 12%<br>IVH 3-4 or cPVL, n=14, 42% p<0.0001<br>Normal scan, n=35, 12%<br>aOR 0.61 95% CI (0.14, 2.59)<br><br>**Minimal or no disability**<br>No IVH 3-4 or cPVL, n=143, 41%<br>IVH 3-4 or cPVL, n=7, 21% p<0.0001<br>Normal scan, n=120, 43%<br><br>**Cognitive impairment, FSIQ mean (SD)**<br>No IVH 3-4 or cPVL, 86.4 (17)<br>IVH 3-4 or cPVL, 77.9 (19.1) p=0.008<br>Normal scan, 86 (16.7)<br><br>**Cognitive impairment FSIQ <70**<br>No IVH 3-4 or cPVL, n=38, 11%<br>IVH 3-4 or cPVL, n=9, 28% p=0.006<br>Normal scan, n=31, 11%<br>aOR 0.42 95% CI (0.07, 2.33)<br><br>**Cognitive impairment FSIQ <85**<br>No IVH 3-4 or cPVL, n=149, 44%<br>IVH 3-4 or cPVL, n=20, 63% p=0.041<br>Normal scan, n=123, 44%<br><br>**No cognitive impairment FSIQ ≥85**<br>No IVH 3-4 or cPVL, n=192, 56%<br>IVH 3-4 or cPVL, n=12, 38% p=0.041<br>Normal scan, n=154, 56%<br><br>**Any cerebral palsy**<br>No IVH 3-4 or cPVL, n=149, 44%<br>IVH 3-4 or cPVL, n=20, 63% p=0.041<br>Normal scan, n=123, 44%<br><br>**Cerebral palsy with GMFCS ≥2**<br>No IVH 3-4 or cPVL, n=3, 1%<br>IVH 3-4 or cPVL, n=3, 9% p<0.0001<br>Normal scan, n=2, 1%<br><br>**Late cranial ultrasound abnormalities**<br>**Moderate to severe disability**<br>No porencephalic cyst, cPVL, moderate to severe ventricular enlargement or shunt, n=40, 11%<br>Porencephalic cyst, cPVL, moderate to severe ventricular enlargement or shunt, n=17, 77% p<0.0001<br>Normal scan, n=27, 10%<br>aOR 27.85 95% CI (6.03, 128.68)<br><br>**Minimal or no disability**<br>No porencephalic cyst, cPVL, moderate to severe ventricular enlargement or shunt, n=149, 42%<br>Porencephalic cyst, cPVL, moderate to severe ventricular enlargement or shunt, n=1, 5% P<0.0001<br>Normal scan, n=117, 43%<br><br>**Cognitive impairment (FSIQ mean (SD))**<br>No porencephalic cyst, cPVL, moderate to severe ventricular enlargement or shunt, 86.7 (16.7)<br>Porencephalic cyst, cPVL, moderate to severe ventricular enlargement or shunt, 65.9 (18.7) P<0.0001

				Normal scan, 87 (16.1)<br><br>**Cognitive impairment FSIQ <70**<br>No porencephalic cyst, cPVL, moderate to severe ventricular enlargement or shunt, n=36, 10%<br>Porencephalic cyst, cPVL, moderate to severe ventricular enlargement or shunt, n=11, 58% p<0.0001<br>Normal scan, n=24, 9%<br>aOR 20.05 95% CI (3.63, 110.84)<br><br>**Cognitive impairment FSIQ <85**<br>No porencephalic cyst, cPVL, moderate to severe ventricular enlargement or shunt, n=153, 43%<br>Porencephalic cyst, cPVL, moderate to severe ventricular enlargement or shunt, n=16, 84% p<0.0001<br>Normal scan, n=118, 43%<br><br>**No cognitive impairment FSIQ ≥85**<br>No porencephalic cyst, cPVL, moderate to severe ventricular enlargement or shunt, n=201, 57%<br>Porencephalic cyst, cPVL, moderate to severe ventricular enlargement or shunt, n=3, 16% p<0.0001<br>Normal scan, n=156, 57%<br><br>**Any cerebral palsy**<br>No porencephalic cyst, cPVL, moderate to severe ventricular enlargement or shunt, n=10, 3%<br>Porencephalic cyst, cPVL, moderate to severe ventricular enlargement or shunt, n=12, 50% p<0.0001<br>Normal scan, n=6, 2%<br><br>**Cerebral palsy with GMFCS ≥2**<br>No porencephalic cyst, cPVL, moderate to severe ventricular enlargement or shunt, n=2, 1%<br>Porencephalic cyst, cPVL, moderate to severe ventricular enlargement or shunt, n=4, 17% p<0.0001<br>Normal scan, n=1, 0%
10	Hirovonen, 2017[22]<br><br>Finland<br><br>Retrospective cohort	**Population**<br>• Gestation >22 weeks<br>• Birth weight >500g<br>• Born 1991-2008<br><br>**Exposure (n=557)**<br>• Intracranial haemorrhage<br><br>**Comparison (n=708,977)**<br>• No intracranial haemorrhage<br>• ICD code<br><br>**Ascertainment/ definition**<br>• Finnish national register<br>• ICD codes	**Outcomes**<br>• Cognitive<br><br>**Measurement/ assessment**<br>• ICD 9 and 10 codes<br>• BSID 1993<br>• Finnish WISC<br><br>**Follow-up**<br>• 7 years<br>• 98% follow-up	**Any intellectual disability after intracranial haemorrhage (HR (95%CI); p-value)**<br>Very preterm infants 2.92 (1.58–5.41); p= 0.001<br>Moderately preterm 5.59 (1.57–19.9); p= 0.008<br>Late preterm 4.58 (1.36–15.4); p= 0.014<br>Term 2.94 (1.08-8); p=0.035
11	Hollebrandse 2021[19]<br><br>Australia<br><br>Retrospective cohort	**Population**<br>• Gestation <28 weeks<br>• Born 1991-1992, 1997, 2005<br><br>**Exposure**<br>• IVH grade 1 n=80<br>• IVH grade 2 n=53<br>• IVH grade 3 n=23<br>• IVH grade 4 n=12<br><br>**Comparator**<br>• Unmatched<br>• Preterm infants without IVH n=331<br><br>**Ascertainment/ definition**<br>• Ultrasound diagnosis<br>• Worst grade of IVH<br>• Papile classification	**Outcomes**<br>• Cognitive<br>• Motor<br>• Cerebral palsy<br><br>**Assessment/ measurement**<br>• WISC III (1991-1992 cohort)<br>• WISC IV (1997 cohort)<br>• Differential Abilities Scale 2nd edition (2005 cohort)<br>• WRAT III (1991-92; 1997 cohorts)<br>• WRAT IV (2005 cohort)<br>• Behaviour rating inventory of executive functioning (parent-completed)<br>• Movement ABC 1st edition (1991-1992 and 1997 cohorts)<br>• Movement ABC 2nd edition (2005 cohort)<br>• GMFCS (1997 and 2005 cohort)<br>• Blinded assessment<br><br>**Follow-up**<br>• 8 years<br>• Follow-up 85-91.4%	**Cognitive**<br>**IQ score <-2 SD**<br>IVH grade 4 n=5, 42% p=0.08 (X^2 trend)<br>IVH grade 3 n=5, 22%<br>No IVH n=41, 12%<br><br>IVH 3-4: OR 2.68 95% CI (1.21, 5.94) p=0.01<br><br>**Impaired executive function**<br>**Global executive composite ≥65**<br>IVH grade 4 n=2, 18% p=0.78 (X^2 trend)<br>IVH grade 3 n=4, 18%<br>No IVH n=49, 16%<br><br>IVH 3-4: OR 1.17 95% CI (0.46, 2.97) p=0.75<br><br>**Behavioural regulation index ≥65**<br>IVH grade 4 n=2, 18% p=0.21 (X^2 trend)<br>IVH grade 3 n=6, 27%<br>No IVH n=46, 15%<br><br>IVH 3-4: OR 1.76 95% CI (0.75, 4.11) p=0.2<br><br>**Metacognition index ≥65**<br>IVH grade 4 n=3, 27% p=0.1 (X^2 trend)<br>IVH grade 3 n=5, 23%<br>No IVH n=48, 16%<br><br>IVH 3-4: OR 1.73 95% CI (0.74, 4.06) p=0.21<br><br>**Impaired academic skills (any academic skill <-2SD)**<br>IVH grade 4 n=7, 64% p<0.001 (X^2 trend)<br>IVH grade 3 n=5, 24%<br>No IVH n=50, 16%<br><br>IVH 3-4: OR 2.91 95% CI (1.35, 6.27) p=0.006<br><br>**Impaired reading <-2SD**<br>IVH grade 4 n=6, 55% p=0.002 (X^2 trend)<br>IVH grade 3 n=4, 19%<br>No IVH n=21, 10%<br><br>IVH 3-4: OR 3.62 95% CI (1.59, 8.24) p=0.002<br><br>**Impaired spelling <- 2 SD**<br>IVH grade 4 n=5, 45% p=0.011 (X^2 trend)<br>IVH grade 3 n=3, 14%

				No IVH n=21, 7%<br><br>IVH 3-4: OR 4.48 95% CI (1.8, 11.2) p=0.001<br><br>**Impaired arithmetic < -2 SD**<br>IVH grade 4 n=5, 45% p=0.09 (X^2 trend)<br>IVH grade 3 n=4, 19%<br>No IVH n=38, 12%<br><br>IVH 3-4: OR 2.79 95% CI (1.2, 6.48) p=0.017<br><br>**Motor and cerebral palsy**<br>**Any motor dysfunction (cerebral palsy or MABC <5th centile)**<br>IVH grade 4 n=11, 92% p<0.001 (X^2 trend)<br>IVH grade 3 n=10, 43%<br>No IVH n=81, 24%<br><br>IVH 3-4: OR 4.45 95% CI (2.18, 9.08) p<0.001<br><br>**Cerebral palsy**<br>IVH grade 4 n=9, 75% p<0.001(X^2 trend)<br>IVH grade 3 n=6, 26%<br>No IVH n=26, 8%<br><br>IVH 3-4: OR 8.8 95% CI (4.03, 19.2) p<0.001<br><br>**MABC <5th percentile (for the 2005 cohort)**<br>IVH grade 4 n=11, 92% p<0.001 (X^2 trend)<br>IVH grade 3 n=9, 45%<br>No IVH n=79, 26%<br><br>IVH 3-4: OR 4.7 95% CI (2.21, 9.97) p<0.001
12	Hreinsdottir 2018[48]<br><br>Sweden<br><br>Prospective cohort study	**Population**<br>• Born 2004-2007<br>• Gestation <32 years<br><br>**Exposure (n=9)**<br>• IVH grade 3-4 and/ or PVL<br><br>**Comparator (n=99)**<br>• Unmatched<br>• No IVH grade 3-4 or PVL<br><br>**Ascertainment/ definition**<br>• Ultrasound imaging performed by paediatric radiologist<br>• Papile classification for IVH<br>• PVL defined by size, laterality and as cystic of diffuse	**Outcomes**<br>• Visual impairment<br><br>**Assessment/ measurement**<br>• Linear visual acuity (Lea Hyvarinen chart)<br>• Cover test<br>• Refraction<br><br>**Follow-up**<br>• 6.5 years<br>• 78% follow-up	**Vision**<br>**Subnormal visual acuity**<br>IVH 3-4 and or PVL<br>OR 1.11 95% CI (0.25, 4.83) p=0.891<br><br>**Contrast sensitivity**<br>IVH 3-4 and or PVL<br>OR 1.87 95% CI (0.43, 8.17) p=0.403<br><br>**Refractive error**<br>IVH 3-4 and or PVL<br>OR 2.5 95% CI (0.55, 11.41) p=0.237<br><br>**Manifest strabismus**<br>IVH 3-4 and or PVL<br>OR 4 95% CI (0.65, 24.55) p=0.134<br><br>**Composite score 1: Visual acuity with both eyes of less than 0.3, significant refractive error in the better eye and manifest strabismus**<br>IVH 3-4 and or PVL<br>OR 3.63 95% CI (0.86, 15.41) p=0.08<br>aOR 4.95 95% CI (0.65, 37.48) p=0.121<br><br>**Composite score 2: Visual acuity in worse eye of less than 0.3, significant refractive error in worse eye according and manifest strabismus**<br>IVH 3-4 and or PVL<br>OR 5.67 95% CI (1.34, 24.07) p=0.019<br>aOR 10.4 95% CI (1.23, 88) p=0.032<br><br>**Composite score 3: Visual acuity with both eyes of less than 0.5, significant refractive error in the better eye, manifest strabismus, negative stereopsis and contrast sensitivity less than 0.4**<br>IVH 3-4 and or PVL<br>OR 7.6 95% CI (1.7, 34) p=0.008<br>aOR 18.19 95% CI (2.15, 154.05) p=0.008<br><br>**Composite score 4: Visual acuity with both eyes of 0.8 or less, significant refractive error in the better eye, manifest strabismus, negative stereopsis and CS less than 0.5**<br>IVH 3-4 and or PVL<br>OR 4.63 95% CI (0.9, 23.85) p=0.067<br>a6.23 95% CI (1.15, 33.83) p=0.034
13	Jansen 2020[23]<br><br>Netherlands<br><br>Prospective cohort study	**Population**<br>• Gestation <32 weeks<br>• Admitted 2006-2007<br><br>**Exposure**<br>• Mild WMI (n=18)<br>• Moderate WMI (n=14)<br>• Severe WMI (n=8)<br>• Mild cerebellar injury (n=11)<br>• Moderate cerebellar injury (n=4)<br>• Severe cerebellar injury (n=6)<br><br>**Comparator**<br>• Unmatched<br>• No WMI (n=46)<br>• No cerebellar injury (n=65)<br><br>**Ascertainment/ definition**<br>• Ultrasound imaging and term MRI<br>• Imaging reviewed by two blinded experienced investigators (neonatologists or radiologists)	**Outcomes**<br>• Cognitive<br><br>**Assessment/ measurement**<br>• National standardised achievement tests<br><br>**Follow-up**<br>• 9-10 years<br>• 77% follow-up	**Cognitive**<br>**Reading comprehension**<br>Moderate-severe WMI vs. no injury<br>B 0.241 p=0.483<br><br>Moderate-severe cerebellar injury vs. no injury<br>B 0.799 p=0.325<br><br>**Spelling**<br>Moderate-severe WMI vs. no injury<br>B 1.076 p=0.075<br><br>Moderate-severe cerebellar injury vs. no injury<br>B 1.293 p= 0.115<br><br>**Mathematics**<br>Moderate-severe WMI vs. no injury<br>B 1.856 p=0.003<br><br>Moderate-severe cerebellar injury vs. no injury<br>B 1.504 p=0.088

14	Kaur 2020[32]<br><br>Canada<br><br>Retrospective cohort study	**Population**<br>• Preterm and term infants<br>• Born 2006-2016<br><br>**Exposure**<br>• IVH grade 1 (n=811)<br>• IVH grade 2 (n=186)<br>• IVH grade 3-4 (n=194)<br>• Preterm haemorrhage (n=1139)<br><br>**Comparator**<br>• Unmatched<br>• No IVH (n=793, 062)<br>• Preterm no haemorrhage (n=50, 185)<br><br>**Ascertainment/ definition**<br>• ICD 10 codes (based on ultrasound or MRI imaging)<br>• Papile classification	**Outcome**<br>• Reason for hospitalisation<br><br>**Assessment/ measurement**<br>• ICD 10 codes<br><br>**Follow-up**<br>• 12 years<br>• Completeness of follow-up not specified	**Incidence of hospitalisation for:**<br>**Cerebral palsy, n, incident rate per 1,000 person years (95%CI)**<br>IVH n=57, 6.8 (5.3, 8.8)<br>No haemorrhage n=432, 0.1 (0.1, 0.1)<br>Hazard ratio: 4.78 95% CI (3.21, 7.13)<br><br>IVH grade 3-4 n=24 HR 14.78 95% CI (8.72-25.06)<br><br>**Ophthalmologic, n, incident rate per 1,000 person years (95%CI)**<br>IVH n=91 11.1 (9, 13.6)<br>No haemorrhage n=6773, 1.2 (1.2, 1.3)<br>HR 3.01 95% CI (2.32, 3.89)<br><br>IVH grade 3-4 n=32 HR 7.87 95% CI (5.31-11.67)<br><br>**Otologic n, incident rate per 1,000 person years (95%CI)**<br>IVH n=328, 46.7 (41.9, 52)<br>No haemorrhage n=102,153 22.1 (22, 22.2)<br>HR 1.19 95% CI (1.06, 1.34)<br><br>IVH grade 3-4 n=202 HR 1.07 95% CI (0.79-1.46)
15	Kiechl-Kohlendorfer 2013[28]<br><br>Austria<br><br>Prospective cohort	**Population**<br>• Gestation <32 weeks<br>• Born 2003-2006<br><br>**Exposure**<br>• Intracranial haemorrhage (all grades) (n=24)<br>• Intracranial haemorrhage grade 3-4 (n=4)<br>• PVL (n=2)<br>• Intraparenchymal echodense lesions (n=2)<br><br>**Comparator**<br>• Unmatched<br><br>**Ascertainment/ definition**<br>• Ultrasound imaging<br>• Papile classification	**Outcomes**<br>• Cognitive<br><br>**Measurement/assessment**<br>• Physical examination<br>• Hannover-Wechsler Intelligence Test for preschool children, third edition<br>• WPPSI<br>• Snijders-Oomen Nonverbal Intelligence Test<br>• TEDI-MATH<br><br>**Follow-up**<br>• 5 years<br>• 72.2% follow-up	**Delayed numerical skills**<br>Intracranial haemorrhage (all grades) n=11, 40,7%<br>aOR 4.66 95% CI (1.56, 13.93) p=0.007<br><br>Intracranial haemorrhage grade 3-4 n=3, 11.1%<br>PVL n=2, 7.4%<br>Intraparenchymal echodense lesions n=0
16	Klebermass-Schrehof 2012[20]<br><br>Austria<br><br>Prospective cohort	**Population**<br>• Gestation <32 weeks<br>• Admitted to NICU 1994-2005<br><br>**Exposure**<br>• IVH grade 1 (n=37)<br>• IVH grade 2 (n=84)<br>• IVH grade 3 (n=18)<br>• IVH grade 4 (n=12)<br><br>**Comparator (n=320)**<br>• Unmatched<br>• No IVH<br><br>**Ascertainment/ definition**<br>• Ultrasound diagnosis<br>• Most severe scan used<br>• Papile classification	**Outcomes**<br>• Neurosensory impairment (composite)<br>• Motor<br>• Cerebral palsy<br>• Language<br>• Visual<br>• Hearing<br><br>**Measurement/assessment**<br>• BSID II (MDI, PDI)<br>• K-ABC<br>• Beery-Buktenica Developmental Test of VMI<br>• Clinical assessment<br><br>**Follow-up**<br>• 5 years (1 ,2, and 3.5 years)<br>• Only those with follow-up included (loss to follow-up not specified)	**Outcomes at 5.5 years**<br><br>**Group 1: infants born < 28 weeks' gestation**<br>**KABC <70**<br>No IVH, 7.6%<br>IVH grade 3, 33.3%<br>IVH grade 4, 50%<br><br>**KABC mean (SD)**<br>No IVH, 91.5 (15.1)<br>IVH grade 3, 88.6 (11.1) p=not significant<br>IVH grade 4, 88.5 (10.6) p= not significant<br><br>**VMI mean (SD)**<br>No IVH, 92.7 (20)<br>IVH grade 3, 67.5 (14) p=0.04<br>IVH grade 4, 76 (26.8) p=0.04<br><br>**Cerebral palsy**<br>No IVH, 14.3%<br>IVH grade 3, 63.6% p<0.01<br>IVH grade 4, 90.9% p<0.01<br><br>**Visual impairment**<br>No IVH, 7.5%<br>IVH grade 3, 45.5%, p=0.03<br>IVH grade 4, 90.9% p<0.01<br><br>**Acoustic impairment**<br>No IVH, 2.2%<br>IVH grade 3, 0% p= not significant<br>IVH grade 4, 0% p= not significant
17	Koc 2016[24]<br><br>Turkey<br><br>Retrospective cohort	**Population (n=90)**<br>• Gestation <32 weeks<br>• Birthweight <1500g<br>• Born 2001<br><br>**Exposure**<br>• IVH grade 1-2 (n= 7)<br>• IVH grade 3-4 (n= 8)<br><br>**Comparator**<br>• No IVH (n=75)<br><br>**Ascertainment/ definition**<br>• Neonatal unit database and medical records	**Outcomes**<br>• Cognitive<br><br>**Measurement/ assessment**<br>• WISC-R<br><br>**Follow-up**<br>• 5.9-7.9 years<br>• 100% follow-up	**WISC-R score <85**<br>IVH (n=7; 46.7%)<br>No IVH (n= 25; 33.3%)<br><br>**WISC-R score >85**<br>IVH grade (n=8; 13.8%)<br>No IVH (n= 50; 84.2%)<br><br>p=0.381
18	Martinez-Cruz 2008[45]<br><br>Mexico<br><br>Case control	**Population**<br>• Gestation <34 weeks<br>• Birthweight <1500g<br>• Born 1990-2005<br><br>**Exposure (n=103)**<br>• IVH	**Outcomes**<br>• Sensorineural hearing loss<br><br>**Measurement/ assessment**<br>• Brainstem auditory evoked potentials<br>• Transient auditory evoked otoacoustic emissions<br>• Behavioural hearing evaluation	**IVH**<br>Sensorineural hearing loss (n=71; 48.6%)<br>No sensorineural hearing loss (n=32; 11.8%)<br><br>**Multivariate logistic regression of risk factors for sensorineural hearing loss**<br>IVH: aOR 7.1 95% CI (4.34, 11.6) p<0.000

		**Comparator (n=315)**<br>• No IVH<br><br>**Ascertainment/ definition**<br>• Medical records<br>• Ultrasound diagnosis.<br>• Papile classification.	• Free field audiometry<br>• Tympanometry<br>• Pure Tone Audiometry<br><br>**Follow-up**<br>• Mean age 7.8±3.7 years<br>• 100% follow-up (case control)	
19	Neubauer 2008[12]<br><br>Germany<br><br>Prospective cohort	**Population**<br>• Birthweight <1000g<br>• Born 1993-1998<br><br>**Exposure**<br>• IVH grade 1-2 (n=26)<br>• IVH grade 3-4, PVL (n=18)<br><br>**Comparator**<br>• Unmatched<br>• No IVH or PVL (n=91)<br><br>**Ascertainment/ definition**<br>• Ultrasound diagnosis<br>• Papile classification	**Outcomes**<br>• Neurodevelopmental impairment (composite)<br><br>**Measurement/assessment**<br>• Modified Touwen test<br>• K-ABC<br>• Snijders-Oomen Non-Verbal Intelligence Test<br>• Hamburg-Wechsler Intelligence Test for Children<br><br>**Follow-up**<br>• 10 years<br>• 79% follow-up	**Logistic regression for major impairment vs. normal development or minor impairment at school age**<br><br>**Grade 3-4 IVH or PVL**<br>Normal (n=4, 22%)<br>Minor (n=2, 11%)<br>Major (n=12, 67%)<br>Risk of impairment: OR 2.46 95% CI (0.52–11.7)
20	Piris Borregas 2019[13]<br><br>Spain<br><br>Retrospective cohort study	**Population (n=1001)**<br>• Birthweight 500-1250g<br>• Born 1991-2008<br><br>**Exposure**<br>• Severe brain injury (IVH grade 3-4, ventriculomegaly III, PVL or intraparenchymal echodense lesion grade 3 or greater)<br><br>**Comparator**<br>• Unmatched<br><br>**Ascertainment/ definition**<br>• Neonatal database<br>• Ultrasound diagnosis<br>• Papile classification	**Outcomes**<br>• Neurodevelopment (composite)<br>• Cognitive<br>• Motor<br>• Hearing impairment<br>• Visual impairment<br><br>**Assessment/ measurement**<br>• GMFCS<br><br>**Follow-up**<br>• 7 years	**Poor neurodevelopmental outcome**<br>**Severe brain injury**, n=46, 32%<br>**No severe brain injury**, n=208, 24%<br>OR 1.41 95% CI (0.94, 2.10) p=0.09<br>Independent OR 2.02 95% CI (1.22, 3.31) p=0.18<br><br>**Severe brain injury (birthweight 500-1000g)**<br>Independent OR 2.02 95% CI (1.22, 3.31)
21	Pittet 2019[25]<br><br>Switzerland<br><br>Prospective cohort study	**Population**<br>• Gestation <30 weeks<br>• Born 2006<br><br>**Exposure**<br>• IVH grade 3-4 or cPVL (n=22)<br><br>**Comparator**<br>• Unmatched<br>• No IVH grade 3-4 or cPVL (n=213)<br><br>**Ascertainment/ definition**<br>• Swiss neonatal network follow-up group	**Outcomes**<br>• Cognitive<br>• Cerebral palsy<br>• Visual impairment<br>• Hearing impairment<br><br>**Assessment/ measurement**<br>• Kaufman ABC<br>• Neurological exam<br>• GMFCS<br><br>**Follow-up**<br>• 5.5 – 6 years<br>• 81% follow-up	**Cognitive (K-ABC – MPC score < 1SD)**<br>IVH 3-4 or PVL<br>OR 2.9 95% CI (1, 8.2) p=0.04<br>aOR 2.3 95% CI (0.7, 7.7) p=0.15<br><br>**Use of early intervention/ therapy service**<br>IVH 3-4 or cPVL aOR 2.7 95% CI (1.3, 5.7)
22	Sherlock 2005[14]<br><br>Australia<br><br>Prospective cohort	**Population**<br>• Gestation <28 weeks<br>• Birthweight <1000g<br>• Survivors born 1991-1992<br><br>**Exposure**<br>• IVH Grade 1 (n=47)<br>• IVH Grade 2 (n= 25)<br>• IVH Grade 3 (n= 12)<br>• IVH Grade 4 (n= 6)<br><br>**Comparator**<br>• Matched on sex, mother's country of birth, and health insurance status<br>• Extremely low birth weight or very preterm infants without IVH (n=180)<br><br>**Ascertainment/ definition**<br>• Enrolled in Victorian Collaborative Study<br>• Ultrasound diagnosis (at least one scan by a certified sonographer)<br>• Worst grade of IVH on either side used<br>• Papile classification	**Outcomes**<br>• Disability (composite)<br>• Neurosensory disability (composite)<br>• Cognitive<br>• Motor<br>• Cerebral palsy<br>• Speech and language<br>• Visual impairment<br>• Hearing impairment<br><br>**Measurement/assessment**<br>• Medical assessment<br>• Movement ABC<br>• WISC-III<br>• Tower of London<br>• Rey Complex Figure<br>• WRAT<br><br>**Follow-up**<br>• Mean 8.7 years<br>• 92.3% follow-up	**Abnormal movement**<br>No IVH (n=39, 22.5%)<br>Grade 1 IVH (n=11, 25%)<br>Grade 2 IVH (n=6, 30%)<br>Grade 3 IVH (n=3, 27.3%)<br>Grade 4 IVH (n=4, 100%)<br>X^2 linear trend = 5.3; P = 0.021<br><br>**Cerebral palsy**<br>No IVH (n=12, 6.7%)<br>Grade 1 IVH (n=3, 6.4%)<br>Grade 2 IVH (n=6, 24%)<br>Grade 3 IVH (n=2, 16.7%)<br>Grade 4 IVH (n=6, 100%)<br>X^2 linear trend = 31.7; p <0.0001<br><br>**Moderate to severe cerebral palsy**<br>No IVH (n=4, 2.2%)<br>Grade 1 IVH (n=0, 0%)<br>Grade 2 IVH (n=4, 15%)<br>Grade 3 IVH (n=1, 8.3%)<br>Grade 4 IVH (n=5, 83.3%)<br>X^2 linear trend = 40.8; p <0.0001<br><br>**Major neurosensory disability**<br>No IVH (n=28, 15.6%)<br>Grade 1 IVH (n=5, 10.6%)<br>Grade 2 IVH (n=5, 20%)<br>Grade 3 IVH (n=1, 8.3%)<br>Grade 4 IVH (n=6, 100%)<br>X^2 linear trend = 6.9; p = 0.009<br><br>**IQ score mean (SD)**<br>No IVH 0.71 (1.25)<br>Grade 1 IVH 0.76 (1.32)<br>Grade 2 IVH 0.71 (1.12)<br>Grade 3 IVH 1.21 (1.13)<br>Grade 4 IVH 3.28 (0.88)<br>ANOVA F4,265 = 6.7; p<0.0001<br><br>**Verbal comprehension index mean (SD)**<br>No IVH 96.6 (16.2)<br>Grade 1 IVH 96.3 (15.7)<br>Grade 2 IVH 99.6 (12.8)<br>Grade 3 IVH 93.1 (15.4)

				Grade 4 IVH 74.3 (12.7)<br>ANOVA F4,251 = 1.8; p = 0.12<br><br>**Perceptual organisation index mean (SD)**<br>No IVH 98.5 (16.3)<br>Grade 1 IVH 98.2 (15.7)<br>Grade 2 IVH 96.9 (14.8)<br>Grade 3 IVH 91.6 (12.7)<br>Grade 4 IVH 71.7 (11.1)<br>ANOVA F4,249 = 2.5; p = 0.042<br><br>**Freedom from distractibility index mean (SD)**<br>No IVH 92.3 (114.9)<br>Grade 1 IVH 95.5 (15.0)<br>Grade 2 IVH 97.7 (12.8)<br>Grade 3 IVH 94.9 (17.4)<br>Grade 4 IVH 71.0 (3.5)<br>ANOVA F4,250 = 2.8; p = 0.026<br><br>**Processing speed index mean (SD)**<br>No IVH 99.5 (15.8)<br>Grade 1 IVH 99.1 (16.6)<br>Grade 2 IVH 99.3 (13.0)<br>Grade 3 IVH 94.9 (19.3)<br>Grade 4 IVH 71.0 (9.5)<br>ANOVA F4,245 = 2.7; p = 0.033<br><br>**Tower of London (executive function) raw score mean (SD)**<br>No IVH 73.3 (14.4)<br>Grade 1 IVH 71.5 (12.4)<br>Grade 2 IVH 71.1 (20.4)<br>Grade 3 IVH 66.5 (8.3)<br>Grade 4 IVH 54.3 (22.0)<br>ANOVA F4,244 = 1.8; p = 0.13<br><br>**Rey complex figure (executive function) raw score mean (SD)**<br>No IVH 22.5 (7.5)<br>Grade 1 IVH 23.1 (7.4)<br>Grade 2 IVH 24.2 (5.8)<br>Grade 3 IVH 19.3 (8.3)<br>Grade 4 IVH 11.2 (9.8)<br>ANOVA F4,242 = 2.6; p = 0.037<br><br>**Wide range achievements test score mean (SD)**<br>**Reading**<br>No IVH 95.2 (15.7)<br>Grade 1 IVH 102.7 (15.4)<br>Grade 2 IVH 99.0 (14.2)<br>Grade 3 IVH 98.1 (11.9)<br>Grade 4 IVH 70.5g (20.9)<br>ANOVA F4,251 = 5.1; p = 0.001<br><br>**Spelling**<br>No IVH 93.6 (12.4)<br>Grade 1 IVH 97.8 (12.3)<br>Grade 2 IVH 95.9 (10.8)<br>Grade 3 IVH 96.8 (11.9)<br>Grade 4 IVH 73.5 (20.0)<br>ANOVA F4,250 = 4.0; p = 0.003<br><br>**Arithmetic**<br>No IVH 88.3 (14.3)<br>Grade 1 IVH 93.6 (14.9)<br>Grade 2 IVH 92.6 (10.6)<br>Grade 3 IVH 89.1 (10.1)<br>Grade 4 IVH 65.5 (14.5)<br>ANOVA F4,248 = 4.5; p = 0.002<br><br>**Cognitive test scores (compared to normal birthweight controls)**<br>**IQ score <1 SD from the mean (n, %)**<br>No IVH n=64 (35.6%)<br>Grade 1 IVH n=18 (38.3%)<br>Grade 2 IVH n=9 (36%)<br>Grade 3 IVH n=7 (58.3%)<br>Grade 4 IVH n=6(100%)<br>X^2 linear trend=6.8; P=0.009<br><br>**Wide range achievements test score <1 SD from the mean, n (%)**<br>**Low reading**<br>No IVH n=42 (24.4%)<br>Grade 1 IVH n=6 (13.3%)<br>Grade 2 IVH n=5 (20.8%)<br>Grade 3 IVH n=2 (18.2%)<br>Grade 4 IVH n=3 (75%)<br>X^2 linear trend=0.1; p=0.77<br><br>**Low spelling**<br>No IVH n=33 (19.2%)<br>Grade 1 IVH n=6 (13.6%)<br>Grade 2 IVH n=2 (8.3%)<br>Grade 3 IVH n=3 (27.3%)<br>Grade 4 IVH n=3 (75%)<br>X^2 linear trend=0.7; p=0.39<br><br>**Low arithmetic**<br>No IVH n=47 (27.6%)<br>Grade 1 IVH n=9 (20.5%)<br>Grade 2 IVH n=2 (8.3%)<br>Grade 3 IVH n=3 (27.3%)<br>Grade 4 IVH n=4 (100%)<br>X^2 linear trend=0.1; p=0.79

23	Tymofiyeva 2018[33]<br><br>USA<br><br>Prospective cohort	**Population (n=24)**<br>• Gestation < 33 weeks<br><br>**Exposure**<br>• Mild WMI (n=4)<br>• Moderate WMI (n=5)<br>• Severe WMI (n=1)<br><br>• IVH grade 1 (n=5)<br>• IVH grade 2 (n=0)<br>• IVH grade 3 (n=0)<br>• IVH grade 4 (n=0)<br><br>**Comparator**<br>• Unmatched<br>• No WMI (n=14)<br>• No IVH (n=19)<br><br>**Ascertainment/ definition**<br>• MRI imaging reviewed by a blinded paediatric neuroradiologist<br>• Used own classification of white matter injury<br>• Papile classification	**Outcome**<br>• Cognitive<br>• Behaviour<br><br>**Assessment/ measurement**<br>• Test of variables of attention<br>• Conners comprehensive behaviour rating scales<br>• CBCL<br>• Assessment undertaken by a blinded psychologist<br>• Parental questionnaire<br><br>**Follow-up**<br>• 10-14 years<br>• Completeness not specified	**Attention (abnormal)**<br>Mild WMI n=3, 75%<br>Moderate WMI n=0, 0%<br>No WMI n=8, 57% p=0.05
24	Van de Bor 2004[15]<br><br>Netherlands<br><br>Prospective cohort	**Population**<br>• Gestation < 32 weeks<br>• Birthweight < 1500 g<br>• Born 1983<br><br>**Exposure**<br>• IVH grade 1-2 (n=45)<br>• IVH grade 3-4 (n=17)<br><br>**Comparator (n=216)**<br>• Unmatched<br>• No IVH<br><br>**Ascertainment/ definition**<br>• Ultrasound diagnosis<br>• Papile classification	**Outcomes**<br>• Disability (composite)<br>• Cognitive<br>• Neurological status (motor)<br>• Speech and language<br>• Behaviour<br>• Hearing<br>• Vision<br>•<br>**Measurement/assessment**<br>• Questionnaires (completed by parents at 9 years; adolescents at 14 years)<br>• Home visit and neurodevelopmental assessment by paediatrician unaware of medical history<br>• WHO classification of impairment, disability, and handicap<br><br>**Follow-up**<br>• 5, 9 and 14 years<br>91.5% follow-up of survivors at 14 years	**Disability at 5 years**<br>No IVH n=49 (23%)<br>IVH grade 3-4 n=5 (31.3%)<br><br>**Cognitive disability**<br>No IVH n=18 (8.3%)<br>IVH grade 3-4 n=1 (5.9%) p=not significant<br><br>**Motor disability**<br>No IVH n=8 (3.7%)<br>IVH grade 3-4 n=3 (17.6%) p=0.00<br><br>**Speech/language disability**<br>No IVH n=34 (15.7%)<br>IVH grade 3-4 n=1 (5.9%) p= not significant<br><br>**Visual disability**<br>No IVH n=1 (0.5%)<br>IVH grade 3-4 n=0 p= not significant<br><br>**Hearing disability**<br>No IVH n=5 (2.3%)<br>IVH grade 3-4 n=0 p= not significant<br><br><br>**School performance at 5 years**<br>**Special education**<br>No IVH n=17 (8.7%)<br>IVH grade 3-4 n=3 (20%) p=0.02<br><br>**School performance at 9 years**<br>**Slow learner**<br>No IVH n=57 (29.5%)<br>IVH grade 3-4 n=4 (26.7%)<br><br>**Special education**<br>No IVH n=29 (15%)<br>IVH grade 3-4 n=4 (26.7%) p=0.04<br><br>**School performance at 14 years**<br>**Slow learner**<br>No IVH n=93 (44.1)<br>IVH grade 3-4 n=4 (23.5%)<br><br>**Special education**<br>No IVH n=26 (12%)<br>IVH grade 3-4 n=6 (35.3%) p=0.00<br><br>**Need for special education at 14 years**<br>IVH (all grades)<br>OR 2.56 95%CI (1.17-4.86)<br>aOR 2.33 95%CI (1.15, 4.75)<br><br>IVH grade 3-4<br>aOR 3.99 95%CI (1.36, 11.69)
25	Van Den Hout 2000[26]<br><br>Netherlands<br><br>Prospective cohort	**Population**<br>• Mean gestation 28-30 weeks<br>• Born 1989-1991<br><br>**Exposure**<br>• IVH (n=17)<br>• PVL (n=12)<br><br>**Comparator (n=17)**<br>• Preterm<br>• Normal cranial ultrasound<br><br>**Ascertainment/ definition**<br>• Ultrasound diagnosis<br>• Modified Levene and DeVries classification for IVH<br>• DeVries classification for PVL	**Outcomes**<br>• Cognitive<br>• Visual acuity<br><br>**Measurement/ assessment**<br>• L94 visual-perceptual ability test<br>• Grating acuity cards<br>• McCarthy scales of children's abilities<br>• Wechsler preschool and primary scale of intelligence<br>• Snijders-Oomen non-verbal intelligence test<br>• Leiden Diagnostic test<br><br>**Follow-up**<br>• Mean 5.3 years<br>• 88% follow-up	**Total intelligence quotient, mean (SD)**<br>IVH 92.4 (16.3)<br>PVL 79.6 (20.5)<br>No brain injury 102.8 (14.4)<br><br>**IQ <85**<br>IVH n=6, 35.3%<br>PVL n=6, 50%<br>No brain injury n=2, 11.8%<br><br>**Performance age in years, mean (SD)**<br>IVH 5.22 (1.16)<br>PVL 4.37 (1.19)<br>No brain injury 6.22 (0.89)<br><br>**Visual grating acuity in c/deg, mean (SD)**<br>IVH 37.4 (13.5)<br>PVL 33.5 (15.9)

				No brain injury 47.1 (13.5)<br><br>**Visual grating acuity <25c/deg (%)**<br>IVH (11.8)<br>PVL (33.3)<br>No brain injury (0)<br><br>**Impairment on each of the eight L94 tasks**<br>**Visual matching % (n)**<br>IVH 0 (17)<br>PVL 0 (12)<br>No brain injury 5.9 (17)<br><br>**Unconventional Object Views % (n)**<br>IVH 29.4 (17)<br>PVL 41.7 (12)<br>No brain injury 17.6 (17)<br><br>**De Vos task % (n)**<br>IVH 29.4 (17)<br>PVL 41.7 (12)<br>No brain injury 11.8 (17)<br><br>**Line Drawings Occluded by Noise% (n)**<br>IVH 6.3 (16)<br>PVL 36.4 (11)<br>No brain injury 0 (17)<br><br>**Line Drawings Occluded by Noise% (n)**<br>IVH 13.3 (15)<br>PVL 25.0 (8)<br>No brain injury 5.9 (17)<br><br>**Developmental test of visual motor integration % (n)**<br>IVH 0 (16)<br>PVL 0 (7)<br>No brain injury 0 (17)<br><br>**Matching block designs % (n)**<br>IVH 5.9 (17)<br>PVL 20.0 (10)<br>No brain injury 17.6 (17)<br><br>**Constructing block designs% (n)**<br>IVH 30.8 (13)<br>PVL 80.0 (5)<br>No brain injury 31.3 (16)<br><br>**Mean percentage of L94 tasks on which child is impaired (mean, SD; %)**<br>IVH 14.71 (17.81)<br>PVL 32.04 (24.64)<br>No brain injury 11.13 (9.79)
26 *	Vollmer 2003[16]<br><br>UK<br><br>Prospective cohort	**Population**<br>• Gestation <33 weeks<br>• Born 1983-1988<br><br>**Exposure**<br>• IVH (n=159)<br>• Ventricular dilatation (n=32)<br>• IVH, PV flare, ventricular dilatation (n=164)<br>• Hydrocephalus (n=36)<br>• Haemorrhagic parenchymal infarction (HPI) (n=61)<br>• cPVL n=26<br><br>**Comparator (n=348)**<br>• Unmatched<br>• Normal scan<br><br>**Ascertainment/ definition**<br>• Ultrasound imaging reviewed by two experienced observers<br>• In-house classification used	**Outcomes**<br>• Neurodevelopmental impairment (composite)<br>• Visual impairment<br>• Hearing impairment<br><br>**Measurement/ assessment**<br>• Structured neurologic examination<br>• Pure-tone audiogram<br>• Vision test (Snellen chart)<br>• Henderson-Stott TOMI<br>• Beery test of VMI<br>• WISC-R for children born 1983-1986<br>• WISC-III for children born 1987-1988<br><br>**Follow-up**<br>• 8 years<br>• 91.7% follow-up	**Neurodevelopmental status**<br>**Group A (<28 weeks)**<br>**All impairments (n,%)**<br>GMH/IVH (5, 18%)<br>Ventricular dilatation (4, 50%)<br>GMH/IVH, flare, ventricular dilatation (19, 51%)<br>Hydrocephalus (7, 78%)<br>HPI (15, 100%)<br>cPVL (4, 100%)<br>No brain injury (12, 32%)<br><br>**Disabling impairments (n, %)**<br>GMH/IVH (1, 4%)<br>Ventricular dilatation (0, 0%)<br>GMH/IVH, flare, ventricular dilatation (9, 24%)<br>Hydrocephalus (7, 78%)<br>HPI (14, 93%)<br>cPVL (3, 75%)<br>No brain injury (3, 8%)<br><br>**Group B (28-32 weeks)**<br>**All impairments (n, %)**<br>GMH/IVH (16, 29%)<br>Ventricular dilatation (5, 31%)<br>GMH/IVH, flare, ventricular dilatation (30, 43%)<br>Hydrocephalus (7, 54%)<br>HPI (5, 83%)<br>cPVL (9, 75%)<br>No brain injury (67, 29%)<br><br>**Disabling impairments (n, %)**<br>GMH/IVH (5, 5%)<br>Ventricular dilatation (1, 6%)<br>GMH/IVH, flare, ventricular dilatation (16, 23%)<br>Hydrocephalus (6, 46%)<br>HPI (3, 50%)<br>cPVL (6, 50%)<br>No brain injury (14, 6%)
27 *	Vollmer 2006a[21]<br><br>UK<br><br>Prospective cohort	**Population**<br>• Gestation <33 weeks<br>• Born 1985-1991<br><br>**Exposure**<br>• Bilateral brain lesions (n=201)<br>• Right-sided brain lesion (n=41)	**Outcomes**<br>• Motor<br>• Cognitive<br>• Cerebral palsy<br>• Visual	**TOMI error score, mean (SD)**<br>Normal scan 2.78 (2.1)<br><br>All left-sided lesions 4.3 (3.5)<br>Left-sided non-parenchymal lesions 4.5 (3.8)<br>Left-sided parenchymal lesions 3.7 (2.1)

		• Left-sided brain lesion (n=57)<br><br>Brain lesion types<br>Non-parenchymal:<br>• Uncomplicated IVH<br>Parenchymal:<br>• Haemorrhagic parenchymal infarction (HPI)<br>• cPVL<br>• PV flare<br><br>**Comparator (n=369)**<br>• Unmatched<br>• Normal ultrasound<br><br>**Ascertainment/ definition**<br>• Ultrasound imaging reviewed by two experienced observers<br>• Modified Stewart classification	**Measurement/ assessment**<br>• Neurological examination (modified Amiel-Tison assessment)<br>• TOMI<br>• WISC-R<br>• Test of VMI<br><br>**Follow-up**<br>• 8 years<br>• 80% follow-up	All right-sided lesions 3.5 (2.9)<br>Right-sided non-parenchymal lesions 2.7 (1.8)<br>Right-sided parenchymal lesions 4.9 (3.8)<br><br>All bilateral lesions 4.5 (4.3)<br>Bilateral non-parenchymal lesions 4.1 (3.7)<br>Bilateral parenchymal lesions 4.9 (4.7)<br><br>ANOVA for parenchymal lesions only p <0.0001<br>ANOVA including parenchymal and non-parenchymal lesions p <0.0001<br>ANOVA excluding parenchymal lesions, p <0.0001<br><br>**VMI centile, mean (SD)**<br>Normal scan 59.2 (30.0)<br><br>All left-sided lesions 40.3 (30.1)<br>Left-sided non-parenchymal lesions 46.8 (31.0)<br>Left-sided parenchymal lesions 21 (22)<br><br>All right-sided lesions 60.2 (31.9)<br>Right-sided non-parenchymal lesions 64.2 (30.2)<br>Right-sided parenchymal lesions 54 (35)<br><br>All bilateral lesions 46.0 (33.5)<br>Bilateral non-parenchymal lesions 55.1 (32.1)<br>Bilateral parenchymal lesions 38 (32)<br><br>ANOVA for parenchymal lesions only p <0.0001<br>ANOVA including parenchymal and non-parenchymal lesions p <0.0001<br>ANOVA excluding parenchymal lesions reported as both p <0.0001 and p=0.98 Ω(potential error in the manuscript table)<br><br>**Cerebral palsy, n (%)**<br>Normal scan 2 (0.7%)<br><br>All left-sided lesions 4 (9%)<br>Left-sided non-parenchymal lesions 2 (6%)<br>Left-sided parenchymal lesions 2 (16%)<br><br>All right-sided lesions 2 (6%)<br>Right-sided non-parenchymal lesions 1 (4%)<br>Right-sided parenchymal lesions 1 (8%)<br><br>All bilateral lesions 37 (21%)<br>Bilateral non-parenchymal lesions 8 (10%)<br>Bilateral parenchymal lesions 29 (31%)<br><br>Chi-square for parenchymal and non-parenchymal lesions, p <0.0001<br>Chi-square excluding parenchymal lesions, p <0.0001<br>Chi-square for parenchymal lesions only, p <0.0001<br>ANOVA parenchymal lesions only, p <0.0001<br><br>**Full scale IQ, mean (SD)**<br><br>Normal scan 101 (16)<br><br>All left-sided lesions 93 (17)<br>Left-sided non-parenchymal lesions 98 (15)<br>Left-sided parenchymal lesions 80 (15)<br><br>All right-sided lesions 102 (17)<br>Right-sided non-parenchymal lesions 104 (15)<br>Right-sided parenchymal lesions 100 (19)<br><br>All bilateral lesions 91 (21)<br>Bilateral non-parenchymal lesions 96(19)<br>Bilateral parenchymal lesions 86 (22)<br><br>ANOVA for parenchymal lesions only, p <0.0001.<br>ANOVA including parenchymal and non-parenchymal lesions, p <0.0001.<br>ANOVA excluding parenchymal lesions, p =0.137.<br><br>**Verbal IQ, mean (SD)**<br>Normal scan 103 (19)<br><br>All left-sided lesions 98 (20)<br>Left-sided non-parenchymal lesions 102 (20)<br>Left-sided parenchymal lesions 85 (18)<br><br>All right-sided lesions 107 (18)<br>Right-sided non-parenchymal lesions 108 (16)<br>Right-sided parenchymal lesions 107 (22)<br><br>All bilateral lesions 96 (23)<br>Bilateral non-parenchymal lesions 100 (20)<br>Bilateral parenchymal lesions 91 (25)<br><br>ANOVA for parenchymal lesions only, p <0.0001<br>ANOVA including parenchymal and non-parenchymal lesions, p <0.0001<br>ANOVA excluding parenchymal lesions, p =0.38<br><br>**Performance IQ, mean (SD)**<br>Normal scan 96 (15)<br><br>All left-sided lesions 86 (16)<br>Left-sided non-parenchymal lesions 90 (15)<br>Left-sided parenchymal lesions 76 (15)

				All right-sided lesions 95 (16)<br>Right-sided non-parenchymal lesions 98 (13)<br>Right-sided parenchymal lesions 92 (19)<br><br>All bilateral lesions 85 (22)<br>Bilateral non-parenchymal lesions 91 (20)<br>Bilateral parenchymal lesions 80 (21)<br><br>ANOVA for parenchymal lesions only, p <0.0001<br>ANOVA including parenchymal and non-parenchymal lesions, p <0.0001<br>ANOVA excluding parenchymal lesions, p =0.59
28<br>*	Vollmer 2006b[27]<br><br>UK<br><br>Prospective cohort	**Population**<br>• Gestation <33 weeks<br>• Born 1979-1991<br><br>**Exposure (n=66)**<br>• Ventricular dilatation and IVH<br><br>**Comparator (n=616)**<br>• Unmatched<br>• Normal cranial ultrasound<br><br>**Ascertainment/ definition**<br>• Ultrasound imaging reviewed by two experienced observers<br>• In-house classification used	**Outcomes**<br>• Neurological impairment with or without disability (composite)<br>• Cognitive<br>• Motor<br>• Vision<br><br>**Measurement/ assessment**<br>• Structured neurological exam<br>• TOMI<br>• Test of VMI<br>• WISC<br><br>**Follow-up**<br>• 8 years<br>• 81% follow-up	**Disabling motor impairment, n (%)**<br>Ventricular dilatation and IVH n=10 (16%)<br>Normal ultrasound n=10 (2%)<br><br>**Cognitive**<br>**Full scale IQ, mean (SD)**<br>Ventricular dilatation and IVH 96 (23)<br>Normal ultrasound 101 (17)<br><br>**Verbal IQ, mean (SD)**<br>Ventricular dilatation and IVH 101 (22)<br>Normal ultrasound 104 (19)<br><br>**Performance IQ mean (SD)**<br>Ventricular dilatation and IVH 97 (15)<br>Normal ultrasound 91 (21)<br><br>**Motor and vision**<br>**VMI centile, mean (SD)**<br>Ventricular dilatation and IVH 37 (33)<br>Normal ultrasound 52 (31)<br><br>**TOMI, mean (SD)**<br>Ventricular dilatation and IVH 5.98 (4.2)<br>Normal ultrasound 3.26 (2.5)
29	Whitaker 2011[30]<br><br>USA<br><br>Prospective cohort	**Population**<br>• Birthweight <2000g<br>• 'Non-disabled' survivors<br>• Born 1984-1987<br><br>**Exposure**<br>• IVH (n=69)<br>• Parenchymal lesions and/or ventricular enlargement (n=21)<br><br>**Comparison (n=368)**<br>• Unmatched<br>• Normal cranial ultrasound<br><br>**Ascertainment/ definition**<br>• Ultrasound imaging reviewed by three blinded radiologists independently, disagreements resolved through consensus and inter-observer reliability checked.<br>• Paneth classification	**Outcomes**<br>• Mental health conditions<br><br>**Measurement/ assessment**<br>• Parent report version of the Diagnostic Interview Schedule for Children–IV<br>• WASI<br><br>**Follow-up**<br>• 16 years<br>• 72.9% follow-up	**Logistic regression assessing odds of current and lifetime mental health conditions after brain injury**<br><br>**Current ADHD- inattentive type**<br>**IVH**<br>OR 0.97 95% CI (0.21-4.47)<br>aOR 1.01 95% CI (0.19-5.44)<br><br>**Parenchymal lesions and/or ventricular enlargement**<br>OR 7.64[b] 95% CI (2.20-24.48)<br>aOR 6.83[c] 95% CI (1.26-36.91)<br><br>**Lifetime ADHD – inattentive type**<br>**IVH**<br>OR 0.83 95% CI (0.34-2.04)<br>aOR 0.64 95% CI (0.24-1.74)<br><br>**Parenchymal lesions and/or ventricular enlargement**<br>OR 2.71 95% CI (0.94-7.82)<br>aOR 1.13 95% CI (0.31-4.10)<br><br>**Current major depression**<br>**IVH**<br>OR 2.66 95% CI (1.04-6.78)<br>aOR 2.23 95% CI (0.80-6.24)<br><br>**Lifetime major depression**<br>**IVH**<br>OR 2.76 95% CI (1.19-6.38)<br>aOR 2.59 95% CI (1.02-6.58)<br><br>**Current tic disorders**<br>**IVH**<br>OR 1.63 95% CI (0.44-6.07)<br>aOR 1.89 95% CI (0.42-8.57)<br><br>**Parenchymal lesions and/or ventricular enlargement**<br>OR 8.42 95% CI (2.40-29.62)<br>aOR 9.77 95% CI (1.69-56.47)<br><br>**Lifetime tic disorders**<br>**IVH**<br>OR 0.95 95% CI (0.27-3.34)<br>aOR 0.85 95% CI (0.21-3.51)<br><br>**Parenchymal lesions and/or ventricular enlargement**<br>OR 5.07 95% CI (1.53-16.82)<br>aOR 5.02 95% CI (1.05-23.92)<br><br>**Current obsessive-compulsive disorder**<br>**IVH**<br>OR 9.52 95% CI (3.02-30.06)<br>aOR 11.85 95% CI (3.22-43.62)<br><br>**Parenchymal lesions and/or ventricular enlargement**<br>OR 7.64 95% CI (1.39-41.98)<br>aOR 15.32 95% CI (1.82-128.74)<br><br>**Lifetime obsessive compulsive disorder**<br>**IVH**<br>OR 9.52 95% CI (3.05-30.06)<br>aOR 11.85 95% CI (3.22-43.62)

				**Parenchymal lesions and/or ventricular enlargement**<br>OR 7.64 95% CI (1.39-41.98)<br>aOR 15.32 95% CI (1.82-128.74)<br><br>**Current diagnoses additionally controlled for full score IQ and motor function**<br><br>**ADHD inattentive type**<br>**IVH**<br>OR 0.86 95% CI (0.18-3.99)<br>aOR 0.99 95% CI (0.21-4.62)<br><br>**Parenchymal lesions and/or ventricular enlargement**<br>OR 5.04 95% CI (1.36-18.65)<br>aOR 5.43 95% CI (1.32-22.40)<br><br>**Major depression**<br>**IVH**<br>OR 0.43 95% CI (0.16-1.11)<br>aOR 0.40 95% CI (0.15-1.05)<br><br>**Tic disorders**<br>**IVH**<br>OR 1.54 95% CI (0.41-5.78)<br>aOR 1.45 95% CI (0.38-5.48)<br><br>**Parenchymal lesions and/or ventricular enlargement**<br>OR 7.01 95% CI (1.88-28.14)<br>aOR 4.38 95% CI (1.05-18.23)<br><br>**Obsessive compulsive disorder**<br>**IVH**<br>OR 8.68 95% CI (2.72-27.69)<br>aOR 10.91 95% CI (3.13-37.99)<br><br>**Parenchymal lesions and/or ventricular enlargement**<br>OR 4.78 95% CI (0.83-28.10)<br>aOR 3.58 95% CI (0.50-25.94)
Perinatal stroke				
30	Ballantyne * 2007 [41]<br>USA<br><br>Prospective cohort	**Population**<br>• Mean gestation 38.5 weeks<br>• Born 1991-2001<br><br>**Exposure (n=28)**<br>• Left lesions (n=17)<br>• Right lesions (n=11)<br><br>**Comparator (n=57)**<br>• Unmatched<br>• Healthy controls with normal medical and developmental histories<br>• Recruited from the community<br><br>**Ascertainment/ definition**<br>• Single unilateral lesions the result of perinatal strokes occurring between 28 weeks' gestation and 28 days after birth; infarct or haemorrhage<br>• Identified through medical history and neuroimaging<br>• Severity rated on a 5-point scale adapted from the Vargha-Khadem classification	**Outcomes**<br>• Speech and language<br><br>**Assessment/ measurement**<br>• CELF-R<br>• Wechsler Intelligence Scales (WPPSI-R, WISC-R, or WISC-III)<br>• PPVT–Revised<br>• Expressive One-Word Picture Vocabulary Test–Revised or Upper-Extension<br>• Total Language Standard Scores<br><br>**Follow-up**<br>• 6-9 years<br>• 100% follow-up	**Speech and language**<br>**CELF-R Receptive, mean (SD)**<br>All strokes: 82.54 (17.12) p<.0001<br>Left stroke: 83.18 (16.66) p<.0001<br>Right stroke: 81.55 (18.59) p=0.001<br>Control: 106.37 (12.51)<br><br>**CELF-R Expressive mean (SD)**<br>All strokes: 73.75 (16.79) p<.0001<br>Left stroke: 73.06 (14.88) p<.0001<br>Right stroke: 74.82 (20.11) p=0.001<br>Control: 101.02 (13.63)<br><br>**CELF-R Total mean (SD)**<br>All strokes: 76.93 (17.31) p<.0001<br>Left stroke: 76.94 (15.39) p<.0001<br>Right stroke: 76.91 (20.74) p=0.001<br>Control: 104.00 (12.58)<br>.
31	Ballantyne 2008[34] *<br><br>USA<br><br>Prospective cohort	**Population**<br>• 32- 40 weeks' gestation<br>• Birth years not reported<br><br>**Exposure (n=29)**<br>• Left hemisphere (n=20)<br>• Right hemisphere (n=9)<br><br>**Control (n=38)**<br>• Healthy controls (normal neurodevelopment)<br>• Recruited through a university and community adverts<br><br>**Ascertainment/ definition**<br>• Unilateral ischaemic perinatal stroke confirmed through clinical history and neuroimaging<br>• Lesion location and severity reviewed by blinded neuroradiologist<br>• Severity rated on a 5-point scale adapted from the Vargha-Khadem classification	**Outcomes**<br>• Cognitive (academic skills)<br>• Speech and language<br>• Motor<br>• Cerebral palsy<br>• Vision<br>• Epilepsy<br><br>**Measurement/ assessment**<br>• WISC- Revised<br>• WRAT- Revised<br>• CELF- Revised<br>• PPVT-Revised<br>• WPPSI/WPPSI- Revised<br>• WISC-III<br><br>**Follow-up**<br>• 7-12 years<br>• 100% follow up	**Hemiparesis**<br>Stroke n=18,62%<br><br>**Visual field deficit**<br>Stroke n=7, 26%<br><br>**Seizures**<br>Stroke n=11, 38%<br><br>**Cognitive, mean (SD)**<br>**Verbal IQ (WISC-R)**<br>**Time point 1 (mean age 7-8 years)**<br>Stroke 96.6 (20.5)<br>Control 126.1 (16)<br><br>**Time point 2 (mean age 10 – 12 years)**<br>Stroke 98.7 (20)<br>Control 123.6 (13.1)<br>Between group affect (stroke vs. control) p<0.0001<br>Time effect not significant<br><br>**Performance IQ (WISC-R)**<br>**Time point 1 (mean age 7-8 years)**<br>Stroke 92.8 (19.9)<br>Control 115.2 (13.8)<br><br>**Time point 2 (mean age 10 – 12 years)**<br>Stroke 93.5 (20)<br>Control 116 (10.5)<br>Between group affect (stroke vs. control) p=0.002<br>Time effect not significant<br><br>**Full scale IQ (WISC-R)**

				**Time point 1 (mean age 7-8 years)**<br>Stroke 94.7 (20.4)<br>Control 123 (15)<br><br>**Time point 2 (mean age 10 – 12 years)**<br>Stroke 96.1 (19.1)<br>Control 122.3 (10.2)<br><br>Between group affect (stroke vs. control) p<0.0001<br>Time effect not significant<br><br>**Reading (WRAT -R)**<br>**Time point 1 (mean age 7-8 years)**<br>Stroke 85 (16.1)<br>Control 113 (13.3)<br><br>**Time point 2 (mean age 10 – 12 years)**<br>Stroke 89.4 (13.3)<br>Control 108.9 (13.8)<br><br>Between group affect (stroke vs. control) p<0.0001<br>Time effect not significant<br>Time group interaction p=0.045<br><br>**Spelling (WRAT -R)**<br>**Time point 1 (mean age 7-8 years)**<br>Stroke 82.5 (18.2)<br>Control 106.2 (15.9)<br><br>**Time point 2 (mean age 10 – 12 years)**<br>Stroke 87 (16.8)<br>Control 104.6 (13.1)<br><br>Between group affect (stroke vs. control) p=0.001<br>Time effect not significant<br><br>**Arithmetic (WRAT -R)**<br>**Time point 1 (mean age 7-8 years)**<br>Stroke 91.5 (10.2)<br>Control 111.9 (11.2)<br><br>**Time point 2 (mean age 10 – 12 years)**<br>Stroke 94.2 (18.7)<br>Control 113.1 (16.2)<br><br>Between group affect (stroke vs. control) p<0.0001<br>Time effect not significant<br><br>**Speech and language**<br>**Receptive language score**<br>**Time point 1 (mean age 7-8 years)**<br>Stroke 84.2 (10.9)<br>Control 109.1 (12.2)<br><br>**Time point 2 (mean age 10 – 12 years)**<br>Stroke 82.3 (20.1)<br>Control 111.4 (13.7)<br><br>Between group affect (stroke vs. control) p<0.0001<br>Time effect not significant<br><br>**Expressive language score**<br>**Time point 1 (mean age 7-8 years)**<br>Stroke 72.5 (12)<br>Control 101 (17.5)<br><br>**Time point 2 (mean age 10 – 12 years)**<br>Stroke 78.4 (16)<br>Control 105.8 (11.9)<br><br>Between group affect (stroke vs. control) p<0.0001<br>Time effect p=0.017<br><br>**Total language score**<br>**Time point 1 (mean age 7-8 years)**<br>Stroke 76.9 (11.1)<br>Control 105.6 (14.2)<br><br>**Time point 2 (mean age 10 – 12 years)**<br>Stroke 79.1 (18.3)<br>Control 109.8 (14)<br><br>Between group affect (stroke vs. control) p<0.0001<br>Time effect not significant<br><br>**Vocabulary score**<br>**Time point 1 (mean age 7-8 years)**<br>Stroke 97.5 (19.7)<br>Control 117.1 (17)<br><br>**Time point 2 (mean age 10 – 12 years)**<br>Stroke 99.9 (20)<br>Control 118.9 (13.9)<br><br>Between group affect (stroke vs. control) p=0.002<br>Time effect not significant
32	Gold 2014[35]<br><br>USA	**Population**<br>• Gestation not provided<br>• Birth years not provided	**Outcomes**<br>• Cognitive (IQ and memory)<br>• Motor<br>• Cerebral palsy	**Cognitive**<br>**Memory**<br>**Stories immediate recall**<br>Controls, mean (SE)13.5 (0.7)

	Prospective cohort	**Exposure (n=27)**<br>• Right-sided stroke (n=12)<br>• Left-sided stroke (n=15)<br><br>**Comparator (n=19)**<br>• Matched for age at follow up, sex, socioeconomic group and maternal education<br>• Healthy controls<br>• Recruited through local advertising<br><br>**Ascertainment/ definition**<br>• Single, unilateral brain lesion in an arterial vascular distribution, either identified in the neonatal period with neuroimaging, or identified later in infancy after presentation with a hemiparesis and imaging documentation of an old unilateral infarct (presumed perinatal stroke)<br>• Recruited from paediatric neurology clinics<br>• Severity graded 1-5 using Trauner/ Vargha-Khaldem classification	**Measurement/ assessment**<br>• WISC-III<br>• Dots and Stories subtests of the Children's Memory Scales<br><br>**Follow-up**<br>• 6-16 years<br>• 100% follow-up	Stroke, mean (SE) 8.4 (0.8) p<0.001<br><br>Stroke and seizures, mean (SE)7 (0.8)<br>Stroke and no seizures, mean (SE) 10.1 (1.4) p=0.06<br><br>Right lesion, mean (SE) 7.8 (1.1)<br>Left lesion, mean (SE) 8.9 (1.2) p=0.51<br><br>**Delayed recall**<br>Controls, mean (SE) 13.9 (0.8)<br>Stroke, mean (SE) 7.9 (0.8) p<0.001<br><br>Stroke and seizures, mean (SE) 6.2 (0.9)<br>Stroke and no seizures, mean (SE) 10 (1.2) p=0.02<br><br>Right lesion, mean (SE) 7.3 (1.1)<br>Left lesion, mean (SE) 8.3 (1.2) p=0.56<br><br>**Delayed recognition**<br>Controls, mean (SE) 11.5 (0.5)<br>Stroke, mean (SE) 8 (0.8) p=0.001<br><br>Stroke and seizures, mean (SE) 7.1 (1.1)<br>Stroke and no seizures, mean (SE) 9.2 (0.9) p=0.17<br><br>Right lesion, mean (SE) 8.3 (1.4)<br>Left lesion, mean (SE) 7.9 (0.9) p=0.8<br><br>**Dots learning**<br>Controls, mean (SE) 10.9 (0.5)<br>Stroke, mean (SE) 8.9 (0.8) p=0.05<br><br>Stroke and seizures, mean (SE) 7.6 (1.1)<br>Stroke and no seizures, mean (SE) 10.6 (0.8) p=0.05<br><br>Right lesion, mean (SE) 9.3 (1.4)<br>Left lesion, mean (SE) 8.7 (0.9) p=0.71<br><br>**Total**<br>Controls, mean (SE) 11.8 (0.5)<br>Stroke, mean (SE) 9 (0.7) p=0.003<br><br>Stroke and seizures, mean (SE) 7.8 (0.9)<br>Stroke and no seizures, mean (SE) 10.6 (0.9) p=0.04<br><br>Right lesion, mean (SE) 9.2 (0.7)<br>Left lesion, mean (SE) 10.2 (0.7) p=0.62<br><br>**Delayed recall**<br>Controls, mean (SE) 12.6 (0.4)<br>Stroke, mean (SE) 10 (0.5) p<0.001<br><br>Stroke and seizures, mean (SE) 8.8 (0.5)<br>Stroke and no seizures, mean (SE) 11.4 (0.8) p=0.009<br><br>Right lesion, mean (SE) 9.7 (0.7)<br>Left lesion, mean (SE) 10.2 (0.7) p=0.62<br><br>**WISC- III IQ, mean (SD)**<br>Right stroke, 85.0 (6)<br>Left stroke, 91 (6) p=0.49<br><br>**IQ scores**<br>Controls 117 (2.7)<br>All stroke patients 88 (4.0) p<0.001<br>No seizures 100 (6.4)<br>Seizures 78 (3.7)<br><br>**<u>Motor (hemiparesis)</u>**<br>Stroke patients n=16; 59%<br>Control n=0; p=0.05
33	Kolk 2011[36]<br><br>Estonia<br><br>Retrospective cohort	**Population**<br>• Gestation not provided<br>• Born 1995-2006<br><br>**Exposed (n=21)**<br>• Neonatal stroke<br><br>**Control (n=31)**<br>• Matched on age and sex<br>• Healthy children<br>• Recruited locally<br><br>**Ascertainment/ definition**<br>• Estonian stroke registry<br>• Arterial ischaemic stroke or haemorrhagic	**Outcomes**<br>• Cognitive<br>• Neuropsychological<br>• Motor<br>• Cerebral palsy<br>• Speech and language<br>• Epilepsy<br><br>**Measurement/ assessment**<br>• NEPSY<br>• Kaufman ABC<br>• Paediatric Stroke Outcome Measure<br><br>**Follow-up**<br>• 4-10 years<br>• 100% follow-up	**<u>Neuromotor impairment (Paediatric Stroke Outcome Measure)</u>**<br>**Neonatal stroke**<br>Severe n=4, 19%<br>Moderate n=9, 43%<br>Good n=6, 28.6%<br>Normal n=2, 9.5%<br><br>**<u>Cognitive/ neuropsychological</u>**<br><br>**<u>Attention and executive function, mean, SD, 95% CI</u>**<br>**Tower**<br>Control 0.22, 0.64 (-0.05, 0.48)<br>Neonatal stroke -0.34, 1.34 (-1.03, 0.35) p=0.142<br><br>**Auditory attention**<br>Control 0.27, 0.72 (-0.03, 0.57)<br>Neonatal stroke -0.38, 1.10 (-1.04, 0.28) p=0.009<br><br>**Visual attention: time**<br>Control 0.37, 0.81, (0.07, 0.67)<br>Neonatal stroke -0.40, 0.93 (-0.82, 0.03) p=0.004<br><br>**Visual attention: correct**<br>Control 0.48, 0.50 (0.30, 0.67)<br>Neonatal stroke -0.54, 0.97 (0.98, 0.1) p<0.0001<br><br>**Statue**

				Control 0.26, 0.77 (-0.03, 0.54)<br>Neonatal stroke -0.23, 1.09, (-0.73, 0.28) p=0.086<br><br>**Design fluency**<br>Control 0.18, 1.04 (-0.25, 0.61)<br>Neonatal stroke -0.36, 0.70 (-0.78, 0.06) p=0.06<br><br>**Knock and tap**<br>Control 0.31, 0.50 (0.10, 0.51)<br>Neonatal stroke -0.44, 1.52, (-1.32, 0.43) p==0.03<br><br>**Language, mean, SD, 95% CI**<br>**Phonological processing**<br>Control 0.24, 0.80 (-0.05, 0.54)<br>Neonatal stroke -0.38, 0.99 (-0.83, 0.08) p=0.001<br><br>**Comprehension of instructions**<br>Control 0.43, 0.70 (0.18, 0.69)<br>Neonatal stroke -0.59 1.06 (-1.07, 0.11) p<0.0001<br><br>**Speeded naming: time**<br>Control 0.24, 0.70 (-0.05, 0.52)<br>Neonatal stroke -0.14, 1.03 (-0.73, 0.46) p=0.188<br><br>**Speeded naming: correct**<br>Control 0.42, 0.41 (0.25, 0.59)<br>Neonatal stroke -0.45, 1.41 (-1.26, 0.37) p=0.008<br><br>**Repetition of nonsense words**<br>Control 0.30, 0.53 (0.08, 0.52)<br>Neonatal stroke -0.40, 1.23 (-1.03, 0.24) p=0.026<br><br>**Verbal fluency: semantic**<br>Control 0.43, 0.81 (0.13, 0.73)<br>Neonatal stroke -0.60, 0.95 (-1.04, 0.15) p<0.0001<br><br>**Verbal fluency: phonemic**<br>Control 0.40, 0.93 (-0.12, 0.92)<br>Neonatal stroke -0.67, 0.90 (-1.42, 0.08) p=0.008<br><br>**Oromotor sequences**<br>Control 0.31, 0.64 (0.07, 0.54)<br>Neonatal stroke -0.52, 1.25 (-1.15, 0.10)<br><br>**Sentence comprehension**<br>Control 0.19, 0.78 (-0.09, 0.48)<br>Neonatal stroke -0.35, 1.09 (-0.91, 0.21) p=0.027<br><br>**Sensorimotor functions, mean, SD, 95% CI**<br>**Finger tapping**<br>Control 0.49, 0.33 (0.35, 0.62)<br>Neonatal stroke -0.53, 1.27 (-1.16, 0.10) p=0.0007<br><br>**Imitating hand positions**<br>Control 0.57, 0.68 (0.32-0.82)<br>Neonatal stroke -0.72, 0.92 (-1.14, 0.30) p<0.0001<br><br>**Visuomotor precision: time**<br>Control 0.13, 0.83 (-0.17, 0.43)<br>Neonatal stroke -0.24, 0.97 (-0.69, 0.20) p=0.145<br><br>**Visuomotor precision: mistakes**<br>Control 0.45, 0.50 (0.27, 0.64)<br>Neonatal stroke -0.42, 1.05 (-0.90, 0.05) p=0.0002<br><br>**Manual motor sequences**<br>Control 0.50, 0.62 (0.27, 0.73)<br>Neonatal stroke -0.92, 0.95 (-1.43, 0.41) p<0.0001<br><br>**Finger discrimination**<br>Control 0.53, 0.57 (0.29, 0.77)<br>Neonatal stroke -0.77, 1.03 (-1.30, 0.24) p<0.0001<br><br>**Visuospatial functions, mean, SD, 95% CI**<br>**Design copying**<br>Control 0.36, 0.80 (0.06, 0.65)<br>Neonatal stroke -0.54, 0.97 (-1.0, 0.09) p<0.0001<br><br>**Arrows**<br>Control 0.37, 0.79 (0.05, 0.70)<br>Neonatal stroke -0.61, 1.07 (-1.16, 0.06) p=0.0004<br><br>**Block construction**<br>Control 0.29, 0.81 (-0.01, 0.58)<br>Neonatal stroke -0.45, 1.04 (-0.92, 0.03) p=0.0003<br><br>**Route finding**<br>Control 0.25, 1.05 (-0.33, 0.83)<br>Neonatal stroke -0.66, 0.80 (-1.23, 0.09) p=0.033<br><br>**Picture perception**<br>Control 0.13, 1.00 (-0.49, 0.24)<br>Neonatal stroke -0.09, 1.03 (-0.56, 0.37) p=0.341<br><br>**Memory and learning, mean, SD, 95% CI**<br>**Memory for faces**<br>Control 0.42, 0.74 (0.11, 0.73)<br>Neonatal stroke -0.41, 1.15 (-0.96, 0.15) p=0.016

				**Memory for names**<br>Control 0.15, 0.92 (-0.23. 0.53)<br>Neonatal stroke -0.31, 1.09 (-0.87, 0.25) p=0.295<br><br>**Narrative memory**<br>Control 0.26, 0.80 (-0.03, 0.55)<br>Neonatal stroke -0.22, 1.16 (-0.78, 0.34) p=0.077<br><br>**Sentence repetition**<br>Control 0.49, 0.61 (0.26, 0.71)<br>Neonatal stroke -0.64, 0.96 (-1.09, 0.19) p<0.0001<br><br>**List learning**<br>Control 0.30, 0.82 (-0.16, 0.76)<br>Neonatal stroke -0.38, 1.22 (-1.32, 0.56) p=0.151<br><br>**Picture recognition**<br>Control 0.39, 0.72 (0.10, 0.69)<br>Neonatal stroke -0.36, 1.24 (-0.98, 0.25) p=0.027<br><br>**Motor (hemiparesis)**<br>Neonatal stroke and any hemiparesis n=19, 90%<br>Mild functional impairment n=6, 29%<br>Significant functional impairment n= 8, 38%<br>Very severe functional impairment n= 4, 19%<br><br>**Epilepsy**<br>Stroke n=9, 33.3%
34	Martin 2019[40] *<br><br>USA<br><br>Prospective cohort	**Population**<br>• Gestation not provided<br>• Birth years not provided<br><br>**Exposure (n=21)**<br>• Left hemisphere (n=13)<br>• Right hemisphere (n=8)<br><br>**Control (n=21)**<br>• Matched on age, sex and socioeconomic status<br>• Healthy controls<br>• Recruited from local community using adverts<br><br>**Ascertainment/ definition**<br>• Unilateral focal brain lesion (ischaemic or haemorrhagic thought to have occurred between 28 weeks' gestation and 28 days postnatally)<br>• Recruited from a neurologist in San Diego	**Outcomes**<br>• Hearing<br>• Motor (cerebral palsy)<br>• Epilepsy<br><br>**Measurement/ assessment**<br>• Auditory neglect task<br><br>**Follow-up**<br>• 6-14 years (mean 9-10 years)<br>• Completeness not specified	**Time to correct response**<br>**Left sided sound:**<br>Left stroke 1550 ms±580 ms<br>Control 1465 ms±666 ms *not significant*<br><br>Right stroke 1708 ms±951 ms<br>Control 1074 ms±514 ms* (p=0.043)<br><br>**Right sided sound**<br>Left stroke 1595 ms±553 ms<br>Control 1501 ms±720 ms *not significant*<br><br>Right stroke 2032 ms±1496 ms<br>Control 1291 ms±792 ms p=0.118<br><br>**Number of correct auditory responses**<br>**Left sided sound**<br>Left stroke 5.15±1.21<br>Control 4.62±1.26 p=0.338<br><br>Right stroke 4.25±1.67<br>Control 4.63±1.19 p=0.307<br><br>**Right sided sound**<br>Left stroke 4.31±1.18<br>Control 4.62±1.71 p=0.3<br><br>Right stroke 4.50±1.31<br>Control 5.50±0.92 p=0.05<br><br>**Seizures outside of neonatal period**<br>Stroke n=4; 19%<br><br>**Hemiparesis**<br>Stroke n=13, 70%<br><br>Right stroke n=3, 28%<br>Left stroke n=10, 77%
35	Northam 2018[37]<br><br>UK<br><br>Prospective cohort	**Population**<br>• Gestation not provided<br>• Born 1991-2001<br><br>**Exposure (n=30)**<br>• Perinatal stroke<br><br>**Control (n=40)**<br>• Matched on age, sex and maternal education<br>• Term infants<br><br>**Ascertainment/ definition**<br>• Arterial or ischaemic stroke confirmed by MRI in the neonatal period	**Outcomes**<br>• Cognitive<br>• Speech and language<br>• Motor (cerebral palsy)<br><br>**Measurement/ assessment**<br>• WASI<br>• CELF<br>• Comprehensive Test of Phonological Processing<br><br>**Follow-up**<br>• 6-18 years (mean 12.4 and 13.5)<br>• 100% follow up	**Cognitive**<br>**Full scale IQ mean (SD)**<br>Stroke 99 (14)<br>Control 112 (16) p<0.0001<br><br>**Mainstream education**<br>Stroke n=28, 93%<br><br>**Receiving additional education support**<br>Stroke n=12, 40%<br><br>**Speech and language**<br>**Expressive language score, mean (SD)**<br>Stroke 95 (17)<br>Control 108 (13) p=0.001<br><br>**Receptive language score, mean (SD)**<br>Stroke 91 (16)<br>Control 104 (14) p < 0.0001<br><br>**Motor (hemiparesis)**<br>Stroke n=9, 3%
36	Tillema 2008[38]<br><br>USA<br><br>Retrospective cohort	**Population**<br>• Gestation not provided<br>• Birth years not provided<br><br>**Exposure (n=10)**<br>• Left perinatal stroke<br><br>**Control (n=10)**	**Outcomes**<br>• Cognitive<br>• Epilepsy<br><br>**Measurement/ assessment**<br>• WISC-III<br>• Language activation tasks – Verb generation task whilst in an fMRI	**Focal epilepsy**<br>Stroke, n=6, 60%<br><br>**Cognitive, mean (SD)**<br>Stroke VIQ 84 (13.4)<br>Control VIQ 108 (14.2) p=0.002

		• Matched on age, sex, and handedness<br>• Healthy<br>• Randomly drawn from a large database of children recruited for a different study of language development in healthy children<br><br>**Ascertainment/ definition**<br>• Middle cerebral artery ischaemic stroke	**Follow-up**<br>• 6-16 years<br>• 100% follow up	Stroke FSIQ 80 (14.1)<br>Control FSIQ 108 (11.7) p=0.001
37	Trauner 2001[39]<br><br>USA<br><br>Retrospective cohort	**Population**<br>• Gestation not reported<br>• Birth years not reported<br><br>**Exposure (n=39)**<br>• Left perinatal stroke (n=25)<br>• Right perinatal stroke (n=14)<br><br>**Control (n=54)**<br>• Matched on age and socioeconomic status<br>• Normal neurodevelopmental history<br>• Identified from clinics, community adverts, schools<br><br>**Ascertainment/ definition**<br>• Pre or perinatal onset unilateral brain damage (focal lesion) from cerebral infarction or intraparenchymal haemorrhage<br>• Identified through from clinical referrals.<br>• All confirmed by neuroimaging.<br>• Severity rated on 5-point scale adapted from Vargha-Khadem et al.<br>•	**Outcomes**<br>• Behavioural<br>• Cognitive<br>• Epilepsy<br><br>**Measurement/ assessment**<br>• Achenbach CBCL<br>• WPPSI-R (4-5 years)<br>• WISC-R (6-16 years)<br><br>**Follow-up**<br>• 4-18 years<br>• 100% follow up	**<u>Cognitive</u>**<br>**Full scale IQ mean (SD)**<br>Stroke 93.4 (22)<br>Control 116.2 (13) p<0.0001<br><br>Left stroke 90.1 (22)<br>Right stroke 97.4 (22) – no significant difference<br><br>**<u>Seizures (outside of the neonatal period)</u>**<br>Stroke n=17, 50% (missing data for 5 subjects)
**Central nervous system infections**				
38	Bedford 2001[42]<br><br>England & Wales<br><br>Prospective cohort	**Population**<br>• All gestational ages included<br>• Born 1985-1987<br><br>**Exposure (n=274)**<br>• Neonatal meningitis<br><br>**Comparison (n=1391)**<br>• Matched on age and sex<br>• Recruited through GP<br><br>**Ascertainment/ definition**<br>• Identified through clinician reporting	**Outcomes**<br>• Neuromotor disability (composite)<br>• Cognitive<br>• Hearing<br>• Vision<br>• Behaviour<br>• Seizure disorder<br><br>**Assessment/ measurement**<br>• Parental questionnaire<br>• GP questionnaire<br>• McIntyre et al. classification of disability severity<br><br>**Follow-up**<br>• 5 years<br>• 85-94% follow-up	**<u>Neuromotor disability</u>**<br>Meningitis, n=45, 16%<br>No meningitis, n=2, 0.1%<br><br>**Severe disability**<br>Meningitis, n=20, 7%<br>No meningitis, n=1, 0.1%<br><br>**Moderate disability**<br>Meningitis, n=50, 18%<br>No meningitis, n=20, 1%<br><br>**Mild disorder**<br>Meningitis, n=66, 24%<br>No meningitis, n=275, 20%<br><br>**No disability**<br>Meningitis, n=138, 50%<br>No meningitis, n=1095, 79%
39	Horváth-Puhó 2021[43]<br><br>Denmark and Netherlands<br><br>Retrospective matched cohort study	**Population**<br>• Gestation not specified<br>• Born 1997-2017<br><br>**Exposure**<br>• GBS meningitis (Denmark) (n=168)<br>• GBS meningitis (Netherlands) (n=198)<br><br>**Comparison**<br>• Randomly selected<br>• Matched 1:10 on sex, birth year and month, and gestation<br>• No GBS (Denmark) (n=13,689)<br>• No GBS (Netherlands) (n=4,983)<br><br>**Ascertainment/ definition**<br>• Invasive Group B Streptococcal disease by 89 days of age (most were neonatal – hence inclusion)<br>• ICD 10 codes (Denmark)<br>• CSF culture positive on national laboratory register (Netherlands)	**Outcomes**<br>• Neurodevelopmental impairment (composite)<br>• Cognitive<br>• Motor<br>• Behavioural, mental and social disorders<br>• Hearing impairment<br>• Visual impairment<br><br>**Assessment/ Measurement**<br>• ICD 10 codes<br><br>**Follow-up**<br>• Denmark 5 years, 7 years, 10 years, 15 years<br>• Netherlands 5 years, 7 years, 10 years and 11 years<br>• 95% follow-up	**<u>Any neurodevelopmental impairment RR (95%CI)</u>**<br>**<u><5 years</u>**<br>Denmark GBS meningitis 7·80 (4·42-13·77)<br>Netherlands GBS meningitis 5·30 (2·57-10·89)<br><br>**<u><7 years</u>**<br>Denmark GBS meningitis 4·69 (2·78-7·89)<br>Netherlands GBS meningitis 3·71 (1·05-6·72)<br><br>**<u><10 years</u>**<br>Denmark GBS meningitis 3·47 (2·19–5·50)<br>Netherlands GBS meningitis 2·81 (1·69-4·68)<br><br>**<u><11 years</u>**<br>Netherlands GBS meningitis 2·99 (1·83-4·88)<br><br>**<u><15 years</u>**<br>Denmark GBS meningitis 3·15 (1·82–5·46)<br><br>**<u>Moderate to severe neurodevelopmental impairment RR (95%CI)</u>**<br>**<u><5 years</u>**<br>Denmark GBS meningitis 8·49 (4·28-16·86)<br>Netherlands GBS meningitis 5·13 (2·24-11·79)<br><br>**<u><7 years</u>**<br>Denmark GBS meningitis 5·27 (2·80-9·92)<br>Netherlands GBS meningitis n/a<br><br>**<u><10 years</u>**<br>Denmark GBS meningitis 3·88 (2·15–6·99)<br>Netherlands GBS meningitis 3·05 (1·62-5·73)<br><br>**<u><11 years</u>**<br>Netherlands GBS meningitis 3·34 (1·77-6·33)<br><br>**<u><15 years</u>**<br>Denmark GBS meningitis 4·52 (2·35–8·67)
40	Martinez-Cruz 2008[45]	**Population**<br>• Gestation < 34 weeks<br>• Birthweight <1500g	**Outcomes**<br>• Sensorineural hearing loss	**Meningitis**<br>Sensorineural hearing loss: n=15; 10.3%<br>No Sensorineural hearing loss: n=7; 2.6%

	Mexico<br><br>Retrospective case control	• Born 1990-2005<br><br>**Exposure (n=22)**<br>• Neonatal meningitis<br><br>**Comparator (n=374)**<br>• No meningitis<br><br>**Ascertainment/ definition**<br>• Meningitis not defined	**Assessment/ measurement**<br>• Brainstem Auditory Evoked Potentials<br>• Transient Auditory Evoked Otoacoustic Emissions<br>• Tympanometry<br>• Free Field Audiometry<br>• Pure tone audiometry<br>• Behavioural hearing evaluation<br><br>**Follow-up**<br>• 7- 11 years<br>• 100% follow-up	**Odds of previous neonatal meningitis if sensorineural hearing loss**<br>OR 4.368, 95% CI (1.7, 10.9) p= 0.002
41	Stevens 2003[44]<br><br>England & Wales<br><br>Prospective cohort study	**Population**<br>• Term born infants<br>• Born 1985-1987<br><br>**Exposure (n=111)**<br>• Meningitis<br><br>**Comparison (n=162)**<br>• Matched on hospital of birth, birthweight and sex<br>• Hospital control (n=113)<br>• GP control (n=49)<br><br>**Ascertainment/ definition**<br>• CSF positive culture	**Outcomes**<br>• Disability and functional impairment (composite)<br>• Cognitive<br>• Motor<br>• Vision<br>• Hearing<br><br>**Assessment/ measurement**<br>• WISC-III<br>• Movement ABC<br>• Blinded examination<br>• Hearing screening<br>• Sonksen-Silver acuity system<br><br>**Follow-up**<br>• 9-10 years<br>• 67% follow-up of meningitis group	**Cognitive**<br>**IQ, mean (95% CI)**<br>Meningitis, 88.8 (85, 92)<br>Hospital control, 99.4 (97, 102)<br>GP control, 99.6 (95, 103)<br><br>**Motor**<br>**mABC score, mean (95% CI)**<br>Meningitis 7.1 (5.9, 8.5)<br>Hospital controls 5.0 (4.3, 5.8)<br>GP controls 4.0 (2.9, 5.4)<br><br>**Severe disability/ functional impairment**<br>Meningitis, n=12, 10.8%<br>Hospital control, n=0, 0%<br>GP control, n=0, 0%<br><br>**Moderate disability/ functional impairment**<br>Meningitis, n=10, 9%<br>Hospital control, n=2, 1.8%<br>GP control, n=0, 0%<br><br>**Mild disability/ functional impairment**<br>Meningitis, n=19, 17.1%<br>Hospital control, n=13, 11.5%<br>GP control, n=8, 16%<br><br>**No disability or functional impairment**<br>Meningitis, n=70, 63.1%<br>Hospital control, n=98, 86.7%<br>GP control, n=41, 84%<br><br>**Hearing loss (unilateral or bilateral sensorineural hearing loss or requiring hearing aids)**<br>Meningitis, n=4, 3,6%<br>Hospital control, n=0, 0%<br>GP control, n=0, 0%<br><br>**Visual impairment (bilateral)**<br>Meningitis, n= 18, 17% (6 unassessed because of their disability)<br>Hospital control, n=21, 18.5%<br>GP control, n=4, 8%<br><br>**Visual impairment (unilateral)**<br>Meningitis, n= 10, 9.9% (6 unassessed because of their disability)<br>Hospital control, n=8, 7%<br>GP control, n=2, 4%<br><br>**Seizures outside of the neonatal period**<br>Meningitis, n=6, 5.4%<br>Hospital control, n=2, 1.8%<br>GP control, n=0, 0%
Hypoxic-ischaemic encephalopathy				
42	3383 Koc 2016[24]<br><br>Turkey<br><br>Retrospective cohort	**Population**<br>• Gestation < 32 weeks<br>• Birthweight < 1500g<br>• Born 2001<br><br>**Exposure (n=9)**<br>• Perinatal asphyxia<br><br>**Comparator (n=81)**<br>• No asphyxia<br><br>**Ascertainment/ definition**<br>• Perinatal asphyxia diagnosed on: fetal pH, Apgar score, and neonatal cerebral and multiorgan dysfunction	**Outcomes**<br>• Cognitive<br><br>**Assessment/ measurement**<br>• WISC-R<br>• Performed by blinded psychologist<br><br>**Follow-up**<br>• 5-8 years<br>• 100% follow-up	**Cognitive**<br>**WISC-R IQ Score (combined verbal and performance scores) <85**<br>Perinatal asphyxia n=8, 89%<br>No asphyxia n=24, 30%<br>p=0.001
43	Lee-Kelland 2019[46]*<br><br>United Kingdom<br><br>Retrospective cohort study	**Population**<br>• Gestation ≥ 36 weeks<br>• Born 2008-2010<br><br>**Exposure (n=29)**<br>• Moderate-severe HIE without subsequent cerebral palsy<br><br>**Comparator (n=20)**<br>• Matched on age, sex and social class<br>• Born without HIE<br><br>**Ascertainment/ definition**<br>• Received therapeutic hypothermia based on TOBY trial criteria	**Outcomes**<br>• Cognitive<br>• Motor<br>• Speech and language<br>• Behaviour<br><br>**Assessment/ measurement**<br>• WISC IV (blinded)<br>• Movement ABC 2<br>• Strengths and difficulties questionnaire<br><br>**Follow-up**<br>• 6-8 years<br>• 61% follow-up	**Cognitive**<br>**Full scale IQ, mean (SD)**<br>HIE 91 (10.37)<br>No HIE 105 (13.41)<br>Mean difference −13.62 95% CI (−20.53 to −6.71) p<0.001<br><br>**Perceptual reasoning, mean (SD)**<br>HIE 89 (11.15)<br>No HIE 103 (12.49)<br>Mean difference −13.9 95% CI (−20.78 to −7.09) p<0.001<br><br>**Working memory, mean (SD)**<br>HIE 94 (13.76)<br>No HIE 102 (13.82)<br>Mean difference −8.2 95% CI (−16.29 to −0.17) p=0.04

				**Processing speed, mean (SD)**<br>HIE 96 (13.76)<br>No HIE 107 (17.59)<br>Mean difference −11.6 95% CI (−20.69 to −2.47) p=0.01<br><br>**Additional classroom support**<br>HIE n=10, 34%<br>No HIE n=1, 5%<br>OR: 10.0, 95%CI 1.16 to 86.0<br><br>**Special educational needs**<br>HIE n=1, 3.4%<br>No HIE n=0, 0%<br><br>**<u>Motor</u>**<br>**MABC-2 score, mean (SD)**<br>HIE 7.9 (3.26)<br>No HIE 10.2 (2.86)<br>Mean difference −2.12 95% CI (−3.93 to −0.30) p=0.02<br><br>**<u>Speech and language</u>**<br>**Verbal comprehension, mean SD)**<br>HIE 94 (8.79)<br>No HIE 103 (10.09)<br>Mean difference −8.8 95% CI (−14.25 to −3.34) p=0.002<br><br>**<u>Behaviour</u>**<br>**Total difficulties, median (IQR)**<br>HIE 12 (6.5–13.5)<br>No HIE 6 (2.25–10) P=0.005<br><br>**Emotional problems, median (IQR)**<br>HIE 2 (1–4.5)<br>No HIE 0.5 (0–2.75) P=0.03<br><br>**Hyperactivity, median (IQR)**<br>HIE 2 (1–3)<br>No HIE 1 (0–2) P=0.06<br><br>**Conduct problems, median (IQR)**<br>HIE 4 (2.5–6.5)<br>No HIE 3 (1–5) p=0.06<br><br>**Peer problems, median (IQR)**<br>HIE 0 (0–2.5)<br>No HIE 0 (0–1) p=3.56 Ω (potential error in manuscript table)<br><br>**Prosocial, median (IQR)**<br>HIE 9 (7.5–10)<br>No HIE 9 (8.25–10) p=0.13<br><br>**Impact score, median (IQR)**<br>HIE 0 (0–2.5)<br>No HIE 0 (0–2.0) p=0.31
44	Tonks 2019[47]*<br><br>United Kingdom<br>Prospective cohort study	**Population**<br>• Gestation ≥36 weeks<br>• Born 2008-2011<br>• English as primary language<br><br>**Exposure (n=29)**<br>• Moderate-severe HIE without subsequent cerebral palsy<br><br>**Comparator (n=20)**<br>• Matched on age, sex and social class<br>• Recruited from schools in the area<br>• Born without HIE<br><br>**Ascertainment/ definition**<br>• Received therapeutic hypothermia based on TOBY trial criteria	**Outcomes**<br>• Cognitive<br>• Neuropsychological<br><br>**Assessment/ measurement**<br>• Conner's continuous performance test<br>• NEPSY-II block construction test<br>• NEPSY-II arrows' test<br><br>**Follow-up**<br>• 6-8 years<br>• 77% follow-up	**<u>Attention</u>**<br>**Hit response time**<br>**HIE**<br>84.1 percentile mean rank 27;<br>Proportion performing below 2 SD 32%<br><br>**Comparator**<br>67.3 percentile mean rank 17.89; p = .024<br>Proportion performing below 2 SD 11%<br><br>**Hit response time standard error**<br>**HIE**<br>standard error mean rank 26.8<br>Proportion performing below 2 SD 18%<br><br>**Comparator**<br>standard error mean rank 18.2; p = 0.032<br>Proportion performing below 2 SD 11%<br><br>**Hit response time by block**<br>**HIE**<br>Mean 49.1, SD 23.9<br><br>**Comparator**<br>Mean 61.9, SD 18.4; p = 0.047<br><br>**<u>Visual discrimination</u>**<br>**<u>HIE</u>**<br>Below 1 SD 10%<br><br>**<u>Comparator</u>**<br>Below 1 SD 5%<br>HIE vs comparator scores, p = 0.049<br><br>**<u>Visuo-spatial mental rotation task</u>**<br>**<u>HIE</u>**<br>Below 1 SD 17%<br><br>**<u>Comparator</u>**<br>Below 1 SD 5%<br>HIE vs comparator scores, p = 0.034
