# Supplementary material for: School-age outcomes of children after perinatal brain injury: a systematic review and meta-analysis
Source: BMJ Paediatr Open. 2023 Jun 2;7(1):e001810. doi: 10.1136/bmjpo-2022-001810 (PMC10255042; doi:10.1136/bmjpo-2022-001810)
Supplement: Supplementary data [file bmjpo-2022-001810supp004.pdf]

BMJ Publishing Group Limited (BMJ) disclaims all liability and responsibility arising from any reliance placed on this supplemental material which has been supplied by the author(s)

Supplement 4: Risk of bias table

overlapping data; Clinical Evaluation of Language Fundamentals (CELF); Cystic Periventricular leukomalacia (cPVL); Intelligence Quotient (IQ); Intraventricular haemorrhage (IVH); Mental Developmental Index (MDI); Neonatal Intensive Care Unit (NICU); Psychomotor Development Index (PDI); Periventricular leukomalacia (PVL); Spontaneous Intestinal Perforation (SIP); Wechsler Intelligence Scale for Children (WISC); White Matter Injury (WMI);

Preterm brain injury: cohort studies

	Selection (*satisfactory; No =not satisfactorily done; n/a)				Comparability (*satisfactory; No =not satisfactorily done; n/a)		Exposure/ Outcome (*satisfactory; No =not satisfactorily done; n/a)			Subtotal assessment			Total score: 0-3 high risk of bias; 4-6 moderate risk of bias 7-9 low risk of bias	Additional comments
	1	2	3	4	1a	1b	1	2	3	Selection (0-1=Poor; 2=Fair; 3+ Good)	Comparability (0=poor; 1=fair; 2+=good)	Exposure / outcome (0=poor; 1=fair; 2+=good)		
Adant 2019	No	*	*	* (excluded those with congenital anomalies)	*	*	No	*	No	Good	Good	Fair	6	Population not representative as focus of study was spontaneous intestinal perforation. Infants without IVH didn't have brain injury excluded per se (but didn't have IVH 3-4 on imaging). Matched on gender, gestational age, date of birth. Multiples matched to sibling without SIP. Excluded those with necrotising enterocolitis, mechanical obstruction or congenital anomalies. Adjusted for gender, gestation, birthweight, SIP and IVH.<br><br>Independent outcome assessment but not blinded; telephone survey of parents. High numbers lost to follow-up. Table 3 contains errors with respect to outcomes (MDI and PDI mislabelled as motor and cognitive respectively).

Beaino 2010#	*	*	No	* (cerebral palsy could not be present at birth)	*	*	*	*	*	Good	Good	Good	8	3% of infants did not have a cranial ultrasound, a further 11% had only one cranial ultrasound during neonatal period - therefore ascertainment of exposure may be compromised<br><br>Model A adjusted for:<br>• obstetric factors<br>• cerebral lesions<br>Model B adjusted for:<br>• obstetric factors<br>• neonatal factors<br><br>Model C was the same as model B for those without cPVL or Intraparenchymal haemorrhage<br><br><85% follow-up for enrolled infants but clear description of those lost to follow-up and no significant differences with respect to ultrasound brain injury findings between groups
Brouwer 2012	No	No	*	* (given the types of outcomes assessed)	No	No	No	*	*	Fair	Poor	Good	4	Study of a select group i.e. those with IVH requiring neurosurgical intervention.<br>No description of setting, how patients were enrolled, how many were excluded<br>No description of how control group was derived, or what era they were from.<br>Only some infants (those <30weeks) were matched on gestation, birthweight, sex to controls.<br>Different intelligence tests used at follow-up. >80% completion rate of Child Behaviour Checklist and teacher report form by parents and teachers

Campbell 2021	*	*	*	* (given the types of outcomes assessed)	*	*	*	*	No	Good	Good	Good	8	Males and those born at 23-24 weeks gestation were overrepresented in the IVH WMI group.<br>Adjusted for gestation, birthweight Z score, sex, maternal education, bronchopulmonary dysplasia, sepsis, necrotising enterocolitis (Bell stage 2-3) and severe retinopathy of prematurity.
Cheong 2018	*	*	*	No (visual or hearing impairment could be congenital)	*	*	*	*	*	Good	Good	Good	8	Adjusted for era of birth, antenatal corticosteroid exposure, inborn status, gestation, sex, multiple birth, birthweight Z score, surfactant use, IVH grade 3 or 4 (in cPVL), cPVL (in IVH grade 3-4), bronchopulmonary dysplasia, postnatal corticosteroid use, necrotising enterocolitis (stage 2 or worse), surgery in the newborn period, and retinopathy of prematurity (stage 3 or worse).
Chou 2020	*	*	*	* (given the types of outcomes assessed)	No	*	*	*	No	Good	Fair	Good	7	Matched and adjusted for, urbanisation and parental occupation.<br><br>No information about missing data or completeness of follow-up

Davidovitch 2020	*	*	*	* (given the types of outcomes assessed)	No	*	*	*	No	Good	Fair	Good	7	Only low birthweight infants included (therefore birthweight partially accounted for). Unmatched.<br>No information about excluding brain injury from comparators e.g. comparing those with IVH grade 3-4 to those without could include those with IVH 1-2; both groups could also include infants with other types of brain injury.<br>Missing data not presented or accounted for. Adjusted the composite brain injury group (which included retinopathy of prematurity in its definition) for gestation, maternal diabetes, small for gestational age, year of birth, bronchopulmonary dysplasia, and receipt of postnatal steroids.
Doyle 2000 #	*	*	*	* (given the types of outcomes assessed)	No	No	*	*	*	Good	Poor	Good	7	IVH and no IVH groups not matched for gestation or birthweight, no adjustment for these variables appears to have been done.<br><br>Relatively old cohort (most did not receive surfactant), comparator group only includes infants born in the 1980s. Not representative due to time-period of care.
Hintz 2018	*	*	*	* (given the types of outcomes assessed)	*	*	*	*	*	Good	Good	Good	9	Assessed interobserver reliability of central imaging readers.<br><br>Unmatched<br><br>Adjusted for gestation, race, sex, multiple gestation, maternal education, sepsis, bronchopulmonary dysplasia, postnatal steroids, surgery for patent ductus arteriosus, necrotising enterocolitis, retinopathy of prematurity.<br><br>Only 83% follow-up of survivors but those lost to follow-up are accounted for.

Hirovonen 2017	*	*	*	* (given the types of outcomes assessed)	*	*	*	*	*	Good	Good	Good	9	Excluded infants who died at <1 year of age, infants with major congenital anomalies, and those with missing data.<br><br>Characteristics of those with brain injury not presented.<br><br>No breakdown by severity of brain injury because that level of detail was not available in the database.<br><br>No matching but there is stratification by gestation and adjustment for: maternal characteristics, pregnancy characteristics, delivery characteristics, sex, gestation, birthweight, Apgar score at 1-minute, umbilical artery pH, resuscitation provided, NICU admission, receipt of phototherapy, ventilator requirement, antibiotic receipt, respiratory distress syndrome, sepsis, seizures, hyperbilirubinaemia.
Hollebrandse 2021	*	*	*	* (given the types of outcomes assessed)	*	*	*	*	*	Good	Good	Good	9	Gestation similar across all groups and other baseline perinatal characteristics similar across groups.<br><br>Preterm brain injury and no brain injury group not matched. Unclear if IVH and no IVH group had other brain injuries excluded or may have had more than one injury type (e.g. PVL).<br>Impact of epoch/ era of birth explored and adjusted for.
Hreinsdottir 2018	*	*	*	No (visual impairment could have been congenital)	*	*	*	*	No	Good	Good	Good	7	Unsure if comparator group in logistic regression includes those with IVH 1-2.<br>Adjusted for gestation, birthweight, retinopathy of prematurity, sex, cognitive score, cerebral palsy.

Jansen 2020	*	*	*	* (given the types of outcomes assessed)	No	No	*	*	No	Good	Poor	Good	6	Excluded infants with congenital abnormalities, metabolic disorders or neonatal meningitis.
Kaur 2020	*	*	*	No (visual or hearing impairment could be congenital)	No	No	*	*	No	Good	Poor	Good	5	Unmatched. Compared IVH with all infant without haemorrhage (of all gestations).
Kiechl-Kohlendorfer 2013	*	*	*	* (given the types of outcomes assessed)	*	*	*	No	No	Good	Good	Fair	7	Low numbers of infants included. Outcomes assessed at 1 year - likely not long enough for robust assessment of neurodevelopmental outcomes; <85% follow-up and no detailed description of those lost to follow up - though authors do state that there were no significant differences between those followed up and those lost to follow up.
Klebermass-Schrehof 2012	*	*	*	No (could have had congenital blindness)	*	*	*	*	No	Good	Good	Good	7	Adjusted for gestation. No clear description of number lost to follow-up, though mentions that follow-up rate at 5.5 years was 54-61%.
Koc 2016	*	*	No	* (given the types of outcomes assessed)	No	No	*	*	No	Good	Poor	Good	5	Small numbers included. No breakdown of characteristics of those with brain injury. No description of IVH grading used or schedule of ultrasound exams; no description of criteria for establishing perinatal asphyxia, number lost to follow-up not stated.
Neubauer 2008	*	n/a	*	No (deafness or blindness could have been congenital)	*	*	*	*	*	Fair	Good	Fair	7	Neurodevelopmental assessors not blinded; follow-up rate <85% but paper does give description of those lost to follow-up

Piris Borregas 2019	*	*	*	* (excluded infants with congenital malformations)	No	No	*	*	No	Good	Poor	Good	6	Only those followed up to 7 years included.<br><br>Excluded infants who died before 36 weeks corrected age, with major malformations, or those with missing data.<br><br>Unclear if independent odds ratio includes adjustment for covariates.<br><br>Unclear if those without 'severe brain injury' had other types of brain injury.
Pittet 2019	*	*	*	* (excluded infants with congenital malformations)	No	*	*	*	*	Good	Fair	Good	8	Excluded infants with congenital malformations affecting neurodevelopment and infants from centres without 5 years of follow-up cognitive testing.<br><br>Unclear if other types of brain injury excluded from comparator group.<br><br>Adjusted for gender and socioeconomic status. No significant difference in cognitive outcome between extreme preterms and those 28-30 weeks' gestation. Gestation not adjusted for.
Sherlock 2005#	*	*	*	No (deafness or blindness could have been congenital)	No	No	*	*	*	Good	Poor	Good	6	Comparability of IVH vs. no IVH cohorts not clear - not enough information to determine if groups were comparable with respect to gestational age or birthweight
Tymofiyev a 2018	*	*	*	* (given the types of outcomes assessed)	No	No	*	*	No	Good	Poor	Good	6	Excluded infants with congenital malformations/ syndromes, congenital infections, or those who were too unstable for MR imaging. The last exclusion criteria in particular could limit generalisability quite considerably.<br><br>Unclear about the validity of grouping the attention scores across different assessment tools together into a dichotomous variable for attention.

Van De Bor 2004	*	*	*	* (excluded those with major congenital malformations)	*	*	No	No	*	Good	Good	Fair	7	IVH vs. no IVH cohorts comparable with respect to gestation; some differences in gender composition but paper states this was controlled for in the analysis. Primary outcome entirely self-reported. Outcomes reported at 14 years.
Van Den Hout 2000	* (except for HIE exposure group)	*	*	* (excluded those with congenital anomalies)	No	No	*	*	*	Good	Poor	Good	7	Low numbers and relatively old cohort. Relative gender imbalance in IVH group compared to those with normal scans or PVL. IVH group also 1.4 weeks more premature than 'normal scan' group.
Vollmer 2003#	*	*	*	No (deafness or blindness could have been congenital)	*	No	*	*	*	Good	Fair	Good	7	Note change in version of Weschler scale during follow-up period. Authors state no difference in mean IQ after change. Baseline characteristics of groups with and without brain injury not given; no indication of matching or adjustment for factors other than gestation.
Vollmer 2006a#	*	*	*	No (deafness or blindness could have been congenital)	*	*	*	*	*	Good	Good	Good	8	Note gender imbalance in cohort as a whole (M>F), but male: female ratio in each group appears similar.<br>No matching or adjustment for covariates.<br><br><85% follow-up but clear description of those lost and appears no significant differences.
Vollmer 2006b#	*	*	*	No (deafness or blindness could have been congenital)	No	No	*	*	No	Good	Poor	Good	5	Marked gender imbalance in ventricular dilatation group. Lower birthweight and gestation in groups with abnormal cranial ultrasound. No indication of matching or adjustment.<br><br><85% follow-up and the limited description of those lost to follow-up indicates that these babies were of lower birthweight and gestation.

Whitaker 2011	*	*	*	* (given the types of outcomes assessed)	*	*	(No)	*	*	Good	Good	Good	8	Severely disabled survivors (n=33) were excluded.<br><br>Half had later ultrasounds (just before discharge).<br><br>No breakdown of the characteristics of the exposed and comparator groups – unable to assess how comparable they are.<br><br>Adjusted for: maternal social risk, sex, gestation, fetal growth ratio, multiplicity, maternal smoking status, maternal alcohol status, labour onset, presentation at birth, base excess on first postnatal blood gas, thyroid status, hypocapnia, hypoxia, systolic hypotension, prolonged ventilation.<br><br>Primary outcome assessment reliant on parental report, albeit via structured interview with some evidence for validity. Interviewers were blinded to the child's history. Parents were blinded to the study hypothesis.<br><br>Less than 85% follow-up (psychiatric interviews in 51% of survivors) however clear descriptions of groups with and without psychiatric evaluation given in table 2 and little apparent difference between groups.
**Preterm brain injury: case-control studies**														
	1 Case definition	2 Representativeness of cases	3 Selection of controls	4 Definition of controls	1a	1b	1 Ascertainment of exposure	2 Same method of ascertainment for cases and controls	3 Non-response rate	(0-1=Poor; 2=Fair; 3+ Good)	y (0=poor; 1=fair; 2+=good)	(0=poor; 1=fair; 2+=good)	Total score: 0-3 high risk of bias; 4-6 moderate risk of bias 7-9 low risk of bias	Additional comments

Martinez-Cruz 2008 (IVH)	*	*	*	*	*	No	*	*	No	Good	Fair	Good	7	Appears to be case-control design hence star ratings are as per case control rating sheet. Controls not well matched for birth weight. No description of whether full information on exposures could be obtained for all cases/controls e.g. missing records etc.
Perinatal stroke: cohort studies														
	Selection (*satisfactory; No =not satisfactorily done; n/a)				Comparability (*satisfactory; No =not satisfactorily done; n/a)		Exposure/ Outcome (*satisfactory; No =not satisfactorily done; n/a)			Subtotal assessment			Total score: 0-3 high risk of bias; 4-6 moderate risk of bias 7-9 low risk of bias	Additional comments
	1	2	3	4	1a	1b	1	2	3	Selection (0-1=Poor; 2=Fair; 3+ Good)	Comparability (0=poor; 1=fair; 2+=good)	Exposure / outcome (0=poor; 1=fair; 2+=good)		
Ballantyne 2007	No	No	*	*	No	*	No	*	No	Fair	Fair	Fair	4	No description of derivation of exposed cohort - whether single institute or multicentre, whether same community as non-exposed group or not.<br><br>Predominance of right-handed children amongst controls otherwise similar baseline characteristics. Note male preponderance in exposed group and female preponderance in non-exposed<br><br>No matching or adjustment for confounders.<br><br>No description of who performed outcome assessment, whether blinded and independent.
Ballantyne 2008	*	*	*	No	No	*	*	*	No	Good	Fair	Good	6	Excluded children with brain lesions from other causes e.g. head trauma, tumours

														Gestational age of exposed cohort ranged from 32 to 40 weeks. No statement as to whether control group were matched on this. Note preponderance of males in stroke group and females in control group.<br><br>In study 1, significant numbers of participants did not complete the planned developmental assessments - across exposed and control groups, completeness ranged from 50% for WISC-R to 69% for CELF-R.
Gold 2014	No	No	*	*	No	*	*	*	*	Fair	Fair	Good	6	No description of how subjects were selected or recruited from neurology clinics. Nonexposed group selected from a different source. No description of gestational age of subjects or of controlling for this. Matched for age at follow up, sex, socioeconomic group and maternal education.<br><br>Excluded infants with bilateral lesions, a history of hypoxic ischemic encephalopathy, central nervous system infection, in-utero drug exposure, significant closed head injury, or any other condition that might have caused brain damage other than from the stroke.
Kolk 2011	*	*	*	*	No	*	*	*	No	Good	Fair	Good	7	No description of gestational age of subjects or of controlling for this. Difficult to ascertain completeness of follow-up from paper. Adjusted for age of outcome assessment.
Martin 2019	*	*	*	*	No	*	*	*	*	Good	Fair	Good	8	Excluded infants with bilateral lesions, hearing impairment, or a history of a problem that may have caused more global brain damage (e.g. meningitis, closed head injury, hypoxic-ischemic encephalopathy). Matched on age, sex and socioeconomic status

Northam 2018	*	No	*	*	*	*	*	*	*	Good	Good	Good	8	No description of source of unexposed cohort. Matched on age, sex, and maternal education.
Tillema 2008	*	*	*	*	No	*	*	*	No	Good	Fair	Good	7	Exposed and comparator groups not matched for gestation, but were matched for age, sex and handedness. 17 subjects included initially but 7 of these excluded for various reasons meaning that neurodevelopmental outcome data/Weschler scores only presented for 10 of 17.
Trauner 2013	*	*	*	*	No	No	No	*	No	Good	Poor	Fair	5	Excluded infants if bilateral or multifocal lesions identified, history of meningitis, or history of antenatal drug exposure<br><br>Matched on age and socioeconomic status<br><br>No baseline characteristics given to establish comparability of exposed and comparator cohorts. Likely comparable with regards to gestation based on stated inclusion criteria. Main outcome measure based on parental questionnaire - no direct linguistic assessments done, however may not have been feasible/appropriate in such a young cohort. No information on response rate/loss to follow-up.<br><br>IQ used as covariate<br><br>IQ combined across the age range and assessed with two different tools. This assumes IQ is fixed which may not be true.
Central nervous infections: cohort studies														

	Selection (*satisfactory; No =not satisfactorily done; n/a)				Comparability (*satisfactory; No =not satisfactorily done; n/a)		Exposure/ Outcome (*satisfactory; No =not satisfactorily done; n/a)			Subtotal assessment			Total score: 0-3 high risk of bias; 4-6 moderate risk of bias 7-9 low risk of bias	Additional comments
	1	2	3	4	1a	1b	1	2	3	Selection (0-1=Poor; 2=Fair; 3+ Good)	Comparability (0=poor; 1=fair; 2+=good)	Exposure / outcome (0=poor; 1=fair; 2+=good)		
Bedford 2001#	*	*	*	No	*	*	No	*	*	Good	Good	Good	7	Matched on sex and age.<br><br>Study focuses on meningitis in infancy but also presents outcomes after neonatal meningitis.<br><br>Did not exclude children with other comorbidities e.g. congenital conditions associated with neurodevelopmental impairment. Exposed cases derived from same cohort as Stevens 2003. Outcome assessment based on parent or GP report with no formal neurodevelopmental assessment.
Horváth-Puhó 2021	*	*	*	No	*	*	*	*	*	Good	Good	Good	8	Invasive Group B Streptococcal infection diagnosed in the first 89 days (however most of these were neonatal, particularly in the first week of life (45%) hence inclusion.<br><br>Matched 1:10 on sex, birth year and month, and gestation.<br>Neurodevelopmental impairment defined differently in each cohort.<br>Missing data accounted for and its impact explored.

Stevens 2003#	(*)	(*)	*	No	*	*	*	*	No	Good	Good	Good	7	Exposed cohort based on recall of consultant paediatricians filling out monthly returns thus may be biased towards more severe or otherwise memorable cases. Some in comparator group selected from a different hospital than exposed cohort.<br><br>Matched on hospital of birth, birth weight and sex.<br><br>Results stratified by birthweight<br><br>Significant rate of loss to follow-up.
**Central nervous system infections: case control studies**														
	1 Case definition	2 Representativeness of cases	3 Selection of controls	4 Definition of controls	1a	1b	1 Ascertainment of exposure	2 Same method of ascertainment for cases and controls	3 Non-response rate	(0-1=Poor; 2=Fair; 3+ Good)	y (0=poor; 1=fair; 2+=good)	(0=poor; 1=fair; 2+=good)	Total score: 0-3 high risk of bias; 4-6 moderate risk of bias 7-9 low risk of bias	Additional comments
Martinez-Cruz 2008	*	*	*	*	No	No	*	*	No	Good	Poor	Good	6	Excluded those with history of parental consanguinity or TORCH infections.<br><br>Number of those with and without meningitis who may have had other types of brain injuries not specified – unable to assess overlap/ impact of meningitis alone.<br><br>Odds ratio presented for meningitis does not appear to be crude so potential adjustment for confounding factors but no description of this in the methods section.<br><br>No description of proportion of missing data.
**Hypoxic-ischaemic encephalopathy: cohort studies**														

	Selection (*satisfactory; No =not satisfactorily done; n/a)				Comparability (*satisfactory; No =not satisfactorily done; n/a)		Exposure/ Outcome (*satisfactory; No =not satisfactorily done; n/a)			Subtotal assessment			Selection (*satisfactory; No =not satisfactorily done; n/a)	Additional comments
	1	2	3	4	1a	1b	1	2	3	Selection (0-1=Poor; 2=Fair; 3+ Good)	Comparability (0=poor; 1=fair; 2+=good)	Exposure / outcome (0=poor; 1=fair; 2+=good)	Total score: 0-3 high risk of bias; 4-6 moderate risk of bias 7-9 low risk of bias	
Koc 2016	No	*	*	*	No	No	*	*	No	Fair	Poor	Good	5	Representativeness not clear as no description given of babies who did not complete follow-up at the study institution. No apparent adjustment for gestation or other covariates. Pre-therapeutic hypothermia era.<br><br>Small number, no breakdown of characteristics or other neurodevelopmental outcomes by brain injury<br><br>Number of those with and without birth asphyxia who had other types of brain injuries e.g. IVH not specified.
Lee-Kelland 2019	No	*	*	*	*	*	*	No	No	Good	Good	Good	6	Excluded those who underwent therapeutic hypothermia outside of the standard criteria, infants with metabolic disorders and non-English speaking infants.<br><br>Matched on age, sex and social class.
Tonks 2019	*	No	*	*	No	*	*	*	No	Good	Fair	Good	6	Included cases had no diagnoses other than encephalopathy.<br>Excluded infants with neurological issues other than encephalopathy. Matched on age, sex and socioeconomic status.
