# Supplementary figures and images for: School-age outcomes of children after perinatal brain injury: a systematic review and meta-analysis
Source: BMJ Paediatr Open. 2023 Jun 2;7(1):e001810. doi: 10.1136/bmjpo-2022-001810 (PMC10255042; doi:10.1136/bmjpo-2022-001810)

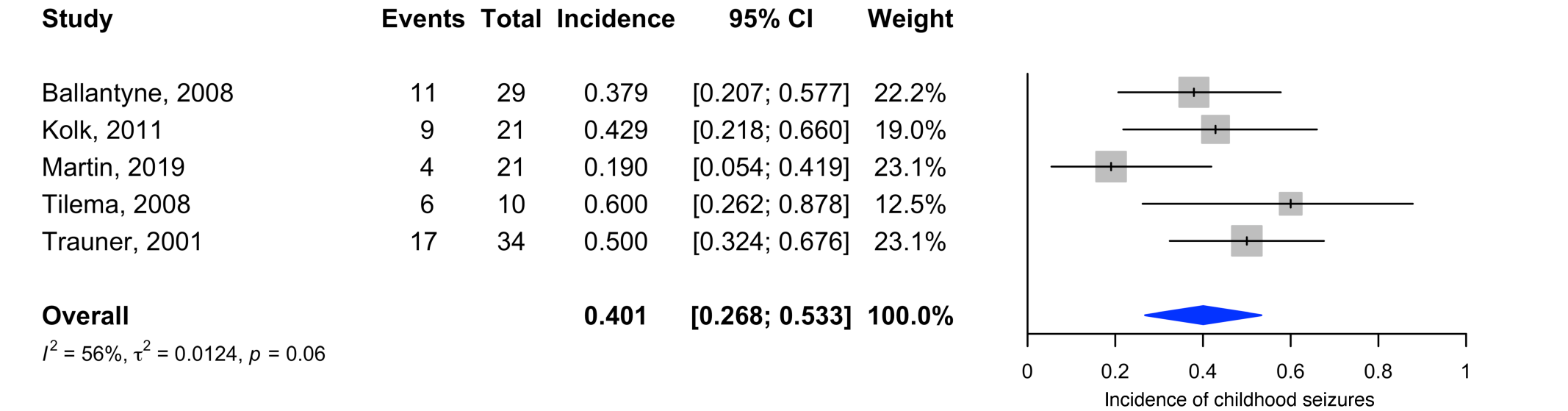

Supplement: Supplementary data [file bmjpo-2022-001810supp005.pdf]

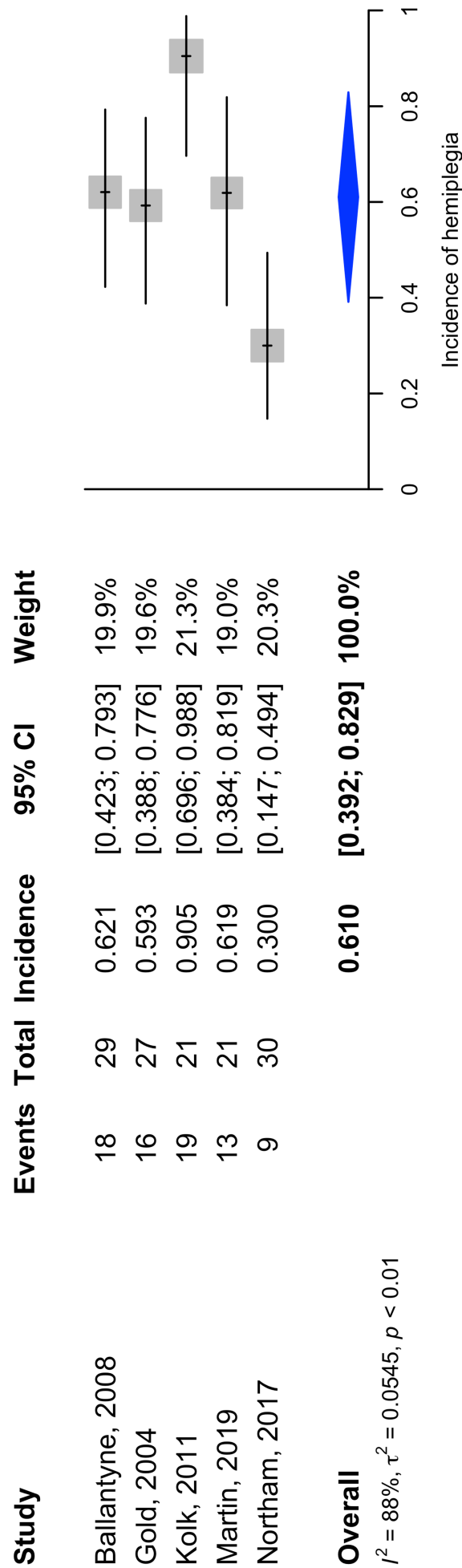

Supplement: Supplementary data [file bmjpo-2022-001810supp006.pdf]

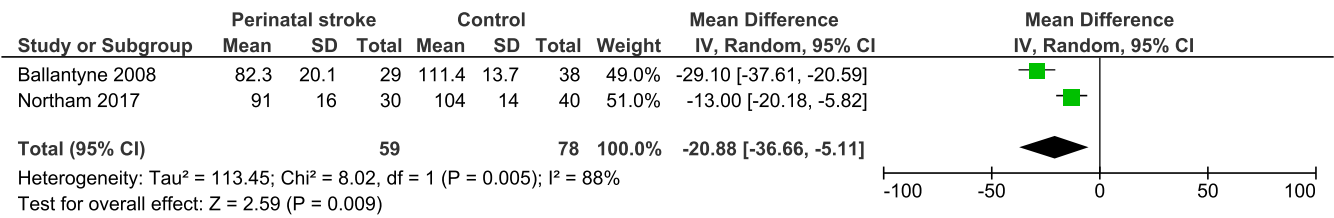

Supplement: Supplementary data [file bmjpo-2022-001810supp007.pdf]

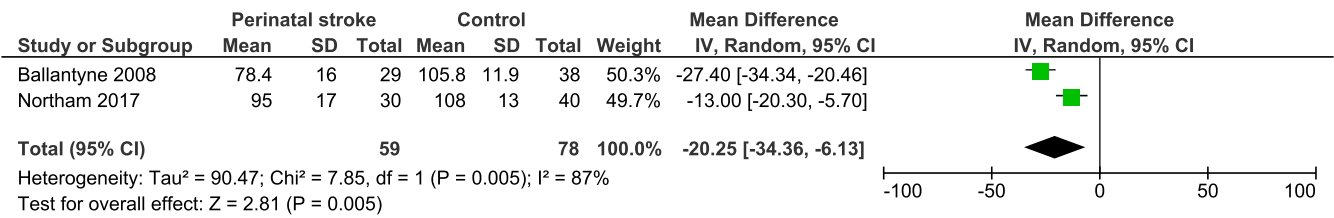

Supplement: Supplementary data [file bmjpo-2022-001810supp008.pdf]
