# Supplementary material for: School-age outcomes of children after perinatal brain injury: a systematic review and meta-analysis
Source: BMJ Paediatr Open. 2023 Jun 2;7(1):e001810. doi: 10.1136/bmjpo-2022-001810 (PMC10255042; doi:10.1136/bmjpo-2022-001810)
Supplement: Author's manuscript [file bmjpo-2022-001810.draft_revisions.pdf]

BMJ Paediatrics Open

BMJ Paediatrics Open is committed to open peer review. As part of this commitment we make the peer review history of every article we publish publicly available.

When an article is published we post the peer reviewers' comments and the authors' responses online. We also post the versions of the paper that were used during peer review. These are the versions that the peer review comments apply to.

The versions of the paper that follow are the versions that were submitted during the peer review process. They are not the versions of record or the final published versions. They should not be cited or distributed as the published version of this manuscript.

BMJ Paediatrics Open is an open access journal and the full, final, typeset and author-corrected version of record of the manuscript is available on our site with no access controls, subscription charges or pay-per-view fees (http://bmjpaedsopen.bmj.com).

If you have any questions on BMJ Paediatrics Open's open peer review process please email info.bmjpo@bmj.com

BMJ Paediatrics Open

School-age outcomes of children after perinatal brain injury: a systematic review and meta-analysis

Journal:	*BMJ Paediatrics Open*
Manuscript ID	bmjpo-2022-001810
Article Type:	Original research
Date Submitted by the Author:	06-Dec-2022

Complete List of Authors:	Rees, Philippa; University College London Institute of Child Health, Population policy and Practice<br>Callan, Caitriona; University of Oxford Nuffield Department of Primary Care Health Sciences<br>Chadda, Karan; Cambridge University Hospitals NHS Foundation Trust, Department of Paediatrics<br>Vaal, Meriel; University College London Institute of Child Health, University College London and Great Ormond Street Institute of Child Health<br>Diviney, James; Great Ormond Street Hospital for Children NHS Foundation Trust, Paediatric Intensive Care Unit<br>Sabti, Shahad; King's College London<br>Harnden, Fergus; Chelsea and Westminster Hospital NHS Foundation Trust<br>Gardiner, Julian; University College London Institute of Child Health, University College London and Great Ormond Street Institute of Child Health<br>Battersby, Cheryl; Imperial College London, Neonatal Medicine<br>Gale, Chris; Imperial College London, Neonatal Medicine<br>Sutcliffe, Alastair; University College London Institute of Child Health, University College London and Great Ormond Street Institute of Child Health

Keywords:	Epidemiology, Neonatology

SCHOLARONE™
Manuscripts

I, the Submitting Author has the right to grant and does grant on behalf of all authors of the Work (as defined in the below author licence), an exclusive licence and/or a non-exclusive licence for contributions from authors who are: i) UK Crown employees; ii) where BMJ has agreed a CC-BY licence shall apply, and/or iii) in accordance with the terms applicable for US Federal Government officers or employees acting as part of their official duties; on a worldwide, perpetual, irrevocable, royalty-free basis to BMJ Publishing Group Ltd ("BMJ") its licensees and where the relevant Journal is co-owned by BMJ to the co-owners of the Journal, to publish the Work in this journal and any other BMJ products and to exploit all rights, as set out in our licence.

The Submitting Author accepts and understands that any supply made under these terms is made by BMJ to the Submitting Author unless you are acting as an employee on behalf of your employer or a postgraduate student of an affiliated institution which is paying any applicable article publishing charge ("APC") for Open Access articles. Where the Submitting Author wishes to make the Work available on an Open Access basis (and intends to pay the relevant APC), the terms of reuse of such Open Access shall be governed by a Creative Commons licence – details of these licences and which Creative Commons licence will apply to this Work are set out in our licence referred to above.

Other than as permitted in any relevant BMJ Author's Self Archiving Policies, I confirm this Work has not been accepted for publication elsewhere, is not being considered for publication elsewhere and does not duplicate material already published. I confirm all authors consent to publication of this Work and authorise the granting of this licence.

Confidential: For Review Only

School-age outcomes of children after perinatal brain injury: a systematic review and meta-analysis

Philippa Rees[1] MPhil MBBCh, Caitriona Callan[2] MB BChir, Karan R Chadda[3] MB BChir, Meriel Vaal MRes MBChB[1], James Diviney[4] MB BChir, Shahad Sabti[5] MBBS, Fergus Harnden[6] MBChB, Julian Gardiner[1] PhD, Cheryl Battersby[7] PhD, Chris Gale[7] PhD, Alastair Sutcliffe[1] PhD

Affiliations:
1. Population Policy and Practice, Great Ormond Street UCL Institute of Child Health, London, UK.

2. Nuffield Department of Primary Care Health Sciences, University of Oxford.

3. Department of Paediatrics, Cambridge University Hospitals NHS Foundation Trust, Cambridge, UK.

4. Paediatric Intensive Care Unit, Great Ormond Street Hospital, London, UK

5. Kings College London, UK.

6. Chelsea and Westminster Hospital NHS Foundation Trust, London, UK.

7. Neonatal Medicine, School of Public Health, Faculty of Medicine, Imperial College London, London, UK.

Address correspondence to: Dr Philippa Rees, Population Policy Practice, UCL Institute of Child Health, 1st Floor 30 Guilford Street, London, WC1N 1EH, p.rees@ucl.ac.uk

Confidential: For Review Only

School-age outcomes of children after perinatal brain injury: a systematic review and meta-analysis

Background

Over 3,000 children suffer a perinatal brain injury in England every year according to national surveillance. The childhood outcomes of infants with perinatal brain injury are however unknown.

Methods

A systematic review and meta-analyses were undertaken to explore school-aged neurodevelopmental outcomes of children after perinatal brain injury compared to those without perinatal brain injury. The primary outcome was neurodevelopmental impairment which included cognitive, motor, speech and language, behavioural, hearing, or visual impairment after 5 years of age.

Results

This review included 42 studies. Preterm infants with intraventricular haemorrhage (IVH) grade 3-4 were found to have a three-fold greater risk of moderate-severe neurodevelopmental impairment at school age OR 3.69 (95%CI: 1.7, 7.98). Infants with perinatal stroke had an increased incidence of hemiplegia 61% (95%CI: 39.2, 82.9) and an increased risk of cognitive impairment (difference in full scale IQ -24.2 (95%CI: -30.73, -17.67) . Perinatal stroke was also associated with poorer academic performance; and lower receptive -20.25 (95%CI: -34.36, -6.13) and expressive language scores -20.25 (95%CI: -34.36, -6.13). Studies reported an increased risk of persisting neurodevelopmental impairment at school age after neonatal meningitis. Cognitive impairment and special educational needs were highlighted after moderate-severe HIE. However, there were limited comparative studies providing school-aged outcome data across neurodevelopmental domains and few provided adjusted data. Findings were further limited by the heterogeneity of studies.

Conclusions

Longitudinal population studies exploring childhood outcomes after perinatal brain injury are urgently needed to better enable clinicians to prepare affected families, and to facilitate targeted developmental support to help affected children reach their full potential.

Confidential: For Review Only

School-age outcomes of children after perinatal brain injury: a systematic review and meta-analysis

What is already known on this topic

Thousands of children suffer a brain injury around the time of birth every year in England. Many of these injuries are associated with neurodevelopmental impairment at two years of age. However, two-year outcomes are not necessarily representative of later childhood outcomes and function, which are a priority for parents.

What this study adds

This review provides an overview of existing evidence of childhood outcomes after perinatal brain injury. It indicates that there is some evidence of on-going impairment throughout childhood for different types of perinatal brain injury but that there are considerable gaps in knowledge.

How this study might affect research, practice or policy

This review shows the need for detailed high-quality longitudinal population studies exploring childhood outcomes after perinatal brain injury

Confidential: For Review Only

School-age outcomes of children after perinatal brain injury: a systematic review and meta-analysis

Perinatal brain injuries can have wide-ranging deleterious consequences for children, families and broader society.(1-4) Over 3,000 infants experience perinatal brain injury in England annually[1] and the Department of Health and Social Care (DHSC) has committed to halving the rate of perinatal brain injuries by 2030 as part of the national maternity ambition.(5) To monitor progress towards this goal, a standardised definition of perinatal brain injury was developed. This definition – which encompasses moderate to severe Hypoxic Ischaemic Encephalopathy (HIE), perinatal stroke, central nervous system infections (CNS), kernicterus, intraventricular haemorrhage (IVH) grade 3-4, and cystic periventricular leukomalacia – includes 'indicators' of such injuries during the neonatal period.(6) The degree to which this definition captures and represents true perinatal brain injuries is unclear and requires us to look beyond the neonatal period.(6)

Focusing on the childhood outcomes of infants with perinatal brain injury provides a fuller understanding of the population captured by the DHSC definition. Despite their importance to families, school-aged outcomes following neonatal care have been an overlooked research priority. Neonatal studies typically focus on two-year composite outcomes which have less meaning for parents, may mask the true neurodevelopmental burden of injuries, and are known to be poorly predictive of future functioning.(7-10) As such, our understanding of childhood developmental trajectories after brain injuries – and whether any sequelae are fixed, stable or amenable to interventions – is limited. We therefore undertook a systematic review to explore the school-age neurodevelopmental outcomes of children following perinatal brain injury.

Confidential: For Review Only

METHODS

Study selection

The review was conducted as per the pre-registered protocol (CRD 42021278572) and the PRISMA statement.(11) We included observational comparative studies exploring neurodevelopmental outcomes of children over five years of age after perinatal brain injury, published between 2000-2021 (Table 1). For inclusion, studies were required to have a non-brain injured comparator group. The primary outcome of interest was neurodevelopmental impairment as defined by study authors; secondary outcomes included motor, cognitive, speech and language, behavioural and neuropsychological, visual and hearing outcomes and seizures.

A search strategy incorporating 99 key terms and mesh headings was developed in Medline Ovid, adapted and run across 10 databases to identify published and grey literature. Snowballing techniques were used to augment search sensitivity (Supplement 1 & 2). All titles were screened independently by two reviewers. The full-texts of all potentially relevant titles were retrieved, reviewed and their risk of bias assessed by two trained reviewers independently (PR, CC, MV, JD, SS). Disagreements were arbitrated by a third reviewer.

Data extraction and synthesis

Studies were stratified by brain injury type, sub-stratified by age of outcome assessment and outcome type, and summarised in a narrative synthesis. Where sufficient suitable data were available from contextually and clinically comparable studies, data were pooled in random effects meta-analyses using RevMan 5.4. Continuous data were pooled using the inverse variance method; dichotomous data were pooled using the Mantel-Haenszel method; and analysis data from studies which did not provide raw data were pooled with dichotomous data

Confidential: For Review Only

from other studies using the generic inverse variance method.(12) Where studies provided insufficient comparative data for a particular outcome, the combined incidence figures for that outcome within the brain injured population was calculated across studies using the Fisher exact test for binomial data.(13) Statistical heterogeneity was assessed using the I^2 statistic and substantial heterogeneity (>85%) was explored further in sub-group analyses.

Quality assessment

The Newcastle Ottawa Tool was used to assess risk of bias across three domains: population selection, the comparability of the 'brain injured' and 'non brain injured' comparator groups, and outcome assessment.(14) Studies were classed as poor, fair, or good for each domain and given an overall risk of bias classification.

Patient and Public Involvement

Patients or the public were not involved in the design or conduct of this review. However the review's findings will be used to shape the larger CHERuB study in partnership with our parent advisory panel.

Confidential: For Review Only

RESULTS

Searches identified 14,210 records and 42 studies were included (Supplement 3). Studies focused on intraventricular haemorrhage (n=27), white matter injury (WMI) amongst preterm infants (n=15), perinatal stroke (n=8), neonatal meningitis (n=4), and HIE (n=3); these were not mutually exclusive (Supplement 4). Most studies were undertaken in the USA (n=10), the UK (n=8), the Netherlands (n=5) or Australia (n=4). These were prospective (n=27) or retrospective cohort studies (n=14). Included studies were deemed to be moderate (n=17) or low risk of bias (n=27) (Supplement 5).

Preterm injuries

The 29 studies exploring outcomes after IVH or WMI mostly included infants born <32 weeks' gestation (n=22) after the year 2000 (n=18) (Supplement 4). Most studies confirmed injury on ultrasound or MRI imaging (n=22) these were reviewed by radiologists (n=6), neonatologists (n=3) or both (n=1); 14 studies used the Papile classification; only 2 studies stratified results by laterality.

Nine studies explored neurodevelopmental impairment at 5-14 years of age after preterm brain injury including IVH (n=9) and WMI (n=6).(15-23) Two comparable studies highlighted a considerably increased pooled crude risk of moderate-severe neurodevelopmental impairment after IVH grade 3-4 at 8 years of age OR 3.69 (95%CI: 1.7, 7.98) I^2 = 0% (Figure 1, Supplement 6).(17, 20)

Six studies explored motor outcomes after IVH grade 3-4: they consistently highlighted an increased risk of motor impairment at 5-12 years of age.(20, 23-27) Additionally, two

Confidential: For Review Only

comparable studies reported an 8-fold increased crude risk of cerebral palsy after IVH grade 3-4 OR 8.13 (95%CI: 4.64, 14.22) I^2=0% (Figure 2).

Cognitive outcomes at school-age after preterm brain injuries were reported by 16 studies using 25 different cognitive assessment tools - limiting the potential for meta-analysis (Supplement 4).(15, 16, 20, 21, 23-34) Educational outcomes were reported by 5 studies.(20, 21, 25, 29, 34)

Studies consistently reported lower cognitive scores at school-age following IVH grade 3-4.(15, 20, 21, 24-26, 30, 34) Hollebrandse 2021 reported an increased risk of cognitive impairment at 8 years of age OR 2.68 (95%CI: 1.21, 5.94).(25) Van de Bor 2000 and Hollebrandse 2021 reported that the cognitive impact of IVH grade 3-4 affected educational needs.(21, 25) Van de Bor 2000 reported increased special educational needs at 5, 9 and 14 years: the adjusted risk at 14 years of age was marked, aOR 3.99 (95%CI: 1.36, 11.69).(21) Studies reported no significant differences in language scores after IVH grade 3-4.(20, 21) However, an association with reading OR 3.62 (95%CI: 1.59, 8.24), spelling OR 4.48 (95%CI: 1.8, 11.2), and arithmetic OR 2.79 (95%CI: 1.2, 6.48) impairment was demonstrated.(25) Most studies highlighted cognitive effects after WMI.(16, 29, 32, 34)

Studies exploring behavioural outcomes after IVH 3-4 did not find any associations with attention deficits, conduct issues or autism spectrum disorder (Supplement 6).(15, 24, 35) However, there was conflicting evidence around the mental health effects of WMI.(16, 36)

Confidential: For Review Only

Studies exploring hearing impairment after IVH and/or WMI were small or not comparable. 10 studies explored visual impairment after IVH or WMI, 4 provided meaningful outcome data.(15, 20-22, 26, 27, 32, 33, 37, 38) An increased prevalence of visual impairment after IVH grade 3-4 (45.4% and 90.9%) compared to controls (7.5%) was reported in addition to significantly lower visual motor integration scores.(26)

Perinatal stroke

Eight comparative studies explored school-age outcomes after perinatal stroke, these included 177 children with perinatal stroke (100 left-sided and 54 right-sided – not all studies specified laterality) and 232 comparator children (Supplement 4).(39-46) Infants' gestation age was largely unspecified. Five studies presented a combined incidence of childhood seizures after perinatal stroke of 40.1% (95%CI: 26.8-53.3% I^2=56%) (Supplement 7).(39, 42, 43, 45, 46) The combined incidence of hemiparesis after perinatal stroke was 61% (95%CI: 39.2, 82.9 I^2=88%). There was considerable heterogeneity across studies, and likely detection bias as only symptomatic children would have undergone diagnostic investigations (Supplement 8).(39, 41-44)

Five studies identified a significant combined mean difference in full scale IQ scores at 7-13 years of age after perinatal stroke: -24.2 (95%CI: -30.73, -17.67) I^2=80% (Figure 3).(39, 41, 44-46) There was heterogeneity across studies in terms of assessment timing, assessment tools, and combining those with left and right-sided strokes.

Differences in stroke laterality partially explained the heterogeneity. The combined mean difference in full scale IQ following left-sided strokes was -26.1 (95%CI: -29.1, -22.93)

Confidential: For Review Only

I^2=0%; compared to -26.7 (95%CI: -39.38. -14.02) I^2=76% for right-sided strokes. No significant differences in cognitive outcomes were found by laterality.(39, 41, 44-46) Kolk 2011 reported significantly lower scores across all NEPSY domains other than executive function after perinatal stroke, including attention, visuo-spacial function, memory, and learning.(42)

Two studies presented educational outcomes after perinatal stroke. Although Northam 2018 found that most children with perinatal stroke were in mainstream education (n=28, 93%), they also highlighted that additional educational support was often required (n=12, 40%). This was in keeping with Ballantyne 2008 reporting lower mean scores for reading (p<0.0001), spelling (p=0.001) and arithmetic (p<0.0001) after perinatal stroke compared to controls at 7-8 years of age, persisting on re-assessment at 10-12 years.

Kolk 2011 reported significantly lower scores compared to controls across most NEPSY language domains following perinatal stroke.(42) Significantly lower receptive and expressive language scores were also reported across studies: -20.88 (95%CI: -36.66, -5.11) I^2=88% and -20.25 (95%CI: -34.36, -6.13) I^2=87% respectively (Supplement 9, 10).(39, 44) Statistical heterogeneity may have been as a result of studies combining left and right-sided strokes and the varying age of outcome assessment. Studies highlighted that deficits in receptive language scores present at 7-8 years persisted at 10-12 years but that expressive language scores improved (p=0.012).(39, 40)

Meningitis

Studies consistently reported an increased risk of neurodevelopmental impairment after neonatal meningitis (Supplement 6).(47-49) An increased likelihood of neuromotor disability

Confidential: For Review Only

at 5 years of age (n=45/274, 16%) compared to controls (n=2/1391, 0.1%) was reported (Supplement 4).(47) On re-assessment of the same population at 9-10 years, this increased risk of severe disability persisted (n=12, 10.8% compared to n=0, 0%).(49) An increased risk of any neurodevelopmental impairment at 5 years after neonatal *Group-B Streptococcal* meningitis was also reported in the Netherlands, RR 5.30 (95%CI: 2·57-10·89), and in Denmark, RR 7.80 (95%CI: 4·42-13·77).(48) This increased risk persisted on subsequent assessment: at 11 years of age in the Netherlands, RR 2.99 (95%CI: 1.83, 4.88) and at 15 years of age in Denmark RR, 3.15 (95%CI: 1.82, 5,46).(48)

Hypoxic-ischaemic encephalopathy

Two comparative studies (of the same cohort) explored outcomes of term-born infants with moderate-severe HIE, but without cerebral palsy, at school age (Supplement 4).(50, 51) They highlighted significantly lower full scale IQ scores after HIE (mean difference −13.62 (95%CI: −20.53 to −6.71)).(50) This difference in cognition was also seen for perceptual reasoning, working memory, and processing speed. Children with HIE were also more likely than controls to receive additional classroom support: OR 10 (95%CI: 1.16, 86) although the confidence interval for this risk estimate was wide.(50) Children with HIE (without cerebral palsy) also had significantly lower motor scores (mean difference −2.12 (95%CI: −3.93, −0.30)) and verbal comprehension scores (mean difference −8.8 (95%CI: −14.25, −3.34)).(50) They were also noted to have higher behavioural difficulty scores especially for emotional problems.(50)

Confidential: For Review Only

DISCUSSION

This review brings together the existing evidence on the later childhood outcomes of infants with perinatal brain injury. Although 42 studies were included, small study populations, limited data on injury severity and laterality, and the heterogeneity of outcome measures limited the potential power of results. However, studies did demonstrate a three-fold higher risk of moderate-severe neurodevelopmental impairment at school age following IVH grade 3-4. Studies consistently report cognitive impairment after IVH grade 3-4 but suggest that speech and language is relatively preserved. A higher risk of hemiplegia, cognitive impairment and poorer academic performance after perinatal stroke is reported in addition to poorer receptive and expressive language scores. Studies consistently report a higher risk of persisting neurodevelopmental impairment after neonatal meningitis – however few studies address this question. Few comparative studies explore school-age outcomes after HIE.

This is the first systematic review to focus on school-age outcomes after perinatal brain injury using the DHSC definition.(6) An extensive search strategy was employed alongside a rigorous review process. Most studies were deemed to be of low risk of bias. Due to our strict inclusion criteria (especially requiring a non-brain injured comparator group) many pertinent studies were excluded. Heterogeneity in terms of outcomes assessed, outcome assessment tools, and timing of outcome assessment limited the comparability of studies and the potential for meta-analyses. This review was also limited by the size of available studies and how studies presented data for extraction. Few studies presented adjusted data or explored childhood trajectories after perinatal brain injury.

Previous reviews were limited by a lack of comparable studies, heterogeneity across studies, the inclusion of much older cohorts (from the pre-surfactant era for example) or by including

Confidential: For Review Only

non-comparative studies.(4, 52-54) Whilst this review was also limited by studies' heterogeneity and the quality of available data, new and important findings - for example the risk of neurodevelopmental impairment - at school age after IVH 3-4 were identified. Our finding of a higher risk of cerebral palsy after IVH and motor impairments after preterm brain injuries is echoed by previous studies.(52, 53, 55)

Lynch 2001 highlighted that 60% of infants have neurological sequelae that emerge over time following perinatal stroke. This was in-keeping with our findings of a higher risk of hemiparesis, cognitive impairment, and speech and language impairment at school age.(56) Several large non-comparative population-based studies also mirror these findings.(57-60)

Although previous reviews highlight an increased risk of various neurodevelopmental impairments after neonatal meningitis in early childhood – we are unaware of any focusing on school-age outcomes after neonatal meningitis.(4, 61)

The review's findings of potential on-going impairments across cognitive, speech and language, and behavioural domains - in addition to a need for increased school support – after HIE are mirrored by other studies.(62-66) Shankaran 2012 and Azzopardi 2014 highlight on-going neurodevelopmental sequelae at school age amongst children who received therapeutic hypothermia for moderate-severe HIE.(62, 63, 65) Unfortunately these studies were not powered to explore individual (non-composite) developmental outcomes or school-age outcomes.(63, 66, 67)

Confidential: For Review Only

Implications

Considerable gaps in the evidence are highlighted, particularly around the risk of specific outcomes following different types of injury, the precision around risk estimates, the impact of different factors (such as injury laterality), and the developmental trajectories of these children i.e. whether outcomes are fixed, deteriorate, or improve over time. This information is key to prepare families for the future, inform enhanced developmental surveillance, and enable targeted multidisciplinary support to help affected children to reach their full potential. As such, this review highlights a pressing need for high-quality, comparative studies which use the 'Core Outcomes In Neonatology' to explore long-term outcomes after perinatal brain injury and permit future meta-analyses.(10) Additionally, to meet the DHSC ambition to reduce perinatal brain injury, real-time longitudinal population data, extending beyond the neonatal period to childhood, are necessary as the current definition is limited to 'indicators' of injury from the neonatal period. This could be achieved through linkage of existing population datasets within the UK and would enable monitoring of progress towards the DHSC goal and evaluation of the impact of national Quality Improvement efforts targeting perinatal brain injuries.(68, 69)

CONCLUSION

This review provides an overview of existing evidence that perinatal brain injuries can have a lasting impact throughout childhood. Considerable gaps in the evidence are highlighted and studies' heterogeneity significantly limited the potential for evidence synthesis. Longitudinal population studies are needed to robustly explore childhood trajectories after perinatal brain injury.

Confidential: For Review Only

Funding/support: This review was supported by an NIHR Doctoral Fellowship award (NIHR301457)

Role of funder/sponsor (if any): The NIHR had no role in the design or conduct of the review.

Conflict of interest disclosures (includes financial disclosures): CG is funded by the United Kingdom Medical Research Council (MRC) through a Transition Support Award. In the past 5 years He has received support from Chiesi Pharmaceuticals to attend an educational conference, and has been investigator on received research grants from Medical Research Council, National Institute of Health Research, Canadian Institute of Health Research, Department of Health in England, Mason Medical Research Foundation, Westminster Medical School Research Trust and Chiesi Pharmaceuticals. CB is funded by the United Kingdom National Institute of Health Research (NIHR) Advanced Fellowship Award.

Contributors' statement

Dr Rees conceptualised and designed the review, reviewed and appraised studies, undertook data extraction and synthesis, drafted the initial manuscript, and reviewed and revised the manuscript.

Dr Callan conceptualized and designed the review, designed and oversaw the search strategy, reviewed and appraised studies, undertook data extraction, and reviewed and revised the manuscript.

Dr Chadda reviewed and appraised studies, undertook data extraction, and reviewed and revised the manuscript.

Dr Vaal reviewed and appraised studies, undertook data extraction, and reviewed and revised the manuscript.

Dr Diviney reviewed and appraised studies, undertook data extraction, and reviewed and revised the manuscript.

Dr Sabti reviewed and appraised studies, undertook data extraction, and reviewed and revised the manuscript.

Dr Harnden reviewed and appraised studies, undertook data extraction, and reviewed and revised the manuscript.

Dr Gardiner was the lead statistician for the review, he advised on and oversaw the data analysis, and reviewed and revised the manuscript.

Dr Battersby oversaw and supervised the review and critically revised the manuscript for important intellectual content.

Confidential: For Review Only

Professor Gale oversaw and supervised the review and critically revised the manuscript for important intellectual content.
Professor Sutcliffe oversaw and supervised the review and critically revised the manuscript for important intellectual content.
All authors approve the final manuscript as submitted and agree to be accountable for all aspects of the work.

Additional Contributions: The authors would like to thank Dr Roxanna Short for creating the figures in supplemental file 7 and 8.

Confidential: For Review Only

Figure 1: Crude risk of neurodevelopmental impairment at 8 years of age after IVH grade 3-4

Figure 2: Crude risk of cerebral palsy after IVH grade 3-4

Figure 3: Pooled mean difference in IQ scores at 7-13 years between those with and without perinatal stroke

Confidential: For Review Only

References

1. Lawn JE, Blencowe H, Oza S, You D, Lee AC, Waiswa P, et al. Every Newborn: progress, priorities, and potential beyond survival. Lancet. 2014;384(9938):189-205.
2. Lee AC, Kozuki N, Blencowe H, Vos T, Bahalim A, Darmstadt GL, et al. Intrapartum-related neonatal encephalopathy incidence and impairment at regional and global levels for 2010 with trends from 1990. Pediatric Research. 2013;74(S1):50-72.
3. Liu L, Oza S, Hogan D, Chu Y, Perin J, Zhu J, et al. Global, regional, and national causes of under-5 mortality in 2000–15: an updated systematic analysis with implications for the Sustainable Development Goals. Lancet. 2016;388(10063):3027-35.
4. Mwaniki MK, Atieno M, Lawn JE, Newton CR. Long-term neurodevelopmental outcomes after intrauterine and neonatal insults: a systematic review. Lancet. 2012;379(9814):445-52.
5. Department of Health & Social Care. New ambition to halve rate of stillbirths and infant deaths 2015 [Available from: https://www.gov.uk/government/news/new-ambition-to-halve-rate-of-stillbirths-and-infant-deaths.
6. Gale C, Statnikov Y, Jawad S, Uthaya SN, Modi N, Brain Injuries expert working group. Neonatal brain injuries in England: population-based incidence derived from routinely recorded clinical data held in the National Neonatal Research Database. Archives of Disease in Childhood - Fetal and Neonatal Edition. 2018;103(4):F301-F6.
7. Marlow N. Measuring neurodevelopmental outcome in neonatal trials: a continuing and increasing challenge. Archives of Disease in Childhood-Fetal and Neonatal Edition. 2013;98(6):F554-F8.
8. Marlow N, Wolke D, Bracewell MA, Samara M. Neurologic and developmental disability at six years of age after extremely preterm birth. New England Journal of Medicine. 2005;352(1):9-19.
9. Webbe J, Brunton G, Ali S, Longford N, Modi N, Gale C. Parent, patient and clinician perceptions of outcomes during and following neonatal care: a systematic review of qualitative research. BMJ Paediatrics Open. 2018;2(1).
10. Webbe JWH, Duffy JMN, Afonso E, Al-Muzaffar I, Brunton G, Greenough A, et al. Core outcomes in neonatology: development of a core outcome set for neonatal research. Archives of Disease in Childhood-Fetal and Neonatal Edition. 2019.
11. Page MJ, McKenzie JE, Bossuyt PM, Boutron I, Hoffmann TC, Mulrow CD, et al. The PRISMA 2020 statement: an updated guideline for reporting systematic reviews. Systematic Reviews. 2021;10(1):1-11.
12. Higgins JPT, Thomas J, Chandler J, Cumpston M, Li T, Page MJ, et al. Cochrane Handbook for Systematic Reviews of Interventions version 6.2 (updated February 2021): Cochrane; 2021. Available from: www.training.cochrane.org/handbook.
13. Mehanna H, Al-Maqbili T, Carter B, Martin E, Campain N, Watkinson J, et al. Differences in the recurrence and mortality outcomes rates of incidental and nonincidental papillary thyroid microcarcinoma: a systematic review and meta-analysis of 21 329 person-years of follow-up. Journal of Clinical Endocrinology and Metabolism. 2014;99(8):2834-43.
14. Wells GA, Shea B, O'Connell D, Peterson J, Welch V, Losos M, et al. The Newcastle-Ottawa Scale (NOS) for assessing the quality of nonrandomised studies in meta-analyses. Oxford; 2000.
15. Adant I, Miserez M, Naulaers G, Carkeek K, Ortibus E, Aerts R, et al. Long-term outcomes of very low birth weight infants with spontaneous intestinal perforation: A retrospective case-matched cohort study. Journal of Pediatric Surgery. 2019;54(10):2084-91.

Confidential: For Review Only

16. Campbell H, Check J, Kuban KC, Leviton A, Joseph RM, Frazier JA, et al. Neonatal cranial ultrasound findings among infants born extremely preterm: associations with neurodevelopmental outcomes at ten years of age. The Journal of Pediatrics. 2021.
17. Cheong JL, Lee KJ, Boland RA, Spittle AJ, Opie GF, Burnett AC, et al. Changes in long-term prognosis with increasing postnatal survival and the occurrence of postnatal morbidities in extremely preterm infants offered intensive care: a prospective observational study. The Lancet Child & Adolescent Health. 2018;2(12):872-9.
18. Neubauer A-P, Voss W, Kattner E. Outcome of extremely low birth weight survivors at school age: the influence of perinatal parameters on neurodevelopment. European Journal of Pediatrics. 2008;167(1):87-95.
19. Piris Borregas S, Torres Valdivieso MJ, Martín-Arriscado C, de la Cruz Bértolo J, Sierra García P, Pallás Alonso CR. Model that predicted death or disabilities in premature infants was valid at seven years of age. Acta Paediatrica. 2019;108(7):1245-9.
20. Sherlock RL, Anderson PJ, Doyle LW, Group VICS. Neurodevelopmental sequelae of intraventricular haemorrhage at 8 years of age in a regional cohort of ELBW/very preterm infants. Early Human Development. 2005;81(11):909-16.
21. Van de Bor M, den Ouden L, editors. School performance in adolescents with and without periventricular-intraventricular hemorrhage in the neonatal period. Seminars in Perinatology; 2004: Elsevier.
22. Vollmer B, Roth S, Baudin J, Stewart AL, Neville BG, Wyatt JS. Predictors of long-term outcome in very preterm infants: gestational age versus neonatal cranial ultrasound. Pediatrics. 2003;112(5):1108-14.
23. Hintz SR, Vohr BR, Bann CM, Taylor HG, Das A, Gustafson KE, et al. Preterm neuroimaging and school-age cognitive outcomes. Pediatrics. 2018;142(1).
24. Brouwer A, Van Stam C, Venema MU, Koopman C, Groenendaal F, De Vries L. Cognitive and neurological outcome at the age of 5–8 years of preterm infants with post-hemorrhagic ventricular dilatation requiring neurosurgical intervention. Neonatology. 2012;101(3):210-6.
25. Hollebrandse NL, Spittle AJ, Burnett AC, Anderson PJ, Roberts G, Doyle LW, et al. School-age outcomes following intraventricular haemorrhage in infants born extremely preterm. Archives of Disease in Childhood-Fetal and Neonatal Edition. 2021;106(1):4-8.
26. Klebermass-Schrehof K, Czaba C, Olischar M, Fuiko R, Waldhoer T, Rona Z, et al. Impact of low-grade intraventricular hemorrhage on long-term neurodevelopmental outcome in preterm infants. Child's Nervous System. 2012;28(12):2085-92.
27. Vollmer B, Roth S, Riley K, O'Brien F, Baudin J, De Haan M, et al. Long-term neurodevelopmental outcome of preterm children with unilateral cerebral lesions diagnosed by neonatal ultrasound. Early Human Development. 2006;82(10):655-61.
28. Hirvonen M, Ojala R, Korhonen P, Haataja P, Eriksson K, Rantanen K, et al. Intellectual disability in children aged less than seven years born moderately and late preterm compared with very preterm and term-born children–a nationwide birth cohort study. Journal of Intellectual Disability Research. 2017;61(11):1034-54.
29. Jansen L, Peeters-Scholte C, Wiggers-de Bruine S, van den Berg-Huysmans A, van Klink J, van Steenis A, et al. Classroom-evaluated school performance at nine years of age after very preterm birth. Early Human Development. 2020;140:104834.
30. Koç Ö, Kavuncuoğlu S, Ramoğlu MG, Aldemir E, Aktalay A, Eras Z. School performance and neurodevelopment of very low birth weight preterm infants: first report from Turkey. Journal of Child Neurology. 2016;31(2):170-6.
31. Pittet M-P, Mürner-Lavanchy I, Adams M, Bickle-Graz M, Pfister R, Natalucci G, et al. Neurodevelopmental outcome at early school age in a Swiss national cohort of very preterm children. Swiss Medical Weekly. 2019;149:w20084.

Confidential: For Review Only

32. Van den Hout B, Stiers P, Haers M, van der Schouw YT, Eken P, Vandenbussche E, et al. Relation between visual perceptual impairment and neonatal ultrasound diagnosis of haemorrhagic–ischaemic brain lesions in 5-year-old children. Developmental Medicine and Child Neurology. 2000;42(6):376-86.
33. Vollmer B, Roth S, Riley K, Sellwood MW, Baudin J, Neville BG, et al. Neurodevelopmental outcome of preterm infants with ventricular dilatation with and without associated haemorrhage. Developmental Medicine and Child Neurology. 2006;48(5):348-52.
34. Kiechl-Kohlendorfer U, Ralser E, Pupp Peglow U, Pehboeck-Walser N, Fussenegger B. Early risk predictors for impaired numerical skills in 5-year-old children born before 32 weeks of gestation. Acta Paediatrica. 2013;102(1):66-71.
35. Davidovitch M, Kuint J, Lerner-Geva L, Zaslavsky-Paltiel I, Rotem RS, Chodick G, et al. Postnatal steroid therapy is associated with autism spectrum disorder in children and adolescents of very low birth weight infants. Pediatric Research. 2020;87(6):1045-51.
36. Whitaker AH, Feldman JF, Lorenz JM, McNicholas F, Fisher PW, Shen S, et al. Neonatal head ultrasound abnormalities in preterm infants and adolescent psychiatric disorders. Archives of General Psychiatry. 2011;68(7):742-52.
37. Hreinsdottir J, Fredriksson Kaul Y, Hellström-Westas L, Rosander K, von Hofsten C, Holmström G. Impaired cognitive ability at 2.5 years predicts later visual and ophthalmological problems in children born very preterm. Acta Paediatrica. 2018;107(5):822-30.
38. Kaur A, Luu TM, Shah PS, Ayoub A, Auger N. Neonatal intraventricular hemorrhage and hospitalization in childhood. Pediatric Neurology. 2020;103:35-42.
39. Ballantyne AO, Spilkin AM, Hesselink J, Trauner DA. Plasticity in the developing brain: intellectual, language and academic functions in children with ischaemic perinatal stroke. Brain. 2008;131(11):2975-85.
40. Ballantyne AO, Spilkin AM, Trauner DA. Language outcome after perinatal stroke: does side matter? Child Neuropsychology. 2007;13(6):494-509.
41. Gold JJ, Trauner DA. Hippocampal volume and memory performance in children with perinatal stroke. Pediatric Neurology. 2014;50(1):18-25.
42. Kolk A, Ennok M, Laugesaar R, Kaldoja M-L, Talvik T. Long-term cognitive outcomes after pediatric stroke. Pediatric Neurology. 2011;44(2):101-9.
43. Martin K, Trauner DA. Auditory neglect in children following perinatal stroke. Behavioural Brain Research. 2019;359:878-85.
44. Northam GB, Adler S, Eschmann KC, Chong WK, Cowan FM, Baldeweg T. Developmental conduction aphasia after neonatal stroke. Annals of Neurology. 2018;83(4):664-75.
45. Tillema J-M, Byars AW, Jacola LM, Schapiro MB, Schmithorst VJ, Szaflarski JP, et al. Reprint of "Cortical reorganization of language functioning following perinatal left MCA stroke"[Brain and Language 105 (2008) 99–111]. Brain and Language. 2008;106(3):184-94.
46. Trauner DA, Nass R, Ballantyne A. Behavioural profiles of children and adolescents after pre-or perinatal unilateral brain damage. Brain. 2001;124(5):995-1002.
47. Bedford H, de Louvois J, Halket S, Peckham C, Hurley R, Harvey D. Meningitis in infancy in England and Wales: follow up at age 5 years. BMJ. 2001;323(7312):533.
48. Horváth-Puhó E, van Kassel MN, Gonçalves BP, de Gier B, Procter SR, Paul P, et al. Mortality, neurodevelopmental impairments, and economic outcomes after invasive group B streptococcal disease in early infancy in Denmark and the Netherlands: a national matched cohort study. The Lancet Child & Adolescent Health. 2021;5(6):398-407.
49. Stevens J, Eames M, Kent A, Halket S, Holt D, Harvey D. Long term outcome of neonatal meningitis. Archives of Disease in Childhood-Fetal and Neonatal Edition. 2003;88(3):F179-F84.

Confidential: For Review Only

50. Lee-Kelland R, Jary S, Tonks J, Cowan FM, Thoresen M, Chakkarapani E. School-age outcomes of children without cerebral palsy cooled for neonatal hypoxic–ischaemic encephalopathy in 2008–2010. Archives of Disease in Childhood-Fetal and Neonatal Edition. 2020;105(1):8-13.
51. Tonks J, Cloke G, Lee-Kelland R, Jary S, Thoresen M, Cowan FM, et al. Attention and visuo-spatial function in children without cerebral palsy who were cooled for neonatal encephalopathy: a case-control study. Brain Injury. 2019;33(7):894-8.
52. Gotardo JW, Volkmer NdFV, Stangler GP, Dornelles AD, Bohrer BBdA, Carvalho CG. Impact of peri-intraventricular haemorrhage and periventricular leukomalacia in the neurodevelopment of preterms: A systematic review and meta-analysis. PloS one. 2019;14(10):e0223427.
53. Mukerji A, Shah V, Shah PS. Periventricular/intraventricular hemorrhage and neurodevelopmental outcomes: a meta-analysis. Pediatrics. 2015;136(6):1132-43.
54. Magai DN, Karyotaki E, Mutua AM, Chongwo E, Nasambu C, Ssewanyana D, et al. Long-term outcomes of survivors of neonatal insults: A systematic review and meta-analysis. PloS one. 2020;15(4):e0231947.
55. Rees P, Callan C, Chadda KR, Vaal MMBChB, Diviney J, Sabti S, et al. Preterm brain injury and neurodevelopmental outcomes: a meta-analysis. Pediatrics. in-press.
56. Lynch JK, Nelson KB. Epidemiology of perinatal stroke. Current Opinion in Pediatrics. 2001;13(6):499-505.
57. Lee J, Croen LA, Lindan C, Nash KB, Yoshida CK, Ferriero DM, et al. Predictors of outcome in perinatal arterial stroke: a population-based study. Annals of Neurology. 2005;58(2):303-8.
58. Grunt S, Mazenauer L, Buerki SE, Boltshauser E, Mori AC, Datta AN, et al. Incidence and outcomes of symptomatic neonatal arterial ischemic stroke. Pediatrics. 2015;135(5):e1220-e8.
59. Husson B, Hertz-Pannier L, Renaud C, Allard D, Presles E, Landrieu P, et al. Motor outcomes after neonatal arterial ischemic stroke related to early MRI data in a prospective study. Pediatrics. 2010;126(4):e912-e8.
60. Wusthoff CJ, Kessler SK, Vossough A, Ichord R, Zelonis S, Halperin A, et al. Risk of later seizure after perinatal arterial ischemic stroke: a prospective cohort study. Pediatrics. 2011;127(6):e1550-e7.
61. Kohli-Lynch M, Russell NJ, Seale AC, Dangor Z, Tann CJ, Baker CJ, et al. Neurodevelopmental impairment in children after group B streptococcal disease worldwide: systematic review and meta-analyses. Clinical Infectious Diseases. 2017;65(suppl_2):S190-S9.
62. Shankaran S, Pappas A, McDonald SA, Vohr BR, Hintz SR, Yolton K, et al. Childhood outcomes after hypothermia for neonatal encephalopathy. New England Journal of Medicine. 2012;366(22):2085-92.
63. Azzopardi D, Strohm B, Marlow N, Brocklehurst P, Deierl A, Eddama O, et al. Effects of hypothermia for perinatal asphyxia on childhood outcomes. New England Journal of Medicine. 2014;371(2):140-9.
64. Jary S, Lee-Kelland R, Tonks J, Cowan FM, Thoresen M, Chakkarapani E. Motor performance and cognitive correlates in children cooled for neonatal encephalopathy without cerebral palsy at school age. Acta Paediatrica. 2019;108(10):1773-80.
65. Natarajan G, Shankaran S, Pappas A, Bann C, Tyson JE, McDonald S, et al. Functional status at 18 months of age as a predictor of childhood disability after neonatal hypoxic-ischemic encephalopathy. Developmental Medicine & Child Neurology. 2014;56(11):1052-8.

Confidential: For Review Only

66. Guillet R, Edwards AD, Thoresen M, Ferriero DM, Gluckman PD, Whitelaw A, et al. Seven-to eight-year follow-up of the CoolCap trial of head cooling for neonatal encephalopathy. Pediatric Research. 2012;71(2):205-9.
67. Marlow N, Shankaran S, Rogers EE, Maitre NL, Smyser CD, Newborn Brain Society G, et al. Neurological and developmental outcomes following neonatal encephalopathy treated with therapeutic hypothermia. Seminars in Fetal and Neonatal Medicine. 2021;26(5):101274.
68. THIS.Institute. Avoiding Brain Injury in Childbirth (ABC) collaboration 2022 [Available from: https://www.thisinstitute.cam.ac.uk/research-projects/avoiding-brain-injury-in-childbirth-collaboration/#:~:text=The%20Avoiding%20Brain%20Injury%20in,to%20suspected%20intrapartum%20fetal%20deterioration.
69. NHS England. Maternity and Neonatal Safety Improvement Programme [Available from: https://www.england.nhs.uk/mat-transformation/maternal-and-neonatal-safety-collaborative/.

Confidential: For Review Only

Table 1 Inclusion and exclusion criteria

Inclusion Criteria	Exclusion Criteria
Peer-reviewed observational studies (cohort, case-control, cross-sectional)	Non-comparative studies; opinions; commentaries; reviews; case-reports; lab studies
Studies in all languages	Studies where the population includes adults and children and the data for children cannot be extracted
Studies published after 2000	Studies focused on children with IVH grade 1-2, neonatal seizures, hypoglycaemic brain injury, or neonatal abstinence syndrome
Children with a diagnosis of brain injury occurring at or around the time of birth (including during the neonatal period) as defined by the DHSC (including those with any white matter injury but not including those with isolated seizures)	Studies which include infants with brain injuries diagnosed during the neonatal and infancy period where most were diagnosed outside of the neonatal period
Studies including infants with moderate to severe HIE born in the post therapeutic hypothermia era (i.e. where infants received therapeutic hypothermia)	Studies including infants with moderate-severe HIE born during the pre-therapeutic hypothermia era or in low- or middle-income countries that do not offer therapeutic hypothermia
Studies focused on school-aged neurodevelopmental outcomes (of children between 5-18 years of age) including:<br>**Primary outcome(s):**<br>Neurodevelopmental impairment, as defined by authors (including direct testing, clinical record review, and parental interview/ survey)<br><br>**Secondary outcome(s):**<br>1. Any cognitive impairment, as defined by authors (direct testing)<br><br>2. Mild cognitive impairment (intelligence or developmental quotient 1-2 standard deviations below the mean)<br><br>3. Moderate-severe cognitive impairment (intelligence or developmental quotient more than 2 standard deviations below the mean)<br><br>4 Executive dysfunction, as defined by authors (direct testing)<br><br>5. Low numeracy, as defined by authors (by direct testing or educational achievement tests)<br><br>6. Low literacy, as defined by authors (by direct testing or educational achievement tests)<br><br>7. Special educational needs as defined by authors (school or parental report)<br><br>8. Motor impairment, as defined by authors (including direct testing, clinical record review, and reporting)<br><br>9. Visual-motor impairment, as defined by authors (on direct testing)	Studies of infants with mild HIE

Confidential: For Review Only

10. Emotional-behavioural difficulty, as defined by authors (including direct testing, clinical record review, and parental reporting<br><br>11. Speech and language impairment, as defined by authors (on direct testing)<br><br>12. Visual impairment, as defined by authors (including direct testing, clinical record review, and parental reporting)<br><br>13. Hearing impairment, as defined by authors (including direct testing, clinical record review, and parental reporting)<br><br>14. Epilepsy/seizures, as defined by authors (including medical history taking, clinical record review and parental reporting	
	Studies reporting outcomes for children diagnosed with brain injury beyond the neonatal period
	Studies where comparable outcome data from those with and without perinatal brain injury cannot be extracted

Confidential: For Review Only

Study or Subgroup	log[Odds Ratio]	SE	Weight	Odds Ratio IV, Random, 95% CI
Cheong 2018	1.38879124	0.59424024	43.9%	4.01 [1.25, 12.85]
Sherlock 2005	1.23969089	0.52602402	56.1%	3.45 [1.23, 9.69]
**Total (95% CI)**			**100.0%**	**3.69 [1.70, 7.98]**

Heterogeneity: Tau² = 0.00; Chi² = 0.04, df = 1 (P = 0.85); I² = 0%
Test for overall effect: Z = 3.31 (P = 0.0009)

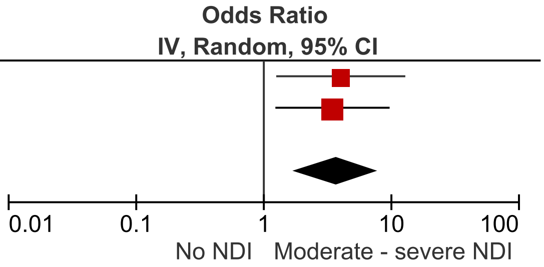

Confidential: For Review Only

Confidential: For Review Only

Study or Subgroup	IVH grade 3-4 Events	IVH grade 3-4 Total	No IVH Events	No IVH Total	Weight	Odds Ratio M-H, Random, 95% CI
Beaino 2010	9	38	46	1153	48.5%	7.47 [3.34, 16.69]
Hollebrandse 2021	15	35	26	331	51.5%	8.80 [4.03, 19.19]
**Total (95% CI)**		**73**		**1484**	**100.0%**	**8.13 [4.64, 14.22]**
Total events	24		72			

Heterogeneity: Tau² = 0.00; Chi² = 0.08, df = 1 (P = 0.77); I² = 0%
Test for overall effect: Z = 7.33 (P < 0.00001)

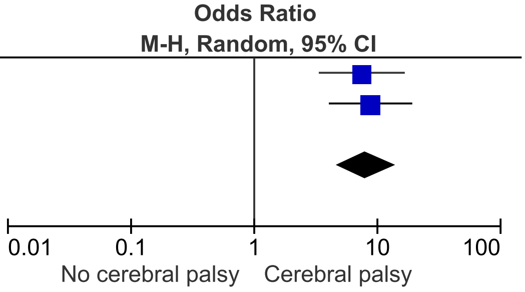

Study or Subgroup	Perinatal stroke Mean	SD	Total	No stroke Mean	SD	Total	Weight	Mean Difference IV, Random, 95% CI
Ballayntyne 2008	94.7	20.4	29	123	15	38	18.2%	-28.30 [-37.12, -19.48]
Gold 2014	88	4	27	117	2.7	19	26.6%	-29.00 [-30.94, -27.06]
Northam 2017	99	14	30	112	16	40	20.7%	-13.00 [-20.05, -5.95]
Tilema 2008	80	14.1	10	108	11.7	10	14.9%	-28.00 [-39.36, -16.64]
Trauner 2001	93.4	22	39	116.2	13	54	19.7%	-22.80 [-30.53, -15.07]
**Total (95% CI)**			**135**			**161**	**100.0%**	**-24.20 [-30.73, -17.67]**

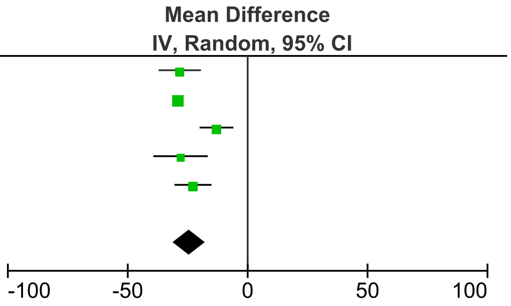

Heterogeneity: Tau² = 40.85; Chi² = 20.00, df = 4 (P = 0.0005); I² = 80%
Test for overall effect: Z = 7.26 (P < 0.00001)

Confidential: For Review Only

**Supplement 1: databases searched**
Cochrane Central Register of Controlled Trials
EBSCO–CINAHL (Cumulative Index to Nursing and Allied Health Literature)
Google Scholar
Ovid–EMBASE
Ovid–MEDLINE
Ovid–MEDLINE E-pub ahead of print
Ovid–MEDLINE In-Process and Other Non-Indexed Citations
PubMed
Scopus
Web of Knowledge (Science Citation Index Expanded and Conference Proceedings Citation Index Science)

Confidential: For Review Only

Supplement 2: Medline Ovid Search Strategy

1. exp CHILD/
2. exp Child, Preschool/
3. exp ADOLESCENT/
4. exp INFANT/ or exp INFANT, NEWBORN/
5. (child* or toddler* or baby or infant* or adolescent*).mp.
6. 1 or 2 or 3 or 4 or 5
7. exp Educational Status/
8. exp Child Development/
9. exp Learning Disorders/
10. exp Educational Measurement/
11. exp SCHOOLS/
12. exp Academic Performance/
13. school performance.mp.
14. exp COGNITION/
15. exp LEARNING/
16. exp SPATIAL LEARNING/
17. exp VERBAL LEARNING/
18. exp SOCIAL LEARNING/
19. exp Intelligence Tests/
20. exp INTELLIGENCE/
21. exp Intellectual Disability/
22. exp Neurodevelopmental Disorders/
23. neurodevelopm*.mp.
24. (nervous system dys* or CNS dys*).mp. [mp=title, abstract, heading word, drug trade name, original title, device manufacturer, drug manufacturer, device trade name, keyword, floating subheading word, candidate term word]
25. (nervous system abnorm* or CNS abnorm*).mp. [mp=title, abstract, heading word, drug trade name, original title, device manufacturer, drug manufacturer, device trade name, keyword, floating subheading word, candidate term word]
26. (nervous system malform* or CNS malform*).mp. [mp=title, abstract, heading word, drug trade name, original title, device manufacturer, drug manufacturer, device trade name, keyword, floating subheading word, candidate term word]
27. (nervous system dis* or CNS dis*).mp. [mp=title, abstract, heading word, drug trade name, original title, device manufacturer, drug manufacturer, device trade name, keyword, floating subheading word, candidate term word]
28. (mental health condi* or mental health dis*).mp. [mp=title, abstract, heading word, drug trade name, original title, device manufacturer, drug manufacturer, device trade name, keyword, floating subheading word, candidate term word]
29. mental health outcome.mp.
30. behaviour* abnorm*.mp.
31. cognitive impairment.mp. or exp Cognitive Dysfunction/
32. visual impairment.mp. or exp Vision Disorders/
33. visual develop*.mp.
34. (visual dis* or visual dys*).mp. [mp=title, abstract, heading word, drug trade name, original title, device manufacturer, drug manufacturer, device trade name, keyword, floating subheading word, candidate term word]

Confidential: For Review Only

35. (nystagmus or strabismus).mp.
36. (visual acuity or refractive error*).mp.
37. hearing impairment.mp. or exp Hearing Loss/
38. exp Deafness/
39. exp DEAF-BLIND DISORDERS/
40. exp Hearing Loss, Sensorineural/
41. exp Movement Disorders/
42. exp Cerebral Palsy/
43. motor impairment.mp.
44. (seizure* or convulsi*).mp.
45. exp EPILEPSY/ or epilepsy.mp.
46. exp Executive Function/
47. visual-motor impairment.mp.
48. numeracy.mp.
49. literacy.mp. or exp LITERACY/
50. jaundice.mp.
51. exp Language Development Disorders/ or exp Child Language/ or language impairment.mp. or exp Reading/ or exp Dyslexia/ or reading impairment.mp.
52. 7 or 8 or 9 or 10 or 11 or 12 or 13 or 14 or 15 or 16 or 17 or 18 or 19 or 20 or 21 or 22 or 23 or 24 or 25 or 26 or 27 or 28 or 29 or 30 or 31 or 32 or 33 or 34 or 35 or 36 or 37 or 38 or 39 or 40 or 41 or 42 or 43 or 44 or 45 or 46 or 47 or 48
53. 49 or 50 or 51
54. 52 or 53
55. exp JAUNDICE, NEONATAL/
56. exp JAUNDICE/
57. exp Hyperbilirubinemia, Neonatal/
58. exp Hyperbilirubinemia/
59. hyperbilirubin*.mp.
60. exp Hyperbilirubinemia, Hereditary/
61. bilirubin encephalopathy.mp.
62. bilirubin-induced neuro*.mp.
63. exchange transfusion.mp.
64. exp ASPHYXIA NEONATORUM/
65. (exp ASPHYXIA/ or asphyxia.mp.) and neonat*.mp.
66. exp Hypoxia-Ischemia, Brain/ and neonat*.mp.
67. perinatal asphyxia.mp.
68. birth asphyxia.mp.
69. (hypoxic-ischemic encephalopathy or hypoxic-ischaemic encephalopathy).mp.
70. neonatal encephalopathy.mp.
71. (exp Cerebral Hemorrhage/ or exp Intracranial Hemorrhages/ or exp Brain Ischemia/ or intracranial haemorrhage.mp. or exp Subarachnoid Hemorrhage/ or exp Stroke/) and neonat*.mp.
72. perinatal stroke.mp.
73. (central nervous system infection.mp. or exp Central Nervous System Infections/) and neonat*.mp.
74. (exp Meningoencephalitis/ or meningo-encephalitis.mp.) and neonat*.mp.
75. (MENINGITIS/ or meningitis.mp.) and neonat*.mp.

Confidential: For Review Only

76. exp MENINGITIS, VIRAL/ and neonat*.mp.
77. (meningoencephalitis and neonat*).mp.
78. (encephalitis.mp. or exp ENCEPHALITIS, VIRAL/ or exp INFECTIOUS ENCEPHALITIS/ or exp ENCEPHALITIS/) and neonat*.mp.
79. kernicterus.mp. or exp KERNICTERUS/
80. preterm white matter disease.mp.
81. (periventricular leukomalacia.mp. or exp Leukomalacia, Periventricular/) and neonat*.mp.
82. (therapeutic hypothermia.mp. or exp Hypothermia, Induced/) and neonat*.mp.
83. ((subdural haemorrhage or subdural hemorrhage) and neonat*).mp.
84. (exp Hematoma, Subdural/ or subdural haemorrhage.mp. or exp Craniocerebral Trauma/) and neonat*.mp.
85. (intraventricular haemorrhage and neonat*).mp.
86. (tentorial tear and neonat*).mp.
87. (parenchymal haemorrhage and neonat*).mp.
88. (ventriculoperitoneal shunt.mp. or exp Cerebrospinal Fluid Shunts/ or exp Ventriculoperitoneal Shunt/) and neonat*.mp.
89. ((ventricular drain or Rickham reservoir or CSF shunt) and neonat*).mp.
90. neonatal stroke.mp.
91. (cerebrovascular accident and neonat*).mp.
92. neonatal cerebral ischaemia.mp.
93. (exp Intracranial Thrombosis/ or cerebral venous thrombosis.mp.) and neonat*.mp.
94. (seizure.mp. or exp Seizures/) and neonat*.mp.
95. 55 or 56 or 57 or 58 or 59 or 60 or 61 or 62 or 63 or 64 or 65 or 66 or 67 or 68 or 69 or 70 or 71 or 72 or 73 or 74 or 75 or 76 or 77 or 78 or 79 or 80 or 81 or 82 or 83 or 84 or 85 or 86 or 87 or 88 or 89 or 90 or 91 or 92 or 93 or 94
96. exp Cohort Studies/
97. exp Retrospective Studies/
98. (cohort* or (case$ and control$)).tw.
99. exp Cross-Sectional Studies/
100. exp Randomized Controlled Trial/
101. 96 or 97 or 98 or 99 or 100
102. exp "REVIEW"/
103. exp Case Reports/
104. Animals/
105. animal stud*.mp.
106. 102 or 103 or 104 or 105
107. 6 and 52 and 95 and 101
108. 107 not 106

Confidential: For Review Only

PRISMA 2009 Flow Diagram

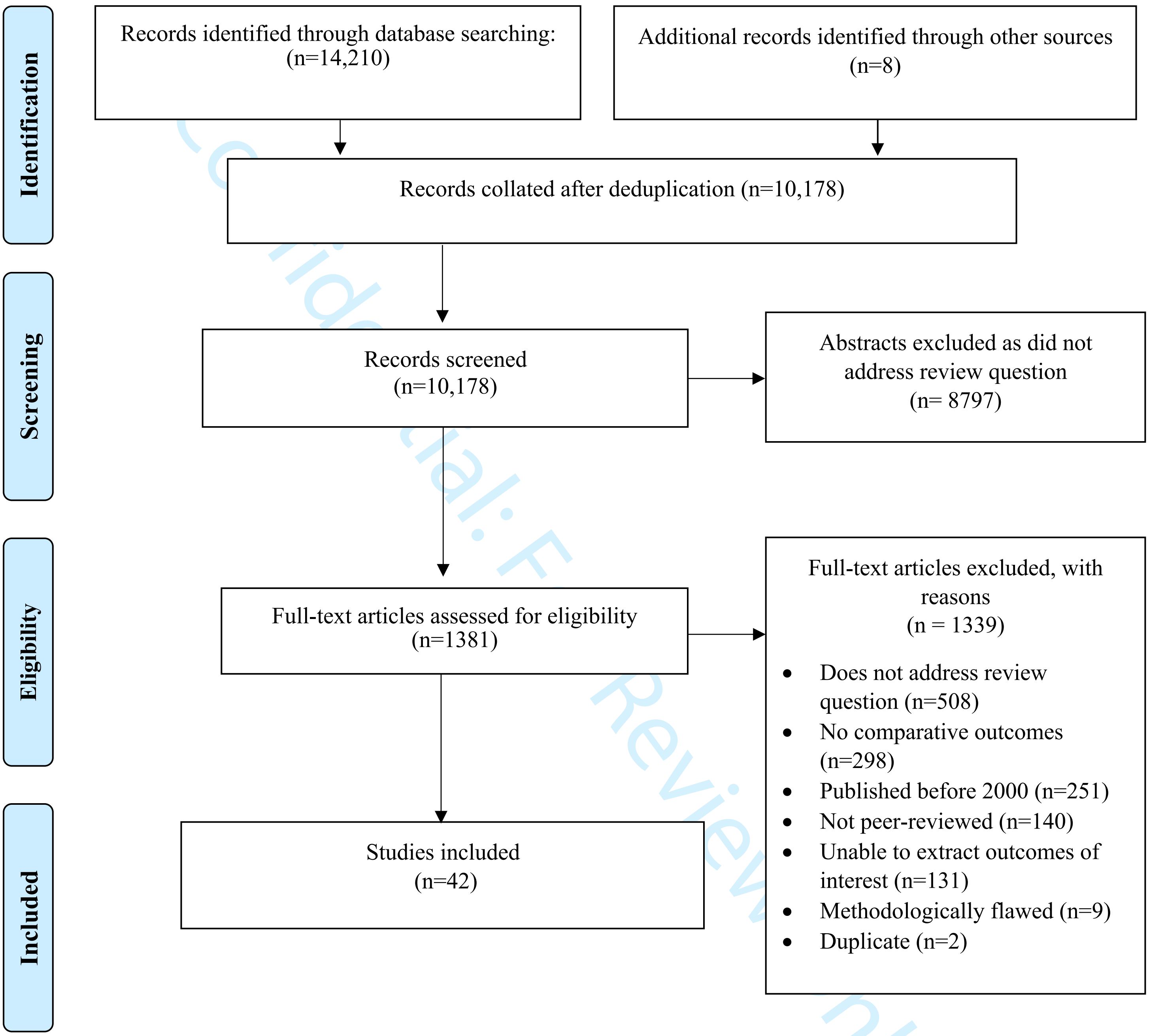

Confidential: For Review Only

Confidential: For Review Only

Supplement 4: included studies of school-aged outcomes after perinatal brain injury

* overlapping study data; Ω potential error in manuscript; Adjusted Odds Ratio (aOR); Attention Deficit Hyperactivity Disorder (ADHD); Autism Spectrum Disorder (ASD); Bayley Scale of Infant Development (BSID); Child Behaviour Checklist (CBCL); Clinical Evaluation of Language Fundamentals (CELF); Cystic Periventricular leukomalacia (cPVL); Gross Motor Function Classification System, (GMFCS); Haemorrhagic parenchymal infarction (HPI); Hazard Ratio (HR); International Classification of Disease (ICD); Intraventricular haemorrhage (IVH); Intelligence Quotient (IQ); Kaufman Assessment Battery for Children (K-ABC); Mental Developmental Index (MDI); Peabody Picture Vocabulary Test (PPVT); Periventricular (PV); Periventricular leukomalacia (PVL); National Institute of Child Health and Human Development (NICHD); Neonatal Intensive Care Unit (NICU); Psychomotor Development Index (PDI); Retinopathy of Prematurity (ROP); Small for Gestational Age (SGA); Spontaneous Intestinal Perforation (SIP); Standard Deviation (SD); Standard Error (SE); Test of Motor Impairment (TOMI); Very low birthweight (VLBW); Visuomotor integration (VMI); Wechsler Abbreviated Scale of Intelligence (WASI); Wechsler Intelligence Scale for Children (WISC); Wechsler Preschool & Primary Scale of Intelligence (WPPSI); White Matter Injury (WMI); Wide Range Achievement Test (WRAT)

	Author<br>Year<br>Country<br>Study type	Population<br>Exposures<br>Comparator<br>Ascertainment/ definition	Outcomes	Main result(s)
1	Adant 2019[15]<br><br>Belgium<br><br>Retrospective cohort	**Population**<br>• Gestation ≤32 weeks with and without spontaneous intestinal perforation (SIP)<br>• Born 1994-2014<br><br>**Exposure (n=19)**<br>• IVH grade 3-4<br><br>**Comparator (n=44)**<br>• Matched on gender, gestational age, date of birth (multiples matched to sibling without SIP)<br>• No IVH<br><br>**Ascertainment/ definition**<br>• Clinical record review	**Outcomes**<br>• Functional disability (composite)<br>• Cognitive<br>• Motor<br>• Visual<br>• Behavioural/ mental health<br>• Wellbeing<br>• Quality of life<br>• Physical health<br><br>**Measurement/ assessment**<br>• BSID II<br>• Telephone survey (parents)<br>• PedsQL<br>• IQ testing<br><br>**Follow-up**<br>• 67% follow-up at 7-11 months<br>• 41% follow-up at 18-22 months<br>• 49% follow-up at 4-10 years<br>• 86% follow-up telephone survey	**Outcomes of those with SIP compared to controls without SIP – by IVH subgroup**<br><br>**Disability**<br>aOR 8.79 95%CI (1.72, 44.86)<br><br>**Multiple disabilities**<br>aOR 5.97 95%CI (1.61, 22.15)<br><br>**Cognitive**<br>**Regular education system (not a special educational needs school)**<br>aOR 8.73 95%CI (2.1, 36.72*)*<br><br>**Visual outcomes** (wearing glasses)<br>aOR 0.474 95%CI (0.13, 1.69)<br><br>**Behavioural/ mental health disorder** (including attention problems, conduct problems and autism spectrum disorders)<br>aOR 1.24 95%CI (0.32, 4.8)<br><br>**PedsQL low quality of life score**<br>aOR 0.87 95%CI (0.77, 0.99)<br><br>**PedsQL low physical health score**<br>aOR 0.82 95%CI (0.66, 1.01)
2	Beaino 2010[73]<br><br>France<br><br>Prospective cohort	**Population**<br>• Gestation <33 weeks<br>• Born 1997<br><br>**Exposure**<br>• IVH grade 1 (n=173)<br>• IVH grade 2 (n=117)<br>• IVH grade 3 (n=32)<br>• Intraparenchymal haemorrhage (IPH) (n=6)<br>• Persistent echodensities or ventricular dilatation (n=241)<br>• cPVL (n=66)<br><br>**Comparator (n=1153)**<br>• Unmatched<br>• No IVH<br><br>**Ascertainment/ definition**<br>• Ultrasound imaging undertaken and reviewed by neonatologists or radiographers	**Outcomes**<br>• Cerebral palsy<br><br>**Measurement/assessment**<br>• Standardised questionnaires completed by physicians<br><br>**Follow-up**<br>• 5 years<br>• 77% follow-up	**Cerebral palsy**<br>**Grade 3 IVH**<br>OR 3.75 95%CI (2.41–5.85)<br><br>**Grade 3 IVH or echodensities of ventricular dilatation**<br>Model A aOR 3.25 95%CI (2.02–5.22)<br>Model B aOR 3.40 95%CI (2.07–5.60)<br>Model C aOR 3.31 95%CI (2.00–5.48)<br><br>**cPVL**<br>OR 33.41 95%CI (19.25–57.96)<br><br>**Cystic PVL or IPH**<br>Model A aOR 29.66 95%CI (16.71–52.62)<br>Model B aOR 28.41 95%CI (15.65–51.59)<br>Model C n/a

Confidential: For Review Only

3	Brouwer 2012[24]<br><br>Netherlands<br><br>Prospective cohort	**Population**<br>• Gestation <32 weeks<br>• Born 1999-2004<br><br>**Exposure (n=32)**<br>• Post-haemorrhagic ventricular dilatation after IVH grade 3-4 requiring neurosurgical intervention<br>• No PVL<br><br>**Comparator (n=23)**<br>• Matched on gestation, birthweight, and sex<br>• No IVH<br><br>**Ascertainment/ definition**<br>• Ultrasound diagnosis<br>• Papile classification	**Outcomes**<br>• Motor<br>• Cerebral palsy<br>• Cognitive<br>• Behavioural<br><br>**Measurement/ assessment**<br>• Movement ABC<br>• GMFCS<br>• WPPSI (3rd edition Dutch version)<br>• Revisie Amsterdamse Kinder Intelligentietest<br>• Snijders Oomen Nonverbal Intelligence Test 2.5-7 – Revised<br>• CBCL<br>• Teacher Report Form<br><br>**Follow-up**<br>• 4-8 years (median 5.7)<br>• 97% follow-up	**Cerebral palsy**<br>IVH grade 3 n=0<br>IVH grade 4 n=8, 53%; all unilateral spastic cerebral palsy<br>GMFCS level 1, n=5<br>GMFCS level 2, n=2<br>GMFCS level 3, n=1<br><br>**Movement ABC motor score (for those without cerebral palsy)**<br>**Score <p 5 (definite motor problems)**<br>IVH grade 3 n=6, 26%<br>IVH grade 4 n=3, 13%<br>No IVH n=0<br><br>**Score p 5-15 (borderline motor function)**<br>IVH grade 3 (n=6; 26%)<br>IVH grade 4 (n=0; 0%)<br>No IVH (n=5; 29.4%)<br><br>**Score p> 15**<br>IVH grade 3 n=6, 26%<br>IVH grade 4 n=0, 0%<br>No IVH n=12, 70.6%<br><br>**Cognition**<br>**Wechsler intelligence test (mean ±SD)**<br>**Verbal scale**<br>IVH n=23, 97±13<br>IVH <30weeks' gestation n=16, 94±13<br>No IVH n=24, 96±13;<br><br>**Performance scale**<br>IVH, n=23, 94±16;<br>IVH <30weeks' gestation n=16, 93±15<br>No IVH n=24, 103±14;<br><br>**Production scale**<br>IVH n=23, 87±22;<br>IVH <30weeks' gestation n=16, 85±24<br>No IVH n=24, 93±14<br><br>**Intelligence quotient (n; mean +/-SD)**<br>IVH grade 3 n=17; IQ 96±15;<br>IQ>85 n=13 (76.5%)<br><br>IVH IV n=15; IQ 91±10;<br>IQ >85 n=9 (64.3%)<br><br>IVH <30 weeks' gestation n=23; IQ 92±17;<br>IQ>85 n=15 (65.2%)<br><br>No IVH n=23; IQ 98±15,<br>IQ>85 n=17 (74%)<br><br>**Behavioural outcomes**<br>**CBCL parental score: mean T score ±SD, n in subclinical range (%)**<br>**Total scale**<br>IVH n=26: 48.2 ±8.4, n=3 (12%)<br>IVH <30 weeks' gestation n=20: 46.9 ±8.3, n=2 (10%)<br>No IVH <30 weeks' gestation n=23: 44.3 ±7.8, n=1 (4%)<br><br>**Internalising problem scale**<br>IVH: 49.2 ±8.9, n=5 (19%)<br>IVH <30 weeks' gestation: 28.2 ±8.4, n=3 (15%)<br>No IVH <30 weeks' gestation: 49.2 ±9.1, n=5 (21%)<br><br>**Externalizing problem scale**<br>IVH: 46.8 ±9.4, n=2 (8%)<br>IVH <30 weeks' gestation: 45.1 ±9.5, n=1 (15%)<br>No IVH < 30weeks' gestation: 43.7 ±7.5, n=0 (0%)<br><br>**TRF teachers score: mean T score ±SD, n in subclinical range (%)**<br>**Total scale**<br>IVH n=25: 54.7 ±8.7, n=6 (24%)<br>IVH <30 weeks' gestation n=19: 53.9 ±9.0, n=4 (21%)<br>No IVH <30 weeks' gestation n=22: 50.9 ±9.8, n=4 (18%)<br><br>**Internalising problem scale**<br>IVH: 53.2 ±10.8, 4 (16%)<br>IVH <30 weeks' gestation: 52.2 ±11.7, n=3 (16%)<br>No IVH <30 weeks' gestation: 52.4 ±11.4, n=7 (32%)<br><br>**Externalizing problem scale**<br>IVH: 54.3 ±6.7, 3 (12%)<br>IVH <30 weeks' gestation: 54.1 ±7.0, n=2 (11%)<br>No IVH <30 weeks' gestation: 49.7 ±7.7, n=2 (9%)<br><br>N=13 (41%) had repeated a school class, had educational help and/or attended special education

Confidential: For Review Only

4	Campbell 2021[16]<br><br>USA<br><br>Prospective cohort study	**Population (n=858)**<br>• Gestation 23-27 weeks<br>• Born 2002-2004<br><br>**Exposure**<br>• IVH without WMI (n=124)<br>• WMI without IVH (n=30)<br>• IVH and WMI (n=63)<br><br>**Comparator (n=641)**<br>• Unmatched<br>• No IVH or WMI<br><br>**Ascertainment/ definition**<br>• Ultrasound imaging reviewed by two independent blinded radiologists<br>• WMI: parenchymal echolucency or moderate to severe ventriculomegaly on a late scan	**Outcomes**<br>• Neurocognitive development (composite)<br>• Cognitive<br>• Cerebral palsy<br>• Behavioural/ mental health<br>• Epilepsy<br>• Quality of life<br><br>**Measurement/ assessment**<br>• Differential Ability Scale II<br>• NEPSY II<br>• Neurological exam<br>• GMFCS<br>• Parental questionnaire<br>• Social Communication Questionnaire<br>• Child Symptom Inventory 4<br>• Peds QoL 4<br><br>**Follow up**<br>• 10 years<br>• 74% follow-up	**Neurodevelopmental burden**<br>**No impairments**<br>IVH and WMI n=24, 38%<br>WMI n=12, 40%<br>IVH n= 86, 69%<br>No IVH or WMI n=487, 76%<br><br>**No cognitive impairment; 1 or more of cerebral palsy, ASD, or epilepsy**<br>IVH and WMI n=4, 6%<br>WMI n=4, 13%<br>IVH n=7, 6%<br>No IVH or WMI n=26, 4%<br><br>**Cognitive**<br>**Normal cognitive function**<br>IVH and WMI n=8, 13%<br>WMI n=5, 17%<br>IVH n=41, 33%<br>No IVH or WMI n=235, 37%<br><br>**Cognitive impairment (moderate to severe)**<br>**IVH and WMI**<br>n=35, 56%<br>OR 5.01 95% CI (2.94, 8.54)<br>aOR 4.49 95% CI (2.49, 8.11)<br><br>**WMI**<br>n=14, 47%<br>OR 3.51 95% CI (1.67, 7.37)<br>aOR 5.07 95% CI (2.13, 12.02)<br><br>**IVH**<br>n=31, 25%<br>OR 1.34 95% CI (0.85, 2.1)<br>aOR 1.21 95% CI (0.73, 1.98)<br><br>**No IVH or WMI**<br>n=128. 20%<br>Reference category<br><br>**Low cognitive function**<br>IVH and WMI n=18. 30%<br>WMI n=10, 34%<br>IVH n=50, 41%<br>No IVH or WMI n=269, 43%<br><br>**Moderate cognitive impairment**<br>IVH and WMI n=17, 28%<br>WMI n=7, 24%<br>IVH n=24, 20%<br>No IVH or WMI n=93, 15%<br><br>**Severe cognitive impairment**<br>IVH and WMI n=18, 30%<br>WMI n=7, 24%<br>IVH n=7, 6%<br>No IVH or WMI n=35, 6%<br><br>**Nonverbal IQ**<br>**IVH vs. No IVH or WMI**<br>Crude mean difference -3 95%CI (-6.6, 0.6)<br><br>**Full scale IQ**<br>**IVH vs No IVH or WMI**<br>Crude mean difference -2.2 95%CI (-5.7, 1.4)<br><br>**Cerebral palsy**<br>**IVH and WMI**<br>n=32, 51%<br>OR 16.85 95% CI (9.29, 30.55)<br>aOR 13.43 95% CI (7, 25.78)<br><br>**WMI**<br>n=14, 47%<br>OR 14.28 95% CI (6.48, 41.48)<br>aOR 18.63 95% CI (7.37, 47.06)<br><br>**IVH**<br>n=9, 7%<br>OR 1.28 95% CI (0.6, 2.72)<br>aOR 1.19 95% CI (0.54, 2.61)<br><br>**No IVH or WMI**<br>n=37, 6%<br>Reference category<br><br>**GMFCS>0**<br>IVH and WMI n=16, 25%<br>WMI n=10, 33%<br>IVH n=4, 3%<br>No IVH or WMI n=13, 2%<br><br>**Epilepsy**<br>**IVH and WMI**<br>n=12, 19%<br>OR 5.44 95 % CI (2.72, 10.86)<br>aOR 4.89 95% CI (2.31, 10.35)<br><br>**WMI**<br>n=8, 27%;<br>OR 6.92 95% CI (2.86, 16.75)

Confidential: For Review Only

				aOR 7.56 95% CI (2.85, 20.06)<br><br>**IVH**<br>n= 11, 9%;<br>OR 1.85 95% CI (0.91, 3.78)<br>aOR 1.5 95% CI (0.68, 3.3)<br><br>**No IVH or WMI**<br>n=25, 4%<br>Reference category<br><br>**Neuropsychiatric/ behavioural outcomes**<br>**ASD**<br>**IVH and WMI**<br>n=4, 6%<br>OR 0.97 95% CI (0.34, 2.79)<br>aOR 0.58 95% CI (0.19, 1.77)<br><br>**WMI**<br>n=2, 7%<br>OR 1.02 95% CI (0.23, 4.42)<br>aOR 0.74 95% CI (0.09, 5.88)<br><br>**IVH**<br>n=11, 9%<br>OR 1.39 95% CI (0.69, 2.78)<br>aOR 1.24 95% CI (0.59, 2.6)<br><br>**No IVH or WMI**<br>n=42, 7%<br>Reference category<br><br>**Social responsiveness scale** (over 65 among children with IQ >85 excluding those with ASD)<br>IVH and WMI n=5, 8%<br>WMI n=4, 13%<br>IVH n=14, 11%<br>No IVH or WMI n=62, 10%<br><br>**ADHD**<br>IVH and WMI n=13, 24%<br>WMI n=3, 10%<br><br>IVH n=31, 25%<br>OR 1.6 95% CI (1.1, 2.5)<br><br>No IVH or WMI n=97, 15%<br><br>**Anxiety (parent-reported)**<br>IVH and WMI n=6, 10%<br>WMI n=3, 10%<br>IVH n=10, 8%<br>No IVH or WMI n=98, 15%<br><br>**Anxiety (teacher-reported)**<br>IVH and WMI n=12, 19%<br>WMI n=3, 10%<br>IVH n=14, 11%<br>No IVH or WMI n=88, 14%<br><br>**Depression (parent-reported)**<br>IVH and WMI n=7, 11%<br>WMI n=7, 23%<br>IVH n=14, 11%<br>No IVH or WMI n=100, 16%<br><br>**Depression (teacher-reported)**<br>IVH and WMI n=20, 32%<br>WMI n=7 23%<br>IVH n=18, 15%<br>No IVH or WMI n=96, 15%<br><br>**Poor quality of life (<70)**<br>IVH and WMI n=31, 49%<br>WMI n=12, 40%<br>IVH n=41, 25%<br>No IVH or WMI n=131, 20%
5*	Cheong 2018[17]<br><br>Australia<br><br>Three prospective cohort studies	**Population**<br>• Gestation 22-27 weeks<br>• Born 1991-1992; 1997-1998; 2005-2006<br><br>**Exposure**<br>• IVH grade 3-4 (n=100)<br>• cPVL (n=38)<br><br>**Comparator**<br>• Unmatched<br>• No IVH grade 3-4 (n=446)<br>• No cPVL (n=508)<br><br>**Ascertainment/ definition**<br>• Not specified	**Outcomes**<br>• Survival with major disability (composite)<br>• Survival without major disability (composite)<br>• Cognitive<br>• Cerebral palsy<br>• Visual impairment (acuity less than 6/60 in better eye)<br>• Hearing impairment (requiring hearing aid or cochlear amplification)<br>**Assessment/ measurement**<br>• GMFCS<br>• WISC III<br>• WISC IV<br>• Differential Abilities Scales 2nd edition<br>**Follow-up**<br>• 8 years<br>• 91% follow-up of survivors	**Survival with major disability**<br>**IVH grade 3-4**<br>OR 2·98 95% CI (1·34, 6·63) p=0.01<br>aOR 2·61 95%CI (1·11–6·15) p=0·028<br><br>1997 and 2005 cohort only:<br>OR 4·01 95% CI (1·25, 12·84) p=0.02<br><br>**cPVL**<br>OR 8·11 95% CI (3·24, 20·30) p<0.001<br>aOR 9·17 95% CI (3·57–23·53) p<0·0001<br><br>1997 and 2005 cohort only<br>OR 17·0 95% CI (4·19, 69·02) p<0·001

Confidential: For Review Only

6	Chou 2020[74]<br><br>Taiwan<br><br>Retrospective cohort study	**Population**<br>• Preterms infants <37 weeks' gestation (n=21,474)<br>• Infants born small for gestational age (n=2206)<br>• Born 2000-2010<br><br>**Exposure**<br>• Preterm with cerebral haemorrhage<br>• SGA with cerebral haemorrhage<br><br>**Comparator (n=94,720)**<br>• Matched 1:4 on gender, urbanisation of residential area and parental occupation<br>• No cerebral haemorrhage<br><br>**Ascertainment/ definition**<br>• National children's medical record database<br>• ICD 9 codes	**Outcome**<br>• Epilepsy<br><br>**Assessment/ measurement**<br>• ICD 9<br><br>**Follow-up**<br>• 2-12 years (mean 9 years)<br>• Completeness of follow-up not specified	**<u>Epilepsy</u>**<br>**Preterm with cerebral haemorrhage**<br>HR 42.4 95%CI (29.8, 60.3)<br>aHR 42.5 95 %CI (29.6, 60.5)<br><br>**SGA with cerebral haemorrhage**<br>HR 39.3 95%CI (5.51, 274.5)<br>aHR 38.7 95%CI (5.43, 275.5)
7	Davidovitch 2020[35]<br><br>Israel<br><br>Retrospective cohort study	**Population (n=4963)**<br>• VLBW infants ≤1500g<br>• Born 1999-2012<br><br>**Exposure**<br>• IVH grade 3-4 (n=256)<br>• PVL (n=200)<br>• Post-haemorrhagic hydrocephalus (n=152)<br><br>**Comparator**<br>• Unmatched<br>• No IVH grade 3-4 (n=4600)<br>• No PVL (n=3813)<br>• No post-haemorrhagic hydrocephalus (n=4810)<br><br>**Ascertainment/ definition**<br>• Israel national very low birthweight infant database linked to electronic medical records.<br>• Ultrasound diagnosis<br>• Papile classification	**Outcome**<br>• ASD<br><br>**Assessment/ measurement**<br>• Physical, neurological, and developmental assessment (by a qualified healthcare professional)<br>• Independent psychological assessment<br><br>**Follow-up**<br>• 8- 15 years (median 11.6)<br>• Only those linked to electronic medical records included	**<u>ASD</u>**<br>IVH n=10, 3.9%<br>No IVH n=103, 2.2% p=0.085<br><br>PVL n=5, 2.5%<br>No PVL n=88, 2.3% p=0.86<br><br>Post-haemorrhagic hydrocephalus n=7, 4.6%<br>No post-haemorrhagic hydrocephalus n=106, 2.2% p=0.051<br><br>IVH, PVL, post-haemorrhagic hydrocephalus or ROP n=27,23.9%<br>No brain injury n=571, 11.8% p<0.0001<br>aOR 1.62 95% CI (0.96–2.73)
8*	Doyle 2000[75]<br><br>Australia<br><br>Prospective Cohort	**Population**<br>• Birthweight 500–1499 g<br>• Born 1980-1981; 1992<br><br>**Exposure**<br>**1980s epoch**<br>• IVH grade 1 (n=18)<br>• IVH grade 2 (n=9)<br>• IVH grade 3 (n=7)<br>• IVH grade 4 (n=4)<br><br>**1992 epoch**<br>• IVH grade 1 (n=23)<br>• IVH grade 2 (n=10)<br>• IVH grade 3 (n=9)<br>• IVH grade 4 (n=1)<br><br>**Comparator**<br>• Unmatched<br>• No intracranial haemorrhage (n=223)<br>• 1980s epoch (n=110)<br>• 1992 epoch (n=113)<br><br>**Ascertainment/ definition**<br>• Ultrasound imaging<br>• Post-mortem examination<br>• Papile classification	**Outcomes**<br>• Survival<br>• Cerebral palsy<br><br>**Measurement/assessment**<br>• Clinical assessment by blinded paediatricians<br>• Functional assessment<br><br>**Follow-up**<br>• 5 years<br>• 93% follow-up for 1980s epoch<br>• 94% follow-up for 1992 epoch	**<u>Cerebral Palsy</u>**<br><br>Grade of IVH<br><br>**1980s epoch**<br>No IVH n=5, 5%<br>IVH grade 3 n=2, 29%<br>IVH grade 4 n=0<br><br>**1992s epoch**<br>No IVH n=4, 4%<br>IVH grade 3 n=3, 33%<br>IVH grade 4 n=1, 100%

Confidential: For Review Only

9	Hintz 2018[23]<br><br>USA<br><br>Retrospective cohort	**Population**<br>• Gestation 24-28 weeks<br>• Born 2005-2009<br><br>**Exposure**<br><u>MRI</u><br>• Mild WMI (n=223)<br>• Moderate WMI (n=51)<br>• Severe WMI (n=15)<br><br>• Any cerebellar lesion (n=57)<br><br>• Significant cerebellar lesion (n=39)<br><br><u>Early cranial ultrasound</u><br>• No IVH 3-4 or cPVL (n=341)<br>• IVH 3-4 or cPVL (n=32)<br><br><u>Late cranial ultrasound</u><br>• No porencephalic cyst, cPVL, moderate to severe ventricular enlargement or shunt (n=354)<br>• Porencephalic cyst, cPVL, moderate to severe ventricular enlargement or shunt (n=19)<br><br>**Comparator**<br>• No white matter injury on MRI (n=84)<br>• No cerebellar lesion on MRI (n=316)<br>• No IVH 3-4 or cPVL (n=32)<br>• Normal early cranial ultrasound (n=227)<br>• No porencephalic cyst, cPVL moderate to severe ventricular enlargement or (n=19)<br>• Normal late cranial ultrasound (n=284)<br><br>**Ascertainment/ definition**<br>• NICHD neonatal research network (NEURO study and SUPPORT cohort)<br>• Two masked central imaging readers for all cranial ultrasounds and one for MRI<br>• All had cranial ultrasound and MRI (at 35-42 weeks)<br>• Unilateral and bilateral cranial ultrasound lesions combined	**Outcomes**<br>• Moderate to severe disability (composite)<br>• Minimal or no disability<br>• Cognitive<br>• Cerebral palsy<br>• Hearing<br>• Vision<br><br>**Measurement/ assessment**<br>• WISC IV<br>• Neurological exam<br>• GMFCS<br>• Clinical examination<br>• Parental report<br><br>**Follow-up**<br>• 6-7 years<br>• 83.3% follow-up of survivors	<u>**White matter injury**</u><br>**Moderate to severe disability**<br>No white matter injury, n=8, 9%<br>Mild white matter injury, n=27, 12%<br>Moderate white matter injury, n=8, 15%<br>Severe white matter injury, n=14, 82%<br>p<0.0001<br><br>Moderate or severe white matter injury<br>aOR 1.1 95% CI (0.42, 2.92)<br><br>**Minimal or no disability**<br>No white matter injury, n=47, 55%<br>Mild white matter injury, n=88, 224%<br>Moderate white matter injury, n=15, 28%<br>Severe white matter injury, n=0, 0%<br>p<0.0001<br><br>**Cognitive impairment (FSIQ mean (SD))**<br>No white matter injury, 90.1 (15.5)<br>Mild white matter injury, 85.9 (16.8)<br>Moderate white matter injury, 84 (17)<br>Severe white matter injury, 62.7 (19.6)<br>p<0.0001<br><br>**Cognitive impairment FSIQ <70**<br>No white matter injury, n=7, 8%<br>Mild white matter injury, n=25, 11%<br>Moderate white matter injury, n=6, 12%<br>Severe white matter injury, n=9, 60%<br>p<0.0001<br><br>Moderate or severe white matter injury<br>aOR 1.14 95% CI (0.39, 3.26)<br><br>**Cognitive impairment FSIQ <85**<br>No white matter injury, n=27, 32%<br>Mild white matter injury, n=100, 45%<br>Moderate white matter injury, n=29, 57%<br>Severe white matter injury, n=13, 87%<br>p<0.0001<br><br>**No cognitive impairment FSIQ ≥85**<br>No white matter injury, n=57, 68%<br>Mild white matter injury, n=123, 55%<br>Moderate white matter injury, n=22, 43%<br>Severe white matter injury, n=2, 13%<br>p<0.0001<br><br>**Any cerebral palsy**<br>No white matter injury, n=2, 2%<br>Mild white matter injury, n=6, 3%<br>Moderate white matter injury, n=4, 7%<br>Severe white matter injury, n=10, 59%<br>p<0.0001<br><br>**Cerebral palsy with GMFCS ≥2**<br>No white matter injury, n=0, 0%<br>Mild white matter injury, n=1, 0%<br>Moderate white matter injury, n=1, 2%<br>Severe white matter injury, n=4, 24%<br>p<0.0001<br><br><u>**Cerebellar lesions**</u><br>**Moderate to severe disability**<br>No cerebellar lesion, n=37, 12%<br>Any cerebellar lesion, n=20, 33% p<0.0001<br>Significant cerebellar lesion, n=15, 36%<br><br>Significant cerebellar lesions<br>aOR 2.71 95% CI (1.09, 6.71)<br><br>**Minimal or no disability**<br>No cerebellar lesion, n=135, 42%<br>Any cerebellar lesion n=15, 25% p<0.0001<br>Significant cerebellar lesion, n=15, 36%<br><br>**Cognitive impairment (FSIQ mean (SD))**<br>No cerebellar lesion, 87 (16.5)<br>Any cerebellar lesion 78.4 (20) p=0.001<br>Significant cerebellar lesion 76.8 (20.4)<br><br>**Cognitive impairment FSIQ <70**<br>No cerebellar lesion, n=32, 10%<br>Any cerebellar lesion, n=15, 26% p=0.001<br>Significant cerebellar lesion, n=10, 26%<br><br>Significant cerebellar lesions<br>aOR 1.96 95% CI (0.72, 5.36)<br><br>**Cognitive impairment FSIQ <85**<br>No cerebellar lesion, n=136, 43%<br>Any cerebellar lesion, n=33, 58% p=0.038<br>Significant cerebellar lesion, n=22, 56%<br><br>**No cognitive impairment FSIQ ≥85**<br>No cerebellar lesion, n=180, 57%<br>Any cerebellar lesion, n=24, 42% P=0.038<br>Significant cerebellar lesion, n=17, 44%<br><br>**Any cerebral palsy**

Confidential: For Review Only

				No cerebellar lesion, n=13, 4%<br>Any cerebellar lesion, n=9, 15% p=0.001<br>Significant cerebellar lesion, n=9, 21%<br><br>**Cerebral palsy with GMFCS ≥2**<br>No cerebellar lesion, n=3, 1%<br>Any cerebellar lesion, n=3, 5% p=0.19<br>Significant cerebellar lesion, n=3, 7%<br><br>**Early cranial ultrasound abnormalities**<br>**Moderate to severe disability**<br>No IVH 3-4 or cPVL, n=43, 12%<br>IVH 3-4 or cPVL, n=14, 42% p<0.0001<br>Normal scan, n=35, 12%<br>aOR 0.61 95% CI (0.14, 2.59)<br><br>**Minimal or no disability**<br>No IVH 3-4 or cPVL, n=143, 41%<br>IVH 3-4 or cPVL, n=7, 21% p<0.0001<br>Normal scan, n=120, 43%<br><br>**Cognitive impairment, FSIQ mean (SD)**<br>No IVH 3-4 or cPVL, 86.4 (17)<br>IVH 3-4 or cPVL, 77.9 (19.1) p=0.008<br>Normal scan, 86 (16.7)<br><br>**Cognitive impairment FSIQ <70**<br>No IVH 3-4 or cPVL, n=38, 11%<br>IVH 3-4 or cPVL, n=9, 28% p=0.006<br>Normal scan, n=31, 11%<br>aOR 0.42 95% CI (0.07, 2.33)<br><br>**Cognitive impairment FSIQ <85**<br>No IVH 3-4 or cPVL, n=149, 44%<br>IVH 3-4 or cPVL, n=20, 63% p=0.041<br>Normal scan, n=123, 44%<br><br>**No cognitive impairment FSIQ ≥85**<br>No IVH 3-4 or cPVL, n=192, 56%<br>IVH 3-4 or cPVL, n=12, 38% p=0.041<br>Normal scan, n=154, 56%<br><br>**Any cerebral palsy**<br>No IVH 3-4 or cPVL, n=149, 44%<br>IVH 3-4 or cPVL, n=20, 63% p=0.041<br>Normal scan, n=123, 44%<br><br>**Cerebral palsy with GMFCS ≥2**<br>No IVH 3-4 or cPVL, n=3, 1%<br>IVH 3-4 or cPVL, n=3, 9% p<0.0001<br>Normal scan, n=2, 1%<br><br>**Late cranial ultrasound abnormalities**<br>**Moderate to severe disability**<br>No porencephalic cyst, cPVL, moderate to severe ventricular enlargement or shunt, n=40, 11%<br>Porencephalic cyst, cPVL, moderate to severe ventricular enlargement or shunt, n=17, 77% p<0.0001<br>Normal scan, n=27, 10%<br>aOR 27.85 95% CI (6.03, 128.68)<br><br>**Minimal or no disability**<br>No porencephalic cyst, cPVL, moderate to severe ventricular enlargement or shunt, n=149, 42%<br>Porencephalic cyst, cPVL, moderate to severe ventricular enlargement or shunt, n=1, 5% P<0.0001<br>Normal scan, n=117, 43%<br><br>**Cognitive impairment (FSIQ mean (SD))**<br>No porencephalic cyst, cPVL, moderate to severe ventricular enlargement or shunt, 86.7 (16.7)<br>Porencephalic cyst, cPVL, moderate to severe ventricular enlargement or shunt, 65.9 (18.7) P<0.0001<br>Normal scan, 87 (16.1)<br><br>**Cognitive impairment FSIQ <70**<br>No porencephalic cyst, cPVL, moderate to severe ventricular enlargement or shunt, n=36, 10%<br>Porencephalic cyst, cPVL, moderate to severe ventricular enlargement or shunt, n=11, 58% p<0.0001<br>Normal scan, n=24, 9%<br>aOR 20.05 95% CI (3.63, 110.84)<br><br>**Cognitive impairment FSIQ <85**<br>No porencephalic cyst, cPVL, moderate to severe ventricular enlargement or shunt, n=153, 43%<br>Porencephalic cyst, cPVL, moderate to severe ventricular enlargement or shunt, n=16, 84% p<0.0001<br>Normal scan, n=118, 43%<br><br>**No cognitive impairment FSIQ ≥85**<br>No porencephalic cyst, cPVL, moderate to severe ventricular enlargement or shunt, n=201, 57%<br>Porencephalic cyst, cPVL, moderate to severe ventricular enlargement or shunt, n=3, 16% p<0.0001<br>Normal scan, n=156, 57%<br><br>**Any cerebral palsy**<br>No porencephalic cyst, cPVL, moderate to severe ventricular enlargement or shunt, n=10, 3%<br>Porencephalic cyst, cPVL, moderate to severe ventricular enlargement or shunt,

Confidential: For Review Only

				n=12, 50% p<0.0001<br>Normal scan, n=6, 2%<br><br>**Cerebral palsy with GMFCS ≥2**<br>No porencephalic cyst, cPVL, moderate to severe ventricular enlargement or shunt, n=2, 1%<br>Porencephalic cyst, cPVL, moderate to severe ventricular enlargement or shunt, n=4, 17% p<0.0001<br>Normal scan, n=1, 0%
10	Hirovonen, 2017[28]<br><br>Finland<br><br>Retrospective cohort	**Population**<br>• Gestation >22 weeks<br>• Birth weight >500g<br>• Born 1991-2008<br><br>**Exposure (n=557)**<br>• Intracranial haemorrhage<br><br>**Comparison (n=708,977)**<br>• No intracranial haemorrhage<br>• ICD code<br><br>**Ascertainment/ definition**<br>• Finnish national register<br>• ICD codes	**Outcomes**<br>• Cognitive<br><br>**Measurement/ assessment**<br>• ICD 9 and 10 codes<br>• BSID 1993<br>• Finnish WISC<br><br>**Follow-up**<br>• 7 years<br>• 98% follow-up	**Any intellectual disability after intracranial haemorrhage (HR (95%CI); p-value)**<br>Very preterm infants 2.92 (1.58–5.41); p= 0.001<br>Moderately preterm 5.59 (1.57–19.9); p= 0.008<br>Late preterm 4.58 (1.36–15.4); p= 0.014<br>Term 2.94 (1.08-8); p=0.035

Confidential: For Review Only

11 *	Hollebrandse 2021[25]<br><br>Australia<br><br>Retrospective cohort	**Population**<br>• Gestation <28 weeks<br>• Born 1991-1992, 1997, 2005<br><br>**Exposure**<br>• IVH grade 1 n=80<br>• IVH grade 2 n=53<br>• IVH grade 3 n=23<br>• IVH grade 4 n=12<br><br>**Comparator**<br>• Unmatched<br>• Preterm infants without IVH n=331<br><br>**Ascertainment/ definition**<br>• Ultrasound diagnosis<br>• Worst grade of IVH<br>• Papile classification	**Outcomes**<br>• Cognitive<br>• Motor<br>• Cerebral palsy<br><br>**Assessment/ measurement**<br>• WISC III (1991-1992 cohort)<br>• WISC IV (1997 cohort)<br>• Differential Abilities Scale 2nd edition (2005 cohort)<br>• WRAT III (1991-92; 1997 cohorts)<br>• WRAT IV (2005 cohort)<br>• Behaviour rating inventory of executive functioning (parent-completed)<br>• Movement ABC 1st edition (1991-1992 and 1997 cohorts)<br>• Movement ABC 2nd edition (2005 cohort)<br>• GMFCS (1997 and 2005 cohort)<br>• Blinded assessment<br><br>**Follow-up**<br>• 8 years<br>• Follow-up 85-91.4%	<u>**Cognitive**</u><br>**IQ score <-2 SD**<br>IVH grade 4 n=5, 42% p=0.08 (X^2 trend)<br>IVH grade 3 n=5, 22%<br>No IVH n=41, 12%<br><br>IVH 3-4: OR 2.68 95% CI (1.21, 5.94) p=0.01<br><br>**Impaired executive function**<br>**Global executive composite ≥65**<br>IVH grade 4 n=2, 18% p=0.78 (X^2 trend)<br>IVH grade 3 n=4, 18%<br>No IVH n=49, 16%<br><br>IVH 3-4: OR 1.17 95% CI (0.46, 2.97) p=0.75<br><br>**Behavioural regulation index ≥65**<br>IVH grade 4 n=2, 18% p=0.21 (X^2 trend)<br>IVH grade 3 n=6, 27%<br>No IVH n=46, 15%<br><br>IVH 3-4: OR 1.76 95% CI (0.75, 4.11) p=0.2<br><br>**Metacognition index ≥65**<br>IVH grade 4 n=3, 27% p=0.1 (X^2 trend)<br>IVH grade 3 n=5, 23%<br>No IVH n=48, 16%<br><br>IVH 3-4: OR 1.73 95% CI (0.74, 4.06) p=0.21<br><br>**Impaired academic skills (any academic skill <-2SD)**<br>IVH grade 4 n=7, 64% p<0.001 (X^2 trend)<br>IVH grade 3 n=5, 24%<br>No IVH n=50, 16%<br><br>IVH 3-4: OR 2.91 95% CI (1.35, 6.27) p=0.006<br><br>**Impaired reading <-2SD**<br>IVH grade 4 n=6, 55% p=0.002 (X^2 trend)<br>IVH grade 3 n=4, 19%<br>No IVH n=21, 10%<br><br>IVH 3-4: OR 3.62 95% CI (1.59, 8.24) p=0.002<br><br>**Impaired spelling <- 2 SD**<br>IVH grade 4 n=5, 45% p=0.011 (X^2 trend)<br>IVH grade 3 n=3, 14%<br>No IVH n=21, 7%<br><br>IVH 3-4: OR 4.48 95% CI (1.8, 11.2) p=0.001<br><br>**Impaired arithmetic < -2 SD**<br>IVH grade 4 n=5, 45% p=0.09 (X^2 trend)<br>IVH grade 3 n=4, 19%<br>No IVH n=38, 12%<br><br>IVH 3-4: OR 2.79 95% CI (1.2, 6.48) p=0.017<br><br><u>**Motor and cerebral palsy**</u><br>**Any motor dysfunction (cerebral palsy or MABC <5th centile)**<br>IVH grade 4 n=11, 92% p<0.001 (X^2 trend)<br>IVH grade 3 n=10, 43%<br>No IVH n=81, 24%<br><br>IVH 3-4: OR 4.45 95% CI (2.18, 9.08) p<0.001<br><br>**Cerebral palsy**<br>IVH grade 4 n=9, 75% p<0.001(X^2 trend)<br>IVH grade 3 n=6, 26%<br>No IVH n=26, 8%<br><br>IVH 3-4: OR 8.8 95% CI (4.03, 19.2) p<0.001<br><br>**MABC <5th percentile (for the 2005 cohort)**<br>IVH grade 4 n=11, 92% p<0.001 (X^2 trend)<br>IVH grade 3 n=9, 45%<br>No IVH n=79, 26%<br><br>IVH 3-4: OR 4.7 95% CI (2.21, 9.97) p<0.001

Confidential: For Review Only

12	Hreinsdottir 2018[54]<br><br>Sweden<br><br>Prospective cohort study	**Population**<br>• Born 2004-2007<br>• Gestation <32 years<br><br>**Exposure (n=9)**<br>• IVH grade 3-4 and/ or PVL<br><br>**Comparator (n=99)**<br>• Unmatched<br>• No IVH grade 3-4 or PVL<br><br>**Ascertainment/ definition**<br>• Ultrasound imaging performed by paediatric radiologist<br>• Papile classification for IVH<br>• PVL defined by size, laterality and as cystic of diffuse	**Outcomes**<br>• Visual impairment<br><br>**Assessment/ measurement**<br>• Linear visual acuity (Lea Hyvarinen chart)<br>• Cover test<br>• Refraction<br><br>**Follow-up**<br>• 6.5 years<br>• 78% follow-up	**<u>Vision</u>**<br>**Subnormal visual acuity**<br>IVH 3-4 and or PVL<br>OR 1.11 95% CI (0.25, 4.83) p=0.891<br><br>**Contrast sensitivity**<br>IVH 3-4 and or PVL<br>OR 1.87 95% CI (0.43, 8.17) p=0.403<br><br>**Refractive error**<br>IVH 3-4 and or PVL<br>OR 2.5 95% CI (0.55, 11.41) p=0.237<br><br>**Manifest strabismus**<br>IVH 3-4 and or PVL<br>OR 4 95% CI (0.65, 24.55) p=0.134<br><br>**<u>Composite score 1:</u> Visual acuity with both eyes of less than 0.3, significant refractive error in the better eye and manifest strabismus**<br>IVH 3-4 and or PVL<br>OR 3.63 95% CI (0.86, 15.41) p=0.08<br>aOR 4.95 95% CI (0.65, 37.48) p=0.121<br><br>**<u>Composite score 2:</u> Visual acuity in worse eye of less than 0.3, significant refractive error in worse eye according and manifest strabismus**<br>IVH 3-4 and or PVL<br>OR 5.67 95% CI (1.34, 24.07) p=0.019<br>aOR 10.4 95% CI (1.23, 88) p=0.032<br><br>**<u>Composite score 3:</u> Visual acuity with both eyes of less than 0.5, significant refractive error in the better eye, manifest strabismus, negative stereopsis and contrast sensitivity less than 0.4**<br>IVH 3-4 and or PVL<br>OR 7.6 95% CI (1.7, 34) p=0.008<br>aOR 18.19 95% CI (2.15, 154.05) p=0.008<br><br>**<u>Composite score 4:</u> Visual acuity with both eyes of 0.8 or less, significant refractive error in the better eye, manifest strabismus, negative stereopsis and CS less than 0.5**<br>IVH 3-4 and or PVL<br>OR 4.63 95% CI (0.9, 23.85) p=0.067<br>a6.23 95% CI (1.15, 33.83) p=0.034
13	Jansen 2020[29]<br><br>Netherlands<br><br>Prospective cohort study	**Population**<br>• Gestation <32 weeks<br>• Admitted 2006-2007<br><br>**Exposure**<br>• Mild WMI (n=18)<br>• Moderate WMI (n=14)<br>• Severe WMI (n=8)<br>• Mild cerebellar injury (n=11)<br>• Moderate cerebellar injury (n=4)<br>• Severe cerebellar injury (n=6)<br><br>**Comparator**<br>• Unmatched<br>• No WMI (n=46)<br>• No cerebellar injury (n=65)<br><br>**Ascertainment/ definition**<br>• Ultrasound imaging and term MRI<br>• Imaging reviewed by two blinded experienced investigators (neonatologists or radiologists)	**Outcomes**<br>• Cognitive<br><br>**Assessment/ measurement**<br>• National standardised achievement tests<br><br>**Follow-up**<br>• 9-10 years<br>• 77% follow-up	**<u>Cognitive</u>**<br>**Reading comprehension**<br>Moderate-severe WMI vs. no injury<br>B 0.241 p=0.483<br><br>Moderate-severe cerebellar injury vs. no injury<br>B 0.799 p=0.325<br><br>**Spelling**<br>Moderate-severe WMI vs. no injury<br>B 1.076 p=0.075<br><br>Moderate-severe cerebellar injury vs. no injury<br>B 1.293 p= 0.115<br><br>**Mathematics**<br>Moderate-severe WMI vs. no injury<br>B 1.856 p=0.003<br><br>Moderate-severe cerebellar injury vs. no injury<br>B 1.504 p=0.088
14	Kaur 2020[38]<br><br>Canada<br><br>Retrospective cohort study	**Population**<br>• Preterm and term infants<br>• Born 2006-2016<br><br>**Exposure**<br>• IVH grade 1 (n=811)<br>• IVH grade 2 (n=186)<br>• IVH grade 3-4 (n=194)<br>• Preterm haemorrhage (n=1139)<br><br>**Comparator**<br>• Unmatched<br>• No IVH (n=793, 062)<br>• Preterm no haemorrhage (n=50, 185)<br><br>**Ascertainment/ definition**<br>• ICD 10 codes (based on ultrasound or MRI imaging)<br>• Papile classification	**Outcome**<br>• Reason for hospitalisation<br><br>**Assessment/ measurement**<br>• ICD 10 codes<br><br>**Follow-up**<br>• 12 years<br>• Completeness of follow-up not specified	**<u>Incidence of hospitalisation for:</u>**<br>**Cerebral palsy, n, incident rate per 1,000 person years (95%CI)**<br>IVH n=57, 6.8 (5.3, 8.8)<br>No haemorrhage n=432, 0.1 (0.1, 0.1)<br>Hazard ratio: 4.78 95% CI (3.21, 7.13)<br><br>IVH grade 3-4 n=24 HR 14.78 95% CI (8.72-25.06)<br><br>**Ophthalmologic, n, incident rate per 1,000 person years (95%CI)**<br>IVH n=91 11.1 (9, 13.6)<br>No haemorrhage n=6773, 1.2 (1.2, 1.3)<br>HR 3.01 95% CI (2.32, 3.89)<br><br>IVH grade 3-4 n=32 HR 7.87 95% CI (5.31-11.67)<br><br>**Otologic n, incident rate per 1,000 person years (95%CI)**<br>IVH n=328, 46.7 (41.9, 52)<br>No haemorrhage n=102,153 22.1 (22, 22.2)<br>HR 1.19 95% CI (1.06, 1.34)<br><br>IVH grade 3-4 n=202 HR 1.07 95% CI (0.79-1.46)

Confidential: For Review Only

15	Kiechl-Kohlendorfer 2013[34]<br><br>Austria<br><br>Prospective cohort	**Population**<br>• Gestation <32 weeks<br>• Born 2003-2006<br><br>**Exposure**<br>• Intracranial haemorrhage (all grades) (n=24)<br>• Intracranial haemorrhage grade 3-4 (n=4)<br>• PVL (n=2)<br>• Intraparenchymal echodense lesions (n=2)<br><br>**Comparator**<br>• Unmatched<br><br>**Ascertainment/ definition**<br>• Ultrasound imaging<br>• Papile classification	**Outcomes**<br>• Cognitive<br><br>**Measurement/assessment**<br>• Physical examination<br>• Hannover-Wechsler Intelligence Test for preschool children, third edition<br>• WPPSI<br>• Snijders-Oomen Nonverbal Intelligence Test<br>• TEDI-MATH<br><br>**Follow-up**<br>• 5 years<br>• 72.2% follow-up	**Delayed numerical skills**<br>Intracranial haemorrhage (all grades) n=11, 40,7%<br>aOR 4.66 95% CI (1.56, 13.93) p=0.007<br><br>Intracranial haemorrhage grade 3-4 n=3, 11.1%<br>PVL n=2, 7.4%<br>Intraparenchymal echodense lesions n=0
16	Klebermass-Schrehof 2012[26]<br><br>Austria<br><br>Prospective cohort	**Population**<br>• Gestation <32 weeks<br>• Admitted to NICU 1994-2005<br><br>**Exposure**<br>• IVH grade 1 (n=37)<br>• IVH grade 2 (n=84)<br>• IVH grade 3 (n=18)<br>• IVH grade 4 (n=12)<br><br>**Comparator (n=320)**<br>• Unmatched<br>• No IVH<br><br>**Ascertainment/ definition**<br>• Ultrasound diagnosis<br>• Most severe scan used<br>• Papile classification	**Outcomes**<br>• Neurosensory impairment (composite)<br>• Motor<br>• Cerebral palsy<br>• Language<br>• Visual<br>• Hearing<br><br>**Measurement/assessment**<br>• BSID II (MDI, PDI)<br>• K-ABC<br>• Beery-Buktenica Developmental Test of VMI<br>• Clinical assessment<br><br>**Follow-up**<br>• 5 years (1 ,2, and 3.5 years)<br>• Only those with follow-up included (loss to follow-up not specified)	**<u>Outcomes at 5.5 years</u>**<br><br>**<u>Group 1: infants born < 28 weeks' gestation</u>**<br>**KABC <70**<br>No IVH, 7.6%<br>IVH grade 3, 33.3%<br>IVH grade 4, 50%<br><br>**KABC mean (SD)**<br>No IVH, 91.5 (15.1)<br>IVH grade 3, 88.6 (11.1) p=not significant<br>IVH grade 4, 88.5 (10.6) p= not significant<br><br>**VMI mean (SD)**<br>No IVH, 92.7 (20)<br>IVH grade 3, 67.5 (14) p=0.04<br>IVH grade 4, 76 (26.8) p=0.04<br><br>**Cerebral palsy**<br>No IVH, 14.3%<br>IVH grade 3, 63.6% p<0.01<br>IVH grade 4, 90.9% p<0.01<br><br>**Visual impairment**<br>No IVH, 7.5%<br>IVH grade 3, 45.5%, p=0.03<br>IVH grade 4, 90.9% p<0.01<br><br>**Acoustic impairment**<br>No IVH, 2.2%<br>IVH grade 3, 0% p= not significant<br>IVH grade 4, 0% p= not significant
17	Koc 2016[30]<br><br>Turkey<br><br>Retrospective cohort	**Population (n=90)**<br>• Gestation <32 weeks<br>• Birthweight <1500g<br>• Born 2001<br><br>**Exposure**<br>• IVH grade 1-2 (n= 7)<br>• IVH grade 3-4 (n= 8)<br><br>**Comparator**<br>• No IVH (n=75)<br><br>**Ascertainment/ definition**<br>• Neonatal unit database and medical records	**Outcomes**<br>• Cognitive<br><br>**Measurement/ assessment**<br>• WISC-R<br><br>**Follow-up**<br>• 5.9-7.9 years<br>• 100% follow-up	**<u>WISC-R score <85</u>**<br>IVH (n=7; 46.7%)<br>No IVH (n= 25; 33.3%)<br><br>**<u>WISC-R score >85</u>**<br>IVH grade (n=8; 13.8%)<br>No IVH (n= 50; 84.2%)<br><br>p=0.381
18	Martinez-Cruz 2008[51]<br><br>Mexico<br><br>Case control	**Population**<br>• Gestation <34 weeks<br>• Birthweight <1500g<br>• Born 1990-2005<br><br>**Exposure (n=103)**<br>• IVH<br><br>**Comparator (n=315)**<br>• No IVH<br><br>**Ascertainment/ definition**<br>• Medical records<br>• Ultrasound diagnosis.<br>• Papile classification.	**Outcomes**<br>• Sensorineural hearing loss<br><br>**Measurement/ assessment**<br>• Brainstem auditory evoked potentials<br>• Transient auditory evoked otoacoustic emissions<br>• Behavioural hearing evaluation<br>• Free field audiometry<br>• Tympanometry<br>• Pure Tone Audiometry<br><br>**Follow-up**<br>• Mean age 7.8±3.7 years<br>• 100% follow-up (case control)	**IVH**<br>Sensorineural hearing loss (n=71; 48.6%)<br>No sensorineural hearing loss (n=32; 11.8%)<br><br>**<u>Multivariate logistic regression of risk factors for sensorineural hearing loss</u>**<br>IVH: aOR 7.1 95% CI (4.34, 11.6) p<0.000

Confidential: For Review Only

19	Neubauer 2008[18]<br><br>Germany<br><br>Prospective cohort	**Population**<br>• Birthweight <1000g<br>• Born 1993-1998<br><br>**Exposure**<br>• IVH grade 1-2 (n=26)<br>• IVH grade 3-4, PVL (n=18)<br><br>**Comparator**<br>• Unmatched<br>• No IVH or PVL (n=91)<br><br>**Ascertainment/ definition**<br>• Ultrasound diagnosis<br>• Papile classification	**Outcomes**<br>• Neurodevelopmental impairment (composite)<br><br>**Measurement/assessment**<br>• Modified Touwen test<br>• K-ABC<br>• Snijders-Oomen Non-Verbal Intelligence Test<br>• Hamburg-Wechsler Intelligence Test for Children<br><br>**Follow-up**<br>• 10 years<br>• 79% follow-up	**<u>Logistic regression for major impairment vs. normal development or minor impairment at school age</u>**<br><br>**Grade 3-4 IVH or PVL**<br>Normal (n=4, 22%)<br>Minor (n=2, 11%)<br>Major (n=12, 67%)<br>Risk of impairment: OR 2.46 95% CI (0.52–11.7)
20	Piris Borregas 2019[19]<br><br>Spain<br><br>Retrospective cohort study	**Population (n=1001)**<br>• Birthweight 500-1250g<br>• Born 1991-2008<br><br>**Exposure**<br>• Severe brain injury (IVH grade 3-4, ventriculomegaly III, PVL or intraparenchymal echodense lesion grade 3 or greater)<br><br>**Comparator**<br>• Unmatched<br><br>**Ascertainment/ definition**<br>• Neonatal database<br>• Ultrasound diagnosis<br>• Papile classification	**Outcomes**<br>• Neurodevelopment (composite)<br>• Cognitive<br>• Motor<br>• Hearing impairment<br>• Visual impairment<br><br>**Assessment/ measurement**<br>• GMFCS<br><br>**Follow-up**<br>• 7 years	**<u>Poor neurodevelopmental outcome</u>**<br>**Severe brain injury**, n=46, 32%<br>**No severe brain injury**, n=208, 24%<br>OR 1.41 95% CI (0.94, 2.10) p=0.09<br>Independent OR 2.02 95% CI (1.22, 3.31) p=0.18<br><br>**Severe brain injury (birthweight 500-1000g)**<br>Independent OR 2.02 95% CI (1.22, 3.31)
21	Pittet 2019[31]<br><br>Switzerland<br><br>Prospective cohort study	**Population**<br>• Gestation <30 weeks<br>• Born 2006<br><br>**Exposure**<br>• IVH grade 3-4 or cPVL (n=22)<br><br>**Comparator**<br>• Unmatched<br>• No IVH grade 3-4 or cPVL (n=213)<br><br>**Ascertainment/ definition**<br>• Swiss neonatal network follow-up group	**Outcomes**<br>• Cognitive<br>• Cerebral palsy<br>• Visual impairment<br>• Hearing impairment<br><br>**Assessment/ measurement**<br>• Kaufman ABC<br>• Neurological exam<br>• GMFCS<br><br>**Follow-up**<br>• 5.5 – 6 years<br>• 81% follow-up	**<u>Cognitive (K-ABC – MPC score < 1SD)</u>**<br>IVH 3-4 or PVL<br>OR 2.9 95% CI (1, 8.2) p=0.04<br>aOR 2.3 95% CI (0.7, 7.7) p=0.15<br><br>**<u>Use of early intervention/ therapy service</u>**<br>IVH 3-4 or cPVL aOR 2.7 95% CI (1.3, 5.7)

Confidential: For Review Only

22<br>*	Sherlock 2005[20]<br><br>Australia<br><br>Prospective cohort	**Population**<br>• Gestation <28 weeks<br>• Birthweight <1000g<br>• Survivors born 1991-1992<br><br>**Exposure**<br>• IVH Grade 1 (n=47)<br>• IVH Grade 2 (n= 25)<br>• IVH Grade 3 (n= 12)<br>• IVH Grade 4 (n= 6)<br><br>**Comparator**<br>• Matched on sex, mother's country of birth, and health insurance status<br>• Extremely low birth weight or very preterm infants without IVH (n=180)<br><br>**Ascertainment/ definition**<br>• Enrolled in Victorian Collaborative Study<br>• Ultrasound diagnosis (at least one scan by a certified sonographer)<br>• Worst grade of IVH on either side used<br>• Papile classification	**Outcomes**<br>• Disability (composite)<br>• Neurosensory disability (composite)<br>• Cognitive<br>• Motor<br>• Cerebral palsy<br>• Speech and language<br>• Visual impairment<br>• Hearing impairment<br><br>**Measurement/assessment**<br>• Medical assessment<br>• Movement ABC<br>• WISC-III<br>• Tower of London<br>• Rey Complex Figure<br>• WRAT<br><br>**Follow-up**<br>• Mean 8.7 years<br>• 92.3% follow-up	**Abnormal movement**<br>No IVH (n=39, 22.5%)<br>Grade 1 IVH (n=11, 25%)<br>Grade 2 IVH (n=6, 30%)<br>Grade 3 IVH (n=3, 27.3%)<br>Grade 4 IVH (n=4, 100%)<br>X^2 linear trend = 5.3; P = 0.021<br><br>**Cerebral palsy**<br>No IVH (n=12, 6.7%)<br>Grade 1 IVH (n=3, 6.4%)<br>Grade 2 IVH (n=6, 24%)<br>Grade 3 IVH (n=2, 16.7%)<br>Grade 4 IVH (n=6, 100%)<br>X^2 linear trend = 31.7; p <0.0001<br><br>**Moderate to severe cerebral palsy**<br>No IVH (n=4, 2.2%)<br>Grade 1 IVH (n=0, 0%)<br>Grade 2 IVH (n=4, 15%)<br>Grade 3 IVH (n=1, 8.3%)<br>Grade 4 IVH (n=5, 83.3%)<br>X^2 linear trend = 40.8; p <0.0001<br><br>**Major neurosensory disability**<br>No IVH (n=28, 15.6%)<br>Grade 1 IVH (n=5, 10.6%)<br>Grade 2 IVH (n=5, 20%)<br>Grade 3 IVH (n=1, 8.3%)<br>Grade 4 IVH (n=6, 100%)<br>X^2 linear trend = 6.9; p = 0.009<br><br>**IQ score mean (SD)**<br>No IVH 0.71 (1.25)<br>Grade 1 IVH 0.76 (1.32)<br>Grade 2 IVH 0.71 (1.12)<br>Grade 3 IVH 1.21 (1.13)<br>Grade 4 IVH 3.28 (0.88)<br>ANOVA F4,265 = 6.7; p<0.0001<br><br>**Verbal comprehension index mean (SD)**<br>No IVH 96.6 (16.2)<br>Grade 1 IVH 96.3 (15.7)<br>Grade 2 IVH 99.6 (12.8)<br>Grade 3 IVH 93.1 (15.4)<br>Grade 4 IVH 74.3 (12.7)<br>ANOVA F4,251 = 1.8; p = 0.12<br><br>**Perceptual organisation index mean (SD)**<br>No IVH 98.5 (16.3)<br>Grade 1 IVH 98.2 (15.7)<br>Grade 2 IVH 96.9 (14.8)<br>Grade 3 IVH 91.6 (12.7)<br>Grade 4 IVH 71.7 (11.1)<br>ANOVA F4,249 = 2.5; p = 0.042<br><br>**Freedom from distractibility index mean (SD)**<br>No IVH 92.3 (114.9)<br>Grade 1 IVH 95.5 (15.0)<br>Grade 2 IVH 97.7 (12.8)<br>Grade 3 IVH 94.9 (17.4)<br>Grade 4 IVH 71.0 (3.5)<br>ANOVA F4,250 = 2.8; p = 0.026<br><br>**Processing speed index mean (SD)**<br>No IVH 99.5 (15.8)<br>Grade 1 IVH 99.1 (16.6)<br>Grade 2 IVH 99.3 (13.0)<br>Grade 3 IVH 94.9 (19.3)<br>Grade 4 IVH 71.0 (9.5)<br>ANOVA F4,245 = 2.7; p = 0.033<br><br>**Tower of London (executive function) raw score mean (SD)**<br>No IVH 73.3 (14.4)<br>Grade 1 IVH 71.5 (12.4)<br>Grade 2 IVH 71.1 (20.4)<br>Grade 3 IVH 66.5 (8.3)<br>Grade 4 IVH 54.3 (22.0)<br>ANOVA F4,244 = 1.8; p = 0.13<br><br>**Rey complex figure (executive function) raw score mean (SD)**<br>No IVH 22.5 (7.5)<br>Grade 1 IVH 23.1 (7.4)<br>Grade 2 IVH 24.2 (5.8)<br>Grade 3 IVH 19.3 (8.3)<br>Grade 4 IVH 11.2 (9.8)<br>ANOVA F4,242 = 2.6; p = 0.037<br><br>**Wide range achievements test score mean (SD)**<br>**Reading**<br>No IVH 95.2 (15.7)<br>Grade 1 IVH 102.7 (15.4)<br>Grade 2 IVH 99.0 (14.2)<br>Grade 3 IVH 98.1 (11.9)<br>Grade 4 IVH 70.5g (20.9)<br>ANOVA F4,251 = 5.1; p = 0.001<br><br>**Spelling**<br>No IVH 93.6 (12.4)<br>Grade 1 IVH 97.8 (12.3)<br>Grade 2 IVH 95.9 (10.8)

Confidential: For Review Only

				Grade 3 IVH 96.8 (11.9)<br>Grade 4 IVH 73.5 (20.0)<br>ANOVA F4,250 = 4.0; p = 0.003<br><br>**Arithmetic**<br>No IVH 88.3 (14.3)<br>Grade 1 IVH 93.6 (14.9)<br>Grade 2 IVH 92.6 (10.6)<br>Grade 3 IVH 89.1 (10.1)<br>Grade 4 IVH 65.5 (14.5)<br>ANOVA F4,248 = 4.5; p = 0.002<br><br>**Cognitive test scores (compared to normal birthweight controls)**<br>**IQ score <1 SD from the mean (n, %)**<br>No IVH n=64 (35.6%)<br>Grade 1 IVH n=18 (38.3%)<br>Grade 2 IVH n=9 (36%)<br>Grade 3 IVH n=7 (58.3%)<br>Grade 4 IVH n=6(100%)<br>X^2 linear trend=6.8; P=0.009<br><br>**Wide range achievements test score <1 SD from the mean, n (%)**<br>**Low reading**<br>No IVH n=42 (24.4%)<br>Grade 1 IVH n=6 (13.3%)<br>Grade 2 IVH n=5 (20.8%)<br>Grade 3 IVH n=2 (18.2%)<br>Grade 4 IVH n=3 (75%)<br>X^2 linear trend=0.1; p=0.77<br><br>**Low spelling**<br>No IVH n=33 (19.2%)<br>Grade 1 IVH n=6 (13.6%)<br>Grade 2 IVH n=2 (8.3%)<br>Grade 3 IVH n=3 (27.3%)<br>Grade 4 IVH n=3 (75%)<br>X^2 linear trend=0.7; p=0.39<br><br>**Low arithmetic**<br>No IVH n=47 (27.6%)<br>Grade 1 IVH n=9 (20.5%)<br>Grade 2 IVH n=2 (8.3%)<br>Grade 3 IVH n=3 (27.3%)<br>Grade 4 IVH n=4 (100%)<br>X^2 linear trend=0.1; p=0.79
23	Tymofiyeva 2018[39]<br><br>USA<br><br>Prospective cohort	**Population (n=24)**<br>• Gestation < 33 weeks<br><br>**Exposure**<br>• Mild WMI (n=4)<br>• Moderate WMI (n=5)<br>• Severe WMI (n=1)<br><br>• IVH grade 1 (n=5)<br>• IVH grade 2 (n=0)<br>• IVH grade 3 (n=0)<br>• IVH grade 4 (n=0)<br><br>**Comparator**<br>• Unmatched<br>• No WMI (n=14)<br>• No IVH (n=19)<br><br>**Ascertainment/ definition**<br>• MRI imaging reviewed by a blinded paediatric neuroradiologist<br>• Used own classification of white matter injury<br>• Papile classification	**Outcome**<br>• Cognitive<br>• Behaviour<br><br>**Assessment/ measurement**<br>• Test of variables of attention<br>• Conners comprehensive behaviour rating scales<br>• CBCL<br>• Assessment undertaken by a blinded psychologist<br>• Parental questionnaire<br><br>**Follow-up**<br>• 10-14 years<br>• Completeness not specified	**Attention (abnormal)**<br>Mild WMI n=3, 75%<br>Moderate WMI n=0, 0%<br>No WMI n=8, 57% p=0.05

Confidential: For Review Only

24	Van de Bor 2004[21]<br><br>Netherlands<br><br>Prospective cohort	**Population**<br>• Gestation < 32 weeks<br>• Birthweight < 1500 g<br>• Born 1983<br><br>**Exposure**<br>• IVH grade 1-2 (n=45)<br>• IVH grade 3-4 (n=17)<br><br>**Comparator (n=216)**<br>• Unmatched<br>• No IVH<br><br>**Ascertainment/ definition**<br>• Ultrasound diagnosis<br>• Papile classification	**Outcomes**<br>• Disability (composite)<br>• Cognitive<br>• Neurological status (motor)<br>• Speech and language<br>• Behaviour<br>• Hearing<br>• Vision<br>•<br>**Measurement/assessment**<br>• Questionnaires (completed by parents at 9 years; adolescents at 14 years)<br>• Home visit and neurodevelopmental assessment by paediatrician unaware of medical history<br>• WHO classification of impairment, disability, and handicap<br><br>**Follow-up**<br>• 5, 9 and 14 years<br>91.5% follow-up of survivors at 14 years	**Disability at 5 years**<br>No IVH n=49 (23%)<br>IVH grade 3-4 n=5 (31.3%)<br><br>**Cognitive disability**<br>No IVH n=18 (8.3%)<br>IVH grade 3-4 n=1 (5.9%) p=not significant<br><br>**Motor disability**<br>No IVH n=8 (3.7%)<br>IVH grade 3-4 n=3 (17.6%) p=0.00<br><br>**Speech/language disability**<br>No IVH n=34 (15.7%)<br>IVH grade 3-4 n=1 (5.9%) p= not significant<br><br>**Visual disability**<br>No IVH n=1 (0.5%)<br>IVH grade 3-4 n=0 p= not significant<br><br>**Hearing disability**<br>No IVH n=5 (2.3%)<br>IVH grade 3-4 n=0 p= not significant<br><br>**School performance at 5 years**<br>**Special education**<br>No IVH n=17 (8.7%)<br>IVH grade 3-4 n=3 (20%) p=0.02<br><br>**School performance at 9 years**<br>**Slow learner**<br>No IVH n=57 (29.5%)<br>IVH grade 3-4 n=4 (26.7%)<br><br>**Special education**<br>No IVH n=29 (15%)<br>IVH grade 3-4 n=4 (26.7%) p=0.04<br><br>**School performance at 14 years**<br>**Slow learner**<br>No IVH n=93 (44.1)<br>IVH grade 3-4 n=4 (23.5%)<br><br>**Special education**<br>No IVH n=26 (12%)<br>IVH grade 3-4 n=6 (35.3%) p=0.00<br><br>**Need for special education at 14 years**<br>IVH (all grades)<br>OR 2.56 95%CI (1.17-4.86)<br>aOR 2.33 95%CI (1.15, 4.75)<br><br>IVH grade 3-4<br>aOR 3.99 95%CI (1.36, 11.69)

Confidential: For Review Only

25	Van Den Hout 2000[32]<br><br>Netherlands<br><br>Prospective cohort	**Population**<br>• Mean gestation 28-30 weeks<br>• Born 1989-1991<br><br>**Exposure**<br>• IVH (n=17)<br>• PVL (n=12)<br><br>**Comparator (n=17)**<br>• Preterm<br>• Normal cranial ultrasound<br><br>**Ascertainment/ definition**<br>• Ultrasound diagnosis<br>• Modified Levene and DeVries classification for IVH<br>• DeVries classification for PVL	**Outcomes**<br>• Cognitive<br>• Visual acuity<br><br>**Measurement/ assessment**<br>• L94 visual-perceptual ability test<br>• Grating acuity cards<br>• McCarthy scales of children's abilities<br>• Wechsler preschool and primary scale of intelligence<br>• Snijders-Oomen non-verbal intelligence test<br>• Leiden Diagnostic test<br><br>**Follow-up**<br>• Mean 5.3 years<br>• 88% follow-up	**Total intelligence quotient, mean (SD)**<br>IVH 92.4 (16.3)<br>PVL 79.6 (20.5)<br>No brain injury 102.8 (14.4)<br><br>**IQ <85**<br>IVH n=6, 35.3%<br>PVL n=6, 50%<br>No brain injury n=2, 11.8%<br><br>**Performance age in years, mean (SD)**<br>IVH 5.22 (1.16)<br>PVL 4.37 (1.19)<br>No brain injury 6.22 (0.89)<br><br>**Visual grating acuity in c/deg, mean (SD)**<br>IVH 37.4 (13.5)<br>PVL 33.5 (15.9)<br>No brain injury 47.1 (13.5)<br><br>**Visual grating acuity <25c/deg (%)**<br>IVH (11.8)<br>PVL (33.3)<br>No brain injury (0)<br><br>**Impairment on each of the eight L94 tasks**<br>**Visual matching % (n)**<br>IVH 0 (17)<br>PVL 0 (12)<br>No brain injury 5.9 (17)<br><br>**Unconventional Object Views % (n)**<br>IVH 29.4 (17)<br>PVL 41.7 (12)<br>No brain injury 17.6 (17)<br><br>**De Vos task % (n)**<br>IVH 29.4 (17)<br>PVL 41.7 (12)<br>No brain injury 11.8 (17)<br><br>**Line Drawings Occluded by Noise% (n)**<br>IVH 6.3 (16)<br>PVL 36.4 (11)<br>No brain injury 0 (17)<br><br>**Line Drawings Occluded by Noise% (n)**<br>IVH 13.3 (15)<br>PVL 25.0 (8)<br>No brain injury 5.9 (17)<br><br>**Developmental test of visual motor integration % (n)**<br>IVH 0 (16)<br>PVL 0 (7)<br>No brain injury 0 (17)<br><br>**Matching block designs % (n)**<br>IVH 5.9 (17)<br>PVL 20.0 (10)<br>No brain injury 17.6 (17)<br><br>**Constructing block designs% (n)**<br>IVH 30.8 (13)<br>PVL 80.0 (5)<br>No brain injury 31.3 (16)<br><br>**Mean percentage of L94 tasks on which child is impaired (mean, SD; %)**<br>IVH 14.71 (17.81)<br>PVL 32.04 (24.64)<br>No brain injury 11.13 (9.79)

Confidential: For Review Only

26<br>*	Vollmer 2003[22]<br><br>UK<br><br>Prospective cohort	**Population**<br>• Gestation <33 weeks<br>• Born 1983-1988<br><br>**Exposure**<br>• IVH (n=159)<br>• Ventricular dilatation (n=32)<br>• IVH, PV flare, ventricular dilatation (n=164)<br>• Hydrocephalus (n=36)<br>• Haemorrhagic parenchymal infarction (HPI) (n=61)<br>• cPVL n=26<br><br>**Comparator (n=348)**<br>• Unmatched<br>• Normal scan<br><br>**Ascertainment/ definition**<br>• Ultrasound imaging reviewed by two experienced observers<br>• In-house classification used	**Outcomes**<br>• Neurodevelopmental impairment (composite)<br>• Visual impairment<br>• Hearing impairment<br><br>**Measurement/ assessment**<br>• Structured neurologic examination<br>• Pure-tone audiogram<br>• Vision test (Snellen chart)<br>• Henderson-Stott TOMI<br>• Beery test of VMI<br>• WISC-R for children born 1983-1986<br>• WISC-III for children born 1987-1988<br><br>**Follow-up**<br>• 8 years<br>• 91.7% follow-up	**Neurodevelopmental status**<br>**Group A (<28 weeks)**<br>**All impairments (n,%)**<br>GMH/IVH (5, 18%)<br>Ventricular dilatation (4, 50%)<br>GMH/IVH, flare, ventricular dilatation (19, 51%)<br>Hydrocephalus (7, 78%)<br>HPI (15, 100%)<br>cPVL (4, 100%)<br>No brain injury (12, 32%)<br><br>**Disabling impairments (n, %)**<br>GMH/IVH (1, 4%)<br>Ventricular dilatation (0, 0%)<br>GMH/IVH, flare, ventricular dilatation (9, 24%)<br>Hydrocephalus (7, 78%)<br>HPI (14, 93%)<br>cPVL (3, 75%)<br>No brain injury (3, 8%)<br><br>**Group B (28-32 weeks)**<br>**All impairments (n, %)**<br>GMH/IVH (16, 29%)<br>Ventricular dilatation (5, 31%)<br>GMH/IVH, flare, ventricular dilatation (30, 43%)<br>Hydrocephalus (7, 54%)<br>HPI (5, 83%)<br>cPVL (9, 75%)<br>No brain injury (67, 29%)<br><br>**Disabling impairments (n, %)**<br>GMH/IVH (5, 5%)<br>Ventricular dilatation (1, 6%)<br>GMH/IVH, flare, ventricular dilatation (16, 23%)<br>Hydrocephalus (6, 46%)<br>HPI (3, 50%)<br>cPVL (6, 50%)<br>No brain injury (14, 6%)

Confidential: For Review Only

27<br>*	Vollmer 2006a[27]<br><br>UK<br><br>Prospective cohort	**Population**<br>• Gestation <33 weeks<br>• Born 1985-1991<br><br>**Exposure**<br>• Bilateral brain lesions (n=201)<br>• Right-sided brain lesion (n=41)<br>• Left-sided brain lesion (n=57)<br><br>Brain lesion types<br>Non-parenchymal:<br>• Uncomplicated IVH<br>Parenchymal:<br>• Haemorrhagic parenchymal infarction (HPI)<br>• cPVL<br>• PV flare<br><br>**Comparator (n=369)**<br>• Unmatched<br>• Normal ultrasound<br><br>**Ascertainment/ definition**<br>• Ultrasound imaging reviewed by two experienced observers<br>• Modified Stewart classification	**Outcomes**<br>• Motor<br>• Cognitive<br>• Cerebral palsy<br>• Visual<br><br>**Measurement/ assessment**<br>• Neurological examination (modified Amiel-Tison assessment)<br>• TOMI<br>• WISC-R<br>• Test of VMI<br><br>**Follow-up**<br>• 8 years<br>• 80% follow-up	**TOMI error score, mean (SD)**<br>Normal scan 2.78 (2.1)<br><br>All left-sided lesions 4.3 (3.5)<br>Left-sided non-parenchymal lesions 4.5 (3.8)<br>Left-sided parenchymal lesions 3.7 (2.1)<br><br>All right-sided lesions 3.5 (2.9)<br>Right-sided non-parenchymal lesions 2.7 (1.8)<br>Right-sided parenchymal lesions 4.9 (3.8)<br><br>All bilateral lesions 4.5 (4.3)<br>Bilateral non-parenchymal lesions 4.1 (3.7)<br>Bilateral parenchymal lesions 4.9 (4.7)<br><br>ANOVA for parenchymal lesions only p <0.0001<br>ANOVA including parenchymal and non-parenchymal lesions p <0.0001<br>ANOVA excluding parenchymal lesions, p <0.0001<br><br>**VMI centile, mean (SD)**<br>Normal scan 59.2 (30.0)<br><br>All left-sided lesions 40.3 (30.1)<br>Left-sided non-parenchymal lesions 46.8 (31.0)<br>Left-sided parenchymal lesions 21 (22)<br><br>All right-sided lesions 60.2 (31.9)<br>Right-sided non-parenchymal lesions 64.2 (30.2)<br>Right-sided parenchymal lesions 54 (35)<br><br>All bilateral lesions 46.0 (33.5)<br>Bilateral non-parenchymal lesions 55.1 (32.1)<br>Bilateral parenchymal lesions 38 (32)<br><br>ANOVA for parenchymal lesions only p <0.0001<br>ANOVA including parenchymal and non-parenchymal lesions p <0.0001<br>ANOVA excluding parenchymal lesions reported as both p <0.0001 and p=0.98 Ω(potential error in the manuscript table)<br><br>**Cerebral palsy, n (%)**<br>Normal scan 2 (0.7%)<br><br>All left-sided lesions 4 (9%)<br>Left-sided non-parenchymal lesions 2 (6%)<br>Left-sided parenchymal lesions 2 (16%)<br><br>All right-sided lesions 2 (6%)<br>Right-sided non-parenchymal lesions 1 (4%)<br>Right-sided parenchymal lesions 1 (8%)<br><br>All bilateral lesions 37 (21%)<br>Bilateral non-parenchymal lesions 8 (10%)<br>Bilateral parenchymal lesions 29 (31%)<br><br>Chi-square for parenchymal and non-parenchymal lesions, p <0.0001<br>Chi-square excluding parenchymal lesions, p <0.0001<br>Chi-square for parenchymal lesions only, p <0.0001<br>ANOVA parenchymal lesions only, p <0.0001<br><br>**Full scale IQ, mean (SD)**<br><br>Normal scan 101 (16)<br><br>All left-sided lesions 93 (17)<br>Left-sided non-parenchymal lesions 98 (15)<br>Left-sided parenchymal lesions 80 (15)<br><br>All right-sided lesions 102 (17)<br>Right-sided non-parenchymal lesions 104 (15)<br>Right-sided parenchymal lesions 100 (19)<br><br>All bilateral lesions 91 (21)<br>Bilateral non-parenchymal lesions 96(19)<br>Bilateral parenchymal lesions 86 (22)<br><br>ANOVA for parenchymal lesions only, p <0.0001.<br>ANOVA including parenchymal and non-parenchymal lesions, p <0.0001.<br>ANOVA excluding parenchymal lesions, p =0.137.<br><br>**Verbal IQ, mean (SD)**<br>Normal scan 103 (19)<br><br>All left-sided lesions 98 (20)<br>Left-sided non-parenchymal lesions 102 (20)<br>Left-sided parenchymal lesions 85 (18)<br><br>All right-sided lesions 107 (18)<br>Right-sided non-parenchymal lesions 108 (16)<br>Right-sided parenchymal lesions 107 (22)<br><br>All bilateral lesions 96 (23)<br>Bilateral non-parenchymal lesions 100 (20)<br>Bilateral parenchymal lesions 91 (25)<br><br>ANOVA for parenchymal lesions only, p <0.0001<br>ANOVA including parenchymal and non-parenchymal lesions, p <0.0001<br>ANOVA excluding parenchymal lesions, p =0.38

Confidential: For Review Only

				**Performance IQ, mean (SD)**<br>Normal scan 96 (15)<br><br>All left-sided lesions 86 (16)<br>Left-sided non-parenchymal lesions 90 (15)<br>Left-sided parenchymal lesions 76 (15)<br><br>All right-sided lesions 95 (16)<br>Right-sided non-parenchymal lesions 98 (13)<br>Right-sided parenchymal lesions 92 (19)<br><br>All bilateral lesions 85 (22)<br>Bilateral non-parenchymal lesions 91 (20)<br>Bilateral parenchymal lesions 80 (21)<br><br>ANOVA for parenchymal lesions only, p <0.0001<br>ANOVA including parenchymal and non-parenchymal lesions, p <0.0001<br>ANOVA excluding parenchymal lesions, p =0.59
28<br>*	Vollmer 2006b[33]<br><br>UK<br><br>Prospective cohort	**Population**<br>• Gestation <33 weeks<br>• Born 1979-1991<br><br>**Exposure (n=66)**<br>• Ventricular dilatation and IVH<br><br>**Comparator (n=616)**<br>• Unmatched<br>• Normal cranial ultrasound<br><br>**Ascertainment/ definition**<br>• Ultrasound imaging reviewed by two experienced observers<br>• In-house classification used	**Outcomes**<br>• Neurological impairment with or without disability (composite)<br>• Cognitive<br>• Motor<br>• Vision<br><br>**Measurement/ assessment**<br>• Structured neurological exam<br>• TOMI<br>• Test of VMI<br>• WISC<br><br>**Follow-up**<br>• 8 years<br>• 81% follow-up	**Disabling motor impairment, n (%)**<br>Ventricular dilatation and IVH n=10 (16%)<br>Normal ultrasound n=10 (2%)<br><br>**Cognitive**<br>**Full scale IQ, mean (SD)**<br>Ventricular dilatation and IVH 96 (23)<br>Normal ultrasound 101 (17)<br><br>**Verbal IQ, mean (SD)**<br>Ventricular dilatation and IVH 101 (22)<br>Normal ultrasound 104 (19)<br><br>**Performance IQ mean (SD)**<br>Ventricular dilatation and IVH 97 (15)<br>Normal ultrasound 91 (21)<br><br>**Motor and vision**<br>**VMI centile, mean (SD)**<br>Ventricular dilatation and IVH 37 (33)<br>Normal ultrasound 52 (31)<br><br>**TOMI, mean (SD)**<br>Ventricular dilatation and IVH 5.98 (4.2)<br>Normal ultrasound 3.26 (2.5)

Confidential: For Review Only

29	Whitaker 2011[36]<br><br>USA<br><br>Prospective cohort	**Population**<br>• Birthweight <2000g<br>• 'Non-disabled' survivors<br>• Born 1984-1987<br><br>**Exposure**<br>• IVH (n=69)<br>• Parenchymal lesions and/or ventricular enlargement (n=21)<br><br>**Comparison (n=368)**<br>• Unmatched<br>• Normal cranial ultrasound<br><br>**Ascertainment/ definition**<br>• Ultrasound imaging reviewed by three blinded radiologists independently, disagreements resolved through consensus and inter-observer reliability checked.<br>• Paneth classification	**Outcomes**<br>• Mental health conditions<br><br>**Measurement/ assessment**<br>• Parent report version of the Diagnostic Interview Schedule for Children–IV<br>• WASI<br><br>**Follow-up**<br>• 16 years<br>• 72.9% follow-up	**Logistic regression assessing odds of current and lifetime mental health conditions after brain injury**<br><br>**Current ADHD- inattentive type**<br>**IVH**<br>OR 0.97 95% CI (0.21-4.47)<br>aOR 1.01 95% CI (0.19-5.44)<br><br>**Parenchymal lesions and/or ventricular enlargement**<br>OR 7.64[b] 95% CI (2.20-24.48)<br>aOR 6.83[c] 95% CI (1.26-36.91)<br><br>**Lifetime ADHD – inattentive type**<br>**IVH**<br>OR 0.83 95% CI (0.34-2.04)<br>aOR 0.64 95% CI (0.24-1.74)<br><br>**Parenchymal lesions and/or ventricular enlargement**<br>OR 2.71 95% CI (0.94-7.82)<br>aOR 1.13 95% CI (0.31-4.10)<br><br>**Current major depression**<br>**IVH**<br>OR 2.66 95% CI (1.04-6.78)<br>aOR 2.23 95% CI (0.80-6.24)<br><br>**Lifetime major depression**<br>**IVH**<br>OR 2.76 95% CI (1.19-6.38)<br>aOR 2.59 95% CI (1.02-6.58)<br><br>**Current tic disorders**<br>**IVH**<br>OR 1.63 95% CI (0.44-6.07)<br>aOR 1.89 95% CI (0.42-8.57)<br><br>**Parenchymal lesions and/or ventricular enlargement**<br>OR 8.42 95% CI (2.40-29.62)<br>aOR 9.77 95% CI (1.69-56.47)<br><br>**Lifetime tic disorders**<br>**IVH**<br>OR 0.95 95% CI (0.27-3.34)<br>aOR 0.85 95% CI (0.21-3.51)<br><br>**Parenchymal lesions and/or ventricular enlargement**<br>OR 5.07 95% CI (1.53-16.82)<br>aOR 5.02 95% CI (1.05-23.92)<br><br>**Current obsessive-compulsive disorder**<br>**IVH**<br>OR 9.52 95% CI (3.02-30.06)<br>aOR 11.85 95% CI (3.22-43.62)<br><br>**Parenchymal lesions and/or ventricular enlargement**<br>OR 7.64 95% CI (1.39-41.98)<br>aOR 15.32 95% CI (1.82-128.74)<br><br>**Lifetime obsessive compulsive disorder**<br>**IVH**<br>OR 9.52 95% CI (3.05-30.06)<br>aOR 11.85 95% CI (3.22-43.62)<br><br>**Parenchymal lesions and/or ventricular enlargement**<br>OR 7.64 95% CI (1.39-41.98)<br>aOR 15.32 95% CI (1.82-128.74)<br><br>**Current diagnoses additionally controlled for full score IQ and motor function**<br><br>**ADHD inattentive type**<br>**IVH**<br>OR 0.86 95% CI (0.18-3.99)<br>aOR 0.99 95% CI (0.21-4.62)<br><br>**Parenchymal lesions and/or ventricular enlargement**<br>OR 5.04 95% CI (1.36-18.65)<br>aOR 5.43 95% CI (1.32-22.40)<br><br>**Major depression**<br>**IVH**<br>OR 0.43 95% CI (0.16-1.11)<br>aOR 0.40 95% CI (0.15-1.05)<br><br>**Tic disorders**<br>**IVH**<br>OR 1.54 95% CI (0.41-5.78)<br>aOR 1.45 95% CI (0.38-5.48)<br><br>**Parenchymal lesions and/or ventricular enlargement**<br>OR 7.01 95% CI (1.88-28.14)<br>aOR 4.38 95% CI (1.05-18.23)<br><br>**Obsessive compulsive disorder**<br>**IVH**<br>OR 8.68 95% CI (2.72-27.69)<br>aOR 10.91 95% CI (3.13-37.99)<br><br>**Parenchymal lesions and/or ventricular enlargement**<br>OR 4.78 95% CI (0.83-28.10)<br>aOR 3.58 95% CI (0.50-25.94)

Confidential: For Review Only

Perinatal stroke				
30	Ballantyne * 2007 [47]<br>USA<br><br>Prospective cohort	**Population**<br>• Mean gestation 38.5 weeks<br>• Born 1991-2001<br><br>**Exposure (n=28)**<br>• Left lesions (n=17)<br>• Right lesions (n=11)<br><br>**Comparator (n=57)**<br>• Unmatched<br>• Healthy controls with normal medical and developmental histories<br>• Recruited from the community<br><br>**Ascertainment/ definition**<br>• Single unilateral lesions the result of perinatal strokes occurring between 28 weeks' gestation and 28 days after birth; infarct or haemorrhage<br>• Identified through medical history and neuroimaging<br>• Severity rated on a 5-point scale adapted from the Vargha-Khadem classification	**Outcomes**<br>• Speech and language<br><br>**Assessment/ measurement**<br>• CELF-R<br>• Wechsler Intelligence Scales (WPPSI-R, WISC-R, or WISC-III)<br>• PPVT–Revised<br>• Expressive One-Word Picture Vocabulary Test–Revised or Upper-Extension<br>• Total Language Standard Scores<br><br>**Follow-up**<br>• 6-9 years<br>• 100% follow-up	**Speech and language**<br>**CELF-R Receptive, mean (SD)**<br>All strokes: 82.54 (17.12) p<.0001<br>Left stroke: 83.18 (16.66) p<.0001<br>Right stroke: 81.55 (18.59) p=0.001<br>Control: 106.37 (12.51)<br><br>**CELF-R Expressive mean (SD)**<br>All strokes: 73.75 (16.79) p<.0001<br>Left stroke: 73.06 (14.88) p<.0001<br>Right stroke: 74.82 (20.11) p=0.001<br>Control: 101.02 (13.63)<br><br>**CELF-R Total mean (SD)**<br>All strokes: 76.93 (17.31) p<.0001<br>Left stroke: 76.94 (15.39) p<.0001<br>Right stroke: 76.91 (20.74) p=0.001<br>Control: 104.00 (12.58)<br>.

Confidential: For Review Only

31	Ballantyne 2008[40] *<br><br>USA<br><br>Prospective cohort	**Population**<br>• 32- 40 weeks' gestation<br>• Birth years not reported<br><br>**Exposure (n=29)**<br>• Left hemisphere (n=20)<br>• Right hemisphere (n=9)<br><br>**Control (n=38)**<br>• Healthy controls (normal neurodevelopment)<br>• Recruited through a university and community adverts<br><br>**Ascertainment/ definition**<br>• Unilateral ischaemic perinatal stroke confirmed through clinical history and neuroimaging<br>• Lesion location and severity reviewed by blinded neuroradiologist<br>• Severity rated on a 5-point scale adapted from the Vargha-Khadem classification	**Outcomes**<br>• Cognitive (academic skills)<br>• Speech and language<br>• Motor<br>• Cerebral palsy<br>• Vision<br>• Epilepsy<br><br>**Measurement/ assessment**<br>• WISC- Revised<br>• WRAT- Revised<br>• CELF- Revised<br>• PPVT-Revised<br>• WPPSI/WPPSI- Revised<br>• WISC-III<br><br>**Follow-up**<br>• 7-12 years<br>• 100% follow up	**Hemiparesis**<br>Stroke n=18,62%<br><br>**Visual field deficit**<br>Stroke n=7, 26%<br><br>**Seizures**<br>Stroke n=11, 38%<br><br>**Cognitive, mean (SD)**<br>**Verbal IQ (WISC-R)**<br>**Time point 1 (mean age 7-8 years)**<br>Stroke 96.6 (20.5)<br>Control 126.1 (16)<br><br>**Time point 2 (mean age 10 – 12 years)**<br>Stroke 98.7 (20)<br>Control 123.6 (13.1)<br>Between group affect (stroke vs. control) p<0.0001<br>Time effect not significant<br><br>**Performance IQ (WISC-R)**<br>**Time point 1 (mean age 7-8 years)**<br>Stroke 92.8 (19.9)<br>Control 115.2 (13.8)<br><br>**Time point 2 (mean age 10 – 12 years)**<br>Stroke 93.5 (20)<br>Control 116 (10.5)<br>Between group affect (stroke vs. control) p=0.002<br>Time effect not significant<br><br>**Full scale IQ (WISC-R)**<br>**Time point 1 (mean age 7-8 years)**<br>Stroke 94.7 (20.4)<br>Control 123 (15)<br><br>**Time point 2 (mean age 10 – 12 years)**<br>Stroke 96.1 (19.1)<br>Control 122.3 (10.2)<br><br>Between group affect (stroke vs. control) p<0.0001<br>Time effect not significant<br><br>**Reading (WRAT -R)**<br>**Time point 1 (mean age 7-8 years)**<br>Stroke 85 (16.1)<br>Control 113 (13.3)<br><br>**Time point 2 (mean age 10 – 12 years)**<br>Stroke 89.4 (13.3)<br>Control 108.9 (13.8)<br><br>Between group affect (stroke vs. control) p<0.0001<br>Time effect not significant<br>Time group interaction p=0.045<br><br>**Spelling (WRAT -R)**<br>**Time point 1 (mean age 7-8 years)**<br>Stroke 82.5 (18.2)<br>Control 106.2 (15.9)<br><br>**Time point 2 (mean age 10 – 12 years)**<br>Stroke 87 (16.8)<br>Control 104.6 (13.1)<br><br>Between group affect (stroke vs. control) p=0.001<br>Time effect not significant<br><br>**Arithmetic (WRAT -R)**<br>**Time point 1 (mean age 7-8 years)**<br>Stroke 91.5 (10.2)<br>Control 111.9 (11.2)<br><br>**Time point 2 (mean age 10 – 12 years)**<br>Stroke 94.2 (18.7)<br>Control 113.1 (16.2)<br><br>Between group affect (stroke vs. control) p<0.0001<br>Time effect not significant<br><br>**Speech and language**<br>**Receptive language score**<br>**Time point 1 (mean age 7-8 years)**<br>Stroke 84.2 (10.9)<br>Control 109.1 (12.2)<br><br>**Time point 2 (mean age 10 – 12 years)**<br>Stroke 82.3 (20.1)<br>Control 111.4 (13.7)<br><br>Between group affect (stroke vs. control) p<0.0001<br>Time effect not significant<br><br>**Expressive language score**<br>**Time point 1 (mean age 7-8 years)**<br>Stroke 72.5 (12)<br>Control 101 (17.5)<br><br>**Time point 2 (mean age 10 – 12 years)**<br>Stroke 78.4 (16)

Confidential: For Review Only

				Control 105.8 (11.9)<br><br>Between group affect (stroke vs. control) p<0.0001<br>Time effect p=0.017<br><br>**Total language score**<br>**Time point 1 (mean age 7-8 years)**<br>Stroke 76.9 (11.1)<br>Control 105.6 (14.2)<br><br>**Time point 2 (mean age 10 – 12 years)**<br>Stroke 79.1 (18.3)<br>Control 109.8 (14)<br><br>Between group affect (stroke vs. control) p<0.0001<br>Time effect not significant<br><br>**Vocabulary score**<br>**Time point 1 (mean age 7-8 years)**<br>Stroke 97.5 (19.7)<br>Control 117.1 (17)<br><br>**Time point 2 (mean age 10 – 12 years)**<br>Stroke 99.9 (20)<br>Control 118.9 (13.9)<br><br>Between group affect (stroke vs. control) p=0.002<br>Time effect not significant

Confidential: For Review Only

32	Gold 2014[41]<br><br>USA<br><br>Prospective cohort	**Population**<br>• Gestation not provided<br>• Birth years not provided<br><br>**Exposure (n=27)**<br>• Right-sided stroke (n=12)<br>• Left-sided stroke (n=15)<br><br>**Comparator (n=19)**<br>• Matched for age at follow up, sex, socioeconomic group and maternal education<br>• Healthy controls<br>• Recruited through local advertising<br><br>**Ascertainment/ definition**<br>• Single, unilateral brain lesion in an arterial vascular distribution, either identified in the neonatal period with neuroimaging, or identified later in infancy after presentation with a hemiparesis and imaging documentation of an old unilateral infarct (presumed perinatal stroke)<br>• Recruited from paediatric neurology clinics<br>• Severity graded 1-5 using Trauner/ Vargha-Khaldem classification	**Outcomes**<br>• Cognitive (IQ and memory)<br>• Motor<br>• Cerebral palsy<br><br>**Measurement/ assessment**<br>• WISC-III<br>• Dots and Stories subtests of the Children's Memory Scales<br><br>**Follow-up**<br>• 6-16 years<br>• 100% follow-up	**Cognitive**<br>**Memory**<br>**Stories immediate recall**<br>Controls, mean (SE)13.5 (0.7)<br>Stroke, mean (SE) 8.4 (0.8) p<0.001<br><br>Stroke and seizures, mean (SE)7 (0.8)<br>Stroke and no seizures, mean (SE) 10.1 (1.4) p=0.06<br><br>Right lesion, mean (SE) 7.8 (1.1)<br>Left lesion, mean (SE) 8.9 (1.2) p=0.51<br><br>**Delayed recall**<br>Controls, mean (SE) 13.9 (0.8)<br>Stroke, mean (SE) 7.9 (0.8) p<0.001<br><br>Stroke and seizures, mean (SE) 6.2 (0.9)<br>Stroke and no seizures, mean (SE) 10 (1.2) p=0.02<br><br>Right lesion, mean (SE) 7.3 (1.1)<br>Left lesion, mean (SE) 8.3 (1.2) p=0.56<br><br>**Delayed recognition**<br>Controls, mean (SE) 11.5 (0.5)<br>Stroke, mean (SE) 8 (0.8) p=0.001<br><br>Stroke and seizures, mean (SE) 7.1 (1.1)<br>Stroke and no seizures, mean (SE) 9.2 (0.9) p=0.17<br><br>Right lesion, mean (SE) 8.3 (1.4)<br>Left lesion, mean (SE) 7.9 (0.9) p=0.8<br><br>**Dots learning**<br>Controls, mean (SE) 10.9 (0.5)<br>Stroke, mean (SE) 8.9 (0.8) p=0.05<br><br>Stroke and seizures, mean (SE) 7.6 (1.1)<br>Stroke and no seizures, mean (SE) 10.6 (0.8) p=0.05<br><br>Right lesion, mean (SE) 9.3 (1.4)<br>Left lesion, mean (SE) 8.7 (0.9) p=0.71<br><br>**Total**<br>Controls, mean (SE) 11.8 (0.5)<br>Stroke, mean (SE) 9 (0.7) p=0.003<br><br>Stroke and seizures, mean (SE) 7.8 (0.9)<br>Stroke and no seizures, mean (SE) 10.6 (0.9) p=0.04<br><br>Right lesion, mean (SE) 9.2 (0.7)<br>Left lesion, mean (SE) 10.2 (0.7) p=0.62<br><br>**Delayed recall**<br>Controls, mean (SE) 12.6 (0.4)<br>Stroke, mean (SE) 10 (0.5) p<0.001<br><br>Stroke and seizures, mean (SE) 8.8 (0.5)<br>Stroke and no seizures, mean (SE) 11.4 (0.8) p=0.009<br><br>Right lesion, mean (SE) 9.7 (0.7)<br>Left lesion, mean (SE) 10.2 (0.7) p=0.62<br><br>**WISC- III IQ, mean (SD)**<br>Right stroke, 85.0 (6)<br>Left stroke, 91 (6) p=0.49<br><br>**IQ scores**<br>Controls 117 (2.7)<br>All stroke patients 88 (4.0) p<0.001<br>No seizures 100 (6.4)<br>Seizures 78 (3.7)<br><br>**Motor (hemiparesis)**<br>Stroke patients n=16; 59%<br>Control n=0; p=0.05

Confidential: For Review Only

33	Kolk 2011[42]<br><br>Estonia<br><br>Retrospective cohort	**Population**<br>• Gestation not provided<br>• Born 1995-2006<br><br>**Exposed (n=21)**<br>• Neonatal stroke<br><br>**Control (n=31)**<br>• Matched on age and sex<br>• Healthy children<br>• Recruited locally<br><br>**Ascertainment/ definition**<br>• Estonian stroke registry<br>• Arterial ischaemic stroke or haemorrhagic	**Outcomes**<br>• Cognitive<br>• Neuropsychological<br>• Motor<br>• Cerebral palsy<br>• Speech and language<br>• Epilepsy<br><br>**Measurement/ assessment**<br>• NEPSY<br>• Kaufman ABC<br>• Paediatric Stroke Outcome Measure<br><br>**Follow-up**<br>• 4-10 years<br>• 100% follow-up	**Neuromotor impairment (Paediatric Stroke Outcome Measure)**<br>**Neonatal stroke**<br>Severe n=4, 19%<br>Moderate n=9, 43%<br>Good n=6, 28.6%<br>Normal n=2, 9.5%<br><br>**Cognitive/ neuropsychological**<br><br>**Attention and executive function, mean, SD, 95% CI**<br>**Tower**<br>Control 0.22, 0.64 (-0.05, 0.48)<br>Neonatal stroke -0.34, 1.34 (-1.03, 0.35) p=0.142<br><br>**Auditory attention**<br>Control 0.27, 0.72 (-0.03, 0.57)<br>Neonatal stroke -0.38, 1.10 (-1.04, 0.28) p=0.009<br><br>**Visual attention: time**<br>Control 0.37, 0.81, (0.07, 0.67)<br>Neonatal stroke -0.40, 0.93 (-0.82, 0.03) p=0.004<br><br>**Visual attention: correct**<br>Control 0.48, 0.50 (0.30, 0.67)<br>Neonatal stroke -0.54, 0.97 (0.98, 0.1) p<0.0001<br><br>**Statue**<br>Control 0.26, 0.77 (-0.03, 0.54)<br>Neonatal stroke -0.23, 1.09, (-0.73, 0.28) p=0.086<br><br>**Design fluency**<br>Control 0.18, 1.04 (-0.25, 0.61)<br>Neonatal stroke -0.36, 0.70 (-0.78, 0.06) p=0.06<br><br>**Knock and tap**<br>Control 0.31, 0.50 (0.10, 0.51)<br>Neonatal stroke -0.44, 1.52, (-1.32, 0.43) p==0.03<br><br>**Language, mean, SD, 95% CI**<br>**Phonological processing**<br>Control 0.24, 0.80 (-0.05, 0.54)<br>Neonatal stroke -0.38, 0.99 (-0.83, 0.08) p=0.001<br><br>**Comprehension of instructions**<br>Control 0.43, 0.70 (0.18, 0.69)<br>Neonatal stroke -0.59 1.06 (-1.07, 0.11) p<0.0001<br><br>**Speeded naming: time**<br>Control 0.24, 0.70 (-0.05, 0.52)<br>Neonatal stroke -0.14, 1.03 (-0.73, 0.46) p=0.188<br><br>**Speeded naming: correct**<br>Control 0.42, 0.41 (0.25, 0.59)<br>Neonatal stroke -0.45, 1.41 (-1.26, 0.37) p=0.008<br><br>**Repetition of nonsense words**<br>Control 0.30, 0.53 (0.08, 0.52)<br>Neonatal stroke -0.40, 1.23 (-1.03, 0.24) p=0.026<br><br>**Verbal fluency: semantic**<br>Control 0.43, 0.81 (0.13, 0.73)<br>Neonatal stroke -0.60, 0.95 (-1.04, 0.15) p<0.0001<br><br>**Verbal fluency: phonemic**<br>Control 0.40, 0.93 (-0.12, 0.92)<br>Neonatal stroke -0.67, 0.90 (-1.42, 0.08) p=0.008<br><br>**Oromotor sequences**<br>Control 0.31, 0.64 (0.07, 0.54)<br>Neonatal stroke -0.52, 1.25 (-1.15, 0.10)<br><br>**Sentence comprehension**<br>Control 0.19, 0.78 (-0.09, 0.48)<br>Neonatal stroke -0.35, 1.09 (-0.91, 0.21) p=0.027<br><br>**Sensorimotor functions, mean, SD, 95% CI**<br>**Finger tapping**<br>Control 0.49, 0.33 (0.35, 0.62)<br>Neonatal stroke -0.53, 1.27 (-1.16, 0.10) p=0.0007<br><br>**Imitating hand positions**<br>Control 0.57, 0.68 (0.32-0.82)<br>Neonatal stroke -0.72, 0.92 (-1.14, 0.30) p<0.0001<br><br>**Visuomotor precision: time**<br>Control 0.13, 0.83 (-0.17, 0.43)<br>Neonatal stroke -0.24, 0.97 (-0.69, 0.20) p=0.145<br><br>**Visuomotor precision: mistakes**<br>Control 0.45, 0.50 (0.27, 0.64)<br>Neonatal stroke -0.42, 1.05 (-0.90, 0.05) p=0.0002<br><br>**Manual motor sequences**<br>Control 0.50, 0.62 (0.27, 0.73)<br>Neonatal stroke -0.92, 0.95 (-1.43, 0.41) p<0.0001<br><br>**Finger discrimination**<br>Control 0.53, 0.57 (0.29, 0.77)

Confidential: For Review Only

				Neonatal stroke -0.77, 1.03 (-1.30, 0.24) p<0.0001<br><br>**Visuospatial functions, mean, SD, 95% CI**<br>**Design copying**<br>Control 0.36, 0.80 (0.06, 0.65)<br>Neonatal stroke -0.54, 0.97 (-1.0, 0.09) p<0.0001<br><br>**Arrows**<br>Control 0.37, 0.79 (0.05, 0.70)<br>Neonatal stroke -0.61, 1.07 (-1.16, 0.06) p=0.0004<br><br>**Block construction**<br>Control 0.29, 0.81 (-0.01, 0.58)<br>Neonatal stroke -0.45, 1.04 (-0.92, 0.03) p=0.0003<br><br>**Route finding**<br>Control 0.25, 1.05 (-0.33, 0.83)<br>Neonatal stroke -0.66, 0.80 (-1.23, 0.09) p=0.033<br><br>**Picture perception**<br>Control 0.13, 1.00 (-0.49, 0.24)<br>Neonatal stroke -0.09, 1.03 (-0.56, 0.37) p=0.341<br><br>**Memory and learning, mean, SD, 95% CI**<br>**Memory for faces**<br>Control 0.42, 0.74 (0.11, 0.73)<br>Neonatal stroke -0.41, 1.15 (-0.96, 0.15) p=0.016<br><br>**Memory for names**<br>Control 0.15, 0.92 (-0.23. 0.53)<br>Neonatal stroke -0.31, 1.09 (-0.87, 0.25) p=0.295<br><br>**Narrative memory**<br>Control 0.26, 0.80 (-0.03, 0.55)<br>Neonatal stroke -0.22, 1.16 (-0.78, 0.34) p=0.077<br><br>**Sentence repetition**<br>Control 0.49, 0.61 (0.26, 0.71)<br>Neonatal stroke -0.64, 0.96 (-1.09, 0.19) p<0.0001<br><br>**List learning**<br>Control 0.30, 0.82 (-0.16, 0.76)<br>Neonatal stroke -0.38, 1.22 (-1.32, 0.56) p=0.151<br><br>**Picture recognition**<br>Control 0.39, 0.72 (0.10, 0.69)<br>Neonatal stroke -0.36, 1.24 (-0.98, 0.25) p=0.027<br><br>**Motor (hemiparesis)**<br>Neonatal stroke and any hemiparesis n=19, 90%<br>Mild functional impairment n=6, 29%<br>Significant functional impairment n= 8, 38%<br>Very severe functional impairment n= 4, 19%<br><br>**Epilepsy**<br>Stroke n=9, 33.3%
34	Martin 2019[46]<br>*<br><br>USA<br><br>Prospective cohort	**Population**<br>• Gestation not provided<br>• Birth years not provided<br><br>**Exposure (n=21)**<br>• Left hemisphere (n=13)<br>• Right hemisphere (n=8)<br><br>**Control (n=21)**<br>• Matched on age, sex and socioeconomic status<br>• Healthy controls<br>• Recruited from local community using adverts<br><br>**Ascertainment/ definition**<br>• Unilateral focal brain lesion (ischaemic or haemorrhagic thought to have occurred between 28 weeks' gestation and 28 days postnatally)<br>• Recruited from a neurologist in San Diego	**Outcomes**<br>• Hearing<br>• Motor (cerebral palsy)<br>• Epilepsy<br><br>**Measurement/ assessment**<br>• Auditory neglect task<br><br>**Follow-up**<br>• 6-14 years (mean 9-10 years)<br>• Completeness not specified	**Time to correct response**<br>**Left sided sound:**<br>Left stroke 1550 ms±580 ms<br>Control 1465 ms±666 ms *not significant*<br><br>Right stroke 1708 ms±951 ms<br>Control 1074 ms±514 ms* (p=0.043)<br><br>**Right sided sound**<br>Left stroke 1595 ms±553 ms<br>Control 1501 ms±720 ms *not significant*<br><br>Right stroke 2032 ms±1496 ms<br>Control 1291 ms±792 ms p=0.118<br><br>**Number of correct auditory responses**<br>**Left sided sound**<br>Left stroke 5.15±1.21<br>Control 4.62±1.26 p=0.338<br><br>Right stroke 4.25±1.67<br>Control 4.63±1.19 p=0.307<br><br>**Right sided sound**<br>Left stroke 4.31±1.18<br>Control 4.62±1.71 p=0.3<br><br>Right stroke 4.50±1.31<br>Control 5.50±0.92 p=0.05<br><br>**Seizures outside of neonatal period**<br>Stroke n=4; 19%<br><br>**Hemiparesis**<br>Stroke n=13, 70%<br><br>Right stroke n=3, 28%<br>Left stroke n=10, 77%

Confidential: For Review Only

35	Northam 2018[43]<br><br>UK<br><br>Prospective cohort	**Population**<br>• Gestation not provided<br>• Born 1991-2001<br><br>**Exposure (n=30)**<br>• Perinatal stroke<br><br>**Control (n=40)**<br>• Matched on age, sex and maternal education<br>• Term infants<br><br>**Ascertainment/ definition**<br>• Arterial or ischaemic stroke confirmed by MRI in the neonatal period	**Outcomes**<br>• Cognitive<br>• Speech and language<br>• Motor (cerebral palsy)<br><br>**Measurement/ assessment**<br>• WASI<br>• CELF<br>• Comprehensive Test of Phonological Processing<br><br>**Follow-up**<br>• 6-18 years (mean 12.4 and 13.5)<br>• 100% follow up	**Cognitive**<br>**Full scale IQ mean (SD)**<br>Stroke 99 (14)<br>Control 112 (16) p<0.0001<br><br>**Mainstream education**<br>Stroke n=28, 93%<br><br>**Receiving additional education support**<br>Stroke n=12, 40%<br><br>**Speech and language**<br>**Expressive language score, mean (SD)**<br>Stroke 95 (17)<br>Control 108 (13) p=0.001<br><br>**Receptive language score, mean (SD)**<br>Stroke 91 (16)<br>Control 104 (14) p < 0.0001<br><br>**Motor (hemiparesis)**<br>Stroke n=9, 3%
36	Tillema 2008[44]<br><br>USA<br><br>Retrospective cohort	**Population**<br>• Gestation not provided<br>• Birth years not provided<br><br>**Exposure (n=10)**<br>• Left perinatal stroke<br><br>**Control (n=10)**<br>• Matched on age, sex, and handedness<br>• Healthy<br>• Randomly drawn from a large database of children recruited for a different study of language development in healthy children<br><br>**Ascertainment/ definition**<br>• Middle cerebral artery ischaemic stroke	**Outcomes**<br>• Cognitive<br>• Epilepsy<br><br>**Measurement/ assessment**<br>• WISC-III<br>• Language activation tasks – Verb generation task whilst in an fMRI<br><br>**Follow-up**<br>• 6-16 years<br>• 100% follow up	**Focal epilepsy**<br>Stroke, n=6, 60%<br><br>**Cognitive, mean (SD)**<br>Stroke VIQ 84 (13.4)<br>Control VIQ 108 (14.2) p=0.002<br><br>Stroke FSIQ 80 (14.1)<br>Control FSIQ 108 (11.7) p=0.001
37	Trauner 2001[45]<br><br>USA<br><br>Retrospective cohort	**Population**<br>• Gestation not reported<br>• Birth years not reported<br><br>**Exposure (n=39)**<br>• Left perinatal stroke (n=25)<br>• Right perinatal stroke (n=14)<br><br>**Control (n=54)**<br>• Matched on age and socioeconomic status<br>• Normal neurodevelopmental history<br>• Identified from clinics, community adverts, schools<br><br>**Ascertainment/ definition**<br>• Pre or perinatal onset unilateral brain damage (focal lesion) from cerebral infarction or intraparenchymal haemorrhage<br>• Identified through from clinical referrals.<br>• All confirmed by neuroimaging.<br>• Severity rated on 5-point scale adapted from Vargha-Khadem et al.<br>•	**Outcomes**<br>• Behavioural<br>• Cognitive<br>• Epilepsy<br><br>**Measurement/ assessment**<br>• Achenbach CBCL<br>• WPPSI-R (4-5 years)<br>• WISC-R (6-16 years)<br><br>**Follow-up**<br>• 4-18 years<br>• 100% follow up	**Cognitive**<br>**Full scale IQ mean (SD)**<br>Stroke 93.4 (22)<br>Control 116.2 (13) p<0.0001<br><br>Left stroke 90.1 (22)<br>Right stroke 97.4 (22) – no significant difference<br><br>**Seizures (outside of the neonatal period)**<br>Stroke n=17, 50% (missing data for 5 subjects)
**Central nervous system infections**				
38	Bedford 2001[48]<br><br>England & Wales<br><br>Prospective cohort	**Population**<br>• All gestational ages included<br>• Born 1985-1987<br><br>**Exposure (n=274)**<br>• Neonatal meningitis<br><br>**Comparison (n=1391)**<br>• Matched on age and sex<br>• Recruited through GP<br><br>**Ascertainment/ definition**<br>• Identified through clinician reporting	**Outcomes**<br>• Neuromotor disability (composite)<br>• Cognitive<br>• Hearing<br>• Vision<br>• Behaviour<br>• Seizure disorder<br><br>**Assessment/ measurement**<br>• Parental questionnaire<br>• GP questionnaire<br>• McIntyre et al. classification of disability severity<br><br>**Follow-up**<br>• 5 years<br>• 85-94% follow-up	**Neuromotor disability**<br>Meningitis, n=45, 16%<br>No meningitis, n=2, 0.1%<br><br>**Severe disability**<br>Meningitis, n=20, 7%<br>No meningitis, n=1, 0.1%<br><br>**Moderate disability**<br>Meningitis, n=50, 18%<br>No meningitis, n=20, 1%<br><br>**Mild disorder**<br>Meningitis, n=66, 24%<br>No meningitis, n=275, 20%<br><br>**No disability**<br>Meningitis, n=138, 50%<br>No meningitis, n=1095, 79%

Confidential: For Review Only

39	Horváth-Puhó 2021[49]<br><br>Denmark and Netherlands<br><br>Retrospective matched cohort study	**Population**<br>• Gestation not specified<br>• Born 1997-2017<br><br>**Exposure**<br>• GBS meningitis (Denmark) (n=168)<br>• GBS meningitis (Netherlands) (n=198)<br><br>**Comparison**<br>• Randomly selected<br>• Matched 1:10 on sex, birth year and month, and gestation<br>• No GBS (Denmark) (n=13,689)<br>• No GBS (Netherlands) (n=4,983)<br><br>**Ascertainment/ definition**<br>• Invasive Group B Streptococcal disease by 89 days of age (most were neonatal – hence inclusion)<br>• ICD 10 codes (Denmark)<br>• CSF culture positive on national laboratory register (Netherlands)	**Outcomes**<br>• Neurodevelopmental impairment (composite)<br>• Cognitive<br>• Motor<br>• Behavioural, mental and social disorders<br>• Hearing impairment<br>• Visual impairment<br><br>**Assessment/ Measurement**<br>• ICD 10 codes<br><br>**Follow-up**<br>• Denmark 5 years, 7 years, 10 years, 15 years<br>• Netherlands 5 years, 7 years, 10 years and 11 years<br>• 95% follow-up	**Any neurodevelopmental impairment RR (95%CI)**<br>**<5 years**<br>Denmark GBS meningitis 7·80 (4·42-13·77)<br>Netherlands GBS meningitis 5·30 (2·57-10·89)<br><br>**<7 years**<br>Denmark GBS meningitis 4·69 (2·78-7·89)<br>Netherlands GBS meningitis 3·71 (1·05-6·72)<br><br>**<10 years**<br>Denmark GBS meningitis 3·47 (2·19–5·50)<br>Netherlands GBS meningitis 2·81 (1·69-4·68)<br><br>**<11 years**<br>Netherlands GBS meningitis 2·99 (1·83-4·88)<br><br>**<15 years**<br>Denmark GBS meningitis 3·15 (1·82–5·46)<br><br>**Moderate to severe neurodevelopmental impairment RR (95%CI)**<br>**<5 years**<br>Denmark GBS meningitis 8·49 (4·28-16·86)<br>Netherlands GBS meningitis 5·13 (2·24-11·79)<br><br>**<7 years**<br>Denmark GBS meningitis 5·27 (2·80-9·92)<br>Netherlands GBS meningitis n/a<br><br>**<10 years**<br>Denmark GBS meningitis 3·88 (2·15–6·99)<br>Netherlands GBS meningitis 3·05 (1·62-5·73)<br><br>**<11 years**<br>Netherlands GBS meningitis 3·34 (1·77-6·33)<br><br>**<15 years**<br>Denmark GBS meningitis 4·52 (2·35–8·67)
40	Martinez-Cruz 2008[51]<br><br>Mexico<br><br>Retrospective case control	**Population**<br>• Gestation < 34 weeks<br>• Birthweight <1500g<br>• Born 1990-2005<br><br>**Exposure (n=22)**<br>• Neonatal meningitis<br><br>**Comparator (n=374)**<br>• No meningitis<br><br>**Ascertainment/ definition**<br>• Meningitis not defined	**Outcomes**<br>• Sensorineural hearing loss<br><br>**Assessment/ measurement**<br>• Brainstem Auditory Evoked Potentials<br>• Transient Auditory Evoked Otoacoustic Emissions<br>• Tympanometry<br>• Free Field Audiometry<br>• Pure tone audiometry<br>• Behavioural hearing evaluation<br><br>**Follow-up**<br>• 7- 11 years<br>• 100% follow-up	**Meningitis**<br>Sensorineural hearing loss: n=15; 10.3%<br>No Sensorineural hearing loss: n=7; 2.6%<br>**Odds of previous neonatal meningitis if sensorineural hearing loss**<br>OR 4.368, 95% CI (1.7, 10.9) p= 0.002

Confidential: For Review Only

41	Stevens 2003[50]<br><br>England & Wales<br><br>Prospective cohort study	**Population**<br>• Term born infants<br>• Born 1985-1987<br><br>**Exposure (n=111)**<br>• Meningitis<br><br>**Comparison (n=162)**<br>• Matched on hospital of birth, birthweight and sex<br>• Hospital control (n=113)<br>• GP control (n=49)<br><br>**Ascertainment/ definition**<br>• CSF positive culture	**Outcomes**<br>• Disability and functional impairment (composite)<br>• Cognitive<br>• Motor<br>• Vision<br>• Hearing<br><br>**Assessment/ measurement**<br>• WISC-III<br>• Movement ABC<br>• Blinded examination<br>• Hearing screening<br>• Sonksen-Silver acuity system<br><br>**Follow-up**<br>• 9-10 years<br>• 67% follow-up of meningitis group	**Cognitive**<br>**IQ, mean (95% CI)**<br>Meningitis, 88.8 (85, 92)<br>Hospital control, 99.4 (97, 102)<br>GP control, 99.6 (95, 103)<br><br>**Motor**<br>**mABC score, mean (95% CI)**<br>Meningitis 7.1 (5.9, 8.5)<br>Hospital controls 5.0 (4.3, 5.8)<br>GP controls 4.0 (2.9, 5.4)<br><br>**Severe disability/ functional impairment**<br>Meningitis, n=12, 10.8%<br>Hospital control, n=0, 0%<br>GP control, n=0, 0%<br><br>**Moderate disability/ functional impairment**<br>Meningitis, n=10, 9%<br>Hospital control, n=2, 1.8%<br>GP control, n=0, 0%<br><br>**Mild disability/ functional impairment**<br>Meningitis, n=19, 17.1%<br>Hospital control, n=13, 11.5%<br>GP control, n=8, 16%<br><br>**No disability or functional impairment**<br>Meningitis, n=70, 63.1%<br>Hospital control, n=98, 86.7%<br>GP control, n=41, 84%<br><br>**Hearing loss (unilateral or bilateral sensorineural hearing loss or requiring hearing aids)**<br>Meningitis, n=4, 3,6%<br>Hospital control, n=0, 0%<br>GP control, n=0, 0%<br><br>**Visual impairment (bilateral)**<br>Meningitis, n= 18, 17% (6 unassessed because of their disability)<br>Hospital control, n=21, 18.5%<br>GP control, n=4, 8%<br><br>**Visual impairment (unilateral)**<br>Meningitis, n= 10, 9.9% (6 unassessed because of their disability)<br>Hospital control, n=8, 7%<br>GP control, n=2, 4%<br><br>**Seizures outside of the neonatal period**<br>Meningitis, n=6, 5.4%<br>Hospital control, n=2, 1.8%<br>GP control, n=0, 0%
Hypoxic-ischaemic encephalopathy				
42	3383 Koc 2016[30]<br><br>Turkey<br><br>Retrospective cohort	**Population**<br>• Gestation < 32 weeks<br>• Birthweight < 1500g<br>• Born 2001<br><br>**Exposure (n=9)**<br>• Perinatal asphyxia<br><br>**Comparator (n=81)**<br>• No asphyxia<br><br>**Ascertainment/ definition**<br>• Perinatal asphyxia diagnosed on: fetal pH, Apgar score, and neonatal cerebral and multiorgan dysfunction	**Outcomes**<br>• Cognitive<br><br>**Assessment/ measurement**<br>• WISC-R<br>• Performed by blinded psychologist<br><br>**Follow-up**<br>• 5-8 years<br>• 100% follow-up	**Cognitive**<br>**WISC-R IQ Score (combined verbal and performance scores) <85**<br>Perinatal asphyxia n=8, 89%<br>No asphyxia n=24, 30%<br>p=0.001

Confidential: For Review Only

43	Lee-Kelland 2019[52]*<br><br>United Kingdom<br><br>Retrospective cohort study	**Population**<br>• Gestation ≥ 36 weeks<br>• Born 2008-2010<br><br>**Exposure (n=29)**<br>• Moderate-severe HIE without subsequent cerebral palsy<br><br>**Comparator (n=20)**<br>• Matched on age, sex and social class<br>• Born without HIE<br><br>**Ascertainment/ definition**<br>• Received therapeutic hypothermia based on TOBY trial criteria	**Outcomes**<br>• Cognitive<br>• Motor<br>• Speech and language<br>• Behaviour<br><br>**Assessment/ measurement**<br>• WISC IV (blinded)<br>• Movement ABC 2<br>• Strengths and difficulties questionnaire<br><br>**Follow-up**<br>• 6-8 years<br>• 61% follow-up	**Cognitive**<br>**Full scale IQ, mean (SD)**<br>HIE 91 (10.37)<br>No HIE 105 (13.41)<br>Mean difference −13.62 95% CI (−20.53 to −6.71) p<0.001<br><br>**Perceptual reasoning, mean (SD)**<br>HIE 89 (11.15)<br>No HIE 103 (12.49)<br>Mean difference −13.9 95% CI (−20.78 to −7.09) p<0.001<br><br>**Working memory, mean (SD)**<br>HIE 94 (13.76)<br>No HIE 102 (13.82)<br>Mean difference −8.2 95% CI (−16.29 to −0.17) p=0.04<br><br>**Processing speed, mean (SD)**<br>HIE 96 (13.76)<br>No HIE 107 (17.59)<br>Mean difference −11.6 95% CI (−20.69 to −2.47) p=0.01<br><br>**Additional classroom support**<br>HIE n=10, 34%<br>No HIE n=1, 5%<br>OR: 10.0, 95%CI 1.16 to 86.0<br><br>**Special educational needs**<br>HIE n=1, 3.4%<br>No HIE n=0, 0%<br><br>**Motor**<br>**MABC-2 score, mean (SD)**<br>HIE 7.9 (3.26)<br>No HIE 10.2 (2.86)<br>Mean difference −2.12 95% CI (−3.93 to −0.30) p=0.02<br><br>**Speech and language**<br>**Verbal comprehension, mean SD)**<br>HIE 94 (8.79)<br>No HIE 103 (10.09)<br>Mean difference −8.8 95% CI (−14.25 to −3.34) p=0.002<br><br>**Behaviour**<br>**Total difficulties, median (IQR)**<br>HIE 12 (6.5–13.5)<br>No HIE 6 (2.25–10) P=0.005<br><br>**Emotional problems, median (IQR)**<br>HIE 2 (1–4.5)<br>No HIE 0.5 (0–2.75) P=0.03<br><br>**Hyperactivity, median (IQR)**<br>HIE 2 (1–3)<br>No HIE 1 (0–2) P=0.06<br><br>**Conduct problems, median (IQR)**<br>HIE 4 (2.5–6.5)<br>No HIE 3 (1–5) p=0.06<br><br>**Peer problems, median (IQR)**<br>HIE 0 (0–2.5)<br>No HIE 0 (0–1) p=3.56 Ω (potential error in manuscript table)<br><br>**Prosocial, median (IQR)**<br>HIE 9 (7.5–10)<br>No HIE 9 (8.25–10) p=0.13<br><br>**Impact score, median (IQR)**<br>HIE 0 (0–2.5)<br>No HIE 0 (0–2.0) p=0.31

Confidential: For Review Only

44	Tonks 2019[53]*<br><br>United Kingdom<br>Prospective cohort study	**Population**<br>• Gestation ≥36 weeks<br>• Born 2008-2011<br>• English as primary language<br><br>**Exposure (n=29)**<br>• Moderate-severe HIE without subsequent cerebral palsy<br><br>**Comparator (n=20)**<br>• Matched on age, sex and social class<br>• Recruited from schools in the area<br>• Born without HIE<br><br>**Ascertainment/ definition**<br>• Received therapeutic hypothermia based on TOBY trial criteria	**Outcomes**<br>• Cognitive<br>• Neuropsychological<br><br>**Assessment/ measurement**<br>• Conner's continuous performance test<br>• NEPSY-II block construction test<br>• NEPSY-II arrows' test<br><br>**Follow-up**<br>• 6-8 years<br>• 77% follow-up	<u>**Attention**</u><br>**Hit response time**<br>**HIE**<br>84.1 percentile mean rank 27;<br>Proportion performing below 2 SD 32%<br><br>**Comparator**<br>67.3 percentile mean rank 17.89; p = .024<br>Proportion performing below 2 SD 11%<br><br>**Hit response time standard error**<br>**HIE**<br>standard error mean rank 26.8<br>Proportion performing below 2 SD 18%<br><br>**Comparator**<br>standard error mean rank 18.2; p = 0.032<br>Proportion performing below 2 SD 11%<br><br>**Hit response time by block**<br>**HIE**<br>Mean 49.1, SD 23.9<br><br>**Comparator**<br>Mean 61.9, SD 18.4; p = 0.047<br><br><u>**Visual discrimination**</u><br><u>**HIE**</u><br>Below 1 SD 10%<br><br><u>**Comparator**</u><br>Below 1 SD 5%<br>HIE vs comparator scores, p = 0.049<br><br><u>**Visuo-spatial mental rotation task**</u><br><u>**HIE**</u><br>Below 1 SD 17%<br><br><u>**Comparator**</u><br>Below 1 SD 5%<br>HIE vs comparator scores, p = 0.034

Confidential: For Review Only

Supplement 5: Risk of bias table

overlapping data; Clinical Evaluation of Language Fundamentals (CELF); Cystic Periventricular leukomalacia (cPVL); Intelligence Quotient (IQ); Intraventricular haemorrhage (IVH); Mental Developmental Index (MDI); Neonatal Intensive Care Unit (NICU); Psychomotor Development Index (PDI); Periventricular leukomalacia (PVL); Spontaneous Intestinal Perforation (SIP); Wechsler Intelligence Scale for Children (WISC); White Matter Injury (WMI);

Preterm brain injury: cohort studies

	Selection (*satisfactory; No =not satisfactorily done; n/a)				Comparability (*satisfactory; No =not satisfactorily done; n/a)		Exposure/ Outcome (*satisfactory; No =not satisfactorily done; n/a)			Subtotal assessment			Total score: 0-3 high risk of bias; 4-6 moderate risk of bias 7-9 low risk of bias	Additional comments
	1	2	3	4	1a	1b	1	2	3	Selection (0-1=Poor; 2=Fair; 3+ Good)	Comparability (0=poor; 1=fair; 2+=good)	Exposure / outcome (0=poor; 1=fair; 2+=good)		
Adant 2019	No	*	*	* (excluded those with congenital anomalies)	*	*	No	*	No	Good	Good	Fair	6	Population not representative as focus of study was spontaneous intestinal perforation. Infants without IVH didn't have brain injury excluded per se (but didn't have IVH 3-4 on imaging). Matched on gender, gestational age, date of birth. Multiples matched to sibling without SIP. Excluded those with necrotising enterocolitis, mechanical obstruction or congenital anomalies. Adjusted for gender, gestation, birthweight, SIP and IVH.<br><br>Independent outcome assessment but not blinded; telephone survey of parents. High numbers lost to follow-up. Table 3 contains errors with respect to outcomes (MDI and PDI mislabelled as motor and cognitive respectively).

Confidential: For Review Only

Beaino 2010	*	*	No	* (cerebral palsy could not be present at birth)	*	*	*	*	*	Good	Good	Good	8	3% of infants did not have a cranial ultrasound, a further 11% had only one cranial ultrasound during neonatal period - therefore ascertainment of exposure may be compromised<br><br>Model A adjusted for:<br>• obstetric factors<br>• cerebral lesions<br>Model B adjusted for:<br>• obstetric factors<br>• neonatal factors<br><br>Model C was the same as model B for those without cPVL or Intraparenchymal haemorrhage<br><br><85% follow-up for enrolled infants but clear description of those lost to follow-up and no significant differences with respect to ultrasound brain injury findings between groups
Brouwer 2012	No	No	*	* (given the types of outcomes assessed)	No	No	No	*	*	Fair	Poor	Good	4	Study of a select group i.e. those with IVH requiring neurosurgical intervention.<br>No description of setting, how patients were enrolled, how many were excluded<br>No description of how control group was derived, or what era they were from.<br>Only some infants (those <30weeks) were matched on gestation, birthweight, sex to controls.<br>Different intelligence tests used at follow-up. >80% completion rate of Child Behaviour Checklist and teacher report form by parents and teachers

Confidential: For Review Only

Campbell 2021	*	*	*	* (given the types of outcomes assessed)	*	*	*	*	No	Good	Good	Good	8	Males and those born at 23-24 weeks gestation were overrepresented in the IVH WMI group.<br>Adjusted for gestation, birthweight Z score, sex, maternal education, bronchopulmonary dysplasia, sepsis, necrotising enterocolitis (Bell stage 2-3) and severe retinopathy of prematurity.
Cheong 2018#	*	*	*	No (visual or hearing impairment could be congenital)	*	*	*	*	*	Good	Good	Good	8	Adjusted for era of birth, antenatal corticosteroid exposure, inborn status, gestation, sex, multiple birth, birthweight Z score, surfactant use, IVH grade 3 or 4 (in cPVL), cPVL (in IVH grade 3-4), bronchopulmonary dysplasia, postnatal corticosteroid use, necrotising enterocolitis (stage 2 or worse), surgery in the newborn period, and retinopathy of prematurity (stage 3 or worse).
Chou 2020	*	*	*	* (given the types of outcomes assessed)	No	*	*	*	No	Good	Fair	Good	7	Matched and adjusted for, urbanisation and parental occupation.<br><br>No information about missing data or completeness of follow-up

Confidential: For Review Only

Davidovitch 2020	*	*	*	* (given the types of outcomes assessed)	No	*	*	*	No	Good	Fair	Good	7	Only low birthweight infants included (therefore birthweight partially accounted for). Unmatched.<br>No information about excluding brain injury from comparators e.g. comparing those with IVH grade 3-4 to those without could include those with IVH 1-2; both groups could also include infants with other types of brain injury.<br>Missing data not presented or accounted for. Adjusted the composite brain injury group (which included retinopathy of prematurity in its definition) for gestation, maternal diabetes, small for gestational age, year of birth, bronchopulmonary dysplasia, and receipt of postnatal steroids.
Doyle 2000 #	*	*	*	* (given the types of outcomes assessed)	No	No	*	*	*	Good	Poor	Good	7	IVH and no IVH groups not matched for gestation or birthweight, no adjustment for these variables appears to have been done.<br><br>Relatively old cohort (most did not receive surfactant), comparator group only includes infants born in the 1980s. Not representative due to time-period of care.
Hintz 2018	*	*	*	* (given the types of outcomes assessed)	*	*	*	*	*	Good	Good	Good	9	Assessed interobserver reliability of central imaging readers.<br><br>Unmatched<br><br>Adjusted for gestation, race, sex, multiple gestation, maternal education, sepsis, bronchopulmonary dysplasia, postnatal steroids, surgery for patent ductus arteriosus, necrotising enterocolitis, retinopathy of prematurity.<br><br>Only 83% follow-up of survivors but those lost to follow-up are accounted for.

Confidential: For Review Only

Hirovonen 2017	*	*	*	* (given the types of outcomes assessed)	*	*	*	*	*	Good	Good	Good	9	Excluded infants who died at <1 year of age, infants with major congenital anomalies, and those with missing data.<br><br>Characteristics of those with brain injury not presented.<br><br>No breakdown by severity of brain injury because that level of detail was not available in the database.<br><br>No matching but there is stratification by gestation and adjustment for: maternal characteristics, pregnancy characteristics, delivery characteristics, sex, gestation, birthweight, Apgar score at 1-minute, umbilical artery pH, resuscitation provided, NICU admission, receipt of phototherapy, ventilator requirement, antibiotic receipt, respiratory distress syndrome, sepsis, seizures, hyperbilirubinaemia.
Hollebrandse 2021#	*	*	*	* (given the types of outcomes assessed)	*	*	*	*	*	Good	Good	Good	9	Gestation similar across all groups and other baseline perinatal characteristics similar across groups.<br><br>Preterm brain injury and no brain injury group not matched. Unclear if IVH and no IVH group had other brain injuries excluded or may have had more than one injury type (e.g. PVL).<br>Impact of epoch/ era of birth explored and adjusted for.
Hreinsdottir 2018	*	*	*	No (visual impairment could have been congenital)	*	*	*	*	No	Good	Good	Good	7	Unsure if comparator group in logistic regression includes those with IVH 1-2. Adjusted for gestation, birthweight, retinopathy of prematurity, sex, cognitive score, cerebral palsy.

Confidential: For Review Only

Jansen 2020	*	*	*	* (given the types of outcomes assessed)	No	No	*	*	No	Good	Poor	Good	6	Excluded infants with congenital abnormalities, metabolic disorders or neonatal meningitis.
Kaur 2020	*	*	*	No (visual or hearing impairment could be congenital)	No	*	*	*	No	Good	Fair	Good	6	Unmatched. Compared infants with IVH to all infant without haemorrhage (of all gestations).<br>Adjusted for maternal age, pregnancy complications, infant sex, neonatal comorbidity, birthweight, socioeconomic deprivation, and year of birth.
Kiechl-Kohlendorfer 2013	*	*	*	* (given the types of outcomes assessed)	*	*	*	No	No	Good	Good	Fair	7	Low numbers of infants included. Outcomes assessed at 1 year - likely not long enough for robust assessment of neurodevelopmental outcomes; <85% follow-up and no detailed description of those lost to follow up - though authors do state that there were no significant differences between those followed up and those lost to follow up.
Klebermass-Schrehof 2012	*	*	*	No (could have had congenital blindness)	*	No	*	*	No	Good	Fair	Good	6	Adjusted for gestation.<br>Significant difference between groups for key neonatal comorbidities such as ROP, RDS, CLD and characteristics such as antenatal steroid exposure.<br><br>No clear description of number lost to follow-up, though mentions that follow-up rate at 5.5 years was 54-61%.
Koc 2016	*	*	No	* (given the types of outcomes assessed)	No	No	*	*	No	Good	Poor	Good	5	Small numbers included. No breakdown of characteristics of those with brain injury. No description of IVH grading used or schedule of ultrasound exams; no description of criteria for establishing perinatal asphyxia, number lost to follow-up not stated.

Confidential: For Review Only

Neubauer 2008	*	n/a	*	No (deafness or blindness could have been congenital)	*	*	*	*	*	Fair	Good	Fair	7	Neurodevelopmental assessors not blinded; follow-up rate <85% but paper does give description of those lost to follow-up
Piris Borregas 2019	*	*	*	* (excluded infants with congenital malformations)	No	No	*	*	No	Good	Poor	Good	6	Only those followed up to 7 years included.<br><br>Excluded infants who died before 36 weeks corrected age, with major malformations, or those with missing data.<br><br>Unclear if independent odds ratio includes adjustment for covariates.<br><br>Unclear if those without 'severe brain injury' had other types of brain injury.
Pittet 2019	*	*	*	* (excluded infants with congenital malformations)	No	*	*	*	*	Good	Fair	Good	8	Excluded infants with congenital malformations affecting neurodevelopment and infants from centres without 5 years of follow-up cognitive testing.<br><br>Unclear if other types of brain injury excluded from comparator group.<br><br>Adjusted for gender and socioeconomic status. No significant difference in cognitive outcome between extreme preterms and those 28-30 weeks' gestation. Gestation not adjusted for.
Sherlock 2005#	*	*	*	No (deafness or blindness could have been congenital)	No	No	*	*	*	Good	Poor	Good	6	Comparability of IVH vs. no IVH cohorts not clear - not enough information to determine if groups were comparable with respect to gestational age or birthweight

Tymofiyeva 2018	*	*	*	* (given the types of outcomes assessed)	No	No	*	*	No	Good	Poor	Good	6	Excluded infants with congenital malformations/ syndromes, congenital infections, or those who were too unstable for MR imaging. The last exclusion criteria in particular could limit generalisability quite considerably.<br><br>Unclear about the validity of grouping the attention scores across different assessment tools together into a dichotomous variable for attention.
Van De Bor 2004	*	*	*	* (excluded those with major congenital malformations)	*	*	No	No	*	Good	Good	Fair	7	IVH vs. no IVH cohorts comparable with respect to gestation; some differences in gender composition but paper states this was controlled for in the analysis. Primary outcome entirely self-reported. Outcomes reported at 14 years.<br><br>Adjusted for gestational age, birth weight, small for gestational age, sex, ethnicity, duration of assisted ventilation, maximum serum total bilirubin concentration and maternal education.
Van Den Hout 2000	* (except for HIE exposure group)	*	*	* (excluded those with congenital anomalies)	No	No	*	*	*	Good	Poor	Good	7	Low numbers and relatively old cohort. Relative gender imbalance in IVH group compared to those with normal scans or PVL. IVH group also 1.4 weeks more premature than 'normal scan' group.
Vollmer 2003#	*	*	*	No (deafness or blindness could have been congenital)	*	No	*	*	*	Good	Fair	Good	7	Note change in version of Weschler scale during follow-up period. Authors state no difference in mean IQ after change. Baseline characteristics of groups with and without brain injury not given; no indication of matching or adjustment for factors other than gestation.

Vollmer 2006a#	*	*	*	No (deafness or blindness could have been congenital)	*	*	*	*	*	Good	Good	Good	8	Note gender imbalance in cohort as a whole (M>F), but male: female ratio in each group appears similar. No matching or adjustment for covariates.<br><br><85% follow-up but clear description of those lost and appears no significant differences.
Vollmer 2006b#	*	*	*	No (deafness or blindness could have been congenital)	No	No	*	*	No	Good	Poor	Good	5	Marked gender imbalance in ventricular dilatation group. Lower birthweight and gestation in groups with abnormal cranial ultrasound. No indication of matching or adjustment.<br><br><85% follow-up and the limited description of those lost to follow-up indicates that these babies were of lower birthweight and gestation.
Whitaker 2011	*	*	*	* (given the types of outcomes assessed)	*	*	(No)	*	*	Good	Good	Good	8	Severely disabled survivors (n=33) were excluded.<br><br>Half had later ultrasounds (just before discharge).<br><br>No breakdown of the characteristics of the exposed and comparator groups – unable to assess how comparable they are.<br><br>Adjusted for: maternal social risk, sex, gestation, fetal growth ratio, multiplicity, maternal smoking status, maternal alcohol status, labour onset, presentation at birth, base excess on first postnatal blood gas, thyroid status, hypocapnia, hypoxia, systolic hypotension, prolonged ventilation.<br><br>Primary outcome assessment reliant on parental report, albeit via structured interview with some evidence for validity. Interviewers were blinded to the child's history. Parents were blinded to the study hypothesis.

Confidential: For Review Only

														Less than 85% follow-up (psychiatric interviews in 51% of survivors) however clear descriptions of groups with and without psychiatric evaluation given in table 2 and little apparent difference between groups.
**Preterm brain injury: case-control studies**														
	1 Case definition	2 Representativeness of cases	3 Selection of controls	4 Definition of controls	1a	1b	1 Ascertainment of exposure	2 Same method of ascertainment for cases and controls	3 Non-response rate	(0-1=Poor; 2=Fair; 3+ Good)	y (0=poor; 1=fair; 2+=good)	(0=poor; 1=fair; 2+=good)	Total score: 0-3 high risk of bias; 4-6 moderate risk of bias 7-9 low risk of bias	Additional comments
Martinez-Cruz 2008 (IVH)	*	*	*	*	*	No	*	*	No	Good	Fair	Good	7	Appears to be case-control design hence star ratings are as per case control rating sheet. Controls not well matched for birth weight. No description of whether full information on exposures could be obtained for all cases/controls e.g. missing records etc.
Perinatal stroke: cohort studies														

Confidential: For Review Only

	Selection (*satisfactory; No =not satisfactorily done; n/a)				Comparability (*satisfactory; No =not satisfactorily done; n/a)		Exposure/ Outcome (*satisfactory; No =not satisfactorily done; n/a)			Subtotal assessment			Total score: 0-3 high risk of bias; 4-6 moderate risk of bias 7-9 low risk of bias	Additional comments
	1	2	3	4	1a	1b	1	2	3	Selection (0-1=Poor; 2=Fair; 3+ Good)	Comparability (0=poor; 1=fair; 2+=good)	Exposure / outcome (0=poor; 1=fair; 2+=good)		
Ballantyne 2007	No	No	*	*	No	*	No	*	No	Fair	Fair	Fair	4	No description of derivation of exposed cohort - whether single institute or multicentre, whether same community as non-exposed group or not.<br><br>Predominance of right-handed children amongst controls otherwise similar baseline characteristics. Note male preponderance in exposed group and female preponderance in non-exposed<br><br>No matching or adjustment for confounders.<br><br>No description of who performed outcome assessment, whether blinded and independent.
Ballantyne 2008	*	*	*	No	No	*	*	*	No	Good	Fair	Good	6	Excluded children with brain lesions from other causes e.g. head trauma, tumours<br><br>Gestational age of exposed cohort ranged from 32 to 40 weeks. No statement as to whether control group were matched on this. Note preponderance of males in stroke group and females in control group.<br><br>In study 1, significant numbers of participants did not complete the planned developmental assessments - across exposed and control groups, completeness ranged from 50% for WISC-R to 69% for CELF-R.

Gold 2014	No	No	*	*	No	*	*	*	*	Fair	Fair	Good	6	No description of how subjects were selected or recruited from neurology clinics. Nonexposed group selected from a different source. No description of gestational age of subjects or of controlling for this. Matched for age at follow up, sex, socioeconomic group and maternal education.<br><br>Excluded infants with bilateral lesions, a history of hypoxic ischemic encephalopathy, central nervous system infection, in-utero drug exposure, significant closed head injury, or any other condition that might have caused brain damage other than from the stroke.
Kolk 2011	*	*	*	*	No	*	*	*	No	Good	Fair	Good	7	No description of gestational age of subjects or of controlling for this. Difficult to ascertain completeness of follow-up from paper. Adjusted for age of outcome assessment.
Martin 2019	*	*	*	*	No	*	*	*	*	Good	Fair	Good	8	Excluded infants with bilateral lesions, hearing impairment, or a history of a problem that may have caused more global brain damage (e.g. meningitis, closed head injury, hypoxic-ischemic encephalopathy). Matched on age, sex and socioeconomic status
Northam 2018	*	No	*	*	*	*	*	*	*	Good	Good	Good	8	No description of source of unexposed cohort. Matched on age, sex, and maternal education.
Tillema 2008	*	*	*	*	No	*	*	*	No	Good	Fair	Good	7	Exposed and comparator groups not matched for gestation, but were matched for age, sex and handedness. 17 subjects included initially but 7 of these excluded for various reasons meaning that neurodevelopmental outcome data/Weschler scores only presented for 10 of 17.

Trauner 2013	*	*	*	*	No	No	No	*	No	Good	Poor	Fair	5	Excluded infants if bilateral or multifocal lesions identified, history of meningitis, or history of antenatal drug exposure<br><br>Matched on age and socioeconomic status<br><br>No baseline characteristics given to establish comparability of exposed and comparator cohorts. Likely comparable with regards to gestation based on stated inclusion criteria. Main outcome measure based on parental questionnaire - no direct linguistic assessments done, however may not have been feasible/appropriate in such a young cohort. No information on response rate/loss to follow-up.<br><br>IQ used as covariate<br><br>IQ combined across the age range and assessed with two different tools. This assumes IQ is fixed which may not be true.
Central nervous infections: cohort studies														
	Selection (*satisfactory; No =not satisfactorily done; n/a)				Comparability (*satisfactory; No =not satisfactorily done; n/a)		Exposure/ Outcome (*satisfactory; No =not satisfactorily done; n/a)			Subtotal assessment			Total score: 0-3 high risk of bias; 4-6 moderate risk of bias 7-9 low risk of bias	Additional comments
	1	2	3	4	1a	1b	1	2	3	Selection (0-1=Poor; 2=Fair; 3+ Good)	Comparability (0=poor; 1=fair; 2+=good)	Exposure / outcome (0=poor; 1=fair; 2+=good)		

Confidential: For Review Only

Bedford 2001#	*	*	*	No	*	*	No	*	*	Good	Good	Good	7	Matched on sex and age.<br><br>Study focuses on meningitis in infancy but also presents outcomes after neonatal meningitis.<br><br>Did not exclude children with other comorbidities e.g. congenital conditions associated with neurodevelopmental impairment. Exposed cases derived from same cohort as Stevens 2003. Outcome assessment based on parent or GP report with no formal neurodevelopmental assessment.
Horváth-Puhó 2021	*	*	*	No	*	*	*	*	*	Good	Good	Good	8	Invasive Group B Streptococcal infection diagnosed in the first 89 days (however most of these were neonatal, particularly in the first week of life (45%) hence inclusion.<br><br>Matched 1:10 on sex, birth year and month, and gestation.<br>Neurodevelopmental impairment defined differently in each cohort.<br>Missing data accounted for and its impact explored.
Stevens 2003#	(*)	(*)	*	No	*	*	*	*	No	Good	Good	Good	7	Exposed cohort based on recall of consultant paediatricians filling out monthly returns thus may be biased towards more severe or otherwise memorable cases. Some in comparator group selected from a different hospital than exposed cohort.<br><br>Matched on hospital of birth, birth weight and sex.<br><br>Results stratified by birthweight<br><br>Significant rate of loss to follow-up.
**Central nervous system infections: case control studies**														

Confidential: For Review Only

	1 Case definition	2 Representativeness of cases	3 Selection of controls	4 Definition of controls	1a	1b	1 Ascertainment of exposure	2 Same method of ascertainment for cases and controls	3 Non-response rate	(0-1=Poor; 2=Fair; 3+ Good)	y (0=poor; 1=fair; 2+=good)	(0=poor; 1=fair; 2+=good)	Total score: 0-3 high risk of bias; 4-6 moderate risk of bias 7-9 low risk of bias	Additional comments
Martinez-Cruz 2008	*	*	*	*	No	No	*	*	No	Good	Poor	Good	6	Excluded those with history of parental consanguinity or TORCH infections.<br><br>Number of those with and without meningitis who may have had other types of brain injuries not specified – unable to assess overlap/ impact of meningitis alone.<br><br>Odds ratio presented for meningitis does not appear to be crude so potential adjustment for confounding factors but no description of this in the methods section.<br><br>No description of proportion of missing data.
**Hypoxic-ischaemic encephalopathy: cohort studies**														
	Selection (*satisfactory; No =not satisfactorily done; n/a)				Comparability (*satisfactory; No =not satisfactorily done; n/a)		Exposure/ Outcome (*satisfactory; No =not satisfactorily done; n/a)			Subtotal assessment			Selection (*satisfactory; No =not satisfactorily done; n/a)	Additional comments
	1	2	3	4	1a	1b	1	2	3	Selection (0-1=Poor; 2=Fair; 3+ Good)	Comparability (0=poor; 1=fair; 2+=good)	Exposure / outcome (0=poor; 1=fair; 2+=good)	Total score: 0-3 high risk of bias; 4-6 moderate risk of bias 7-9 low risk of bias	

Confidential: For Review Only

Koc 2016	No	*	*	*	No	No	*	*	No	Fair	Poor	Good	5	Representativeness not clear as no description given of babies who did not complete follow-up at the study institution. No apparent adjustment for gestation or other covariates. Pre-therapeutic hypothermia era.<br><br>Small number, no breakdown of characteristics or other neurodevelopmental outcomes by brain injury<br><br>Number of those with and without birth asphyxia who had other types of brain injuries e.g. IVH not specified.
Lee-Kelland 2019	No	*	*	*	*	*	*	No	No	Good	Good	Good	6	Excluded those who underwent therapeutic hypothermia outside of the standard criteria, infants with metabolic disorders and non-English speaking infants.<br><br>Matched on age, sex and social class.
Tonks 2019	*	No	*	*	No	*	*	*	No	Good	Fair	Good	6	Included cases had no diagnoses other than encephalopathy.<br>Excluded infants with neurological issues other than encephalopathy. Matched on age, sex and socioeconomic status.

Confidential: For Review Only

Supplement 6: Overview of key findings for school-age outcomes of infants with perinatal brain injury compared to those without brain injury
(*Does not include studies where infants with IVH grade 3-4 cannot be separated from those with WMI or those with IVH 1-2)
(# Does not include studies using hearing or visual outcomes only as part of their composite outcome)
Adjusted Odds Ratio (aOR); Attention Deficit Hyperactivity Disorder (ADHD); Autism Spectrum Disorder (ASD); Confidence Interval (CI); cystic periventricular leukomalacia (cPVL); Group B Streptococcus (GBS); Hypoxic-Ischaemic Encephalopathy (HIE); Hazard Ratio (HR); Intelligence Quotient (IQ); Interquartile range (IQR); Intraventricular Haemorrhage (IVH); Odds Ratio (OR); Periventricular Leukomalacia (PVL); Visual Motor Integration (VMI); White Matter Injury (WMI)

	NDI	Cognitive	Motor	Speech and language	Behavioural	Hearing#	Vision#	Other
IVH grade 3-4*	6 studies(15, 17-21)<br><br>2 comparable studies in meta-analysis(17, 20)<br><br>**Meta-analysis** (2 studies): Increased risk of moderate - severe neurodevelopmental impairment OR 3.69 (95%CI: 1.7, 7.98) $I^2 = 0\%$<br><br>**Van de Bor 2004**: increased prevalence of disability 31% vs. 16%	9 studies(15, 20, 21, 24-26, 30, 70)<br><br>Not comparable<br><br>Consistently highlighted lower cognitive scores<br><br>**Brouwer 2012**: significantly lower performance IQ but preserved verbal IQ. Lower IQ for those with IVH grade 4 requiring neurosurgery (91+/-10 vs. 98+/-15) but little difference for those with grade 3 IVH requiring neurosurgery (96+/-15 vs. 98+/-15).<br><br>**Hollebrandse 2021**: increased risk of cognitive impairment OR 2.68 (95%CI: 1.21, 5.94). Increased risk of academic impairment across all academic domains:<br>reading OR 3.62 (95%CI: 1.59, 8.24);<br>spelling OR 4.48 (95%CI: 1.8, 11.2);<br>arithmetic OR 2.79)95%CI: 1.2, 6.48)<br><br>**Sherlock 2005:** significantly lower IQ scores after IVH grade 4 vs. IVH 1-3 and no brain injury, also seen for several domains: freedom from distractibility,	6 studies(20, 23-26, 33)<br><br>Not comparable<br><br>All reported increased risk of motor impairment<br><br>**Cerebral palsy**<br>2 comparable studies<br><br>OR 8.13 (95%CI: 4.64, 14.22) I^2=0%.	3 studies(20, 21, 25)<br><br>Not comparable<br><br>**Van de Bor 2004**: no significant difference in language scores<br><br>**Sherlock 2005**: downward trend in language scores from no brain injury to each grade of IVH but not statistically significant p=0.12<br><br>**Hollebrandse 2021**: Increased risk of impaired reading OR 3.62 (95%CI: 1.59, 8.24), and spelling OR 4.48 (95%CI: 1.8, 11.2)	3 studies(15, 24, 35)<br><br>Not comparable<br><br>**Brouwer 2012**: no association with any behavioural domains assessed (internalising, externalising and sleep problems)<br><br>**Adant 2019**: no increased risk of attention deficits, conduct issues or ASD aOR 1.24 (95%CI: 0.32, 4.8).<br><br>**Davidovich 2020**: no increased risk of ASD (n=10, 3.9% vs. n=103, 2.2% p=0.085)	3 studies(21, 26, 38)<br><br>Not comparable<br><br>Outcome too rare for inferential analysis<br><br>**Kaur 2020:** increased risk of hospitalisation for otologic reasons HR 7.87 (95%CI: 5.31, 11.67)	5 studies(15, 21, 26, 33, 38)<br><br>Not comparable<br><br>Outcome to rare for inferential analysis in most studies.<br><br>**Adant 2019**: no increased risk of visual impairment (needing glasses) aOR 0.47 (95%CI: 0.13, 1.69)<br><br>**Klebermass-Schrehof 2012:** increased prevalence of visual impairment (needing glasses or blindness) after IVH grade 3 (45.4%) and IVH grade 4 (90.9%) vs. comparators (7.5%).<br><br>**Kaur 2020:** increased risk of hospitalisation for ophthalmic reasons HR 7.87 (95%CI: 5.31, 11.67).<br><br>**Klebermass-Schrehof 2012:** significantly lower VMI scores	

Confidential: For Review Only

		processing speed, reading, spelling and arithmetic. No difference in executive function.<br><br>**Van de Bor 2004**: increased special education needs at 5, 9 and 14 years<br>aOR 3.99 (95%CI: 1.36, 11.69).						(67.5 ± 14 vs. 76 ± 26.8; p=0.04)	
WMI*	3 studies(16, 17, 22)<br><br>Not comparable<br><br>**Campbell 2021**: living with no impairment was less common with WMI (n=12, 40%) vs. controls (n=487, 76%)<br><br>**Cheong 2018**: increased risk of survival with major disability after cPVL aOR 9·17 (95%CI: 3·57, 23·53)<br><br>**Vollmer 2003:** Disabling impairments were more common after cPVL at<28 weeks' gestation (n=3, 75% <28 weeks) vs. controls (n=3,	4 studies(16, 29, 32, 70)<br><br>Not comparable<br><br>**Van den Hout 2000**: 50% with PVL had IQ scores <85 vs. 11.8% without injury and a lower performance age 4.3 years vs. 6.2 years<br><br>**Campbell 2021**: increased risk of moderate-severe cognitive impairment aOR 5.07 (95%CI: 2.13, 12.02)<br><br>**Jansen 2020**: WMI predictive of poorer performance on standardised mathematics tests (B 1.856 p=0.003), but not performance on spelling (B 1.076 p=0.075) or reading tests (B 0.241 p=0.483)	**<u>Cerebral palsy</u>**<br>1 study(16)<br><br>**Campbell 2020**: increased risk of cerebral palsy aOR 18.63 (95%CI: 7.37, 47.06)	1 study(29)<br><br>**Jansen 2020**: No association between WMI and spelling (B 1.076 p=0.075) or reading performance (B 0.241 p=0.483)	4 studies(16, 35, 36, 71)<br>Not comparable<br><br>Conflicting results<br><br>**Campbell 2021**: No increased risk of:<br>ADHD (n=3, 10% vs. n=97, 15%); anxiety (n=3, 10% vs. n=98, 15%); depression (n=7, 23% vs. n=100, 16%); or ASD aOR 0.74 (95%CI: 0.09, 5.88)<br><br>**Davidovich 2020**: No increased risk of ASD after PVL (n=5, 2.5% vs. n=88, 2.3% p=0.86)<br><br>**Whitaker 2011:** increased risk of ADHD aOR 6.83 (95%CI: 1.26-36.91); major depression aOR 2.59 (95%CI: 1.02-6.58); tic disorders aOR 9.77 (95%CI: 1.69-56.47); obsessive compulsive disorders aOR 15.32 (95%CI: 1.82-128.74)	0 studies	1 study(32)	

Confidential: For Review Only

	8%) and at over 28 weeks' gestation (n=6,50% vs. n=14, 6%)							
Stroke	0 studies	6 studies(39, 41, 42, 44-46)<br>5 comparable studies in meta-analysis (39, 41, 44-46)<br><br>**Meta-analysis** (5 studies): significant mean difference in full scale IQ: -24.2 (95%CI: -30.73, -17.67) I^2=80%<br><br>**Trauner 2001 and Gold 2014**: no significant difference in full scale IQ scores in left vs. right-sided strokes<br><br>**Ballantyne 2008:** significantly lower performance IQ (p=0.002) and verbal IQ (p<0.0001). Lower mean scores for reading (p<0.0001), spelling (p=0.001) and arithmetic (p<0.0001) at 7-8 years persisting to 10-12 years<br><br>**Tillema 2008:** reduced verbal IQ scores (mean 84 SD 13.4) vs. (mean 108 SD 14.2 P=0.002)<br><br>**Kolk 2011**: poorer attention (across 4 of the 7 assessment sub-domains), visuo-spacial function (across 4 of the 5 sub-domains), and memory and learning (across 4 of the 6 sub-domains), but normal executive function scores. Those with left-sided strokes	5 studies(39, 41-44)<br>Combined hemiparesis incidence: 61% (95%CI: 39.2, 82.9 I^2=88%)<br><br>**Kolk 2011:** moderate to severe neuromotor impairment in 62% n=13) and significantly lower scores on 5/6 sensorimotor domains of the NEPSY	5 studies(39, 40, 42, 44, 45)<br><br>3 comparable studies in meta-analysis<br>**Meta-analysis** (3 studies): lower receptive language scores-20.88 (95%CI: -36.66, -5.11) I^2=88% and lower expressive language scores -20.25 (95%CI: -34.36, -6.13) I^2=87%<br><br>**Ballantyne 2007 and Ballantyne 2008**: deficits in receptive language scores at 7-8 years persist at 10-12 years but expressive language scores improved (p=0.012) particularly for children with right-sided strokes (p=0.034)<br><br>**Kolk 2011:** significantly lower scores for 8/9 NEPSY domains including phonologic processing, comprehension of instructions, correct speeded naming, repetition of nonsense words, verbal fluency (semantic and phonetic), oromotor sequences, and sentence comprehension	1 study(46)	1 study(43)<br><br>**Martin 2019:** left-sided strokes predispose children to contralateral auditory neglect and right-sided strokes predispose children to bilateral auditory neglect	1 study(39)<br><br>**Ballantyne 2008:** visual field defects are common (n=7, 26%) after perinatal stroke	<u>**Seizures**</u><br>8 studies(39, 42, 43, 45, 46)<br><br>5 comparable studies(39, 42, 43, 45, 46)<br>Combined incidence of seizures: 40.1% (95%CI: 26.8, 53.3) I^2=56%

Confidential: For Review Only

		had poorer neuropsychological scores.<br><br>**Northam 2018:** most children are in mainstream education (n=28, 93%) but many require additional support (n=12, 40%)							
Meningitis	3 studies(47-49)<br>Not comparable<br><br>All reported increased risk of neurodevelopmental impairment<br><br>**Bedford 2011:** increased prevalence of neuromotor disability (n=45, 16% vs. n=2, 0.1%)<br><br>**Stevens 2003:** Risk of severe disability seen in Bedford 2011 at 5 years of age persisted until 9-10 years (n=12, 10.8% vs. n=0, 0%)<br><br>**Horvath-Puho 2021:** increased risk of any neurodevelopmental impairment after GBS	1 study(49)<br><br>**Stevens 2003:** significantly lower mean cognitive scores (mean 88.8 (95%CI: 85, 92) vs. mean 99.4 (95%CI: 97, 102))	1 study(49)<br><br>**Stevens 2003:** significantly higher motor impairment scores (mean 7.1 (95%CI: 5.9, 8.5) vs. mean 5 (95%CI: 4.3, 5.8))	0 studies	0 studies	2 studies(49, 72)<br><br>**Martinez Cruz 2008:** increased odds of neonatal meningitis amongst preterm infants with sensorineural hearing loss OR 4.37 (95%CI: 1.7, 10.9<br><br>**Stevens 2003:** 3.6% (n=4) had hearing loss compared to none in the control group.	1 study(49)<br><br>**Stevens 2003:** Bilateral visual impairment was common after neonatal meningitis (n=18, 17%)	

Confidential: For Review Only

	meningitis in the Netherlands RR 5.30 (95%CI: 2·57, 10·89) and Denmark RR 7.80 (95%CI: 4·42, 13·77) at 5 years of age persisting to 11 years in the Netherlands RR 2.99 (95%CI: 1.83, 4.88) and 15 years in Denmark RR 3.15 (95%CI: 1.82, 5,46)							
HIE	0 studies	3 studies(30, 50, 51) (two of the same population)<br><br>Not comparable<br><br>**Koc 2016:** preterm infants with HIE significantly more likely to have below average IQ scores (n=8, 89% vs. n=24, 30% p=0.001)<br><br>**Lee-Kelland 2020 and Tonks 2019:** report lower full scale IQ scores after moderate to severe HIE (mean difference −13.62 (95%CI: −20.53, −6.71)) and poorer perceptual reasoning, working memory and processing speed. Children with previous HIE more likely to receive additional classroom support OR 10 (95%CI: 1.16, 86)	2 studies(50, 51) (of the same population)<br><br>**Lee-Kelland 2020 and Tonks 2019:** significantly lower motor scores (mean difference −2.12 (95%CI: −3.93, −0.30)) after moderate-severe HIE (for children without cerebral palsy)	2 studies(50, 51) (of the same population)<br><br>**Lee-Kelland 2020 and Tonks 2019:** significantly lower verbal comprehension scores (mean difference −8.8 (95%CI: −14.25, −3.34)) after moderate-severe HIE.	2 studies(50, 51) (of the same population)<br><br>**Lee-Kelland 2020 and Tonks 2019:** higher behavioural difficulty scores (median score 12 IQR (6.5, 13.5 vs. median score 6 IQR (2.25, 10) p=0.005)	0 studies	0 studies	
Kernicterus	0 studies							

Confidential: For Review Only

Study	Events	Total	Incidence	95% CI	Weight
Ballantyne, 2008	11	29	0.379	[0.207; 0.577]	22.2%
Kolk, 2011	9	21	0.429	[0.218; 0.660]	19.0%
Martin, 2019	4	21	0.190	[0.054; 0.419]	23.1%
Tilema, 2008	6	10	0.600	[0.262; 0.878]	12.5%
Trauner, 2001	17	34	0.500	[0.324; 0.676]	23.1%
**Overall**			**0.401**	**[0.268; 0.533]**	**100.0%**

$I^2 = 56\%$, $\tau^2 = 0.0124$, $p = 0.06$

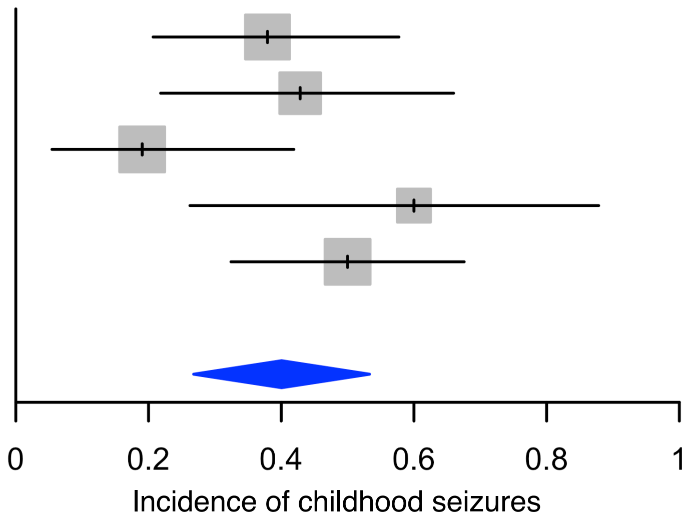

Study	Events	Total	Incidence	95% CI	Weight
Ballantyne, 2008	18	29	0.621	[0.423; 0.793]	19.9%
Gold, 2004	16	27	0.593	[0.388; 0.776]	19.6%
Kolk, 2011	19	21	0.905	[0.696; 0.988]	21.3%
Martin, 2019	13	21	0.619	[0.384; 0.819]	19.0%
Northam, 2017	9	30	0.300	[0.147; 0.494]	20.3%
**Overall**			**0.610**	**[0.392; 0.829]**	**100.0%**

$I^2 = 88\%$, $\tau^2 = 0.0545$, $p < 0.01$

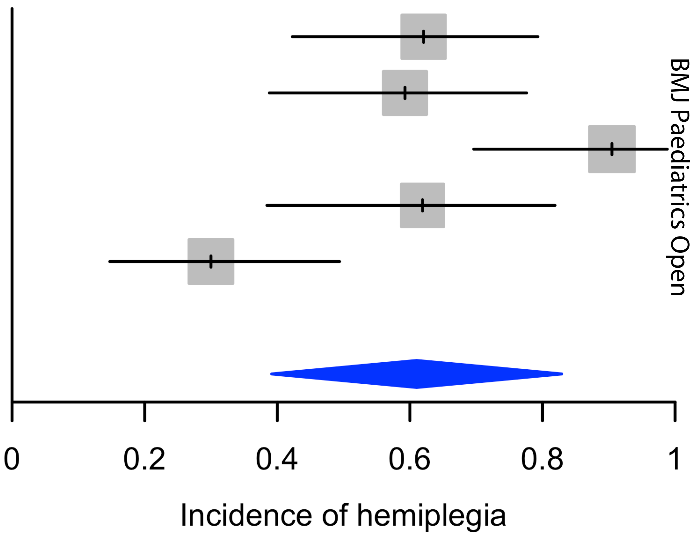

Confidential: For Review Only

	Perinatal stroke			Control				Mean Difference
**Study or Subgroup**	**Mean**	**SD**	**Total**	**Mean**	**SD**	**Total**	**Weight**	**IV, Random, 95% CI**
Ballantyne 2008	82.3	20.1	29	111.4	13.7	38	49.0%	-29.10 [-37.61, -20.59]
Northam 2017	91	16	30	104	14	40	51.0%	-13.00 [-20.18, -5.82]
**Total (95% CI)**			**59**			**78**	**100.0%**	**-20.88 [-36.66, -5.11]**

Heterogeneity: Tau² = 113.45; Chi² = 8.02, df = 1 (P = 0.005); I² = 88%
Test for overall effect: Z = 2.59 (P = 0.009)

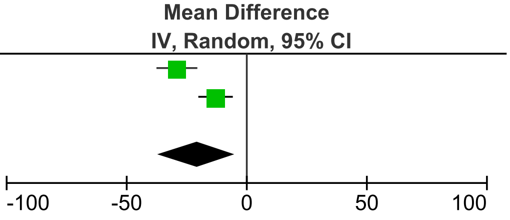

	Perinatal stroke			Control				Mean Difference
**Study or Subgroup**	**Mean**	**SD**	**Total**	**Mean**	**SD**	**Total**	**Weight**	**IV, Random, 95% CI**
Ballantyne 2008	78.4	16	29	105.8	11.9	38	50.3%	-27.40 [-34.34, -20.46]
Northam 2017	95	17	30	108	13	40	49.7%	-13.00 [-20.30, -5.70]
**Total (95% CI)**			**59**			**78**	**100.0%**	**-20.25 [-34.36, -6.13]**

Heterogeneity: $Tau^2 = 90.47$; $Chi^2 = 7.85$, df = 1 (P = 0.005); $I^2 = 87\%$
Test for overall effect: Z = 2.81 (P = 0.005)

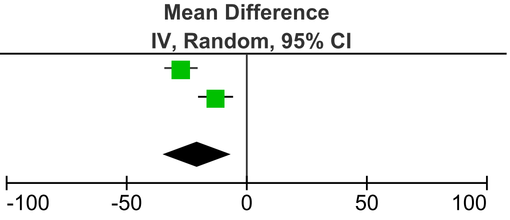

Confidential: For Review Only

BMJ Paediatrics Open

School-age outcomes of children after perinatal brain injury: a systematic review and meta-analysis

Journal:	*BMJ Paediatrics Open*
Manuscript ID	bmjpo-2022-001810.R1
Article Type:	Original research
Date Submitted by the Author:	29-Jan-2023
Complete List of Authors:	Rees, Philippa; University College London Institute of Child Health, Population policy and Practice<br>Callan, Caitriona; University of Oxford Nuffield Department of Primary Care Health Sciences<br>Chadda, Karan; Cambridge University Hospitals NHS Foundation Trust, Department of Paediatrics<br>Vaal, Meriel; University College London Institute of Child Health, University College London and Great Ormond Street Institute of Child Health<br>Diviney, James; Great Ormond Street Hospital for Children NHS Foundation Trust, Paediatric Intensive Care Unit<br>Sabti, Shahad; King's College London<br>Harnden, Fergus; Chelsea and Westminster Hospital NHS Foundation Trust<br>Gardiner, Julian; University College London Institute of Child Health, University College London and Great Ormond Street Institute of Child Health<br>Battersby, Cheryl; Imperial College London, Neonatal Medicine<br>Gale, Chris; Imperial College London, Neonatal Medicine<br>Sutcliffe, Alastair; University College London Institute of Child Health, University College London and Great Ormond Street Institute of Child Health
Keywords:	Epidemiology, Neonatology

SCHOLARONE™
Manuscripts

I, the Submitting Author has the right to grant and does grant on behalf of all authors of the Work (as defined in the below author licence), an exclusive licence and/or a non-exclusive licence for contributions from authors who are: i) UK Crown employees; ii) where BMJ has agreed a CC-BY licence shall apply, and/or iii) in accordance with the terms applicable for US Federal Government officers or employees acting as part of their official duties; on a worldwide, perpetual, irrevocable, royalty-free basis to BMJ Publishing Group Ltd ("BMJ") its licensees and where the relevant Journal is co-owned by BMJ to the co-owners of the Journal, to publish the Work in this journal and any other BMJ products and to exploit all rights, as set out in our licence.

The Submitting Author accepts and understands that any supply made under these terms is made by BMJ to the Submitting Author unless you are acting as an employee on behalf of your employer or a postgraduate student of an affiliated institution which is paying any applicable article publishing charge ("APC") for Open Access articles. Where the Submitting Author wishes to make the Work available on an Open Access basis (and intends to pay the relevant APC), the terms of reuse of such Open Access shall be governed by a Creative Commons licence – details of these licences and which Creative Commons licence will apply to this Work are set out in our licence referred to above.

Other than as permitted in any relevant BMJ Author's Self Archiving Policies, I confirm this Work has not been accepted for publication elsewhere, is not being considered for publication elsewhere and does not duplicate material already published. I confirm all authors consent to publication of this Work and authorise the granting of this licence.

Confidential: For Review Only

School-age outcomes of children after perinatal brain injury: a systematic review and meta-analysis

Philippa Rees[1] MPhil MBBCh, Caitriona Callan[2] MB BChir, Karan R Chadda[3] MB BChir, Meriel Vaal MRes MBChB[1], James Diviney[4] MB BChir, Shahad Sabti[5] MBBS, Fergus Harnden[6] MBChB, Julian Gardiner[1]PhD, Cheryl Battersby[7] PhD, Chris Gale[7] PhD, Alastair Sutcliffe[1] PhD

Affiliations:

1. Population Policy and Practice, Great Ormond Street UCL Institute of Child Health, London, UK.

2. Nuffield Department of Primary Care Health Sciences, University of Oxford.

3. Department of Paediatrics, Cambridge University Hospitals NHS Foundation Trust, Cambridge, UK.

4. Paediatric Intensive Care Unit, Great Ormond Street Hospital, London, UK

5. Kings College London, UK.

6. Chelsea and Westminster Hospital NHS Foundation Trust, London, UK.

7. Neonatal Medicine, School of Public Health, Faculty of Medicine, Imperial College London, London, UK.

Address correspondence to: Dr Philippa Rees, Population Policy Practice, UCL Institute of Child Health, 1st Floor 30 Guilford Street, London, WC1N 1EH, p.rees@ucl.ac.uk

Confidential: For Review Only

School-age outcomes of children after perinatal brain injury: a systematic review and meta-analysis

Background
Over 3,000 children suffer a perinatal brain injury in England every year according to national surveillance. The childhood outcomes of infants with perinatal brain injury are however unknown.

Methods
A systematic review and meta-analyses were undertaken of studies published between 2000-September 2021 exploring school-aged neurodevelopmental outcomes of children after perinatal brain injury compared to those without perinatal brain injury. The primary outcome was neurodevelopmental impairment which included cognitive, motor, speech and language, behavioural, hearing, or visual impairment after 5 years of age.

Results
This review included 42 studies. Preterm infants with intraventricular haemorrhage (IVH) grade 3-4 were found to have a three-fold greater risk of moderate-severe neurodevelopmental impairment at school age OR 3.69 (95%CI: 1.7, 7.98). Infants with perinatal stroke had an increased incidence of hemiplegia 61% (95%CI: 39.2, 82.9) and an increased risk of cognitive impairment (difference in full scale IQ -24.2 (95%CI: -30.73, -17.67) . Perinatal stroke was also associated with poorer academic performance; and lower mean receptive -20.88 (95%CI: -36.66, -5.11) and expressive language scores -20.25 (95%CI: -34.36, -6.13) on the CELF assessment. Studies reported an increased risk of persisting neurodevelopmental impairment at school age after neonatal meningitis. Cognitive impairment and special educational needs were highlighted after moderate-severe HIE. However, there were limited comparative studies providing school-aged outcome data across neurodevelopmental domains and few provided adjusted data. Findings were further limited by the heterogeneity of studies.

Conclusions
Longitudinal population studies exploring childhood outcomes after perinatal brain injury are urgently needed to better enable clinicians to prepare affected families, and to facilitate targeted developmental support to help affected children reach their full potential.

Confidential: For Review Only

School-age outcomes of children after perinatal brain injury: a systematic review and meta-analysis

What is already known on this topic

Thousands of children suffer a brain injury around the time of birth every year in England. Many of these injuries are associated with neurodevelopmental impairment at two years of age. However, two-year outcomes are not necessarily representative of later childhood outcomes and function, which are a priority for parents.

What this study adds

This review provides an overview of existing evidence of childhood outcomes after perinatal brain injury. It indicates that there is some evidence of on-going impairment throughout childhood for different types of perinatal brain injury but that there are considerable gaps in knowledge.

How this study might affect research, practice or policy

This review shows the need for detailed high-quality longitudinal population studies exploring childhood outcomes after perinatal brain injury

Confidential: For Review Only

School-age outcomes of children after perinatal brain injury: a systematic review and meta-analysis

Perinatal brain injuries can have wide-ranging deleterious consequences for children, families and broader society.(1-4) Over 3,000 infants experience perinatal brain injury in England annually[1] and the Department of Health and Social Care (DHSC) has committed to halving the rate of perinatal brain injuries by 2030 as part of the national maternity ambition.(5) To monitor progress towards this goal, a standardised definition of perinatal brain injury was developed.(6) The degree to which this definition captures and represents true perinatal brain injuries is unclear and requires us to look beyond the neonatal period.(6)

Focusing on the childhood outcomes of infants with perinatal brain injury provides a fuller understanding of the population captured by the DHSC definition. Despite their importance to families, school-age outcomes following neonatal care have been an overlooked research priority. Neonatal studies typically focus on two-year composite outcomes which may mask the true neurodevelopmental burden of injuries, and are known to be poorly predictive of future functioning.(7-10) As such, our understanding of childhood developmental trajectories after brain injuries – and whether any sequelae are fixed, stable or amenable to interventions – is limited. We therefore undertook a systematic review to explore school-age neurodevelopmental outcomes following perinatal brain injury.

Confidential: For Review Only

METHODS

Study selection

The review was conducted as per the pre-registered protocol (CRD 42021278572) and the PRISMA statement.(11) We included observational comparative studies exploring neurodevelopmental outcomes of children over five years of age after perinatal brain injury, published between 2000-September 2021 (Table 1). The DHSC definition of perinatal brain injuries used includes intraventricular haemorrhage, preterm white matter injuries, stroke, central nervous system infection, hypoxic ischaemic encephalopathy, and kernicterus diagnosed during the neonatal period.(6, 12) We did not include seizures in isolation. For inclusion, studies were required to have a non-brain injured comparator group. The primary outcome was neurodevelopmental impairment; secondary outcomes included motor, cognitive, speech and language, behavioural and neuropsychological, visual and hearing outcomes and seizures.

A search strategy incorporating 99 key terms and mesh headings was developed in Medline Ovid, adapted and run across 10 databases. Snowballing techniques were used to augment search sensitivity (Supplement 1 & 2). All titles were screened independently by two reviewers. The full-texts of all potentially relevant titles were retrieved, reviewed and their risk of bias assessed by two trained reviewers independently (PR, CC, MV, JD, SS). Disagreements were arbitrated by a third reviewer.

Data extraction and synthesis

Studies were stratified by brain injury type, sub-stratified by age of outcome assessment and outcome type, and summarised in a narrative synthesis. Where sufficient suitable data were available from contextually and clinically comparable studies, data were pooled in random

Confidential: For Review Only

effects meta-analyses using RevMan 5.4. Continuous data were pooled using the inverse variance method; dichotomous data were pooled using the Mantel-Haenszel method; and analysis data from studies which did not provide raw data were pooled with dichotomous data from other studies using the generic inverse variance method.(13) Where studies provided insufficient comparative data for a particular outcome, the combined incidence figures for that outcome within the brain injured population was calculated across studies using the Fisher exact test for binomial data.(14) Statistical heterogeneity was assessed using the I^2 statistic and substantial heterogeneity (>85%) was explored further in sub-group analyses.

Quality assessment

The Newcastle Ottawa Tool was used to assess risk of bias across three domains: population selection, the comparability of the 'brain injured' and 'non brain injured' comparator groups, and outcome assessment.(15) Studies were classed as poor, fair, or good for each domain and given an overall risk of bias classification.

Patient and Public Involvement

Patients or the public were not involved in the design or conduct of this review. However the review's findings will be used to shape the larger CHERuB study in partnership with our parent advisory panel.

Confidential: For Review Only

RESULTS

Searches identified 14,210 records and 42 studies were included (Figure 1). Studies focused on intraventricular haemorrhage (n=27), white matter injury (WMI) amongst preterm infants (n=15), perinatal stroke (n=8), neonatal meningitis (n=4), and HIE (n=3); these were not mutually exclusive (Supplement 3). Most studies were undertaken in the USA (n=10), the UK (n=8), the Netherlands (n=5) or Australia (n=4). These were prospective (n=27) or retrospective cohort studies (n=14). Included studies were deemed to be moderate (n=17) or low risk of bias (n=27) (Supplement 4).

Preterm injuries

The 29 studies exploring outcomes after IVH or WMI mostly included infants born <32 weeks' gestation (n=22) after the year 2000 (n=18) (Supplement 3). Most studies confirmed injury on ultrasound or MRI imaging (n=22) these were reviewed by radiologists (n=6), neonatologists (n=3) or both (n=1); 14 studies used the Papile classification; only 2 studies stratified results by laterality.

Nine studies explored neurodevelopmental impairment at 5-14 years of age after preterm brain injury including IVH (n=9) and WMI (n=6).(16-24) Two comparable studies highlighted a considerably increased pooled crude risk of moderate-severe neurodevelopmental impairment after IVH grade 3-4 at 8 years of age OR 3.69 (95%CI: 1.7, 7.98; 2 studies) I^2 = 0% (Figure 2, Table 2).(18, 21)

Six studies explored motor outcomes after IVH grade 3-4: they consistently highlighted an increased risk of motor impairment at 5-12 years of age.(21, 24-28) Additionally, two

Confidential: For Review Only

comparable studies reported an 8-fold increased crude risk of cerebral palsy after IVH grade 3-4 OR 8.13 (95%CI: 4.64, 14.22; 2 studies; 1,557 subjects) I^2=0% (Figure 3).

Cognitive outcomes at school-age after preterm brain injuries were reported by 16 studies using 25 different cognitive assessment tools - limiting the potential for meta-analysis (Supplement 3).(16, 17, 21, 22, 24-35) Educational outcomes were reported by 5 studies.(21, 22, 26, 30, 35)

Studies consistently reported lower cognitive scores at school-age following IVH grade 3-4. (16, 21, 22, 25-27, 31, 35) Hollebrandse 2021 reported an increased risk of cognitive impairment at 8 years of age OR 2.68 (95%CI: 1.21, 5.94).(26) Van de Bor 2000 and Hollebrandse 2021 reported that the cognitive impact of IVH grade 3-4 affected educational needs.(22, 26) Van de Bor 2000 reported increased special educational needs at 5, 9 and 14 years: the adjusted risk at 14 years of age was marked, aOR 3.99 (95%CI: 1.36, 11.69).(22) Studies reported no significant differences in language scores after IVH grade 3-4.(21, 22) However, an association with reading OR 3.62 (95%CI: 1.59, 8.24), spelling OR 4.48 (95%CI: 1.8, 11.2), and arithmetic OR 2.79 (95%CI: 1.2, 6.48) impairment was demonstrated.(26) Most studies highlighted cognitive effects after WMI.(17, 30, 33, 35)

Studies exploring behavioural outcomes after IVH 3-4 did not find any associations with attention deficits, conduct issues or autism spectrum disorder (Table 2).(16, 25, 36) However, there was conflicting evidence around the mental health effects of WMI.(17, 37)

Confidential: For Review Only

Studies exploring hearing impairment after IVH and/or WMI were small or not comparable. 10 studies explored visual impairment after IVH or WMI, 4 provided meaningful outcome data.(16, 21-23, 27, 28, 33, 34, 38, 39) An increased prevalence of visual impairment after IVH grade 3-4 (45.4% and 90.9%) compared to controls (7.5%) was reported in addition to significantly lower visual motor integration scores.(27)

Perinatal stroke

Eight comparative studies explored school-age outcomes after perinatal stroke, these included 177 children with perinatal stroke (100 left-sided and 54 right-sided – not all studies specified laterality) and 232 comparator children (Supplement 3).(40-47) Infants' gestation age was largely unspecified. Five studies presented a combined incidence of childhood seizures after perinatal stroke of 40.1% (95%CI: 26.8-53.3%; 5 studies; 115 subjects) I^2=56% (Supplement 5).(40, 43, 44, 46, 47) The combined incidence of hemiparesis after perinatal stroke was 61% (95%CI: 39.2, 82.9 I^2=88%). There was considerable heterogeneity across studies, and likely detection bias (Supplement 6).(40, 42-45)

Five studies identified a significant combined mean difference in full scale IQ scores at 7-13 years of age after perinatal stroke: -24.2 (95%CI: -30.73, -17.67; 5 studies; 296 subjects) I^2=80% (Figure 4).(40, 42, 45-47) There was heterogeneity across studies in terms of assessment timing, assessment tools, and combining those with left and right-sided strokes.

Differences in stroke laterality partially explained the heterogeneity. The combined mean difference in full scale IQ following left-sided strokes was -26.01 (95%CI: -29.1, -22.93; 2 studies; 113 subjects) I^2=0%; compared to -26.7 (95%CI: -39.38. -14.02; 2 studies; 99

Confidential: For Review Only

subjects) I^2=76% for right-sided strokes. No significant differences in cognitive outcomes were found by laterality.(40, 42, 45-47)

Kolk 2011 reported significantly lower scores across all NEPSY domains other than executive function after perinatal stroke, including attention, visuo-spacial function, memory, and learning.(43)

Two studies presented educational outcomes after perinatal stroke. Although Northam 2018 found that most children with perinatal stroke were in mainstream education (n=28, 93%), they also highlighted that additional educational support was often required (n=12, 40%). This was in keeping with Ballantyne 2008 reporting lower mean scores for reading (85 (16.1) vs. 113 (13.3); p<0.0001), spelling (82.5 (18.2) vs. 106.2 (15.9) p=0.001) and arithmetic (91.5 (10.2) vs. 111.9 (11.2) p<0.0001) after perinatal stroke compared to controls at 7-8 years of age, persisting on re-assessment at 10-12 years.

Kolk 2011 reported significantly lower scores compared to controls across most NEPSY language domains following perinatal stroke.(43) Significantly lower receptive and expressive mean language scores on the CELF assessment were also reported across studies: -20.88 (95%CI: -36.66, -5.11; 2 studies; 137 subjects) I^2=88% and -20.25 (95%CI: -34.36, -6.13; 2 studies; 137 subjects) I^2=87% respectively (Supplement 7, 8).(40, 45) Statistical heterogeneity may have been as a result of studies combining left and right-sided strokes and the varying age of outcome assessment. Studies highlighted that deficits in receptive language scores present at 7-8 years persisted at 10-12 years but that expressive language scores improved (p=0.012).(40, 41)

Meningitis

https://mc.manuscriptcentral.com/bmjpo

Confidential: For Review Only

Studies consistently reported an increased risk of neurodevelopmental impairment after neonatal meningitis (Table 2).(48-50) An increased likelihood of neuromotor disability at 5 years of age (n=45/274, 16%) compared to controls (n=2/1391, 0.1%) was reported (Supplement 3).(48) On re-assessment of the same population at 9-10 years, this increased risk of severe disability persisted (n=12, 10.8% compared to n=0, 0%).(50) An increased risk of any neurodevelopmental impairment at 5 years after neonatal *Group-B Streptococcal* meningitis was also reported in the Netherlands, RR 5.30 (95%CI: 2·57-10·89), and in Denmark, RR 7.80 (95%CI: 4·42-13·77).(49) This increased risk persisted on subsequent assessment: at 11 years of age in the Netherlands, RR 2.99 (95%CI: 1.83, 4.88) and at 15 years of age in Denmark RR, 3.15 (95%CI: 1.82, 5,46).(49)

Hypoxic-ischaemic encephalopathy

Two comparative studies (of the same cohort) explored outcomes of term-born infants with moderate-severe HIE, but without cerebral palsy, at school age (Supplement 3).(51, 52) They highlighted significantly lower full scale IQ scores after HIE (mean difference −13.62 (95%CI: −20.53 to −6.71)).(51) This difference in cognition was also seen for perceptual reasoning, working memory, and processing speed. Children with HIE were also more likely than controls to receive additional classroom support: OR 10 (95%CI: 1.16, 86) although the confidence interval for this risk estimate was wide.(51) Children with HIE (without cerebral palsy) also had significantly lower motor scores (mean difference −2.12 (95%CI: −3.93, −0.30)) and verbal comprehension scores (mean difference −8.8 (95%CI: −14.25, −3.34)).(51) They were also noted to have higher behavioural difficulty scores especially for emotional problems.(51)

https://mc.manuscriptcentral.com/bmjpo
Confidential: For Review Only

DISCUSSION

This review brings together the existing evidence on the later childhood outcomes of infants with perinatal brain injury. Although 42 studies are included, small study populations, limited data on injury severity and laterality, and the heterogeneity of outcome measures limited the potential power of results. However, studies demonstrate a three-fold higher risk of moderate-severe neurodevelopmental impairment at school age following IVH grade 3-4. Studies consistently report cognitive impairment after IVH grade 3-4 but suggest that speech and language is relatively preserved. A higher risk of hemiplegia, cognitive impairment and poorer academic performance after perinatal stroke is reported in addition to poorer receptive and expressive language scores. Studies report a higher risk of persisting neurodevelopmental impairment after neonatal meningitis – however few studies address this question. Few comparative studies explore school-age outcomes after HIE.

In following our a priori protocol only comparative studies were included. This was with a view to enabling inferential analyses and adjustment for key confounders such as gestation. Unfortunately due to this strict inclusion criterion many pertinent non-comparative studies were excluded.

Heterogeneity in terms of outcomes assessed, outcome assessment tools, and timing of outcome assessment limited the comparability of studies and the potential for meta-analyses. Several meta-analyses included low numbers of studies, reducing the reliability of the I^2 statistic.(53) This review was also limited by the size of available studies and how studies presented data for extraction. Few studies presented adjusted data or explored childhood trajectories after perinatal brain injury.

Confidential: For Review Only

Previous reviews were limited by a lack of comparable studies, heterogeneity, the inclusion of much older cohorts, or by including non-comparative studies.(4, 54-56) Whilst this review was also limited by studies' heterogeneity and the quality of available data, new and important findings - for example the risk of neurodevelopmental impairment - at school age after IVH 3-4 were identified. Our finding of a higher risk of cerebral palsy after IVH and motor impairments after preterm brain injuries is echoed by previous studies.(54, 55, 57)

Lynch 2001 highlighted that 60% of infants have neurological sequelae that emerge over time following perinatal stroke. This was in-keeping with our findings of a higher risk of hemiparesis, cognitive impairment, and speech and language impairment.(58) Several non-comparative population-based studies also mirror these findings.(59-62)

Although previous reviews highlight an increased risk of various neurodevelopmental impairments after neonatal meningitis in early childhood – we are unaware of any focusing on school-age outcomes after neonatal meningitis.(4, 63)

The review's findings of potential on-going impairments across cognitive, speech and language, and behavioural domains - in addition to a need for increased school support – after HIE are mirrored by other studies.(64-68) Shankaran 2012 and Azzopardi 2014 highlight on-going neurodevelopmental sequelae at school age amongst children who received therapeutic hypothermia for moderate-severe HIE.(64, 65, 67)

Implications

Considerable gaps in the evidence are highlighted, particularly around the risk of specific outcomes following different types of injury, the precision around risk estimates, the impact

https://mc.manuscriptcentral.com/bmjpo

Confidential: For Review Only

of different factors (such as injury laterality), and the developmental trajectories of these children. This information is key to prepare families for the future, inform enhanced developmental surveillance, and enable targeted multidisciplinary support to help affected children to reach their full potential. As such, this review highlights a pressing need for high-quality, comparative studies which use the 'Core Outcomes In Neonatology' to explore long-term outcomes after perinatal brain injury and permit future meta-analyses.(10) Additionally, to meet the DHSC ambition to reduce perinatal brain injury, real-time longitudinal population data, extending beyond the neonatal period to childhood, are necessary as the current definition is limited to 'indicators' of injury from the neonatal period. This could be achieved through linkage of existing population datasets within the UK.

CONCLUSION

This review provides an overview of existing evidence of the impact of perinatal brain throughout childhood. Studies' heterogeneity significantly limited the potential for evidence synthesis.

Confidential: For Review Only

Funding/support: This review was supported by an NIHR Doctoral Fellowship award (NIHR301457)

Role of funder/sponsor (if any): The NIHR had no role in the design or conduct of the review.

Conflict of interest disclosures (includes financial disclosures): CG is funded by the United Kingdom Medical Research Council (MRC) through a Transition Support Award. In the past 5 years He has received support from Chiesi Pharmaceuticals to attend an educational conference, and has been investigator on received research grants from Medical Research Council, National Institute of Health Research, Canadian Institute of Health Research, Department of Health in England, Mason Medical Research Foundation, Westminster Medical School Research Trust and Chiesi Pharmaceuticals. CB is funded by the United Kingdom National Institute of Health Research (NIHR) Advanced Fellowship Award.

Contributors' statement

Dr Rees conceptualised and designed the review, reviewed and appraised studies, undertook data extraction and synthesis, drafted the initial manuscript, and reviewed and revised the manuscript.

Dr Callan conceptualized and designed the review, designed and oversaw the search strategy, reviewed and appraised studies, undertook data extraction, and reviewed and revised the manuscript.

Dr Chadda reviewed and appraised studies, undertook data extraction, and reviewed and revised the manuscript.

Dr Vaal reviewed and appraised studies, undertook data extraction, and reviewed and revised the manuscript.

Dr Diviney reviewed and appraised studies, undertook data extraction, and reviewed and revised the manuscript.

Dr Sabti reviewed and appraised studies, undertook data extraction, and reviewed and revised the manuscript.

Dr Harnden reviewed and appraised studies, undertook data extraction, and reviewed and revised the manuscript.

Dr Gardiner was the lead statistician for the review, he advised on and oversaw the data analysis, and reviewed and revised the manuscript.

Dr Battersby oversaw and supervised the review and critically revised the manuscript for important intellectual content.

Confidential: For Review Only

Professor Gale oversaw and supervised the review and critically revised the manuscript for important intellectual content.
Professor Sutcliffe oversaw and supervised the review and critically revised the manuscript for important intellectual content.
All authors approve the final manuscript as submitted and agree to be accountable for all aspects of the work.

Additional Contributions: The authors would like to thank Dr Roxanna Short for creating the figures in supplemental file 5 and 6.

Confidential: For Review Only

Figure 1: PRISMA flow diagram

Figure 2: Crude risk of neurodevelopmental impairment at 8 years of age after IVH grade 3-4

Figure 3: Crude risk of cerebral palsy after IVH grade 3-4

Figure 4: Pooled mean difference in IQ scores at 7-13 years between those with and without perinatal stroke

Confidential: For Review Only

References

1. Lawn JE, Blencowe H, Oza S, You D, Lee AC, Waiswa P, et al. Every Newborn: progress, priorities, and potential beyond survival. Lancet. 2014;384(9938):189-205.
2. Lee AC, Kozuki N, Blencowe H, Vos T, Bahalim A, Darmstadt GL, et al. Intrapartum-related neonatal encephalopathy incidence and impairment at regional and global levels for 2010 with trends from 1990. Pediatric Research. 2013;74(S1):50-72.
3. Liu L, Oza S, Hogan D, Chu Y, Perin J, Zhu J, et al. Global, regional, and national causes of under-5 mortality in 2000–15: an updated systematic analysis with implications for the Sustainable Development Goals. Lancet. 2016;388(10063):3027-35.
4. Mwaniki MK, Atieno M, Lawn JE, Newton CR. Long-term neurodevelopmental outcomes after intrauterine and neonatal insults: a systematic review. Lancet. 2012;379(9814):445-52.
5. Department of Health & Social Care. New ambition to halve rate of stillbirths and infant deaths 2015 [Available from: https://www.gov.uk/government/news/new-ambition-to-halve-rate-of-stillbirths-and-infant-deaths.
6. Gale C, Statnikov Y, Jawad S, Uthaya SN, Modi N, Brain Injuries expert working group. Neonatal brain injuries in England: population-based incidence derived from routinely recorded clinical data held in the National Neonatal Research Database. Archives of Disease in Childhood - Fetal and Neonatal Edition. 2018;103(4):F301-F6.
7. Marlow N. Measuring neurodevelopmental outcome in neonatal trials: a continuing and increasing challenge. Archives of Disease in Childhood-Fetal and Neonatal Edition. 2013;98(6):F554-F8.
8. Marlow N, Wolke D, Bracewell MA, Samara M. Neurologic and developmental disability at six years of age after extremely preterm birth. New England Journal of Medicine. 2005;352(1):9-19.
9. Webbe J, Brunton G, Ali S, Longford N, Modi N, Gale C. Parent, patient and clinician perceptions of outcomes during and following neonatal care: a systematic review of qualitative research. BMJ Paediatrics Open. 2018;2(1).
10. Webbe JWH, Duffy JMN, Afonso E, Al-Muzaffar I, Brunton G, Greenough A, et al. Core outcomes in neonatology: development of a core outcome set for neonatal research. Archives of Disease in Childhood-Fetal and Neonatal Edition. 2019.
11. Page MJ, McKenzie JE, Bossuyt PM, Boutron I, Hoffmann TC, Mulrow CD, et al. The PRISMA 2020 statement: an updated guideline for reporting systematic reviews. Systematic Reviews. 2021;10(1):1-11.
12. Gale C, Stanikov E, Jawad S, Uthaya S, Modi N. Brain injury occurring during or soon after birth: a report for the national maternity ambition commissioned by the Department of Health. Imperial College london; 2017.
13. Higgins JPT, Thomas J, Chandler J, Cumpston M, Li T, Page MJ, et al. Cochrane handbook for systematic reviews of interventions version 6.2 (updated February 2021): Cochrane; 2021. Available from: www.training.cochrane.org/handbook.
14. Mehanna H, Al-Maqbili T, Carter B, Martin E, Campain N, Watkinson J, et al. Differences in the recurrence and mortality outcomes rates of incidental and nonincidental papillary thyroid microcarcinoma: a systematic review and meta-analysis of 21 329 person-years of follow-up. Journal of Clinical Endocrinology and Metabolism. 2014;99(8):2834-43.
15. Wells GA, Shea B, O'Connell D, Peterson J, Welch V, Losos M, et al. The Newcastle-Ottawa Scale (NOS) for assessing the quality of nonrandomised studies in meta-analyses. Oxford; 2000.

Confidential: For Review Only

16. Adant I, Miserez M, Naulaers G, Carkeek K, Ortibus E, Aerts R, et al. Long-term outcomes of very low birth weight infants with spontaneous intestinal perforation: A retrospective case-matched cohort study. Journal of Pediatric Surgery. 2019;54(10):2084-91.
17. Campbell H, Check J, Kuban KC, Leviton A, Joseph RM, Frazier JA, et al. Neonatal cranial ultrasound findings among infants born extremely preterm: associations with neurodevelopmental outcomes at ten years of age. The Journal of Pediatrics. 2021.
18. Cheong JL, Lee KJ, Boland RA, Spittle AJ, Opie GF, Burnett AC, et al. Changes in long-term prognosis with increasing postnatal survival and the occurrence of postnatal morbidities in extremely preterm infants offered intensive care: a prospective observational study. The Lancet Child & Adolescent Health. 2018;2(12):872-9.
19. Neubauer A-P, Voss W, Kattner E. Outcome of extremely low birth weight survivors at school age: the influence of perinatal parameters on neurodevelopment. European Journal of Pediatrics. 2008;167(1):87-95.
20. Piris Borregas S, Torres Valdivieso MJ, Martín-Arriscado C, de la Cruz Bértolo J, Sierra García P, Pallás Alonso CR. Model that predicted death or disabilities in premature infants was valid at seven years of age. Acta Paediatrica. 2019;108(7):1245-9.
21. Sherlock RL, Anderson PJ, Doyle LW, Group VICS. Neurodevelopmental sequelae of intraventricular haemorrhage at 8 years of age in a regional cohort of ELBW/very preterm infants. Early Human Development. 2005;81(11):909-16.
22. Van de Bor M, den Ouden L. School performance in adolescents with and without periventricular-intraventricular hemorrhage in the neonatal period. Seminars in Perinatology. 2004;28(4):295-303.
23. Vollmer B, Roth S, Baudin J, Stewart AL, Neville BG, Wyatt JS. Predictors of long-term outcome in very preterm infants: gestational age versus neonatal cranial ultrasound. Pediatrics. 2003;112(5):1108-14.
24. Hintz SR, Vohr BR, Bann CM, Taylor HG, Das A, Gustafson KE, et al. Preterm neuroimaging and school-age cognitive outcomes. Pediatrics. 2018;142(1).
25. Brouwer A, Van Stam C, Venema MU, Koopman C, Groenendaal F, De Vries L. Cognitive and neurological outcome at the age of 5–8 years of preterm infants with post-hemorrhagic ventricular dilatation requiring neurosurgical intervention. Neonatology. 2012;101(3):210-6.
26. Hollebrandse NL, Spittle AJ, Burnett AC, Anderson PJ, Roberts G, Doyle LW, et al. School-age outcomes following intraventricular haemorrhage in infants born extremely preterm. Archives of Disease in Childhood-Fetal and Neonatal Edition. 2021;106(1):4-8.
27. Klebermass-Schrehof K, Czaba C, Olischar M, Fuiko R, Waldhoer T, Rona Z, et al. Impact of low-grade intraventricular hemorrhage on long-term neurodevelopmental outcome in preterm infants. Child's Nervous System. 2012;28(12):2085-92.
28. Vollmer B, Roth S, Riley K, O'Brien F, Baudin J, De Haan M, et al. Long-term neurodevelopmental outcome of preterm children with unilateral cerebral lesions diagnosed by neonatal ultrasound. Early Human Development. 2006;82(10):655-61.
29. Hirvonen M, Ojala R, Korhonen P, Haataja P, Eriksson K, Rantanen K, et al. Intellectual disability in children aged less than seven years born moderately and late preterm compared with very preterm and term-born children–a nationwide birth cohort study. Journal of Intellectual Disability Research. 2017;61(11):1034-54.
30. Jansen L, Peeters-Scholte C, Wiggers-de Bruine S, van den Berg-Huysmans A, van Klink J, van Steenis A, et al. Classroom-evaluated school performance at nine years of age after very preterm birth. Early Human Development. 2020;140:104834.
31. Koç Ö, Kavuncuoğlu S, Ramoğlu MG, Aldemir E, Aktalay A, Eras Z. School performance and neurodevelopment of very low birth weight preterm infants: first report from Turkey. Journal of Child Neurology. 2016;31(2):170-6.

Confidential: For Review Only

32. Pittet M-P, Mürner-Lavanchy I, Adams M, Bickle-Graz M, Pfister R, Natalucci G, et al. Neurodevelopmental outcome at early school age in a Swiss national cohort of very preterm children. Swiss Medical Weekly. 2019;149:w20084.
33. Van den Hout B, Stiers P, Haers M, van der Schouw YT, Eken P, Vandenbussche E, et al. Relation between visual perceptual impairment and neonatal ultrasound diagnosis of haemorrhagic–ischaemic brain lesions in 5-year-old children. Developmental Medicine and Child Neurology. 2000;42(6):376-86.
34. Vollmer B, Roth S, Riley K, Sellwood MW, Baudin J, Neville BG, et al. Neurodevelopmental outcome of preterm infants with ventricular dilatation with and without associated haemorrhage. Developmental Medicine and Child Neurology. 2006;48(5):348-52.
35. Kiechl-Kohlendorfer U, Ralser E, Pupp Peglow U, Pehboeck-Walser N, Fussenegger B. Early risk predictors for impaired numerical skills in 5-year-old children born before 32 weeks of gestation. Acta Paediatrica. 2013;102(1):66-71.
36. Davidovitch M, Kuint J, Lerner-Geva L, Zaslavsky-Paltiel I, Rotem RS, Chodick G, et al. Postnatal steroid therapy is associated with autism spectrum disorder in children and adolescents of very low birth weight infants. Pediatric Research. 2020;87(6):1045-51.
37. Whitaker AH, Feldman JF, Lorenz JM, McNicholas F, Fisher PW, Shen S, et al. Neonatal head ultrasound abnormalities in preterm infants and adolescent psychiatric disorders. Archives of General Psychiatry. 2011;68(7):742-52.
38. Hreinsdottir J, Fredriksson Kaul Y, Hellström-Westas L, Rosander K, von Hofsten C, Holmström G. Impaired cognitive ability at 2.5 years predicts later visual and ophthalmological problems in children born very preterm. Acta Paediatrica. 2018;107(5):822-30.
39. Kaur A, Luu TM, Shah PS, Ayoub A, Auger N. Neonatal intraventricular hemorrhage and hospitalization in childhood. Pediatric Neurology. 2020;103:35-42.
40. Ballantyne AO, Spilkin AM, Hesselink J, Trauner DA. Plasticity in the developing brain: intellectual, language and academic functions in children with ischaemic perinatal stroke. Brain. 2008;131(11):2975-85.
41. Ballantyne AO, Spilkin AM, Trauner DA. Language outcome after perinatal stroke: does side matter? Child Neuropsychology. 2007;13(6):494-509.
42. Gold JJ, Trauner DA. Hippocampal volume and memory performance in children with perinatal stroke. Pediatric Neurology. 2014;50(1):18-25.
43. Kolk A, Ennok M, Laugesaar R, Kaldoja M-L, Talvik T. Long-term cognitive outcomes after pediatric stroke. Pediatric Neurology. 2011;44(2):101-9.
44. Martin K, Trauner DA. Auditory neglect in children following perinatal stroke. Behavioural Brain Research. 2019;359:878-85.
45. Northam GB, Adler S, Eschmann KC, Chong WK, Cowan FM, Baldeweg T. Developmental conduction aphasia after neonatal stroke. Annals of Neurology. 2018;83(4):664-75.
46. Tillema J-M, Byars AW, Jacola LM, Schapiro MB, Schmithorst VJ, Szaflarski JP, et al. Reprint of "Cortical reorganization of language functioning following perinatal left MCA stroke"[Brain and Language 105 (2008) 99–111]. Brain and Language. 2008;106(3):184-94.
47. Trauner DA, Nass R, Ballantyne A. Behavioural profiles of children and adolescents after pre-or perinatal unilateral brain damage. Brain. 2001;124(5):995-1002.
48. Bedford H, de Louvois J, Halket S, Peckham C, Hurley R, Harvey D. Meningitis in infancy in England and Wales: follow up at age 5 years. BMJ. 2001;323(7312):533.
49. Horváth-Puhó E, van Kassel MN, Gonçalves BP, de Gier B, Procter SR, Paul P, et al. Mortality, neurodevelopmental impairments, and economic outcomes after invasive group B streptococcal disease in early infancy in Denmark and the Netherlands: a national matched cohort study. The Lancet Child & Adolescent Health. 2021;5(6):398-407.

Confidential: For Review Only

50. Stevens J, Eames M, Kent A, Halket S, Holt D, Harvey D. Long term outcome of neonatal meningitis. Archives of Disease in Childhood-Fetal and Neonatal Edition. 2003;88(3):F179-F84.
51. Lee-Kelland R, Jary S, Tonks J, Cowan FM, Thoresen M, Chakkarapani E. School-age outcomes of children without cerebral palsy cooled for neonatal hypoxic–ischaemic encephalopathy in 2008–2010. Archives of Disease in Childhood-Fetal and Neonatal Edition. 2020;105(1):8-13.
52. Tonks J, Cloke G, Lee-Kelland R, Jary S, Thoresen M, Cowan FM, et al. Attention and visuo-spatial function in children without cerebral palsy who were cooled for neonatal encephalopathy: a case-control study. Brain Injury. 2019;33(7):894-8.
53. von Hippel PT. The heterogeneity statistic I2 can be biased in small meta-analyses. BMC medical research methodology. 2015;15(1):1-8.
54. Gotardo JW, Volkmer NdFV, Stangler GP, Dornelles AD, Bohrer BBdA, Carvalho CG. Impact of peri-intraventricular haemorrhage and periventricular leukomalacia in the neurodevelopment of preterms: A systematic review and meta-analysis. PloS one. 2019;14(10):e0223427.
55. Mukerji A, Shah V, Shah PS. Periventricular/intraventricular hemorrhage and neurodevelopmental outcomes: a meta-analysis. Pediatrics. 2015;136(6):1132-43.
56. Magai DN, Karyotaki E, Mutua AM, Chongwo E, Nasambu C, Ssewanyana D, et al. Long-term outcomes of survivors of neonatal insults: A systematic review and meta-analysis. PloS one. 2020;15(4):e0231947.
57. Rees P, Callan C, Chadda KR, Vaal M, Diviney J, Sabti S, et al. Preterm brain injury and neurodevelopmental outcomes: a meta-analysis. Pediatrics. 2022;Nov 4:e2022057442.
58. Lynch JK, Nelson KB. Epidemiology of perinatal stroke. Current Opinion in Pediatrics. 2001;13(6):499-505.
59. Lee J, Croen LA, Lindan C, Nash KB, Yoshida CK, Ferriero DM, et al. Predictors of outcome in perinatal arterial stroke: a population-based study. Annals of Neurology. 2005;58(2):303-8.
60. Grunt S, Mazenauer L, Buerki SE, Boltshauser E, Mori AC, Datta AN, et al. Incidence and outcomes of symptomatic neonatal arterial ischemic stroke. Pediatrics. 2015;135(5):e1220-e8.
61. Husson B, Hertz-Pannier L, Renaud C, Allard D, Presles E, Landrieu P, et al. Motor outcomes after neonatal arterial ischemic stroke related to early MRI data in a prospective study. Pediatrics. 2010;126(4):e912-e8.
62. Wusthoff CJ, Kessler SK, Vossough A, Ichord R, Zelonis S, Halperin A, et al. Risk of later seizure after perinatal arterial ischemic stroke: a prospective cohort study. Pediatrics. 2011;127(6):e1550-e7.
63. Kohli-Lynch M, Russell NJ, Seale AC, Dangor Z, Tann CJ, Baker CJ, et al. Neurodevelopmental impairment in children after group B streptococcal disease worldwide: systematic review and meta-analyses. Clinical Infectious Diseases. 2017;65(suppl_2):S190-S9.
64. Shankaran S, Pappas A, McDonald SA, Vohr BR, Hintz SR, Yolton K, et al. Childhood outcomes after hypothermia for neonatal encephalopathy. New England Journal of Medicine. 2012;366(22):2085-92.
65. Azzopardi D, Strohm B, Marlow N, Brocklehurst P, Deierl A, Eddama O, et al. Effects of hypothermia for perinatal asphyxia on childhood outcomes. New England Journal of Medicine. 2014;371(2):140-9.
66. Jary S, Lee-Kelland R, Tonks J, Cowan FM, Thoresen M, Chakkarapani E. Motor performance and cognitive correlates in children cooled for neonatal encephalopathy without cerebral palsy at school age. Acta Paediatrica. 2019;108(10):1773-80.

Confidential: For Review Only

67. Natarajan G, Shankaran S, Pappas A, Bann C, Tyson JE, McDonald S, et al. Functional status at 18 months of age as a predictor of childhood disability after neonatal hypoxic-ischemic encephalopathy. Developmental Medicine & Child Neurology. 2014;56(11):1052-8.
68. Guillet R, Edwards AD, Thoresen M, Ferriero DM, Gluckman PD, Whitelaw A, et al. Seven-to eight-year follow-up of the CoolCap trial of head cooling for neonatal encephalopathy. Pediatric Research. 2012;71(2):205-9.

Confidential: For Review Only

Table 1 Inclusion and exclusion criteria

Inclusion Criteria	Exclusion Criteria
Peer-reviewed observational studies (cohort, case-control, cross-sectional)	Non-comparative studies; opinions; commentaries; reviews; case-reports; lab studies
Studies in all languages	Studies where the population includes adults and children and the data for children cannot be extracted
Studies published after 2000	Studies focused on children with IVH grade 1-2, neonatal seizures, hypoglycaemic brain injury, or neonatal abstinence syndrome
Children with a diagnosis of brain injury occurring at or around the time of birth (including during the neonatal period) as defined by the DHSC (including those with any white matter injury but not including those with isolated seizures)	Studies which include infants with brain injuries diagnosed during the neonatal and infancy period where most were diagnosed outside of the neonatal period
Studies including infants with moderate to severe HIE born in the post therapeutic hypothermia era (i.e. where infants received therapeutic hypothermia)	Studies including infants with moderate-severe HIE born during the pre-therapeutic hypothermia era or in low- or middle-income countries that do not offer therapeutic hypothermia
Studies focused on school-aged neurodevelopmental outcomes (of children between 5-18 years of age) including:<br>**Primary outcome(s):**<br>Neurodevelopmental impairment, as defined by authors (including direct testing, clinical record review, and parental interview/ survey)<br><br>**Secondary outcome(s):**<br>1. Any cognitive impairment, as defined by authors (direct testing)<br><br>2. Mild cognitive impairment (intelligence or developmental quotient 1-2 standard deviations below the mean)<br><br>3. Moderate-severe cognitive impairment (intelligence or developmental quotient more than 2 standard deviations below the mean)<br><br>4 Executive dysfunction, as defined by authors (direct testing)<br><br>5. Low numeracy, as defined by authors (by direct testing or educational achievement tests)<br><br>6. Low literacy, as defined by authors (by direct testing or educational achievement tests)<br><br>7. Special educational needs as defined by authors (school or parental report)<br><br>8. Motor impairment, as defined by authors (including direct testing, clinical record review, and reporting)<br><br>9. Visual-motor impairment, as defined by authors (on direct testing)	Studies of infants with mild HIE

Confidential: For Review Only

10. Emotional-behavioural difficulty, as defined by authors (including direct testing, clinical record review, and parental reporting<br><br>11. Speech and language impairment, as defined by authors (on direct testing)<br><br>12. Visual impairment, as defined by authors (including direct testing, clinical record review, and parental reporting)<br><br>13. Hearing impairment, as defined by authors (including direct testing, clinical record review, and parental reporting)<br><br>14. Epilepsy/seizures, as defined by authors (including medical history taking, clinical record review and parental reporting	
	Studies reporting outcomes for children diagnosed with brain injury beyond the neonatal period
	Studies where comparable outcome data from those with and without perinatal brain injury cannot be extracted

Confidential: For Review Only

Table 2: Overview of key findings for school-age outcomes of infants with perinatal brain injury compared to those without brain injury
(*Does not include studies where infants with IVH grade 3-4 cannot be separated from those with WMI or those with IVH 1-2)
(# Does not include studies using hearing or visual outcomes only as part of their composite outcome)
Adjusted Odds Ratio (aOR); Attention Deficit Hyperactivity Disorder (ADHD); Autism Spectrum Disorder (ASD); Confidence Interval (CI); cystic periventricular leukomalacia (cPVL); Group B Streptococcus (GBS); Hypoxic-Ischaemic Encephalopathy (HIE); Hazard Ratio (HR); Intelligence Quotient (IQ); Interquartile range (IQR); Intraventricular Haemorrhage (IVH); Odds Ratio (OR); Periventricular Leukomalacia (PVL); Visual Motor Integration (VMI); White Matter Injury (WMI)

	NDI	Cognitive	Motor	Speech and language	Behavioural	Hearing#	Vision#	Other
IVH grade 3-4*	6 studies(15, 17-21)<br><br>2 comparable studies in meta-analysis(17, 20)<br><br>**Meta-analysis** (2 studies): Increased risk of moderate - severe neurodevelopmental impairment OR 3.15 (95%CI: 1.67, 5.92) I^2 = 0%<br><br>**Van de Bor 2004**: increased prevalence of disability 31% vs. 16%	9 studies(15, 20, 21, 24-26, 30, 70)<br><br>Not comparable<br><br>Consistently highlighted lower cognitive scores<br><br>**Brouwer 2012**: significantly lower performance IQ but preserved verbal IQ. Lower IQ for those with IVH grade 4 requiring neurosurgery (91+/-10 vs. 98+/-15) but little difference for those with grade 3 IVH requiring neurosurgery (96+/-15 vs. 98+/-15).<br><br>**Hollebrandse 2021**: increased risk of cognitive impairment OR 2.68 (95%CI: 1.21, 5.94). Increased risk of academic impairment across all academic domains: reading OR 3.62 (95%CI: 1.59, 8.24); spelling OR 4.48 (95%CI: 1.8, 11.2); arithmetic OR 2.79)95%CI: 1.2, 6.48)<br><br>**Sherlock 2005:** significantly lower IQ scores after IVH grade 4 vs. IVH 1-3 and no brain injury, also seen for	6 studies(20, 23-26, 33)<br><br>Not comparable<br><br>All reported increased risk of motor impairment<br><br>**<u>Cerebral palsy</u>**<br>3 comparable studies<br><br>OR 8.67 (95%CI: 5.27, 14.28) I^2=0%.	3 studies(20, 21, 25)<br><br>Not comparable<br><br>**Van de Bor 2004**: no significant difference in language scores<br><br>**Sherlock 2005**: downward trend in language scores from no brain injury to each grade of IVH but not statistically significant p=0.12<br><br>**Hollebrandse 2021**: Increased risk of impaired reading OR 3.62 (95%CI: 1.59, 8.24), and spelling OR 4.48 (95%CI: 1.8, 11.2)	3 studies(15, 24, 35)<br><br>Not comparable<br><br>**Brouwer 2012**: no association with any behavioural domains assessed (internalising, externalising and sleep problems)<br><br>**Adant 2019**: no increased risk of attention deficits, conduct issues or ASD aOR 1.24 (95%CI: 0.32, 4.8).<br><br>**Davidovich 2020**: no increased risk of ASD (n=10, 3.9% vs. n=103, 2.2% p=0.085)	3 studies(21, 26, 38)<br><br>Not comparable<br><br>Outcome too rare for inferential analysis<br><br>**Kaur 2020:** increased risk of hospitalisation for otologic reasons HR 7.87 (95%CI: 5.31, 11.67)	5 studies(15, 21, 26, 33, 38)<br><br>Not comparable<br><br>Outcome to rare for inferential analysis in most studies.<br><br>**Adant 2019**: no increased risk of visual impairment (needing glasses) aOR 0.47 (95%CI: 0.13, 1.69)<br><br>**Klebermass-Schrehof 2012:** increased prevalence of visual impairment (needing glasses or blindness) after IVH grade 3 (45.4%) and IVH grade 4 (90.9%) vs. comparators (7.5%).<br><br>**Kaur 2020:** increased risk of hospitalisation for ophthalmic reasons HR 7.87 (95%CI: 5.31, 11.67).<br><br>**Klebermass-Schrehof 2012:** significantly lower	

Confidential: For Review Only

		several domains: freedom from distractibility, processing speed, reading, spelling and arithmetic. No difference in executive function.<br><br>**Van de Bor 2004**: increased special education needs at 5, 9 and 14 years aOR 3.99 (95%CI: 1.36, 11.69).					VMI scores (67.5 ± 14 vs. 76 ± 26.8; p=0.04)		
WMI*	3 studies(16, 17, 22)<br><br>Not comparable<br><br>**Campbell 2021**: living with no impairment was less common with WMI (n=12, 40%) vs. controls (n=487, 76%)<br><br>**Cheong 2018**: increased risk of survival with major disability after cPVL aOR 9·17 (95%CI: 3·57, 23·53)<br><br>**Vollmer 2003:** Disabling impairments were more common after cPVL at<28 weeks' gestation (n=3, 75% <28	4 studies(16, 29, 32, 70)<br><br>Not comparable<br><br>**Van den Hout 2000**: 50% with PVL had IQ scores <85 vs. 11.8% without injury and a lower performance age 4.3 years vs. 6.2 years<br><br>**Campbell 2021**: increased risk of moderate-severe cognitive impairment aOR 5.07 (95%CI: 2.13, 12.02)<br><br>**Jansen 2020**: WMI predictive of poorer performance on standardised mathematics tests (B 1.856 p=0.003), but not performance on spelling (B 1.076 p=0.075) or reading tests (B 0.241 p=0.483)	<u>**Cerebral palsy**</u><br>1 study(16)<br><br>**Campbell 2020**: increased risk of cerebral palsy aOR 18.63 (95%CI: 7.37, 47.06)	1 study(29)<br><br>**Jansen 2020**: No association between WMI and spelling (B 1.076 p=0.075) or reading performance (B 0.241 p=0.483)	4 studies(16, 35, 36, 71)<br>Not comparable<br><br>Conflicting results<br><br>**Campbell 2021**: No increased risk of: ADHD (n=3, 10% vs. n=97, 15%); anxiety (n=3, 10% vs. n=98, 15%); depression (n=7, 23% vs. n=100, 16%); or ASD aOR 0.74 (95%CI: 0.09, 5.88)<br><br>**Davidovich 2020**: No increased risk of ASD after PVL (n=5, 2.5% vs. n=88, 2.3% p=0.86)<br><br>**Whitaker 2011:** increased risk of ADHD aOR 6.83 (95%CI: 1.26-36.91); major depression aOR 2.59 (95%CI: 1.02-6.58); tic disorders aOR 9.77 (95%CI: 1.69-56.47); obsessive compulsive disorders aOR 15.32 (95%CI: 1.82-128.74)	0 studies	1 study(32)	

Confidential: For Review Only

	weeks) vs. controls (n=3, 8%) and at over 28 weeks' gestation (n=6,50% vs. n=14, 6%)							
Stroke	0 studies	6 studies(39, 41, 42, 44-46)<br>5 comparable studies in meta-analysis (39, 41, 44-46)<br><br>**Meta-analysis** (5 studies): significant mean difference in full scale IQ: -24.2 (95%CI: -30.73, -17.67) I^2=80%<br><br>**Trauner 2001 and Gold 2014**: no significant difference in full scale IQ scores in left vs. right-sided strokes<br><br>**Ballantyne 2008:** significantly lower performance IQ (p=0.002) and verbal IQ (p<0.0001). Lower mean scores for reading (p<0.0001), spelling (p=0.001) and arithmetic (p<0.0001) at 7-8 years persisting to 10-12 years<br><br>**Tillema 2008:** reduced verbal IQ scores (mean 84 SD 13.4) vs. (mean 108 SD 14.2 P=0.002)<br><br>**Kolk 2011**: poorer attention (across 4 of the 7 assessment sub-domains), visuo-spacial function (across 4 of the 5 sub-domains), and memory and learning (across 4 of the 6 sub-domains), but normal executive function scores.	5 studies(39, 41-44)<br>Combined hemiparesis incidence: 61% (95%CI: 39.2, 82.9 I^2=88%)<br><br>**Kolk 2011:** moderate to severe neuromotor impairment in 62% n=13) and significantly lower scores on 5/6 sensorimotor domains of the NEPSY	5 studies(39, 40, 42, 44, 45)<br><br>3 comparable studies in meta-analysis<br>**Meta-analysis** (3 studies): lower receptive language scores-20.88 (95%CI: -36.66, -5.11) I^2=88% and lower expressive language scores -20.25 (95%CI: -34.36, -6.13) I^2=87%<br><br>**Ballantyne 2007 and Ballantyne 2008**: deficits in receptive language scores at 7-8 years persist at 10-12 years but expressive language scores improved (p=0.012) particularly for children with right-sided strokes (p=0.034)<br><br>**Kolk 2011:** significantly lower scores for 8/9 NEPSY domains including phonologic processing, comprehension of instructions, correct speeded naming, repetition of nonsense words, verbal fluency (semantic and phonetic), oromotor sequences, and sentence comprehension	1 study(46)	1 study(43)<br><br>**Martin 2019:** left-sided strokes predispose children to contralateral auditory neglect and right-sided strokes predispose children to bilateral auditory neglect	1 study(39)<br><br>**Ballantyne 2008:** visual field defects are common (n=7, 26%) after perinatal stroke	<u>**Seizures**</u><br>8 studies(39, 42, 43, 45, 46)<br><br>5 comparable studies(39, 42, 43, 45, 46)<br>Combined incidence of seizures: 40.1% (95%CI: 26.8, 53.3) I^2=56%

Confidential: For Review Only

		Those with left-sided strokes had poorer neuropsychological scores.<br><br>**Northam 2018:** most children are in mainstream education (n=28, 93%) but many require additional support (n=12, 40%)						
Meningitis	3 studies(47-49)<br>Not comparable<br><br>All reported increased risk of neurodevelopmental impairment<br><br>**Bedford 2011:** increased prevalence of neuromotor disability (n=45, 16% vs. n=2, 0.1%)<br><br>**Stevens 2003:** Risk of severe disability seen in Bedford 2011 at 5 years of age persisted until 9-10 years (n=12, 10.8% vs. n=0, 0%)<br><br>**Horvath-Puho 2021:** increased risk of any neurodevelopmental impairment	1 study(49)<br><br>**Stevens 2003:** significantly lower mean cognitive scores (mean 88.8 (95%CI: 85, 92) vs. mean 99.4 (95%CI: 97, 102))	1 study(49)<br><br>**Stevens 2003:** significantly higher motor impairment scores (mean 7.1 (95%CI: 5.9, 8.5) vs. mean 5 (95%CI: 4.3, 5.8))	0 studies	0 studies	2 studies(49, 72)<br><br>**Martinez Cruz 2008:** increased odds of neonatal meningitis amongst preterm infants with sensorineural hearing loss OR 4.37 (95%CI: 1.7, 10.9<br><br>**Stevens 2003:** 3.6% (n=4) had hearing loss compared to none in the control group.	1 study(49)<br><br>**Stevens 2003:** Bilateral visual impairment was common after neonatal meningitis (n=18, 17%)	

Confidential: For Review Only

	after GBS meningitis in the Netherlands RR 5.30 (95%CI: 2·57, 10·89) and Denmark RR 7.80 (95%CI: 4·42, 13·77) at 5 years of age persisting to 11 years in the Netherlands RR 2.99 (95%CI: 1.83, 4.88) and 15 years in Denmark RR 3.15 (95%CI: 1.82, 5,46)							
HIE	0 studies	3 studies(30, 50, 51) (two of the same population)<br><br>Not comparable<br><br>**Koc 2016:** preterm infants with HIE significantly more likely to have below average IQ scores (n=8, 89% vs. n=24, 30% p=0.001)<br><br>**Lee-Kelland 2020 and Tonks 2019:** report lower full scale IQ scores after moderate to severe HIE (mean difference −13.62 (95%CI: −20.53, −6.71)) and poorer perceptual reasoning, working memory and processing speed. Children with previous HIE more likely to receive additional classroom support OR 10 (95%CI: 1.16, 86)	2 studies(50, 51) (of the same population)<br><br>**Lee-Kelland 2020 and Tonks 2019:** significantly lower motor scores (mean difference −2.12 (95%CI: −3.93, −0.30)) after moderate-severe HIE (for children without cerebral palsy)	2 studies(50, 51) (of the same population)<br><br>**Lee-Kelland 2020 and Tonks 2019:** significantly lower verbal comprehension scores (mean difference −8.8 (95%CI: −14.25, −3.34)) after moderate-severe HIE.	2 studies(50, 51) (of the same population)<br><br>**Lee-Kelland 2020 and Tonks 2019:** higher behavioural difficulty scores (median score 12 IQR (6.5, 13.5 vs. median score 6 IQR (2.25, 10) p=0.005)	0 studies	0 studies	
Kernicterus	0 studies							

Confidential: For Review Only

Confidential: For Review Only

PRISMA 2009 Flow Diagram

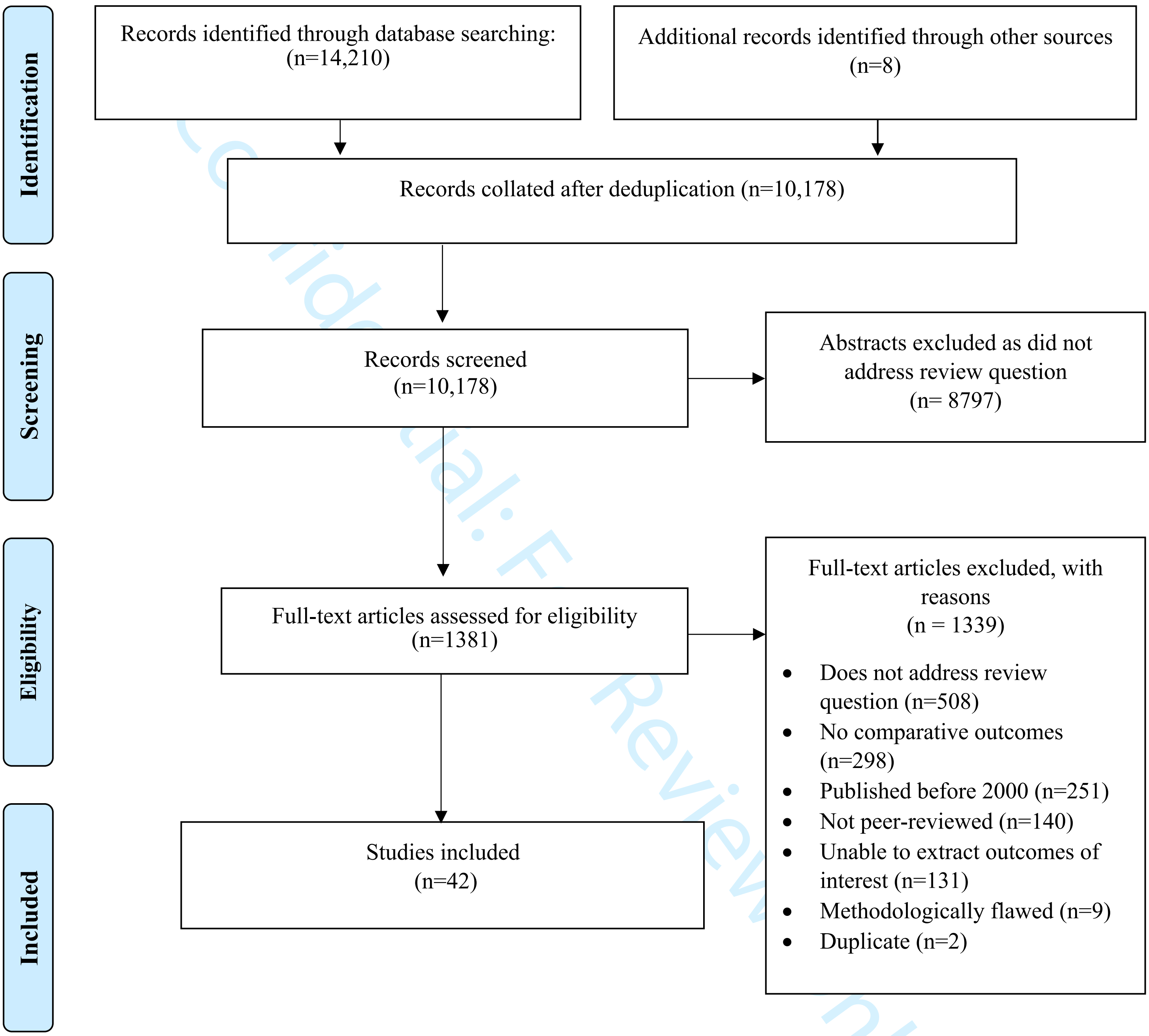

Confidential: For Review Only

Confidential: For Review Only

Confidential: For Review Only

Study or Subgroup	log[Odds Ratio]	SE	Weight	Odds Ratio IV, Random, 95% CI
Cheong 2018	1.38879124	0.59424024	43.9%	4.01 [1.25, 12.85]
Sherlock 2005	1.23969089	0.52602402	56.1%	3.45 [1.23, 9.69]
**Total (95% CI)**			**100.0%**	**3.69 [1.70, 7.98]**

Heterogeneity: Tau² = 0.00; Chi² = 0.04, df = 1 (P = 0.85); I² = 0%
Test for overall effect: Z = 3.31 (P = 0.0009)

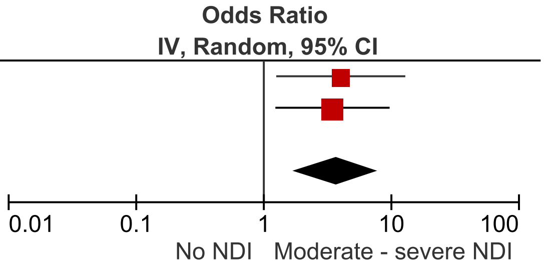

Study or Subgroup	IVH grade 3-4 Events	IVH grade 3-4 Total	No IVH Events	No IVH Total	Weight	Odds Ratio M-H, Random, 95% CI
Beaino 2010	9	38	46	1153	48.5%	7.47 [3.34, 16.69]
Hollebrandse 2021	15	35	26	331	51.5%	8.80 [4.03, 19.19]
**Total (95% CI)**		**73**		**1484**	**100.0%**	**8.13 [4.64, 14.22]**
Total events	24		72			

Heterogeneity: Tau² = 0.00; Chi² = 0.08, df = 1 (P = 0.77); I² = 0%
Test for overall effect: Z = 7.33 (P < 0.00001)

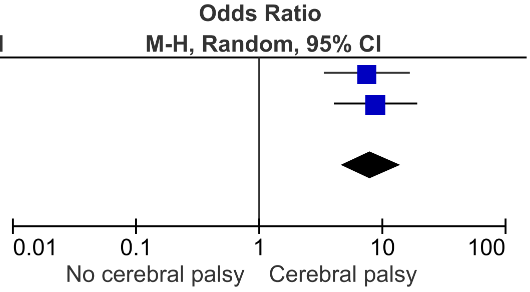

Confidential: For Review Only

	Perinatal stroke			No stroke				Mean Difference
Study or Subgroup	Mean	SD	Total	Mean	SD	Total	Weight	IV, Random, 95% CI
Ballayntyne 2008	94.7	20.4	29	123	15	38	18.2%	-28.30 [-37.12, -19.48]
Gold 2014	88	4	27	117	2.7	19	26.6%	-29.00 [-30.94, -27.06]
Northam 2017	99	14	30	112	16	40	20.7%	-13.00 [-20.05, -5.95]
Tilema 2008	80	14.1	10	108	11.7	10	14.9%	-28.00 [-39.36, -16.64]
Trauner 2001	93.4	22	39	116.2	13	54	19.7%	-22.80 [-30.53, -15.07]
**Total (95% CI)**			**135**			**161**	**100.0%**	**-24.20 [-30.73, -17.67]**

Heterogeneity: Tau² = 40.85; Chi² = 20.00, df = 4 (P = 0.0005); I² = 80%
Test for overall effect: Z = 7.26 (P < 0.00001)

Mean Difference
IV, Random, 95% CI

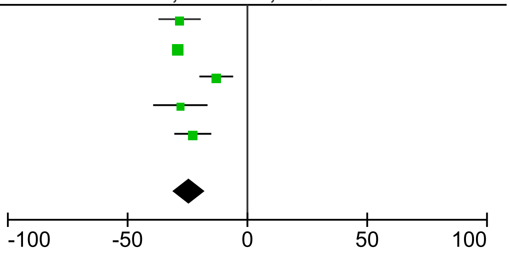

Confidential: For Review Only

**Supplement 1: databases searched**
Cochrane Central Register of Controlled Trials
EBSCO–CINAHL (Cumulative Index to Nursing and Allied Health Literature)
Google Scholar
Ovid–EMBASE
Ovid–MEDLINE
Ovid–MEDLINE E-pub ahead of print
Ovid–MEDLINE In-Process and Other Non-Indexed Citations
PubMed
Scopus
Web of Knowledge (Science Citation Index Expanded and Conference Proceedings Citation Index Science)

Confidential: For Review Only

Supplement 2: Medline Ovid Search Strategy

1. exp CHILD/
2. exp Child, Preschool/
3. exp ADOLESCENT/
4. exp INFANT/ or exp INFANT, NEWBORN/
5. (child* or toddler* or baby or infant* or adolescent*).mp.
6. 1 or 2 or 3 or 4 or 5
7. exp Educational Status/
8. exp Child Development/
9. exp Learning Disorders/
10. exp Educational Measurement/
11. exp SCHOOLS/
12. exp Academic Performance/
13. school performance.mp.
14. exp COGNITION/
15. exp LEARNING/
16. exp SPATIAL LEARNING/
17. exp VERBAL LEARNING/
18. exp SOCIAL LEARNING/
19. exp Intelligence Tests/
20. exp INTELLIGENCE/
21. exp Intellectual Disability/
22. exp Neurodevelopmental Disorders/
23. neurodevelopm*.mp.
24. (nervous system dys* or CNS dys*).mp. [mp=title, abstract, heading word, drug trade name, original title, device manufacturer, drug manufacturer, device trade name, keyword, floating subheading word, candidate term word]
25. (nervous system abnorm* or CNS abnorm*).mp. [mp=title, abstract, heading word, drug trade name, original title, device manufacturer, drug manufacturer, device trade name, keyword, floating subheading word, candidate term word]
26. (nervous system malform* or CNS malform*).mp. [mp=title, abstract, heading word, drug trade name, original title, device manufacturer, drug manufacturer, device trade name, keyword, floating subheading word, candidate term word]
27. (nervous system dis* or CNS dis*).mp. [mp=title, abstract, heading word, drug trade name, original title, device manufacturer, drug manufacturer, device trade name, keyword, floating subheading word, candidate term word]
28. (mental health condi* or mental health dis*).mp. [mp=title, abstract, heading word, drug trade name, original title, device manufacturer, drug manufacturer, device trade name, keyword, floating subheading word, candidate term word]
29. mental health outcome.mp.
30. behaviour* abnorm*.mp.
31. cognitive impairment.mp. or exp Cognitive Dysfunction/
32. visual impairment.mp. or exp Vision Disorders/
33. visual develop*.mp.
34. (visual dis* or visual dys*).mp. [mp=title, abstract, heading word, drug trade name, original title, device manufacturer, drug manufacturer, device trade name, keyword, floating subheading word, candidate term word]

Confidential: For Review Only

35. (nystagmus or strabismus).mp.
36. (visual acuity or refractive error*).mp.
37. hearing impairment.mp. or exp Hearing Loss/
38. exp Deafness/
39. exp DEAF-BLIND DISORDERS/
40. exp Hearing Loss, Sensorineural/
41. exp Movement Disorders/
42. exp Cerebral Palsy/
43. motor impairment.mp.
44. (seizure* or convulsi*).mp.
45. exp EPILEPSY/ or epilepsy.mp.
46. exp Executive Function/
47. visual-motor impairment.mp.
48. numeracy.mp.
49. literacy.mp. or exp LITERACY/
50. jaundice.mp.
51. exp Language Development Disorders/ or exp Child Language/ or language impairment.mp. or exp Reading/ or exp Dyslexia/ or reading impairment.mp.
52. 7 or 8 or 9 or 10 or 11 or 12 or 13 or 14 or 15 or 16 or 17 or 18 or 19 or 20 or 21 or 22 or 23 or 24 or 25 or 26 or 27 or 28 or 29 or 30 or 31 or 32 or 33 or 34 or 35 or 36 or 37 or 38 or 39 or 40 or 41 or 42 or 43 or 44 or 45 or 46 or 47 or 48
53. 49 or 50 or 51
54. 52 or 53
55. exp JAUNDICE, NEONATAL/
56. exp JAUNDICE/
57. exp Hyperbilirubinemia, Neonatal/
58. exp Hyperbilirubinemia/
59. hyperbilirubin*.mp.
60. exp Hyperbilirubinemia, Hereditary/
61. bilirubin encephalopathy.mp.
62. bilirubin-induced neuro*.mp.
63. exchange transfusion.mp.
64. exp ASPHYXIA NEONATORUM/
65. (exp ASPHYXIA/ or asphyxia.mp.) and neonat*.mp.
66. exp Hypoxia-Ischemia, Brain/ and neonat*.mp.
67. perinatal asphyxia.mp.
68. birth asphyxia.mp.
69. (hypoxic-ischemic encephalopathy or hypoxic-ischaemic encephalopathy).mp.
70. neonatal encephalopathy.mp.
71. (exp Cerebral Hemorrhage/ or exp Intracranial Hemorrhages/ or exp Brain Ischemia/ or intracranial haemorrhage.mp. or exp Subarachnoid Hemorrhage/ or exp Stroke/) and neonat*.mp.
72. perinatal stroke.mp.
73. (central nervous system infection.mp. or exp Central Nervous System Infections/) and neonat*.mp.
74. (exp Meningoencephalitis/ or meningo-encephalitis.mp.) and neonat*.mp.
75. (MENINGITIS/ or meningitis.mp.) and neonat*.mp.

Confidential: For Review Only

76. exp MENINGITIS, VIRAL/ and neonat*.mp.
77. (meningoencephalitis and neonat*).mp.
78. (encephalitis.mp. or exp ENCEPHALITIS, VIRAL/ or exp INFECTIOUS ENCEPHALITIS/ or exp ENCEPHALITIS/) and neonat*.mp.
79. kernicterus.mp. or exp KERNICTERUS/
80. preterm white matter disease.mp.
81. (periventricular leukomalacia.mp. or exp Leukomalacia, Periventricular/) and neonat*.mp.
82. (therapeutic hypothermia.mp. or exp Hypothermia, Induced/) and neonat*.mp.
83. ((subdural haemorrhage or subdural hemorrhage) and neonat*).mp.
84. (exp Hematoma, Subdural/ or subdural haemorrhage.mp. or exp Craniocerebral Trauma/) and neonat*.mp.
85. (intraventricular haemorrhage and neonat*).mp.
86. (tentorial tear and neonat*).mp.
87. (parenchymal haemorrhage and neonat*).mp.
88. (ventriculoperitoneal shunt.mp. or exp Cerebrospinal Fluid Shunts/ or exp Ventriculoperitoneal Shunt/) and neonat*.mp.
89. ((ventricular drain or Rickham reservoir or CSF shunt) and neonat*).mp.
90. neonatal stroke.mp.
91. (cerebrovascular accident and neonat*).mp.
92. neonatal cerebral ischaemia.mp.
93. (exp Intracranial Thrombosis/ or cerebral venous thrombosis.mp.) and neonat*.mp.
94. (seizure.mp. or exp Seizures/) and neonat*.mp.
95. 55 or 56 or 57 or 58 or 59 or 60 or 61 or 62 or 63 or 64 or 65 or 66 or 67 or 68 or 69 or 70 or 71 or 72 or 73 or 74 or 75 or 76 or 77 or 78 or 79 or 80 or 81 or 82 or 83 or 84 or 85 or 86 or 87 or 88 or 89 or 90 or 91 or 92 or 93 or 94
96. exp Cohort Studies/
97. exp Retrospective Studies/
98. (cohort* or (case$ and control$)).tw.
99. exp Cross-Sectional Studies/
100. exp Randomized Controlled Trial/
101. 96 or 97 or 98 or 99 or 100
102. exp "REVIEW"/
103. exp Case Reports/
104. Animals/
105. animal stud*.mp.
106. 102 or 103 or 104 or 105
107. 6 and 52 and 95 and 101
108. 107 not 106

Confidential: For Review Only

Supplement 3: included studies of school-aged outcomes after perinatal brain injury

* overlapping study data; Ω potential error in manuscript; Adjusted Odds Ratio (aOR); Autism spectrum Disorder (ASD); Attention Deficit Hyperactivity Disorder (ADHD); Bayley Scale of Infant Development (BSID); Child Behaviour Checklist (CBCL); Clinical Evaluation of Language Fundamentals (CELF); Cystic Periventricular leukomalacia (cPVL); Gross Motor Function Classification System, (GMFCS); Haemorrhagic parenchymal infarction (HPI); Hazard Ratio (HR); International Classification of Disease (ICD); Intraventricular haemorrhage (IVH); Intelligence Quotient (IQ); Kaufman Assessment Battery for Children (K-ABC); Mental Developmental Index (MDI); Peabody Picture Vocabulary Test (PPVT); Periventricular (PV); Periventricular leukomalacia (PVL); National Institute of Child Health and Human Development (NICHD); Neonatal Intensive Care Unit (NICU); Psychomotor Development Index (PDI); Retinopathy of Prematurity (ROP); Small for Gestational Age (SGA); Spontaneous Intestinal Perforation (SIP); Standard Deviation (SD); Standard Error (SE); Test of Motor Impairment (TOMI); Very low birthweight (VLBW); Visuomotor integration (VMI); Wechsler Abbreviated Scale of Intelligence (WASI); Wechsler Intelligence Scale for Children (WISC); Wechsler Preschool & Primary Scale of Intelligence (WPPSI); White Matter Injury (WMI); Wide Range Achievement Test (WRAT)

	Author<br>Year<br>Country<br>Study type	Population<br>Exposures<br>Comparator<br>Ascertainment/ definition	Outcomes	Main result(s)
1	Adant 2019[9]<br><br>Belgium<br><br>Retrospective cohort	**Population**<br>• Gestation ≤32 weeks with and without spontaneous intestinal perforation (SIP)<br>• Born 1994-2014<br><br>**Exposure (n=19)**<br>• IVH grade 3-4<br><br>**Comparator (n=44)**<br>• Matched on gender, gestational age, date of birth (multiples matched to sibling without SIP)<br>• No IVH<br><br>**Ascertainment/ definition**<br>• Clinical record review	**Outcomes**<br>• Functional disability (composite)<br>• Cognitive<br>• Motor<br>• Visual<br>• Behavioural/ mental health<br>• Wellbeing<br>• Quality of life<br>• Physical health<br><br>**Measurement/ assessment**<br>• BSID II<br>• Telephone survey (parents)<br>• PedsQL<br>• IQ testing<br><br>**Follow-up**<br>• 67% follow-up at 7-11 months<br>• 41% follow-up at 18-22 months<br>• 49% follow-up at 4-10 years<br>• 86% follow-up telephone survey	**Outcomes of those with SIP compared to controls without SIP – by IVH subgroup**<br><br>**Disability**<br>aOR 8.79 95%CI (1.72, 44.86)<br><br>**Multiple disabilities**<br>aOR 5.97 95%CI (1.61, 22.15)<br><br>**Cognitive**<br>**Regular education system (not a special educational needs school)**<br>aOR 8.73 95%CI (2.1, 36.72*)*<br><br>**Visual outcomes** (wearing glasses)<br>aOR 0.474 95%CI (0.13, 1.69)<br><br>**Behavioural/ mental health disorder** (including attention problems, conduct problems and autism spectrum disorders)<br>aOR 1.24 95%CI (0.32, 4.8)<br><br>**PedsQL low quality of life score**<br>aOR 0.87 95%CI (0.77, 0.99)<br><br>**PedsQL low physical health score**<br>aOR 0.82 95%CI (0.66, 1.01)
2*	Beaino 2010[68]<br><br>France<br><br>Prospective cohort	**Population**<br>• Gestation <33 weeks<br>• Born 1997<br><br>**Exposure**<br>• IVH grade 1 (n=173)<br>• IVH grade 2 (n=117)<br>• IVH grade 3 (n=32)<br>• Intraparenchymal haemorrhage (IPH) (n=6)<br>• Persistent echodensities or ventricular dilatation (n=241)<br>• cPVL (n=66)<br><br>**Comparator (n=1153)**<br>• Unmatched<br>• No IVH<br><br>**Ascertainment/ definition**<br>• Ultrasound imaging undertaken and reviewed by neonatologists or radiographers	**Outcomes**<br>• Cerebral palsy<br><br>**Measurement/assessment**<br>• Standardised questionnaires completed by physicians<br><br>**Follow-up**<br>• 5 years<br>• 77% follow-up	**Cerebral palsy**<br>**Grade 3 IVH**<br>OR 3.75 95%CI (2.41–5.85)<br><br>**Grade 3 IVH or echodensities of ventricular dilatation**<br>Model A aOR 3.25 95%CI (2.02–5.22)<br>Model B aOR 3.40 95%CI (2.07–5.60)<br>Model C aOR 3.31 95%CI (2.00–5.48)<br><br>**cPVL**<br>OR 33.41 95%CI (19.25–57.96)<br><br>**Cystic PVL or IPH**<br>Model A aOR 29.66 95%CI (16.71–52.62)<br>Model B aOR 28.41 95%CI (15.65–51.59)<br>Model C n/a
3	Brouwer 2012[18]<br><br>Netherlands<br><br>Prospective cohort	**Population**<br>• Gestation <32 weeks<br>• Born 1999-2004<br><br>**Exposure (n=32)**<br>• Post-haemorrhagic ventricular dilatation after IVH grade 3-4 requiring neurosurgical intervention<br>• No PVL<br><br>**Comparator (n=23)**<br>• Matched on gestation, birthweight, and sex<br>• No IVH<br><br>**Ascertainment/ definition**<br>• Ultrasound diagnosis<br>• Papile classification	**Outcomes**<br>• Motor<br>• Cerebral palsy<br>• Cognitive<br>• Behavioural<br><br>**Measurement/ assessment**<br>• Movement ABC<br>• GMFCS<br>• WPPSI (3rd edition Dutch version)<br>• Revisie Amsterdamse Kinder Intelligentietest<br>• Snijders Oomen Nonverbal Intelligence Test 2.5-7 – Revised<br>• CBCL<br>• Teacher Report Form<br><br>**Follow-up**<br>• 4-8 years (median 5.7)<br>• 97% follow-up	**Cerebral palsy**<br>IVH grade 3 n=0<br>IVH grade 4 n=8, 53%; all unilateral spastic cerebral palsy<br>GMFCS level 1, n=5<br>GMFCS level 2, n=2<br>GMFCS level 3, n=1<br><br>**Movement ABC motor score (for those without cerebral palsy)**<br>**Score <p 5 (definite motor problems)**<br>IVH grade 3 n=6, 26%<br>IVH grade 4 n=3, 13%<br>No IVH n=0<br><br>**Score p 5-15 (borderline motor function)**<br>IVH grade 3 (n=6; 26%)<br>IVH grade 4 (n=0; 0%)<br>No IVH (n=5; 29.4%)<br><br>**Score p> 15**<br>IVH grade 3 n=6, 26%<br>IVH grade 4 n=0, 0%<br>No IVH n=12, 70.6%<br><br>**Cognition**<br>**Wechsler intelligence test (mean ±SD)**<br>**Verbal scale**<br>IVH n=23, 97±13<br>IVH <30weeks' gestation n=16, 94±13<br>No IVH n=24, 96±13;<br><br>**Performance scale**<br>IVH, n=23, 94±16;<br>IVH <30weeks' gestation n=16, 93±15<br>No IVH n=24, 103±14;<br><br>**Production scale**

Confidential: For Review Only

				IVH n=23, 87±22;<br>IVH <30weeks' gestation n=16, 85±24<br>No IVH n=24, 93±14<br><br>**Intelligence quotient (n; mean +/-SD)**<br>IVH grade 3 n=17; IQ 96±15;<br>IQ>85 n=13 (76.5%)<br><br>IVH IV n=15; IQ 91±10;<br>IQ >85 n=9 (64.3%)<br><br>IVH <30 weeks' gestation n=23; IQ 92±17;<br>IQ>85 n=15 (65.2%)<br><br>No IVH n=23; IQ 98±15,<br>IQ>85 n=17 (74%)<br><br>**Behavioural outcomes**<br>**CBCL parental score: mean T score ±SD, n in subclinical range (%)**<br>**Total scale**<br>IVH n=26: 48.2 ±8.4, n=3 (12%)<br>IVH <30 weeks' gestation n=20: 46.9 ±8.3, n=2 (10%)<br>No IVH <30 weeks' gestation n=23: 44.3 ±7.8, n=1 (4%)<br><br>**Internalising problem scale**<br>IVH: 49.2 ±8.9, n=5 (19%)<br>IVH <30 weeks' gestation: 28.2 ±8.4, n=3 (15%)<br>No IVH <30 weeks' gestation: 49.2 ±9.1, n=5 (21%)<br><br>**Externalizing problem scale**<br>IVH: 46.8 ±9.4, n=2 (8%)<br>IVH <30 weeks' gestation: 45.1 ±9.5, n=1 (15%)<br>No IVH < 30weeks' gestation: 43.7 ±7.5, n=0 (0%)<br><br>**TRF teachers score: mean T score ±SD, n in subclinical range (%)**<br>**Total scale**<br>IVH n=25: 54.7 ±8.7, n=6 (24%)<br>IVH <30 weeks' gestation n=19: 53.9 ±9.0, n=4 (21%)<br>No IVH <30 weeks' gestation n=22: 50.9 ±9.8, n=4 (18%)<br><br>**Internalising problem scale**<br>IVH: 53.2 ±10.8, 4 (16%)<br>IVH <30 weeks' gestation: 52.2 ±11.7, n=3 (16%)<br>No IVH <30 weeks' gestation: 52.4 ±11.4, n=7 (32%)<br><br>**Externalizing problem scale**<br>IVH: 54.3 ±6.7, 3 (12%)<br>IVH <30 weeks' gestation: 54.1 ±7.0, n=2 (11%)<br>No IVH <30 weeks' gestation: 49.7 ±7.7, n=2 (9%)<br><br>N=13 (41%) had repeated a school class, had educational help and/or attended special education
4	Campbell 2021[10]<br><br>USA<br><br>Prospective cohort study	**Population (n=858)**<br>• Gestation 23-27 weeks<br>• Born 2002-2004<br><br>**Exposure**<br>• IVH without WMI (n=124)<br>• WMI without IVH (n=30)<br>• IVH and WMI (n=63)<br><br>**Comparator (n=641)**<br>• Unmatched<br>• No IVH or WMI<br><br>**Ascertainment/ definition**<br>• Ultrasound imaging reviewed by two independent blinded radiologists<br>• WMI: parenchymal echolucency or moderate to severe ventriculomegaly on a late scan	**Outcomes**<br>• Neurocognitive development (composite)<br>• Cognitive<br>• Cerebral palsy<br>• Behavioural/ mental health<br>• Epilepsy<br>• Quality of life<br><br>**Measurement/ assessment**<br>• Differential Ability Scale II<br>• NEPSY II<br>• Neurological exam<br>• GMFCS<br>• Parental questionnaire<br>• Social Communication Questionnaire<br>• Child Symptom Inventory 4<br>• Peds QoL 4<br><br>**Follow up**<br>• 10 years<br>• 74% follow-up	**Neurodevelopmental burden**<br>**No impairments**<br>IVH and WMI n=24, 38%<br>WMI n=12, 40%<br>IVH n= 86, 69%<br>No IVH or WMI n=487, 76%<br><br>**No cognitive impairment; 1 or more of cerebral palsy, ASD, or epilepsy**<br>IVH and WMI n=4, 6%<br>WMI n=4, 13%<br>IVH n=7, 6%<br>No IVH or WMI n=26, 4%<br><br>**Cognitive**<br>**Normal cognitive function**<br>IVH and WMI n=8, 13%<br>WMI n=5, 17%<br>IVH n=41, 33%<br>No IVH or WMI n=235, 37%<br><br>**Cognitive impairment (moderate to severe)**<br>**IVH and WMI**<br>n=35, 56%<br>OR 5.01 95% CI (2.94, 8.54)<br>aOR 4.49 95% CI (2.49, 8.11)<br><br>**WMI**<br>n=14, 47%<br>OR 3.51 95% CI (1.67, 7.37)<br>aOR 5.07 95% CI (2.13, 12.02)<br><br>**IVH**<br>n=31, 25%<br>OR 1.34 95% CI (0.85, 2.1)<br>aOR 1.21 95% CI (0.73, 1.98)<br><br>**No IVH or WMI**<br>n=128. 20%<br>Reference category<br><br>**Low cognitive function**<br>IVH and WMI n=18. 30%<br>WMI n=10, 34%<br>IVH n=50, 41%<br>No IVH or WMI n=269, 43%<br><br>**Moderate cognitive impairment**<br>IVH and WMI n=17, 28%

Confidential: For Review Only

				WMI n=7, 24%<br>IVH n=24, 20%<br>No IVH or WMI n=93, 15%<br><br>**Severe cognitive impairment**<br>IVH and WMI n=18, 30%<br>WMI n=7, 24%<br>IVH n=7, 6%<br>No IVH or WMI n=35, 6%<br><br>**Nonverbal IQ**<br>**IVH vs. No IVH or WMI**<br>Crude mean difference -3 95%CI (-6.6, 0.6)<br><br>**Full scale IQ**<br>**IVH vs No IVH or WMI**<br>Crude mean difference -2.2 95%CI (-5.7, 1.4)<br><br>**Cerebral palsy**<br>**IVH and WMI**<br>n=32, 51%<br>OR 16.85 95% CI (9.29, 30.55)<br>aOR 13.43 95% CI (7, 25.78)<br><br>**WMI**<br>n=14, 47%<br>OR 14.28 95% CI (6.48, 41.48)<br>aOR 18.63 95% CI (7.37, 47.06)<br><br>**IVH**<br>n=9, 7%<br>OR 1.28 95% CI (0.6, 2.72)<br>aOR 1.19 95% CI (0.54, 2.61)<br><br>**No IVH or WMI**<br>n=37, 6%<br>Reference category<br><br>**GMFCS>0**<br>IVH and WMI n=16, 25%<br>WMI n=10, 33%<br>IVH n=4, 3%<br>No IVH or WMI n=13, 2%<br><br>**Epilepsy**<br>**IVH and WMI**<br>n=12, 19%<br>OR 5.44 95 % CI (2.72, 10.86)<br>aOR 4.89 95% CI (2.31, 10.35)<br><br>**WMI**<br>n=8, 27%;<br>OR 6.92 95% CI (2.86, 16.75)<br>aOR 7.56 95% CI (2.85, 20.06)<br><br>**IVH**<br>n= 11, 9%;<br>OR 1.85 95% CI (0.91, 3.78)<br>aOR 1.5 95% CI (0.68, 3.3)<br><br>**No IVH or WMI**<br>n=25, 4%<br>Reference category<br><br>**Neuropsychiatric/ behavioural outcomes**<br>**ASD**<br>**IVH and WMI**<br>n=4, 6%<br>OR 0.97 95% CI (0.34, 2.79)<br>aOR 0.58 95% CI (0.19, 1.77)<br><br>**WMI**<br>n=2, 7%<br>OR 1.02 95% CI (0.23, 4.42)<br>aOR 0.74 95% CI (0.09, 5.88)<br><br>**IVH**<br>n=11, 9%<br>OR 1.39 95% CI (0.69, 2.78)<br>aOR 1.24 95% CI (0.59, 2.6)<br><br>**No IVH or WMI**<br>n=42, 7%<br>Reference category<br><br>**Social responsiveness scale** (over 65 among children with IQ >85 excluding those with ASD)<br>IVH and WMI n=5, 8%<br>WMI n=4, 13%<br>IVH n=14, 11%<br>No IVH or WMI n=62, 10%<br><br>**ADHD**<br>IVH and WMI n=13, 24%<br>WMI n=3, 10%<br><br>IVH n=31, 25%<br>OR 1.6 95% CI (1.1, 2.5)<br><br>No IVH or WMI n=97, 15%

Confidential: For Review Only

				**Anxiety (parent-reported)**<br>IVH and WMI n=6, 10%<br>WMI n=3, 10%<br>IVH n=10, 8%<br>No IVH or WMI n=98, 15%<br><br>**Anxiety (teacher-reported)**<br>IVH and WMI n=12, 19%<br>WMI n=3, 10%<br>IVH n=14, 11%<br>No IVH or WMI n=88, 14%<br><br>**Depression (parent-reported)**<br>IVH and WMI n=7, 11%<br>WMI n=7, 23%<br>IVH n=14, 11%<br>No IVH or WMI n=100, 16%<br><br>**Depression (teacher-reported)**<br>IVH and WMI n=20, 32%<br>WMI n=7 23%<br>IVH n=18, 15%<br>No IVH or WMI n=96, 15%<br><br>**Poor quality of life (<70)**<br>IVH and WMI n=31, 49%<br>WMI n=12, 40%<br>IVH n=41, 25%<br>No IVH or WMI n=131, 20%
5	Cheong 2018[11]<br><br>Australia<br><br>Three prospective cohort studies	**Population**<br>• Gestation 22-27 weeks<br>• Born 1991-1992; 1997-1998; 2005-2006<br><br>**Exposure**<br>• IVH grade 3-4 (n=100)<br>• cPVL (n=38)<br><br>**Comparator**<br>• Unmatched<br>• No IVH grade 3-4 (n=446)<br>• No cPVL (n=508)<br><br>**Ascertainment/ definition**<br>• Not specified	**Outcomes**<br>• Survival with major disability (composite)<br>• Survival without major disability (composite)<br>• Cognitive<br>• Cerebral palsy<br>• Visual impairment (acuity less than 6/60 in better eye)<br>• Hearing impairment (requiring hearing aid or cochlear amplification)<br>**Assessment/ measurement**<br>• GMFCS<br>• WISC III<br>• WISC IV<br>• Differential Abilities Scales 2nd edition<br>**Follow-up**<br>• 8 years<br>• 91% follow-up of survivors	**Survival with major disability**<br>**IVH grade 3-4**<br>OR 2·98 95% CI (1·34, 6·63) p=0.01<br>aOR 2·61 95%CI (1·11–6·15) p=0·028<br><br>1997 and 2005 cohort only:<br>OR 4·01 95% CI (1·25, 12·84) p=0.02<br><br>**cPVL**<br>OR 8·11 95% CI (3·24, 20·30) p<0.001<br>aOR 9·17 95% CI (3·57–23·53) p<0·0001<br><br>1997 and 2005 cohort only<br>OR 17·0 95% CI (4·19, 69·02) p<0·001
6	Chou 2020[69]<br><br>Taiwan<br><br>Retrospective cohort study	**Population**<br>• Preterms infants <37 weeks' gestation (n=21,474)<br>• Infants born small for gestational age (n=2206)<br>• Born 2000-2010<br><br>**Exposure**<br>• Preterm with cerebral haemorrhage<br>• SGA with cerebral haemorrhage<br><br>**Comparator (n=94,720)**<br>• Matched 1:4 on gender, urbanisation of residential area and parental occupation<br>• No cerebral haemorrhage<br><br>**Ascertainment/ definition**<br>• National children's medical record database<br>• ICD 9 codes	**Outcome**<br>• Epilepsy<br><br>**Assessment/ measurement**<br>• ICD 9<br><br>**Follow-up**<br>• 2-12 years (mean 9 years)<br>• Completeness of follow-up not specified	**Epilepsy**<br>**Preterm with cerebral haemorrhage**<br>HR 42.4 95%CI (29.8, 60.3)<br>aHR 42.5 95 %CI (29.6, 60.5)<br><br>**SGA with cerebral haemorrhage**<br>HR 39.3 95%CI (5.51, 274.5)<br>aHR 38.7 95%CI (5.43, 275.5)
7	Davidovitch 2020[29]<br><br>Israel<br><br>Retrospective cohort study	**Population (n=4963)**<br>• VLBW infants ≤1500g<br>• Born 1999-2012<br><br>**Exposure**<br>• IVH grade 3-4 (n=256)<br>• PVL (n=200)<br>• Post-haemorrhagic hydrocephalus (n=152)<br><br>**Comparator**<br>• Unmatched<br>• No IVH grade 3-4 (n=4600)<br>• No PVL (n=3813)<br>• No post-haemorrhagic hydrocephalus (n=4810)<br><br>**Ascertainment/ definition**<br>• Israel national very low birthweight infant database linked to electronic medical records.<br>• Ultrasound diagnosis<br>• Papile classification	**Outcome**<br>• ASD<br><br>**Assessment/ measurement**<br>• Physical, neurological, and developmental assessment (by a qualified healthcare professional)<br>• Independent psychological assessment<br><br>**Follow-up**<br>• 8- 15 years (median 11.6)<br>• Only those linked to electronic medical records included	**ASD**<br>IVH n=10, 3.9%<br>No IVH n=103, 2.2% p=0.085<br><br>PVL n=5, 2.5%<br>No PVL n=88, 2.3% p=0.86<br><br>Post-haemorrhagic hydrocephalus n=7, 4.6%<br>No post-haemorrhagic hydrocephalus n=106, 2.2% p=0.051<br><br>IVH, PVL, post-haemorrhagic hydrocephalus or ROP n=27,23.9%<br>No brain injury n=571, 11.8% p<0.0001<br>aOR 1.62 95% CI (0.96–2.73)
8	Doyle 2000[70]<br><br>Australia	**Population**<br>• Birthweight 500–1499 g<br>• Born 1980-1981; 1992	**Outcomes**<br>• Survival<br>• Cerebral palsy	**Cerebral Palsy**<br><br>Grade of IVH

Confidential: For Review Only

	Prospective Cohort	**Exposure**<br>**1980s epoch**<br>• IVH grade 1 (n=18)<br>• IVH grade 2 (n=9)<br>• IVH grade 3 (n=7)<br>• IVH grade 4 (n=4)<br><br>**1992 epoch**<br>• IVH grade 1 (n=23)<br>• IVH grade 2 (n=10)<br>• IVH grade 3 (n=9)<br>• IVH grade 4 (n=1)<br><br>**Comparator**<br>• Unmatched<br>• No intracranial haemorrhage (n=223)<br>• 1980s epoch (n=110)<br>• 1992 epoch (n=113)<br><br>**Ascertainment/ definition**<br>• Ultrasound imaging<br>• Post-mortem examination<br>• Papile classification	**Measurement/assessment**<br>• Clinical assessment by blinded paediatricians<br>• Functional assessment<br><br>**Follow-up**<br>• 5 years<br>• 93% follow-up for 1980s epoch<br>• 94% follow-up for 1992 epoch	**1980s epoch**<br>No IVH n=5, 5%<br>IVH grade 3 n=2, 29%<br>IVH grade 4 n=0<br><br>**1992s epoch**<br>No IVH n=4, 4%<br>IVH grade 3 n=3, 33%<br>IVH grade 4 n=1, 100%
9	Hintz 2018[17]<br><br>USA<br><br>Retrospective cohort	**Population**<br>• Gestation 24-28 weeks<br>• Born 2005-2009<br><br>**Exposure**<br>MRI<br>• Mild WMI (n=223)<br>• Moderate WMI (n=51)<br>• Severe WMI (n=15)<br><br>• Any cerebellar lesion (n=57)<br><br>• Significant cerebellar lesion (n=39)<br><br>Early cranial ultrasound<br>• No IVH 3-4 or cPVL (n=341)<br>• IVH 3-4 or cPVL (n=32)<br><br>Late cranial ultrasound<br>• No porencephalic cyst, cPVL, moderate to severe ventricular enlargement or shunt (n=354)<br>• Porencephalic cyst, cPVL, moderate to severe ventricular enlargement or shunt (n=19)<br><br>**Comparator**<br>• No white matter injury on MRI (n=84)<br>• No cerebellar lesion on MRI (n=316)<br>• No IVH 3-4 or cPVL (n=32)<br>• Normal early cranial ultrasound (n=227)<br>• No porencephalic cyst, cPVL moderate to severe ventricular enlargement or shunt (n=19)<br>• Normal late cranial ultrasound (n=284)<br><br>**Ascertainment/ definition**<br>• NICHD neonatal research network (NEURO study and SUPPORT cohort)<br>• Two masked central imaging readers for all cranial ultrasounds and one for MRI<br>• All had cranial ultrasound and MRI (at 35-42 weeks)<br>• Unilateral and bilateral cranial ultrasound lesions combined	**Outcomes**<br>• Moderate to severe disability (composite)<br>• Minimal or no disability<br>• Cognitive<br>• Cerebral palsy<br>• Hearing<br>• Vision<br><br>**Measurement/ assessment**<br>• WISC IV<br>• Neurological exam<br>• GMFCS<br>• Clinical examination<br>• Parental report<br><br>**Follow-up**<br>• 6-7 years<br>• 83.3% follow-up of survivors	**White matter injury**<br>**Moderate to severe disability**<br>No white matter injury, n=8, 9%<br>Mild white matter injury, n=27, 12%<br>Moderate white matter injury, n=8, 15%<br>Severe white matter injury, n=14, 82%<br>p<0.0001<br><br>Moderate or severe white matter injury<br>aOR 1.1 95% CI (0.42, 2.92)<br><br>**Minimal or no disability**<br>No white matter injury, n=47, 55%<br>Mild white matter injury, n=88, 224%<br>Moderate white matter injury, n=15, 28%<br>Severe white matter injury, n=0, 0%<br>p<0.0001<br><br>**Cognitive impairment (FSIQ mean (SD))**<br>No white matter injury, 90.1 (15.5)<br>Mild white matter injury, 85.9 (16.8)<br>Moderate white matter injury, 84 (17)<br>Severe white matter injury, 62.7 (19.6)<br>p<0.0001<br><br>**Cognitive impairment FSIQ <70**<br>No white matter injury, n=7, 8%<br>Mild white matter injury, n=25, 11%<br>Moderate white matter injury, n=6, 12%<br>Severe white matter injury, n=9, 60%<br>p<0.0001<br><br>Moderate or severe white matter injury<br>aOR 1.14 95% CI (0.39, 3.26)<br><br>**Cognitive impairment FSIQ <85**<br>No white matter injury, n=27, 32%<br>Mild white matter injury, n=100, 45%<br>Moderate white matter injury, n=29, 57%<br>Severe white matter injury, n=13, 87%<br>p<0.0001<br><br>**No cognitive impairment FSIQ ≥85**<br>No white matter injury, n=57, 68%<br>Mild white matter injury, n=123, 55%<br>Moderate white matter injury, n=22, 43%<br>Severe white matter injury, n=2, 13%<br>p<0.0001<br><br>**Any cerebral palsy**<br>No white matter injury, n=2, 2%<br>Mild white matter injury, n=6, 3%<br>Moderate white matter injury, n=4, 7%<br>Severe white matter injury, n=10, 59%<br>p<0.0001<br><br>**Cerebral palsy with GMFCS ≥2**<br>No white matter injury, n=0, 0%<br>Mild white matter injury, n=1, 0%<br>Moderate white matter injury, n=1, 2%<br>Severe white matter injury, n=4, 24%<br>p<0.0001<br><br>**Cerebellar lesions**<br>**Moderate to severe disability**<br>No cerebellar lesion, n=37, 12%<br>Any cerebellar lesion, n=20, 33% p<0.0001<br>Significant cerebellar lesion, n=15, 36%<br><br>Significant cerebellar lesions<br>aOR 2.71 95% CI (1.09, 6.71)<br><br>**Minimal or no disability**

Confidential: For Review Only

				No cerebellar lesion, n=135, 42%<br>Any cerebellar lesion n=15, 25% p<0.0001<br>Significant cerebellar lesion, n=15, 36%<br><br>**Cognitive impairment (FSIQ mean (SD))**<br>No cerebellar lesion, 87 (16.5)<br>Any cerebellar lesion 78.4 (20) p=0.001<br>Significant cerebellar lesion 76.8 (20.4)<br><br>**Cognitive impairment FSIQ <70**<br>No cerebellar lesion, n=32, 10%<br>Any cerebellar lesion, n=15, 26% p=0.001<br>Significant cerebellar lesion, n=10, 26%<br><br>Significant cerebellar lesions<br>aOR 1.96 95% CI (0.72, 5.36)<br><br>**Cognitive impairment FSIQ <85**<br>No cerebellar lesion, n=136, 43%<br>Any cerebellar lesion, n=33, 58% p=0.038<br>Significant cerebellar lesion, n=22, 56%<br><br>**No cognitive impairment FSIQ ≥85**<br>No cerebellar lesion, n=180, 57%<br>Any cerebellar lesion, n=24, 42% P=0.038<br>Significant cerebellar lesion, n=17, 44%<br><br>**Any cerebral palsy**<br>No cerebellar lesion, n=13, 4%<br>Any cerebellar lesion, n=9, 15% p=0.001<br>Significant cerebellar lesion, n=9, 21%<br><br>**Cerebral palsy with GMFCS ≥2**<br>No cerebellar lesion, n=3, 1%<br>Any cerebellar lesion, n=3, 5% p=0.19<br>Significant cerebellar lesion, n=3, 7%<br><br>**Early cranial ultrasound abnormalities**<br>**Moderate to severe disability**<br>No IVH 3-4 or cPVL, n=43, 12%<br>IVH 3-4 or cPVL, n=14, 42% p<0.0001<br>Normal scan, n=35, 12%<br>aOR 0.61 95% CI (0.14, 2.59)<br><br>**Minimal or no disability**<br>No IVH 3-4 or cPVL, n=143, 41%<br>IVH 3-4 or cPVL, n=7, 21% p<0.0001<br>Normal scan, n=120, 43%<br><br>**Cognitive impairment, FSIQ mean (SD)**<br>No IVH 3-4 or cPVL, 86.4 (17)<br>IVH 3-4 or cPVL, 77.9 (19.1) p=0.008<br>Normal scan, 86 (16.7)<br><br>**Cognitive impairment FSIQ <70**<br>No IVH 3-4 or cPVL, n=38, 11%<br>IVH 3-4 or cPVL, n=9, 28% p=0.006<br>Normal scan, n=31, 11%<br>aOR 0.42 95% CI (0.07, 2.33)<br><br>**Cognitive impairment FSIQ <85**<br>No IVH 3-4 or cPVL, n=149, 44%<br>IVH 3-4 or cPVL, n=20, 63% p=0.041<br>Normal scan, n=123, 44%<br><br>**No cognitive impairment FSIQ ≥85**<br>No IVH 3-4 or cPVL, n=192, 56%<br>IVH 3-4 or cPVL, n=12, 38% p=0.041<br>Normal scan, n=154, 56%<br><br>**Any cerebral palsy**<br>No IVH 3-4 or cPVL, n=149, 44%<br>IVH 3-4 or cPVL, n=20, 63% p=0.041<br>Normal scan, n=123, 44%<br><br>**Cerebral palsy with GMFCS ≥2**<br>No IVH 3-4 or cPVL, n=3, 1%<br>IVH 3-4 or cPVL, n=3, 9% p<0.0001<br>Normal scan, n=2, 1%<br><br>**Late cranial ultrasound abnormalities**<br>**Moderate to severe disability**<br>No porencephalic cyst, cPVL, moderate to severe ventricular enlargement or shunt, n=40, 11%<br>Porencephalic cyst, cPVL, moderate to severe ventricular enlargement or shunt, n=17, 77% p<0.0001<br>Normal scan, n=27, 10%<br>aOR 27.85 95% CI (6.03, 128.68)<br><br>**Minimal or no disability**<br>No porencephalic cyst, cPVL, moderate to severe ventricular enlargement or shunt, n=149, 42%<br>Porencephalic cyst, cPVL, moderate to severe ventricular enlargement or shunt, n=1, 5% P<0.0001<br>Normal scan, n=117, 43%<br><br>**Cognitive impairment (FSIQ mean (SD))**<br>No porencephalic cyst, cPVL, moderate to severe ventricular enlargement or shunt, 86.7 (16.7)<br>Porencephalic cyst, cPVL, moderate to severe ventricular enlargement or shunt, 65.9 (18.7) P<0.0001

Confidential: For Review Only

				Normal scan, 87 (16.1)<br><br>**Cognitive impairment FSIQ <70**<br>No porencephalic cyst, cPVL, moderate to severe ventricular enlargement or shunt, n=36, 10%<br>Porencephalic cyst, cPVL, moderate to severe ventricular enlargement or shunt, n=11, 58% p<0.0001<br>Normal scan, n=24, 9%<br>aOR 20.05 95% CI (3.63, 110.84)<br><br>**Cognitive impairment FSIQ <85**<br>No porencephalic cyst, cPVL, moderate to severe ventricular enlargement or shunt, n=153, 43%<br>Porencephalic cyst, cPVL, moderate to severe ventricular enlargement or shunt, n=16, 84% p<0.0001<br>Normal scan, n=118, 43%<br><br>**No cognitive impairment FSIQ ≥85**<br>No porencephalic cyst, cPVL, moderate to severe ventricular enlargement or shunt, n=201, 57%<br>Porencephalic cyst, cPVL, moderate to severe ventricular enlargement or shunt, n=3, 16% p<0.0001<br>Normal scan, n=156, 57%<br><br>**Any cerebral palsy**<br>No porencephalic cyst, cPVL, moderate to severe ventricular enlargement or shunt, n=10, 3%<br>Porencephalic cyst, cPVL, moderate to severe ventricular enlargement or shunt, n=12, 50% p<0.0001<br>Normal scan, n=6, 2%<br><br>**Cerebral palsy with GMFCS ≥2**<br>No porencephalic cyst, cPVL, moderate to severe ventricular enlargement or shunt, n=2, 1%<br>Porencephalic cyst, cPVL, moderate to severe ventricular enlargement or shunt, n=4, 17% p<0.0001<br>Normal scan, n=1, 0%
10	Hirovonen, 2017[22]<br><br>Finland<br><br>Retrospective cohort	**Population**<br>• Gestation >22 weeks<br>• Birth weight >500g<br>• Born 1991-2008<br><br>**Exposure (n=557)**<br>• Intracranial haemorrhage<br><br>**Comparison (n=708,977)**<br>• No intracranial haemorrhage<br>• ICD code<br><br>**Ascertainment/ definition**<br>• Finnish national register<br>• ICD codes	**Outcomes**<br>• Cognitive<br><br>**Measurement/ assessment**<br>• ICD 9 and 10 codes<br>• BSID 1993<br>• Finnish WISC<br><br>**Follow-up**<br>• 7 years<br>• 98% follow-up	**Any intellectual disability after intracranial haemorrhage (HR (95%CI); p-value)**<br>Very preterm infants 2.92 (1.58–5.41); p= 0.001<br>Moderately preterm 5.59 (1.57–19.9); p= 0.008<br>Late preterm 4.58 (1.36–15.4); p= 0.014<br>Term 2.94 (1.08-8); p=0.035
11	Hollebrandse 2021[19]<br><br>Australia<br><br>Retrospective cohort	**Population**<br>• Gestation <28 weeks<br>• Born 1991-1992, 1997, 2005<br><br>**Exposure**<br>• IVH grade 1 n=80<br>• IVH grade 2 n=53<br>• IVH grade 3 n=23<br>• IVH grade 4 n=12<br><br>**Comparator**<br>• Unmatched<br>• Preterm infants without IVH n=331<br><br>**Ascertainment/ definition**<br>• Ultrasound diagnosis<br>• Worst grade of IVH<br>• Papile classification	**Outcomes**<br>• Cognitive<br>• Motor<br>• Cerebral palsy<br><br>**Assessment/ measurement**<br>• WISC III (1991-1992 cohort)<br>• WISC IV (1997 cohort)<br>• Differential Abilities Scale 2nd edition (2005 cohort)<br>• WRAT III (1991-92; 1997 cohorts)<br>• WRAT IV (2005 cohort)<br>• Behaviour rating inventory of executive functioning (parent-completed)<br>• Movement ABC 1st edition (1991-1992 and 1997 cohorts)<br>• Movement ABC 2nd edition (2005 cohort)<br>• GMFCS (1997 and 2005 cohort)<br>• Blinded assessment<br><br>**Follow-up**<br>• 8 years<br>• Follow-up 85-91.4%	**Cognitive**<br>**IQ score <-2 SD**<br>IVH grade 4 n=5, 42% p=0.08 (X^2 trend)<br>IVH grade 3 n=5, 22%<br>No IVH n=41, 12%<br><br>IVH 3-4: OR 2.68 95% CI (1.21, 5.94) p=0.01<br><br>**Impaired executive function**<br>**Global executive composite ≥65**<br>IVH grade 4 n=2, 18% p=0.78 (X^2 trend)<br>IVH grade 3 n=4, 18%<br>No IVH n=49, 16%<br><br>IVH 3-4: OR 1.17 95% CI (0.46, 2.97) p=0.75<br><br>**Behavioural regulation index ≥65**<br>IVH grade 4 n=2, 18% p=0.21 (X^2 trend)<br>IVH grade 3 n=6, 27%<br>No IVH n=46, 15%<br><br>IVH 3-4: OR 1.76 95% CI (0.75, 4.11) p=0.2<br><br>**Metacognition index ≥65**<br>IVH grade 4 n=3, 27% p=0.1 (X^2 trend)<br>IVH grade 3 n=5, 23%<br>No IVH n=48, 16%<br><br>IVH 3-4: OR 1.73 95% CI (0.74, 4.06) p=0.21<br><br>**Impaired academic skills (any academic skill <-2SD)**<br>IVH grade 4 n=7, 64% p<0.001 (X^2 trend)<br>IVH grade 3 n=5, 24%<br>No IVH n=50, 16%<br><br>IVH 3-4: OR 2.91 95% CI (1.35, 6.27) p=0.006<br><br>**Impaired reading <-2SD**<br>IVH grade 4 n=6, 55% p=0.002 (X^2 trend)<br>IVH grade 3 n=4, 19%<br>No IVH n=21, 10%<br><br>IVH 3-4: OR 3.62 95% CI (1.59, 8.24) p=0.002<br><br>**Impaired spelling <- 2 SD**<br>IVH grade 4 n=5, 45% p=0.011 (X^2 trend)<br>IVH grade 3 n=3, 14%

Confidential: For Review Only

Confidential: For Review Only

				No IVH n=21, 7%<br><br>IVH 3-4: OR 4.48 95% CI (1.8, 11.2) p=0.001<br><br>**Impaired arithmetic < -2 SD**<br>IVH grade 4 n=5, 45% p=0.09 (X^2 trend)<br>IVH grade 3 n=4, 19%<br>No IVH n=38, 12%<br><br>IVH 3-4: OR 2.79 95% CI (1.2, 6.48) p=0.017<br><br>**Motor and cerebral palsy**<br>**Any motor dysfunction (cerebral palsy or MABC <5th centile)**<br>IVH grade 4 n=11, 92% p<0.001 (X^2 trend)<br>IVH grade 3 n=10, 43%<br>No IVH n=81, 24%<br><br>IVH 3-4: OR 4.45 95% CI (2.18, 9.08) p<0.001<br><br>**Cerebral palsy**<br>IVH grade 4 n=9, 75% p<0.001(X^2 trend)<br>IVH grade 3 n=6, 26%<br>No IVH n=26, 8%<br><br>IVH 3-4: OR 8.8 95% CI (4.03, 19.2) p<0.001<br><br>**MABC <5th percentile (for the 2005 cohort)**<br>IVH grade 4 n=11, 92% p<0.001 (X^2 trend)<br>IVH grade 3 n=9, 45%<br>No IVH n=79, 26%<br><br>IVH 3-4: OR 4.7 95% CI (2.21, 9.97) p<0.001
12	Hreinsdottir 2018[48]<br><br>Sweden<br><br>Prospective cohort study	**Population**<br>• Born 2004-2007<br>• Gestation <32 years<br><br>**Exposure (n=9)**<br>• IVH grade 3-4 and/ or PVL<br><br>**Comparator (n=99)**<br>• Unmatched<br>• No IVH grade 3-4 or PVL<br><br>**Ascertainment/ definition**<br>• Ultrasound imaging performed by paediatric radiologist<br>• Papile classification for IVH<br>• PVL defined by size, laterality and as cystic of diffuse	**Outcomes**<br>• Visual impairment<br><br>**Assessment/ measurement**<br>• Linear visual acuity (Lea Hyvarinen chart)<br>• Cover test<br>• Refraction<br><br>**Follow-up**<br>• 6.5 years<br>• 78% follow-up	**Vision**<br>**Subnormal visual acuity**<br>IVH 3-4 and or PVL<br>OR 1.11 95% CI (0.25, 4.83) p=0.891<br><br>**Contrast sensitivity**<br>IVH 3-4 and or PVL<br>OR 1.87 95% CI (0.43, 8.17) p=0.403<br><br>**Refractive error**<br>IVH 3-4 and or PVL<br>OR 2.5 95% CI (0.55, 11.41) p=0.237<br><br>**Manifest strabismus**<br>IVH 3-4 and or PVL<br>OR 4 95% CI (0.65, 24.55) p=0.134<br><br>**Composite score 1: Visual acuity with both eyes of less than 0.3, significant refractive error in the better eye and manifest strabismus**<br>IVH 3-4 and or PVL<br>OR 3.63 95% CI (0.86, 15.41) p=0.08<br>aOR 4.95 95% CI (0.65, 37.48) p=0.121<br><br>**Composite score 2: Visual acuity in worse eye of less than 0.3, significant refractive error in worse eye according and manifest strabismus**<br>IVH 3-4 and or PVL<br>OR 5.67 95% CI (1.34, 24.07) p=0.019<br>aOR 10.4 95% CI (1.23, 88) p=0.032<br><br>**Composite score 3: Visual acuity with both eyes of less than 0.5, significant refractive error in the better eye, manifest strabismus, negative stereopsis and contrast sensitivity less than 0.4**<br>IVH 3-4 and or PVL<br>OR 7.6 95% CI (1.7, 34) p=0.008<br>aOR 18.19 95% CI (2.15, 154.05) p=0.008<br><br>**Composite score 4: Visual acuity with both eyes of 0.8 or less, significant refractive error in the better eye, manifest strabismus, negative stereopsis and CS less than 0.5**<br>IVH 3-4 and or PVL<br>OR 4.63 95% CI (0.9, 23.85) p=0.067<br>a6.23 95% CI (1.15, 33.83) p=0.034
13	Jansen 2020[23]<br><br>Netherlands<br><br>Prospective cohort study	**Population**<br>• Gestation <32 weeks<br>• Admitted 2006-2007<br><br>**Exposure**<br>• Mild WMI (n=18)<br>• Moderate WMI (n=14)<br>• Severe WMI (n=8)<br>• Mild cerebellar injury (n=11)<br>• Moderate cerebellar injury (n=4)<br>• Severe cerebellar injury (n=6)<br><br>**Comparator**<br>• Unmatched<br>• No WMI (n=46)<br>• No cerebellar injury (n=65)<br><br>**Ascertainment/ definition**<br>• Ultrasound imaging and term MRI<br>• Imaging reviewed by two blinded experienced investigators (neonatologists or radiologists)	**Outcomes**<br>• Cognitive<br><br>**Assessment/ measurement**<br>• National standardised achievement tests<br><br>**Follow-up**<br>• 9-10 years<br>• 77% follow-up	**Cognitive**<br>**Reading comprehension**<br>Moderate-severe WMI vs. no injury<br>B 0.241 p=0.483<br><br>Moderate-severe cerebellar injury vs. no injury<br>B 0.799 p=0.325<br><br>**Spelling**<br>Moderate-severe WMI vs. no injury<br>B 1.076 p=0.075<br><br>Moderate-severe cerebellar injury vs. no injury<br>B 1.293 p= 0.115<br><br>**Mathematics**<br>Moderate-severe WMI vs. no injury<br>B 1.856 p=0.003<br><br>Moderate-severe cerebellar injury vs. no injury<br>B 1.504 p=0.088

14	Kaur 2020[32]<br><br>Canada<br><br>Retrospective cohort study	**Population**<br>• Preterm and term infants<br>• Born 2006-2016<br><br>**Exposure**<br>• IVH grade 1 (n=811)<br>• IVH grade 2 (n=186)<br>• IVH grade 3-4 (n=194)<br>• Preterm haemorrhage (n=1139)<br><br>**Comparator**<br>• Unmatched<br>• No IVH (n=793, 062)<br>• Preterm no haemorrhage (n=50, 185)<br><br>**Ascertainment/ definition**<br>• ICD 10 codes (based on ultrasound or MRI imaging)<br>• Papile classification	**Outcome**<br>• Reason for hospitalisation<br><br>**Assessment/ measurement**<br>• ICD 10 codes<br><br>**Follow-up**<br>• 12 years<br>• Completeness of follow-up not specified	**<u>Incidence of hospitalisation for:</u>**<br>**Cerebral palsy, n, incident rate per 1,000 person years (95%CI)**<br>IVH n=57, 6.8 (5.3, 8.8)<br>No haemorrhage n=432, 0.1 (0.1, 0.1)<br>Hazard ratio: 4.78 95% CI (3.21, 7.13)<br><br>IVH grade 3-4 n=24 HR 14.78 95% CI (8.72-25.06)<br><br>**Ophthalmologic, n, incident rate per 1,000 person years (95%CI)**<br>IVH n=91 11.1 (9, 13.6)<br>No haemorrhage n=6773, 1.2 (1.2, 1.3)<br>HR 3.01 95% CI (2.32, 3.89)<br><br>IVH grade 3-4 n=32 HR 7.87 95% CI (5.31-11.67)<br><br>**Otologic n, incident rate per 1,000 person years (95%CI)**<br>IVH n=328, 46.7 (41.9, 52)<br>No haemorrhage n=102,153 22.1 (22, 22.2)<br>HR 1.19 95% CI (1.06, 1.34)<br><br>IVH grade 3-4 n=202 HR 1.07 95% CI (0.79-1.46)
15	Kiechl-Kohlendorfer 2013[28]<br><br>Austria<br><br>Prospective cohort	**Population**<br>• Gestation <32 weeks<br>• Born 2003-2006<br><br>**Exposure**<br>• Intracranial haemorrhage (all grades) (n=24)<br>• Intracranial haemorrhage grade 3-4 (n=4)<br>• PVL (n=2)<br>• Intraparenchymal echodense lesions (n=2)<br><br>**Comparator**<br>• Unmatched<br><br>**Ascertainment/ definition**<br>• Ultrasound imaging<br>• Papile classification	**Outcomes**<br>• Cognitive<br><br>**Measurement/assessment**<br>• Physical examination<br>• Hannover-Wechsler Intelligence Test for preschool children, third edition<br>• WPPSI<br>• Snijders-Oomen Nonverbal Intelligence Test<br>• TEDI-MATH<br><br>**Follow-up**<br>• 5 years<br>• 72.2% follow-up	**Delayed numerical skills**<br>Intracranial haemorrhage (all grades) n=11, 40,7%<br>aOR 4.66 95% CI (1.56, 13.93) p=0.007<br><br>Intracranial haemorrhage grade 3-4 n=3, 11.1%<br>PVL n=2, 7.4%<br>Intraparenchymal echodense lesions n=0
16	Klebermass-Schrehof 2012[20]<br><br>Austria<br><br>Prospective cohort	**Population**<br>• Gestation <32 weeks<br>• Admitted to NICU 1994-2005<br><br>**Exposure**<br>• IVH grade 1 (n=37)<br>• IVH grade 2 (n=84)<br>• IVH grade 3 (n=18)<br>• IVH grade 4 (n=12)<br><br>**Comparator (n=320)**<br>• Unmatched<br>• No IVH<br><br>**Ascertainment/ definition**<br>• Ultrasound diagnosis<br>• Most severe scan used<br>• Papile classification	**Outcomes**<br>• Neurosensory impairment (composite)<br>• Motor<br>• Cerebral palsy<br>• Language<br>• Visual<br>• Hearing<br><br>**Measurement/assessment**<br>• BSID II (MDI, PDI)<br>• K-ABC<br>• Beery-Buktenica Developmental Test of VMI<br>• Clinical assessment<br><br>**Follow-up**<br>• 5 years (1 ,2, and 3.5 years)<br>• Only those with follow-up included (loss to follow-up not specified)	**<u>Outcomes at 5.5 years</u>**<br><br>**<u>Group 1: infants born < 28 weeks' gestation</u>**<br>**KABC <70**<br>No IVH, 7.6%<br>IVH grade 3, 33.3%<br>IVH grade 4, 50%<br><br>**KABC mean (SD)**<br>No IVH, 91.5 (15.1)<br>IVH grade 3, 88.6 (11.1) p=not significant<br>IVH grade 4, 88.5 (10.6) p= not significant<br><br>**VMI mean (SD)**<br>No IVH, 92.7 (20)<br>IVH grade 3, 67.5 (14) p=0.04<br>IVH grade 4, 76 (26.8) p=0.04<br><br>**Cerebral palsy**<br>No IVH, 14.3%<br>IVH grade 3, 63.6% p<0.01<br>IVH grade 4, 90.9% p<0.01<br><br>**Visual impairment**<br>No IVH, 7.5%<br>IVH grade 3, 45.5%, p=0.03<br>IVH grade 4, 90.9% p<0.01<br><br>**Acoustic impairment**<br>No IVH, 2.2%<br>IVH grade 3, 0% p= not significant<br>IVH grade 4, 0% p= not significant
17	Koc 2016[24]<br><br>Turkey<br><br>Retrospective cohort	**Population (n=90)**<br>• Gestation <32 weeks<br>• Birthweight <1500g<br>• Born 2001<br><br>**Exposure**<br>• IVH grade 1-2 (n= 7)<br>• IVH grade 3-4 (n= 8)<br><br>**Comparator**<br>• No IVH (n=75)<br><br>**Ascertainment/ definition**<br>• Neonatal unit database and medical records	**Outcomes**<br>• Cognitive<br><br>**Measurement/ assessment**<br>• WISC-R<br><br>**Follow-up**<br>• 5.9-7.9 years<br>• 100% follow-up	**<u>WISC-R score <85</u>**<br>IVH (n=7; 46.7%)<br>No IVH (n= 25; 33.3%)<br><br>**<u>WISC-R score >85</u>**<br>IVH grade (n=8; 13.8%)<br>No IVH (n= 50; 84.2%)<br><br>p=0.381
18	Martinez-Cruz 2008[45]<br><br>Mexico<br><br>Case control	**Population**<br>• Gestation <34 weeks<br>• Birthweight <1500g<br>• Born 1990-2005<br><br>**Exposure (n=103)**<br>• IVH	**Outcomes**<br>• Sensorineural hearing loss<br><br>**Measurement/ assessment**<br>• Brainstem auditory evoked potentials<br>• Transient auditory evoked otoacoustic emissions<br>• Behavioural hearing evaluation	**IVH**<br>Sensorineural hearing loss (n=71; 48.6%)<br>No sensorineural hearing loss (n=32; 11.8%)<br><br>**<u>Multivariate logistic regression of risk factors for sensorineural hearing loss</u>**<br>IVH: aOR 7.1 95% CI (4.34, 11.6) p<0.000

Confidential: For Review Only

		**Comparator (n=315)**<br>• No IVH<br><br>**Ascertainment/ definition**<br>• Medical records<br>• Ultrasound diagnosis.<br>• Papile classification.	• Free field audiometry<br>• Tympanometry<br>• Pure Tone Audiometry<br><br>**Follow-up**<br>• Mean age 7.8±3.7 years<br>• 100% follow-up (case control)	
19	Neubauer 2008[12]<br><br>Germany<br><br>Prospective cohort	**Population**<br>• Birthweight <1000g<br>• Born 1993-1998<br><br>**Exposure**<br>• IVH grade 1-2 (n=26)<br>• IVH grade 3-4, PVL (n=18)<br><br>**Comparator**<br>• Unmatched<br>• No IVH or PVL (n=91)<br><br>**Ascertainment/ definition**<br>• Ultrasound diagnosis<br>• Papile classification	**Outcomes**<br>• Neurodevelopmental impairment (composite)<br><br>**Measurement/assessment**<br>• Modified Touwen test<br>• K-ABC<br>• Snijders-Oomen Non-Verbal Intelligence Test<br>• Hamburg-Wechsler Intelligence Test for Children<br><br>**Follow-up**<br>• 10 years<br>• 79% follow-up	**<u>Logistic regression for major impairment vs. normal development or minor impairment at school age</u>**<br><br>**Grade 3-4 IVH or PVL**<br>Normal (n=4, 22%)<br>Minor (n=2, 11%)<br>Major (n=12, 67%)<br>Risk of impairment: OR 2.46 95% CI (0.52–11.7)
20	Piris Borregas 2019[13]<br><br>Spain<br><br>Retrospective cohort study	**Population (n=1001)**<br>• Birthweight 500-1250g<br>• Born 1991-2008<br><br>**Exposure**<br>• Severe brain injury (IVH grade 3-4, ventriculomegaly III, PVL or intraparenchymal echodense lesion grade 3 or greater)<br><br>**Comparator**<br>• Unmatched<br><br>**Ascertainment/ definition**<br>• Neonatal database<br>• Ultrasound diagnosis<br>• Papile classification	**Outcomes**<br>• Neurodevelopment (composite)<br>• Cognitive<br>• Motor<br>• Hearing impairment<br>• Visual impairment<br><br>**Assessment/ measurement**<br>• GMFCS<br><br>**Follow-up**<br>• 7 years	**<u>Poor neurodevelopmental outcome</u>**<br>**Severe brain injury**, n=46, 32%<br>**No severe brain injury**, n=208, 24%<br>OR 1.41 95% CI (0.94, 2.10) p=0.09<br>Independent OR 2.02 95% CI (1.22, 3.31) p=0.18<br><br>**Severe brain injury (birthweight 500-1000g)**<br>Independent OR 2.02 95% CI (1.22, 3.31)
21	Pittet 2019[25]<br><br>Switzerland<br><br>Prospective cohort study	**Population**<br>• Gestation <30 weeks<br>• Born 2006<br><br>**Exposure**<br>• IVH grade 3-4 or cPVL (n=22)<br><br>**Comparator**<br>• Unmatched<br>• No IVH grade 3-4 or cPVL (n=213)<br><br>**Ascertainment/ definition**<br>• Swiss neonatal network follow-up group	**Outcomes**<br>• Cognitive<br>• Cerebral palsy<br>• Visual impairment<br>• Hearing impairment<br><br>**Assessment/ measurement**<br>• Kaufman ABC<br>• Neurological exam<br>• GMFCS<br><br>**Follow-up**<br>• 5.5 – 6 years<br>• 81% follow-up	**<u>Cognitive (K-ABC – MPC score < 1SD)</u>**<br>IVH 3-4 or PVL<br>OR 2.9 95% CI (1, 8.2) p=0.04<br>aOR 2.3 95% CI (0.7, 7.7) p=0.15<br><br>**<u>Use of early intervention/ therapy service</u>**<br>IVH 3-4 or cPVL aOR 2.7 95% CI (1.3, 5.7)
22	Sherlock 2005[14]<br><br>Australia<br><br>Prospective cohort	**Population**<br>• Gestation <28 weeks<br>• Birthweight <1000g<br>• Survivors born 1991-1992<br><br>**Exposure**<br>• IVH Grade 1 (n=47)<br>• IVH Grade 2 (n= 25)<br>• IVH Grade 3 (n= 12)<br>• IVH Grade 4 (n= 6)<br><br>**Comparator**<br>• Matched on sex, mother's country of birth, and health insurance status<br>• Extremely low birth weight or very preterm infants without IVH (n=180)<br><br>**Ascertainment/ definition**<br>• Enrolled in Victorian Collaborative Study<br>• Ultrasound diagnosis (at least one scan by a certified sonographer)<br>• Worst grade of IVH on either side used<br>• Papile classification	**Outcomes**<br>• Disability (composite)<br>• Neurosensory disability (composite)<br>• Cognitive<br>• Motor<br>• Cerebral palsy<br>• Speech and language<br>• Visual impairment<br>• Hearing impairment<br><br>**Measurement/assessment**<br>• Medical assessment<br>• Movement ABC<br>• WISC-III<br>• Tower of London<br>• Rey Complex Figure<br>• WRAT<br><br>**Follow-up**<br>• Mean 8.7 years<br>• 92.3% follow-up	**Abnormal movement**<br>No IVH (n=39, 22.5%)<br>Grade 1 IVH (n=11, 25%)<br>Grade 2 IVH (n=6, 30%)<br>Grade 3 IVH (n=3, 27.3%)<br>Grade 4 IVH (n=4, 100%)<br>X^2 linear trend = 5.3; P = 0.021<br><br>**Cerebral palsy**<br>No IVH (n=12, 6.7%)<br>Grade 1 IVH (n=3, 6.4%)<br>Grade 2 IVH (n=6, 24%)<br>Grade 3 IVH (n=2, 16.7%)<br>Grade 4 IVH (n=6, 100%)<br>X^2 linear trend = 31.7; p <0.0001<br><br>**Moderate to severe cerebral palsy**<br>No IVH (n=4, 2.2%)<br>Grade 1 IVH (n=0, 0%)<br>Grade 2 IVH (n=4, 15%)<br>Grade 3 IVH (n=1, 8.3%)<br>Grade 4 IVH (n=5, 83.3%)<br>X^2 linear trend = 40.8; p <0.0001<br><br>**Major neurosensory disability**<br>No IVH (n=28, 15.6%)<br>Grade 1 IVH (n=5, 10.6%)<br>Grade 2 IVH (n=5, 20%)<br>Grade 3 IVH (n=1, 8.3%)<br>Grade 4 IVH (n=6, 100%)<br>X^2 linear trend = 6.9; p = 0.009<br><br>**IQ score mean (SD)**<br>No IVH 0.71 (1.25)<br>Grade 1 IVH 0.76 (1.32)<br>Grade 2 IVH 0.71 (1.12)<br>Grade 3 IVH 1.21 (1.13)<br>Grade 4 IVH 3.28 (0.88)<br>ANOVA F4,265 = 6.7; p<0.0001<br><br>**Verbal comprehension index mean (SD)**<br>No IVH 96.6 (16.2)<br>Grade 1 IVH 96.3 (15.7)<br>Grade 2 IVH 99.6 (12.8)<br>Grade 3 IVH 93.1 (15.4)

Confidential: For Review Only

				Grade 4 IVH 74.3 (12.7)<br>ANOVA F4,251 = 1.8; p = 0.12<br><br>**Perceptual organisation index mean (SD)**<br>No IVH 98.5 (16.3)<br>Grade 1 IVH 98.2 (15.7)<br>Grade 2 IVH 96.9 (14.8)<br>Grade 3 IVH 91.6 (12.7)<br>Grade 4 IVH 71.7 (11.1)<br>ANOVA F4,249 = 2.5; p = 0.042<br><br>**Freedom from distractibility index mean (SD)**<br>No IVH 92.3 (114.9)<br>Grade 1 IVH 95.5 (15.0)<br>Grade 2 IVH 97.7 (12.8)<br>Grade 3 IVH 94.9 (17.4)<br>Grade 4 IVH 71.0 (3.5)<br>ANOVA F4,250 = 2.8; p = 0.026<br><br>**Processing speed index mean (SD)**<br>No IVH 99.5 (15.8)<br>Grade 1 IVH 99.1 (16.6)<br>Grade 2 IVH 99.3 (13.0)<br>Grade 3 IVH 94.9 (19.3)<br>Grade 4 IVH 71.0 (9.5)<br>ANOVA F4,245 = 2.7; p = 0.033<br><br>**Tower of London (executive function) raw score mean (SD)**<br>No IVH 73.3 (14.4)<br>Grade 1 IVH 71.5 (12.4)<br>Grade 2 IVH 71.1 (20.4)<br>Grade 3 IVH 66.5 (8.3)<br>Grade 4 IVH 54.3 (22.0)<br>ANOVA F4,244 = 1.8; p = 0.13<br><br>**Rey complex figure (executive function) raw score mean (SD)**<br>No IVH 22.5 (7.5)<br>Grade 1 IVH 23.1 (7.4)<br>Grade 2 IVH 24.2 (5.8)<br>Grade 3 IVH 19.3 (8.3)<br>Grade 4 IVH 11.2 (9.8)<br>ANOVA F4,242 = 2.6; p = 0.037<br><br>**Wide range achievements test score mean (SD)**<br>**Reading**<br>No IVH 95.2 (15.7)<br>Grade 1 IVH 102.7 (15.4)<br>Grade 2 IVH 99.0 (14.2)<br>Grade 3 IVH 98.1 (11.9)<br>Grade 4 IVH 70.5g (20.9)<br>ANOVA F4,251 = 5.1; p = 0.001<br><br>**Spelling**<br>No IVH 93.6 (12.4)<br>Grade 1 IVH 97.8 (12.3)<br>Grade 2 IVH 95.9 (10.8)<br>Grade 3 IVH 96.8 (11.9)<br>Grade 4 IVH 73.5 (20.0)<br>ANOVA F4,250 = 4.0; p = 0.003<br><br>**Arithmetic**<br>No IVH 88.3 (14.3)<br>Grade 1 IVH 93.6 (14.9)<br>Grade 2 IVH 92.6 (10.6)<br>Grade 3 IVH 89.1 (10.1)<br>Grade 4 IVH 65.5 (14.5)<br>ANOVA F4,248 = 4.5; p = 0.002<br><br>**Cognitive test scores (compared to normal birthweight controls)**<br>**IQ score <1 SD from the mean (n, %)**<br>No IVH n=64 (35.6%)<br>Grade 1 IVH n=18 (38.3%)<br>Grade 2 IVH n=9 (36%)<br>Grade 3 IVH n=7 (58.3%)<br>Grade 4 IVH n=6(100%)<br>X^2 linear trend=6.8; P=0.009<br><br>**Wide range achievements test score <1 SD from the mean, n (%)**<br>**Low reading**<br>No IVH n=42 (24.4%)<br>Grade 1 IVH n=6 (13.3%)<br>Grade 2 IVH n=5 (20.8%)<br>Grade 3 IVH n=2 (18.2%)<br>Grade 4 IVH n=3 (75%)<br>X^2 linear trend=0.1; p=0.77<br><br>**Low spelling**<br>No IVH n=33 (19.2%)<br>Grade 1 IVH n=6 (13.6%)<br>Grade 2 IVH n=2 (8.3%)<br>Grade 3 IVH n=3 (27.3%)<br>Grade 4 IVH n=3 (75%)<br>X^2 linear trend=0.7; p=0.39<br><br>**Low arithmetic**<br>No IVH n=47 (27.6%)<br>Grade 1 IVH n=9 (20.5%)<br>Grade 2 IVH n=2 (8.3%)<br>Grade 3 IVH n=3 (27.3%)<br>Grade 4 IVH n=4 (100%)<br>X^2 linear trend=0.1; p=0.79

Confidential: For Review Only

23	Tymofiyeva 2018[33]<br><br>USA<br><br>Prospective cohort	**Population (n=24)**<br>• Gestation < 33 weeks<br><br>**Exposure**<br>• Mild WMI (n=4)<br>• Moderate WMI (n=5)<br>• Severe WMI (n=1)<br><br>• IVH grade 1 (n=5)<br>• IVH grade 2 (n=0)<br>• IVH grade 3 (n=0)<br>• IVH grade 4 (n=0)<br><br>**Comparator**<br>• Unmatched<br>• No WMI (n=14)<br>• No IVH (n=19)<br><br>**Ascertainment/ definition**<br>• MRI imaging reviewed by a blinded paediatric neuroradiologist<br>• Used own classification of white matter injury<br>• Papile classification	**Outcome**<br>• Cognitive<br>• Behaviour<br><br>**Assessment/ measurement**<br>• Test of variables of attention<br>• Conners comprehensive behaviour rating scales<br>• CBCL<br>• Assessment undertaken by a blinded psychologist<br>• Parental questionnaire<br><br>**Follow-up**<br>• 10-14 years<br>• Completeness not specified	**Attention (abnormal)**<br>Mild WMI n=3, 75%<br>Moderate WMI n=0, 0%<br>No WMI n=8, 57% p=0.05
24	Van de Bor 2004[15]<br><br>Netherlands<br><br>Prospective cohort	**Population**<br>• Gestation < 32 weeks<br>• Birthweight < 1500 g<br>• Born 1983<br><br>**Exposure**<br>• IVH grade 1-2 (n=45)<br>• IVH grade 3-4 (n=17)<br><br>**Comparator (n=216)**<br>• Unmatched<br>• No IVH<br><br>**Ascertainment/ definition**<br>• Ultrasound diagnosis<br>• Papile classification	**Outcomes**<br>• Disability (composite)<br>• Cognitive<br>• Neurological status (motor)<br>• Speech and language<br>• Behaviour<br>• Hearing<br>• Vision<br>•<br>**Measurement/assessment**<br>• Questionnaires (completed by parents at 9 years; adolescents at 14 years)<br>• Home visit and neurodevelopmental assessment by paediatrician unaware of medical history<br>• WHO classification of impairment, disability, and handicap<br><br>**Follow-up**<br>• 5, 9 and 14 years<br>91.5% follow-up of survivors at 14 years	**Disability at 5 years**<br>No IVH n=49 (23%)<br>IVH grade 3-4 n=5 (31.3%)<br><br>**Cognitive disability**<br>No IVH n=18 (8.3%)<br>IVH grade 3-4 n=1 (5.9%) p=not significant<br><br>**Motor disability**<br>No IVH n=8 (3.7%)<br>IVH grade 3-4 n=3 (17.6%) p=0.00<br><br>**Speech/language disability**<br>No IVH n=34 (15.7%)<br>IVH grade 3-4 n=1 (5.9%) p= not significant<br><br>**Visual disability**<br>No IVH n=1 (0.5%)<br>IVH grade 3-4 n=0 p= not significant<br><br>**Hearing disability**<br>No IVH n=5 (2.3%)<br>IVH grade 3-4 n=0 p= not significant<br><br><br>**School performance at 5 years**<br>**Special education**<br>No IVH n=17 (8.7%)<br>IVH grade 3-4 n=3 (20%) p=0.02<br><br>**School performance at 9 years**<br>**Slow learner**<br>No IVH n=57 (29.5%)<br>IVH grade 3-4 n=4 (26.7%)<br><br>**Special education**<br>No IVH n=29 (15%)<br>IVH grade 3-4 n=4 (26.7%) p=0.04<br><br>**School performance at 14 years**<br>**Slow learner**<br>No IVH n=93 (44.1)<br>IVH grade 3-4 n=4 (23.5%)<br><br>**Special education**<br>No IVH n=26 (12%)<br>IVH grade 3-4 n=6 (35.3%) p=0.00<br><br>**Need for special education at 14 years**<br>IVH (all grades)<br>OR 2.56 95%CI (1.17-4.86)<br>aOR 2.33 95%CI (1.15, 4.75)<br><br>IVH grade 3-4<br>aOR 3.99 95%CI (1.36, 11.69)
25	Van Den Hout 2000[26]<br><br>Netherlands<br><br>Prospective cohort	**Population**<br>• Mean gestation 28-30 weeks<br>• Born 1989-1991<br><br>**Exposure**<br>• IVH (n=17)<br>• PVL (n=12)<br><br>**Comparator (n=17)**<br>• Preterm<br>• Normal cranial ultrasound<br><br>**Ascertainment/ definition**<br>• Ultrasound diagnosis<br>• Modified Levene and DeVries classification for IVH<br>• DeVries classification for PVL	**Outcomes**<br>• Cognitive<br>• Visual acuity<br><br>**Measurement/ assessment**<br>• L94 visual-perceptual ability test<br>• Grating acuity cards<br>• McCarthy scales of children's abilities<br>• Wechsler preschool and primary scale of intelligence<br>• Snijders-Oomen non-verbal intelligence test<br>• Leiden Diagnostic test<br><br>**Follow-up**<br>• Mean 5.3 years<br>• 88% follow-up	**Total intelligence quotient, mean (SD)**<br>IVH 92.4 (16.3)<br>PVL 79.6 (20.5)<br>No brain injury 102.8 (14.4)<br><br>**IQ <85**<br>IVH n=6, 35.3%<br>PVL n=6, 50%<br>No brain injury n=2, 11.8%<br><br>**Performance age in years, mean (SD)**<br>IVH 5.22 (1.16)<br>PVL 4.37 (1.19)<br>No brain injury 6.22 (0.89)<br><br>**Visual grating acuity in c/deg, mean (SD)**<br>IVH 37.4 (13.5)<br>PVL 33.5 (15.9)

Confidential: For Review Only

				No brain injury 47.1 (13.5)<br><br>**Visual grating acuity <25c/deg (%)**<br>IVH (11.8)<br>PVL (33.3)<br>No brain injury (0)<br><br>**Impairment on each of the eight L94 tasks**<br>**Visual matching % (n)**<br>IVH 0 (17)<br>PVL 0 (12)<br>No brain injury 5.9 (17)<br><br>**Unconventional Object Views % (n)**<br>IVH 29.4 (17)<br>PVL 41.7 (12)<br>No brain injury 17.6 (17)<br><br>**De Vos task % (n)**<br>IVH 29.4 (17)<br>PVL 41.7 (12)<br>No brain injury 11.8 (17)<br><br>**Line Drawings Occluded by Noise% (n)**<br>IVH 6.3 (16)<br>PVL 36.4 (11)<br>No brain injury 0 (17)<br><br>**Line Drawings Occluded by Noise% (n)**<br>IVH 13.3 (15)<br>PVL 25.0 (8)<br>No brain injury 5.9 (17)<br><br>**Developmental test of visual motor integration % (n)**<br>IVH 0 (16)<br>PVL 0 (7)<br>No brain injury 0 (17)<br><br>**Matching block designs % (n)**<br>IVH 5.9 (17)<br>PVL 20.0 (10)<br>No brain injury 17.6 (17)<br><br>**Constructing block designs% (n)**<br>IVH 30.8 (13)<br>PVL 80.0 (5)<br>No brain injury 31.3 (16)<br><br>**Mean percentage of L94 tasks on which child is impaired (mean, SD; %)**<br>IVH 14.71 (17.81)<br>PVL 32.04 (24.64)<br>No brain injury 11.13 (9.79)
26<br>*	Vollmer 2003[16]<br><br>UK<br><br>Prospective cohort	**Population**<br>• Gestation <33 weeks<br>• Born 1983-1988<br><br>**Exposure**<br>• IVH (n=159)<br>• Ventricular dilatation (n=32)<br>• IVH, PV flare, ventricular dilatation (n=164)<br>• Hydrocephalus (n=36)<br>• Haemorrhagic parenchymal infarction (HPI) (n=61)<br>• cPVL n=26<br><br>**Comparator (n=348)**<br>• Unmatched<br>• Normal scan<br><br>**Ascertainment/ definition**<br>• Ultrasound imaging reviewed by two experienced observers<br>• In-house classification used	**Outcomes**<br>• Neurodevelopmental impairment (composite)<br>• Visual impairment<br>• Hearing impairment<br><br>**Measurement/ assessment**<br>• Structured neurologic examination<br>• Pure-tone audiogram<br>• Vision test (Snellen chart)<br>• Henderson-Stott TOMI<br>• Beery test of VMI<br>• WISC-R for children born 1983-1986<br>• WISC-III for children born 1987-1988<br><br>**Follow-up**<br>• 8 years<br>• 91.7% follow-up	**Neurodevelopmental status**<br>**Group A (<28 weeks)**<br>**All impairments (n,%)**<br>GMH/IVH (5, 18%)<br>Ventricular dilatation (4, 50%)<br>GMH/IVH, flare, ventricular dilatation (19, 51%)<br>Hydrocephalus (7, 78%)<br>HPI (15, 100%)<br>cPVL (4, 100%)<br>No brain injury (12, 32%)<br><br>**Disabling impairments (n, %)**<br>GMH/IVH (1, 4%)<br>Ventricular dilatation (0, 0%)<br>GMH/IVH, flare, ventricular dilatation (9, 24%)<br>Hydrocephalus (7, 78%)<br>HPI (14, 93%)<br>cPVL (3, 75%)<br>No brain injury (3, 8%)<br><br>**Group B (28-32 weeks)**<br>**All impairments (n, %)**<br>GMH/IVH (16, 29%)<br>Ventricular dilatation (5, 31%)<br>GMH/IVH, flare, ventricular dilatation (30, 43%)<br>Hydrocephalus (7, 54%)<br>HPI (5, 83%)<br>cPVL (9, 75%)<br>No brain injury (67, 29%)<br><br>**Disabling impairments (n, %)**<br>GMH/IVH (5, 5%)<br>Ventricular dilatation (1, 6%)<br>GMH/IVH, flare, ventricular dilatation (16, 23%)<br>Hydrocephalus (6, 46%)<br>HPI (3, 50%)<br>cPVL (6, 50%)<br>No brain injury (14, 6%)
27<br>*	Vollmer 2006a[21]<br><br>UK<br><br>Prospective cohort	**Population**<br>• Gestation <33 weeks<br>• Born 1985-1991<br><br>**Exposure**<br>• Bilateral brain lesions (n=201)<br>• Right-sided brain lesion (n=41)	**Outcomes**<br>• Motor<br>• Cognitive<br>• Cerebral palsy<br>• Visual	**TOMI error score, mean (SD)**<br>Normal scan 2.78 (2.1)<br><br>All left-sided lesions 4.3 (3.5)<br>Left-sided non-parenchymal lesions 4.5 (3.8)<br>Left-sided parenchymal lesions 3.7 (2.1)

Confidential: For Review Only

		• Left-sided brain lesion (n=57)<br><br>Brain lesion types<br>Non-parenchymal:<br>• Uncomplicated IVH<br>Parenchymal:<br>• Haemorrhagic parenchymal infarction (HPI)<br>• cPVL<br>• PV flare<br><br>**Comparator (n=369)**<br>• Unmatched<br>• Normal ultrasound<br><br>**Ascertainment/ definition**<br>• Ultrasound imaging reviewed by two experienced observers<br>• Modified Stewart classification	**Measurement/ assessment**<br>• Neurological examination (modified Amiel-Tison assessment)<br>• TOMI<br>• WISC-R<br>• Test of VMI<br><br>**Follow-up**<br>• 8 years<br>• 80% follow-up	All right-sided lesions 3.5 (2.9)<br>Right-sided non-parenchymal lesions 2.7 (1.8)<br>Right-sided parenchymal lesions 4.9 (3.8)<br><br>All bilateral lesions 4.5 (4.3)<br>Bilateral non-parenchymal lesions 4.1 (3.7)<br>Bilateral parenchymal lesions 4.9 (4.7)<br><br>ANOVA for parenchymal lesions only p <0.0001<br>ANOVA including parenchymal and non-parenchymal lesions p <0.0001<br>ANOVA excluding parenchymal lesions, p <0.0001<br><br>**VMI centile, mean (SD)**<br>Normal scan 59.2 (30.0)<br><br>All left-sided lesions 40.3 (30.1)<br>Left-sided non-parenchymal lesions 46.8 (31.0)<br>Left-sided parenchymal lesions 21 (22)<br><br>All right-sided lesions 60.2 (31.9)<br>Right-sided non-parenchymal lesions 64.2 (30.2)<br>Right-sided parenchymal lesions 54 (35)<br><br>All bilateral lesions 46.0 (33.5)<br>Bilateral non-parenchymal lesions 55.1 (32.1)<br>Bilateral parenchymal lesions 38 (32)<br><br>ANOVA for parenchymal lesions only p <0.0001<br>ANOVA including parenchymal and non-parenchymal lesions p <0.0001<br>ANOVA excluding parenchymal lesions reported as both p <0.0001 and p=0.98 Ω(potential error in the manuscript table)<br><br>**Cerebral palsy, n (%)**<br>Normal scan 2 (0.7%)<br><br>All left-sided lesions 4 (9%)<br>Left-sided non-parenchymal lesions 2 (6%)<br>Left-sided parenchymal lesions 2 (16%)<br><br>All right-sided lesions 2 (6%)<br>Right-sided non-parenchymal lesions 1 (4%)<br>Right-sided parenchymal lesions 1 (8%)<br><br>All bilateral lesions 37 (21%)<br>Bilateral non-parenchymal lesions 8 (10%)<br>Bilateral parenchymal lesions 29 (31%)<br><br>Chi-square for parenchymal and non-parenchymal lesions, p <0.0001<br>Chi-square excluding parenchymal lesions, p <0.0001<br>Chi-square for parenchymal lesions only, p <0.0001<br>ANOVA parenchymal lesions only, p <0.0001<br><br>**Full scale IQ, mean (SD)**<br><br>Normal scan 101 (16)<br><br>All left-sided lesions 93 (17)<br>Left-sided non-parenchymal lesions 98 (15)<br>Left-sided parenchymal lesions 80 (15)<br><br>All right-sided lesions 102 (17)<br>Right-sided non-parenchymal lesions 104 (15)<br>Right-sided parenchymal lesions 100 (19)<br><br>All bilateral lesions 91 (21)<br>Bilateral non-parenchymal lesions 96(19)<br>Bilateral parenchymal lesions 86 (22)<br><br>ANOVA for parenchymal lesions only, p <0.0001.<br>ANOVA including parenchymal and non-parenchymal lesions, p <0.0001.<br>ANOVA excluding parenchymal lesions, p =0.137.<br><br>**Verbal IQ, mean (SD)**<br>Normal scan 103 (19)<br><br>All left-sided lesions 98 (20)<br>Left-sided non-parenchymal lesions 102 (20)<br>Left-sided parenchymal lesions 85 (18)<br><br>All right-sided lesions 107 (18)<br>Right-sided non-parenchymal lesions 108 (16)<br>Right-sided parenchymal lesions 107 (22)<br><br>All bilateral lesions 96 (23)<br>Bilateral non-parenchymal lesions 100 (20)<br>Bilateral parenchymal lesions 91 (25)<br><br>ANOVA for parenchymal lesions only, p <0.0001<br>ANOVA including parenchymal and non-parenchymal lesions, p <0.0001<br>ANOVA excluding parenchymal lesions, p =0.38<br><br>**Performance IQ, mean (SD)**<br>Normal scan 96 (15)<br><br>All left-sided lesions 86 (16)<br>Left-sided non-parenchymal lesions 90 (15)<br>Left-sided parenchymal lesions 76 (15)

Confidential: For Review Only

				All right-sided lesions 95 (16)<br>Right-sided non-parenchymal lesions 98 (13)<br>Right-sided parenchymal lesions 92 (19)<br><br>All bilateral lesions 85 (22)<br>Bilateral non-parenchymal lesions 91 (20)<br>Bilateral parenchymal lesions 80 (21)<br><br>ANOVA for parenchymal lesions only, p <0.0001<br>ANOVA including parenchymal and non-parenchymal lesions, p <0.0001<br>ANOVA excluding parenchymal lesions, p =0.59
28<br>*	Vollmer 2006b[27]<br><br>UK<br><br>Prospective cohort	**Population**<br>• Gestation <33 weeks<br>• Born 1979-1991<br><br>**Exposure (n=66)**<br>• Ventricular dilatation and IVH<br><br>**Comparator (n=616)**<br>• Unmatched<br>• Normal cranial ultrasound<br><br>**Ascertainment/ definition**<br>• Ultrasound imaging reviewed by two experienced observers<br>• In-house classification used	**Outcomes**<br>• Neurological impairment with or without disability (composite)<br>• Cognitive<br>• Motor<br>• Vision<br><br>**Measurement/ assessment**<br>• Structured neurological exam<br>• TOMI<br>• Test of VMI<br>• WISC<br><br>**Follow-up**<br>• 8 years<br>• 81% follow-up	**<u>Disabling motor impairment, n (%)</u>**<br>Ventricular dilatation and IVH n=10 (16%)<br>Normal ultrasound n=10 (2%)<br><br>**<u>Cognitive</u>**<br>**Full scale IQ, mean (SD)**<br>Ventricular dilatation and IVH 96 (23)<br>Normal ultrasound 101 (17)<br><br>**Verbal IQ, mean (SD)**<br>Ventricular dilatation and IVH 101 (22)<br>Normal ultrasound 104 (19)<br><br>**Performance IQ mean (SD)**<br>Ventricular dilatation and IVH 97 (15)<br>Normal ultrasound 91 (21)<br><br>**<u>Motor and vision</u>**<br>**VMI centile, mean (SD)**<br>Ventricular dilatation and IVH 37 (33)<br>Normal ultrasound 52 (31)<br><br>**TOMI, mean (SD)**<br>Ventricular dilatation and IVH 5.98 (4.2)<br>Normal ultrasound 3.26 (2.5)
29	Whitaker 2011[30]<br><br>USA<br><br>Prospective cohort	**Population**<br>• Birthweight <2000g<br>• 'Non-disabled' survivors<br>• Born 1984-1987<br><br>**Exposure**<br>• IVH (n=69)<br>• Parenchymal lesions and/or ventricular enlargement (n=21)<br><br>**Comparison (n=368)**<br>• Unmatched<br>• Normal cranial ultrasound<br><br>**Ascertainment/ definition**<br>• Ultrasound imaging reviewed by three blinded radiologists independently, disagreements resolved through consensus and inter-observer reliability checked.<br>• Paneth classification	**Outcomes**<br>• Mental health conditions<br><br>**Measurement/ assessment**<br>• Parent report version of the Diagnostic Interview Schedule for Children–IV<br>• WASI<br><br>**Follow-up**<br>• 16 years<br>• 72.9% follow-up	**<u>Logistic regression assessing odds of current and lifetime mental health conditions after brain injury</u>**<br><br>**<u>Current ADHD- inattentive type</u>**<br>**IVH**<br>OR 0.97 95% CI (0.21-4.47)<br>aOR 1.01 95% CI (0.19-5.44)<br><br>**Parenchymal lesions and/or ventricular enlargement**<br>OR 7.64[b] 95% CI (2.20-24.48)<br>aOR 6.83[c] 95% CI (1.26-36.91)<br><br>**<u>Lifetime ADHD – inattentive type</u>**<br>**IVH**<br>OR 0.83 95% CI (0.34-2.04)<br>aOR 0.64 95% CI (0.24-1.74)<br><br>**Parenchymal lesions and/or ventricular enlargement**<br>OR 2.71 95% CI (0.94-7.82)<br>aOR 1.13 95% CI (0.31-4.10)<br><br>**<u>Current major depression</u>**<br>**IVH**<br>OR 2.66 95% CI (1.04-6.78)<br>aOR 2.23 95% CI (0.80-6.24)<br><br>**<u>Lifetime major depression</u>**<br>**IVH**<br>OR 2.76 95% CI (1.19-6.38)<br>aOR 2.59 95% CI (1.02-6.58)<br><br>**<u>Current tic disorders</u>**<br>**IVH**<br>OR 1.63 95% CI (0.44-6.07)<br>aOR 1.89 95% CI (0.42-8.57)<br><br>**Parenchymal lesions and/or ventricular enlargement**<br>OR 8.42 95% CI (2.40-29.62)<br>aOR 9.77 95% CI (1.69-56.47)<br><br>**<u>Lifetime tic disorders</u>**<br>**IVH**<br>OR 0.95 95% CI (0.27-3.34)<br>aOR 0.85 95% CI (0.21-3.51)<br><br>**Parenchymal lesions and/or ventricular enlargement**<br>OR 5.07 95% CI (1.53-16.82)<br>aOR 5.02 95% CI (1.05-23.92)<br><br>**<u>Current obsessive-compulsive disorder</u>**<br>**IVH**<br>OR 9.52 95% CI (3.02-30.06)<br>aOR 11.85 95% CI (3.22-43.62)<br><br>**Parenchymal lesions and/or ventricular enlargement**<br>OR 7.64 95% CI (1.39-41.98)<br>aOR 15.32 95% CI (1.82-128.74)<br><br>**<u>Lifetime obsessive compulsive disorder</u>**<br>**IVH**<br>OR 9.52 95% CI (3.05-30.06)<br>aOR 11.85 95% CI (3.22-43.62)

Confidential: For Review Only

				**Parenchymal lesions and/or ventricular enlargement**<br>OR 7.64 95% CI (1.39-41.98)<br>aOR 15.32 95% CI (1.82-128.74)<br><br>**Current diagnoses additionally controlled for full score IQ and motor function**<br><br>**ADHD inattentive type**<br>**IVH**<br>OR 0.86 95% CI (0.18-3.99)<br>aOR 0.99 95% CI (0.21-4.62)<br><br>**Parenchymal lesions and/or ventricular enlargement**<br>OR 5.04 95% CI (1.36-18.65)<br>aOR 5.43 95% CI (1.32-22.40)<br><br>**Major depression**<br>**IVH**<br>OR 0.43 95% CI (0.16-1.11)<br>aOR 0.40 95% CI (0.15-1.05)<br><br>**Tic disorders**<br>**IVH**<br>OR 1.54 95% CI (0.41-5.78)<br>aOR 1.45 95% CI (0.38-5.48)<br><br>**Parenchymal lesions and/or ventricular enlargement**<br>OR 7.01 95% CI (1.88-28.14)<br>aOR 4.38 95% CI (1.05-18.23)<br><br>**Obsessive compulsive disorder**<br>**IVH**<br>OR 8.68 95% CI (2.72-27.69)<br>aOR 10.91 95% CI (3.13-37.99)<br><br>**Parenchymal lesions and/or ventricular enlargement**<br>OR 4.78 95% CI (0.83-28.10)<br>aOR 3.58 95% CI (0.50-25.94)
Perinatal stroke				
30	Ballantyne * 2007 [41]<br>USA<br><br>Prospective cohort	**Population**<br>• Mean gestation 38.5 weeks<br>• Born 1991-2001<br><br>**Exposure (n=28)**<br>• Left lesions (n=17)<br>• Right lesions (n=11)<br><br>**Comparator (n=57)**<br>• Unmatched<br>• Healthy controls with normal medical and developmental histories<br>• Recruited from the community<br><br>**Ascertainment/ definition**<br>• Single unilateral lesions the result of perinatal strokes occurring between 28 weeks' gestation and 28 days after birth; infarct or haemorrhage<br>• Identified through medical history and neuroimaging<br>• Severity rated on a 5-point scale adapted from the Vargha-Khadem classification	**Outcomes**<br>• Speech and language<br><br>**Assessment/ measurement**<br>• CELF-R<br>• Wechsler Intelligence Scales (WPPSI-R, WISC-R, or WISC-III)<br>• PPVT–Revised<br>• Expressive One-Word Picture Vocabulary Test–Revised or Upper-Extension<br>• Total Language Standard Scores<br><br>**Follow-up**<br>• 6-9 years<br>• 100% follow-up	**Speech and language**<br>**CELF-R Receptive, mean (SD)**<br>All strokes: 82.54 (17.12) p<.0001<br>Left stroke: 83.18 (16.66) p<.0001<br>Right stroke: 81.55 (18.59) p=0.001<br>Control: 106.37 (12.51)<br><br>**CELF-R Expressive mean (SD)**<br>All strokes: 73.75 (16.79) p<.0001<br>Left stroke: 73.06 (14.88) p<.0001<br>Right stroke: 74.82 (20.11) p=0.001<br>Control: 101.02 (13.63)<br><br>**CELF-R Total mean (SD)**<br>All strokes: 76.93 (17.31) p<.0001<br>Left stroke: 76.94 (15.39) p<.0001<br>Right stroke: 76.91 (20.74) p=0.001<br>Control: 104.00 (12.58)<br>.
31	Ballantyne 2008[34] *<br><br>USA<br><br>Prospective cohort	**Population**<br>• 32- 40 weeks' gestation<br>• Birth years not reported<br><br>**Exposure (n=29)**<br>• Left hemisphere (n=20)<br>• Right hemisphere (n=9)<br><br>**Control (n=38)**<br>• Healthy controls (normal neurodevelopment)<br>• Recruited through a university and community adverts<br><br>**Ascertainment/ definition**<br>• Unilateral ischaemic perinatal stroke confirmed through clinical history and neuroimaging<br>• Lesion location and severity reviewed by blinded neuroradiologist<br>• Severity rated on a 5-point scale adapted from the Vargha-Khadem classification	**Outcomes**<br>• Cognitive (academic skills)<br>• Speech and language<br>• Motor<br>• Cerebral palsy<br>• Vision<br>• Epilepsy<br><br>**Measurement/ assessment**<br>• WISC- Revised<br>• WRAT- Revised<br>• CELF- Revised<br>• PPVT-Revised<br>• WPPSI/WPPSI- Revised<br>• WISC-III<br><br>**Follow-up**<br>• 7-12 years<br>• 100% follow up	**Hemiparesis**<br>Stroke n=18,62%<br><br>**Visual field deficit**<br>Stroke n=7, 26%<br><br>**Seizures**<br>Stroke n=11, 38%<br><br>**Cognitive, mean (SD)**<br>**Verbal IQ (WISC-R)**<br>**Time point 1 (mean age 7-8 years)**<br>Stroke 96.6 (20.5)<br>Control 126.1 (16)<br><br>**Time point 2 (mean age 10 – 12 years)**<br>Stroke 98.7 (20)<br>Control 123.6 (13.1)<br>Between group affect (stroke vs. control) p<0.0001<br>Time effect not significant<br><br>**Performance IQ (WISC-R)**<br>**Time point 1 (mean age 7-8 years)**<br>Stroke 92.8 (19.9)<br>Control 115.2 (13.8)<br><br>**Time point 2 (mean age 10 – 12 years)**<br>Stroke 93.5 (20)<br>Control 116 (10.5)<br>Between group affect (stroke vs. control) p=0.002<br>Time effect not significant<br><br>**Full scale IQ (WISC-R)**

Confidential: For Review Only

	USA			**Time point 1 (mean age 7-8 years)**<br>Stroke 94.7 (20.4)<br>Control 123 (15)<br><br>**Time point 2 (mean age 10 – 12 years)**<br>Stroke 96.1 (19.1)<br>Control 122.3 (10.2)<br><br>Between group affect (stroke vs. control) p<0.0001<br>Time effect not significant<br><br>**<u>Reading (WRAT -R)</u>**<br>**Time point 1 (mean age 7-8 years)**<br>Stroke 85 (16.1)<br>Control 113 (13.3)<br><br>**Time point 2 (mean age 10 – 12 years)**<br>Stroke 89.4 (13.3)<br>Control 108.9 (13.8)<br><br>Between group affect (stroke vs. control) p<0.0001<br>Time effect not significant<br>Time group interaction p=0.045<br><br>**<u>Spelling (WRAT -R)</u>**<br>**Time point 1 (mean age 7-8 years)**<br>Stroke 82.5 (18.2)<br>Control 106.2 (15.9)<br><br>**Time point 2 (mean age 10 – 12 years)**<br>Stroke 87 (16.8)<br>Control 104.6 (13.1)<br><br>Between group affect (stroke vs. control) p=0.001<br>Time effect not significant<br><br>**<u>Arithmetic (WRAT -R)</u>**<br>**Time point 1 (mean age 7-8 years)**<br>Stroke 91.5 (10.2)<br>Control 111.9 (11.2)<br><br>**Time point 2 (mean age 10 – 12 years)**<br>Stroke 94.2 (18.7)<br>Control 113.1 (16.2)<br><br>Between group affect (stroke vs. control) p<0.0001<br>Time effect not significant<br><br>**<u>Speech and language</u>**<br>**Receptive language score**<br>**Time point 1 (mean age 7-8 years)**<br>Stroke 84.2 (10.9)<br>Control 109.1 (12.2)<br><br>**Time point 2 (mean age 10 – 12 years)**<br>Stroke 82.3 (20.1)<br>Control 111.4 (13.7)<br><br>Between group affect (stroke vs. control) p<0.0001<br>Time effect not significant<br><br>**Expressive language score**<br>**Time point 1 (mean age 7-8 years)**<br>Stroke 72.5 (12)<br>Control 101 (17.5)<br><br>**Time point 2 (mean age 10 – 12 years)**<br>Stroke 78.4 (16)<br>Control 105.8 (11.9)<br><br>Between group affect (stroke vs. control) p<0.0001<br>Time effect p=0.017<br><br>**Total language score**<br>**Time point 1 (mean age 7-8 years)**<br>Stroke 76.9 (11.1)<br>Control 105.6 (14.2)<br><br>**Time point 2 (mean age 10 – 12 years)**<br>Stroke 79.1 (18.3)<br>Control 109.8 (14)<br><br>Between group affect (stroke vs. control) p<0.0001<br>Time effect not significant<br><br>**Vocabulary score**<br>**Time point 1 (mean age 7-8 years)**<br>Stroke 97.5 (19.7)<br>Control 117.1 (17)<br><br>**Time point 2 (mean age 10 – 12 years)**<br>Stroke 99.9 (20)<br>Control 118.9 (13.9)<br><br>Between group affect (stroke vs. control) p=0.002<br>Time effect not significant
32	Gold 2014[35]<br><br>USA	**Population**<br>• Gestation not provided<br>• Birth years not provided	**Outcomes**<br>• Cognitive (IQ and memory)<br>• Motor<br>• Cerebral palsy	**<u>Cognitive</u>**<br>**<u>Memory</u>**<br>**Stories immediate recall**<br>Controls, mean (SE)13.5 (0.7)

Confidential: For Review Only

	Prospective cohort	**Exposure (n=27)**<br>• Right-sided stroke (n=12)<br>• Left-sided stroke (n=15)<br><br>**Comparator (n=19)**<br>• Matched for age at follow up, sex, socioeconomic group and maternal education<br>• Healthy controls<br>• Recruited through local advertising<br><br>**Ascertainment/ definition**<br>• Single, unilateral brain lesion in an arterial vascular distribution, either identified in the neonatal period with neuroimaging, or identified later in infancy after presentation with a hemiparesis and imaging documentation of an old unilateral infarct (presumed perinatal stroke)<br>• Recruited from paediatric neurology clinics<br>• Severity graded 1-5 using Trauner/ Vargha-Khaldem classification	**Measurement/ assessment**<br>• WISC-III<br>• Dots and Stories subtests of the Children's Memory Scales<br><br>**Follow-up**<br>• 6-16 years<br>• 100% follow-up	Stroke, mean (SE) 8.4 (0.8) p<0.001<br><br>Stroke and seizures, mean (SE)7 (0.8)<br>Stroke and no seizures, mean (SE) 10.1 (1.4) p=0.06<br><br>Right lesion, mean (SE) 7.8 (1.1)<br>Left lesion, mean (SE) 8.9 (1.2) p=0.51<br><br>**Delayed recall**<br>Controls, mean (SE) 13.9 (0.8)<br>Stroke, mean (SE) 7.9 (0.8) p<0.001<br><br>Stroke and seizures, mean (SE) 6.2 (0.9)<br>Stroke and no seizures, mean (SE) 10 (1.2) p=0.02<br><br>Right lesion, mean (SE) 7.3 (1.1)<br>Left lesion, mean (SE) 8.3 (1.2) p=0.56<br><br>**Delayed recognition**<br>Controls, mean (SE) 11.5 (0.5)<br>Stroke, mean (SE) 8 (0.8) p=0.001<br><br>Stroke and seizures, mean (SE) 7.1 (1.1)<br>Stroke and no seizures, mean (SE) 9.2 (0.9) p=0.17<br><br>Right lesion, mean (SE) 8.3 (1.4)<br>Left lesion, mean (SE) 7.9 (0.9) p=0.8<br><br>**Dots learning**<br>Controls, mean (SE) 10.9 (0.5)<br>Stroke, mean (SE) 8.9 (0.8) p=0.05<br><br>Stroke and seizures, mean (SE) 7.6 (1.1)<br>Stroke and no seizures, mean (SE) 10.6 (0.8) p=0.05<br><br>Right lesion, mean (SE) 9.3 (1.4)<br>Left lesion, mean (SE) 8.7 (0.9) p=0.71<br><br>**Total**<br>Controls, mean (SE) 11.8 (0.5)<br>Stroke, mean (SE) 9 (0.7) p=0.003<br><br>Stroke and seizures, mean (SE) 7.8 (0.9)<br>Stroke and no seizures, mean (SE) 10.6 (0.9) p=0.04<br><br>Right lesion, mean (SE) 9.2 (0.7)<br>Left lesion, mean (SE) 10.2 (0.7) p=0.62<br><br>**Delayed recall**<br>Controls, mean (SE) 12.6 (0.4)<br>Stroke, mean (SE) 10 (0.5) p<0.001<br><br>Stroke and seizures, mean (SE) 8.8 (0.5)<br>Stroke and no seizures, mean (SE) 11.4 (0.8) p=0.009<br><br>Right lesion, mean (SE) 9.7 (0.7)<br>Left lesion, mean (SE) 10.2 (0.7) p=0.62<br><br>**WISC- III IQ, mean (SD)**<br>Right stroke, 85.0 (6)<br>Left stroke, 91 (6) p=0.49<br><br>**IQ scores**<br>Controls 117 (2.7)<br>All stroke patients 88 (4.0) p<0.001<br>No seizures 100 (6.4)<br>Seizures 78 (3.7)<br><br>**<u>Motor (hemiparesis)</u>**<br>Stroke patients n=16; 59%<br>Control n=0; p=0.05
33	Kolk 2011[36]<br><br>Estonia<br><br>Retrospective cohort	**Population**<br>• Gestation not provided<br>• Born 1995-2006<br><br>**Exposed (n=21)**<br>• Neonatal stroke<br><br>**Control (n=31)**<br>• Matched on age and sex<br>• Healthy children<br>• Recruited locally<br><br>**Ascertainment/ definition**<br>• Estonian stroke registry<br>• Arterial ischaemic stroke or haemorrhagic	**Outcomes**<br>• Cognitive<br>• Neuropsychological<br>• Motor<br>• Cerebral palsy<br>• Speech and language<br>• Epilepsy<br><br>**Measurement/ assessment**<br>• NEPSY<br>• Kaufman ABC<br>• Paediatric Stroke Outcome Measure<br><br>**Follow-up**<br>• 4-10 years<br>• 100% follow-up	**<u>Neuromotor impairment (Paediatric Stroke Outcome Measure)</u>**<br>**Neonatal stroke**<br>Severe n=4, 19%<br>Moderate n=9, 43%<br>Good n=6, 28.6%<br>Normal n=2, 9.5%<br><br>**<u>Cognitive/ neuropsychological</u>**<br><br>**<u>Attention and executive function, mean, SD, 95% CI</u>**<br>**Tower**<br>Control 0.22, 0.64 (-0.05, 0.48)<br>Neonatal stroke -0.34, 1.34 (-1.03, 0.35) p=0.142<br><br>**Auditory attention**<br>Control 0.27, 0.72 (-0.03, 0.57)<br>Neonatal stroke -0.38, 1.10 (-1.04, 0.28) p=0.009<br><br>**Visual attention: time**<br>Control 0.37, 0.81, (0.07, 0.67)<br>Neonatal stroke -0.40, 0.93 (-0.82, 0.03) p=0.004<br><br>**Visual attention: correct**<br>Control 0.48, 0.50 (0.30, 0.67)<br>Neonatal stroke -0.54, 0.97 (0.98, 0.1) p<0.0001<br><br>**Statue**

Confidential: For Review Only

				Control 0.26, 0.77 (-0.03, 0.54)<br>Neonatal stroke -0.23, 1.09, (-0.73, 0.28) p=0.086<br><br>**Design fluency**<br>Control 0.18, 1.04 (-0.25, 0.61)<br>Neonatal stroke -0.36, 0.70 (-0.78, 0.06) p=0.06<br><br>**Knock and tap**<br>Control 0.31, 0.50 (0.10, 0.51)<br>Neonatal stroke -0.44, 1.52, (-1.32, 0.43) p==0.03<br><br>**<u>Language, mean, SD, 95% CI</u>**<br>**Phonological processing**<br>Control 0.24, 0.80 (-0.05, 0.54)<br>Neonatal stroke -0.38, 0.99 (-0.83, 0.08) p=0.001<br><br>**Comprehension of instructions**<br>Control 0.43, 0.70 (0.18, 0.69)<br>Neonatal stroke -0.59 1.06 (-1.07, 0.11) p<0.0001<br><br>**Speeded naming: time**<br>Control 0.24, 0.70 (-0.05, 0.52)<br>Neonatal stroke -0.14, 1.03 (-0.73, 0.46) p=0.188<br><br>**Speeded naming: correct**<br>Control 0.42, 0.41 (0.25, 0.59)<br>Neonatal stroke -0.45, 1.41 (-1.26, 0.37) p=0.008<br><br>**Repetition of nonsense words**<br>Control 0.30, 0.53 (0.08, 0.52)<br>Neonatal stroke -0.40, 1.23 (-1.03, 0.24) p=0.026<br><br>**Verbal fluency: semantic**<br>Control 0.43, 0.81 (0.13, 0.73)<br>Neonatal stroke -0.60, 0.95 (-1.04, 0.15) p<0.0001<br><br>**Verbal fluency: phonemic**<br>Control 0.40, 0.93 (-0.12, 0.92)<br>Neonatal stroke -0.67, 0.90 (-1.42, 0.08) p=0.008<br><br>**Oromotor sequences**<br>Control 0.31, 0.64 (0.07, 0.54)<br>Neonatal stroke -0.52, 1.25 (-1.15, 0.10)<br><br>**Sentence comprehension**<br>Control 0.19, 0.78 (-0.09, 0.48)<br>Neonatal stroke -0.35, 1.09 (-0.91, 0.21) p=0.027<br><br>**<u>Sensorimotor functions, mean, SD, 95% CI</u>**<br>**Finger tapping**<br>Control 0.49, 0.33 (0.35, 0.62)<br>Neonatal stroke -0.53, 1.27 (-1.16, 0.10) p=0.0007<br><br>**Imitating hand positions**<br>Control 0.57, 0.68 (0.32-0.82)<br>Neonatal stroke -0.72, 0.92 (-1.14, 0.30) p<0.0001<br><br>**Visuomotor precision: time**<br>Control 0.13, 0.83 (-0.17, 0.43)<br>Neonatal stroke -0.24, 0.97 (-0.69, 0.20) p=0.145<br><br>**Visuomotor precision: mistakes**<br>Control 0.45, 0.50 (0.27, 0.64)<br>Neonatal stroke -0.42, 1.05 (-0.90, 0.05) p=0.0002<br><br>**Manual motor sequences**<br>Control 0.50, 0.62 (0.27, 0.73)<br>Neonatal stroke -0.92, 0.95 (-1.43, 0.41) p<0.0001<br><br>**Finger discrimination**<br>Control 0.53, 0.57 (0.29, 0.77)<br>Neonatal stroke -0.77, 1.03 (-1.30, 0.24) p<0.0001<br><br>**<u>Visuospatial functions, mean, SD, 95% CI</u>**<br>**Design copying**<br>Control 0.36, 0.80 (0.06, 0.65)<br>Neonatal stroke -0.54, 0.97 (-1.0, 0.09) p<0.0001<br><br>**Arrows**<br>Control 0.37, 0.79 (0.05, 0.70)<br>Neonatal stroke -0.61, 1.07 (-1.16, 0.06) p=0.0004<br><br>**Block construction**<br>Control 0.29, 0.81 (-0.01, 0.58)<br>Neonatal stroke -0.45, 1.04 (-0.92, 0.03) p=0.0003<br><br>**Route finding**<br>Control 0.25, 1.05 (-0.33, 0.83)<br>Neonatal stroke -0.66, 0.80 (-1.23, 0.09) p=0.033<br><br>**Picture perception**<br>Control 0.13, 1.00 (-0.49, 0.24)<br>Neonatal stroke -0.09, 1.03 (-0.56, 0.37) p=0.341<br><br>**<u>Memory and learning, mean, SD, 95% CI</u>**<br>**Memory for faces**<br>Control 0.42, 0.74 (0.11, 0.73)<br>Neonatal stroke -0.41, 1.15 (-0.96, 0.15) p=0.016

Confidential: For Review Only

				**Memory for names**<br>Control 0.15, 0.92 (-0.23. 0.53)<br>Neonatal stroke -0.31, 1.09 (-0.87, 0.25) p=0.295<br><br>**Narrative memory**<br>Control 0.26, 0.80 (-0.03, 0.55)<br>Neonatal stroke -0.22, 1.16 (-0.78, 0.34) p=0.077<br><br>**Sentence repetition**<br>Control 0.49, 0.61 (0.26, 0.71)<br>Neonatal stroke -0.64, 0.96 (-1.09, 0.19) p<0.0001<br><br>**List learning**<br>Control 0.30, 0.82 (-0.16, 0.76)<br>Neonatal stroke -0.38, 1.22 (-1.32, 0.56) p=0.151<br><br>**Picture recognition**<br>Control 0.39, 0.72 (0.10, 0.69)<br>Neonatal stroke -0.36, 1.24 (-0.98, 0.25) p=0.027<br><br>**Motor (hemiparesis)**<br>Neonatal stroke and any hemiparesis n=19, 90%<br>Mild functional impairment n=6, 29%<br>Significant functional impairment n= 8, 38%<br>Very severe functional impairment n= 4, 19%<br><br>**Epilepsy**<br>Stroke n=9, 33.3%
34	Martin 2019[40] *<br><br>USA<br><br>Prospective cohort	**Population**<br>• Gestation not provided<br>• Birth years not provided<br><br>**Exposure (n=21)**<br>• Left hemisphere (n=13)<br>• Right hemisphere (n=8)<br><br>**Control (n=21)**<br>• Matched on age, sex and socioeconomic status<br>• Healthy controls<br>• Recruited from local community using adverts<br><br>**Ascertainment/ definition**<br>• Unilateral focal brain lesion (ischaemic or haemorrhagic thought to have occurred between 28 weeks' gestation and 28 days postnatally)<br>• Recruited from a neurologist in San Diego	**Outcomes**<br>• Hearing<br>• Motor (cerebral palsy)<br>• Epilepsy<br><br>**Measurement/ assessment**<br>• Auditory neglect task<br><br>**Follow-up**<br>• 6-14 years (mean 9-10 years)<br>• Completeness not specified	**Time to correct response**<br>**Left sided sound:**<br>Left stroke 1550 ms±580 ms<br>Control 1465 ms±666 ms *not significant*<br><br>Right stroke 1708 ms±951 ms<br>Control 1074 ms±514 ms* (p=0.043)<br><br>**Right sided sound**<br>Left stroke 1595 ms±553 ms<br>Control 1501 ms±720 ms *not significant*<br><br>Right stroke 2032 ms±1496 ms<br>Control 1291 ms±792 ms p=0.118<br><br>**Number of correct auditory responses**<br>**Left sided sound**<br>Left stroke 5.15±1.21<br>Control 4.62±1.26 p=0.338<br><br>Right stroke 4.25±1.67<br>Control 4.63±1.19 p=0.307<br><br>**Right sided sound**<br>Left stroke 4.31±1.18<br>Control 4.62±1.71 p=0.3<br><br>Right stroke 4.50±1.31<br>Control 5.50±0.92 p=0.05<br><br>**Seizures outside of neonatal period**<br>Stroke n=4; 19%<br><br>**Hemiparesis**<br>Stroke n=13, 70%<br><br>Right stroke n=3, 28%<br>Left stroke n=10, 77%
35	Northam 2018[37]<br><br>UK<br><br>Prospective cohort	**Population**<br>• Gestation not provided<br>• Born 1991-2001<br><br>**Exposure (n=30)**<br>• Perinatal stroke<br><br>**Control (n=40)**<br>• Matched on age, sex and maternal education<br>• Term infants<br><br>**Ascertainment/ definition**<br>• Arterial or ischaemic stroke confirmed by MRI in the neonatal period	**Outcomes**<br>• Cognitive<br>• Speech and language<br>• Motor (cerebral palsy)<br><br>**Measurement/ assessment**<br>• WASI<br>• CELF<br>• Comprehensive Test of Phonological Processing<br><br>**Follow-up**<br>• 6-18 years (mean 12.4 and 13.5)<br>• 100% follow up	**Cognitive**<br>**Full scale IQ mean (SD)**<br>Stroke 99 (14)<br>Control 112 (16) p<0.0001<br><br>**Mainstream education**<br>Stroke n=28, 93%<br><br>**Receiving additional education support**<br>Stroke n=12, 40%<br><br>**Speech and language**<br>**Expressive language score, mean (SD)**<br>Stroke 95 (17)<br>Control 108 (13) p=0.001<br><br>**Receptive language score, mean (SD)**<br>Stroke 91 (16)<br>Control 104 (14) p < 0.0001<br><br>**Motor (hemiparesis)**<br>Stroke n=9, 3%
36	Tillema 2008[38]<br><br>USA<br><br>Retrospective cohort	**Population**<br>• Gestation not provided<br>• Birth years not provided<br><br>**Exposure (n=10)**<br>• Left perinatal stroke<br><br>**Control (n=10)**	**Outcomes**<br>• Cognitive<br>• Epilepsy<br><br>**Measurement/ assessment**<br>• WISC-III<br>• Language activation tasks – Verb generation task whilst in an fMRI	**Focal epilepsy**<br>Stroke, n=6, 60%<br><br>**Cognitive, mean (SD)**<br>Stroke VIQ 84 (13.4)<br>Control VIQ 108 (14.2) p=0.002

Confidential: For Review Only

		• Matched on age, sex, and handedness<br>• Healthy<br>• Randomly drawn from a large database of children recruited for a different study of language development in healthy children<br><br>**Ascertainment/ definition**<br>• Middle cerebral artery ischaemic stroke	**Follow-up**<br>• 6-16 years<br>• 100% follow up	Stroke FSIQ 80 (14.1)<br>Control FSIQ 108 (11.7) p=0.001
37	Trauner 2001[39]<br><br>USA<br><br>Retrospective cohort	**Population**<br>• Gestation not reported<br>• Birth years not reported<br><br>**Exposure (n=39)**<br>• Left perinatal stroke (n=25)<br>• Right perinatal stroke (n=14)<br><br>**Control (n=54)**<br>• Matched on age and socioeconomic status<br>• Normal neurodevelopmental history<br>• Identified from clinics, community adverts, schools<br><br>**Ascertainment/ definition**<br>• Pre or perinatal onset unilateral brain damage (focal lesion) from cerebral infarction or intraparenchymal haemorrhage<br>• Identified through from clinical referrals.<br>• All confirmed by neuroimaging.<br>• Severity rated on 5-point scale adapted from Vargha-Khadem et al.<br>•	**Outcomes**<br>• Behavioural<br>• Cognitive<br>• Epilepsy<br><br>**Measurement/ assessment**<br>• Achenbach CBCL<br>• WPPSI-R (4-5 years)<br>• WISC-R (6-16 years)<br><br>**Follow-up**<br>• 4-18 years<br>• 100% follow up	**Cognitive**<br>**Full scale IQ mean (SD)**<br>Stroke 93.4 (22)<br>Control 116.2 (13) p<0.0001<br><br>Left stroke 90.1 (22)<br>Right stroke 97.4 (22) – no significant difference<br><br>**Seizures (outside of the neonatal period)**<br>Stroke n=17, 50% (missing data for 5 subjects)
**Central nervous system infections**				
38	Bedford 2001[42]<br><br>England & Wales<br><br>Prospective cohort	**Population**<br>• All gestational ages included<br>• Born 1985-1987<br><br>**Exposure (n=274)**<br>• Neonatal meningitis<br><br>**Comparison (n=1391)**<br>• Matched on age and sex<br>• Recruited through GP<br><br>**Ascertainment/ definition**<br>• Identified through clinician reporting	**Outcomes**<br>• Neuromotor disability (composite)<br>• Cognitive<br>• Hearing<br>• Vision<br>• Behaviour<br>• Seizure disorder<br><br>**Assessment/ measurement**<br>• Parental questionnaire<br>• GP questionnaire<br>• McIntyre et al. classification of disability severity<br><br>**Follow-up**<br>• 5 years<br>• 85-94% follow-up	**Neuromotor disability**<br>Meningitis, n=45, 16%<br>No meningitis, n=2, 0.1%<br><br>**Severe disability**<br>Meningitis, n=20, 7%<br>No meningitis, n=1, 0.1%<br><br>**Moderate disability**<br>Meningitis, n=50, 18%<br>No meningitis, n=20, 1%<br><br>**Mild disorder**<br>Meningitis, n=66, 24%<br>No meningitis, n=275, 20%<br><br>**No disability**<br>Meningitis, n=138, 50%<br>No meningitis, n=1095, 79%
39	Horváth-Puhó 2021[43]<br><br>Denmark and Netherlands<br><br>Retrospective matched cohort study	**Population**<br>• Gestation not specified<br>• Born 1997-2017<br><br>**Exposure**<br>• GBS meningitis (Denmark) (n=168)<br>• GBS meningitis (Netherlands) (n=198)<br><br>**Comparison**<br>• Randomly selected<br>• Matched 1:10 on sex, birth year and month, and gestation<br>• No GBS (Denmark) (n=13,689)<br>• No GBS (Netherlands) (n=4,983)<br><br>**Ascertainment/ definition**<br>• Invasive Group B Streptococcal disease by 89 days of age (most were neonatal – hence inclusion)<br>• ICD 10 codes (Denmark)<br>• CSF culture positive on national laboratory register (Netherlands)	**Outcomes**<br>• Neurodevelopmental impairment (composite)<br>• Cognitive<br>• Motor<br>• Behavioural, mental and social disorders<br>• Hearing impairment<br>• Visual impairment<br><br>**Assessment/ Measurement**<br>• ICD 10 codes<br><br>**Follow-up**<br>• Denmark 5 years, 7 years, 10 years, 15 years<br>• Netherlands 5 years, 7 years, 10 years and 11 years<br>• 95% follow-up	**Any neurodevelopmental impairment RR (95%CI)**<br>**<5 years**<br>Denmark GBS meningitis 7·80 (4·42-13·77)<br>Netherlands GBS meningitis 5·30 (2·57-10·89)<br><br>**<7 years**<br>Denmark GBS meningitis 4·69 (2·78-7·89)<br>Netherlands GBS meningitis 3·71 (1·05-6·72)<br><br>**<10 years**<br>Denmark GBS meningitis 3·47 (2·19–5·50)<br>Netherlands GBS meningitis 2·81 (1·69-4·68)<br><br>**<11 years**<br>Netherlands GBS meningitis 2·99 (1·83-4·88)<br><br>**<15 years**<br>Denmark GBS meningitis 3·15 (1·82–5·46)<br><br>**Moderate to severe neurodevelopmental impairment RR (95%CI)**<br>**<5 years**<br>Denmark GBS meningitis 8·49 (4·28-16·86)<br>Netherlands GBS meningitis 5·13 (2·24-11·79)<br><br>**<7 years**<br>Denmark GBS meningitis 5·27 (2·80-9·92)<br>Netherlands GBS meningitis n/a<br><br>**<10 years**<br>Denmark GBS meningitis 3·88 (2·15–6·99)<br>Netherlands GBS meningitis 3·05 (1·62-5·73)<br><br>**<11 years**<br>Netherlands GBS meningitis 3·34 (1·77-6·33)<br><br>**<15 years**<br>Denmark GBS meningitis 4·52 (2·35–8·67)
40	Martinez-Cruz 2008[45]	**Population**<br>• Gestation < 34 weeks<br>• Birthweight <1500g	**Outcomes**<br>• Sensorineural hearing loss	**Meningitis**<br>Sensorineural hearing loss: n=15; 10.3%<br>No Sensorineural hearing loss: n=7; 2.6%

Confidential: For Review Only

	Mexico<br><br>Retrospective case control	• Born 1990-2005<br><br>**Exposure (n=22)**<br>• Neonatal meningitis<br><br>**Comparator (n=374)**<br>• No meningitis<br><br>**Ascertainment/ definition**<br>• Meningitis not defined	**Assessment/ measurement**<br>• Brainstem Auditory Evoked Potentials<br>• Transient Auditory Evoked Otoacoustic Emissions<br>• Tympanometry<br>• Free Field Audiometry<br>• Pure tone audiometry<br>• Behavioural hearing evaluation<br><br>**Follow-up**<br>• 7- 11 years<br>• 100% follow-up	**Odds of previous neonatal meningitis if sensorineural hearing loss**<br>OR 4.368, 95% CI (1.7, 10.9) p= 0.002
41	Stevens 2003[44]<br><br>England & Wales<br><br>Prospective cohort study	**Population**<br>• Term born infants<br>• Born 1985-1987<br><br>**Exposure (n=111)**<br>• Meningitis<br><br>**Comparison (n=162)**<br>• Matched on hospital of birth, birthweight and sex<br>• Hospital control (n=113)<br>• GP control (n=49)<br><br>**Ascertainment/ definition**<br>• CSF positive culture	**Outcomes**<br>• Disability and functional impairment (composite)<br>• Cognitive<br>• Motor<br>• Vision<br>• Hearing<br><br>**Assessment/ measurement**<br>• WISC-III<br>• Movement ABC<br>• Blinded examination<br>• Hearing screening<br>• Sonksen-Silver acuity system<br><br>**Follow-up**<br>• 9-10 years<br>• 67% follow-up of meningitis group	**Cognitive**<br>**IQ, mean (95% CI)**<br>Meningitis, 88.8 (85, 92)<br>Hospital control, 99.4 (97, 102)<br>GP control, 99.6 (95, 103)<br><br>**Motor**<br>**mABC score, mean (95% CI)**<br>Meningitis 7.1 (5.9, 8.5)<br>Hospital controls 5.0 (4.3, 5.8)<br>GP controls 4.0 (2.9, 5.4)<br><br>**Severe disability/ functional impairment**<br>Meningitis, n=12, 10.8%<br>Hospital control, n=0, 0%<br>GP control, n=0, 0%<br><br>**Moderate disability/ functional impairment**<br>Meningitis, n=10, 9%<br>Hospital control, n=2, 1.8%<br>GP control, n=0, 0%<br><br>**Mild disability/ functional impairment**<br>Meningitis, n=19, 17.1%<br>Hospital control, n=13, 11.5%<br>GP control, n=8, 16%<br><br>**No disability or functional impairment**<br>Meningitis, n=70, 63.1%<br>Hospital control, n=98, 86.7%<br>GP control, n=41, 84%<br><br>**Hearing loss (unilateral or bilateral sensorineural hearing loss or requiring hearing aids)**<br>Meningitis, n=4, 3,6%<br>Hospital control, n=0, 0%<br>GP control, n=0, 0%<br><br>**Visual impairment (bilateral)**<br>Meningitis, n= 18, 17% (6 unassessed because of their disability)<br>Hospital control, n=21, 18.5%<br>GP control, n=4, 8%<br><br>**Visual impairment (unilateral)**<br>Meningitis, n= 10, 9.9% (6 unassessed because of their disability)<br>Hospital control, n=8, 7%<br>GP control, n=2, 4%<br><br>**Seizures outside of the neonatal period**<br>Meningitis, n=6, 5.4%<br>Hospital control, n=2, 1.8%<br>GP control, n=0, 0%
Hypoxic-ischaemic encephalopathy				
42	3383 Koc 2016[24]<br><br>Turkey<br><br>Retrospective cohort	**Population**<br>• Gestation < 32 weeks<br>• Birthweight < 1500g<br>• Born 2001<br><br>**Exposure (n=9)**<br>• Perinatal asphyxia<br><br>**Comparator (n=81)**<br>• No asphyxia<br><br>**Ascertainment/ definition**<br>• Perinatal asphyxia diagnosed on: fetal pH, Apgar score, and neonatal cerebral and multiorgan dysfunction	**Outcomes**<br>• Cognitive<br><br>**Assessment/ measurement**<br>• WISC-R<br>• Performed by blinded psychologist<br><br>**Follow-up**<br>• 5-8 years<br>• 100% follow-up	**Cognitive**<br>**WISC-R IQ Score (combined verbal and performance scores) <85**<br>Perinatal asphyxia n=8, 89%<br>No asphyxia n=24, 30%<br>p=0.001
43	Lee-Kelland 2019[46]*<br><br>United Kingdom<br><br>Retrospective cohort study	**Population**<br>• Gestation ≥ 36 weeks<br>• Born 2008-2010<br><br>**Exposure (n=29)**<br>• Moderate-severe HIE without subsequent cerebral palsy<br><br>**Comparator (n=20)**<br>• Matched on age, sex and social class<br>• Born without HIE<br><br>**Ascertainment/ definition**<br>• Received therapeutic hypothermia based on TOBY trial criteria	**Outcomes**<br>• Cognitive<br>• Motor<br>• Speech and language<br>• Behaviour<br><br>**Assessment/ measurement**<br>• WISC IV (blinded)<br>• Movement ABC 2<br>• Strengths and difficulties questionnaire<br><br>**Follow-up**<br>• 6-8 years<br>• 61% follow-up	**Cognitive**<br>**Full scale IQ, mean (SD)**<br>HIE 91 (10.37)<br>No HIE 105 (13.41)<br>Mean difference −13.62 95% CI (−20.53 to −6.71) p<0.001<br><br>**Perceptual reasoning, mean (SD)**<br>HIE 89 (11.15)<br>No HIE 103 (12.49)<br>Mean difference −13.9 95% CI (−20.78 to −7.09) p<0.001<br><br>**Working memory, mean (SD)**<br>HIE 94 (13.76)<br>No HIE 102 (13.82)<br>Mean difference −8.2 95% CI (−16.29 to −0.17) p=0.04

Confidential: For Review Only

				**Processing speed, mean (SD)**<br>HIE 96 (13.76)<br>No HIE 107 (17.59)<br>Mean difference −11.6 95% CI (−20.69 to −2.47) p=0.01<br><br>**Additional classroom support**<br>HIE n=10, 34%<br>No HIE n=1, 5%<br>OR: 10.0, 95%CI 1.16 to 86.0<br><br>**Special educational needs**<br>HIE n=1, 3.4%<br>No HIE n=0, 0%<br><br>**<u>Motor</u>**<br>**MABC-2 score, mean (SD)**<br>HIE 7.9 (3.26)<br>No HIE 10.2 (2.86)<br>Mean difference −2.12 95% CI (−3.93 to −0.30) p=0.02<br><br>**<u>Speech and language</u>**<br>**Verbal comprehension, mean SD)**<br>HIE 94 (8.79)<br>No HIE 103 (10.09)<br>Mean difference −8.8 95% CI (−14.25 to −3.34) p=0.002<br><br>**<u>Behaviour</u>**<br>**Total difficulties, median (IQR)**<br>HIE 12 (6.5–13.5)<br>No HIE 6 (2.25–10) P=0.005<br><br>**Emotional problems, median (IQR)**<br>HIE 2 (1–4.5)<br>No HIE 0.5 (0–2.75) P=0.03<br><br>**Hyperactivity, median (IQR)**<br>HIE 2 (1–3)<br>No HIE 1 (0–2) P=0.06<br><br>**Conduct problems, median (IQR)**<br>HIE 4 (2.5–6.5)<br>No HIE 3 (1–5) p=0.06<br><br>**Peer problems, median (IQR)**<br>HIE 0 (0–2.5)<br>No HIE 0 (0–1) p=3.56 Ω (potential error in manuscript table)<br><br>**Prosocial, median (IQR)**<br>HIE 9 (7.5–10)<br>No HIE 9 (8.25–10) p=0.13<br><br>**Impact score, median (IQR)**<br>HIE 0 (0–2.5)<br>No HIE 0 (0–2.0) p=0.31
44	Tonks 2019[47]*<br><br>United Kingdom Prospective cohort study	**Population**<br>• Gestation ≥36 weeks<br>• Born 2008-2011<br>• English as primary language<br><br>**Exposure (n=29)**<br>• Moderate-severe HIE without subsequent cerebral palsy<br><br>**Comparator (n=20)**<br>• Matched on age, sex and social class<br>• Recruited from schools in the area<br>• Born without HIE<br><br>**Ascertainment/ definition**<br>• Received therapeutic hypothermia based on TOBY trial criteria	**Outcomes**<br>• Cognitive<br>• Neuropsychological<br><br>**Assessment/ measurement**<br>• Conner's continuous performance test<br>• NEPSY-II block construction test<br>• NEPSY-II arrows' test<br><br>**Follow-up**<br>• 6-8 years<br>• 77% follow-up	**<u>Attention</u>**<br>**Hit response time**<br>**HIE**<br>84.1 percentile mean rank 27;<br>Proportion performing below 2 SD 32%<br><br>**Comparator**<br>67.3 percentile mean rank 17.89; p = .024<br>Proportion performing below 2 SD 11%<br><br>**Hit response time standard error**<br>**HIE**<br>standard error mean rank 26.8<br>Proportion performing below 2 SD 18%<br><br>**Comparator**<br>standard error mean rank 18.2; p = 0.032<br>Proportion performing below 2 SD 11%<br><br>**Hit response time by block**<br>**HIE**<br>Mean 49.1, SD 23.9<br><br>**Comparator**<br>Mean 61.9, SD 18.4; p = 0.047<br><br>**<u>Visual discrimination</u>**<br>**<u>HIE</u>**<br>Below 1 SD 10%<br><br>**<u>Comparator</u>**<br>Below 1 SD 5%<br>HIE vs comparator scores, p = 0.049<br><br>**<u>Visuo-spatial mental rotation task</u>**<br>**<u>HIE</u>**<br>Below 1 SD 17%<br><br>**<u>Comparator</u>**<br>Below 1 SD 5%<br>HIE vs comparator scores, p = 0.034

Confidential: For Review Only

Confidential: For Review Only

Supplement 4: Risk of bias table

overlapping data; Clinical Evaluation of Language Fundamentals (CELF); Cystic Periventricular leukomalacia (cPVL); Intelligence Quotient (IQ); Intraventricular haemorrhage (IVH); Mental Developmental Index (MDI); Neonatal Intensive Care Unit (NICU); Psychomotor Development Index (PDI); Periventricular leukomalacia (PVL); Spontaneous Intestinal Perforation (SIP); Wechsler Intelligence Scale for Children (WISC); White Matter Injury (WMI);

Preterm brain injury: cohort studies

	Selection (*satisfactory; No =not satisfactorily done; n/a)				Comparability (*satisfactory; No =not satisfactorily done; n/a)		Exposure/ Outcome (*satisfactory; No =not satisfactorily done; n/a)			Subtotal assessment			Total score: 0-3 high risk of bias; 4-6 moderate risk of bias 7-9 low risk of bias	Additional comments
	1	2	3	4	1a	1b	1	2	3	Selection (0-1=Poor; 2=Fair; 3+ Good)	Comparability (0=poor; 1=fair; 2+=good)	Exposure / outcome (0=poor; 1=fair; 2+=good)		
Adant 2019	No	*	*	* (excluded those with congenital anomalies)	*	*	No	*	No	Good	Good	Fair	6	Population not representative as focus of study was spontaneous intestinal perforation. Infants without IVH didn't have brain injury excluded per se (but didn't have IVH 3-4 on imaging). Matched on gender, gestational age, date of birth. Multiples matched to sibling without SIP. Excluded those with necrotising enterocolitis, mechanical obstruction or congenital anomalies. Adjusted for gender, gestation, birthweight, SIP and IVH.<br><br>Independent outcome assessment but not blinded; telephone survey of parents. High numbers lost to follow-up. Table 3 contains errors with respect to outcomes (MDI and PDI mislabelled as motor and cognitive respectively).

Confidential: For Review Only

Beaino 2010#	*	*	No	* (cerebral palsy could not be present at birth)	*	*	*	*	*	Good	Good	Good	8	3% of infants did not have a cranial ultrasound, a further 11% had only one cranial ultrasound during neonatal period - therefore ascertainment of exposure may be compromised<br><br>Model A adjusted for:<br>• obstetric factors<br>• cerebral lesions<br>Model B adjusted for:<br>• obstetric factors<br>• neonatal factors<br><br>Model C was the same as model B for those without cPVL or Intraparenchymal haemorrhage<br><br><85% follow-up for enrolled infants but clear description of those lost to follow-up and no significant differences with respect to ultrasound brain injury findings between groups
Brouwer 2012	No	No	*	* (given the types of outcomes assessed)	No	No	No	*	*	Fair	Poor	Good	4	Study of a select group i.e. those with IVH requiring neurosurgical intervention.<br>No description of setting, how patients were enrolled, how many were excluded<br>No description of how control group was derived, or what era they were from.<br>Only some infants (those <30weeks) were matched on gestation, birthweight, sex to controls.<br>Different intelligence tests used at follow-up. >80% completion rate of Child Behaviour Checklist and teacher report form by parents and teachers

Confidential: For Review Only

Campbell 2021	*	*	*	* (given the types of outcomes assessed)	*	*	*	*	No	Good	Good	Good	8	Males and those born at 23-24 weeks gestation were overrepresented in the IVH WMI group.<br>Adjusted for gestation, birthweight Z score, sex, maternal education, bronchopulmonary dysplasia, sepsis, necrotising enterocolitis (Bell stage 2-3) and severe retinopathy of prematurity.
Cheong 2018	*	*	*	No (visual or hearing impairment could be congenital)	*	*	*	*	*	Good	Good	Good	8	Adjusted for era of birth, antenatal corticosteroid exposure, inborn status, gestation, sex, multiple birth, birthweight Z score, surfactant use, IVH grade 3 or 4 (in cPVL), cPVL (in IVH grade 3-4), bronchopulmonary dysplasia, postnatal corticosteroid use, necrotising enterocolitis (stage 2 or worse), surgery in the newborn period, and retinopathy of prematurity (stage 3 or worse).
Chou 2020	*	*	*	* (given the types of outcomes assessed)	No	*	*	*	No	Good	Fair	Good	7	Matched and adjusted for, urbanisation and parental occupation.<br><br>No information about missing data or completeness of follow-up

Confidential: For Review Only

Davidovitch 2020	*	*	*	* (given the types of outcomes assessed)	No	*	*	*	No	Good	Fair	Good	7	Only low birthweight infants included (therefore birthweight partially accounted for). Unmatched.<br>No information about excluding brain injury from comparators e.g. comparing those with IVH grade 3-4 to those without could include those with IVH 1-2; both groups could also include infants with other types of brain injury.<br>Missing data not presented or accounted for. Adjusted the composite brain injury group (which included retinopathy of prematurity in its definition) for gestation, maternal diabetes, small for gestational age, year of birth, bronchopulmonary dysplasia, and receipt of postnatal steroids.
Doyle 2000 #	*	*	*	* (given the types of outcomes assessed)	No	No	*	*	*	Good	Poor	Good	7	IVH and no IVH groups not matched for gestation or birthweight, no adjustment for these variables appears to have been done.<br><br>Relatively old cohort (most did not receive surfactant), comparator group only includes infants born in the 1980s. Not representative due to time-period of care.
Hintz 2018	*	*	*	* (given the types of outcomes assessed)	*	*	*	*	*	Good	Good	Good	9	Assessed interobserver reliability of central imaging readers.<br><br>Unmatched<br><br>Adjusted for gestation, race, sex, multiple gestation, maternal education, sepsis, bronchopulmonary dysplasia, postnatal steroids, surgery for patent ductus arteriosus, necrotising enterocolitis, retinopathy of prematurity.<br><br>Only 83% follow-up of survivors but those lost to follow-up are accounted for.

Confidential: For Review Only

Hirovonen 2017	*	*	*	* (given the types of outcomes assessed)	*	*	*	*	*	Good	Good	Good	9	Excluded infants who died at <1 year of age, infants with major congenital anomalies, and those with missing data.<br><br>Characteristics of those with brain injury not presented.<br><br>No breakdown by severity of brain injury because that level of detail was not available in the database.<br><br>No matching but there is stratification by gestation and adjustment for: maternal characteristics, pregnancy characteristics, delivery characteristics, sex, gestation, birthweight, Apgar score at 1-minute, umbilical artery pH, resuscitation provided, NICU admission, receipt of phototherapy, ventilator requirement, antibiotic receipt, respiratory distress syndrome, sepsis, seizures, hyperbilirubinaemia.
Hollebrandse 2021	*	*	*	* (given the types of outcomes assessed)	*	*	*	*	*	Good	Good	Good	9	Gestation similar across all groups and other baseline perinatal characteristics similar across groups.<br><br>Preterm brain injury and no brain injury group not matched. Unclear if IVH and no IVH group had other brain injuries excluded or may have had more than one injury type (e.g. PVL).<br>Impact of epoch/ era of birth explored and adjusted for.
Hreinsdottir 2018	*	*	*	No (visual impairment could have been congenital)	*	*	*	*	No	Good	Good	Good	7	Unsure if comparator group in logistic regression includes those with IVH 1-2. Adjusted for gestation, birthweight, retinopathy of prematurity, sex, cognitive score, cerebral palsy.

Jansen 2020	*	*	*	* (given the types of outcomes assessed)	No	No	*	*	No	Good	Poor	Good	6	Excluded infants with congenital abnormalities, metabolic disorders or neonatal meningitis.
Kaur 2020	*	*	*	No (visual or hearing impairment could be congenital)	No	No	*	*	No	Good	Poor	Good	5	Unmatched. Compared IVH with all infant without haemorrhage (of all gestations).
Kiechl-Kohlendorf er 2013	*	*	*	* (given the types of outcomes assessed)	*	*	*	No	No	Good	Good	Fair	7	Low numbers of infants included. Outcomes assessed at 1 year - likely not long enough for robust assessment of neurodevelopmental outcomes; <85% follow-up and no detailed description of those lost to follow up - though authors do state that there were no significant differences between those followed up and those lost to follow up.
Klebermass -Schrehof 2012	*	*	*	No (could have had congenital blindness)	*	*	*	*	No	Good	Good	Good	7	Adjusted for gestation. No clear description of number lost to follow-up, though mentions that follow-up rate at 5.5 years was 54-61%.
Koc 2016	*	*	No	* (given the types of outcomes assessed)	No	No	*	*	No	Good	Poor	Good	5	Small numbers included. No breakdown of characteristics of those with brain injury. No description of IVH grading used or schedule of ultrasound exams; no description of criteria for establishing perinatal asphyxia, number lost to follow-up not stated.
Neubauer 2008	*	n/a	*	No (deafness or blindness could have been congenital)	*	*	*	*	*	Fair	Good	Fair	7	Neurodevelopmental assessors not blinded; follow-up rate <85% but paper does give description of those lost to follow-up

Confidential: For Review Only

Piris Borregas 2019	*	*	*	* (excluded infants with congenital malformations)	No	No	*	*	No	Good	Poor	Good	6	Only those followed up to 7 years included.<br><br>Excluded infants who died before 36 weeks corrected age, with major malformations, or those with missing data.<br><br>Unclear if independent odds ratio includes adjustment for covariates.<br><br>Unclear if those without 'severe brain injury' had other types of brain injury.
Pittet 2019	*	*	*	* (excluded infants with congenital malformations)	No	*	*	*	*	Good	Fair	Good	8	Excluded infants with congenital malformations affecting neurodevelopment and infants from centres without 5 years of follow-up cognitive testing.<br><br>Unclear if other types of brain injury excluded from comparator group.<br><br>Adjusted for gender and socioeconomic status. No significant difference in cognitive outcome between extreme preterms and those 28-30 weeks' gestation. Gestation not adjusted for.
Sherlock 2005#	*	*	*	No (deafness or blindness could have been congenital)	No	No	*	*	*	Good	Poor	Good	6	Comparability of IVH vs. no IVH cohorts not clear - not enough information to determine if groups were comparable with respect to gestational age or birthweight
Tymofiyeva 2018	*	*	*	* (given the types of outcomes assessed)	No	No	*	*	No	Good	Poor	Good	6	Excluded infants with congenital malformations/ syndromes, congenital infections, or those who were too unstable for MR imaging. The last exclusion criteria in particular could limit generalisability quite considerably.<br><br>Unclear about the validity of grouping the attention scores across different assessment tools together into a dichotomous variable for attention.

Van De Bor 2004	*	*	*	* (excluded those with major congenital malformations)	*	*	No	No	*	Good	Good	Fair	7	IVH vs. no IVH cohorts comparable with respect to gestation; some differences in gender composition but paper states this was controlled for in the analysis. Primary outcome entirely self-reported. Outcomes reported at 14 years.
Van Den Hout 2000	* (except for HIE exposure group)	*	*	* (excluded those with congenital anomalies)	No	No	*	*	*	Good	Poor	Good	7	Low numbers and relatively old cohort. Relative gender imbalance in IVH group compared to those with normal scans or PVL. IVH group also 1.4 weeks more premature than 'normal scan' group.
Vollmer 2003#	*	*	*	No (deafness or blindness could have been congenital)	*	No	*	*	*	Good	Fair	Good	7	Note change in version of Weschler scale during follow-up period. Authors state no difference in mean IQ after change. Baseline characteristics of groups with and without brain injury not given; no indication of matching or adjustment for factors other than gestation.
Vollmer 2006a#	*	*	*	No (deafness or blindness could have been congenital)	*	*	*	*	*	Good	Good	Good	8	Note gender imbalance in cohort as a whole (M>F), but male: female ratio in each group appears similar.<br>No matching or adjustment for covariates.<br><br><85% follow-up but clear description of those lost and appears no significant differences.
Vollmer 2006b#	*	*	*	No (deafness or blindness could have been congenital)	No	No	*	*	No	Good	Poor	Good	5	Marked gender imbalance in ventricular dilatation group. Lower birthweight and gestation in groups with abnormal cranial ultrasound. No indication of matching or adjustment.<br><br><85% follow-up and the limited description of those lost to follow-up indicates that these babies were of lower birthweight and gestation.

Confidential: For Review Only

Whitaker 2011	*	*	*	* (given the types of outcomes assessed)	*	*	(No)	*	*	Good	Good	Good	8	Severely disabled survivors (n=33) were excluded.<br><br>Half had later ultrasounds (just before discharge).<br><br>No breakdown of the characteristics of the exposed and comparator groups – unable to assess how comparable they are.<br><br>Adjusted for: maternal social risk, sex, gestation, fetal growth ratio, multiplicity, maternal smoking status, maternal alcohol status, labour onset, presentation at birth, base excess on first postnatal blood gas, thyroid status, hypocapnia, hypoxia, systolic hypotension, prolonged ventilation.<br><br>Primary outcome assessment reliant on parental report, albeit via structured interview with some evidence for validity. Interviewers were blinded to the child's history. Parents were blinded to the study hypothesis.<br><br>Less than 85% follow-up (psychiatric interviews in 51% of survivors) however clear descriptions of groups with and without psychiatric evaluation given in table 2 and little apparent difference between groups.
**Preterm brain injury: case-control studies**														
	1 Case definition	2 Representativeness of cases	3 Selection of controls	4 Definition of controls	1a	1b	1 Ascertainment of exposure	2 Same method of ascertainment for cases and controls	3 Non-response rate	(0-1=Poor; 2=Fair; 3+ Good)	y (0=poor; 1=fair; 2+=good)	(0=poor; 1=fair; 2+=good)	Total score: 0-3 high risk of bias; 4-6 moderate risk of bias 7-9 low risk of bias	Additional comments

Confidential: For Review Only

Martinez-Cruz 2008 (IVH)	*	*	*	*	*	No	*	*	No	Good	Fair	Good	7	Appears to be case-control design hence star ratings are as per case control rating sheet. Controls not well matched for birth weight. No description of whether full information on exposures could be obtained for all cases/controls e.g. missing records etc.
Perinatal stroke: cohort studies														
	Selection (*satisfactory; No =not satisfactorily done; n/a)				Comparability (*satisfactory; No =not satisfactorily done; n/a)		Exposure/ Outcome (*satisfactory; No =not satisfactorily done; n/a)			Subtotal assessment			Total score: 0-3 high risk of bias; 4-6 moderate risk of bias 7-9 low risk of bias	Additional comments
	1	2	3	4	1a	1b	1	2	3	Selection (0-1=Poor; 2=Fair; 3+ Good)	Comparability (0=poor; 1=fair; 2+=good)	Exposure / outcome (0=poor; 1=fair; 2+=good)		
Ballantyne 2007	No	No	*	*	No	*	No	*	No	Fair	Fair	Fair	4	No description of derivation of exposed cohort - whether single institute or multicentre, whether same community as non-exposed group or not.<br><br>Predominance of right-handed children amongst controls otherwise similar baseline characteristics. Note male preponderance in exposed group and female preponderance in non-exposed<br><br>No matching or adjustment for confounders.<br><br>No description of who performed outcome assessment, whether blinded and independent.
Ballantyne 2008	*	*	*	No	No	*	*	*	No	Good	Fair	Good	6	Excluded children with brain lesions from other causes e.g. head trauma, tumours

														Gestational age of exposed cohort ranged from 32 to 40 weeks. No statement as to whether control group were matched on this. Note preponderance of males in stroke group and females in control group.<br><br>In study 1, significant numbers of participants did not complete the planned developmental assessments - across exposed and control groups, completeness ranged from 50% for WISC-R to 69% for CELF-R.
Gold 2014	No	No	*	*	No	*	*	*	*	Fair	Fair	Good	6	No description of how subjects were selected or recruited from neurology clinics. Nonexposed group selected from a different source. No description of gestational age of subjects or of controlling for this. Matched for age at follow up, sex, socioeconomic group and maternal education.<br><br>Excluded infants with bilateral lesions, a history of hypoxic ischemic encephalopathy, central nervous system infection, in-utero drug exposure, significant closed head injury, or any other condition that might have caused brain damage other than from the stroke.
Kolk 2011	*	*	*	*	No	*	*	*	No	Good	Fair	Good	7	No description of gestational age of subjects or of controlling for this. Difficult to ascertain completeness of follow-up from paper. Adjusted for age of outcome assessment.
Martin 2019	*	*	*	*	No	*	*	*	*	Good	Fair	Good	8	Excluded infants with bilateral lesions, hearing impairment, or a history of a problem that may have caused more global brain damage (e.g. meningitis, closed head injury, hypoxic-ischemic encephalopathy). Matched on age, sex and socioeconomic status

Confidential: For Review Only

Northam 2018	*	No	*	*	*	*	*	*	*	Good	Good	Good	8	No description of source of unexposed cohort. Matched on age, sex, and maternal education.
Tillema 2008	*	*	*	*	No	*	*	*	No	Good	Fair	Good	7	Exposed and comparator groups not matched for gestation, but were matched for age, sex and handedness. 17 subjects included initially but 7 of these excluded for various reasons meaning that neurodevelopmental outcome data/Weschler scores only presented for 10 of 17.
Trauner 2013	*	*	*	*	No	No	No	*	No	Good	Poor	Fair	5	Excluded infants if bilateral or multifocal lesions identified, history of meningitis, or history of antenatal drug exposure<br><br>Matched on age and socioeconomic status<br><br>No baseline characteristics given to establish comparability of exposed and comparator cohorts. Likely comparable with regards to gestation based on stated inclusion criteria. Main outcome measure based on parental questionnaire - no direct linguistic assessments done, however may not have been feasible/appropriate in such a young cohort. No information on response rate/loss to follow-up.<br><br>IQ used as covariate<br><br>IQ combined across the age range and assessed with two different tools. This assumes IQ is fixed which may not be true.
Central nervous infections: cohort studies														

Confidential: For Review Only

	Selection (*satisfactory; No =not satisfactorily done; n/a)				Comparability (*satisfactory; No =not satisfactorily done; n/a)		Exposure/ Outcome (*satisfactory; No =not satisfactorily done; n/a)			Subtotal assessment			Total score: 0-3 high risk of bias; 4-6 moderate risk of bias 7-9 low risk of bias	Additional comments
	1	2	3	4	1a	1b	1	2	3	Selection (0-1=Poor; 2=Fair; 3+ Good)	Comparability (0=poor; 1=fair; 2+=good)	Exposure / outcome (0=poor; 1=fair; 2+=good)		
Bedford 2001#	*	*	*	No	*	*	No	*	*	Good	Good	Good	7	Matched on sex and age.<br><br>Study focuses on meningitis in infancy but also presents outcomes after neonatal meningitis.<br><br>Did not exclude children with other comorbidities e.g. congenital conditions associated with neurodevelopmental impairment. Exposed cases derived from same cohort as Stevens 2003. Outcome assessment based on parent or GP report with no formal neurodevelopmental assessment.
Horváth-Puhó 2021	*	*	*	No	*	*	*	*	*	Good	Good	Good	8	Invasive Group B Streptococcal infection diagnosed in the first 89 days (however most of these were neonatal, particularly in the first week of life (45%) hence inclusion.<br><br>Matched 1:10 on sex, birth year and month, and gestation.<br>Neurodevelopmental impairment defined differently in each cohort.<br>Missing data accounted for and its impact explored.

Confidential: For Review Only

Stevens 2003#	(*)	(*)	*	No	*	*	*	*	No	Good	Good	Good	7	Exposed cohort based on recall of consultant paediatricians filling out monthly returns thus may be biased towards more severe or otherwise memorable cases. Some in comparator group selected from a different hospital than exposed cohort.<br><br>Matched on hospital of birth, birth weight and sex.<br><br>Results stratified by birthweight<br><br>Significant rate of loss to follow-up.
**Central nervous system infections: case control studies**														
	1 Case definition	2 Representativeness of cases	3 Selection of controls	4 Definition of controls	1a	1b	1 Ascertainment of exposure	2 Same method of ascertainment for cases and controls	3 Non-response rate	(0-1=Poor; 2=Fair; 3+ Good)	y (0=poor; 1=fair; 2+=good)	(0=poor; 1=fair; 2+=good)	Total score: 0-3 high risk of bias; 4-6 moderate risk of bias 7-9 low risk of bias	Additional comments
Martinez-Cruz 2008	*	*	*	*	No	No	*	*	No	Good	Poor	Good	6	Excluded those with history of parental consanguinity or TORCH infections.<br><br>Number of those with and without meningitis who may have had other types of brain injuries not specified – unable to assess overlap/ impact of meningitis alone.<br><br>Odds ratio presented for meningitis does not appear to be crude so potential adjustment for confounding factors but no description of this in the methods section.<br><br>No description of proportion of missing data.
**Hypoxic-ischaemic encephalopathy: cohort studies**														

Confidential: For Review Only

	Selection (*satisfactory; No =not satisfactorily done; n/a)				Comparability (*satisfactory; No =not satisfactorily done; n/a)		Exposure/ Outcome (*satisfactory; No =not satisfactorily done; n/a)			Subtotal assessment			Selection (*satisfactory; No =not satisfactorily done; n/a)	Additional comments
	1	2	3	4	1a	1b	1	2	3	Selection (0-1=Poor; 2=Fair; 3+ Good)	Comparability (0=poor; 1=fair; 2+=good)	Exposure / outcome (0=poor; 1=fair; 2+=good)	Total score: 0-3 high risk of bias; 4-6 moderate risk of bias 7-9 low risk of bias	
Koc 2016	No	*	*	*	No	No	*	*	No	Fair	Poor	Good	5	Representativeness not clear as no description given of babies who did not complete follow-up at the study institution. No apparent adjustment for gestation or other covariates. Pre-therapeutic hypothermia era.<br><br>Small number, no breakdown of characteristics or other neurodevelopmental outcomes by brain injury<br><br>Number of those with and without birth asphyxia who had other types of brain injuries e.g. IVH not specified.
Lee-Kelland 2019	No	*	*	*	*	*	*	No	No	Good	Good	Good	6	Excluded those who underwent therapeutic hypothermia outside of the standard criteria, infants with metabolic disorders and non-English speaking infants.<br><br>Matched on age, sex and social class.
Tonks 2019	*	No	*	*	No	*	*	*	No	Good	Fair	Good	6	Included cases had no diagnoses other than encephalopathy.<br>Excluded infants with neurological issues other than encephalopathy. Matched on age, sex and socioeconomic status.

Confidential: For Review Only

Study	Events	Total	Incidence	95% CI	Weight
Ballantyne, 2008	11	29	0.379	[0.207; 0.577]	22.2%
Kolk, 2011	9	21	0.429	[0.218; 0.660]	19.0%
Martin, 2019	4	21	0.190	[0.054; 0.419]	23.1%
Tilema, 2008	6	10	0.600	[0.262; 0.878]	12.5%
Trauner, 2001	17	34	0.500	[0.324; 0.676]	23.1%
**Overall**			**0.401**	**[0.268; 0.533]**	**100.0%**

I^2 = 56%, τ^2 = 0.0124, p = 0.06

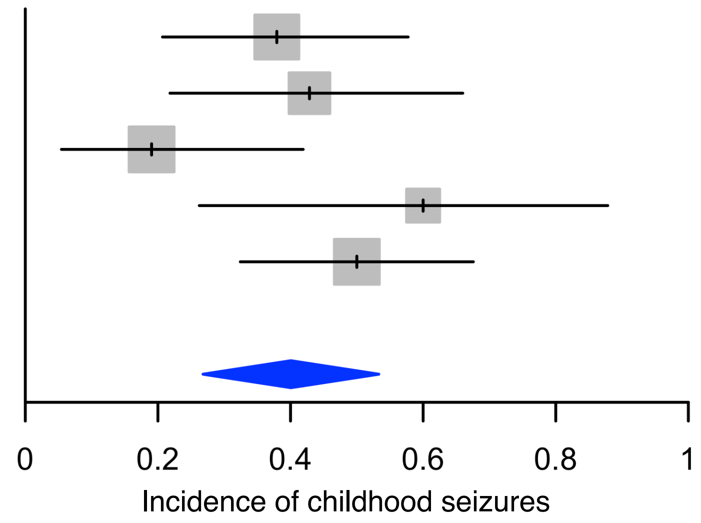

Study	Events	Total	Incidence	95% CI	Weight
Ballantyne, 2008	18	29	0.621	[0.423; 0.793]	19.9%
Gold, 2004	16	27	0.593	[0.388; 0.776]	19.6%
Kolk, 2011	19	21	0.905	[0.696; 0.988]	21.3%
Martin, 2019	13	21	0.619	[0.384; 0.819]	19.0%
Northam, 2017	9	30	0.300	[0.147; 0.494]	20.3%
**Overall**			**0.610**	**[0.392; 0.829]**	**100.0%**

$I^2 = 88\%, \tau^2 = 0.0545, p < 0.01$

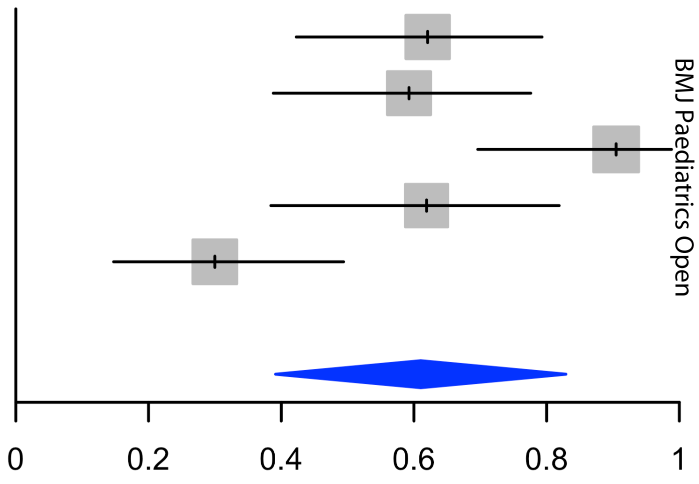

https://mc.manuscriptcentral.com/bmjpo

	Perinatal stroke			Control				Mean Difference
**Study or Subgroup**	**Mean**	**SD**	**Total**	**Mean**	**SD**	**Total**	**Weight**	**IV, Random, 95% CI**
Ballantyne 2008	82.3	20.1	29	111.4	13.7	38	49.0%	-29.10 [-37.61, -20.59]
Northam 2017	91	16	30	104	14	40	51.0%	-13.00 [-20.18, -5.82]
**Total (95% CI)**			**59**			**78**	**100.0%**	**-20.88 [-36.66, -5.11]**

Heterogeneity: Tau² = 113.45; Chi² = 8.02, df = 1 (P = 0.005); I² = 88%
Test for overall effect: Z = 2.59 (P = 0.009)

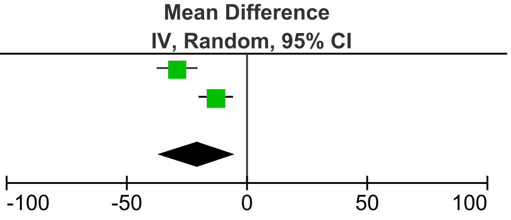

Confidential: For Review Only

Confidential: For Review Only

	Perinatal stroke			Control				Mean Difference
**Study or Subgroup**	**Mean**	**SD**	**Total**	**Mean**	**SD**	**Total**	**Weight**	**IV, Random, 95% CI**
Ballantyne 2008	78.4	16	29	105.8	11.9	38	50.3%	-27.40 [-34.34, -20.46]
Northam 2017	95	17	30	108	13	40	49.7%	-13.00 [-20.30, -5.70]
**Total (95% CI)**			**59**			**78**	**100.0%**	**-20.25 [-34.36, -6.13]**

Heterogeneity: Tau² = 90.47; Chi² = 7.85, df = 1 (P = 0.005); I² = 87%
Test for overall effect: Z = 2.81 (P = 0.005)

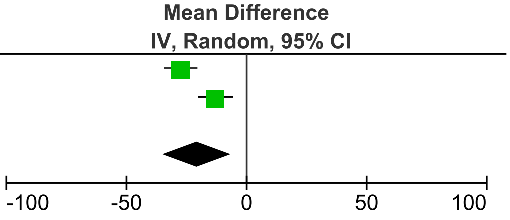

BMJ Paediatrics Open

School-age outcomes of children after perinatal brain injury: a systematic review and meta-analysis

Journal:	*BMJ Paediatrics Open*
Manuscript ID	bmjpo-2022-001810.R2
Article Type:	Original research
Date Submitted by the Author:	07-Feb-2023
Complete List of Authors:	Rees, Philippa; University College London Institute of Child Health, Population policy and Practice<br>Callan, Caitriona; University of Oxford Nuffield Department of Primary Care Health Sciences<br>Chadda, Karan; Cambridge University Hospitals NHS Foundation Trust, Department of Paediatrics<br>Vaal, Meriel; University College London Institute of Child Health, University College London and Great Ormond Street Institute of Child Health<br>Diviney, James; Great Ormond Street Hospital for Children NHS Foundation Trust, Paediatric Intensive Care Unit<br>Sabti, Shahad; King's College London<br>Harnden, Fergus; Chelsea and Westminster Hospital NHS Foundation Trust<br>Gardiner, Julian; University College London Institute of Child Health, University College London and Great Ormond Street Institute of Child Health<br>Battersby, Cheryl; Imperial College London, Neonatal Medicine<br>Gale, Chris; Imperial College London, Neonatal Medicine<br>Sutcliffe, Alastair; University College London Institute of Child Health, University College London and Great Ormond Street Institute of Child Health
Keywords:	Epidemiology, Neonatology

SCHOLARONE™
Manuscripts

I, the Submitting Author has the right to grant and does grant on behalf of all authors of the Work (as defined in the below author licence), an exclusive licence and/or a non-exclusive licence for contributions from authors who are: i) UK Crown employees; ii) where BMJ has agreed a CC-BY licence shall apply, and/or iii) in accordance with the terms applicable for US Federal Government officers or employees acting as part of their official duties; on a worldwide, perpetual, irrevocable, royalty-free basis to BMJ Publishing Group Ltd ("BMJ") its licensees and where the relevant Journal is co-owned by BMJ to the co-owners of the Journal, to publish the Work in this journal and any other BMJ products and to exploit all rights, as set out in our licence.

The Submitting Author accepts and understands that any supply made under these terms is made by BMJ to the Submitting Author unless you are acting as an employee on behalf of your employer or a postgraduate student of an affiliated institution which is paying any applicable article publishing charge ("APC") for Open Access articles. Where the Submitting Author wishes to make the Work available on an Open Access basis (and intends to pay the relevant APC), the terms of reuse of such Open Access shall be governed by a Creative Commons licence – details of these licences and which Creative Commons licence will apply to this Work are set out in our licence referred to above.

Other than as permitted in any relevant BMJ Author's Self Archiving Policies, I confirm this Work has not been accepted for publication elsewhere, is not being considered for publication elsewhere and does not duplicate material already published. I confirm all authors consent to publication of this Work and authorise the granting of this licence.

Confidential: For Review Only

School-age outcomes of children after perinatal brain injury: a systematic review and meta-analysis

Philippa Rees[1] MPhil MBBCh, Caitriona Callan[2] MB BChir, Karan R Chadda[3] MB BChir, Meriel Vaal MRes MBChB[1], James Diviney[4] MB BChir, Shahad Sabti[5] MBBS, Fergus Harnden[6] MBChB, Julian Gardiner[1]PhD, Cheryl Battersby[7] PhD, Chris Gale[7] PhD, Alastair Sutcliffe[1] PhD

Affiliations:
1. Population Policy and Practice, Great Ormond Street UCL Institute of Child Health, London, UK.

2. Nuffield Department of Primary Care Health Sciences, University of Oxford.

3. Department of Paediatrics, Cambridge University Hospitals NHS Foundation Trust, Cambridge, UK.

4. Paediatric Intensive Care Unit, Great Ormond Street Hospital, London, UK

5. Kings College London, UK.

6. Chelsea and Westminster Hospital NHS Foundation Trust, London, UK.

7. Neonatal Medicine, School of Public Health, Faculty of Medicine, Imperial College London, London, UK.

Address correspondence to: Dr Philippa Rees, Population Policy Practice, UCL Institute of Child Health, 1st Floor 30 Guilford Street, London, WC1N 1EH, p.rees@ucl.ac.uk

Confidential: For Review Only

School-age outcomes of children after perinatal brain injury: a systematic review and meta-analysis

Background
Over 3,000 children suffer a perinatal brain injury in England every year according to national surveillance. The childhood outcomes of infants with perinatal brain injury are however unknown.

Methods
A systematic review and meta-analyses were undertaken of studies published between 2000-September 2021 exploring school-aged neurodevelopmental outcomes of children after perinatal brain injury compared to those without perinatal brain injury. The primary outcome was neurodevelopmental impairment which included cognitive, motor, speech and language, behavioural, hearing, or visual impairment after 5 years of age.

Results
This review included 42 studies. Preterm infants with intraventricular haemorrhage (IVH) grade 3-4 were found to have a three-fold greater risk of moderate-severe neurodevelopmental impairment at school age OR 3.69 (95%CI: 1.7, 7.98) compared to preterm infants without IVH. Infants with perinatal stroke had an increased incidence of hemiplegia 61% (95%CI: 39.2, 82.9) and an increased risk of cognitive impairment (difference in full scale IQ -24.2 (95%CI: -30.73, -17.67) . Perinatal stroke was also associated with poorer academic performance; and lower mean receptive -20.88 (95%CI: -36.66, -5.11) and expressive language scores -20.25 (95%CI: -34.36, -6.13) on the CELF assessment. Studies reported an increased risk of persisting neurodevelopmental impairment at school age after neonatal meningitis. Cognitive impairment and special educational needs were highlighted after moderate-severe HIE. However, there were limited comparative studies providing school-aged outcome data across neurodevelopmental domains and few provided adjusted data. Findings were further limited by the heterogeneity of studies.

Conclusions
Longitudinal population studies exploring childhood outcomes after perinatal brain injury are urgently needed to better enable clinicians to prepare affected families, and to facilitate targeted developmental support to help affected children reach their full potential.

Confidential: For Review Only

School-age outcomes of children after perinatal brain injury: a systematic review and meta-analysis

What is already known on this topic
Thousands of children suffer a brain injury around the time of birth every year. Many of these injuries are associated with neurodevelopmental impairment at two years of age. However, two-year outcomes are not necessarily representative of later childhood outcomes and function, which are a priority for parents.

What this study adds
This review provides an overview of existing evidence of childhood outcomes after perinatal brain injury. It indicates that there is some evidence of on-going impairment throughout childhood for different types of perinatal brain injury but that there are considerable gaps in knowledge.

How this study might affect research, practice or policy
This review shows the need for detailed high-quality longitudinal population studies exploring childhood outcomes after perinatal brain injury

Confidential: For Review Only

School-age outcomes of children after perinatal brain injury: a systematic review and meta-analysis

Perinatal brain injuries can have wide-ranging deleterious consequences for children, families and broader society.(1-4) Over 3,000 infants experience perinatal brain injury in England annually[1] and the Department of Health and Social Care (DHSC) has committed to halving the rate of perinatal brain injuries by 2030 as part of the national maternity ambition.(5) To monitor progress towards this goal, a standardised definition of perinatal brain injury was developed.(6) The degree to which this definition captures and represents true perinatal brain injuries is unclear and requires us to look beyond the neonatal period.(6)

Focusing on the childhood outcomes of infants with perinatal brain injury provides a fuller understanding of the population captured by the DHSC definition. Despite their importance to families, school-age outcomes following neonatal care have been an overlooked research priority. Neonatal studies typically focus on two-year composite outcomes which may mask the true neurodevelopmental burden of injuries, and are known to be poorly predictive of future functioning.(7-10) As such, our understanding of childhood developmental trajectories after brain injuries – and whether any sequelae are fixed, stable or amenable to interventions – is limited. We therefore undertook a systematic review to explore school-age neurodevelopmental outcomes following perinatal brain injury.

Confidential: For Review Only

METHODS

Study selection

The review was conducted as per the pre-registered protocol (CRD 42021278572) and the PRISMA statement.(11) We included observational comparative studies exploring neurodevelopmental outcomes of children over five years of age after perinatal brain injury, published between 2000-September 2021 (Table 1). The DHSC definition of perinatal brain injuries used includes intraventricular haemorrhage, preterm white matter injuries, stroke, central nervous system infection, hypoxic ischaemic encephalopathy, and kernicterus diagnosed during the neonatal period.(6, 12) We did not include seizures in isolation. For inclusion, studies were required to have a non-brain injured comparator group. The primary outcome was neurodevelopmental impairment; secondary outcomes included motor, cognitive, speech and language, behavioural and neuropsychological, visual and hearing outcomes and seizures.

A search strategy incorporating 99 key terms and mesh headings was developed in Medline Ovid, adapted and run across 10 databases. Snowballing techniques were used to augment search sensitivity (Supplement 1 & 2). All titles were screened independently by two reviewers. The full-texts of all potentially relevant titles were retrieved, reviewed and their risk of bias assessed by two trained reviewers independently (PR, CC, MV, JD, SS). Disagreements were arbitrated by a third reviewer.

Data extraction and synthesis

Studies were stratified by brain injury type, sub-stratified by age of outcome assessment and outcome type, and summarised in a narrative synthesis. Where sufficient suitable data were available from contextually and clinically comparable studies, data were pooled in random

Confidential: For Review Only

effects meta-analyses using RevMan 5.4. Continuous data were pooled using the inverse variance method; dichotomous data were pooled using the Mantel-Haenszel method; and analysis data from studies which did not provide raw data were pooled with dichotomous data from other studies using the generic inverse variance method.(13) Where studies provided insufficient comparative data for a particular outcome, the combined incidence figures for that outcome within the brain injured population was calculated across studies using the Fisher exact test for binomial data.(14) Statistical heterogeneity was assessed using the I^2 statistic and substantial heterogeneity (>85%) was explored further in sub-group analyses.

Quality assessment

The Newcastle Ottawa Tool was used to assess risk of bias across three domains: population selection, the comparability of the 'brain injured' and 'non brain injured' comparator groups, and outcome assessment.(15) Studies were classed as poor, fair, or good for each domain and given an overall risk of bias classification.

Patient and Public Involvement

Patients or the public were not involved in the design or conduct of this review. However the review's findings will be used to shape the larger CHERuB study in partnership with our parent advisory panel.

Confidential: For Review Only

Confidential: For Review Only

RESULTS

Searches identified 14,210 records and 42 studies were included (Figure 1). Studies focused on intraventricular haemorrhage (n=27), white matter injury (WMI) amongst preterm infants (n=15), perinatal stroke (n=8), neonatal meningitis (n=4), and HIE (n=3); these were not mutually exclusive (Supplement 3). Most studies were undertaken in the USA (n=10), the UK (n=8), the Netherlands (n=5) or Australia (n=4). These were prospective (n=27) or retrospective cohort studies (n=14). Included studies were deemed to be moderate (n=17) or low risk of bias (n=27) (Supplement 4).

Preterm injuries

The 29 studies exploring outcomes after IVH or WMI mostly included infants born <32 weeks' gestation (n=22) after the year 2000 (n=18) (Supplement 3). Most studies confirmed injury on ultrasound or MRI imaging (n=22) these were reviewed by radiologists (n=6), neonatologists (n=3) or both (n=1); 14 studies used the Papile classification; only 2 studies stratified results by laterality.

Nine studies explored neurodevelopmental impairment at 5-14 years of age after preterm brain injury including IVH (n=9) and WMI (n=6).(16-24) Two comparable studies highlighted a considerably increased pooled crude risk of moderate-severe neurodevelopmental impairment after IVH grade 3-4 at 8 years of age OR 3.69 (95%CI: 1.7, 7.98; 2 studies) I^2 = 0% (Figure 2, Table 2).(18, 21)

Six studies explored motor outcomes after IVH grade 3-4: they consistently highlighted an increased risk of motor impairment at 5-12 years of age.(21, 24-28) Additionally, two

comparable studies reported an 8-fold increased crude risk of cerebral palsy after IVH grade 3-4 OR 8.13 (95%CI: 4.64, 14.22; 2 studies; 1,557 subjects) I^2=0% (Figure 3).

Cognitive outcomes at school-age after preterm brain injuries were reported by 16 studies using 25 different cognitive assessment tools - limiting the potential for meta-analysis (Supplement 3).(16, 17, 21, 22, 24-35) Educational outcomes were reported by 5 studies.(21, 22, 26, 30, 35)

Studies consistently reported lower cognitive scores at school-age following IVH grade 3-4.(16, 21, 22, 25-27, 31, 35) Hollebrandse 2021 reported an increased risk of cognitive impairment at 8 years of age OR 2.68 (95%CI: 1.21, 5.94).(26) Van de Bor 2000 and Hollebrandse 2021 reported that the cognitive impact of IVH grade 3-4 affected educational needs.(22, 26) Van de Bor 2000 reported increased special educational needs at 5, 9 and 14 years: the adjusted risk at 14 years of age was marked, aOR 3.99 (95%CI: 1.36, 11.69).(22) Studies reported no significant differences in language scores after IVH grade 3-4.(21, 22) However, an association with reading OR 3.62 (95%CI: 1.59, 8.24), spelling OR 4.48 (95%CI: 1.8, 11.2), and arithmetic OR 2.79 (95%CI: 1.2, 6.48) impairment was demonstrated.(26) Most studies highlighted cognitive effects after WMI.(17, 30, 33, 35)

Studies exploring behavioural outcomes after IVH 3-4 did not find any associations with attention deficits, conduct issues or autism spectrum disorder (Table 2).(16, 25, 36) However, there was conflicting evidence around the mental health effects of WMI.(17, 37)

Confidential: For Review Only

Studies exploring hearing impairment after IVH and/or WMI were small or not comparable. 10 studies explored visual impairment after IVH or WMI, 4 provided meaningful outcome data.(16, 21-23, 27, 28, 33, 34, 38, 39) An increased prevalence of visual impairment after IVH grade 3-4 (45.4% and 90.9%) compared to controls (7.5%) was reported in addition to significantly lower visual motor integration scores.(27)

Perinatal stroke

Eight comparative studies explored school-age outcomes after perinatal stroke, these included 177 children with perinatal stroke (100 left-sided and 54 right-sided – not all studies specified laterality) and 232 comparator children (Supplement 3).(40-47) Infants' gestation age was largely unspecified. Five studies presented a combined incidence of childhood seizures after perinatal stroke of 40.1% (95%CI: 26.8-53.3%; 5 studies; 115 subjects) I^2=56% (Supplement 5).(40, 43, 44, 46, 47) The combined incidence of hemiparesis after perinatal stroke was 61% (95%CI: 39.2, 82.9 I^2=88%). There was considerable heterogeneity across studies, and likely detection bias (Supplement 6).(40, 42-45)

Five studies identified a significant combined mean difference in full scale IQ scores at 7-13 years of age after perinatal stroke: -24.2 (95%CI: -30.73, -17.67; 5 studies; 296 subjects) I^2=80% (Figure 4).(40, 42, 45-47) There was heterogeneity across studies in terms of assessment timing, assessment tools, and combining those with left and right-sided strokes.

Differences in stroke laterality partially explained the heterogeneity. The combined mean difference in full scale IQ following left-sided strokes was -26.01 (95%CI: -29.1, -22.93; 2 studies; 113 subjects) I^2=0%; compared to -26.7 (95%CI: -39.38. -14.02; 2 studies; 99

Confidential: For Review Only

subjects) I^2=76% for right-sided strokes. No significant differences in cognitive outcomes were found by laterality.(40, 42, 45-47)

Kolk 2011 reported significantly lower scores across all NEPSY domains other than executive function after perinatal stroke, including attention, visuo-spacial function, memory, and learning.(43)

Two studies presented educational outcomes after perinatal stroke. Although Northam 2018 found that most children with perinatal stroke were in mainstream education (n=28, 93%), they also highlighted that additional educational support was often required (n=12, 40%). This was in keeping with Ballantyne 2008 reporting lower mean scores for reading (85 (16.1) vs. 113 (13.3); p<0.0001), spelling (82.5 (18.2) vs. 106.2 (15.9) p=0.001) and arithmetic (91.5 (10.2) vs. 111.9 (11.2) p<0.0001) after perinatal stroke compared to controls at 7-8 years of age, persisting on re-assessment at 10-12 years.

Kolk 2011 reported significantly lower scores compared to controls across most NEPSY language domains following perinatal stroke.(43) Significantly lower receptive and expressive mean language scores on the CELF assessment were also reported across studies: -20.88 (95%CI: -36.66, -5.11; 2 studies; 137 subjects) I^2=88% and -20.25 (95%CI: -34.36, -6.13; 2 studies; 137 subjects) I^2=87% respectively (Supplement 7, 8).(40, 45) Statistical heterogeneity may have been as a result of studies combining left and right-sided strokes and the varying age of outcome assessment. Studies highlighted that deficits in receptive language scores present at 7-8 years persisted at 10-12 years but that expressive language scores improved (p=0.012).(40, 41)

Meningitis

https://mc.manuscriptcentral.com/bmjpo
Confidential: For Review Only

Studies consistently reported an increased risk of neurodevelopmental impairment after neonatal meningitis (Table 2).(48-50) An increased likelihood of neuromotor disability at 5 years of age (n=45/274, 16%) compared to controls (n=2/1391, 0.1%) was reported (Supplement 3).(48) On re-assessment of the same population at 9-10 years, this increased risk of severe disability persisted (n=12, 10.8% compared to n=0, 0%).(50) An increased risk of any neurodevelopmental impairment at 5 years after neonatal *Group-B Streptococcal* meningitis was also reported in the Netherlands, RR 5.30 (95%CI: 2·57-10·89), and in Denmark, RR 7.80 (95%CI: 4·42-13·77).(49) This increased risk persisted on subsequent assessment: at 11 years of age in the Netherlands, RR 2.99 (95%CI: 1.83, 4.88) and at 15 years of age in Denmark RR, 3.15 (95%CI: 1.82, 5,46).(49)

Hypoxic-ischaemic encephalopathy

Two comparative studies (of the same cohort) explored outcomes of term-born infants with moderate-severe HIE, but without cerebral palsy, at school age (Supplement 3).(51, 52) They highlighted significantly lower full scale IQ scores after HIE (mean difference −13.62 (95%CI: −20.53 to −6.71)).(51) This difference in cognition was also seen for perceptual reasoning, working memory, and processing speed. Children with HIE were also more likely than controls to receive additional classroom support: OR 10 (95%CI: 1.16, 86) although the confidence interval for this risk estimate was wide.(51) Children with HIE (without cerebral palsy) also had significantly lower motor scores (mean difference −2.12 (95%CI: −3.93, −0.30)) and verbal comprehension scores (mean difference −8.8 (95%CI: −14.25, −3.34)).(51) They were also noted to have higher behavioural difficulty scores especially for emotional problems.(51)

Confidential: For Review Only

DISCUSSION

This review brings together the existing evidence on the later childhood outcomes of infants with perinatal brain injury. Although 42 studies are included, small study populations, limited data on injury severity and laterality, and the heterogeneity of outcome measures limited the potential power of results. However, studies demonstrate a three-fold higher risk of moderate-severe neurodevelopmental impairment at school age following IVH grade 3-4. Studies consistently report cognitive impairment after IVH grade 3-4 but suggest that speech and language is relatively preserved. A higher risk of hemiplegia, cognitive impairment and poorer academic performance after perinatal stroke is reported in addition to poorer receptive and expressive language scores. Studies report a higher risk of persisting neurodevelopmental impairment after neonatal meningitis – however few studies address this question. Few comparative studies explore school-age outcomes after HIE.

In following our a priori protocol only comparative studies were included. This was with a view to enabling inferential analyses and adjustment for key confounders such as gestation. Unfortunately due to this strict inclusion criterion many pertinent non-comparative studies were excluded. Additionally our searches were conducted in September 2021, more recent studies would therefore have been missed.

Heterogeneity in terms of outcomes assessed, outcome assessment tools, and timing of outcome assessment limited the comparability of studies and the potential for meta-analyses. Several meta-analyses included low numbers of studies, reducing the reliability of the I^2 statistic.(53) This review was also limited by the size of available studies and how studies presented data for extraction. Few studies presented adjusted data or explored childhood trajectories after perinatal brain injury.

Confidential: For Review Only

Previous reviews were limited by a lack of comparable studies, heterogeneity, the inclusion of much older cohorts, or by including non-comparative studies.(4, 54-56) Whilst this review was also limited by studies' heterogeneity and the quality of available data, new and important findings - for example the risk of neurodevelopmental impairment - at school age after IVH 3-4 were identified. Our finding of a higher risk of cerebral palsy after IVH and motor impairments after preterm brain injuries is echoed by previous studies.(54, 55, 57)

Lynch 2001 highlighted that 60% of infants have neurological sequelae that emerge over time following perinatal stroke. This was in-keeping with our findings of a higher risk of hemiparesis, cognitive impairment, and speech and language impairment.(58) Several non-comparative population-based studies also mirror these findings.(59-62)

Although previous reviews highlight an increased risk of various neurodevelopmental impairments after neonatal meningitis in early childhood – we are unaware of any focusing on school-age outcomes after neonatal meningitis.(4, 63)

The review's findings of potential on-going impairments across cognitive, speech and language, and behavioural domains - in addition to a need for increased school support – after HIE are mirrored by other studies.(64-68) Shankaran 2012 and Azzopardi 2014 highlight on-going neurodevelopmental sequelae at school age amongst children who received therapeutic hypothermia for moderate-severe HIE.(64, 65, 67)

Implications

Confidential: For Review Only

Considerable gaps in the evidence are highlighted, particularly around the risk of specific outcomes following different types of injury, the precision around risk estimates, the impact of different factors (such as injury laterality), and the developmental trajectories of these children. This information is key to prepare families for the future, inform enhanced developmental surveillance, and enable targeted multidisciplinary support to help affected children to reach their full potential. As such, this review highlights a pressing need for high-quality, comparative studies which use the 'Core Outcomes In Neonatology' to explore long-term outcomes after perinatal brain injury and permit future meta-analyses.(10) Additionally, to meet the DHSC ambition to reduce perinatal brain injury, real-time longitudinal population data, extending beyond the neonatal period to childhood, are necessary as the current definition is limited to 'indicators' of injury from the neonatal period. This could be achieved through linkage of existing population datasets within the UK.

CONCLUSION

This review provides an overview of existing evidence of the impact of perinatal brain throughout childhood. Studies' heterogeneity significantly limited the potential for evidence synthesis.

Confidential: For Review Only

Funding/support: This review was supported by an NIHR Doctoral Fellowship award (NIHR301457)

Role of funder/sponsor (if any): The NIHR had no role in the design or conduct of the review.

Conflict of interest disclosures (includes financial disclosures): CG is funded by the United Kingdom Medical Research Council (MRC) through a Transition Support Award. In the past 5 years He has received support from Chiesi Pharmaceuticals to attend an educational conference, and has been investigator on received research grants from Medical Research Council, National Institute of Health Research, Canadian Institute of Health Research, Department of Health in England, Mason Medical Research Foundation, Westminster Medical School Research Trust and Chiesi Pharmaceuticals. CB is funded by the United Kingdom National Institute of Health Research (NIHR) Advanced Fellowship Award.

Confidential: For Review Only

Contributors' statement

Dr Rees conceptualised and designed the review, reviewed and appraised studies, undertook data extraction and synthesis, drafted the initial manuscript, and reviewed and revised the manuscript.

Dr Callan conceptualized and designed the review, designed and oversaw the search strategy, reviewed and appraised studies, undertook data extraction, and reviewed and revised the manuscript.

Dr Chadda reviewed and appraised studies, undertook data extraction, and reviewed and revised the manuscript.

Dr Vaal reviewed and appraised studies, undertook data extraction, and reviewed and revised the manuscript.

Dr Diviney reviewed and appraised studies, undertook data extraction, and reviewed and revised the manuscript.

Dr Sabti reviewed and appraised studies, undertook data extraction, and reviewed and revised the manuscript.

Dr Harnden reviewed and appraised studies, undertook data extraction, and reviewed and revised the manuscript.

Dr Gardiner was the lead statistician for the review, he advised on and oversaw the data analysis, and reviewed and revised the manuscript.

Dr Battersby oversaw and supervised the review and critically revised the manuscript for important intellectual content.

Professor Gale oversaw and supervised the review and critically revised the manuscript for important intellectual content.
Professor Sutcliffe oversaw and supervised the review and critically revised the manuscript for important intellectual content.
All authors approve the final manuscript as submitted and agree to be accountable for all aspects of the work.

Additional Contributions: The authors would like to thank Dr Roxanna Short for creating the figures in supplemental file 5 and 6.

Confidential: For Review Only

Figure 1: PRISMA flow diagram

Figure 2: Crude risk of neurodevelopmental impairment at 8 years of age after IVH grade 3-4

Figure 3: Crude risk of cerebral palsy after IVH grade 3-4

Figure 4: Pooled mean difference in IQ scores at 7-13 years between those with and without perinatal stroke

Confidential: For Review Only

References

1. Lawn JE, Blencowe H, Oza S, You D, Lee AC, Waiswa P, et al. Every Newborn: progress, priorities, and potential beyond survival. Lancet. 2014;384(9938):189-205.
2. Lee AC, Kozuki N, Blencowe H, Vos T, Bahalim A, Darmstadt GL, et al. Intrapartum-related neonatal encephalopathy incidence and impairment at regional and global levels for 2010 with trends from 1990. Pediatric Research. 2013;74(S1):50-72.
3. Liu L, Oza S, Hogan D, Chu Y, Perin J, Zhu J, et al. Global, regional, and national causes of under-5 mortality in 2000–15: an updated systematic analysis with implications for the Sustainable Development Goals. Lancet. 2016;388(10063):3027-35.
4. Mwaniki MK, Atieno M, Lawn JE, Newton CR. Long-term neurodevelopmental outcomes after intrauterine and neonatal insults: a systematic review. Lancet. 2012;379(9814):445-52.
5. Department of Health & Social Care. New ambition to halve rate of stillbirths and infant deaths 2015 [Available from: https://www.gov.uk/government/news/new-ambition-to-halve-rate-of-stillbirths-and-infant-deaths.
6. Gale C, Statnikov Y, Jawad S, Uthaya SN, Modi N, Brain Injuries expert working group. Neonatal brain injuries in England: population-based incidence derived from routinely recorded clinical data held in the National Neonatal Research Database. Archives of Disease in Childhood - Fetal and Neonatal Edition. 2018;103(4):F301-F6.
7. Marlow N. Measuring neurodevelopmental outcome in neonatal trials: a continuing and increasing challenge. Archives of Disease in Childhood-Fetal and Neonatal Edition. 2013;98(6):F554-F8.
8. Marlow N, Wolke D, Bracewell MA, Samara M. Neurologic and developmental disability at six years of age after extremely preterm birth. New England Journal of Medicine. 2005;352(1):9-19.
9. Webbe J, Brunton G, Ali S, Longford N, Modi N, Gale C. Parent, patient and clinician perceptions of outcomes during and following neonatal care: a systematic review of qualitative research. BMJ Paediatrics Open. 2018;2(1).
10. Webbe JWH, Duffy JMN, Afonso E, Al-Muzaffar I, Brunton G, Greenough A, et al. Core outcomes in neonatology: development of a core outcome set for neonatal research. Archives of Disease in Childhood-Fetal and Neonatal Edition. 2019.
11. Page MJ, McKenzie JE, Bossuyt PM, Boutron I, Hoffmann TC, Mulrow CD, et al. The PRISMA 2020 statement: an updated guideline for reporting systematic reviews. Systematic Reviews. 2021;10(1):1-11.
12. Gale C, Stanikov E, Jawad S, Uthaya S, Modi N. Brain injury occurring during or soon after birth: a report for the national maternity ambition commissioned by the Department of Health. Imperial College london; 2017.
13. Higgins JPT, Thomas J, Chandler J, Cumpston M, Li T, Page MJ, et al. Cochrane handbook for systematic reviews of interventions version 6.2 (updated February 2021): Cochrane; 2021. Available from: www.training.cochrane.org/handbook.
14. Mehanna H, Al-Maqbili T, Carter B, Martin E, Campain N, Watkinson J, et al. Differences in the recurrence and mortality outcomes rates of incidental and nonincidental papillary thyroid microcarcinoma: a systematic review and meta-analysis of 21 329 person-years of follow-up. Journal of Clinical Endocrinology and Metabolism. 2014;99(8):2834-43.
15. Wells GA, Shea B, O'Connell D, Peterson J, Welch V, Losos M, et al. The Newcastle-Ottawa Scale (NOS) for assessing the quality of nonrandomised studies in meta-analyses. Oxford; 2000.

Confidential: For Review Only

16. Adant I, Miserez M, Naulaers G, Carkeek K, Ortibus E, Aerts R, et al. Long-term outcomes of very low birth weight infants with spontaneous intestinal perforation: A retrospective case-matched cohort study. Journal of Pediatric Surgery. 2019;54(10):2084-91.
17. Campbell H, Check J, Kuban KC, Leviton A, Joseph RM, Frazier JA, et al. Neonatal cranial ultrasound findings among infants born extremely preterm: associations with neurodevelopmental outcomes at ten years of age. The Journal of Pediatrics. 2021.
18. Cheong JL, Lee KJ, Boland RA, Spittle AJ, Opie GF, Burnett AC, et al. Changes in long-term prognosis with increasing postnatal survival and the occurrence of postnatal morbidities in extremely preterm infants offered intensive care: a prospective observational study. The Lancet Child & Adolescent Health. 2018;2(12):872-9.
19. Neubauer A-P, Voss W, Kattner E. Outcome of extremely low birth weight survivors at school age: the influence of perinatal parameters on neurodevelopment. European Journal of Pediatrics. 2008;167(1):87-95.
20. Piris Borregas S, Torres Valdivieso MJ, Martín-Arriscado C, de la Cruz Bértolo J, Sierra García P, Pallás Alonso CR. Model that predicted death or disabilities in premature infants was valid at seven years of age. Acta Paediatrica. 2019;108(7):1245-9.
21. Sherlock RL, Anderson PJ, Doyle LW, Group VICS. Neurodevelopmental sequelae of intraventricular haemorrhage at 8 years of age in a regional cohort of ELBW/very preterm infants. Early Human Development. 2005;81(11):909-16.
22. Van de Bor M, den Ouden L. School performance in adolescents with and without periventricular-intraventricular hemorrhage in the neonatal period. Seminars in Perinatology. 2004;28(4):295-303.
23. Vollmer B, Roth S, Baudin J, Stewart AL, Neville BG, Wyatt JS. Predictors of long-term outcome in very preterm infants: gestational age versus neonatal cranial ultrasound. Pediatrics. 2003;112(5):1108-14.
24. Hintz SR, Vohr BR, Bann CM, Taylor HG, Das A, Gustafson KE, et al. Preterm neuroimaging and school-age cognitive outcomes. Pediatrics. 2018;142(1).
25. Brouwer A, Van Stam C, Venema MU, Koopman C, Groenendaal F, De Vries L. Cognitive and neurological outcome at the age of 5–8 years of preterm infants with post-hemorrhagic ventricular dilatation requiring neurosurgical intervention. Neonatology. 2012;101(3):210-6.
26. Hollebrandse NL, Spittle AJ, Burnett AC, Anderson PJ, Roberts G, Doyle LW, et al. School-age outcomes following intraventricular haemorrhage in infants born extremely preterm. Archives of Disease in Childhood-Fetal and Neonatal Edition. 2021;106(1):4-8.
27. Klebermass-Schrehof K, Czaba C, Olischar M, Fuiko R, Waldhoer T, Rona Z, et al. Impact of low-grade intraventricular hemorrhage on long-term neurodevelopmental outcome in preterm infants. Child's Nervous System. 2012;28(12):2085-92.
28. Vollmer B, Roth S, Riley K, O'Brien F, Baudin J, De Haan M, et al. Long-term neurodevelopmental outcome of preterm children with unilateral cerebral lesions diagnosed by neonatal ultrasound. Early Human Development. 2006;82(10):655-61.
29. Hirvonen M, Ojala R, Korhonen P, Haataja P, Eriksson K, Rantanen K, et al. Intellectual disability in children aged less than seven years born moderately and late preterm compared with very preterm and term-born children–a nationwide birth cohort study. Journal of Intellectual Disability Research. 2017;61(11):1034-54.
30. Jansen L, Peeters-Scholte C, Wiggers-de Bruine S, van den Berg-Huysmans A, van Klink J, van Steenis A, et al. Classroom-evaluated school performance at nine years of age after very preterm birth. Early Human Development. 2020;140:104834.
31. Koç Ö, Kavuncuoğlu S, Ramoğlu MG, Aldemir E, Aktalay A, Eras Z. School performance and neurodevelopment of very low birth weight preterm infants: first report from Turkey. Journal of Child Neurology. 2016;31(2):170-6.

Confidential: For Review Only

32. Pittet M-P, Mürner-Lavanchy I, Adams M, Bickle-Graz M, Pfister R, Natalucci G, et al. Neurodevelopmental outcome at early school age in a Swiss national cohort of very preterm children. Swiss Medical Weekly. 2019;149:w20084.
33. Van den Hout B, Stiers P, Haers M, van der Schouw YT, Eken P, Vandenbussche E, et al. Relation between visual perceptual impairment and neonatal ultrasound diagnosis of haemorrhagic–ischaemic brain lesions in 5-year-old children. Developmental Medicine and Child Neurology. 2000;42(6):376-86.
34. Vollmer B, Roth S, Riley K, Sellwood MW, Baudin J, Neville BG, et al. Neurodevelopmental outcome of preterm infants with ventricular dilatation with and without associated haemorrhage. Developmental Medicine and Child Neurology. 2006;48(5):348-52.
35. Kiechl-Kohlendorfer U, Ralser E, Pupp Peglow U, Pehboeck-Walser N, Fussenegger B. Early risk predictors for impaired numerical skills in 5-year-old children born before 32 weeks of gestation. Acta Paediatrica. 2013;102(1):66-71.
36. Davidovitch M, Kuint J, Lerner-Geva L, Zaslavsky-Paltiel I, Rotem RS, Chodick G, et al. Postnatal steroid therapy is associated with autism spectrum disorder in children and adolescents of very low birth weight infants. Pediatric Research. 2020;87(6):1045-51.
37. Whitaker AH, Feldman JF, Lorenz JM, McNicholas F, Fisher PW, Shen S, et al. Neonatal head ultrasound abnormalities in preterm infants and adolescent psychiatric disorders. Archives of General Psychiatry. 2011;68(7):742-52.
38. Hreinsdottir J, Fredriksson Kaul Y, Hellström-Westas L, Rosander K, von Hofsten C, Holmström G. Impaired cognitive ability at 2.5 years predicts later visual and ophthalmological problems in children born very preterm. Acta Paediatrica. 2018;107(5):822-30.
39. Kaur A, Luu TM, Shah PS, Ayoub A, Auger N. Neonatal intraventricular hemorrhage and hospitalization in childhood. Pediatric Neurology. 2020;103:35-42.
40. Ballantyne AO, Spilkin AM, Hesselink J, Trauner DA. Plasticity in the developing brain: intellectual, language and academic functions in children with ischaemic perinatal stroke. Brain. 2008;131(11):2975-85.
41. Ballantyne AO, Spilkin AM, Trauner DA. Language outcome after perinatal stroke: does side matter? Child Neuropsychology. 2007;13(6):494-509.
42. Gold JJ, Trauner DA. Hippocampal volume and memory performance in children with perinatal stroke. Pediatric Neurology. 2014;50(1):18-25.
43. Kolk A, Ennok M, Laugesaar R, Kaldoja M-L, Talvik T. Long-term cognitive outcomes after pediatric stroke. Pediatric Neurology. 2011;44(2):101-9.
44. Martin K, Trauner DA. Auditory neglect in children following perinatal stroke. Behavioural Brain Research. 2019;359:878-85.
45. Northam GB, Adler S, Eschmann KC, Chong WK, Cowan FM, Baldeweg T. Developmental conduction aphasia after neonatal stroke. Annals of Neurology. 2018;83(4):664-75.
46. Tillema J-M, Byars AW, Jacola LM, Schapiro MB, Schmithorst VJ, Szaflarski JP, et al. Reprint of "Cortical reorganization of language functioning following perinatal left MCA stroke"[Brain and Language 105 (2008) 99–111]. Brain and Language. 2008;106(3):184-94.
47. Trauner DA, Nass R, Ballantyne A. Behavioural profiles of children and adolescents after pre-or perinatal unilateral brain damage. Brain. 2001;124(5):995-1002.
48. Bedford H, de Louvois J, Halket S, Peckham C, Hurley R, Harvey D. Meningitis in infancy in England and Wales: follow up at age 5 years. BMJ. 2001;323(7312):533.
49. Horváth-Puhó E, van Kassel MN, Gonçalves BP, de Gier B, Procter SR, Paul P, et al. Mortality, neurodevelopmental impairments, and economic outcomes after invasive group B streptococcal disease in early infancy in Denmark and the Netherlands: a national matched cohort study. The Lancet Child & Adolescent Health. 2021;5(6):398-407.

Confidential: For Review Only

50. Stevens J, Eames M, Kent A, Halket S, Holt D, Harvey D. Long term outcome of neonatal meningitis. Archives of Disease in Childhood-Fetal and Neonatal Edition. 2003;88(3):F179-F84.
51. Lee-Kelland R, Jary S, Tonks J, Cowan FM, Thoresen M, Chakkarapani E. School-age outcomes of children without cerebral palsy cooled for neonatal hypoxic–ischaemic encephalopathy in 2008–2010. Archives of Disease in Childhood-Fetal and Neonatal Edition. 2020;105(1):8-13.
52. Tonks J, Cloke G, Lee-Kelland R, Jary S, Thoresen M, Cowan FM, et al. Attention and visuo-spatial function in children without cerebral palsy who were cooled for neonatal encephalopathy: a case-control study. Brain Injury. 2019;33(7):894-8.
53. von Hippel PT. The heterogeneity statistic I2 can be biased in small meta-analyses. BMC medical research methodology. 2015;15(1):1-8.
54. Gotardo JW, Volkmer NdFV, Stangler GP, Dornelles AD, Bohrer BBdA, Carvalho CG. Impact of peri-intraventricular haemorrhage and periventricular leukomalacia in the neurodevelopment of preterms: A systematic review and meta-analysis. PloS one. 2019;14(10):e0223427.
55. Mukerji A, Shah V, Shah PS. Periventricular/intraventricular hemorrhage and neurodevelopmental outcomes: a meta-analysis. Pediatrics. 2015;136(6):1132-43.
56. Magai DN, Karyotaki E, Mutua AM, Chongwo E, Nasambu C, Ssewanyana D, et al. Long-term outcomes of survivors of neonatal insults: A systematic review and meta-analysis. PloS one. 2020;15(4):e0231947.
57. Rees P, Callan C, Chadda KR, Vaal M, Diviney J, Sabti S, et al. Preterm brain injury and neurodevelopmental outcomes: a meta-analysis. Pediatrics. 2022;Nov 4:e2022057442.
58. Lynch JK, Nelson KB. Epidemiology of perinatal stroke. Current Opinion in Pediatrics. 2001;13(6):499-505.
59. Lee J, Croen LA, Lindan C, Nash KB, Yoshida CK, Ferriero DM, et al. Predictors of outcome in perinatal arterial stroke: a population-based study. Annals of Neurology. 2005;58(2):303-8.
60. Grunt S, Mazenauer L, Buerki SE, Boltshauser E, Mori AC, Datta AN, et al. Incidence and outcomes of symptomatic neonatal arterial ischemic stroke. Pediatrics. 2015;135(5):e1220-e8.
61. Husson B, Hertz-Pannier L, Renaud C, Allard D, Presles E, Landrieu P, et al. Motor outcomes after neonatal arterial ischemic stroke related to early MRI data in a prospective study. Pediatrics. 2010;126(4):e912-e8.
62. Wusthoff CJ, Kessler SK, Vossough A, Ichord R, Zelonis S, Halperin A, et al. Risk of later seizure after perinatal arterial ischemic stroke: a prospective cohort study. Pediatrics. 2011;127(6):e1550-e7.
63. Kohli-Lynch M, Russell NJ, Seale AC, Dangor Z, Tann CJ, Baker CJ, et al. Neurodevelopmental impairment in children after group B streptococcal disease worldwide: systematic review and meta-analyses. Clinical Infectious Diseases. 2017;65(suppl_2):S190-S9.
64. Shankaran S, Pappas A, McDonald SA, Vohr BR, Hintz SR, Yolton K, et al. Childhood outcomes after hypothermia for neonatal encephalopathy. New England Journal of Medicine. 2012;366(22):2085-92.
65. Azzopardi D, Strohm B, Marlow N, Brocklehurst P, Deierl A, Eddama O, et al. Effects of hypothermia for perinatal asphyxia on childhood outcomes. New England Journal of Medicine. 2014;371(2):140-9.
66. Jary S, Lee-Kelland R, Tonks J, Cowan FM, Thoresen M, Chakkarapani E. Motor performance and cognitive correlates in children cooled for neonatal encephalopathy without cerebral palsy at school age. Acta Paediatrica. 2019;108(10):1773-80.

Confidential: For Review Only

67. Natarajan G, Shankaran S, Pappas A, Bann C, Tyson JE, McDonald S, et al. Functional status at 18 months of age as a predictor of childhood disability after neonatal hypoxic-ischemic encephalopathy. Developmental Medicine & Child Neurology. 2014;56(11):1052-8.
68. Guillet R, Edwards AD, Thoresen M, Ferriero DM, Gluckman PD, Whitelaw A, et al. Seven-to eight-year follow-up of the CoolCap trial of head cooling for neonatal encephalopathy. Pediatric Research. 2012;71(2):205-9.

Confidential: For Review Only

Table 1 Inclusion and exclusion criteria

Inclusion Criteria	Exclusion Criteria
Peer-reviewed observational studies (cohort, case-control, cross-sectional)	Non-comparative studies; opinions; commentaries; reviews; case-reports; lab studies
Studies in all languages	Studies where the population includes adults and children and the data for children cannot be extracted
Studies published after 2000	Studies focused on children with IVH grade 1-2, neonatal seizures, hypoglycaemic brain injury, or neonatal abstinence syndrome
Children with a diagnosis of brain injury occurring at or around the time of birth (including during the neonatal period) as defined by the DHSC (including those with any white matter injury but not including those with isolated seizures)	Studies which include infants with brain injuries diagnosed during the neonatal and infancy period where most were diagnosed outside of the neonatal period
Studies including infants with moderate to severe HIE born in the post therapeutic hypothermia era (i.e. where infants received therapeutic hypothermia)	Studies including infants with moderate-severe HIE born during the pre-therapeutic hypothermia era or in low- or middle-income countries that do not offer therapeutic hypothermia
Studies focused on school-aged neurodevelopmental outcomes (of children between 5-18 years of age) including:<br>**Primary outcome(s):**<br>Neurodevelopmental impairment, as defined by authors (including direct testing, clinical record review, and parental interview/ survey)<br><br>**Secondary outcome(s):**<br>1. Any cognitive impairment, as defined by authors (direct testing)<br><br>2. Mild cognitive impairment (intelligence or developmental quotient 1-2 standard deviations below the mean)<br><br>3. Moderate-severe cognitive impairment (intelligence or developmental quotient more than 2 standard deviations below the mean)<br><br>4 Executive dysfunction, as defined by authors (direct testing)<br><br>5. Low numeracy, as defined by authors (by direct testing or educational achievement tests)<br><br>6. Low literacy, as defined by authors (by direct testing or educational achievement tests)<br><br>7. Special educational needs as defined by authors (school or parental report)<br><br>8. Motor impairment, as defined by authors (including direct testing, clinical record review, and reporting)<br><br>9. Visual-motor impairment, as defined by authors (on direct testing)	Studies of infants with mild HIE

Confidential: For Review Only

10. Emotional-behavioural difficulty, as defined by authors (including direct testing, clinical record review, and parental reporting<br><br>11. Speech and language impairment, as defined by authors (on direct testing)<br><br>12. Visual impairment, as defined by authors (including direct testing, clinical record review, and parental reporting)<br><br>13. Hearing impairment, as defined by authors (including direct testing, clinical record review, and parental reporting)<br><br>14. Epilepsy/seizures, as defined by authors (including medical history taking, clinical record review and parental reporting	
	Studies reporting outcomes for children diagnosed with brain injury beyond the neonatal period
	Studies where comparable outcome data from those with and without perinatal brain injury cannot be extracted

Confidential: For Review Only

Table 2: Overview of key findings for school-age outcomes of infants with perinatal brain injury compared to those without brain injury
(*Does not include studies where infants with IVH grade 3-4 cannot be separated from those with WMI or those with IVH 1-2)
(# Does not include studies using hearing or visual outcomes only as part of their composite outcome)
Adjusted Odds Ratio (aOR); Attention Deficit Hyperactivity Disorder (ADHD); Autism Spectrum Disorder (ASD); Confidence Interval (CI); cystic periventricular leukomalacia (cPVL); Group B Streptococcus (GBS); Hypoxic-Ischaemic Encephalopathy (HIE); Hazard Ratio (HR); Intelligence Quotient (IQ); Interquartile range (IQR); Intraventricular Haemorrhage (IVH); Odds Ratio (OR); Periventricular Leukomalacia (PVL); Visual Motor Integration (VMI); White Matter Injury (WMI)

	NDI	Cognitive	Motor	Speech and language	Behavioural	Hearing#	Vision#	Other
IVH grade 3-4*	6 studies(15, 17-21)<br><br>2 comparable studies in meta-analysis(17, 20)<br><br>**Meta-analysis** (2 studies): Increased risk of moderate - severe neurodevelopmental impairment OR 3.15 (95%CI: 1.67, 5.92) $I^2 = 0\%$<br><br>**Van de Bor 2004**: increased prevalence of disability 31% vs. 16%	9 studies(15, 20, 21, 24-26, 30, 70)<br><br>Not comparable<br><br>Consistently highlighted lower cognitive scores<br><br>**Brouwer 2012**: significantly lower performance IQ but preserved verbal IQ. Lower IQ for those with IVH grade 4 requiring neurosurgery (91+/-10 vs. 98+/-15) but little difference for those with grade 3 IVH requiring neurosurgery (96+/-15 vs. 98+/-15).<br><br>**Hollebrandse 2021**: increased risk of cognitive impairment OR 2.68 (95%CI: 1.21, 5.94). Increased risk of academic impairment across all academic domains: reading OR 3.62 (95%CI: 1.59, 8.24); spelling OR 4.48 (95%CI: 1.8, 11.2); arithmetic OR 2.79)95%CI: 1.2, 6.48)<br><br>**Sherlock 2005:** significantly lower IQ scores after IVH grade 4 vs. IVH 1-3 and no brain injury, also seen for	6 studies(20, 23-26, 33)<br><br>Not comparable<br><br>All reported increased risk of motor impairment<br><br>**<u>Cerebral palsy</u>**<br>3 comparable studies<br><br>OR 8.67 (95%CI: 5.27, 14.28) I^2=0%.	3 studies(20, 21, 25)<br><br>Not comparable<br><br>**Van de Bor 2004**: no significant difference in language scores<br><br>**Sherlock 2005**: downward trend in language scores from no brain injury to each grade of IVH but not statistically significant p=0.12<br><br>**Hollebrandse 2021**: Increased risk of impaired reading OR 3.62 (95%CI: 1.59, 8.24), and spelling OR 4.48 (95%CI: 1.8, 11.2)	3 studies(15, 24, 35)<br><br>Not comparable<br><br>**Brouwer 2012**: no association with any behavioural domains assessed (internalising, externalising and sleep problems)<br><br>**Adant 2019**: no increased risk of attention deficits, conduct issues or ASD aOR 1.24 (95%CI: 0.32, 4.8).<br><br>**Davidovich 2020**: no increased risk of ASD (n=10, 3.9% vs. n=103, 2.2% p=0.085)	3 studies(21, 26, 38)<br><br>Not comparable<br><br>Outcome too rare for inferential analysis<br><br>**Kaur 2020:** increased risk of hospitalisation for otologic reasons HR 7.87 (95%CI: 5.31, 11.67)	5 studies(15, 21, 26, 33, 38)<br><br>Not comparable<br><br>Outcome to rare for inferential analysis in most studies.<br><br>**Adant 2019**: no increased risk of visual impairment (needing glasses) aOR 0.47 (95%CI: 0.13, 1.69)<br><br>**Klebermass-Schrehof 2012:** increased prevalence of visual impairment (needing glasses or blindness) after IVH grade 3 (45.4%) and IVH grade 4 (90.9%) vs. comparators (7.5%).<br><br>**Kaur 2020:** increased risk of hospitalisation for ophthalmic reasons HR 7.87 (95%CI: 5.31, 11.67).<br><br>**Klebermass-Schrehof 2012:** significantly lower	

Confidential: For Review Only

		several domains: freedom from distractibility, processing speed, reading, spelling and arithmetic. No difference in executive function.<br><br>**Van de Bor 2004**: increased special education needs at 5, 9 and 14 years aOR 3.99 (95%CI: 1.36, 11.69).					VMI scores (67.5 ± 14 vs. 76 ± 26.8; p=0.04)	
WMI*	3 studies(16, 17, 22)<br><br>Not comparable<br><br>**Campbell 2021**: living with no impairment was less common with WMI (n=12, 40%) vs. controls (n=487, 76%)<br><br>**Cheong 2018**: increased risk of survival with major disability after cPVL aOR 9·17 (95%CI: 3·57, 23·53)<br><br>**Vollmer 2003:** Disabling impairments were more common after cPVL at<28 weeks' gestation (n=3, 75% <28	4 studies(16, 29, 32, 70)<br><br>Not comparable<br><br>**Van den Hout 2000**: 50% with PVL had IQ scores <85 vs. 11.8% without injury and a lower performance age 4.3 years vs. 6.2 years<br><br>**Campbell 2021**: increased risk of moderate-severe cognitive impairment aOR 5.07 (95%CI: 2.13, 12.02)<br><br>**Jansen 2020**: WMI predictive of poorer performance on standardised mathematics tests (B 1.856 p=0.003), but not performance on spelling (B 1.076 p=0.075) or reading tests (B 0.241 p=0.483)	**<u>Cerebral palsy</u>**<br>1 study(16)<br><br>**Campbell 2020**: increased risk of cerebral palsy aOR 18.63 (95%CI: 7.37, 47.06)	1 study(29)<br><br>**Jansen 2020**: No association between WMI and spelling (B 1.076 p=0.075) or reading performance (B 0.241 p=0.483)	4 studies(16, 35, 36, 71)<br>Not comparable<br><br>Conflicting results<br><br>**Campbell 2021**: No increased risk of: ADHD (n=3, 10% vs. n=97, 15%); anxiety (n=3, 10% vs. n=98, 15%); depression (n=7, 23% vs. n=100, 16%); or ASD aOR 0.74 (95%CI: 0.09, 5.88)<br><br>**Davidovich 2020**: No increased risk of ASD after PVL (n=5, 2.5% vs. n=88, 2.3% p=0.86)<br><br>**Whitaker 2011:** increased risk of ADHD aOR 6.83 (95%CI: 1.26-36.91); major depression aOR 2.59 (95%CI: 1.02-6.58); tic disorders aOR 9.77 (95%CI: 1.69-56.47); obsessive compulsive disorders aOR 15.32 (95%CI: 1.82-128.74)	0 studies	1 study(32)	

Confidential: For Review Only

	weeks) vs. controls (n=3, 8%) and at over 28 weeks' gestation (n=6,50% vs. n=14, 6%)							
Stroke	0 studies	6 studies(39, 41, 42, 44-46)<br>5 comparable studies in meta-analysis (39, 41, 44-46)<br><br>**Meta-analysis** (5 studies): significant mean difference in full scale IQ: -24.2 (95%CI: -30.73, -17.67) I^2=80%<br><br>**Trauner 2001 and Gold 2014**: no significant difference in full scale IQ scores in left vs. right-sided strokes<br><br>**Ballantyne 2008:** significantly lower performance IQ (p=0.002) and verbal IQ (p<0.0001). Lower mean scores for reading (p<0.0001), spelling (p=0.001) and arithmetic (p<0.0001) at 7-8 years persisting to 10-12 years<br><br>**Tillema 2008:** reduced verbal IQ scores (mean 84 SD 13.4) vs. (mean 108 SD 14.2 P=0.002)<br><br>**Kolk 2011**: poorer attention (across 4 of the 7 assessment sub-domains), visuo-spacial function (across 4 of the 5 sub-domains), and memory and learning (across 4 of the 6 sub-domains), but normal executive function scores.	5 studies(39, 41-44)<br>Combined hemiparesis incidence: 61% (95%CI: 39.2, 82.9 I^2=88%)<br><br>**Kolk 2011:** moderate to severe neuromotor impairment in 62% n=13) and significantly lower scores on 5/6 sensorimotor domains of the NEPSY	5 studies(39, 40, 42, 44, 45)<br><br>3 comparable studies in meta-analysis<br>**Meta-analysis** (3 studies): lower receptive language scores-20.88 (95%CI: -36.66, -5.11) I^2=88% and lower expressive language scores -20.25 (95%CI: -34.36, -6.13) I^2=87%<br><br>**Ballantyne 2007 and Ballantyne 2008**: deficits in receptive language scores at 7-8 years persist at 10-12 years but expressive language scores improved (p=0.012) particularly for children with right-sided strokes (p=0.034)<br><br>**Kolk 2011:** significantly lower scores for 8/9 NEPSY domains including phonologic processing, comprehension of instructions, correct speeded naming, repetition of nonsense words, verbal fluency (semantic and phonetic), oromotor sequences, and sentence comprehension	1 study(46)	1 study(43)<br><br>**Martin 2019:** left-sided strokes predispose children to contralateral auditory neglect and right-sided strokes predispose children to bilateral auditory neglect	1 study(39)<br><br>**Ballantyne 2008:** visual field defects are common (n=7, 26%) after perinatal stroke	<u>**Seizures**</u><br>8 studies(39, 42, 43, 45, 46)<br><br>5 comparable studies(39, 42, 43, 45, 46)<br>Combined incidence of seizures: 40.1% (95%CI: 26.8, 53.3) I^2=56%

Confidential: For Review Only

		Those with left-sided strokes had poorer neuropsychological scores.<br><br>**Northam 2018:** most children are in mainstream education (n=28, 93%) but many require additional support (n=12, 40%)						
Meningitis	3 studies(47-49)<br>Not comparable<br><br>All reported increased risk of neurodevelopmental impairment<br><br>**Bedford 2011:** increased prevalence of neuromotor disability (n=45, 16% vs. n=2, 0.1%)<br><br>**Stevens 2003:** Risk of severe disability seen in Bedford 2011 at 5 years of age persisted until 9-10 years (n=12, 10.8% vs. n=0, 0%)<br><br>**Horvath-Puho 2021:** increased risk of any neurodevelopmental impairment	1 study(49)<br><br>**Stevens 2003:** significantly lower mean cognitive scores (mean 88.8 (95%CI: 85, 92) vs. mean 99.4 (95%CI: 97, 102))	1 study(49)<br><br>**Stevens 2003:** significantly higher motor impairment scores (mean 7.1 (95%CI: 5.9, 8.5) vs. mean 5 (95%CI: 4.3, 5.8))	0 studies	0 studies	2 studies(49, 72)<br><br>**Martinez Cruz 2008:** increased odds of neonatal meningitis amongst preterm infants with sensorineural hearing loss OR 4.37 (95%CI: 1.7, 10.9<br><br>**Stevens 2003:** 3.6% (n=4) had hearing loss compared to none in the control group.	1 study(49)<br><br>**Stevens 2003:** Bilateral visual impairment was common after neonatal meningitis (n=18, 17%)	

Confidential: For Review Only

	after GBS meningitis in the Netherlands RR 5.30 (95%CI: 2·57, 10·89) and Denmark RR 7.80 (95%CI: 4·42, 13·77) at 5 years of age persisting to 11 years in the Netherlands RR 2.99 (95%CI: 1.83, 4.88) and 15 years in Denmark RR 3.15 (95%CI: 1.82, 5,46)							
HIE	0 studies	3 studies(30, 50, 51) (two of the same population)<br><br>Not comparable<br><br>**Koc 2016:** preterm infants with HIE significantly more likely to have below average IQ scores (n=8, 89% vs. n=24, 30% p=0.001)<br><br>**Lee-Kelland 2020 and Tonks 2019:** report lower full scale IQ scores after moderate to severe HIE (mean difference −13.62 (95%CI: −20.53, −6.71)) and poorer perceptual reasoning, working memory and processing speed. Children with previous HIE more likely to receive additional classroom support OR 10 (95%CI: 1.16, 86)	2 studies(50, 51) (of the same population)<br><br>**Lee-Kelland 2020 and Tonks 2019:** significantly lower motor scores (mean difference −2.12 (95%CI: −3.93, −0.30)) after moderate-severe HIE (for children without cerebral palsy)	2 studies(50, 51) (of the same population)<br><br>**Lee-Kelland 2020 and Tonks 2019:** significantly lower verbal comprehension scores (mean difference −8.8 (95%CI: −14.25, −3.34)) after moderate-severe HIE.	2 studies(50, 51) (of the same population)<br><br>**Lee-Kelland 2020 and Tonks 2019:** higher behavioural difficulty scores (median score 12 IQR (6.5, 13.5 vs. median score 6 IQR (2.25, 10) p=0.005)	0 studies	0 studies	
Kernicterus	0 studies							

Confidential: For Review Only

Confidential: For Review Only

PRISMA 2009 Flow Diagram

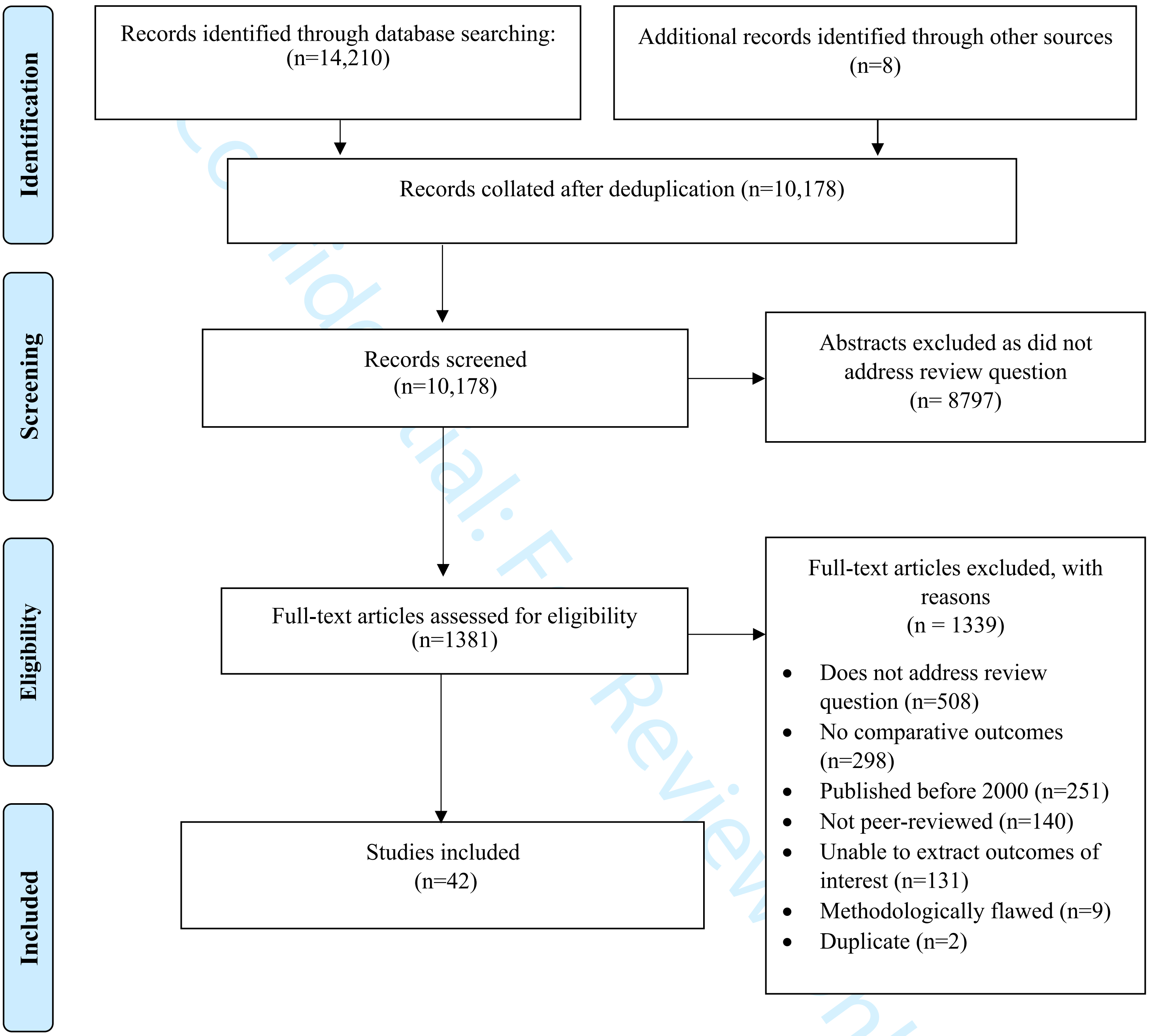

Confidential: For Review Only

Confidential: For Review Only

Confidential: For Review Only

Study or Subgroup	log[Odds Ratio]	SE	Weight	Odds Ratio IV, Random, 95% CI
Cheong 2018	1.0919233	0.40789163	62.4%	2.98 [1.34, 6.63]
Sherlock 2005	1.23969089	0.52602402	37.6%	3.45 [1.23, 9.69]
**Total (95% CI)**			**100.0%**	**3.15 [1.67, 5.92]**

Heterogeneity: Tau² = 0.00; Chi² = 0.05, df = 1 (P = 0.82); I² = 0%
Test for overall effect: Z = 3.56 (P = 0.0004)

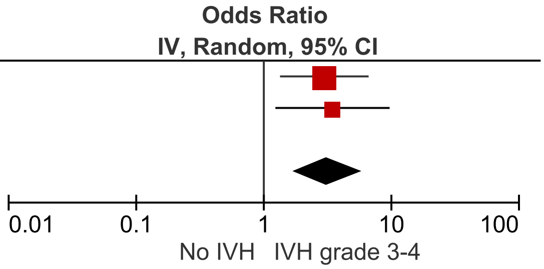

Study or Subgroup	IVH grade 3-4 Events	IVH grade 3-4 Total	No IVH Events	No IVH Total	Weight	Odds Ratio M-H, Random, 95% CI
Beaino 2010	9	38	46	1153	38.5%	7.47 [3.34, 16.69]
Hollebrandse 2021	15	35	26	331	40.9%	8.80 [4.03, 19.19]
Sherlock 2005	8	18	12	179	20.6%	11.13 [3.71, 33.41]
**Total (95% CI)**		**91**		**1663**	**100.0%**	**8.67 [5.27, 14.28]**
Total events	32		84			

Heterogeneity: Tau² = 0.00; Chi² = 0.33, df = 2 (P = 0.85); I² = 0%
Test for overall effect: Z = 8.49 (P < 0.00001)

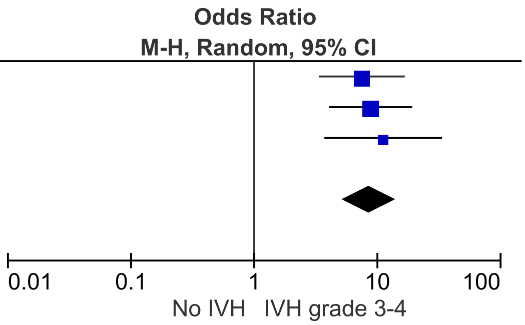

Study or Subgroup	Perinatal stroke Mean	SD	Total	No stroke Mean	SD	Total	Weight	Mean Difference IV, Random, 95% CI
Ballayntyne 2008	94.7	20.4	29	123	15	38	18.2%	-28.30 [-37.12, -19.48]
Gold 2014	88	4	27	117	2.7	19	26.6%	-29.00 [-30.94, -27.06]
Northam 2017	99	14	30	112	16	40	20.7%	-13.00 [-20.05, -5.95]
Tilema 2008	80	14.1	10	108	11.7	10	14.9%	-28.00 [-39.36, -16.64]
Trauner 2001	93.4	22	39	116.2	13	54	19.7%	-22.80 [-30.53, -15.07]
**Total (95% CI)**			**135**			**161**	**100.0%**	**-24.20 [-30.73, -17.67]**

Heterogeneity: Tau² = 40.85; Chi² = 20.00, df = 4 (P = 0.0005); I² = 80%
Test for overall effect: Z = 7.26 (P < 0.00001)

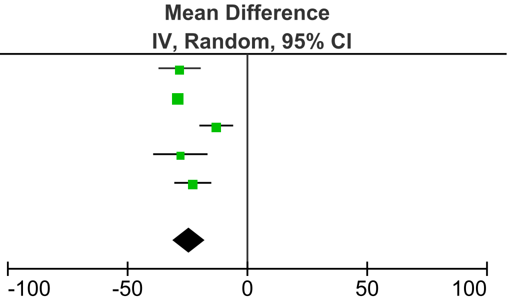

Confidential: For Review Only

**Supplement 1: databases searched**
Cochrane Central Register of Controlled Trials
EBSCO–CINAHL (Cumulative Index to Nursing and Allied Health Literature)
Google Scholar
Ovid–EMBASE
Ovid–MEDLINE
Ovid–MEDLINE E-pub ahead of print
Ovid–MEDLINE In-Process and Other Non-Indexed Citations
PubMed
Scopus
Web of Knowledge (Science Citation Index Expanded and Conference Proceedings Citation Index Science)

Confidential: For Review Only

Supplement 2: Medline Ovid Search Strategy

1. exp CHILD/
2. exp Child, Preschool/
3. exp ADOLESCENT/
4. exp INFANT/ or exp INFANT, NEWBORN/
5. (child* or toddler* or baby or infant* or adolescent*).mp.
6. 1 or 2 or 3 or 4 or 5
7. exp Educational Status/
8. exp Child Development/
9. exp Learning Disorders/
10. exp Educational Measurement/
11. exp SCHOOLS/
12. exp Academic Performance/
13. school performance.mp.
14. exp COGNITION/
15. exp LEARNING/
16. exp SPATIAL LEARNING/
17. exp VERBAL LEARNING/
18. exp SOCIAL LEARNING/
19. exp Intelligence Tests/
20. exp INTELLIGENCE/
21. exp Intellectual Disability/
22. exp Neurodevelopmental Disorders/
23. neurodevelopm*.mp.
24. (nervous system dys* or CNS dys*).mp. [mp=title, abstract, heading word, drug trade name, original title, device manufacturer, drug manufacturer, device trade name, keyword, floating subheading word, candidate term word]
25. (nervous system abnorm* or CNS abnorm*).mp. [mp=title, abstract, heading word, drug trade name, original title, device manufacturer, drug manufacturer, device trade name, keyword, floating subheading word, candidate term word]
26. (nervous system malform* or CNS malform*).mp. [mp=title, abstract, heading word, drug trade name, original title, device manufacturer, drug manufacturer, device trade name, keyword, floating subheading word, candidate term word]
27. (nervous system dis* or CNS dis*).mp. [mp=title, abstract, heading word, drug trade name, original title, device manufacturer, drug manufacturer, device trade name, keyword, floating subheading word, candidate term word]
28. (mental health condi* or mental health dis*).mp. [mp=title, abstract, heading word, drug trade name, original title, device manufacturer, drug manufacturer, device trade name, keyword, floating subheading word, candidate term word]
29. mental health outcome.mp.
30. behaviour* abnorm*.mp.
31. cognitive impairment.mp. or exp Cognitive Dysfunction/
32. visual impairment.mp. or exp Vision Disorders/
33. visual develop*.mp.
34. (visual dis* or visual dys*).mp. [mp=title, abstract, heading word, drug trade name, original title, device manufacturer, drug manufacturer, device trade name, keyword, floating subheading word, candidate term word]

Confidential: For Review Only

35. (nystagmus or strabismus).mp.
36. (visual acuity or refractive error*).mp.
37. hearing impairment.mp. or exp Hearing Loss/
38. exp Deafness/
39. exp DEAF-BLIND DISORDERS/
40. exp Hearing Loss, Sensorineural/
41. exp Movement Disorders/
42. exp Cerebral Palsy/
43. motor impairment.mp.
44. (seizure* or convulsi*).mp.
45. exp EPILEPSY/ or epilepsy.mp.
46. exp Executive Function/
47. visual-motor impairment.mp.
48. numeracy.mp.
49. literacy.mp. or exp LITERACY/
50. jaundice.mp.
51. exp Language Development Disorders/ or exp Child Language/ or language impairment.mp. or exp Reading/ or exp Dyslexia/ or reading impairment.mp.
52. 7 or 8 or 9 or 10 or 11 or 12 or 13 or 14 or 15 or 16 or 17 or 18 or 19 or 20 or 21 or 22 or 23 or 24 or 25 or 26 or 27 or 28 or 29 or 30 or 31 or 32 or 33 or 34 or 35 or 36 or 37 or 38 or 39 or 40 or 41 or 42 or 43 or 44 or 45 or 46 or 47 or 48
53. 49 or 50 or 51
54. 52 or 53
55. exp JAUNDICE, NEONATAL/
56. exp JAUNDICE/
57. exp Hyperbilirubinemia, Neonatal/
58. exp Hyperbilirubinemia/
59. hyperbilirubin*.mp.
60. exp Hyperbilirubinemia, Hereditary/
61. bilirubin encephalopathy.mp.
62. bilirubin-induced neuro*.mp.
63. exchange transfusion.mp.
64. exp ASPHYXIA NEONATORUM/
65. (exp ASPHYXIA/ or asphyxia.mp.) and neonat*.mp.
66. exp Hypoxia-Ischemia, Brain/ and neonat*.mp.
67. perinatal asphyxia.mp.
68. birth asphyxia.mp.
69. (hypoxic-ischemic encephalopathy or hypoxic-ischaemic encephalopathy).mp.
70. neonatal encephalopathy.mp.
71. (exp Cerebral Hemorrhage/ or exp Intracranial Hemorrhages/ or exp Brain Ischemia/ or intracranial haemorrhage.mp. or exp Subarachnoid Hemorrhage/ or exp Stroke/) and neonat*.mp.
72. perinatal stroke.mp.
73. (central nervous system infection.mp. or exp Central Nervous System Infections/) and neonat*.mp.
74. (exp Meningoencephalitis/ or meningo-encephalitis.mp.) and neonat*.mp.
75. (MENINGITIS/ or meningitis.mp.) and neonat*.mp.

Confidential: For Review Only

76. exp MENINGITIS, VIRAL/ and neonat*.mp.
77. (meningoencephalitis and neonat*).mp.
78. (encephalitis.mp. or exp ENCEPHALITIS, VIRAL/ or exp INFECTIOUS ENCEPHALITIS/ or exp ENCEPHALITIS/) and neonat*.mp.
79. kernicterus.mp. or exp KERNICTERUS/
80. preterm white matter disease.mp.
81. (periventricular leukomalacia.mp. or exp Leukomalacia, Periventricular/) and neonat*.mp.
82. (therapeutic hypothermia.mp. or exp Hypothermia, Induced/) and neonat*.mp.
83. ((subdural haemorrhage or subdural hemorrhage) and neonat*).mp.
84. (exp Hematoma, Subdural/ or subdural haemorrhage.mp. or exp Craniocerebral Trauma/) and neonat*.mp.
85. (intraventricular haemorrhage and neonat*).mp.
86. (tentorial tear and neonat*).mp.
87. (parenchymal haemorrhage and neonat*).mp.
88. (ventriculoperitoneal shunt.mp. or exp Cerebrospinal Fluid Shunts/ or exp Ventriculoperitoneal Shunt/) and neonat*.mp.
89. ((ventricular drain or Rickham reservoir or CSF shunt) and neonat*).mp.
90. neonatal stroke.mp.
91. (cerebrovascular accident and neonat*).mp.
92. neonatal cerebral ischaemia.mp.
93. (exp Intracranial Thrombosis/ or cerebral venous thrombosis.mp.) and neonat*.mp.
94. (seizure.mp. or exp Seizures/) and neonat*.mp.
95. 55 or 56 or 57 or 58 or 59 or 60 or 61 or 62 or 63 or 64 or 65 or 66 or 67 or 68 or 69 or 70 or 71 or 72 or 73 or 74 or 75 or 76 or 77 or 78 or 79 or 80 or 81 or 82 or 83 or 84 or 85 or 86 or 87 or 88 or 89 or 90 or 91 or 92 or 93 or 94
96. exp Cohort Studies/
97. exp Retrospective Studies/
98. (cohort* or (case$ and control$)).tw.
99. exp Cross-Sectional Studies/
100. exp Randomized Controlled Trial/
101. 96 or 97 or 98 or 99 or 100
102. exp "REVIEW"/
103. exp Case Reports/
104. Animals/
105. animal stud*.mp.
106. 102 or 103 or 104 or 105
107. 6 and 52 and 95 and 101
108. 107 not 106

Confidential: For Review Only

Supplement 3: included studies of school-aged outcomes after perinatal brain injury

* overlapping study data; Ω potential error in manuscript; Adjusted Odds Ratio (aOR); Autism spectrum Disorder (ASD); Attention Deficit Hyperactivity Disorder (ADHD); Bayley Scale of Infant Development (BSID); Child Behaviour Checklist (CBCL); Clinical Evaluation of Language Fundamentals (CELF); Cystic Periventricular leukomalacia (cPVL); Gross Motor Function Classification System, (GMFCS); Haemorrhagic parenchymal infarction (HPI); Hazard Ratio (HR); International Classification of Disease (ICD); Intraventricular haemorrhage (IVH); Intelligence Quotient (IQ); Kaufman Assessment Battery for Children (K-ABC); Mental Developmental Index (MDI); Peabody Picture Vocabulary Test (PPVT); Periventricular (PV); Periventricular leukomalacia (PVL); National Institute of Child Health and Human Development (NICHD); Neonatal Intensive Care Unit (NICU); Psychomotor Development Index (PDI); Retinopathy of Prematurity (ROP); Small for Gestational Age (SGA); Spontaneous Intestinal Perforation (SIP); Standard Deviation (SD); Standard Error (SE); Test of Motor Impairment (TOMI); Very low birthweight (VLBW); Visuomotor integration (VMI); Wechsler Abbreviated Scale of Intelligence (WASI); Wechsler Intelligence Scale for Children (WISC); Wechsler Preschool & Primary Scale of Intelligence (WPPSI); White Matter Injury (WMI); Wide Range Achievement Test (WRAT)

	Author<br>Year<br>Country<br>Study type	Population<br>Exposures<br>Comparator<br>Ascertainment/ definition	Outcomes	Main result(s)
1	Adant 2019[9]<br><br>Belgium<br><br>Retrospective cohort	**Population**<br>• Gestation ≤32 weeks with and without spontaneous intestinal perforation (SIP)<br>• Born 1994-2014<br><br>**Exposure (n=19)**<br>• IVH grade 3-4<br><br>**Comparator (n=44)**<br>• Matched on gender, gestational age, date of birth (multiples matched to sibling without SIP)<br>• No IVH<br><br>**Ascertainment/ definition**<br>• Clinical record review	**Outcomes**<br>• Functional disability (composite)<br>• Cognitive<br>• Motor<br>• Visual<br>• Behavioural/ mental health<br>• Wellbeing<br>• Quality of life<br>• Physical health<br><br>**Measurement/ assessment**<br>• BSID II<br>• Telephone survey (parents)<br>• PedsQL<br>• IQ testing<br><br>**Follow-up**<br>• 67% follow-up at 7-11 months<br>• 41% follow-up at 18-22 months<br>• 49% follow-up at 4-10 years<br>• 86% follow-up telephone survey	**Outcomes of those with SIP compared to controls without SIP – by IVH subgroup**<br><br>**Disability**<br>aOR 8.79 95%CI (1.72, 44.86)<br><br>**Multiple disabilities**<br>aOR 5.97 95%CI (1.61, 22.15)<br><br>**Cognitive**<br>**Regular education system (not a special educational needs school)**<br>aOR 8.73 95%CI (2.1, 36.72*)*<br><br>**Visual outcomes** (wearing glasses)<br>aOR 0.474 95%CI (0.13, 1.69)<br><br>**Behavioural/ mental health disorder** (including attention problems, conduct problems and autism spectrum disorders)<br>aOR 1.24 95%CI (0.32, 4.8)<br><br>**PedsQL low quality of life score**<br>aOR 0.87 95%CI (0.77, 0.99)<br><br>**PedsQL low physical health score**<br>aOR 0.82 95%CI (0.66, 1.01)
2*	Beaino 2010[68]<br><br>France<br><br>Prospective cohort	**Population**<br>• Gestation <33 weeks<br>• Born 1997<br><br>**Exposure**<br>• IVH grade 1 (n=173)<br>• IVH grade 2 (n=117)<br>• IVH grade 3 (n=32)<br>• Intraparenchymal haemorrhage (IPH) (n=6)<br>• Persistent echodensities or ventricular dilatation (n=241)<br>• cPVL (n=66)<br><br>**Comparator (n=1153)**<br>• Unmatched<br>• No IVH<br><br>**Ascertainment/ definition**<br>• Ultrasound imaging undertaken and reviewed by neonatologists or radiographers	**Outcomes**<br>• Cerebral palsy<br><br>**Measurement/assessment**<br>• Standardised questionnaires completed by physicians<br><br>**Follow-up**<br>• 5 years<br>• 77% follow-up	**Cerebral palsy**<br>**Grade 3 IVH**<br>OR 3.75 95%CI (2.41–5.85)<br><br>**Grade 3 IVH or echodensities of ventricular dilatation**<br>Model A aOR 3.25 95%CI (2.02–5.22)<br>Model B aOR 3.40 95%CI (2.07–5.60)<br>Model C aOR 3.31 95%CI (2.00–5.48)<br><br>**cPVL**<br>OR 33.41 95%CI (19.25–57.96)<br><br>**Cystic PVL or IPH**<br>Model A aOR 29.66 95%CI (16.71–52.62)<br>Model B aOR 28.41 95%CI (15.65–51.59)<br>Model C n/a
3	Brouwer 2012[18]<br><br>Netherlands<br><br>Prospective cohort	**Population**<br>• Gestation <32 weeks<br>• Born 1999-2004<br><br>**Exposure (n=32)**<br>• Post-haemorrhagic ventricular dilatation after IVH grade 3-4 requiring neurosurgical intervention<br>• No PVL<br><br>**Comparator (n=23)**<br>• Matched on gestation, birthweight, and sex<br>• No IVH<br><br>**Ascertainment/ definition**<br>• Ultrasound diagnosis<br>• Papile classification	**Outcomes**<br>• Motor<br>• Cerebral palsy<br>• Cognitive<br>• Behavioural<br><br>**Measurement/ assessment**<br>• Movement ABC<br>• GMFCS<br>• WPPSI (3rd edition Dutch version)<br>• Revisie Amsterdamse Kinder Intelligentietest<br>• Snijders Oomen Nonverbal Intelligence Test 2.5-7 – Revised<br>• CBCL<br>• Teacher Report Form<br><br>**Follow-up**<br>• 4-8 years (median 5.7)<br>• 97% follow-up	**Cerebral palsy**<br>IVH grade 3 n=0<br>IVH grade 4 n=8, 53%; all unilateral spastic cerebral palsy<br>GMFCS level 1, n=5<br>GMFCS level 2, n=2<br>GMFCS level 3, n=1<br><br>**Movement ABC motor score (for those without cerebral palsy)**<br>**Score <p 5 (definite motor problems)**<br>IVH grade 3 n=6, 26%<br>IVH grade 4 n=3, 13%<br>No IVH n=0<br><br>**Score p 5-15 (borderline motor function)**<br>IVH grade 3 (n=6; 26%)<br>IVH grade 4 (n=0; 0%)<br>No IVH (n=5; 29.4%)<br><br>**Score p> 15**<br>IVH grade 3 n=6, 26%<br>IVH grade 4 n=0, 0%<br>No IVH n=12, 70.6%<br><br>**Cognition**<br>**Wechsler intelligence test (mean ±SD)**<br>**Verbal scale**<br>IVH n=23, 97±13<br>IVH <30weeks' gestation n=16, 94±13<br>No IVH n=24, 96±13;<br><br>**Performance scale**<br>IVH, n=23, 94±16;<br>IVH <30weeks' gestation n=16, 93±15<br>No IVH n=24, 103±14;<br><br>**Production scale**

Confidential: For Review Only

				IVH n=23, 87±22;<br>IVH <30weeks' gestation n=16, 85±24<br>No IVH n=24, 93±14<br><br>**Intelligence quotient (n; mean +/-SD)**<br>IVH grade 3 n=17; IQ 96±15;<br>IQ>85 n=13 (76.5%)<br><br>IVH IV n=15; IQ 91±10;<br>IQ >85 n=9 (64.3%)<br><br>IVH <30 weeks' gestation n=23; IQ 92±17;<br>IQ>85 n=15 (65.2%)<br><br>No IVH n=23; IQ 98±15,<br>IQ>85 n=17 (74%)<br><br>**Behavioural outcomes**<br>**CBCL parental score: mean T score ±SD, n in subclinical range (%)**<br>**Total scale**<br>IVH n=26: 48.2 ±8.4, n=3 (12%)<br>IVH <30 weeks' gestation n=20: 46.9 ±8.3, n=2 (10%)<br>No IVH <30 weeks' gestation n=23: 44.3 ±7.8, n=1 (4%)<br><br>**Internalising problem scale**<br>IVH: 49.2 ±8.9, n=5 (19%)<br>IVH <30 weeks' gestation: 28.2 ±8.4, n=3 (15%)<br>No IVH <30 weeks' gestation: 49.2 ±9.1, n=5 (21%)<br><br>**Externalizing problem scale**<br>IVH: 46.8 ±9.4, n=2 (8%)<br>IVH <30 weeks' gestation: 45.1 ±9.5, n=1 (15%)<br>No IVH < 30weeks' gestation: 43.7 ±7.5, n=0 (0%)<br><br>**TRF teachers score: mean T score ±SD, n in subclinical range (%)**<br>**Total scale**<br>IVH n=25: 54.7 ±8.7, n=6 (24%)<br>IVH <30 weeks' gestation n=19: 53.9 ±9.0, n=4 (21%)<br>No IVH <30 weeks' gestation n=22: 50.9 ±9.8, n=4 (18%)<br><br>**Internalising problem scale**<br>IVH: 53.2 ±10.8, 4 (16%)<br>IVH <30 weeks' gestation: 52.2 ±11.7, n=3 (16%)<br>No IVH <30 weeks' gestation: 52.4 ±11.4, n=7 (32%)<br><br>**Externalizing problem scale**<br>IVH: 54.3 ±6.7, 3 (12%)<br>IVH <30 weeks' gestation: 54.1 ±7.0, n=2 (11%)<br>No IVH <30 weeks' gestation: 49.7 ±7.7, n=2 (9%)<br><br>N=13 (41%) had repeated a school class, had educational help and/or attended special education
4	Campbell 2021[10]<br><br>USA<br><br>Prospective cohort study	**Population (n=858)**<br>• Gestation 23-27 weeks<br>• Born 2002-2004<br><br>**Exposure**<br>• IVH without WMI (n=124)<br>• WMI without IVH (n=30)<br>• IVH and WMI (n=63)<br><br>**Comparator (n=641)**<br>• Unmatched<br>• No IVH or WMI<br><br>**Ascertainment/ definition**<br>• Ultrasound imaging reviewed by two independent blinded radiologists<br>• WMI: parenchymal echolucency or moderate to severe ventriculomegaly on a late scan	**Outcomes**<br>• Neurocognitive development (composite)<br>• Cognitive<br>• Cerebral palsy<br>• Behavioural/ mental health<br>• Epilepsy<br>• Quality of life<br><br>**Measurement/ assessment**<br>• Differential Ability Scale II<br>• NEPSY II<br>• Neurological exam<br>• GMFCS<br>• Parental questionnaire<br>• Social Communication Questionnaire<br>• Child Symptom Inventory 4<br>• Peds QoL 4<br><br>**Follow up**<br>• 10 years<br>• 74% follow-up	**Neurodevelopmental burden**<br>**No impairments**<br>IVH and WMI n=24, 38%<br>WMI n=12, 40%<br>IVH n= 86, 69%<br>No IVH or WMI n=487, 76%<br><br>**No cognitive impairment; 1 or more of cerebral palsy, ASD, or epilepsy**<br>IVH and WMI n=4, 6%<br>WMI n=4, 13%<br>IVH n=7, 6%<br>No IVH or WMI n=26, 4%<br><br>**Cognitive**<br>**Normal cognitive function**<br>IVH and WMI n=8, 13%<br>WMI n=5, 17%<br>IVH n=41, 33%<br>No IVH or WMI n=235, 37%<br><br>**Cognitive impairment (moderate to severe)**<br>**IVH and WMI**<br>n=35, 56%<br>OR 5.01 95% CI (2.94, 8.54)<br>aOR 4.49 95% CI (2.49, 8.11)<br><br>**WMI**<br>n=14, 47%<br>OR 3.51 95% CI (1.67, 7.37)<br>aOR 5.07 95% CI (2.13, 12.02)<br><br>**IVH**<br>n=31, 25%<br>OR 1.34 95% CI (0.85, 2.1)<br>aOR 1.21 95% CI (0.73, 1.98)<br><br>**No IVH or WMI**<br>n=128. 20%<br>Reference category<br><br>**Low cognitive function**<br>IVH and WMI n=18. 30%<br>WMI n=10, 34%<br>IVH n=50, 41%<br>No IVH or WMI n=269, 43%<br><br>**Moderate cognitive impairment**<br>IVH and WMI n=17, 28%

Confidential: For Review Only

				WMI n=7, 24%<br>IVH n=24, 20%<br>No IVH or WMI n=93, 15%<br><br>**Severe cognitive impairment**<br>IVH and WMI n=18, 30%<br>WMI n=7, 24%<br>IVH n=7, 6%<br>No IVH or WMI n=35, 6%<br><br>**Nonverbal IQ**<br>**IVH vs. No IVH or WMI**<br>Crude mean difference -3 95%CI (-6.6, 0.6)<br><br>**Full scale IQ**<br>**IVH vs No IVH or WMI**<br>Crude mean difference -2.2 95%CI (-5.7, 1.4)<br><br>**Cerebral palsy**<br>**IVH and WMI**<br>n=32, 51%<br>OR 16.85 95% CI (9.29, 30.55)<br>aOR 13.43 95% CI (7, 25.78)<br><br>**WMI**<br>n=14, 47%<br>OR 14.28 95% CI (6.48, 41.48)<br>aOR 18.63 95% CI (7.37, 47.06)<br><br>**IVH**<br>n=9, 7%<br>OR 1.28 95% CI (0.6, 2.72)<br>aOR 1.19 95% CI (0.54, 2.61)<br><br>**No IVH or WMI**<br>n=37, 6%<br>Reference category<br><br>**GMFCS>0**<br>IVH and WMI n=16, 25%<br>WMI n=10, 33%<br>IVH n=4, 3%<br>No IVH or WMI n=13, 2%<br><br>**Epilepsy**<br>**IVH and WMI**<br>n=12, 19%<br>OR 5.44 95 % CI (2.72, 10.86)<br>aOR 4.89 95% CI (2.31, 10.35)<br><br>**WMI**<br>n=8, 27%;<br>OR 6.92 95% CI (2.86, 16.75)<br>aOR 7.56 95% CI (2.85, 20.06)<br><br>**IVH**<br>n= 11, 9%;<br>OR 1.85 95% CI (0.91, 3.78)<br>aOR 1.5 95% CI (0.68, 3.3)<br><br>**No IVH or WMI**<br>n=25, 4%<br>Reference category<br><br>**Neuropsychiatric/ behavioural outcomes**<br>**ASD**<br>**IVH and WMI**<br>n=4, 6%<br>OR 0.97 95% CI (0.34, 2.79)<br>aOR 0.58 95% CI (0.19, 1.77)<br><br>**WMI**<br>n=2, 7%<br>OR 1.02 95% CI (0.23, 4.42)<br>aOR 0.74 95% CI (0.09, 5.88)<br><br>**IVH**<br>n=11, 9%<br>OR 1.39 95% CI (0.69, 2.78)<br>aOR 1.24 95% CI (0.59, 2.6)<br><br>**No IVH or WMI**<br>n=42, 7%<br>Reference category<br><br>**Social responsiveness scale** (over 65 among children with IQ >85 excluding those with ASD)<br>IVH and WMI n=5, 8%<br>WMI n=4, 13%<br>IVH n=14, 11%<br>No IVH or WMI n=62, 10%<br><br>**ADHD**<br>IVH and WMI n=13, 24%<br>WMI n=3, 10%<br><br>IVH n=31, 25%<br>OR 1.6 95% CI (1.1, 2.5)<br><br>No IVH or WMI n=97, 15%

Confidential: For Review Only

				**Anxiety (parent-reported)**<br>IVH and WMI n=6, 10%<br>WMI n=3, 10%<br>IVH n=10, 8%<br>No IVH or WMI n=98, 15%<br><br>**Anxiety (teacher-reported)**<br>IVH and WMI n=12, 19%<br>WMI n=3, 10%<br>IVH n=14, 11%<br>No IVH or WMI n=88, 14%<br><br>**Depression (parent-reported)**<br>IVH and WMI n=7, 11%<br>WMI n=7, 23%<br>IVH n=14, 11%<br>No IVH or WMI n=100, 16%<br><br>**Depression (teacher-reported)**<br>IVH and WMI n=20, 32%<br>WMI n=7 23%<br>IVH n=18, 15%<br>No IVH or WMI n=96, 15%<br><br>**Poor quality of life (<70)**<br>IVH and WMI n=31, 49%<br>WMI n=12, 40%<br>IVH n=41, 25%<br>No IVH or WMI n=131, 20%
5	Cheong 2018[11]<br><br>Australia<br><br>Three prospective cohort studies	**Population**<br>• Gestation 22-27 weeks<br>• Born 1991-1992; 1997-1998; 2005-2006<br><br>**Exposure**<br>• IVH grade 3-4 (n=100)<br>• cPVL (n=38)<br><br>**Comparator**<br>• Unmatched<br>• No IVH grade 3-4 (n=446)<br>• No cPVL (n=508)<br><br>**Ascertainment/ definition**<br>• Not specified	**Outcomes**<br>• Survival with major disability (composite)<br>• Survival without major disability (composite)<br>• Cognitive<br>• Cerebral palsy<br>• Visual impairment (acuity less than 6/60 in better eye)<br>• Hearing impairment (requiring hearing aid or cochlear amplification)<br>**Assessment/ measurement**<br>• GMFCS<br>• WISC III<br>• WISC IV<br>• Differential Abilities Scales 2nd edition<br>**Follow-up**<br>• 8 years<br>• 91% follow-up of survivors	**Survival with major disability**<br>**IVH grade 3-4**<br>OR 2·98 95% CI (1·34, 6·63) p=0.01<br>aOR 2·61 95%CI (1·11–6·15) p=0·028<br><br>1997 and 2005 cohort only:<br>OR 4·01 95% CI (1·25, 12·84) p=0.02<br><br>**cPVL**<br>OR 8·11 95% CI (3·24, 20·30) p<0.001<br>aOR 9·17 95% CI (3·57–23·53) p<0·0001<br><br>1997 and 2005 cohort only<br>OR 17·0 95% CI (4·19, 69·02) p<0·001
6	Chou 2020[69]<br><br>Taiwan<br><br>Retrospective cohort study	**Population**<br>• Preterms infants <37 weeks' gestation (n=21,474)<br>• Infants born small for gestational age (n=2206)<br>• Born 2000-2010<br><br>**Exposure**<br>• Preterm with cerebral haemorrhage<br>• SGA with cerebral haemorrhage<br><br>**Comparator (n=94,720)**<br>• Matched 1:4 on gender, urbanisation of residential area and parental occupation<br>• No cerebral haemorrhage<br><br>**Ascertainment/ definition**<br>• National children's medical record database<br>• ICD 9 codes	**Outcome**<br>• Epilepsy<br><br>**Assessment/ measurement**<br>• ICD 9<br><br>**Follow-up**<br>• 2-12 years (mean 9 years)<br>• Completeness of follow-up not specified	**Epilepsy**<br>**Preterm with cerebral haemorrhage**<br>HR 42.4 95%CI (29.8, 60.3)<br>aHR 42.5 95 %CI (29.6, 60.5)<br><br>**SGA with cerebral haemorrhage**<br>HR 39.3 95%CI (5.51, 274.5)<br>aHR 38.7 95%CI (5.43, 275.5)
7	Davidovitch 2020[29]<br><br>Israel<br><br>Retrospective cohort study	**Population (n=4963)**<br>• VLBW infants ≤1500g<br>• Born 1999-2012<br><br>**Exposure**<br>• IVH grade 3-4 (n=256)<br>• PVL (n=200)<br>• Post-haemorrhagic hydrocephalus (n=152)<br><br>**Comparator**<br>• Unmatched<br>• No IVH grade 3-4 (n=4600)<br>• No PVL (n=3813)<br>• No post-haemorrhagic hydrocephalus (n=4810)<br><br>**Ascertainment/ definition**<br>• Israel national very low birthweight infant database linked to electronic medical records.<br>• Ultrasound diagnosis<br>• Papile classification	**Outcome**<br>• ASD<br><br>**Assessment/ measurement**<br>• Physical, neurological, and developmental assessment (by a qualified healthcare professional)<br>• Independent psychological assessment<br><br>**Follow-up**<br>• 8- 15 years (median 11.6)<br>• Only those linked to electronic medical records included	**ASD**<br>IVH n=10, 3.9%<br>No IVH n=103, 2.2% p=0.085<br><br>PVL n=5, 2.5%<br>No PVL n=88, 2.3% p=0.86<br><br>Post-haemorrhagic hydrocephalus n=7, 4.6%<br>No post-haemorrhagic hydrocephalus n=106, 2.2% p=0.051<br><br>IVH, PVL, post-haemorrhagic hydrocephalus or ROP n=27,23.9%<br>No brain injury n=571, 11.8% p<0.0001<br>aOR 1.62 95% CI (0.96–2.73)
8	Doyle 2000[70]<br><br>Australia	**Population**<br>• Birthweight 500–1499 g<br>• Born 1980-1981; 1992	**Outcomes**<br>• Survival<br>• Cerebral palsy	**Cerebral Palsy**<br><br>Grade of IVH

Confidential: For Review Only

	Prospective Cohort	**Exposure**<br>**1980s epoch**<br>• IVH grade 1 (n=18)<br>• IVH grade 2 (n=9)<br>• IVH grade 3 (n=7)<br>• IVH grade 4 (n=4)<br><br>**1992 epoch**<br>• IVH grade 1 (n=23)<br>• IVH grade 2 (n=10)<br>• IVH grade 3 (n=9)<br>• IVH grade 4 (n=1)<br><br>**Comparator**<br>• Unmatched<br>• No intracranial haemorrhage (n=223)<br>• 1980s epoch (n=110)<br>• 1992 epoch (n=113)<br><br>**Ascertainment/ definition**<br>• Ultrasound imaging<br>• Post-mortem examination<br>• Papile classification	**Measurement/assessment**<br>• Clinical assessment by blinded paediatricians<br>• Functional assessment<br><br>**Follow-up**<br>• 5 years<br>• 93% follow-up for 1980s epoch<br>• 94% follow-up for 1992 epoch	**1980s epoch**<br>No IVH n=5, 5%<br>IVH grade 3 n=2, 29%<br>IVH grade 4 n=0<br><br>**1992s epoch**<br>No IVH n=4, 4%<br>IVH grade 3 n=3, 33%<br>IVH grade 4 n=1, 100%
9	Hintz 2018[17]<br><br>USA<br><br>Retrospective cohort	**Population**<br>• Gestation 24-28 weeks<br>• Born 2005-2009<br><br>**Exposure**<br>MRI<br>• Mild WMI (n=223)<br>• Moderate WMI (n=51)<br>• Severe WMI (n=15)<br><br>• Any cerebellar lesion (n=57)<br><br>• Significant cerebellar lesion (n=39)<br><br>Early cranial ultrasound<br>• No IVH 3-4 or cPVL (n=341)<br>• IVH 3-4 or cPVL (n=32)<br><br>Late cranial ultrasound<br>• No porencephalic cyst, cPVL, moderate to severe ventricular enlargement or shunt (n=354)<br>• Porencephalic cyst, cPVL, moderate to severe ventricular enlargement or shunt (n=19)<br><br>**Comparator**<br>• No white matter injury on MRI (n=84)<br>• No cerebellar lesion on MRI (n=316)<br>• No IVH 3-4 or cPVL (n=32)<br>• Normal early cranial ultrasound (n=227)<br>• No porencephalic cyst, cPVL moderate to severe ventricular enlargement or shunt (n=19)<br>• Normal late cranial ultrasound (n=284)<br><br>**Ascertainment/ definition**<br>• NICHD neonatal research network (NEURO study and SUPPORT cohort)<br>• Two masked central imaging readers for all cranial ultrasounds and one for MRI<br>• All had cranial ultrasound and MRI (at 35-42 weeks)<br>• Unilateral and bilateral cranial ultrasound lesions combined	**Outcomes**<br>• Moderate to severe disability (composite)<br>• Minimal or no disability<br>• Cognitive<br>• Cerebral palsy<br>• Hearing<br>• Vision<br><br>**Measurement/ assessment**<br>• WISC IV<br>• Neurological exam<br>• GMFCS<br>• Clinical examination<br>• Parental report<br><br>**Follow-up**<br>• 6-7 years<br>• 83.3% follow-up of survivors	**White matter injury**<br>**Moderate to severe disability**<br>No white matter injury, n=8, 9%<br>Mild white matter injury, n=27, 12%<br>Moderate white matter injury, n=8, 15%<br>Severe white matter injury, n=14, 82%<br>p<0.0001<br><br>Moderate or severe white matter injury<br>aOR 1.1 95% CI (0.42, 2.92)<br><br>**Minimal or no disability**<br>No white matter injury, n=47, 55%<br>Mild white matter injury, n=88, 224%<br>Moderate white matter injury, n=15, 28%<br>Severe white matter injury, n=0, 0%<br>p<0.0001<br><br>**Cognitive impairment (FSIQ mean (SD))**<br>No white matter injury, 90.1 (15.5)<br>Mild white matter injury, 85.9 (16.8)<br>Moderate white matter injury, 84 (17)<br>Severe white matter injury, 62.7 (19.6)<br>p<0.0001<br><br>**Cognitive impairment FSIQ <70**<br>No white matter injury, n=7, 8%<br>Mild white matter injury, n=25, 11%<br>Moderate white matter injury, n=6, 12%<br>Severe white matter injury, n=9, 60%<br>p<0.0001<br><br>Moderate or severe white matter injury<br>aOR 1.14 95% CI (0.39, 3.26)<br><br>**Cognitive impairment FSIQ <85**<br>No white matter injury, n=27, 32%<br>Mild white matter injury, n=100, 45%<br>Moderate white matter injury, n=29, 57%<br>Severe white matter injury, n=13, 87%<br>p<0.0001<br><br>**No cognitive impairment FSIQ ≥85**<br>No white matter injury, n=57, 68%<br>Mild white matter injury, n=123, 55%<br>Moderate white matter injury, n=22, 43%<br>Severe white matter injury, n=2, 13%<br>p<0.0001<br><br>**Any cerebral palsy**<br>No white matter injury, n=2, 2%<br>Mild white matter injury, n=6, 3%<br>Moderate white matter injury, n=4, 7%<br>Severe white matter injury, n=10, 59%<br>p<0.0001<br><br>**Cerebral palsy with GMFCS ≥2**<br>No white matter injury, n=0, 0%<br>Mild white matter injury, n=1, 0%<br>Moderate white matter injury, n=1, 2%<br>Severe white matter injury, n=4, 24%<br>p<0.0001<br><br>**Cerebellar lesions**<br>**Moderate to severe disability**<br>No cerebellar lesion, n=37, 12%<br>Any cerebellar lesion, n=20, 33% p<0.0001<br>Significant cerebellar lesion, n=15, 36%<br><br>Significant cerebellar lesions<br>aOR 2.71 95% CI (1.09, 6.71)<br><br>**Minimal or no disability**

Confidential: For Review Only

				No cerebellar lesion, n=135, 42%<br>Any cerebellar lesion n=15, 25% p<0.0001<br>Significant cerebellar lesion, n=15, 36%<br><br>**Cognitive impairment (FSIQ mean (SD))**<br>No cerebellar lesion, 87 (16.5)<br>Any cerebellar lesion 78.4 (20) p=0.001<br>Significant cerebellar lesion 76.8 (20.4)<br><br>**Cognitive impairment FSIQ <70**<br>No cerebellar lesion, n=32, 10%<br>Any cerebellar lesion, n=15, 26% p=0.001<br>Significant cerebellar lesion, n=10, 26%<br><br>Significant cerebellar lesions<br>aOR 1.96 95% CI (0.72, 5.36)<br><br>**Cognitive impairment FSIQ <85**<br>No cerebellar lesion, n=136, 43%<br>Any cerebellar lesion, n=33, 58% p=0.038<br>Significant cerebellar lesion, n=22, 56%<br><br>**No cognitive impairment FSIQ ≥85**<br>No cerebellar lesion, n=180, 57%<br>Any cerebellar lesion, n=24, 42% P=0.038<br>Significant cerebellar lesion, n=17, 44%<br><br>**Any cerebral palsy**<br>No cerebellar lesion, n=13, 4%<br>Any cerebellar lesion, n=9, 15% p=0.001<br>Significant cerebellar lesion, n=9, 21%<br><br>**Cerebral palsy with GMFCS ≥2**<br>No cerebellar lesion, n=3, 1%<br>Any cerebellar lesion, n=3, 5% p=0.19<br>Significant cerebellar lesion, n=3, 7%<br><br>**Early cranial ultrasound abnormalities**<br>**Moderate to severe disability**<br>No IVH 3-4 or cPVL, n=43, 12%<br>IVH 3-4 or cPVL, n=14, 42% p<0.0001<br>Normal scan, n=35, 12%<br>aOR 0.61 95% CI (0.14, 2.59)<br><br>**Minimal or no disability**<br>No IVH 3-4 or cPVL, n=143, 41%<br>IVH 3-4 or cPVL, n=7, 21% p<0.0001<br>Normal scan, n=120, 43%<br><br>**Cognitive impairment, FSIQ mean (SD)**<br>No IVH 3-4 or cPVL, 86.4 (17)<br>IVH 3-4 or cPVL, 77.9 (19.1) p=0.008<br>Normal scan, 86 (16.7)<br><br>**Cognitive impairment FSIQ <70**<br>No IVH 3-4 or cPVL, n=38, 11%<br>IVH 3-4 or cPVL, n=9, 28% p=0.006<br>Normal scan, n=31, 11%<br>aOR 0.42 95% CI (0.07, 2.33)<br><br>**Cognitive impairment FSIQ <85**<br>No IVH 3-4 or cPVL, n=149, 44%<br>IVH 3-4 or cPVL, n=20, 63% p=0.041<br>Normal scan, n=123, 44%<br><br>**No cognitive impairment FSIQ ≥85**<br>No IVH 3-4 or cPVL, n=192, 56%<br>IVH 3-4 or cPVL, n=12, 38% p=0.041<br>Normal scan, n=154, 56%<br><br>**Any cerebral palsy**<br>No IVH 3-4 or cPVL, n=149, 44%<br>IVH 3-4 or cPVL, n=20, 63% p=0.041<br>Normal scan, n=123, 44%<br><br>**Cerebral palsy with GMFCS ≥2**<br>No IVH 3-4 or cPVL, n=3, 1%<br>IVH 3-4 or cPVL, n=3, 9% p<0.0001<br>Normal scan, n=2, 1%<br><br>**Late cranial ultrasound abnormalities**<br>**Moderate to severe disability**<br>No porencephalic cyst, cPVL, moderate to severe ventricular enlargement or shunt, n=40, 11%<br>Porencephalic cyst, cPVL, moderate to severe ventricular enlargement or shunt, n=17, 77% p<0.0001<br>Normal scan, n=27, 10%<br>aOR 27.85 95% CI (6.03, 128.68)<br><br>**Minimal or no disability**<br>No porencephalic cyst, cPVL, moderate to severe ventricular enlargement or shunt, n=149, 42%<br>Porencephalic cyst, cPVL, moderate to severe ventricular enlargement or shunt, n=1, 5% P<0.0001<br>Normal scan, n=117, 43%<br><br>**Cognitive impairment (FSIQ mean (SD))**<br>No porencephalic cyst, cPVL, moderate to severe ventricular enlargement or shunt, 86.7 (16.7)<br>Porencephalic cyst, cPVL, moderate to severe ventricular enlargement or shunt, 65.9 (18.7) P<0.0001

Confidential: For Review Only

				Normal scan, 87 (16.1)<br><br>**Cognitive impairment FSIQ <70**<br>No porencephalic cyst, cPVL, moderate to severe ventricular enlargement or shunt, n=36, 10%<br>Porencephalic cyst, cPVL, moderate to severe ventricular enlargement or shunt, n=11, 58% p<0.0001<br>Normal scan, n=24, 9%<br>aOR 20.05 95% CI (3.63, 110.84)<br><br>**Cognitive impairment FSIQ <85**<br>No porencephalic cyst, cPVL, moderate to severe ventricular enlargement or shunt, n=153, 43%<br>Porencephalic cyst, cPVL, moderate to severe ventricular enlargement or shunt, n=16, 84% p<0.0001<br>Normal scan, n=118, 43%<br><br>**No cognitive impairment FSIQ ≥85**<br>No porencephalic cyst, cPVL, moderate to severe ventricular enlargement or shunt, n=201, 57%<br>Porencephalic cyst, cPVL, moderate to severe ventricular enlargement or shunt, n=3, 16% p<0.0001<br>Normal scan, n=156, 57%<br><br>**Any cerebral palsy**<br>No porencephalic cyst, cPVL, moderate to severe ventricular enlargement or shunt, n=10, 3%<br>Porencephalic cyst, cPVL, moderate to severe ventricular enlargement or shunt, n=12, 50% p<0.0001<br>Normal scan, n=6, 2%<br><br>**Cerebral palsy with GMFCS ≥2**<br>No porencephalic cyst, cPVL, moderate to severe ventricular enlargement or shunt, n=2, 1%<br>Porencephalic cyst, cPVL, moderate to severe ventricular enlargement or shunt, n=4, 17% p<0.0001<br>Normal scan, n=1, 0%
10	Hirovonen, 2017[22]<br><br>Finland<br><br>Retrospective cohort	**Population**<br>• Gestation >22 weeks<br>• Birth weight >500g<br>• Born 1991-2008<br><br>**Exposure (n=557)**<br>• Intracranial haemorrhage<br><br>**Comparison (n=708,977)**<br>• No intracranial haemorrhage<br>• ICD code<br><br>**Ascertainment/ definition**<br>• Finnish national register<br>• ICD codes	**Outcomes**<br>• Cognitive<br><br>**Measurement/ assessment**<br>• ICD 9 and 10 codes<br>• BSID 1993<br>• Finnish WISC<br><br>**Follow-up**<br>• 7 years<br>• 98% follow-up	**Any intellectual disability after intracranial haemorrhage (HR (95%CI); p-value)**<br>Very preterm infants 2.92 (1.58–5.41); p= 0.001<br>Moderately preterm 5.59 (1.57–19.9); p= 0.008<br>Late preterm 4.58 (1.36–15.4); p= 0.014<br>Term 2.94 (1.08-8); p=0.035
11	Hollebrandse 2021[19]<br><br>Australia<br><br>Retrospective cohort	**Population**<br>• Gestation <28 weeks<br>• Born 1991-1992, 1997, 2005<br><br>**Exposure**<br>• IVH grade 1 n=80<br>• IVH grade 2 n=53<br>• IVH grade 3 n=23<br>• IVH grade 4 n=12<br><br>**Comparator**<br>• Unmatched<br>• Preterm infants without IVH n=331<br><br>**Ascertainment/ definition**<br>• Ultrasound diagnosis<br>• Worst grade of IVH<br>• Papile classification	**Outcomes**<br>• Cognitive<br>• Motor<br>• Cerebral palsy<br><br>**Assessment/ measurement**<br>• WISC III (1991-1992 cohort)<br>• WISC IV (1997 cohort)<br>• Differential Abilities Scale 2nd edition (2005 cohort)<br>• WRAT III (1991-92; 1997 cohorts)<br>• WRAT IV (2005 cohort)<br>• Behaviour rating inventory of executive functioning (parent-completed)<br>• Movement ABC 1st edition (1991-1992 and 1997 cohorts)<br>• Movement ABC 2nd edition (2005 cohort)<br>• GMFCS (1997 and 2005 cohort)<br>• Blinded assessment<br><br>**Follow-up**<br>• 8 years<br>• Follow-up 85-91.4%	**Cognitive**<br>**IQ score <-2 SD**<br>IVH grade 4 n=5, 42% p=0.08 (X^2 trend)<br>IVH grade 3 n=5, 22%<br>No IVH n=41, 12%<br><br>IVH 3-4: OR 2.68 95% CI (1.21, 5.94) p=0.01<br><br>**Impaired executive function**<br>**Global executive composite ≥65**<br>IVH grade 4 n=2, 18% p=0.78 (X^2 trend)<br>IVH grade 3 n=4, 18%<br>No IVH n=49, 16%<br><br>IVH 3-4: OR 1.17 95% CI (0.46, 2.97) p=0.75<br><br>**Behavioural regulation index ≥65**<br>IVH grade 4 n=2, 18% p=0.21 (X^2 trend)<br>IVH grade 3 n=6, 27%<br>No IVH n=46, 15%<br><br>IVH 3-4: OR 1.76 95% CI (0.75, 4.11) p=0.2<br><br>**Metacognition index ≥65**<br>IVH grade 4 n=3, 27% p=0.1 (X^2 trend)<br>IVH grade 3 n=5, 23%<br>No IVH n=48, 16%<br><br>IVH 3-4: OR 1.73 95% CI (0.74, 4.06) p=0.21<br><br>**Impaired academic skills (any academic skill <-2SD)**<br>IVH grade 4 n=7, 64% p<0.001 (X^2 trend)<br>IVH grade 3 n=5, 24%<br>No IVH n=50, 16%<br><br>IVH 3-4: OR 2.91 95% CI (1.35, 6.27) p=0.006<br><br>**Impaired reading <-2SD**<br>IVH grade 4 n=6, 55% p=0.002 (X^2 trend)<br>IVH grade 3 n=4, 19%<br>No IVH n=21, 10%<br><br>IVH 3-4: OR 3.62 95% CI (1.59, 8.24) p=0.002<br><br>**Impaired spelling <- 2 SD**<br>IVH grade 4 n=5, 45% p=0.011 (X^2 trend)<br>IVH grade 3 n=3, 14%

Confidential: For Review Only

Confidential: For Review Only

				No IVH n=21, 7%<br><br>IVH 3-4: OR 4.48 95% CI (1.8, 11.2) p=0.001<br><br>**Impaired arithmetic < -2 SD**<br>IVH grade 4 n=5, 45% p=0.09 (X^2 trend)<br>IVH grade 3 n=4, 19%<br>No IVH n=38, 12%<br><br>IVH 3-4: OR 2.79 95% CI (1.2, 6.48) p=0.017<br><br>**<u>Motor and cerebral palsy</u>**<br>**Any motor dysfunction (cerebral palsy or MABC <5th centile)**<br>IVH grade 4 n=11, 92% p<0.001 (X^2 trend)<br>IVH grade 3 n=10, 43%<br>No IVH n=81, 24%<br><br>IVH 3-4: OR 4.45 95% CI (2.18, 9.08) p<0.001<br><br>**Cerebral palsy**<br>IVH grade 4 n=9, 75% p<0.001(X^2 trend)<br>IVH grade 3 n=6, 26%<br>No IVH n=26, 8%<br><br>IVH 3-4: OR 8.8 95% CI (4.03, 19.2) p<0.001<br><br>**MABC <5th percentile (for the 2005 cohort)**<br>IVH grade 4 n=11, 92% p<0.001 (X^2 trend)<br>IVH grade 3 n=9, 45%<br>No IVH n=79, 26%<br><br>IVH 3-4: OR 4.7 95% CI (2.21, 9.97) p<0.001
12	Hreinsdottir 2018[48]<br><br>Sweden<br><br>Prospective cohort study	**Population**<br>• Born 2004-2007<br>• Gestation <32 years<br><br>**Exposure (n=9)**<br>• IVH grade 3-4 and/ or PVL<br><br>**Comparator (n=99)**<br>• Unmatched<br>• No IVH grade 3-4 or PVL<br><br>**Ascertainment/ definition**<br>• Ultrasound imaging performed by paediatric radiologist<br>• Papile classification for IVH<br>• PVL defined by size, laterality and as cystic of diffuse	**Outcomes**<br>• Visual impairment<br><br>**Assessment/ measurement**<br>• Linear visual acuity (Lea Hyvarinen chart)<br>• Cover test<br>• Refraction<br><br>**Follow-up**<br>• 6.5 years<br>• 78% follow-up	**<u>Vision</u>**<br>**Subnormal visual acuity**<br>IVH 3-4 and or PVL<br>OR 1.11 95% CI (0.25, 4.83) p=0.891<br><br>**Contrast sensitivity**<br>IVH 3-4 and or PVL<br>OR 1.87 95% CI (0.43, 8.17) p=0.403<br><br>**Refractive error**<br>IVH 3-4 and or PVL<br>OR 2.5 95% CI (0.55, 11.41) p=0.237<br><br>**Manifest strabismus**<br>IVH 3-4 and or PVL<br>OR 4 95% CI (0.65, 24.55) p=0.134<br><br>**<u>Composite score 1:</u> Visual acuity with both eyes of less than 0.3, significant refractive error in the better eye and manifest strabismus**<br>IVH 3-4 and or PVL<br>OR 3.63 95% CI (0.86, 15.41) p=0.08<br>aOR 4.95 95% CI (0.65, 37.48) p=0.121<br><br>**<u>Composite score 2:</u> Visual acuity in worse eye of less than 0.3, significant refractive error in worse eye according and manifest strabismus**<br>IVH 3-4 and or PVL<br>OR 5.67 95% CI (1.34, 24.07) p=0.019<br>aOR 10.4 95% CI (1.23, 88) p=0.032<br><br>**<u>Composite score 3:</u> Visual acuity with both eyes of less than 0.5, significant refractive error in the better eye, manifest strabismus, negative stereopsis and contrast sensitivity less than 0.4**<br>IVH 3-4 and or PVL<br>OR 7.6 95% CI (1.7, 34) p=0.008<br>aOR 18.19 95% CI (2.15, 154.05) p=0.008<br><br>**<u>Composite score 4:</u> Visual acuity with both eyes of 0.8 or less, significant refractive error in the better eye, manifest strabismus, negative stereopsis and CS less than 0.5**<br>IVH 3-4 and or PVL<br>OR 4.63 95% CI (0.9, 23.85) p=0.067<br>a6.23 95% CI (1.15, 33.83) p=0.034
13	Jansen 2020[23]<br><br>Netherlands<br><br>Prospective cohort study	**Population**<br>• Gestation <32 weeks<br>• Admitted 2006-2007<br><br>**Exposure**<br>• Mild WMI (n=18)<br>• Moderate WMI (n=14)<br>• Severe WMI (n=8)<br>• Mild cerebellar injury (n=11)<br>• Moderate cerebellar injury (n=4)<br>• Severe cerebellar injury (n=6)<br><br>**Comparator**<br>• Unmatched<br>• No WMI (n=46)<br>• No cerebellar injury (n=65)<br><br>**Ascertainment/ definition**<br>• Ultrasound imaging and term MRI<br>• Imaging reviewed by two blinded experienced investigators (neonatologists or radiologists)	**Outcomes**<br>• Cognitive<br><br>**Assessment/ measurement**<br>• National standardised achievement tests<br><br>**Follow-up**<br>• 9-10 years<br>• 77% follow-up	**<u>Cognitive</u>**<br>**Reading comprehension**<br>Moderate-severe WMI vs. no injury<br>B 0.241 p=0.483<br><br>Moderate-severe cerebellar injury vs. no injury<br>B 0.799 p=0.325<br><br>**Spelling**<br>Moderate-severe WMI vs. no injury<br>B 1.076 p=0.075<br><br>Moderate-severe cerebellar injury vs. no injury<br>B 1.293 p= 0.115<br><br>**Mathematics**<br>Moderate-severe WMI vs. no injury<br>B 1.856 p=0.003<br><br>Moderate-severe cerebellar injury vs. no injury<br>B 1.504 p=0.088

14	Kaur 2020[32]<br><br>Canada<br><br>Retrospective cohort study	**Population**<br>• Preterm and term infants<br>• Born 2006-2016<br><br>**Exposure**<br>• IVH grade 1 (n=811)<br>• IVH grade 2 (n=186)<br>• IVH grade 3-4 (n=194)<br>• Preterm haemorrhage (n=1139)<br><br>**Comparator**<br>• Unmatched<br>• No IVH (n=793, 062)<br>• Preterm no haemorrhage (n=50, 185)<br><br>**Ascertainment/ definition**<br>• ICD 10 codes (based on ultrasound or MRI imaging)<br>• Papile classification	**Outcome**<br>• Reason for hospitalisation<br><br>**Assessment/ measurement**<br>• ICD 10 codes<br><br>**Follow-up**<br>• 12 years<br>• Completeness of follow-up not specified	**Incidence of hospitalisation for:**<br>**Cerebral palsy, n, incident rate per 1,000 person years (95%CI)**<br>IVH n=57, 6.8 (5.3, 8.8)<br>No haemorrhage n=432, 0.1 (0.1, 0.1)<br>Hazard ratio: 4.78 95% CI (3.21, 7.13)<br><br>IVH grade 3-4 n=24 HR 14.78 95% CI (8.72-25.06)<br><br>**Ophthalmologic, n, incident rate per 1,000 person years (95%CI)**<br>IVH n=91 11.1 (9, 13.6)<br>No haemorrhage n=6773, 1.2 (1.2, 1.3)<br>HR 3.01 95% CI (2.32, 3.89)<br><br>IVH grade 3-4 n=32 HR 7.87 95% CI (5.31-11.67)<br><br>**Otologic n, incident rate per 1,000 person years (95%CI)**<br>IVH n=328, 46.7 (41.9, 52)<br>No haemorrhage n=102,153 22.1 (22, 22.2)<br>HR 1.19 95% CI (1.06, 1.34)<br><br>IVH grade 3-4 n=202 HR 1.07 95% CI (0.79-1.46)
15	Kiechl-Kohlendorfer 2013[28]<br><br>Austria<br><br>Prospective cohort	**Population**<br>• Gestation <32 weeks<br>• Born 2003-2006<br><br>**Exposure**<br>• Intracranial haemorrhage (all grades) (n=24)<br>• Intracranial haemorrhage grade 3-4 (n=4)<br>• PVL (n=2)<br>• Intraparenchymal echodense lesions (n=2)<br><br>**Comparator**<br>• Unmatched<br><br>**Ascertainment/ definition**<br>• Ultrasound imaging<br>• Papile classification	**Outcomes**<br>• Cognitive<br><br>**Measurement/assessment**<br>• Physical examination<br>• Hannover-Wechsler Intelligence Test for preschool children, third edition<br>• WPPSI<br>• Snijders-Oomen Nonverbal Intelligence Test<br>• TEDI-MATH<br><br>**Follow-up**<br>• 5 years<br>• 72.2% follow-up	**Delayed numerical skills**<br>Intracranial haemorrhage (all grades) n=11, 40,7%<br>aOR 4.66 95% CI (1.56, 13.93) p=0.007<br><br>Intracranial haemorrhage grade 3-4 n=3, 11.1%<br>PVL n=2, 7.4%<br>Intraparenchymal echodense lesions n=0
16	Klebermass-Schrehof 2012[20]<br><br>Austria<br><br>Prospective cohort	**Population**<br>• Gestation <32 weeks<br>• Admitted to NICU 1994-2005<br><br>**Exposure**<br>• IVH grade 1 (n=37)<br>• IVH grade 2 (n=84)<br>• IVH grade 3 (n=18)<br>• IVH grade 4 (n=12)<br><br>**Comparator (n=320)**<br>• Unmatched<br>• No IVH<br><br>**Ascertainment/ definition**<br>• Ultrasound diagnosis<br>• Most severe scan used<br>• Papile classification	**Outcomes**<br>• Neurosensory impairment (composite)<br>• Motor<br>• Cerebral palsy<br>• Language<br>• Visual<br>• Hearing<br><br>**Measurement/assessment**<br>• BSID II (MDI, PDI)<br>• K-ABC<br>• Beery-Buktenica Developmental Test of VMI<br>• Clinical assessment<br><br>**Follow-up**<br>• 5 years (1 ,2, and 3.5 years)<br>• Only those with follow-up included (loss to follow-up not specified)	**Outcomes at 5.5 years**<br><br>**Group 1: infants born < 28 weeks' gestation**<br>**KABC <70**<br>No IVH, 7.6%<br>IVH grade 3, 33.3%<br>IVH grade 4, 50%<br><br>**KABC mean (SD)**<br>No IVH, 91.5 (15.1)<br>IVH grade 3, 88.6 (11.1) p=not significant<br>IVH grade 4, 88.5 (10.6) p= not significant<br><br>**VMI mean (SD)**<br>No IVH, 92.7 (20)<br>IVH grade 3, 67.5 (14) p=0.04<br>IVH grade 4, 76 (26.8) p=0.04<br><br>**Cerebral palsy**<br>No IVH, 14.3%<br>IVH grade 3, 63.6% p<0.01<br>IVH grade 4, 90.9% p<0.01<br><br>**Visual impairment**<br>No IVH, 7.5%<br>IVH grade 3, 45.5%, p=0.03<br>IVH grade 4, 90.9% p<0.01<br><br>**Acoustic impairment**<br>No IVH, 2.2%<br>IVH grade 3, 0% p= not significant<br>IVH grade 4, 0% p= not significant
17	Koc 2016[24]<br><br>Turkey<br><br>Retrospective cohort	**Population (n=90)**<br>• Gestation <32 weeks<br>• Birthweight <1500g<br>• Born 2001<br><br>**Exposure**<br>• IVH grade 1-2 (n= 7)<br>• IVH grade 3-4 (n= 8)<br><br>**Comparator**<br>• No IVH (n=75)<br><br>**Ascertainment/ definition**<br>• Neonatal unit database and medical records	**Outcomes**<br>• Cognitive<br><br>**Measurement/ assessment**<br>• WISC-R<br><br>**Follow-up**<br>• 5.9-7.9 years<br>• 100% follow-up	**WISC-R score <85**<br>IVH (n=7; 46.7%)<br>No IVH (n= 25; 33.3%)<br><br>**WISC-R score >85**<br>IVH grade (n=8; 13.8%)<br>No IVH (n= 50; 84.2%)<br><br>p=0.381
18	Martinez-Cruz 2008[45]<br><br>Mexico<br><br>Case control	**Population**<br>• Gestation <34 weeks<br>• Birthweight <1500g<br>• Born 1990-2005<br><br>**Exposure (n=103)**<br>• IVH	**Outcomes**<br>• Sensorineural hearing loss<br><br>**Measurement/ assessment**<br>• Brainstem auditory evoked potentials<br>• Transient auditory evoked otoacoustic emissions<br>• Behavioural hearing evaluation	**IVH**<br>Sensorineural hearing loss (n=71; 48.6%)<br>No sensorineural hearing loss (n=32; 11.8%)<br><br>**Multivariate logistic regression of risk factors for sensorineural hearing loss**<br>IVH: aOR 7.1 95% CI (4.34, 11.6) p<0.000

Confidential: For Review Only

		**Comparator (n=315)**<br>• No IVH<br><br>**Ascertainment/ definition**<br>• Medical records<br>• Ultrasound diagnosis.<br>• Papile classification.	• Free field audiometry<br>• Tympanometry<br>• Pure Tone Audiometry<br><br>**Follow-up**<br>• Mean age 7.8±3.7 years<br>• 100% follow-up (case control)	
19	Neubauer 2008[12]<br><br>Germany<br><br>Prospective cohort	**Population**<br>• Birthweight <1000g<br>• Born 1993-1998<br><br>**Exposure**<br>• IVH grade 1-2 (n=26)<br>• IVH grade 3-4, PVL (n=18)<br><br>**Comparator**<br>• Unmatched<br>• No IVH or PVL (n=91)<br><br>**Ascertainment/ definition**<br>• Ultrasound diagnosis<br>• Papile classification	**Outcomes**<br>• Neurodevelopmental impairment (composite)<br><br>**Measurement/assessment**<br>• Modified Touwen test<br>• K-ABC<br>• Snijders-Oomen Non-Verbal Intelligence Test<br>• Hamburg-Wechsler Intelligence Test for Children<br><br>**Follow-up**<br>• 10 years<br>• 79% follow-up	**<u>Logistic regression for major impairment vs. normal development or minor impairment at school age</u>**<br><br>**Grade 3-4 IVH or PVL**<br>Normal (n=4, 22%)<br>Minor (n=2, 11%)<br>Major (n=12, 67%)<br>Risk of impairment: OR 2.46 95% CI (0.52–11.7)
20	Piris Borregas 2019[13]<br><br>Spain<br><br>Retrospective cohort study	**Population (n=1001)**<br>• Birthweight 500-1250g<br>• Born 1991-2008<br><br>**Exposure**<br>• Severe brain injury (IVH grade 3-4, ventriculomegaly III, PVL or intraparenchymal echodense lesion grade 3 or greater)<br><br>**Comparator**<br>• Unmatched<br><br>**Ascertainment/ definition**<br>• Neonatal database<br>• Ultrasound diagnosis<br>• Papile classification	**Outcomes**<br>• Neurodevelopment (composite)<br>• Cognitive<br>• Motor<br>• Hearing impairment<br>• Visual impairment<br><br>**Assessment/ measurement**<br>• GMFCS<br><br>**Follow-up**<br>• 7 years	**<u>Poor neurodevelopmental outcome</u>**<br>**Severe brain injury**, n=46, 32%<br>**No severe brain injury**, n=208, 24%<br>OR 1.41 95% CI (0.94, 2.10) p=0.09<br>Independent OR 2.02 95% CI (1.22, 3.31) p=0.18<br><br>**Severe brain injury (birthweight 500-1000g)**<br>Independent OR 2.02 95% CI (1.22, 3.31)
21	Pittet 2019[25]<br><br>Switzerland<br><br>Prospective cohort study	**Population**<br>• Gestation <30 weeks<br>• Born 2006<br><br>**Exposure**<br>• IVH grade 3-4 or cPVL (n=22)<br><br>**Comparator**<br>• Unmatched<br>• No IVH grade 3-4 or cPVL (n=213)<br><br>**Ascertainment/ definition**<br>• Swiss neonatal network follow-up group	**Outcomes**<br>• Cognitive<br>• Cerebral palsy<br>• Visual impairment<br>• Hearing impairment<br><br>**Assessment/ measurement**<br>• Kaufman ABC<br>• Neurological exam<br>• GMFCS<br><br>**Follow-up**<br>• 5.5 – 6 years<br>• 81% follow-up	**<u>Cognitive (K-ABC – MPC score < 1SD)</u>**<br>IVH 3-4 or PVL<br>OR 2.9 95% CI (1, 8.2) p=0.04<br>aOR 2.3 95% CI (0.7, 7.7) p=0.15<br><br>**<u>Use of early intervention/ therapy service</u>**<br>IVH 3-4 or cPVL aOR 2.7 95% CI (1.3, 5.7)
22	Sherlock 2005[14]<br><br>Australia<br><br>Prospective cohort	**Population**<br>• Gestation <28 weeks<br>• Birthweight <1000g<br>• Survivors born 1991-1992<br><br>**Exposure**<br>• IVH Grade 1 (n=47)<br>• IVH Grade 2 (n= 25)<br>• IVH Grade 3 (n= 12)<br>• IVH Grade 4 (n= 6)<br><br>**Comparator**<br>• Matched on sex, mother's country of birth, and health insurance status<br>• Extremely low birth weight or very preterm infants without IVH (n=180)<br><br>**Ascertainment/ definition**<br>• Enrolled in Victorian Collaborative Study<br>• Ultrasound diagnosis (at least one scan by a certified sonographer)<br>• Worst grade of IVH on either side used<br>• Papile classification	**Outcomes**<br>• Disability (composite)<br>• Neurosensory disability (composite)<br>• Cognitive<br>• Motor<br>• Cerebral palsy<br>• Speech and language<br>• Visual impairment<br>• Hearing impairment<br><br>**Measurement/assessment**<br>• Medical assessment<br>• Movement ABC<br>• WISC-III<br>• Tower of London<br>• Rey Complex Figure<br>• WRAT<br><br>**Follow-up**<br>• Mean 8.7 years<br>• 92.3% follow-up	**Abnormal movement**<br>No IVH (n=39, 22.5%)<br>Grade 1 IVH (n=11, 25%)<br>Grade 2 IVH (n=6, 30%)<br>Grade 3 IVH (n=3, 27.3%)<br>Grade 4 IVH (n=4, 100%)<br>X^2 linear trend = 5.3; P = 0.021<br><br>**Cerebral palsy**<br>No IVH (n=12, 6.7%)<br>Grade 1 IVH (n=3, 6.4%)<br>Grade 2 IVH (n=6, 24%)<br>Grade 3 IVH (n=2, 16.7%)<br>Grade 4 IVH (n=6, 100%)<br>X^2 linear trend = 31.7; p <0.0001<br><br>**Moderate to severe cerebral palsy**<br>No IVH (n=4, 2.2%)<br>Grade 1 IVH (n=0, 0%)<br>Grade 2 IVH (n=4, 15%)<br>Grade 3 IVH (n=1, 8.3%)<br>Grade 4 IVH (n=5, 83.3%)<br>X^2 linear trend = 40.8; p <0.0001<br><br>**Major neurosensory disability**<br>No IVH (n=28, 15.6%)<br>Grade 1 IVH (n=5, 10.6%)<br>Grade 2 IVH (n=5, 20%)<br>Grade 3 IVH (n=1, 8.3%)<br>Grade 4 IVH (n=6, 100%)<br>X^2 linear trend = 6.9; p = 0.009<br><br>**IQ score mean (SD)**<br>No IVH 0.71 (1.25)<br>Grade 1 IVH 0.76 (1.32)<br>Grade 2 IVH 0.71 (1.12)<br>Grade 3 IVH 1.21 (1.13)<br>Grade 4 IVH 3.28 (0.88)<br>ANOVA F4,265 = 6.7; p<0.0001<br><br>**Verbal comprehension index mean (SD)**<br>No IVH 96.6 (16.2)<br>Grade 1 IVH 96.3 (15.7)<br>Grade 2 IVH 99.6 (12.8)<br>Grade 3 IVH 93.1 (15.4)

Confidential: For Review Only

				Grade 4 IVH 74.3 (12.7)<br>ANOVA F4,251 = 1.8; p = 0.12<br><br>**Perceptual organisation index mean (SD)**<br>No IVH 98.5 (16.3)<br>Grade 1 IVH 98.2 (15.7)<br>Grade 2 IVH 96.9 (14.8)<br>Grade 3 IVH 91.6 (12.7)<br>Grade 4 IVH 71.7 (11.1)<br>ANOVA F4,249 = 2.5; p = 0.042<br><br>**Freedom from distractibility index mean (SD)**<br>No IVH 92.3 (114.9)<br>Grade 1 IVH 95.5 (15.0)<br>Grade 2 IVH 97.7 (12.8)<br>Grade 3 IVH 94.9 (17.4)<br>Grade 4 IVH 71.0 (3.5)<br>ANOVA F4,250 = 2.8; p = 0.026<br><br>**Processing speed index mean (SD)**<br>No IVH 99.5 (15.8)<br>Grade 1 IVH 99.1 (16.6)<br>Grade 2 IVH 99.3 (13.0)<br>Grade 3 IVH 94.9 (19.3)<br>Grade 4 IVH 71.0 (9.5)<br>ANOVA F4,245 = 2.7; p = 0.033<br><br>**Tower of London (executive function) raw score mean (SD)**<br>No IVH 73.3 (14.4)<br>Grade 1 IVH 71.5 (12.4)<br>Grade 2 IVH 71.1 (20.4)<br>Grade 3 IVH 66.5 (8.3)<br>Grade 4 IVH 54.3 (22.0)<br>ANOVA F4,244 = 1.8; p = 0.13<br><br>**Rey complex figure (executive function) raw score mean (SD)**<br>No IVH 22.5 (7.5)<br>Grade 1 IVH 23.1 (7.4)<br>Grade 2 IVH 24.2 (5.8)<br>Grade 3 IVH 19.3 (8.3)<br>Grade 4 IVH 11.2 (9.8)<br>ANOVA F4,242 = 2.6; p = 0.037<br><br>**Wide range achievements test score mean (SD)**<br>**Reading**<br>No IVH 95.2 (15.7)<br>Grade 1 IVH 102.7 (15.4)<br>Grade 2 IVH 99.0 (14.2)<br>Grade 3 IVH 98.1 (11.9)<br>Grade 4 IVH 70.5g (20.9)<br>ANOVA F4,251 = 5.1; p = 0.001<br><br>**Spelling**<br>No IVH 93.6 (12.4)<br>Grade 1 IVH 97.8 (12.3)<br>Grade 2 IVH 95.9 (10.8)<br>Grade 3 IVH 96.8 (11.9)<br>Grade 4 IVH 73.5 (20.0)<br>ANOVA F4,250 = 4.0; p = 0.003<br><br>**Arithmetic**<br>No IVH 88.3 (14.3)<br>Grade 1 IVH 93.6 (14.9)<br>Grade 2 IVH 92.6 (10.6)<br>Grade 3 IVH 89.1 (10.1)<br>Grade 4 IVH 65.5 (14.5)<br>ANOVA F4,248 = 4.5; p = 0.002<br><br>**Cognitive test scores (compared to normal birthweight controls)**<br>**IQ score <1 SD from the mean (n, %)**<br>No IVH n=64 (35.6%)<br>Grade 1 IVH n=18 (38.3%)<br>Grade 2 IVH n=9 (36%)<br>Grade 3 IVH n=7 (58.3%)<br>Grade 4 IVH n=6(100%)<br>X^2 linear trend=6.8; P=0.009<br><br>**Wide range achievements test score <1 SD from the mean, n (%)**<br>**Low reading**<br>No IVH n=42 (24.4%)<br>Grade 1 IVH n=6 (13.3%)<br>Grade 2 IVH n=5 (20.8%)<br>Grade 3 IVH n=2 (18.2%)<br>Grade 4 IVH n=3 (75%)<br>X^2 linear trend=0.1; p=0.77<br><br>**Low spelling**<br>No IVH n=33 (19.2%)<br>Grade 1 IVH n=6 (13.6%)<br>Grade 2 IVH n=2 (8.3%)<br>Grade 3 IVH n=3 (27.3%)<br>Grade 4 IVH n=3 (75%)<br>X^2 linear trend=0.7; p=0.39<br><br>**Low arithmetic**<br>No IVH n=47 (27.6%)<br>Grade 1 IVH n=9 (20.5%)<br>Grade 2 IVH n=2 (8.3%)<br>Grade 3 IVH n=3 (27.3%)<br>Grade 4 IVH n=4 (100%)<br>X^2 linear trend=0.1; p=0.79

Confidential: For Review Only

23	Tymofiyeva 2018[33]<br><br>USA<br><br>Prospective cohort	**Population (n=24)**<br>• Gestation < 33 weeks<br><br>**Exposure**<br>• Mild WMI (n=4)<br>• Moderate WMI (n=5)<br>• Severe WMI (n=1)<br><br>• IVH grade 1 (n=5)<br>• IVH grade 2 (n=0)<br>• IVH grade 3 (n=0)<br>• IVH grade 4 (n=0)<br><br>**Comparator**<br>• Unmatched<br>• No WMI (n=14)<br>• No IVH (n=19)<br><br>**Ascertainment/ definition**<br>• MRI imaging reviewed by a blinded paediatric neuroradiologist<br>• Used own classification of white matter injury<br>• Papile classification	**Outcome**<br>• Cognitive<br>• Behaviour<br><br>**Assessment/ measurement**<br>• Test of variables of attention<br>• Conners comprehensive behaviour rating scales<br>• CBCL<br>• Assessment undertaken by a blinded psychologist<br>• Parental questionnaire<br><br>**Follow-up**<br>• 10-14 years<br>• Completeness not specified	**<u>Attention (abnormal)</u>**<br>Mild WMI n=3, 75%<br>Moderate WMI n=0, 0%<br>No WMI n=8, 57% p=0.05
24	Van de Bor 2004[15]<br><br>Netherlands<br><br>Prospective cohort	**Population**<br>• Gestation < 32 weeks<br>• Birthweight < 1500 g<br>• Born 1983<br><br>**Exposure**<br>• IVH grade 1-2 (n=45)<br>• IVH grade 3-4 (n=17)<br><br>**Comparator (n=216)**<br>• Unmatched<br>• No IVH<br><br>**Ascertainment/ definition**<br>• Ultrasound diagnosis<br>• Papile classification	**Outcomes**<br>• Disability (composite)<br>• Cognitive<br>• Neurological status (motor)<br>• Speech and language<br>• Behaviour<br>• Hearing<br>• Vision<br>•<br>**Measurement/assessment**<br>• Questionnaires (completed by parents at 9 years; adolescents at 14 years)<br>• Home visit and neurodevelopmental assessment by paediatrician unaware of medical history<br>• WHO classification of impairment, disability, and handicap<br><br>**Follow-up**<br>• 5, 9 and 14 years<br>91.5% follow-up of survivors at 14 years	**<u>Disability at 5 years</u>**<br>No IVH n=49 (23%)<br>IVH grade 3-4 n=5 (31.3%)<br><br>**Cognitive disability**<br>No IVH n=18 (8.3%)<br>IVH grade 3-4 n=1 (5.9%) p=not significant<br><br>**Motor disability**<br>No IVH n=8 (3.7%)<br>IVH grade 3-4 n=3 (17.6%) p=0.00<br><br>**Speech/language disability**<br>No IVH n=34 (15.7%)<br>IVH grade 3-4 n=1 (5.9%) p= not significant<br><br>**Visual disability**<br>No IVH n=1 (0.5%)<br>IVH grade 3-4 n=0 p= not significant<br><br>**Hearing disability**<br>No IVH n=5 (2.3%)<br>IVH grade 3-4 n=0 p= not significant<br><br><br>**<u>School performance at 5 years</u>**<br>**Special education**<br>No IVH n=17 (8.7%)<br>IVH grade 3-4 n=3 (20%) p=0.02<br><br>**<u>School performance at 9 years</u>**<br>**Slow learner**<br>No IVH n=57 (29.5%)<br>IVH grade 3-4 n=4 (26.7%)<br><br>**Special education**<br>No IVH n=29 (15%)<br>IVH grade 3-4 n=4 (26.7%) p=0.04<br><br>**<u>School performance at 14 years</u>**<br>**Slow learner**<br>No IVH n=93 (44.1)<br>IVH grade 3-4 n=4 (23.5%)<br><br>**Special education**<br>No IVH n=26 (12%)<br>IVH grade 3-4 n=6 (35.3%) p=0.00<br><br>**Need for special education at 14 years**<br>IVH (all grades)<br>OR 2.56 95%CI (1.17-4.86)<br>aOR 2.33 95%CI (1.15, 4.75)<br><br>IVH grade 3-4<br>aOR 3.99 95%CI (1.36, 11.69)
25	Van Den Hout 2000[26]<br><br>Netherlands<br><br>Prospective cohort	**Population**<br>• Mean gestation 28-30 weeks<br>• Born 1989-1991<br><br>**Exposure**<br>• IVH (n=17)<br>• PVL (n=12)<br><br>**Comparator (n=17)**<br>• Preterm<br>• Normal cranial ultrasound<br><br>**Ascertainment/ definition**<br>• Ultrasound diagnosis<br>• Modified Levene and DeVries classification for IVH<br>• DeVries classification for PVL	**Outcomes**<br>• Cognitive<br>• Visual acuity<br><br>**Measurement/ assessment**<br>• L94 visual-perceptual ability test<br>• Grating acuity cards<br>• McCarthy scales of children's abilities<br>• Wechsler preschool and primary scale of intelligence<br>• Snijders-Oomen non-verbal intelligence test<br>• Leiden Diagnostic test<br><br>**Follow-up**<br>• Mean 5.3 years<br>• 88% follow-up	**Total intelligence quotient, mean (SD)**<br>IVH 92.4 (16.3)<br>PVL 79.6 (20.5)<br>No brain injury 102.8 (14.4)<br><br>**IQ <85**<br>IVH n=6, 35.3%<br>PVL n=6, 50%<br>No brain injury n=2, 11.8%<br><br>**Performance age in years, mean (SD)**<br>IVH 5.22 (1.16)<br>PVL 4.37 (1.19)<br>No brain injury 6.22 (0.89)<br><br>**Visual grating acuity in c/deg, mean (SD)**<br>IVH 37.4 (13.5)<br>PVL 33.5 (15.9)

Confidential: For Review Only

				No brain injury 47.1 (13.5)<br><br>**Visual grating acuity <25c/deg (%)**<br>IVH (11.8)<br>PVL (33.3)<br>No brain injury (0)<br><br>**Impairment on each of the eight L94 tasks**<br>**Visual matching % (n)**<br>IVH 0 (17)<br>PVL 0 (12)<br>No brain injury 5.9 (17)<br><br>**Unconventional Object Views % (n)**<br>IVH 29.4 (17)<br>PVL 41.7 (12)<br>No brain injury 17.6 (17)<br><br>**De Vos task % (n)**<br>IVH 29.4 (17)<br>PVL 41.7 (12)<br>No brain injury 11.8 (17)<br><br>**Line Drawings Occluded by Noise% (n)**<br>IVH 6.3 (16)<br>PVL 36.4 (11)<br>No brain injury 0 (17)<br><br>**Line Drawings Occluded by Noise% (n)**<br>IVH 13.3 (15)<br>PVL 25.0 (8)<br>No brain injury 5.9 (17)<br><br>**Developmental test of visual motor integration % (n)**<br>IVH 0 (16)<br>PVL 0 (7)<br>No brain injury 0 (17)<br><br>**Matching block designs % (n)**<br>IVH 5.9 (17)<br>PVL 20.0 (10)<br>No brain injury 17.6 (17)<br><br>**Constructing block designs% (n)**<br>IVH 30.8 (13)<br>PVL 80.0 (5)<br>No brain injury 31.3 (16)<br><br>**Mean percentage of L94 tasks on which child is impaired (mean, SD; %)**<br>IVH 14.71 (17.81)<br>PVL 32.04 (24.64)<br>No brain injury 11.13 (9.79)
26<br>*	Vollmer 2003[16]<br><br>UK<br><br>Prospective cohort	**Population**<br>• Gestation <33 weeks<br>• Born 1983-1988<br><br>**Exposure**<br>• IVH (n=159)<br>• Ventricular dilatation (n=32)<br>• IVH, PV flare, ventricular dilatation (n=164)<br>• Hydrocephalus (n=36)<br>• Haemorrhagic parenchymal infarction (HPI) (n=61)<br>• cPVL n=26<br><br>**Comparator (n=348)**<br>• Unmatched<br>• Normal scan<br><br>**Ascertainment/ definition**<br>• Ultrasound imaging reviewed by two experienced observers<br>• In-house classification used	**Outcomes**<br>• Neurodevelopmental impairment (composite)<br>• Visual impairment<br>• Hearing impairment<br><br>**Measurement/ assessment**<br>• Structured neurologic examination<br>• Pure-tone audiogram<br>• Vision test (Snellen chart)<br>• Henderson-Stott TOMI<br>• Beery test of VMI<br>• WISC-R for children born 1983-1986<br>• WISC-III for children born 1987-1988<br><br>**Follow-up**<br>• 8 years<br>• 91.7% follow-up	**Neurodevelopmental status**<br>**Group A (<28 weeks)**<br>**All impairments (n,%)**<br>GMH/IVH (5, 18%)<br>Ventricular dilatation (4, 50%)<br>GMH/IVH, flare, ventricular dilatation (19, 51%)<br>Hydrocephalus (7, 78%)<br>HPI (15, 100%)<br>cPVL (4, 100%)<br>No brain injury (12, 32%)<br><br>**Disabling impairments (n, %)**<br>GMH/IVH (1, 4%)<br>Ventricular dilatation (0, 0%)<br>GMH/IVH, flare, ventricular dilatation (9, 24%)<br>Hydrocephalus (7, 78%)<br>HPI (14, 93%)<br>cPVL (3, 75%)<br>No brain injury (3, 8%)<br><br>**Group B (28-32 weeks)**<br>**All impairments (n, %)**<br>GMH/IVH (16, 29%)<br>Ventricular dilatation (5, 31%)<br>GMH/IVH, flare, ventricular dilatation (30, 43%)<br>Hydrocephalus (7, 54%)<br>HPI (5, 83%)<br>cPVL (9, 75%)<br>No brain injury (67, 29%)<br><br>**Disabling impairments (n, %)**<br>GMH/IVH (5, 5%)<br>Ventricular dilatation (1, 6%)<br>GMH/IVH, flare, ventricular dilatation (16, 23%)<br>Hydrocephalus (6, 46%)<br>HPI (3, 50%)<br>cPVL (6, 50%)<br>No brain injury (14, 6%)
27<br>*	Vollmer 2006a[21]<br><br>UK<br><br>Prospective cohort	**Population**<br>• Gestation <33 weeks<br>• Born 1985-1991<br><br>**Exposure**<br>• Bilateral brain lesions (n=201)<br>• Right-sided brain lesion (n=41)	**Outcomes**<br>• Motor<br>• Cognitive<br>• Cerebral palsy<br>• Visual	**TOMI error score, mean (SD)**<br>Normal scan 2.78 (2.1)<br><br>All left-sided lesions 4.3 (3.5)<br>Left-sided non-parenchymal lesions 4.5 (3.8)<br>Left-sided parenchymal lesions 3.7 (2.1)

Confidential: For Review Only

		• Left-sided brain lesion (n=57)<br><br>Brain lesion types<br>Non-parenchymal:<br>• Uncomplicated IVH<br>Parenchymal:<br>• Haemorrhagic parenchymal infarction (HPI)<br>• cPVL<br>• PV flare<br><br>**Comparator (n=369)**<br>• Unmatched<br>• Normal ultrasound<br><br>**Ascertainment/ definition**<br>• Ultrasound imaging reviewed by two experienced observers<br>• Modified Stewart classification	**Measurement/ assessment**<br>• Neurological examination (modified Amiel-Tison assessment)<br>• TOMI<br>• WISC-R<br>• Test of VMI<br><br>**Follow-up**<br>• 8 years<br>• 80% follow-up	All right-sided lesions 3.5 (2.9)<br>Right-sided non-parenchymal lesions 2.7 (1.8)<br>Right-sided parenchymal lesions 4.9 (3.8)<br><br>All bilateral lesions 4.5 (4.3)<br>Bilateral non-parenchymal lesions 4.1 (3.7)<br>Bilateral parenchymal lesions 4.9 (4.7)<br><br>ANOVA for parenchymal lesions only p <0.0001<br>ANOVA including parenchymal and non-parenchymal lesions p <0.0001<br>ANOVA excluding parenchymal lesions, p <0.0001<br><br>**VMI centile, mean (SD)**<br>Normal scan 59.2 (30.0)<br><br>All left-sided lesions 40.3 (30.1)<br>Left-sided non-parenchymal lesions 46.8 (31.0)<br>Left-sided parenchymal lesions 21 (22)<br><br>All right-sided lesions 60.2 (31.9)<br>Right-sided non-parenchymal lesions 64.2 (30.2)<br>Right-sided parenchymal lesions 54 (35)<br><br>All bilateral lesions 46.0 (33.5)<br>Bilateral non-parenchymal lesions 55.1 (32.1)<br>Bilateral parenchymal lesions 38 (32)<br><br>ANOVA for parenchymal lesions only p <0.0001<br>ANOVA including parenchymal and non-parenchymal lesions p <0.0001<br>ANOVA excluding parenchymal lesions reported as both p <0.0001 and p=0.98 Ω(potential error in the manuscript table)<br><br>**Cerebral palsy, n (%)**<br>Normal scan 2 (0.7%)<br><br>All left-sided lesions 4 (9%)<br>Left-sided non-parenchymal lesions 2 (6%)<br>Left-sided parenchymal lesions 2 (16%)<br><br>All right-sided lesions 2 (6%)<br>Right-sided non-parenchymal lesions 1 (4%)<br>Right-sided parenchymal lesions 1 (8%)<br><br>All bilateral lesions 37 (21%)<br>Bilateral non-parenchymal lesions 8 (10%)<br>Bilateral parenchymal lesions 29 (31%)<br><br>Chi-square for parenchymal and non-parenchymal lesions, p <0.0001<br>Chi-square excluding parenchymal lesions, p <0.0001<br>Chi-square for parenchymal lesions only, p <0.0001<br>ANOVA parenchymal lesions only, p <0.0001<br><br>**Full scale IQ, mean (SD)**<br><br>Normal scan 101 (16)<br><br>All left-sided lesions 93 (17)<br>Left-sided non-parenchymal lesions 98 (15)<br>Left-sided parenchymal lesions 80 (15)<br><br>All right-sided lesions 102 (17)<br>Right-sided non-parenchymal lesions 104 (15)<br>Right-sided parenchymal lesions 100 (19)<br><br>All bilateral lesions 91 (21)<br>Bilateral non-parenchymal lesions 96(19)<br>Bilateral parenchymal lesions 86 (22)<br><br>ANOVA for parenchymal lesions only, p <0.0001.<br>ANOVA including parenchymal and non-parenchymal lesions, p <0.0001.<br>ANOVA excluding parenchymal lesions, p =0.137.<br><br>**Verbal IQ, mean (SD)**<br>Normal scan 103 (19)<br><br>All left-sided lesions 98 (20)<br>Left-sided non-parenchymal lesions 102 (20)<br>Left-sided parenchymal lesions 85 (18)<br><br>All right-sided lesions 107 (18)<br>Right-sided non-parenchymal lesions 108 (16)<br>Right-sided parenchymal lesions 107 (22)<br><br>All bilateral lesions 96 (23)<br>Bilateral non-parenchymal lesions 100 (20)<br>Bilateral parenchymal lesions 91 (25)<br><br>ANOVA for parenchymal lesions only, p <0.0001<br>ANOVA including parenchymal and non-parenchymal lesions, p <0.0001<br>ANOVA excluding parenchymal lesions, p =0.38<br><br>**Performance IQ, mean (SD)**<br>Normal scan 96 (15)<br><br>All left-sided lesions 86 (16)<br>Left-sided non-parenchymal lesions 90 (15)<br>Left-sided parenchymal lesions 76 (15)

Confidential: For Review Only

				All right-sided lesions 95 (16)<br>Right-sided non-parenchymal lesions 98 (13)<br>Right-sided parenchymal lesions 92 (19)<br><br>All bilateral lesions 85 (22)<br>Bilateral non-parenchymal lesions 91 (20)<br>Bilateral parenchymal lesions 80 (21)<br><br>ANOVA for parenchymal lesions only, p <0.0001<br>ANOVA including parenchymal and non-parenchymal lesions, p <0.0001<br>ANOVA excluding parenchymal lesions, p =0.59
28<br>*	Vollmer 2006b[27]<br><br>UK<br><br>Prospective cohort	**Population**<br>• Gestation <33 weeks<br>• Born 1979-1991<br><br>**Exposure (n=66)**<br>• Ventricular dilatation and IVH<br><br>**Comparator (n=616)**<br>• Unmatched<br>• Normal cranial ultrasound<br><br>**Ascertainment/ definition**<br>• Ultrasound imaging reviewed by two experienced observers<br>• In-house classification used	**Outcomes**<br>• Neurological impairment with or without disability (composite)<br>• Cognitive<br>• Motor<br>• Vision<br><br>**Measurement/ assessment**<br>• Structured neurological exam<br>• TOMI<br>• Test of VMI<br>• WISC<br><br>**Follow-up**<br>• 8 years<br>• 81% follow-up	<u>**Disabling motor impairment, n (%)**</u><br>Ventricular dilatation and IVH n=10 (16%)<br>Normal ultrasound n=10 (2%)<br><br><u>**Cognitive**</u><br>**Full scale IQ, mean (SD)**<br>Ventricular dilatation and IVH 96 (23)<br>Normal ultrasound 101 (17)<br><br>**Verbal IQ, mean (SD)**<br>Ventricular dilatation and IVH 101 (22)<br>Normal ultrasound 104 (19)<br><br>**Performance IQ mean (SD)**<br>Ventricular dilatation and IVH 97 (15)<br>Normal ultrasound 91 (21)<br><br><u>**Motor and vision**</u><br>**VMI centile, mean (SD)**<br>Ventricular dilatation and IVH 37 (33)<br>Normal ultrasound 52 (31)<br><br>**TOMI, mean (SD)**<br>Ventricular dilatation and IVH 5.98 (4.2)<br>Normal ultrasound 3.26 (2.5)
29	Whitaker 2011[30]<br><br>USA<br><br>Prospective cohort	**Population**<br>• Birthweight <2000g<br>• 'Non-disabled' survivors<br>• Born 1984-1987<br><br>**Exposure**<br>• IVH (n=69)<br>• Parenchymal lesions and/or ventricular enlargement (n=21)<br><br>**Comparison (n=368)**<br>• Unmatched<br>• Normal cranial ultrasound<br><br>**Ascertainment/ definition**<br>• Ultrasound imaging reviewed by three blinded radiologists independently, disagreements resolved through consensus and inter-observer reliability checked.<br>• Paneth classification	**Outcomes**<br>• Mental health conditions<br><br>**Measurement/ assessment**<br>• Parent report version of the Diagnostic Interview Schedule for Children–IV<br>• WASI<br><br>**Follow-up**<br>• 16 years<br>• 72.9% follow-up	<u>**Logistic regression assessing odds of current and lifetime mental health conditions after brain injury**</u><br><br><u>**Current ADHD- inattentive type**</u><br>**IVH**<br>OR 0.97 95% CI (0.21-4.47)<br>aOR 1.01 95% CI (0.19-5.44)<br><br>**Parenchymal lesions and/or ventricular enlargement**<br>OR 7.64[b] 95% CI (2.20-24.48)<br>aOR 6.83[c] 95% CI (1.26-36.91)<br><br><u>**Lifetime ADHD – inattentive type**</u><br>**IVH**<br>OR 0.83 95% CI (0.34-2.04)<br>aOR 0.64 95% CI (0.24-1.74)<br><br>**Parenchymal lesions and/or ventricular enlargement**<br>OR 2.71 95% CI (0.94-7.82)<br>aOR 1.13 95% CI (0.31-4.10)<br><br><u>**Current major depression**</u><br>**IVH**<br>OR 2.66 95% CI (1.04-6.78)<br>aOR 2.23 95% CI (0.80-6.24)<br><br><u>**Lifetime major depression**</u><br>**IVH**<br>OR 2.76 95% CI (1.19-6.38)<br>aOR 2.59 95% CI (1.02-6.58)<br><br><u>**Current tic disorders**</u><br>**IVH**<br>OR 1.63 95% CI (0.44-6.07)<br>aOR 1.89 95% CI (0.42-8.57)<br><br>**Parenchymal lesions and/or ventricular enlargement**<br>OR 8.42 95% CI (2.40-29.62)<br>aOR 9.77 95% CI (1.69-56.47)<br><br><u>**Lifetime tic disorders**</u><br>**IVH**<br>OR 0.95 95% CI (0.27-3.34)<br>aOR 0.85 95% CI (0.21-3.51)<br><br>**Parenchymal lesions and/or ventricular enlargement**<br>OR 5.07 95% CI (1.53-16.82)<br>aOR 5.02 95% CI (1.05-23.92)<br><br><u>**Current obsessive-compulsive disorder**</u><br>**IVH**<br>OR 9.52 95% CI (3.02-30.06)<br>aOR 11.85 95% CI (3.22-43.62)<br><br>**Parenchymal lesions and/or ventricular enlargement**<br>OR 7.64 95% CI (1.39-41.98)<br>aOR 15.32 95% CI (1.82-128.74)<br><br><u>**Lifetime obsessive compulsive disorder**</u><br>**IVH**<br>OR 9.52 95% CI (3.05-30.06)<br>aOR 11.85 95% CI (3.22-43.62)

Confidential: For Review Only

				**Parenchymal lesions and/or ventricular enlargement**<br>OR 7.64 95% CI (1.39-41.98)<br>aOR 15.32 95% CI (1.82-128.74)<br><br>**Current diagnoses additionally controlled for full score IQ and motor function**<br><br>**ADHD inattentive type**<br>**IVH**<br>OR 0.86 95% CI (0.18-3.99)<br>aOR 0.99 95% CI (0.21-4.62)<br><br>**Parenchymal lesions and/or ventricular enlargement**<br>OR 5.04 95% CI (1.36-18.65)<br>aOR 5.43 95% CI (1.32-22.40)<br><br>**Major depression**<br>**IVH**<br>OR 0.43 95% CI (0.16-1.11)<br>aOR 0.40 95% CI (0.15-1.05)<br><br>**Tic disorders**<br>**IVH**<br>OR 1.54 95% CI (0.41-5.78)<br>aOR 1.45 95% CI (0.38-5.48)<br><br>**Parenchymal lesions and/or ventricular enlargement**<br>OR 7.01 95% CI (1.88-28.14)<br>aOR 4.38 95% CI (1.05-18.23)<br><br>**Obsessive compulsive disorder**<br>**IVH**<br>OR 8.68 95% CI (2.72-27.69)<br>aOR 10.91 95% CI (3.13-37.99)<br><br>**Parenchymal lesions and/or ventricular enlargement**<br>OR 4.78 95% CI (0.83-28.10)<br>aOR 3.58 95% CI (0.50-25.94)
Perinatal stroke				
30	Ballantyne * 2007 [41]<br>USA<br><br>Prospective cohort	**Population**<br>• Mean gestation 38.5 weeks<br>• Born 1991-2001<br><br>**Exposure (n=28)**<br>• Left lesions (n=17)<br>• Right lesions (n=11)<br><br>**Comparator (n=57)**<br>• Unmatched<br>• Healthy controls with normal medical and developmental histories<br>• Recruited from the community<br><br>**Ascertainment/ definition**<br>• Single unilateral lesions the result of perinatal strokes occurring between 28 weeks' gestation and 28 days after birth; infarct or haemorrhage<br>• Identified through medical history and neuroimaging<br>• Severity rated on a 5-point scale adapted from the Vargha-Khadem classification	**Outcomes**<br>• Speech and language<br><br>**Assessment/ measurement**<br>• CELF-R<br>• Wechsler Intelligence Scales (WPPSI-R, WISC-R, or WISC-III)<br>• PPVT–Revised<br>• Expressive One-Word Picture Vocabulary Test–Revised or Upper-Extension<br>• Total Language Standard Scores<br><br>**Follow-up**<br>• 6-9 years<br>• 100% follow-up	**Speech and language**<br>**CELF-R Receptive, mean (SD)**<br>All strokes: 82.54 (17.12) p<.0001<br>Left stroke: 83.18 (16.66) p<.0001<br>Right stroke: 81.55 (18.59) p=0.001<br>Control: 106.37 (12.51)<br><br>**CELF-R Expressive mean (SD)**<br>All strokes: 73.75 (16.79) p<.0001<br>Left stroke: 73.06 (14.88) p<.0001<br>Right stroke: 74.82 (20.11) p=0.001<br>Control: 101.02 (13.63)<br><br>**CELF-R Total mean (SD)**<br>All strokes: 76.93 (17.31) p<.0001<br>Left stroke: 76.94 (15.39) p<.0001<br>Right stroke: 76.91 (20.74) p=0.001<br>Control: 104.00 (12.58)<br>.
31	Ballantyne 2008[34] *<br><br>USA<br><br>Prospective cohort	**Population**<br>• 32- 40 weeks' gestation<br>• Birth years not reported<br><br>**Exposure (n=29)**<br>• Left hemisphere (n=20)<br>• Right hemisphere (n=9)<br><br>**Control (n=38)**<br>• Healthy controls (normal neurodevelopment)<br>• Recruited through a university and community adverts<br><br>**Ascertainment/ definition**<br>• Unilateral ischaemic perinatal stroke confirmed through clinical history and neuroimaging<br>• Lesion location and severity reviewed by blinded neuroradiologist<br>• Severity rated on a 5-point scale adapted from the Vargha-Khadem classification	**Outcomes**<br>• Cognitive (academic skills)<br>• Speech and language<br>• Motor<br>• Cerebral palsy<br>• Vision<br>• Epilepsy<br><br>**Measurement/ assessment**<br>• WISC- Revised<br>• WRAT- Revised<br>• CELF- Revised<br>• PPVT-Revised<br>• WPPSI/WPPSI- Revised<br>• WISC-III<br><br>**Follow-up**<br>• 7-12 years<br>• 100% follow up	**Hemiparesis**<br>Stroke n=18,62%<br><br>**Visual field deficit**<br>Stroke n=7, 26%<br><br>**Seizures**<br>Stroke n=11, 38%<br><br>**Cognitive, mean (SD)**<br>**Verbal IQ (WISC-R)**<br>**Time point 1 (mean age 7-8 years)**<br>Stroke 96.6 (20.5)<br>Control 126.1 (16)<br><br>**Time point 2 (mean age 10 – 12 years)**<br>Stroke 98.7 (20)<br>Control 123.6 (13.1)<br>Between group affect (stroke vs. control) p<0.0001<br>Time effect not significant<br><br>**Performance IQ (WISC-R)**<br>**Time point 1 (mean age 7-8 years)**<br>Stroke 92.8 (19.9)<br>Control 115.2 (13.8)<br><br>**Time point 2 (mean age 10 – 12 years)**<br>Stroke 93.5 (20)<br>Control 116 (10.5)<br>Between group affect (stroke vs. control) p=0.002<br>Time effect not significant<br><br>**Full scale IQ (WISC-R)**

Confidential: For Review Only

				**Time point 1 (mean age 7-8 years)**<br>Stroke 94.7 (20.4)<br>Control 123 (15)<br><br>**Time point 2 (mean age 10 – 12 years)**<br>Stroke 96.1 (19.1)<br>Control 122.3 (10.2)<br><br>Between group affect (stroke vs. control) p<0.0001<br>Time effect not significant<br><br>**Reading (WRAT -R)**<br>**Time point 1 (mean age 7-8 years)**<br>Stroke 85 (16.1)<br>Control 113 (13.3)<br><br>**Time point 2 (mean age 10 – 12 years)**<br>Stroke 89.4 (13.3)<br>Control 108.9 (13.8)<br><br>Between group affect (stroke vs. control) p<0.0001<br>Time effect not significant<br>Time group interaction p=0.045<br><br>**Spelling (WRAT -R)**<br>**Time point 1 (mean age 7-8 years)**<br>Stroke 82.5 (18.2)<br>Control 106.2 (15.9)<br><br>**Time point 2 (mean age 10 – 12 years)**<br>Stroke 87 (16.8)<br>Control 104.6 (13.1)<br><br>Between group affect (stroke vs. control) p=0.001<br>Time effect not significant<br><br>**Arithmetic (WRAT -R)**<br>**Time point 1 (mean age 7-8 years)**<br>Stroke 91.5 (10.2)<br>Control 111.9 (11.2)<br><br>**Time point 2 (mean age 10 – 12 years)**<br>Stroke 94.2 (18.7)<br>Control 113.1 (16.2)<br><br>Between group affect (stroke vs. control) p<0.0001<br>Time effect not significant<br><br>**Speech and language**<br>**Receptive language score**<br>**Time point 1 (mean age 7-8 years)**<br>Stroke 84.2 (10.9)<br>Control 109.1 (12.2)<br><br>**Time point 2 (mean age 10 – 12 years)**<br>Stroke 82.3 (20.1)<br>Control 111.4 (13.7)<br><br>Between group affect (stroke vs. control) p<0.0001<br>Time effect not significant<br><br>**Expressive language score**<br>**Time point 1 (mean age 7-8 years)**<br>Stroke 72.5 (12)<br>Control 101 (17.5)<br><br>**Time point 2 (mean age 10 – 12 years)**<br>Stroke 78.4 (16)<br>Control 105.8 (11.9)<br><br>Between group affect (stroke vs. control) p<0.0001<br>Time effect p=0.017<br><br>**Total language score**<br>**Time point 1 (mean age 7-8 years)**<br>Stroke 76.9 (11.1)<br>Control 105.6 (14.2)<br><br>**Time point 2 (mean age 10 – 12 years)**<br>Stroke 79.1 (18.3)<br>Control 109.8 (14)<br><br>Between group affect (stroke vs. control) p<0.0001<br>Time effect not significant<br><br>**Vocabulary score**<br>**Time point 1 (mean age 7-8 years)**<br>Stroke 97.5 (19.7)<br>Control 117.1 (17)<br><br>**Time point 2 (mean age 10 – 12 years)**<br>Stroke 99.9 (20)<br>Control 118.9 (13.9)<br><br>Between group affect (stroke vs. control) p=0.002<br>Time effect not significant
32	Gold 2014[35]<br><br>USA	**Population**<br>• Gestation not provided<br>• Birth years not provided	**Outcomes**<br>• Cognitive (IQ and memory)<br>• Motor<br>• Cerebral palsy	**Cognitive**<br>**Memory**<br>**Stories immediate recall**<br>Controls, mean (SE)13.5 (0.7)

Confidential: For Review Only

	Prospective cohort	**Exposure (n=27)**<br>• Right-sided stroke (n=12)<br>• Left-sided stroke (n=15)<br><br>**Comparator (n=19)**<br>• Matched for age at follow up, sex, socioeconomic group and maternal education<br>• Healthy controls<br>• Recruited through local advertising<br><br>**Ascertainment/ definition**<br>• Single, unilateral brain lesion in an arterial vascular distribution, either identified in the neonatal period with neuroimaging, or identified later in infancy after presentation with a hemiparesis and imaging documentation of an old unilateral infact (presumed perinatal stroke)<br>• Recruited from paediatric neurology clinics<br>• Severity graded 1-5 using Trauner/ Vargha-Khaldem classification	**Measurement/ assessment**<br>• WISC-III<br>• Dots and Stories subtests of the Children's Memory Scales<br><br>**Follow-up**<br>• 6-16 years<br>• 100% follow-up	Stroke, mean (SE) 8.4 (0.8) p<0.001<br><br>Stroke and seizures, mean (SE)7 (0.8)<br>Stroke and no seizures, mean (SE) 10.1 (1.4) p=0.06<br><br>Right lesion, mean (SE) 7.8 (1.1)<br>Left lesion, mean (SE) 8.9 (1.2) p=0.51<br><br>**Delayed recall**<br>Controls, mean (SE) 13.9 (0.8)<br>Stroke, mean (SE) 7.9 (0.8) p<0.001<br><br>Stroke and seizures, mean (SE) 6.2 (0.9)<br>Stroke and no seizures, mean (SE) 10 (1.2) p=0.02<br><br>Right lesion, mean (SE) 7.3 (1.1)<br>Left lesion, mean (SE) 8.3 (1.2) p=0.56<br><br>**Delayed recognition**<br>Controls, mean (SE) 11.5 (0.5)<br>Stroke, mean (SE) 8 (0.8) p=0.001<br><br>Stroke and seizures, mean (SE) 7.1 (1.1)<br>Stroke and no seizures, mean (SE) 9.2 (0.9) p=0.17<br><br>Right lesion, mean (SE) 8.3 (1.4)<br>Left lesion, mean (SE) 7.9 (0.9) p=0.8<br><br>**Dots learning**<br>Controls, mean (SE) 10.9 (0.5)<br>Stroke, mean (SE) 8.9 (0.8) p=0.05<br><br>Stroke and seizures, mean (SE) 7.6 (1.1)<br>Stroke and no seizures, mean (SE) 10.6 (0.8) p=0.05<br><br>Right lesion, mean (SE) 9.3 (1.4)<br>Left lesion, mean (SE) 8.7 (0.9) p=0.71<br><br>**Total**<br>Controls, mean (SE) 11.8 (0.5)<br>Stroke, mean (SE) 9 (0.7) p=0.003<br><br>Stroke and seizures, mean (SE) 7.8 (0.9)<br>Stroke and no seizures, mean (SE) 10.6 (0.9) p=0.04<br><br>Right lesion, mean (SE) 9.2 (0.7)<br>Left lesion, mean (SE) 10.2 (0.7) p=0.62<br><br>**Delayed recall**<br>Controls, mean (SE) 12.6 (0.4)<br>Stroke, mean (SE) 10 (0.5) p<0.001<br><br>Stroke and seizures, mean (SE) 8.8 (0.5)<br>Stroke and no seizures, mean (SE) 11.4 (0.8) p=0.009<br><br>Right lesion, mean (SE) 9.7 (0.7)<br>Left lesion, mean (SE) 10.2 (0.7) p=0.62<br><br>**WISC- III IQ, mean (SD)**<br>Right stroke, 85.0 (6)<br>Left stroke, 91 (6) p=0.49<br><br>**IQ scores**<br>Controls 117 (2.7)<br>All stroke patients 88 (4.0) p<0.001<br>No seizures 100 (6.4)<br>Seizures 78 (3.7)<br><br>**Motor (hemiparesis)**<br>Stroke patients n=16; 59%<br>Control n=0; p=0.05
33	Kolk 2011[36]<br><br>Estonia<br><br>Retrospective cohort	**Population**<br>• Gestation not provided<br>• Born 1995-2006<br><br>**Exposed (n=21)**<br>• Neonatal stroke<br><br>**Control (n=31)**<br>• Matched on age and sex<br>• Healthy children<br>• Recruited locally<br><br>**Ascertainment/ definition**<br>• Estonian stroke registry<br>• Arterial ischaemic stroke or haemorrhagic	**Outcomes**<br>• Cognitive<br>• Neuropsychological<br>• Motor<br>• Cerebral palsy<br>• Speech and language<br>• Epilepsy<br><br>**Measurement/ assessment**<br>• NEPSY<br>• Kaufman ABC<br>• Paediatric Stroke Outcome Measure<br><br>**Follow-up**<br>• 4-10 years<br>• 100% follow-up	**Neuromotor impairment (Paediatric Stroke Outcome Measure)**<br>**Neonatal stroke**<br>Severe n=4, 19%<br>Moderate n=9, 43%<br>Good n=6, 28.6%<br>Normal n=2, 9.5%<br><br>**Cognitive/ neuropsychological**<br><br>**Attention and executive function, mean, SD, 95% CI**<br>**Tower**<br>Control 0.22, 0.64 (-0.05, 0.48)<br>Neonatal stroke -0.34, 1.34 (-1.03, 0.35) p=0.142<br><br>**Auditory attention**<br>Control 0.27, 0.72 (-0.03, 0.57)<br>Neonatal stroke -0.38, 1.10 (-1.04, 0.28) p=0.009<br><br>**Visual attention: time**<br>Control 0.37, 0.81, (0.07, 0.67)<br>Neonatal stroke -0.40, 0.93 (-0.82, 0.03) p=0.004<br><br>**Visual attention: correct**<br>Control 0.48, 0.50 (0.30, 0.67)<br>Neonatal stroke -0.54, 0.97 (0.98, 0.1) p<0.0001<br><br>**Statue**

Confidential: For Review Only

				Control 0.26, 0.77 (-0.03, 0.54)<br>Neonatal stroke -0.23, 1.09, (-0.73, 0.28) p=0.086<br><br>**Design fluency**<br>Control 0.18, 1.04 (-0.25, 0.61)<br>Neonatal stroke -0.36, 0.70 (-0.78, 0.06) p=0.06<br><br>**Knock and tap**<br>Control 0.31, 0.50 (0.10, 0.51)<br>Neonatal stroke -0.44, 1.52, (-1.32, 0.43) p==0.03<br><br>**Language, mean, SD, 95% CI**<br>**Phonological processing**<br>Control 0.24, 0.80 (-0.05, 0.54)<br>Neonatal stroke -0.38, 0.99 (-0.83, 0.08) p=0.001<br><br>**Comprehension of instructions**<br>Control 0.43, 0.70 (0.18, 0.69)<br>Neonatal stroke -0.59 1.06 (-1.07, 0.11) p<0.0001<br><br>**Speeded naming: time**<br>Control 0.24, 0.70 (-0.05, 0.52)<br>Neonatal stroke -0.14, 1.03 (-0.73, 0.46) p=0.188<br><br>**Speeded naming: correct**<br>Control 0.42, 0.41 (0.25, 0.59)<br>Neonatal stroke -0.45, 1.41 (-1.26, 0.37) p=0.008<br><br>**Repetition of nonsense words**<br>Control 0.30, 0.53 (0.08, 0.52)<br>Neonatal stroke -0.40, 1.23 (-1.03, 0.24) p=0.026<br><br>**Verbal fluency: semantic**<br>Control 0.43, 0.81 (0.13, 0.73)<br>Neonatal stroke -0.60, 0.95 (-1.04, 0.15) p<0.0001<br><br>**Verbal fluency: phonemic**<br>Control 0.40, 0.93 (-0.12, 0.92)<br>Neonatal stroke -0.67, 0.90 (-1.42, 0.08) p=0.008<br><br>**Oromotor sequences**<br>Control 0.31, 0.64 (0.07, 0.54)<br>Neonatal stroke -0.52, 1.25 (-1.15, 0.10)<br><br>**Sentence comprehension**<br>Control 0.19, 0.78 (-0.09, 0.48)<br>Neonatal stroke -0.35, 1.09 (-0.91, 0.21) p=0.027<br><br>**Sensorimotor functions, mean, SD, 95% CI**<br>**Finger tapping**<br>Control 0.49, 0.33 (0.35, 0.62)<br>Neonatal stroke -0.53, 1.27 (-1.16, 0.10) p=0.0007<br><br>**Imitating hand positions**<br>Control 0.57, 0.68 (0.32-0.82)<br>Neonatal stroke -0.72, 0.92 (-1.14, 0.30) p<0.0001<br><br>**Visuomotor precision: time**<br>Control 0.13, 0.83 (-0.17, 0.43)<br>Neonatal stroke -0.24, 0.97 (-0.69, 0.20) p=0.145<br><br>**Visuomotor precision: mistakes**<br>Control 0.45, 0.50 (0.27, 0.64)<br>Neonatal stroke -0.42, 1.05 (-0.90, 0.05) p=0.0002<br><br>**Manual motor sequences**<br>Control 0.50, 0.62 (0.27, 0.73)<br>Neonatal stroke -0.92, 0.95 (-1.43, 0.41) p<0.0001<br><br>**Finger discrimination**<br>Control 0.53, 0.57 (0.29, 0.77)<br>Neonatal stroke -0.77, 1.03 (-1.30, 0.24) p<0.0001<br><br>**Visuospatial functions, mean, SD, 95% CI**<br>**Design copying**<br>Control 0.36, 0.80 (0.06, 0.65)<br>Neonatal stroke -0.54, 0.97 (-1.0, 0.09) p<0.0001<br><br>**Arrows**<br>Control 0.37, 0.79 (0.05, 0.70)<br>Neonatal stroke -0.61, 1.07 (-1.16, 0.06) p=0.0004<br><br>**Block construction**<br>Control 0.29, 0.81 (-0.01, 0.58)<br>Neonatal stroke -0.45, 1.04 (-0.92, 0.03) p=0.0003<br><br>**Route finding**<br>Control 0.25, 1.05 (-0.33, 0.83)<br>Neonatal stroke -0.66, 0.80 (-1.23, 0.09) p=0.033<br><br>**Picture perception**<br>Control 0.13, 1.00 (-0.49, 0.24)<br>Neonatal stroke -0.09, 1.03 (-0.56, 0.37) p=0.341<br><br>**Memory and learning, mean, SD, 95% CI**<br>**Memory for faces**<br>Control 0.42, 0.74 (0.11, 0.73)<br>Neonatal stroke -0.41, 1.15 (-0.96, 0.15) p=0.016

Confidential: For Review Only

				**Memory for names**<br>Control 0.15, 0.92 (-0.23. 0.53)<br>Neonatal stroke -0.31, 1.09 (-0.87, 0.25) p=0.295<br><br>**Narrative memory**<br>Control 0.26, 0.80 (-0.03, 0.55)<br>Neonatal stroke -0.22, 1.16 (-0.78, 0.34) p=0.077<br><br>**Sentence repetition**<br>Control 0.49, 0.61 (0.26, 0.71)<br>Neonatal stroke -0.64, 0.96 (-1.09, 0.19) p<0.0001<br><br>**List learning**<br>Control 0.30, 0.82 (-0.16, 0.76)<br>Neonatal stroke -0.38, 1.22 (-1.32, 0.56) p=0.151<br><br>**Picture recognition**<br>Control 0.39, 0.72 (0.10, 0.69)<br>Neonatal stroke -0.36, 1.24 (-0.98, 0.25) p=0.027<br><br>**<u>Motor (hemiparesis)</u>**<br>Neonatal stroke and any hemiparesis n=19, 90%<br>Mild functional impairment n=6, 29%<br>Significant functional impairment n= 8, 38%<br>Very severe functional impairment n= 4, 19%<br><br>**<u>Epilepsy</u>**<br>Stroke n=9, 33.3%
34	Martin 2019[40] *<br><br>USA<br><br>Prospective cohort	**Population**<br>• Gestation not provided<br>• Birth years not provided<br><br>**Exposure (n=21)**<br>• Left hemisphere (n=13)<br>• Right hemisphere (n=8)<br><br>**Control (n=21)**<br>• Matched on age, sex and socioeconomic status<br>• Healthy controls<br>• Recruited from local community using adverts<br><br>**Ascertainment/ definition**<br>• Unilateral focal brain lesion (ischaemic or haemorrhagic thought to have occurred between 28 weeks' gestation and 28 days postnatally)<br>• Recruited from a neurologist in San Diego	**Outcomes**<br>• Hearing<br>• Motor (cerebral palsy)<br>• Epilepsy<br><br>**Measurement/ assessment**<br>• Auditory neglect task<br><br>**Follow-up**<br>• 6-14 years (mean 9-10 years)<br>• Completeness not specified	**<u>Time to correct response</u>**<br>**Left sided sound:**<br>Left stroke 1550 ms±580 ms<br>Control 1465 ms±666 ms *not significant*<br><br>Right stroke 1708 ms±951 ms<br>Control 1074 ms±514 ms* (p=0.043)<br><br>**Right sided sound**<br>Left stroke 1595 ms±553 ms<br>Control 1501 ms±720 ms *not significant*<br><br>Right stroke 2032 ms±1496 ms<br>Control 1291 ms±792 ms p=0.118<br><br>**<u>Number of correct auditory responses</u>**<br>**Left sided sound**<br>Left stroke 5.15±1.21<br>Control 4.62±1.26 p=0.338<br><br>Right stroke 4.25±1.67<br>Control 4.63±1.19 p=0.307<br><br>**Right sided sound**<br>Left stroke 4.31±1.18<br>Control 4.62±1.71 p=0.3<br><br>Right stroke 4.50±1.31<br>Control 5.50±0.92 p=0.05<br><br>**Seizures outside of neonatal period**<br>Stroke n=4; 19%<br><br>**Hemiparesis**<br>Stroke n=13, 70%<br><br>Right stroke n=3, 28%<br>Left stroke n=10, 77%
35	Northam 2018[37]<br><br>UK<br><br>Prospective cohort	**Population**<br>• Gestation not provided<br>• Born 1991-2001<br><br>**Exposure (n=30)**<br>• Perinatal stroke<br><br>**Control (n=40)**<br>• Matched on age, sex and maternal education<br>• Term infants<br><br>**Ascertainment/ definition**<br>• Arterial or ischaemic stroke confirmed by MRI in the neonatal period	**Outcomes**<br>• Cognitive<br>• Speech and language<br>• Motor (cerebral palsy)<br><br>**Measurement/ assessment**<br>• WASI<br>• CELF<br>• Comprehensive Test of Phonological Processing<br><br>**Follow-up**<br>• 6-18 years (mean 12.4 and 13.5)<br>• 100% follow up	**<u>Cognitive</u>**<br>**Full scale IQ mean (SD)**<br>Stroke 99 (14)<br>Control 112 (16) p<0.0001<br><br>**Mainstream education**<br>Stroke n=28, 93%<br><br>**Receiving additional education support**<br>Stroke n=12, 40%<br><br>**<u>Speech and language</u>**<br>**Expressive language score, mean (SD)**<br>Stroke 95 (17)<br>Control 108 (13) p=0.001<br><br>**Receptive language score, mean (SD)**<br>Stroke 91 (16)<br>Control 104 (14) p < 0.0001<br><br>**<u>Motor (hemiparesis)</u>**<br>Stroke n=9, 3%
36	Tillema 2008[38]<br><br>USA<br><br>Retrospective cohort	**Population**<br>• Gestation not provided<br>• Birth years not provided<br><br>**Exposure (n=10)**<br>• Left perinatal stroke<br><br>**Control (n=10)**	**Outcomes**<br>• Cognitive<br>• Epilepsy<br><br>**Measurement/ assessment**<br>• WISC-III<br>• Language activation tasks – Verb generation task whilst in an fMRI	**<u>Focal epilepsy</u>**<br>Stroke, n=6, 60%<br><br>**<u>Cognitive, mean (SD)</u>**<br>Stroke VIQ 84 (13.4)<br>Control VIQ 108 (14.2) p=0.002

Confidential: For Review Only

		• Matched on age, sex, and handedness<br>• Healthy<br>• Randomly drawn from a large database of children recruited for a different study of language development in healthy children<br><br>**Ascertainment/ definition**<br>• Middle cerebral artery ischaemic stroke	**Follow-up**<br>• 6-16 years<br>• 100% follow up	Stroke FSIQ 80 (14.1)<br>Control FSIQ 108 (11.7) p=0.001
37	Trauner 2001[39]<br><br>USA<br><br>Retrospective cohort	**Population**<br>• Gestation not reported<br>• Birth years not reported<br><br>**Exposure (n=39)**<br>• Left perinatal stroke (n=25)<br>• Right perinatal stroke (n=14)<br><br>**Control (n=54)**<br>• Matched on age and socioeconomic status<br>• Normal neurodevelopmental history<br>• Identified from clinics, community adverts, schools<br><br>**Ascertainment/ definition**<br>• Pre or perinatal onset unilateral brain damage (focal lesion) from cerebral infarction or intraparenchymal haemorrhage<br>• Identified through from clinical referrals.<br>• All confirmed by neuroimaging.<br>• Severity rated on 5-point scale adapted from Vargha-Khadem et al.<br>•	**Outcomes**<br>• Behavioural<br>• Cognitive<br>• Epilepsy<br><br>**Measurement/ assessment**<br>• Achenbach CBCL<br>• WPPSI-R (4-5 years)<br>• WISC-R (6-16 years)<br><br>**Follow-up**<br>• 4-18 years<br>• 100% follow up	**Cognitive**<br>**Full scale IQ mean (SD)**<br>Stroke 93.4 (22)<br>Control 116.2 (13) p<0.0001<br><br>Left stroke 90.1 (22)<br>Right stroke 97.4 (22) – no significant difference<br><br>**Seizures (outside of the neonatal period)**<br>Stroke n=17, 50% (missing data for 5 subjects)
**Central nervous system infections**				
38	Bedford 2001[42]<br><br>England & Wales<br><br>Prospective cohort	**Population**<br>• All gestational ages included<br>• Born 1985-1987<br><br>**Exposure (n=274)**<br>• Neonatal meningitis<br><br>**Comparison (n=1391)**<br>• Matched on age and sex<br>• Recruited through GP<br><br>**Ascertainment/ definition**<br>• Identified through clinician reporting	**Outcomes**<br>• Neuromotor disability (composite)<br>• Cognitive<br>• Hearing<br>• Vision<br>• Behaviour<br>• Seizure disorder<br><br>**Assessment/ measurement**<br>• Parental questionnaire<br>• GP questionnaire<br>• McIntyre et al. classification of disability severity<br><br>**Follow-up**<br>• 5 years<br>• 85-94% follow-up	**Neuromotor disability**<br>Meningitis, n=45, 16%<br>No meningitis, n=2, 0.1%<br><br>**Severe disability**<br>Meningitis, n=20, 7%<br>No meningitis, n=1, 0.1%<br><br>**Moderate disability**<br>Meningitis, n=50, 18%<br>No meningitis, n=20, 1%<br><br>**Mild disorder**<br>Meningitis, n=66, 24%<br>No meningitis, n=275, 20%<br><br>**No disability**<br>Meningitis, n=138, 50%<br>No meningitis, n=1095, 79%
39	Horváth-Puhó 2021[43]<br><br>Denmark and Netherlands<br><br>Retrospective matched cohort study	**Population**<br>• Gestation not specified<br>• Born 1997-2017<br><br>**Exposure**<br>• GBS meningitis (Denmark) (n=168)<br>• GBS meningitis (Netherlands) (n=198)<br><br>**Comparison**<br>• Randomly selected<br>• Matched 1:10 on sex, birth year and month, and gestation<br>• No GBS (Denmark) (n=13,689)<br>• No GBS (Netherlands) (n=4,983)<br><br>**Ascertainment/ definition**<br>• Invasive Group B Streptococcal disease by 89 days of age (most were neonatal – hence inclusion)<br>• ICD 10 codes (Denmark)<br>• CSF culture positive on national laboratory register (Netherlands)	**Outcomes**<br>• Neurodevelopmental impairment (composite)<br>• Cognitive<br>• Motor<br>• Behavioural, mental and social disorders<br>• Hearing impairment<br>• Visual impairment<br><br>**Assessment/ Measurement**<br>• ICD 10 codes<br><br>**Follow-up**<br>• Denmark 5 years, 7 years, 10 years, 15 years<br>• Netherlands 5 years, 7 years, 10 years and 11 years<br>• 95% follow-up	**Any neurodevelopmental impairment RR (95%CI)**<br>**<5 years**<br>Denmark GBS meningitis 7·80 (4·42-13·77)<br>Netherlands GBS meningitis 5·30 (2·57-10·89)<br><br>**<7 years**<br>Denmark GBS meningitis 4·69 (2·78-7·89)<br>Netherlands GBS meningitis 3·71 (1·05-6·72)<br><br>**<10 years**<br>Denmark GBS meningitis 3·47 (2·19–5·50)<br>Netherlands GBS meningitis 2·81 (1·69-4·68)<br><br>**<11 years**<br>Netherlands GBS meningitis 2·99 (1·83-4·88)<br><br>**<15 years**<br>Denmark GBS meningitis 3·15 (1·82–5·46)<br><br>**Moderate to severe neurodevelopmental impairment RR (95%CI)**<br>**<5 years**<br>Denmark GBS meningitis 8·49 (4·28-16·86)<br>Netherlands GBS meningitis 5·13 (2·24-11·79)<br><br>**<7 years**<br>Denmark GBS meningitis 5·27 (2·80-9·92)<br>Netherlands GBS meningitis n/a<br><br>**<10 years**<br>Denmark GBS meningitis 3·88 (2·15–6·99)<br>Netherlands GBS meningitis 3·05 (1·62-5·73)<br><br>**<11 years**<br>Netherlands GBS meningitis 3·34 (1·77-6·33)<br><br>**<15 years**<br>Denmark GBS meningitis 4·52 (2·35–8·67)
40	Martinez-Cruz 2008[45]	**Population**<br>• Gestation < 34 weeks<br>• Birthweight <1500g	**Outcomes**<br>• Sensorineural hearing loss	**Meningitis**<br>Sensorineural hearing loss: n=15; 10.3%<br>No Sensorineural hearing loss: n=7; 2.6%

Confidential: For Review Only

	Mexico<br><br>Retrospective case control	• Born 1990-2005<br><br>**Exposure (n=22)**<br>• Neonatal meningitis<br><br>**Comparator (n=374)**<br>• No meningitis<br><br>**Ascertainment/ definition**<br>• Meningitis not defined	**Assessment/ measurement**<br>• Brainstem Auditory Evoked Potentials<br>• Transient Auditory Evoked Otoacoustic Emissions<br>• Tympanometry<br>• Free Field Audiometry<br>• Pure tone audiometry<br>• Behavioural hearing evaluation<br><br>**Follow-up**<br>• 7- 11 years<br>• 100% follow-up	**Odds of previous neonatal meningitis if sensorineural hearing loss**<br>OR 4.368, 95% CI (1.7, 10.9) p= 0.002
41	Stevens 2003[44]<br><br>England & Wales<br><br>Prospective cohort study	**Population**<br>• Term born infants<br>• Born 1985-1987<br><br>**Exposure (n=111)**<br>• Meningitis<br><br>**Comparison (n=162)**<br>• Matched on hospital of birth, birthweight and sex<br>• Hospital control (n=113)<br>• GP control (n=49)<br><br>**Ascertainment/ definition**<br>• CSF positive culture	**Outcomes**<br>• Disability and functional impairment (composite)<br>• Cognitive<br>• Motor<br>• Vision<br>• Hearing<br><br>**Assessment/ measurement**<br>• WISC-III<br>• Movement ABC<br>• Blinded examination<br>• Hearing screening<br>• Sonksen-Silver acuity system<br><br>**Follow-up**<br>• 9-10 years<br>• 67% follow-up of meningitis group	**<u>Cognitive</u>**<br>**IQ, mean (95% CI)**<br>Meningitis, 88.8 (85, 92)<br>Hospital control, 99.4 (97, 102)<br>GP control, 99.6 (95, 103)<br><br>**<u>Motor</u>**<br>**mABC score, mean (95% CI)**<br>Meningitis 7.1 (5.9, 8.5)<br>Hospital controls 5.0 (4.3, 5.8)<br>GP controls 4.0 (2.9, 5.4)<br><br>**<u>Severe disability/ functional impairment</u>**<br>Meningitis, n=12, 10.8%<br>Hospital control, n=0, 0%<br>GP control, n=0, 0%<br><br>**<u>Moderate disability/ functional impairment</u>**<br>Meningitis, n=10, 9%<br>Hospital control, n=2, 1.8%<br>GP control, n=0, 0%<br><br>**<u>Mild disability/ functional impairment</u>**<br>Meningitis, n=19, 17.1%<br>Hospital control, n=13, 11.5%<br>GP control, n=8, 16%<br><br>**<u>No disability or functional impairment</u>**<br>Meningitis, n=70, 63.1%<br>Hospital control, n=98, 86.7%<br>GP control, n=41, 84%<br><br>**<u>Hearing loss (unilateral or bilateral sensorineural hearing loss or requiring hearing aids)</u>**<br>Meningitis, n=4, 3,6%<br>Hospital control, n=0, 0%<br>GP control, n=0, 0%<br><br>**<u>Visual impairment (bilateral)</u>**<br>Meningitis, n= 18, 17% (6 unassessed because of their disability)<br>Hospital control, n=21, 18.5%<br>GP control, n=4, 8%<br><br>**<u>Visual impairment (unilateral)</u>**<br>Meningitis, n= 10, 9.9% (6 unassessed because of their disability)<br>Hospital control, n=8, 7%<br>GP control, n=2, 4%<br><br>**<u>Seizures outside of the neonatal period</u>**<br>Meningitis, n=6, 5.4%<br>Hospital control, n=2, 1.8%<br>GP control, n=0, 0%
Hypoxic-ischaemic encephalopathy				
42	3383 Koc 2016[24]<br><br>Turkey<br><br>Retrospective cohort	**Population**<br>• Gestation < 32 weeks<br>• Birthweight < 1500g<br>• Born 2001<br><br>**Exposure (n=9)**<br>• Perinatal asphyxia<br><br>**Comparator (n=81)**<br>• No asphyxia<br><br>**Ascertainment/ definition**<br>• Perinatal asphyxia diagnosed on: fetal pH, Apgar score, and neonatal cerebral and multiorgan dysfunction	**Outcomes**<br>• Cognitive<br><br>**Assessment/ measurement**<br>• WISC-R<br>• Performed by blinded psychologist<br><br>**Follow-up**<br>• 5-8 years<br>• 100% follow-up	**<u>Cognitive</u>**<br>**WISC-R IQ Score (combined verbal and performance scores) <85**<br>Perinatal asphyxia n=8, 89%<br>No asphyxia n=24, 30%<br>p=0.001
43	Lee-Kelland 2019[46]*<br><br>United Kingdom<br><br>Retrospective cohort study	**Population**<br>• Gestation ≥ 36 weeks<br>• Born 2008-2010<br><br>**Exposure (n=29)**<br>• Moderate-severe HIE without subsequent cerebral palsy<br><br>**Comparator (n=20)**<br>• Matched on age, sex and social class<br>• Born without HIE<br><br>**Ascertainment/ definition**<br>• Received therapeutic hypothermia based on TOBY trial criteria	**Outcomes**<br>• Cognitive<br>• Motor<br>• Speech and language<br>• Behaviour<br><br>**Assessment/ measurement**<br>• WISC IV (blinded)<br>• Movement ABC 2<br>• Strengths and difficulties questionnaire<br><br>**Follow-up**<br>• 6-8 years<br>• 61% follow-up	**<u>Cognitive</u>**<br>**Full scale IQ, mean (SD)**<br>HIE 91 (10.37)<br>No HIE 105 (13.41)<br>Mean difference −13.62 95% CI (−20.53 to −6.71) p<0.001<br><br>**Perceptual reasoning, mean (SD)**<br>HIE 89 (11.15)<br>No HIE 103 (12.49)<br>Mean difference −13.9 95% CI (−20.78 to −7.09) p<0.001<br><br>**Working memory, mean (SD)**<br>HIE 94 (13.76)<br>No HIE 102 (13.82)<br>Mean difference −8.2 95% CI (−16.29 to −0.17) p=0.04

Confidential: For Review Only

				**Processing speed, mean (SD)**<br>HIE 96 (13.76)<br>No HIE 107 (17.59)<br>Mean difference −11.6 95% CI (−20.69 to −2.47) p=0.01<br><br>**Additional classroom support**<br>HIE n=10, 34%<br>No HIE n=1, 5%<br>OR: 10.0, 95%CI 1.16 to 86.0<br><br>**Special educational needs**<br>HIE n=1, 3.4%<br>No HIE n=0, 0%<br><br>**<u>Motor</u>**<br>**MABC-2 score, mean (SD)**<br>HIE 7.9 (3.26)<br>No HIE 10.2 (2.86)<br>Mean difference −2.12 95% CI (−3.93 to −0.30) p=0.02<br><br>**<u>Speech and language</u>**<br>**Verbal comprehension, mean SD)**<br>HIE 94 (8.79)<br>No HIE 103 (10.09)<br>Mean difference −8.8 95% CI (−14.25 to −3.34) p=0.002<br><br>**<u>Behaviour</u>**<br>**Total difficulties, median (IQR)**<br>HIE 12 (6.5–13.5)<br>No HIE 6 (2.25–10) P=0.005<br><br>**Emotional problems, median (IQR)**<br>HIE 2 (1–4.5)<br>No HIE 0.5 (0–2.75) P=0.03<br><br>**Hyperactivity, median (IQR)**<br>HIE 2 (1–3)<br>No HIE 1 (0–2) P=0.06<br><br>**Conduct problems, median (IQR)**<br>HIE 4 (2.5–6.5)<br>No HIE 3 (1–5) p=0.06<br><br>**Peer problems, median (IQR)**<br>HIE 0 (0–2.5)<br>No HIE 0 (0–1) p=3.56 Ω (potential error in manuscript table)<br><br>**Prosocial, median (IQR)**<br>HIE 9 (7.5–10)<br>No HIE 9 (8.25–10) p=0.13<br><br>**Impact score, median (IQR)**<br>HIE 0 (0–2.5)<br>No HIE 0 (0–2.0) p=0.31
44	Tonks 2019[47]*<br><br>United Kingdom Prospective cohort study	**Population**<br>• Gestation ≥36 weeks<br>• Born 2008-2011<br>• English as primary language<br><br>**Exposure (n=29)**<br>• Moderate-severe HIE without subsequent cerebral palsy<br><br>**Comparator (n=20)**<br>• Matched on age, sex and social class<br>• Recruited from schools in the area<br>• Born without HIE<br><br>**Ascertainment/ definition**<br>• Received therapeutic hypothermia based on TOBY trial criteria	**Outcomes**<br>• Cognitive<br>• Neuropsychological<br><br>**Assessment/ measurement**<br>• Conner's continuous performance test<br>• NEPSY-II block construction test<br>• NEPSY-II arrows' test<br><br>**Follow-up**<br>• 6-8 years<br>• 77% follow-up	**<u>Attention</u>**<br>**Hit response time**<br>**HIE**<br>84.1 percentile mean rank 27;<br>Proportion performing below 2 SD 32%<br><br>**Comparator**<br>67.3 percentile mean rank 17.89; p = .024<br>Proportion performing below 2 SD 11%<br><br>**Hit response time standard error**<br>**HIE**<br>standard error mean rank 26.8<br>Proportion performing below 2 SD 18%<br><br>**Comparator**<br>standard error mean rank 18.2; p = 0.032<br>Proportion performing below 2 SD 11%<br><br>**Hit response time by block**<br>**HIE**<br>Mean 49.1, SD 23.9<br><br>**Comparator**<br>Mean 61.9, SD 18.4; p = 0.047<br><br>**<u>Visual discrimination</u>**<br>**<u>HIE</u>**<br>Below 1 SD 10%<br><br>**<u>Comparator</u>**<br>Below 1 SD 5%<br>HIE vs comparator scores, p = 0.049<br><br>**<u>Visuo-spatial mental rotation task</u>**<br>**<u>HIE</u>**<br>Below 1 SD 17%<br><br>**<u>Comparator</u>**<br>Below 1 SD 5%<br>HIE vs comparator scores, p = 0.034

Confidential: For Review Only

Confidential: For Review Only

Supplement 4: Risk of bias table

overlapping data; Clinical Evaluation of Language Fundamentals (CELF); Cystic Periventricular leukomalacia (cPVL); Intelligence Quotient (IQ); Intraventricular haemorrhage (IVH); Mental Developmental Index (MDI); Neonatal Intensive Care Unit (NICU); Psychomotor Development Index (PDI); Periventricular leukomalacia (PVL); Spontaneous Intestinal Perforation (SIP); Wechsler Intelligence Scale for Children (WISC); White Matter Injury (WMI);

Preterm brain injury: cohort studies

	Selection (*satisfactory; No =not satisfactorily done; n/a)				Comparability (*satisfactory; No =not satisfactorily done; n/a)		Exposure/ Outcome (*satisfactory; No =not satisfactorily done; n/a)			Subtotal assessment			Total score: 0-3 high risk of bias; 4-6 moderate risk of bias 7-9 low risk of bias	Additional comments
	1	2	3	4	1a	1b	1	2	3	Selection (0-1=Poor; 2=Fair; 3+ Good)	Comparability (0=poor; 1=fair; 2+=good)	Exposure / outcome (0=poor; 1=fair; 2+=good)		
Adant 2019	No	*	*	* (excluded those with congenital anomalies)	*	*	No	*	No	Good	Good	Fair	6	Population not representative as focus of study was spontaneous intestinal perforation. Infants without IVH didn't have brain injury excluded per se (but didn't have IVH 3-4 on imaging). Matched on gender, gestational age, date of birth. Multiples matched to sibling without SIP. Excluded those with necrotising enterocolitis, mechanical obstruction or congenital anomalies. Adjusted for gender, gestation, birthweight, SIP and IVH.<br><br>Independent outcome assessment but not blinded; telephone survey of parents. High numbers lost to follow-up. Table 3 contains errors with respect to outcomes (MDI and PDI mislabelled as motor and cognitive respectively).

Confidential: For Review Only

Beaino 2010#	*	*	No	* (cerebral palsy could not be present at birth)	*	*	*	*	*	Good	Good	Good	8	3% of infants did not have a cranial ultrasound, a further 11% had only one cranial ultrasound during neonatal period - therefore ascertainment of exposure may be compromised<br><br>Model A adjusted for:<br>• obstetric factors<br>• cerebral lesions<br>Model B adjusted for:<br>• obstetric factors<br>• neonatal factors<br><br>Model C was the same as model B for those without cPVL or Intraparenchymal haemorrhage<br><br><85% follow-up for enrolled infants but clear description of those lost to follow-up and no significant differences with respect to ultrasound brain injury findings between groups
Brouwer 2012	No	No	*	* (given the types of outcomes assessed)	No	No	No	*	*	Fair	Poor	Good	4	Study of a select group i.e. those with IVH requiring neurosurgical intervention.<br>No description of setting, how patients were enrolled, how many were excluded<br>No description of how control group was derived, or what era they were from.<br>Only some infants (those <30weeks) were matched on gestation, birthweight, sex to controls.<br>Different intelligence tests used at follow-up. >80% completion rate of Child Behaviour Checklist and teacher report form by parents and teachers

Confidential: For Review Only

Campbell 2021	*	*	*	* (given the types of outcomes assessed)	*	*	*	*	No	Good	Good	Good	8	Males and those born at 23-24 weeks gestation were overrepresented in the IVH WMI group.<br>Adjusted for gestation, birthweight Z score, sex, maternal education, bronchopulmonary dysplasia, sepsis, necrotising enterocolitis (Bell stage 2-3) and severe retinopathy of prematurity.
Cheong 2018	*	*	*	No (visual or hearing impairment could be congenital)	*	*	*	*	*	Good	Good	Good	8	Adjusted for era of birth, antenatal corticosteroid exposure, inborn status, gestation, sex, multiple birth, birthweight Z score, surfactant use, IVH grade 3 or 4 (in cPVL), cPVL (in IVH grade 3-4), bronchopulmonary dysplasia, postnatal corticosteroid use, necrotising enterocolitis (stage 2 or worse), surgery in the newborn period, and retinopathy of prematurity (stage 3 or worse).
Chou 2020	*	*	*	* (given the types of outcomes assessed)	No	*	*	*	No	Good	Fair	Good	7	Matched and adjusted for, urbanisation and parental occupation.<br><br>No information about missing data or completeness of follow-up

Confidential: For Review Only

Davidovitch 2020	*	*	*	* (given the types of outcomes assessed)	No	*	*	*	No	Good	Fair	Good	7	Only low birthweight infants included (therefore birthweight partially accounted for). Unmatched.<br>No information about excluding brain injury from comparators e.g. comparing those with IVH grade 3-4 to those without could include those with IVH 1-2; both groups could also include infants with other types of brain injury.<br>Missing data not presented or accounted for. Adjusted the composite brain injury group (which included retinopathy of prematurity in its definition) for gestation, maternal diabetes, small for gestational age, year of birth, bronchopulmonary dysplasia, and receipt of postnatal steroids.
Doyle 2000 #	*	*	*	* (given the types of outcomes assessed)	No	No	*	*	*	Good	Poor	Good	7	IVH and no IVH groups not matched for gestation or birthweight, no adjustment for these variables appears to have been done.<br><br>Relatively old cohort (most did not receive surfactant), comparator group only includes infants born in the 1980s. Not representative due to time-period of care.
Hintz 2018	*	*	*	* (given the types of outcomes assessed)	*	*	*	*	*	Good	Good	Good	9	Assessed interobserver reliability of central imaging readers.<br><br>Unmatched<br><br>Adjusted for gestation, race, sex, multiple gestation, maternal education, sepsis, bronchopulmonary dysplasia, postnatal steroids, surgery for patent ductus arteriosus, necrotising enterocolitis, retinopathy of prematurity.<br><br>Only 83% follow-up of survivors but those lost to follow-up are accounted for.

Confidential: For Review Only

Hirovonen 2017	*	*	*	* (given the types of outcomes assessed)	*	*	*	*	*	Good	Good	Good	9	Excluded infants who died at <1 year of age, infants with major congenital anomalies, and those with missing data.<br><br>Characteristics of those with brain injury not presented.<br><br>No breakdown by severity of brain injury because that level of detail was not available in the database.<br><br>No matching but there is stratification by gestation and adjustment for: maternal characteristics, pregnancy characteristics, delivery characteristics, sex, gestation, birthweight, Apgar score at 1-minute, umbilical artery pH, resuscitation provided, NICU admission, receipt of phototherapy, ventilator requirement, antibiotic receipt, respiratory distress syndrome, sepsis, seizures, hyperbilirubinaemia.
Hollebrandse 2021	*	*	*	* (given the types of outcomes assessed)	*	*	*	*	*	Good	Good	Good	9	Gestation similar across all groups and other baseline perinatal characteristics similar across groups.<br><br>Preterm brain injury and no brain injury group not matched. Unclear if IVH and no IVH group had other brain injuries excluded or may have had more than one injury type (e.g. PVL).<br>Impact of epoch/ era of birth explored and adjusted for.
Hreinsdottir 2018	*	*	*	No (visual impairment could have been congenital)	*	*	*	*	No	Good	Good	Good	7	Unsure if comparator group in logistic regression includes those with IVH 1-2. Adjusted for gestation, birthweight, retinopathy of prematurity, sex, cognitive score, cerebral palsy.

Jansen 2020	*	*	*	* (given the types of outcomes assessed)	No	No	*	*	No	Good	Poor	Good	6	Excluded infants with congenital abnormalities, metabolic disorders or neonatal meningitis.
Kaur 2020	*	*	*	No (visual or hearing impairment could be congenital)	No	No	*	*	No	Good	Poor	Good	5	Unmatched. Compared IVH with all infant without haemorrhage (of all gestations).
Kiechl-Kohlendorfer 2013	*	*	*	* (given the types of outcomes assessed)	*	*	*	No	No	Good	Good	Fair	7	Low numbers of infants included. Outcomes assessed at 1 year - likely not long enough for robust assessment of neurodevelopmental outcomes; <85% follow-up and no detailed description of those lost to follow up - though authors do state that there were no significant differences between those followed up and those lost to follow up.
Klebermass-Schrehof 2012	*	*	*	No (could have had congenital blindness)	*	*	*	*	No	Good	Good	Good	7	Adjusted for gestation. No clear description of number lost to follow-up, though mentions that follow-up rate at 5.5 years was 54-61%.
Koc 2016	*	*	No	* (given the types of outcomes assessed)	No	No	*	*	No	Good	Poor	Good	5	Small numbers included. No breakdown of characteristics of those with brain injury. No description of IVH grading used or schedule of ultrasound exams; no description of criteria for establishing perinatal asphyxia, number lost to follow-up not stated.
Neubauer 2008	*	n/a	*	No (deafness or blindness could have been congenital)	*	*	*	*	*	Fair	Good	Fair	7	Neurodevelopmental assessors not blinded; follow-up rate <85% but paper does give description of those lost to follow-up

Confidential: For Review Only

Piris Borregas 2019	*	*	*	* (excluded infants with congenital malformations)	No	No	*	*	No	Good	Poor	Good	6	Only those followed up to 7 years included.<br><br>Excluded infants who died before 36 weeks corrected age, with major malformations, or those with missing data.<br><br>Unclear if independent odds ratio includes adjustment for covariates.<br><br>Unclear if those without ‘severe brain injury’ had other types of brain injury.
Pittet 2019	*	*	*	* (excluded infants with congenital malformations)	No	*	*	*	*	Good	Fair	Good	8	Excluded infants with congenital malformations affecting neurodevelopment and infants from centres without 5 years of follow-up cognitive testing.<br><br>Unclear if other types of brain injury excluded from comparator group.<br><br>Adjusted for gender and socioeconomic status. No significant difference in cognitive outcome between extreme preterms and those 28-30 weeks’ gestation. Gestation not adjusted for.
Sherlock 2005#	*	*	*	No (deafness or blindness could have been congenital)	No	No	*	*	*	Good	Poor	Good	6	Comparability of IVH vs. no IVH cohorts not clear - not enough information to determine if groups were comparable with respect to gestational age or birthweight
Tymofiyeva 2018	*	*	*	* (given the types of outcomes assessed)	No	No	*	*	No	Good	Poor	Good	6	Excluded infants with congenital malformations/ syndromes, congenital infections, or those who were too unstable for MR imaging. The last exclusion criteria in particular could limit generalisability quite considerably.<br><br>Unclear about the validity of grouping the attention scores across different assessment tools together into a dichotomous variable for attention.

Van De Bor 2004	*	*	*	* (excluded those with major congenital malformations)	*	*	No	No	*	Good	Good	Fair	7	IVH vs. no IVH cohorts comparable with respect to gestation; some differences in gender composition but paper states this was controlled for in the analysis. Primary outcome entirely self-reported. Outcomes reported at 14 years.
Van Den Hout 2000	* (except for HIE exposure group)	*	*	* (excluded those with congenital anomalies)	No	No	*	*	*	Good	Poor	Good	7	Low numbers and relatively old cohort. Relative gender imbalance in IVH group compared to those with normal scans or PVL. IVH group also 1.4 weeks more premature than 'normal scan' group.
Vollmer 2003#	*	*	*	No (deafness or blindness could have been congenital)	*	No	*	*	*	Good	Fair	Good	7	Note change in version of Weschler scale during follow-up period. Authors state no difference in mean IQ after change. Baseline characteristics of groups with and without brain injury not given; no indication of matching or adjustment for factors other than gestation.
Vollmer 2006a#	*	*	*	No (deafness or blindness could have been congenital)	*	*	*	*	*	Good	Good	Good	8	Note gender imbalance in cohort as a whole (M>F), but male: female ratio in each group appears similar.<br>No matching or adjustment for covariates.<br><br><85% follow-up but clear description of those lost and appears no significant differences.
Vollmer 2006b#	*	*	*	No (deafness or blindness could have been congenital)	No	No	*	*	No	Good	Poor	Good	5	Marked gender imbalance in ventricular dilatation group. Lower birthweight and gestation in groups with abnormal cranial ultrasound. No indication of matching or adjustment.<br><br><85% follow-up and the limited description of those lost to follow-up indicates that these babies were of lower birthweight and gestation.

Confidential: For Review Only

Whitaker 2011	*	*	*	* (given the types of outcomes assessed)	*	*	(No)	*	*	Good	Good	Good	8	Severely disabled survivors (n=33) were excluded.<br><br>Half had later ultrasounds (just before discharge).<br><br>No breakdown of the characteristics of the exposed and comparator groups – unable to assess how comparable they are.<br><br>Adjusted for: maternal social risk, sex, gestation, fetal growth ratio, multiplicity, maternal smoking status, maternal alcohol status, labour onset, presentation at birth, base excess on first postnatal blood gas, thyroid status, hypocapnia, hypoxia, systolic hypotension, prolonged ventilation.<br><br>Primary outcome assessment reliant on parental report, albeit via structured interview with some evidence for validity. Interviewers were blinded to the child's history. Parents were blinded to the study hypothesis.<br><br>Less than 85% follow-up (psychiatric interviews in 51% of survivors) however clear descriptions of groups with and without psychiatric evaluation given in table 2 and little apparent difference between groups.
**Preterm brain injury: case-control studies**														
	1 Case definition	2 Representativeness of cases	3 Selection of controls	4 Definition of controls	1a	1b	1 Ascertainment of exposure	2 Same method of ascertainment for cases and controls	3 Non-response rate	(0-1=Poor; 2=Fair; 3+ Good)	y (0=poor; 1=fair; 2+=good)	(0=poor; 1=fair; 2+=good)	Total score: 0-3 high risk of bias; 4-6 moderate risk of bias 7-9 low risk of bias	Additional comments

Martinez-Cruz 2008 (IVH)	*	*	*	*	*	No	*	*	No	Good	Fair	Good	7	Appears to be case-control design hence star ratings are as per case control rating sheet. Controls not well matched for birth weight. No description of whether full information on exposures could be obtained for all cases/controls e.g. missing records etc.
Perinatal stroke: cohort studies														
	Selection (*satisfactory; No =not satisfactorily done; n/a)				Comparability (*satisfactory; No =not satisfactorily done; n/a)		Exposure/ Outcome (*satisfactory; No =not satisfactorily done; n/a)			Subtotal assessment			Total score: 0-3 high risk of bias; 4-6 moderate risk of bias 7-9 low risk of bias	Additional comments
	1	2	3	4	1a	1b	1	2	3	Selection (0-1=Poor; 2=Fair; 3+ Good)	Comparability (0=poor; 1=fair; 2+=good)	Exposure / outcome (0=poor; 1=fair; 2+=good)		
Ballantyne 2007	No	No	*	*	No	*	No	*	No	Fair	Fair	Fair	4	No description of derivation of exposed cohort - whether single institute or multicentre, whether same community as non-exposed group or not.<br><br>Predominance of right-handed children amongst controls otherwise similar baseline characteristics. Note male preponderance in exposed group and female preponderance in non-exposed<br><br>No matching or adjustment for confounders.<br><br>No description of who performed outcome assessment, whether blinded and independent.
Ballantyne 2008	*	*	*	No	No	*	*	*	No	Good	Fair	Good	6	Excluded children with brain lesions from other causes e.g. head trauma, tumours

Confidential: For Review Only

														Gestational age of exposed cohort ranged from 32 to 40 weeks. No statement as to whether control group were matched on this. Note preponderance of males in stroke group and females in control group.<br><br>In study 1, significant numbers of participants did not complete the planned developmental assessments - across exposed and control groups, completeness ranged from 50% for WISC-R to 69% for CELF-R.
Gold 2014	No	No	*	*	No	*	*	*	*	Fair	Fair	Good	6	No description of how subjects were selected or recruited from neurology clinics. Nonexposed group selected from a different source. No description of gestational age of subjects or of controlling for this. Matched for age at follow up, sex, socioeconomic group and maternal education.<br><br>Excluded infants with bilateral lesions, a history of hypoxic ischemic encephalopathy, central nervous system infection, in-utero drug exposure, significant closed head injury, or any other condition that might have caused brain damage other than from the stroke.
Kolk 2011	*	*	*	*	No	*	*	*	No	Good	Fair	Good	7	No description of gestational age of subjects or of controlling for this. Difficult to ascertain completeness of follow-up from paper. Adjusted for age of outcome assessment.
Martin 2019	*	*	*	*	No	*	*	*	*	Good	Fair	Good	8	Excluded infants with bilateral lesions, hearing impairment, or a history of a problem that may have caused more global brain damage (e.g. meningitis, closed head injury, hypoxic-ischemic encephalopathy). Matched on age, sex and socioeconomic status

Northam 2018	*	No	*	*	*	*	*	*	*	Good	Good	Good	8	No description of source of unexposed cohort. Matched on age, sex, and maternal education.
Tillema 2008	*	*	*	*	No	*	*	*	No	Good	Fair	Good	7	Exposed and comparator groups not matched for gestation, but were matched for age, sex and handedness. 17 subjects included initially but 7 of these excluded for various reasons meaning that neurodevelopmental outcome data/Weschler scores only presented for 10 of 17.
Trauner 2013	*	*	*	*	No	No	No	*	No	Good	Poor	Fair	5	Excluded infants if bilateral or multifocal lesions identified, history of meningitis, or history of antenatal drug exposure<br><br>Matched on age and socioeconomic status<br><br>No baseline characteristics given to establish comparability of exposed and comparator cohorts. Likely comparable with regards to gestation based on stated inclusion criteria. Main outcome measure based on parental questionnaire - no direct linguistic assessments done, however may not have been feasible/appropriate in such a young cohort. No information on response rate/loss to follow-up.<br><br>IQ used as covariate<br><br>IQ combined across the age range and assessed with two different tools. This assumes IQ is fixed which may not be true.
Central nervous infections: cohort studies														

Confidential: For Review Only

	Selection (*satisfactory; No =not satisfactorily done; n/a)				Comparability (*satisfactory; No =not satisfactorily done; n/a)		Exposure/ Outcome (*satisfactory; No =not satisfactorily done; n/a)			Subtotal assessment			Total score: 0-3 high risk of bias; 4-6 moderate risk of bias 7-9 low risk of bias	Additional comments
	1	2	3	4	1a	1b	1	2	3	Selection (0-1=Poor; 2=Fair; 3+ Good)	Comparability (0=poor; 1=fair; 2+=good)	Exposure / outcome (0=poor; 1=fair; 2+=good)		
Bedford 2001#	*	*	*	No	*	*	No	*	*	Good	Good	Good	7	Matched on sex and age.<br><br>Study focuses on meningitis in infancy but also presents outcomes after neonatal meningitis.<br><br>Did not exclude children with other comorbidities e.g. congenital conditions associated with neurodevelopmental impairment. Exposed cases derived from same cohort as Stevens 2003. Outcome assessment based on parent or GP report with no formal neurodevelopmental assessment.
Horváth-Puhó 2021	*	*	*	No	*	*	*	*	*	Good	Good	Good	8	Invasive Group B Streptococcal infection diagnosed in the first 89 days (however most of these were neonatal, particularly in the first week of life (45%) hence inclusion.<br><br>Matched 1:10 on sex, birth year and month, and gestation.<br>Neurodevelopmental impairment defined differently in each cohort.<br>Missing data accounted for and its impact explored.

Stevens 2003#	(*)	(*)	*	No	*	*	*	*	No	Good	Good	Good	7	Exposed cohort based on recall of consultant paediatricians filling out monthly returns thus may be biased towards more severe or otherwise memorable cases. Some in comparator group selected from a different hospital than exposed cohort.<br><br>Matched on hospital of birth, birth weight and sex.<br><br>Results stratified by birthweight<br><br>Significant rate of loss to follow-up.
**Central nervous system infections: case control studies**														
	1 Case definition	2 Representativeness of cases	3 Selection of controls	4 Definition of controls	1a	1b	1 Ascertainment of exposure	2 Same method of ascertainment for cases and controls	3 Non-response rate	(0-1=Poor; 2=Fair; 3+ Good)	y (0=poor; 1=fair; 2+=good)	(0=poor; 1=fair; 2+=good)	Total score: 0-3 high risk of bias; 4-6 moderate risk of bias 7-9 low risk of bias	Additional comments
Martinez-Cruz 2008	*	*	*	*	No	No	*	*	No	Good	Poor	Good	6	Excluded those with history of parental consanguinity or TORCH infections.<br><br>Number of those with and without meningitis who may have had other types of brain injuries not specified – unable to assess overlap/ impact of meningitis alone.<br><br>Odds ratio presented for meningitis does not appear to be crude so potential adjustment for confounding factors but no description of this in the methods section.<br><br>No description of proportion of missing data.
**Hypoxic-ischaemic encephalopathy: cohort studies**														

Confidential: For Review Only

	Selection (*satisfactory; No =not satisfactorily done; n/a)				Comparability (*satisfactory; No =not satisfactorily done; n/a)		Exposure/ Outcome (*satisfactory; No =not satisfactorily done; n/a)			Subtotal assessment			Selection (*satisfactory; No =not satisfactorily done; n/a)	Additional comments
	1	2	3	4	1a	1b	1	2	3	Selection (0-1=Poor; 2=Fair; 3+ Good)	Comparability (0=poor; 1=fair; 2+=good)	Exposure / outcome (0=poor; 1=fair; 2+=good)	Total score: 0-3 high risk of bias; 4-6 moderate risk of bias 7-9 low risk of bias	
Koc 2016	No	*	*	*	No	No	*	*	No	Fair	Poor	Good	5	Representativeness not clear as no description given of babies who did not complete follow-up at the study institution. No apparent adjustment for gestation or other covariates. Pre-therapeutic hypothermia era.<br><br>Small number, no breakdown of characteristics or other neurodevelopmental outcomes by brain injury<br><br>Number of those with and without birth asphyxia who had other types of brain injuries e.g. IVH not specified.
Lee-Kelland 2019	No	*	*	*	*	*	*	No	No	Good	Good	Good	6	Excluded those who underwent therapeutic hypothermia outside of the standard criteria, infants with metabolic disorders and non-English speaking infants.<br><br>Matched on age, sex and social class.
Tonks 2019	*	No	*	*	No	*	*	*	No	Good	Fair	Good	6	Included cases had no diagnoses other than encephalopathy.<br>Excluded infants with neurological issues other than encephalopathy. Matched on age, sex and socioeconomic status.

Confidential: For Review Only

Study	Events	Total	Incidence	95% CI	Weight
Ballantyne, 2008	11	29	0.379	[0.207; 0.577]	22.2%
Kolk, 2011	9	21	0.429	[0.218; 0.660]	19.0%
Martin, 2019	4	21	0.190	[0.054; 0.419]	23.1%
Tilema, 2008	6	10	0.600	[0.262; 0.878]	12.5%
Trauner, 2001	17	34	0.500	[0.324; 0.676]	23.1%
**Overall**			**0.401**	**[0.268; 0.533]**	**100.0%**

$I^2 = 56\%$, $\tau^2 = 0.0124$, $p = 0.06$

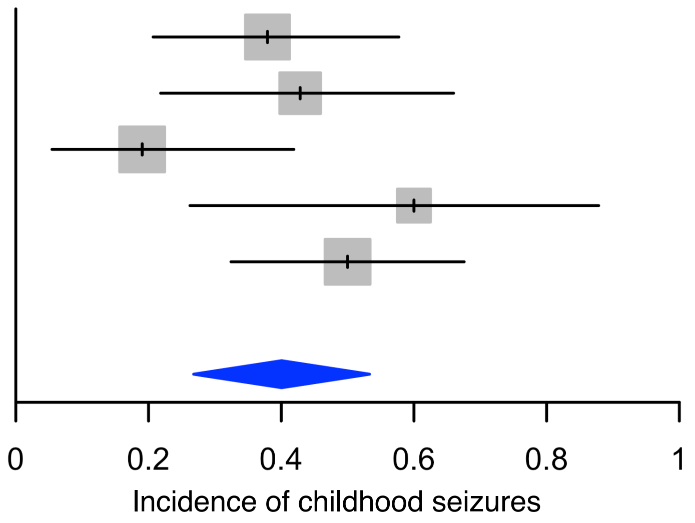

Study	Events	Total	Incidence	95% CI	Weight
Ballantyne, 2008	18	29	0.621	[0.423; 0.793]	19.9%
Gold, 2004	16	27	0.593	[0.388; 0.776]	19.6%
Kolk, 2011	19	21	0.905	[0.696; 0.988]	21.3%
Martin, 2019	13	21	0.619	[0.384; 0.819]	19.0%
Northam, 2017	9	30	0.300	[0.147; 0.494]	20.3%
**Overall**			**0.610**	**[0.392; 0.829]**	**100.0%**

$I^2 = 88\%$, $\tau^2 = 0.0545$, $p < 0.01$

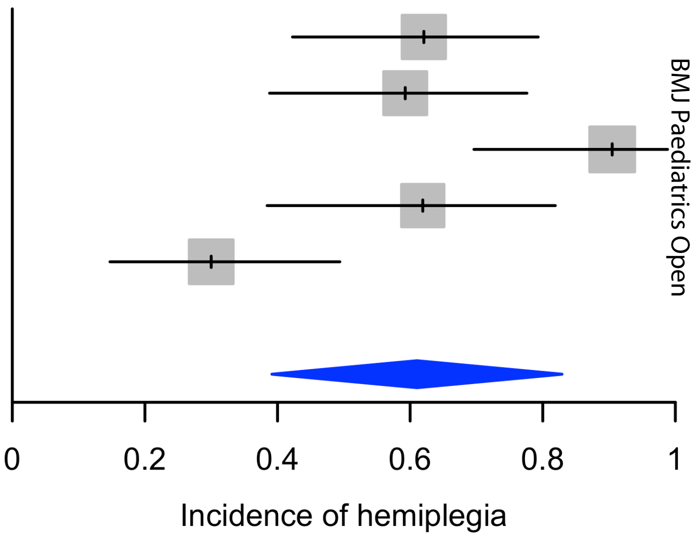

	Perinatal stroke			Control				Mean Difference
**Study or Subgroup**	**Mean**	**SD**	**Total**	**Mean**	**SD**	**Total**	**Weight**	**IV, Random, 95% CI**
Ballantyne 2008	82.3	20.1	29	111.4	13.7	38	49.0%	-29.10 [-37.61, -20.59]
Northam 2017	91	16	30	104	14	40	51.0%	-13.00 [-20.18, -5.82]
**Total (95% CI)**			**59**			**78**	**100.0%**	**-20.88 [-36.66, -5.11]**

Heterogeneity: $Tau^2 = 113.45$; $Chi^2 = 8.02$, df = 1 (P = 0.005); $I^2 = 88\%$
Test for overall effect: Z = 2.59 (P = 0.009)

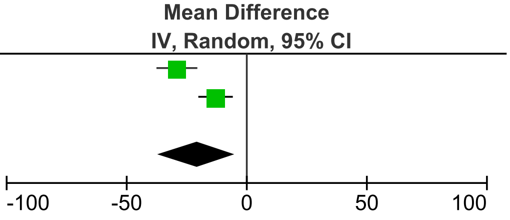

Confidential: For Review Only

	Perinatal stroke			Control				Mean Difference
**Study or Subgroup**	**Mean**	**SD**	**Total**	**Mean**	**SD**	**Total**	**Weight**	**IV, Random, 95% CI**
Ballantyne 2008	78.4	16	29	105.8	11.9	38	50.3%	-27.40 [-34.34, -20.46]
Northam 2017	95	17	30	108	13	40	49.7%	-13.00 [-20.30, -5.70]
**Total (95% CI)**			**59**			**78**	**100.0%**	**-20.25 [-34.36, -6.13]**

Heterogeneity: Tau² = 90.47; Chi² = 7.85, df = 1 (P = 0.005); I² = 87%

Test for overall effect: Z = 2.81 (P = 0.005)

Mean Difference
IV, Random, 95% CI

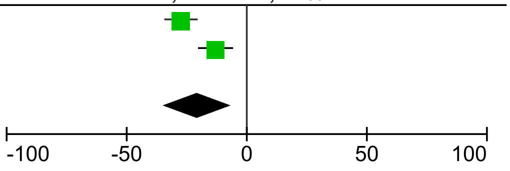

Confidential: For Review Only
